# A multiparametric niche-like drug screening platform in acute myeloid leukemia

**DOI:** 10.1038/s41408-022-00689-3

**Published:** 2022-06-24

**Authors:** Reinaldo Dal Bello, Justine Pasanisi, Romane Joudinaud, Matthieu Duchmann, Bryann Pardieu, Paolo Ayaka, Giuseppe Di Feo, Gaetano Sodaro, Clémentine Chauvel, Rathana Kim, Loic Vasseur, Laureen Chat, Frank Ling, Kim Pacchiardi, Camille Vaganay, Jeannig Berrou, Chaima Benaksas, Nicolas Boissel, Thorsten Braun, Claude Preudhomme, Hervé Dombret, Emmanuel Raffoux, Nina Fenouille, Emmanuelle Clappier, Lionel Adès, Alexandre Puissant, Raphael Itzykson

**Affiliations:** 1 Université Paris Cité, Génomes, biologie cellulaire et thérapeutique U944, INSERM, CNRS, F-75010 Paris, France; 2 Service Hématologie Adultes, Hôpital Saint-Louis, Assistance Publique-Hôpitaux de Paris, F-75010 Paris, France; 3 Univ. Lille, CNRS, Inserm, CHU Lille, IRCL, UMR9020 – UMR1277 - Canther – Cancer Heterogeneity, Plasticity and Resistance to Therapies, F-59000 Lille, France; 4 Laboratoire d’Hématologie, Hôpital Saint-Louis, Assistance Publique-Hôpitaux de Paris, F-75010 Paris, France; 5 Université Paris Cité, EA 3518, IRSL, Hôpital Saint-Louis, F-75010 Paris, France; 6 Service Hématologie Adolescents Jeunes Adultes, Hôpital Saint-Louis, Assistance Publique-Hôpitaux de Paris, F-75010 Paris, France; 7 Service d’Hématologie clinique, Hôpital Avicenne, Assistance Publique-Hôpitaux de Paris, Bobigny, France; 8 Service Hématologie Seniors, Hôpital Saint-Louis, Assistance Publique-Hôpitaux de Paris, F-75010 Paris, France

**Keywords:** Acute myeloid leukaemia, Translational research

## Abstract

Functional precision medicine in AML often relies on short-term in vitro drug sensitivity screening (DSS) of primary patient cells in standard culture conditions. We designed a niche-like DSS assay combining physiologic hypoxia (O_2_ 3%) and mesenchymal stromal cell (MSC) co-culture with multiparameter flow cytometry to enumerate lymphocytes and differentiating (CD11/CD14/CD15+) or leukemic stem cell (LSC)-enriched (GPR56+) cells within the leukemic bulk. After functional validation of GPR56 expression as a surrogate for LSC enrichment, the assay identified three patterns of response, including cytotoxicity on blasts sparing LSCs, induction of differentiation, and selective impairment of LSCs. We refined our niche-like culture by including plasma-like amino-acid and cytokine concentrations identified by targeted metabolomics and proteomics of primary AML bone marrow plasma samples. Systematic interrogation revealed distinct contributions of each niche-like component to leukemic outgrowth and drug response. Short-term niche-like culture preserved clonal architecture and transcriptional states of primary leukemic cells. In a cohort of 45 AML samples enriched for *NPM1c* AML, the niche-like multiparametric assay could predict morphologically (*p* = 0.02) and molecular (*NPM1c* MRD, *p* = 0.04) response to anthracycline-cytarabine induction chemotherapy. In this cohort, a 23-drug screen nominated ruxolitinib as a sensitizer to anthracycline-cytarabine. This finding was validated in an *NPM1c* PDX model.

## Introduction

The outcome of patients diagnosed with acute myeloid leukemia (AML) remains unsatisfactory and new therapeutic approaches are required [1]. Drug sensitivity patterns differ across AML genetic subsets, among which *NPM1*-mutated (NPM1c) AML is the most frequent [2].

After the failure of conventional therapies, there is no standard salvage regimen and patients are increasingly proposing personalized therapies based on their physical status and the genetic make-up of their disease. However, genetics may not suffice to choose the optimal therapy, because some targets may have multiple available inhibitors (e.g., *FLT3* mutations), and some active drugs may lack strong genetic biomarkers (e.g., venetoclax) [3]. Functional precision medicine based on ex vivo drug sensitivity screening (DSS) is gaining momentum to overcome these limitations [4–6].

DSS datasets can be leveraged at the population level, e.g., to identify drugs active in pre-defined AML subsets, or at the individual level, to tailor therapy to each patient. So far, most AML DSS platforms have tested drugs across broad concentration ranges in standard culture conditions, using the global viability of minimally fractionated primary samples to estimate drug sensitivity [4–6]. However, clinical drug exposure is bounded by bioavailability and dose-limiting hematological toxicity [1]. The leukemic niche provides resistance signals [7, 8]. Plasma-like medium improves the metabolic fidelity of in vitro assays [9, 10]. Notably, amino-acid levels differ between cancer patients and healthy subjects [11]. The tumor purity of primary AML samples is not absolute and leukemic cells can exist in different states including stem and differentiated states [12, 13]. Several surface markers have been proposed to enrich leukemic stem cells (LSCs) within the leukemic bulk [14]. Among those, expression of the G-coupled receptor GPR56 appears relatively universal across AML subsets [15, 16] and is stable upon short-term ex vivo culture [15].

Here we report the development and validation of an ex vivo drug screening platform for primary human AML cells that combines a niche-like culture system with a multiparametric flow cytometry readout. Focusing on *NPM1*-mutated AML, we perform clinical and genetic validation of the platform and nominate novel sensitizers to “7 + 3” daunorubicin (DNR) and cytarabine (AraC) combination chemotherapy in AML.

## Methods

Detailed Methods are provided in the *Supplementary Appendix* (online only).

### Primary AML samples

Fresh or viably frozen Bone Marrow (BM) or peripheral blood (PB) mononuclear cells (MNCs) from AML patients were collected after informed consent at the time of diagnosis or relapse by the INCa-labeled Hôpital Saint-Louis Tumor Bank. The project was approved by INSERM IRB (CEEI-15-220). Clinical and genetic annotations are provided in Supplementary Tables 1 and 2.

### Short-term ex vivo culture

All experiments were conducted in 96-well plate format. hTERT-MSC-GFP immortalized human mesenchymal stromal cells (MSCs) were provided by JP Bourquin) [17]. AML MNCs were seeded at 50,000 cells per well in 90 µL of MEMα standard medium, or in plasma-like culture medium (Supplementary Table 7), both supplemented with 25% dialyzed FBS, 100 IU/mL penicillin, and 100 µg/mL streptomycin. TPO 1 ng/mL and EPO 2.5 ng/mL (Peprotech, Neuilly-sur-Seine) were added to reach “plasma-like” cytokine concentrations. Drugs were resuspended in medium and immediately added to each well, with a maximum DMSO 0.1% final concentration. Plates were incubated for 72 hours at 37 °C in 20% or 3% O_2_ and 5% CO_2_ (hypoxia, MCO-19M-PE incubator, Panasonic, Genevilliers). Details are provided in the *Supplementary Appendix*.

### Multiparametric flow cytometry

Cells were washed and stained with Fixable Viability Stain eFluor 780 (Thermo Fisher Scientific), anti-CD45 PerCPCY5.5, anti-GPR56 PE, anti-CD11b APC, anti-CD14 APC, anti-CD15 APC, anti-CD3 BV421 and anti-CD19 BV421 (all BD Biosciences, Le Pont de Claix) and processed on an Attune Next (Thermo Fischer Scientific) flow cytometer. Cell counts were obtained after manual gating on FlowJo V10.6.2 (Beckton Dickinson, Le Pont de Claix). Details are provided in the *Supplementary Appendix*.

### AML drug screening data analyses

Cell counts in each gate were normalized to negative controls (DMSO 0.1% vehicle wells). Drug activity was determined as the actual (trapezoidal) area over the curve (AOC) of cell counts (ie without fitting a dose-response curve) without truncation, using the *PhamarcoGx* R package [18]. With AOCs, higher values indicate greater drug activity. Details are provided in the *Supplementary Appendix*.

### Statistical analyses

Statistical analyses were conducted in Prism 8.0.1 (GraphPad, San Diego, CA) or R version 3.6.0 (https://www.R-project.org/). Details are provided in the *Supplementary Appendix*.

### Data accessibility

Bulk and single-cell RNA-Seq will be available at European Genome-phenome Archive (EGA) under accession code EGAS00001006265. Other data will be available upon reasonable request to the principal investigator.

## Results

### Multiparametric flow cytometry readout for ex vivo culture of primary AML cells

**Fig. 1:**
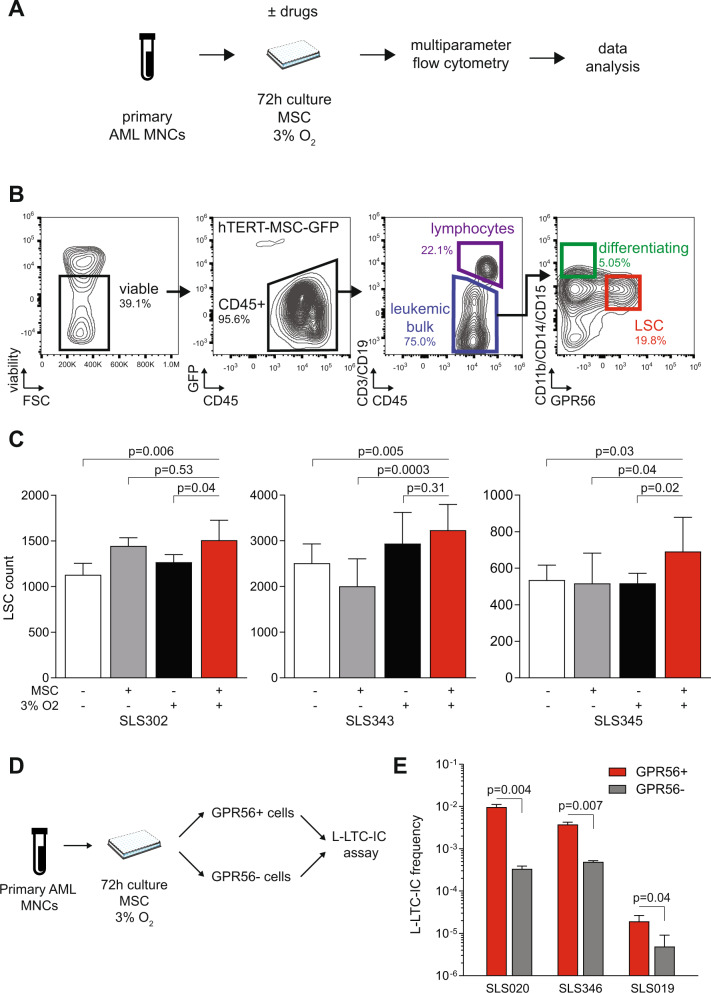
Multiparametric readout after ex vivo culture of primary AML cells. **A** Summary of the workflow for multiparametric flow cytometry following short-term ex vivo co-culture of primary AML MNCs with MSCs in 3% O_2_. **B** Gating strategy. **C** Number of GPR56+/Diff− LSCs after 72-hour culture with or without MSCs and with 20% (standard) or 3% (hypoxia) O_2_ in three samples; 6–10 technical replicates per condition. Mean ± SD. *T* tests with Welch’s correction. **D**, **E** Experiment design (**D**) and L-LTC-IC output (**E**) expressed as the ratio of colonies after 5-week culture per seeded GPR56+ or GPR56- cells sorted after a 72-hour culture with MSCs and 3% O_2_ in three different AML samples. Technical triplicates. Mean ± SD. Two-sided *T* tests with Welch’s correction. Clinical, phenotypic, and genetic annotations of primary AML samples are available in Supplementary Table 1.

We first sought to develop a core flow cytometry panel to enumerate residual cells following a 72-hour co-culture of human primary AML MNCs with human immortalized MSC-hTERT-GFP stromal cells in 3% O_2_ (Fig. 1a), a culture system that has been previously shown to maintain primary human LSCs and to recapitulate micro-environment-driven drug resistance [7, 8]. The gating strategy allows exact counting of viable cells within the leukemic bulk after exclusion of GFP + MSCs and CD3/CD19+ lymphocytes. Within the leukemic bulk, cells are assigned to a GPR56+ (henceforth LSC) state, to a CD11b/CD14/CD15+ (Diff+) differentiating state, or to the basal GPR56-Diff- blast state (Fig. 1b). We chose GPR56 to define the LSC population because its gene belongs to the core 17-gene stemness gene expression signature of AMLs and because its surface expression enriches for AML initiating potential across a broad spectrum of AML subsets (including CD34- *NPM1*-mutated AMLs) and is stable upon short-term ex vivo culture [15, 16, 19]. We validated that combination of hypoxia (3% O_2_) and MSC co-culture enhanced the number of viable GPR56+ leukemic cells after 72-hour culture ex vivo over each feature alone or standard (O_2_ 20%, no MSC) culture (Fig. 1c). In three primary AML samples, residual GPR56+ leukemic cells sorted after 72-hour hypoxic MSC co-culture were enriched ~10-fold in leukemic long-term initiating potential compared to GPR56- cells (Fig. 1d, e).

### Different patterns of drug activity at clinically relevant concentrations

**Fig. 2:**
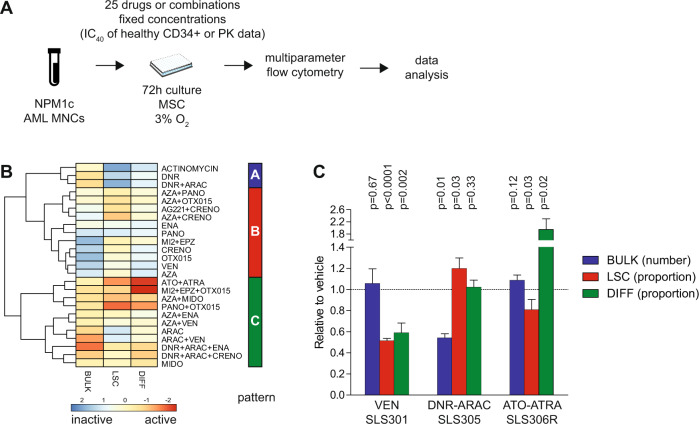
Different patterns of drug activity at clinically relevant concentrations. **A** Workflow of ex vivo drug screening of primary NPM1-mutated AML MNCs with 25 drugs or combinations at fixed, clinically relevant drug concentrations nominated based on published pharmacokinetics data or on dose–response assays in healthy CD34+ cells. **B** Heatmap of drug activity patterns for one illustrative case (SLS305, relapsed AML with trisomy 4 and *NPM1* mutation) displaying the number of residual cells in the leukemic bulk (BULK), the proportion of GPR56+/Diff− LSCs and that of GPR56−/Diff+ differentiating cells (DIFF). Results of the heatmap are displayed after scaling per output so that higher arbitrary values indicate a lower number of bulk leukemic cells, a lower proportion of LSCs, and a higher proportion of DIFF cells, hence higher drug activity. **C** Illustrative output of single-agent venetoclax (VEN), DNR-AraC, or ATO-ATRA in the three *NPM1*-mutant tested samples (SLS301 relapsed AML with isolated del(15q), *NPM1*, *DNMT3A*, *RAD21*, *CUX1*, *NRAS*, *KIT,* and *GATA2* mutations; SLS305 as *supra*; SLS306R relapsed AML with complex karyotype, *NPM1*, *FL3*-ITD, *DNMT3A,* and *SMC3* mutations, details on patient samples and gene mutations are provided in Supplementary Tables 1, 2). Results are shown normalized to DMSO vehicle wells. Mean ± SD of technical triplicates. Unpaired two-sided *t* tests.

We next interrogated the patterns of activity of 25 drugs (or combinations) at fixed concentrations in the niche-like culture system. Clinically relevant maximal concentrations for ex vivo drug testing were defined as either the peak plasma drug concentration in available pharmacokinetics (PK) studies, or as the concentration inhibiting 40% (IC_40_) growth of CD34+ hematopoietic stem/progenitor cells from healthy donors in niche-like (MSC, 3% O_2_) co-culture dose-response assays, whichever was lowest (Fig. 2a, Supplementary Tables 3 and 4). In one illustrative *NPM1*-mutated AML (SLS305), unsupervised clustering revealed different patterns of drug activity, including one with predominant activity on the leukemic bulk with lower efficacy on LSCs and differentiation (“cytotoxic” pattern A), one with predominant activity on the proportion of LSCs (“stemness-specific” pattern B) and one with prominent differentiating activity (pattern C, Fig. 2B). Expectedly, the “cytotoxic” cluster encompassed known chemotherapeutic agents including the standard AML combination therapy DNR and cytarabine (AraC) or actinomycin D, both of which have been shown to be clinically active in *NPM1*-mutated AML [20, 21]. The anti-stemness cluster was enriched in epigenetic drugs or combinations targeting BET bromodomains (OTX015), menin-MLL interactions (MI-2), and DOT1L (EPZ-5676), which have demonstrated pre-clinical activity on the NPM1c leukemia-initiating program [22–24]. Finally, the combination of arsenic trioxide (ATO) and all-trans retinoic acid (ATRA) was the most prominent member of the differentiating cluster, along with other doublets or triplets of epigenetic agents. Figure 2c displays the prototypical output of drugs or combinations from each cluster across different *NPM1*-mutated AMLs, highlighting the reduced proportion of LSCs (and DIFF cells) without impact on the total number of leukemic cells upon single-agent exposure to the BCL-2 inhibitor venetoclax, the relative sparing of LSCs with the cytotoxic DNR-AraC combination, and the differentiating activity of ATO-ATRA, also resulting in a seemingly unchanged count of total leukemic bulk. Altogether, these results are in keeping with known pre-clinical or clinical data regarding these agents in *NPM1*-mutated AML [25–28], and highlight the relevance of a multiparametric readout for ex vivo drug screening of primary AML samples.

### Refinement of niche-like culture with plasma-like medium

**Fig. 3:**
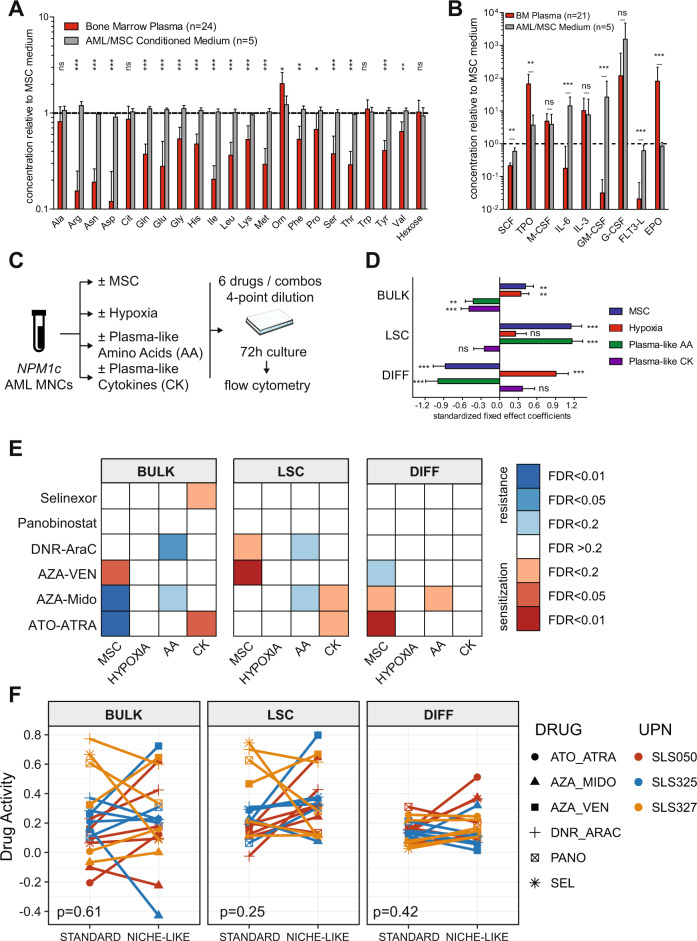
Plasma-like interacts with other pseudo-niche factors to dictate leukemic cell states and drug response ex vivo. **A**, **B** Amino-acid and hexose levels (**A** AbsoluteIDQ® p180 Assay, Biocrates) and cytokine levels (**B** custom U-Plex®, MesoScale Discoveries) from 21–24 diagnostic AML BM plasma samples and five AML/MSC co-cultures conditioned media relative to conditioned medium of MSC alone. Mean+SD. FDR *q* values from Mann–Whitney tests for the comparison of BM plasmas versus AML/MSC conditioned media. **C** Workflow of a mini-screen to systematically interrogate the contribution of the four pseudo-niche components (MSC, 3% O_2_, plasma-like amino acids, and cytokines) on leukemic states and drug sensitivity. **D** Fixed effects estimates (with SD) from mixed effect models investigating the contribution of each pseudo-niche component on each readout in the vehicle (DMSO 0.1%) treated cells from three *NPM1*-mutated AMLs, with random effect terms for patients. *P* values are from the mixed effect models. *p or *q* < 0.05, **p or *q* < 0.01, ***p or *q* < 0.001. **E** Heatmap of FDR *q* values for the impact of each pseudo-niche component on drug activity for each readout and drug, based on mixed-effect models with a random effect for patients. **F** Drug activities for each readout in standard versus niche-like conditions. *P* values from Wilcoxon signed-rank tests. Drug activity ranking in each patient is provided in Supplementary Fig. 3.

Conventional culture media poorly reflect the endogenous levels of metabolites such as amino acids and cytokines from normal human plasma [9, 10]. Furthermore, the metabolome and cytokinome of AML patients may differ from healthy subjects [29–32]. We thus undertook targeted metabolomics and multiplex cytokine dosages on the plasma of BM aspirates collected at AML diagnosis in 24 patients and compared them to conditioned media of primary AML samples co-cultured on an MSC layer in 3% O_2_, or to the conditioned medium of MSC alone. While amino-acid levels from conditioned media of AML co-cultures minimally differed from that of MSC alone co-culture, they significantly exceeded 1.4 to 3.6-fold those from diagnostic AML plasma for 16 of 21 tested amino acids (false discovery rate [FDR] < 0.01, Fig. 3A, Supplementary Table 5). Myeloid cytokine levels were highly variable in AML conditioned media but were systematically lower than BM plasma levels for TPO (mean 62.2 ± 61.1 versus 1128.2 ± 1012.9 pg/mL, *q* = 0.001) and EPO (mean 29.9 ± 7.9 versus 2852.0 ± 4506.0 pg/mL, *q* < 0.001, Fig. 3B, Supplementary Table 6).

We could thus design a custom medium based on amino-acid-free MEMa medium and dialyzed FBS supplemented with plasma-like AA levels and/or plasma-like EPO and TPO concentrations (Supplementary Table 7). We next systematically interrogated the contribution of each of the four components of our pseudo-niche culture system (addition of MSCs, lowering of O_2_ from 20% to 3%, substitution of the standard to plasma-like amino-acid levels, the addition of plasma-like levels of EPO and TPO cytokines) to the output of short-term ex vivo cultures of primary AML cells using multiparametric flow cytometry. Cells were treated with vehicle (DMSO) or with a mini-panel of six drugs/combinations with reported activity in *NPM1*-mutated AML in 4-point 10-fold dilution dose-response assays (Fig. 3c). Focusing first on vehicle-treated cells, each pseudo-niche component was found to have a distinct imprint on leukemic states ex vivo. Notably, both MSCs and hypoxia improved the viability of the leukemic bulk ex vivo while MSCs and plasma-like amino acids maintained leukemic cells in the LSC state at the expanse of differentiation (Fig. 3d). Importantly, up to 40% of the variance between the output of ex vivo culture in these different conditions could be explained by interactions between pseudo-niche factors (Supplementary Fig. 1). We next inspected the impact of each pseudo-niche component on drug activity. Overall, MSCs, plasma-like amino acids, and cytokines conveyed significant but variable sensitization or resistance to the 6 investigated drugs or combinations (Fig. 3e). Again, interactions between pseudo-niche components had a strong impact on drug responses, accounting for up to 70% of the variance (Supplementary Fig. 2). We finally compared drug activities in ‘standard’ culture conditions (no MSCs, 20% O_2_, standard medium without cytokine addition) to those in full “pseudo-niche” conditions (MSC layer, 3% O_2_, plasma-like amino acids, and cytokines). Overall, there was no systematic bias between culture conditions (Fig. 3f), and the ranking of drugs in each patient for each readout was not correlated (Supplementary Fig. 3), indicating that drug screening outputs performed in standard conditions cannot predict the results of those performed in niche-like conditions.

### Short-term niche-like ex vivo culture preserves intra-tumor heterogeneity

**Fig. 4:**
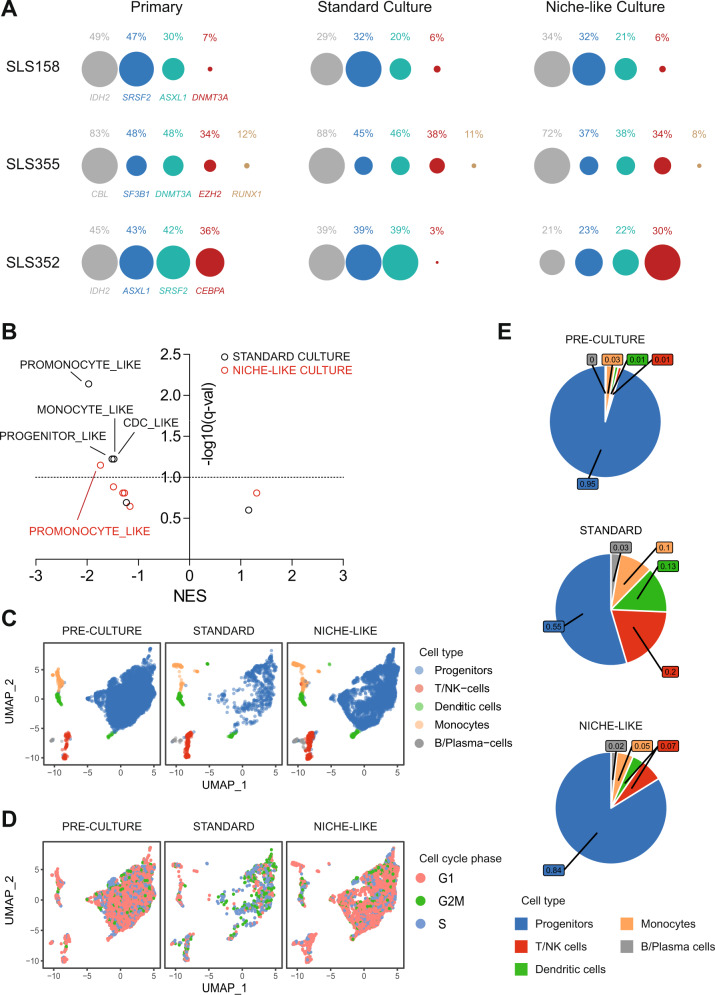
Clonal architecture and transcriptomes after short-term ex vivo culture. **A** Variant Allele Frequencies in primary AML MNCs and residual blasts after 72-hour culture in standard (no MSC, 20% O_2_, standard MEM-alpha medium) or niche-like (MSC co-culture, 3% O_2_, plasma-like amino acids, and cytokines) culture. Circles are proportional to VAFs, normalizing circle diameter based on the highest VAF in each sample. Additional cases are reported in Supplementary Fig. 4 and detailed mutations are provided in Supplementary Table 7**. B** Normalized Enrichment Scores (NES) and FDR-adjusted *q* values for the six leukemic state gene expression signatures from Van Galen et al. [13] (Supplementary Table 8) from RNA-Seq of four cryopreserved *NPM1*-mutated samples (SLS244, SLS354, SLS381, and SLS389) immediately after thawing (primary) or after 6 hours of ex vivo culture in niche-like or standard culture conditions. **C**, **D** UMAP plots of single-cell RNA-sequencing in pre-culture cells (*n* = 7008 cells) from SLS393 (normal cytogenetics, NPM1c and FLT3-ITD, additional details in Supplementary Tables 1, 2), after 72 hours of ex vivo culture in standard (*n* = 1079 cells) or niche-like (*n* = 4928 cells) conditions. **C**, **D** Projection of cell-type identity (**C**) or cell cycle phase identity (**D**). **E** Pie chart of the distribution of cell-type frequencies from **C**.

To determine whether short-term culture distorts the intra-tumor genetic heterogeneity of leukemic cells, we performed targeted sequencing of a 43 gene panel in archived BM MNCs obtained from 7 AML patients (median 4 gene mutations per patient, range 2–5, Supplementary Table 8) and performed amplicon-based sequencing of residual viable blasts after 72-hour culture in either standard or niche-like conditions. All 25 gene mutations were detectable in both culture conditions, including sub-clonal mutations with variant allele frequencies (VAF) < 20%, and limited distortion in VAF distribution compared to the primary specimen. In one sample (SLS352), niche-like culture better maintained *CEBPA*-mutant cells compared to standard culture (Fig. 4a and Supplementary Fig. 4). We similarly performed bulk RNA-Seq after a 6-hour culture and compared the transcriptome of cells exposed to niche-like versus standard culture conditions in 4 patients. When inspecting the gene expression signatures of leukemic cells reported at the single-cell level (Supplementary Table 9) [13], both culture conditions depleted the promonocyte-like signature but standard culture also significantly depleted three additional signatures that were preserved in niche-like conditions (FDR < 0.1, Fig. 4b). Finally, single-cell RNA-sequencing revealed that the distribution of BM mononucleated cell populations was preserved after a 72-hour culture in niche-like condition compared to the reference pre-culture specimen, while standard culture led to selective attrition of leukemic progenitors, with skewed cell cycle distribution (Fig. 4c). Of genes expressed in at least 10% of leukemic progenitor cells, 338 (103 up, 235 down) were differentially expressed ([fold change] >1.5 and FDR-adjusted *q* value <0.05) after standard culture versus pre-culture, compared to only 244 (up 57, down 187) after niche-like culture (Fisher exact test *p* = 0.001, Supplementary Tables 10–12, Supplementary Fig. 5). Collectively, these results suggest that short-term niche culture preserves the clonal heterogeneity and phenotypic diversity of primary AML specimens.

### Validation of the niche-like multiparametric drug screening platform

**Fig. 5:**
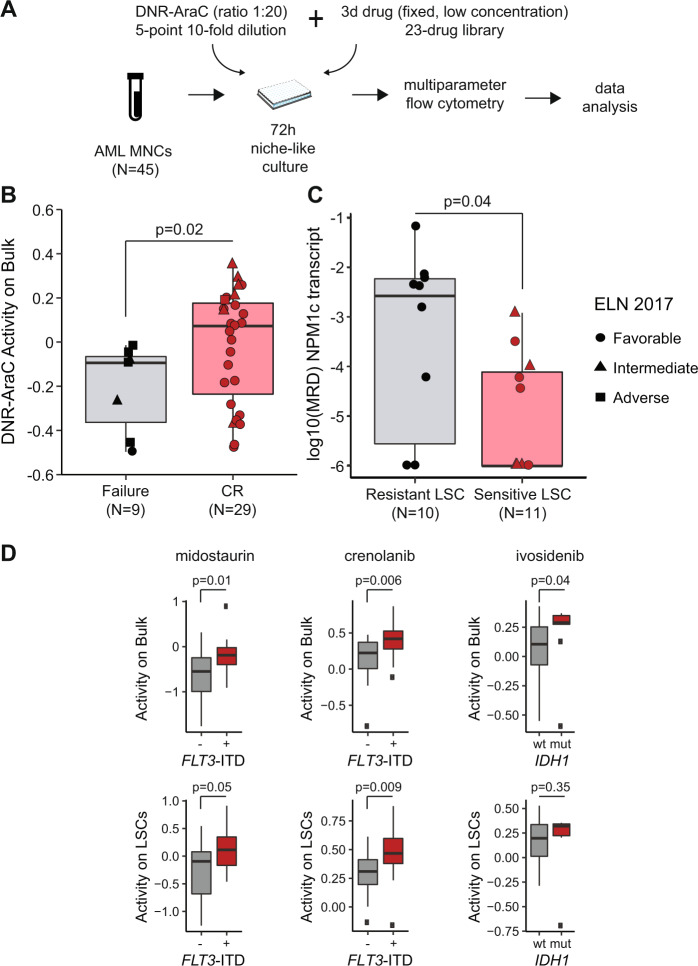
Validation of the niche-like multiparametric drug screening platform. **A** Ex vivo drug screening of primary MNCs from 45 AML patients (Supplementary Table 1, including 37 with *NPM1* mutations) with a 5-point, 10-fold DNR-AraC serial dilution in fixed concentration ratio (1:20) with or without fixed, low concentrations of each of 23 drugs (Supplementary Tables 3, 4). **B** DNR-AraC combination activity on the leukemic bulk in patients achieving CR (*N* = 29) or induction failure (*N* = 9) after an anthracycline-cytarabine induction course. Mann–Whitney test. Circles, triangles, and squares indicate individual data of samples with favorable, intermediate, and adverse genetic risk according to ELN 2017 classification [60]. Details on patient information and gene mutations are reported in Supplementary Tables 1, 2. **C** Log-transformed MRD on *NPM1* transcripts in 21 *NPM1*-mutated AML in CR, according to DNR-AraC ex vivo activity on LSC relative to lymphocytes (sensitive LSCs: activity on LSCs >activity on lymphocytes, *N* = 11; resistant LSCs: activity on LSCs ≤ activity on lymphocytes, *N* = 10). Mann–Whitney test. **E** Activity of the indicated drug on the bulk population (top panel) or the LSC population (bottom panel) according to the presence (*n* = 16) or absence of *FLT3*-ITD (*n* = 29) or the *IDH1* status (wildtype [wt] *n* = 36 or mutated [mut] *n* = 9). *P* values from Mann–Whitney tests.

We next investigated the clinical relevance of our niche-like, multiparametric drug screening platform. We performed a screen of a 5-point, 10-fold serial dilution of the conventional chemotherapy combination of daunorubicin and cytarabine (DNR-AraC) in 45 patient samples, including 37 NPM1c AMLs (Supplementary Table 1). The DNR-AraC combination was delivered at a fixed 1:20 ratio reflective of the average ratio observed in vivo upon administration of the combination [33], either alone, or with the addition of a fixed, low concentration of a 23-drug panel (Fig. 5a, Supplementary Figs. 6–8). Low dose concentrations were chosen as the IC_10_ for healthy CD34+ cells (see supra) or 20% of Cmax from PK data, whichever was lower (Supplementary Tables 3, 4). Drug activity on blasts was higher in patients achieving CR after induction chemotherapy, compared to those with induction failure (*p* = 0.02, Fig. 5B). When adjusting the drug activity on lymphocytes as an internal reference, all nine patients whose blasts were more sensitive to DNR-AraC compared to their lymphocytes (chemosensitive blasts) achieved CR, compared to 20 of the 29 (69%) patients with lower drug activity on blasts relative to lymphocytes (chemoresistant blasts; Fisher’s test *p* = 0.08). In 21 *NPM1*-mutated AML patients with available post-induction *NPM1c* transcript MRD data, MRD levels did not differ between patients with chemosensitive versus chemoresistant blasts (*p* = 0.35). Conversely patients with chemosensitive GPR56 + LSCs (*n* = 11) had lower post-induction MRD than those with chemoresistant LSCs (*n* = 10, *p* = 0.04, Fig. 5C). As a continuous variable, higher relative drug activity on LSCs (i.e., adjusted to activity on lymphocytes) was associated with longer Event-Free Survival (EFS, Cox model hazard ratio [HR] = 0.25, 95% confidence interval [CI] 0.08–0.78, *p* = 0.02), independently of adverse European LeukemiaNet risk (HR = 4.79, 95% CI 1.90–12.10, *p* = 0.001). The niche-like platform also validated known genetically targeted therapies (Fig. 5d). Specifically, addition of the pan-kinase inhibitor midostaurin to DNR-AraC lead to superior combination activity on blasts (*p* = 0.01), and to a lesser extent on LSCs (*p* = 0.05) of *FLT3*-ITD mutated samples (*n* = 16), while addition of the more potent *FLT3*-ITD inhibitor crenolanib had superior activity on both blasts (*p* = 0.006) and LSCs (*p* = 0.009). Finally, the IDH1 inhibitor ivosidenib led to enhanced killing of blasts (*p* = 0.04), but not LSCs (*p* = 0.35) in *IDH1*-mutated samples (*n* = 9). Of note, no difference was noted for the activity of the combination with the IDH2 inhibitor enasidenib between *IDH2* mutated (*n* = 7) and wildtype (*n* = 38) samples on bulk (*p* = 0.20) or LSC (*p* = 0.47) populations (*not shown*). Systematic inspection of chemogenomic relations further revealed that *FLT3* mutations significantly sensitized blasts and LSCs to the addition of venetoclax to DNR-AraC (*q* < 0.05, Supplementary Fig. 9).

### Discovery of novel drug combinations with niche-like multiparametric screening

**Fig. 6:**
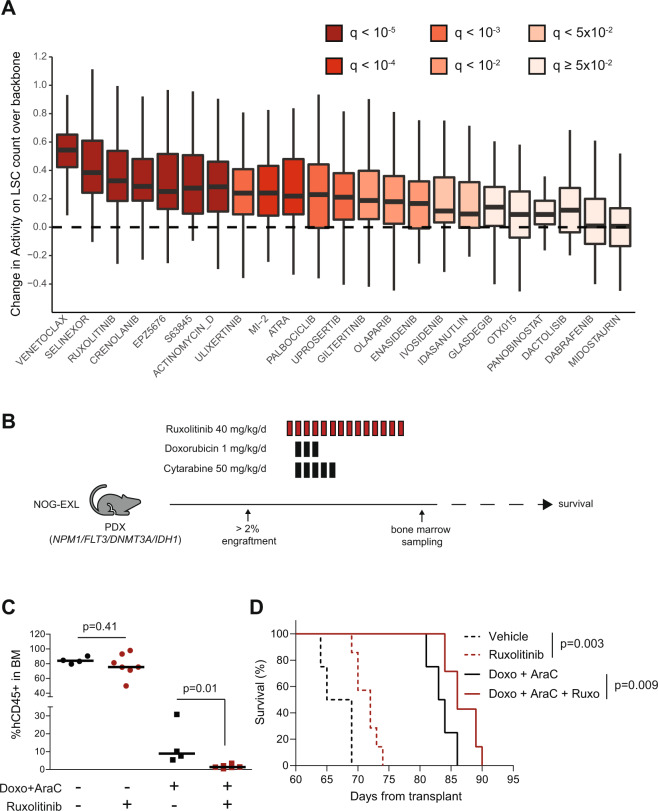
Niche-like multiparametric drug screening platform identifies chemosensitization by ruxolitinib. **A** Boxplot of the difference between drug activity on the count of GPR56+ /Diff− LSCs for each of the 23 triplets tested in the DNR-AraC screen (Fig. 5) and that of DNR-AraC backbone. FDR *q* values from *t* tests. **B** Experimental plan of the in vivo assessment of vehicle (*n* = 4), ruxolitinib (*n* = 8, 40 mg/kg/d orally, d1–14), chemotherapy (*n* = 4, doxorubicin 1 mg/kg/d IV d2-4 and cytarabine 50 mg/kg/d IP d2-6), or chemotherapy + ruxolitinib (*n* = 8) treatment of a PDX model (*NPM1c*, FLT3^*ITD*^, *DNMT3A*^R88H^, and IDH1^R132H^ mutations) after engraftment into sub-lethally irradiated NOG-EXL recipient mice. **C** Proportion of hCD45+ leukemic cells in bone marrow aspirates performed at day 16 (hence 2 days after the last ruxolitinib or vehicle administration). *P* values from Mann–Whitney tests. **D** Overall survival of the four mice groups since the first day of treatments. *P* values from log-rank tests.

Focusing on drug activity against GPR56 + LSCs, the results of this DNR-AraC screen could be interpreted at the individual level to nominate the optimal combination in each patient. Though the DNR-AraC-venetoclax triplet scored as the top combination for 27 (60%) of 45 tested patients, “private” optimal combinations with nine different third agents were nominated for 17 (38%) patients, while only 1 (2%) had no benefit of any of the 23 third agents tested (Supplementary Fig. 10), stressing the potential role for functional assays to tailor individual therapies in AML. Inspecting the screen at the population level to identify the average benefit of each third agent over the DNR-AraC backbone alone, akin to parallel clinical trials, confirmed the benefit of adding venetoclax or selinexor to DNR-AraC, both regimens being currently tested in clinical trials [34, 35], but also revealed a significant activity of the addition of the JAK inhibitor ruxolitinib (*q* < 10^−5^, Fig. 6a), which has so far been explored in patients with AML secondary to myeloproliferative neoplasms [36], but never in the setting of *NPM1* mutations. Of note, the activity of ruxolitinib in this setting was not dependent on *FLT3* status (Supplementary Fig. 9). To validate prospectively this finding, we treated xenotransplanted *NPM1*-mutated AML cells also harboring mutations in *DNMT3A*, *IDH1,* and *FLT3* with a combination of the anthracycline doxorubicin and cytarabine at maximal tolerated dose, ruxolitinib or the triplet combination (Fig. 6b). Post-treatment BM biopsies showed no reduction in leukemic burden with ruxolitinib alone compared to vehicle (*p* = 0.41), whereas the addition of ruxolitinib to doxorubicin-cytarabine further reduced leukemic infiltration over chemotherapy alone (*p* = 0.01, Fig. 6c). Ruxolitinib as a single-agent prolonged the survival of mice over vehicle (*p* = 0.003) and the addition of ruxolitinib to chemotherapy also significantly improved survival compared to chemotherapy alone (*p* = 0.009, Fig. 6d), providing in vivo confirmation of the ex vivo screen.

## Discussion

With the ongoing expansion of therapeutic options in AML, ex vivo DSS of primary AML cells has gained renewed interest to tailor personalized treatment decisions [4, 37–39], reposition existing therapies [40, 41], discover novel agents or combinations [42–44], or perform chemo-genetic correlations [6, 43]. Ex vivo DSS is often limited by the lack of niche mimicry and the limited information obtained by global viability assessment. Little is known of the impact of short-term ex vivo culture on the intra-tumor genetic and transcriptional heterogeneity of AML [13, 45, 46].

We report the development of a niche-like multiparametric platform for ex vivo drug screening of primary AML samples. Combining an immortalized MSC stromal layer with low oxygen concentrations recapitulating BM oxygen tension [7, 47], we could validate the choice of GPR56 as a surface marker to enrich residual LSC activity after short-term culture. Our simple 6-color flow panel was sufficient to capture distinct drug-induced phenotypes, including cytotoxicity sparing LSCs, inhibition of stemness potential, and differentiating activity, recapitulating known features of selected approved AML drugs or combinations [25–28].

A systematic investigation of our niche-like culture system on leukemia growth and drug response revealed the crucial role of interactions between MSCs, low oxygen, plasma-like amino acids, and cytokines on the number and phenotype of leukemic cells, and on their response to selected drugs or combinations. MSC and low oxygen-limited the attrition of primary AML cells upon ex vivo culture, with stroma and plasma-like amino acids maintaining the GPR56 + LSC-enriched phenotype, while hypoxia-induced phenotypic differentiation. Response to five of six tested drugs or combinations was significantly affected by the addition of stroma, plasma-like amino acids, or cytokines. Though oxygen level had no impact on sensitivity to any of the 6 drugs tested, we cannot exclude that it may modulate drug response on larger screens. Further work is thus needed to determine that hypoxia is a critical component of niche-like culture for primary AML cells. Importantly, in none of the three tested primary AML samples could results of the drug screen in niche-like culture be predicted by conducting the screen in standard conditions. In seven AML samples, we could show that short-term ex vivo culture does not distort clonal representation, minor sub-clones (variant allelic frequency <20% in the primary specimen) still being detectable in all tested patients, although the full resolution of clonal architecture would require whole-exome sequencing. Our bulk RNA-Seq results suggest a benefit of niche-like culture over standard culture in preserving leukemic transcriptional states, though a significant depletion of the promonocyte-like state was also noticeable upon niche-like culture, while single-cell RNA-sequencing revealed a skewed cell cycle distribution in residual cells after standard, but not niche-like culture.

Akin to several DSS studies [38, 48–51], we could show that ex vivo response of the leukemic bulk to a DNR-AraC combination was correlated to the achievement of remission after anthracycline-cytarabine “7 + 3” therapy. Focusing on *NPM1*-mutated AMLs, most of whom achieve remission after 7 + 3 chemotherapy [20], and using non-leukemic cells (lymphocytes) as an internal reference as previously proposed [38], we could show that the activity of DNR-AraC on GPR56+ LSC-enriched cells but not on the leukemic bulk, could predict the depth of remission as assessed by *NPM1*-transcript MRD, but also event-free survival. Of note, CD34 expression was not accounted for in this study, given its variable expression in *NPM1*-mutated LSCs [52]. This clinical validation was completed by a genetic one, whereby the presence of *FLT3* or *IDH1* mutations predicted the response to combinations including FLT3 or IDH1 inhibitors, respectively. Of note, no such correlation was noted with the IDH2 inhibitor enasidenib. Though this could simply reflect the limited size of the studied cohort (*n* = 45, including seven with an *IDH2* mutation), this could reflect a potential IDH2-independent activity of enasidenib [53].

The standard randomized clinical trial approach, even when selecting patients based on a genetic biomarker, has so far yielded limited benefit, owing to the variable benefit of adding a third agent to the standard 7 + 3 backbone. Indeed, by screening the addition of 23 drugs at concentrations deemed clinically relevant based on available PK data, or on drug testing of healthy CD34+ cells, we could show that in 39% of patients, the optimal triplet therapy is a private one. This result praises the development of robust DSS assays and innovative clinical trial design to foster functional precision medicine in AML [54]. Analyzing the average benefit of adding each of those 23 drugs to erode the GPR56 + LSC-enriched pool in a cohort of AMLs (many with *NPM1* mutation) confirmed the benefit of adding venetoclax or selinexor to intensive chemotherapy [34, 35]. More surprisingly, the addition of the JAK1/2 inhibitor ruxolitinib also markedly improved anti-LSC activity over DNR-AraC alone ex vivo. This benefit was not confined to the subset of patients harboring *FLT3* or signaling mutations and is reminiscent of the sensitization to BCL-2 inhibition by this kinase inhibitor recently reported by an ex vivo DSS study [55]. A combination of ruxolitinib with intensive chemotherapy in patients selected based on a DSS assay is currently explored by the BEAT-AML master trial (NCT03013998) [54], but to date, no clinical trial has reported the activity of this combination in de novo AML. The benefit of ruxolitinib addition could be validated in vivo in an *NPM1*-mutated PDX model, where engrafted mice received a ruxolitinib regimen in the lower range of reported in vivo experiments [56–58], and/or a combination of the anthracycline doxorubicin with cytarabine at maximally tolerated doses. Further work will be required to determine the mechanism, which could be non-cell-autonomous, through which ruxolitinib chemosensitizer *NPM1*-mutated AML cells [55].

Prospective analytical and clinical validation of our proposed niche-like ex vivo drug testing assay and benchmarking to existing platforms are important future steps [54, 59]. Further improvements to the assay will rely on miniaturization to improve throughput, incorporation of patient-derived MSCs, and culture medium refinement by accounting for plasma concentrations of polar metabolites [9]. More sophisticated flow cytometry panels will need to include additional LSC and differentiation markers and may also allow studying T cell activation. Collectively, our work contributes to the growing interest in functional assays to complement genomics-based precision oncology in leukemias.

## Supplementary information

Supplementary Methods and Figures (Clean)

Supplementary Tables 1–3 and 5-12

Supplementary Table 4
