# Supplementary material for: A multiparametric niche-like drug screening platform in acute myeloid leukemia
Source: Blood Cancer J. 2022 Jun 24;12(6):95. doi: 10.1038/s41408-022-00689-3 (PMC9232632; doi:10.1038/s41408-022-00689-3)

SUPPLEMENTARY INFORMATION FOR

A MULTIPARAMETRIC NICHE-LIKE DRUG SCREENING PLATFORM IN ACUTE MYELOID LEUKEMIA

AUTHORS: Reinaldo Dal Bello, Justine Pasanisi, Romane Joudinaud, Matthieu Duchmann, Gaetano Sodaro, Clémentine Chauvel, Loic Vasseur, Bryann Pardieu, Paolo Ayaka, Giuseppe Di Feo, Frank Ling, Kim Pacchiardi, Camille Vaganay, Jeannig Berrou, Chaima Benaksas, Thorsten Braun, Claude Preudhomme, Hervé Dombret, Emmanuel Raffoux, Nina Fenouille, Emmanuelle Clappier, Lionel Adès, Alexandre Puissant, Raphael Itzykson.

Table of Contents

Supplementary Methods 2
Supplementary References 15
Supplementary Tables 16
Supplementary Figures 17
    Supplementary Figure 1. 17
    Supplementary Figure 2. 18
    Supplementary Figure 3. 19
    Supplementary Figure 4.. 20
    Supplementary Figure 5. 21
    Supplementary Figure 6.. 23
    Supplementary Figure 7.. 24
    Supplementary Figure 8.. 25
    Supplementary Figure 9.. 26
    Supplementary Figure 10.. 27

Supplementary Methods

Clinical Annotations of AML samples

Induction chemotherapy (IC) consisted of a standard anthracycline-cytarabine ‘7+3’ regimen, with addition of a third drug (gemtuzumab ozogamycin or midostaurin) in a minority of cases. Complete Remission (CR) was defined according to ELN recommendations.[1] Patients with primary induction failure or morphologic leukemia-free state were grouped in the ‘failure’ group and those with early death (before end of induction bone marrow evaluation) were excluded from the analysis. Event-free survival was determined as the interval between diagnosis, primary induction failure, relapse or death, whichever occurred first and censored at allogeneic stem cell transplantation in first remission or last follow-up date.

Genetic Annotations of AML samples

NPM1, *FLT3* internal tandem duplications (*FLT3*-ITD) were tested using standard PCR-based methods as previously described.[2] *FLT3*-ITD allelic ratio (AR) quantified by fragment length analysis is expressed as ITD/wild-type allele ratio.[1] *IDH1* and *IDH2* were screened by Sanger sequencing.[3] Targeted sequencing was done with a custom SureSelectXT capture kit (Agilent Technologies) and 150-bp paired-end sequencing (Illumina MiSeq) and analyzed as previously described.[4] For chemogenomic screening, genes were grouped into cohesin (*RAD21*, *SMC1A*, *STAG2* or *SMC3*), *FLT3* (ITD or tyrosine kinase domain [TKD]), histone (*ASXL1*, *BCOR* or *EZH2*), methylation (*DNMT3A*, *IDH1*, *IDH2*, *TET2* or *WT1*), signaling (*BRAF*, *CBL*, *CSF3R*, *KIT*, *KRAS*, *NF1*, *NRAS*, *PTPN11* or *RIT1*) and splice (*PRPF8*, *SF3B1*, *SRSF2*, *U2AF1* or *ZRSR2*) groups. Measurable Residual Disease (MRD)

was performed on bone marrow samples by quantification of *NPM1*-mutated transcript levels with a mutation-specific real-time quantitative reverse transcriptase-polymerase chain reaction assay and *NPM1*-mutated transcript levels and expressed as a percentage of *ABL* copy number, as previously described.[2]

Drugs

All drugs were reconstituted and stored in DMSO except ATO that was stored in HCl 2%. Detailed references and stock concentrations are provided in **Supplementary Table 3**.

Short-term ex vivo culture

All experiments were conducted in 96-well plate format. The day before, hTERT-MSC-GFP immortalized human mesenchymal stromal cells (MSCs, provided by JP Bourquin)[5] were seeded at 10 000 cells per well in Minimal Essential Medium Alpha (MEMα, Thermo Fisher Scientific, Waltham, MA, cat #22571) medium supplemented with 10% Fetal Bovine Serum (FBS, Thermo Fisher Scientific, cat #1027-106), L-glutamine 2 mM, 100IU/mL penicillin and 100 µg/mL streptomycin (all from Eurobio, Les Ulis) and 1 µM hydrocortisone (Sigma-Aldrich, Saint-Louis, MI) and maintained at 37°C in 20% O_2 and 5% CO_2. The next day, the culture medium was removed and washed and AML MNCs were seeded at 50 000 cells per well using an electronic adjustable multichannel equalizer micropipette (E1-ClipTip Equalizer, Thermo Fisher Scientific) in 90 µL of MEMα standard medium, or in plasma-like culture medium (**Supplementary Table 7**), both supplemented with 25% dialyzed FBS (Sigma-Aldrich, cat - F0392), 100 IU/mL penicillin and 100 µg/mL streptomycin. When

indicated, culture medium was also supplemented with TPO 1 ng/mL and EPO 2.5 ng/mL (both from Peprotech, Neuilly-sur-Seine) to reach ‘plasma-like’ cytokine concentrations. Drugs were resuspended in standard medium, or in plasma-like culture medium supplemented as *supra*, and 10 µL of a 10X drug concentration immediately added to each well, resulting in a maximum DMSO 0.1% concentration. Plates were then incubated for 72 hours at 37°C in 20% O_2 and 5% CO_2 or in 3% O_2 and 5% CO_2 (hypoxia, MCO-19M-PE incubator, Panasonic, Genevilliers).

Multiparametric flow cytometry

Cells were washed and stained with Fixable Viability Stain eFluor 780 (Thermo Fisher Scientific), anti-CD45 PerCPCY5.5 (Clone HI30), anti-GPR56 PE (Clone CG4), anti-CD11b APC (Clone ICRF44), anti-CD14 APC (Clone M5E2), anti-CD15 APC (Clone HI98), anti-CD3 BV421 (Clone UCHT1) and anti-CD19 BV421 (Clone HIB19, all from BD Biosciences, Le Pont de Claix) and processed on an Attune Next (Thermo Fischer Scientific) flow cytometer. Cell counts were obtained after manual gating on FlowJo V10.6.2 (Beckton Dickinson, Le Pont de Claix).

Leukemic long-term culture initiating capacity (L-LTC-IC) assay

Leukemic long-term culture initiating capacity (L-LTC-IC) assay of GPR56+ and GPR56- residual leukemic cells was done as previously published.[6]

MS-5 stromal cells (kind gift of Eric Solary) were maintained in MEMα medium (Thermo Fisher Scientific, cat #22571) supplemented with 10% FBS (FBS, Gibco cat #1027-106), L-glutamine 2 mM (ThermoFischer Scientific, cat #25030-024), 100IU/mL penicillin and 100 µg/mL streptomycin (Eurobio, cat # CABPES010U) at

37°C in 20% O_2 and 5% CO_2. Forty-eight hours before co-culture, MS-5 cells were trypsinized, seeded in 6-well culture plates at 40,000 cells/well to reach a 70% confluence after 24 hours. The resulting feeder layer was irradiated at 5 Gy before addition of leukemic cells 24 hours later at $5x10^5$ cells /mL in 6-well plates. Following 72-hour co-culture with MS-5 in 3% O_2 in MEMα (Thermo Fisher Scientific, cat #22571) medium, 12.5% FBS qualified for long-term hematopoietic culture (Stem Cell Technologies, 06150), 12.5% horse serum (Stem Cell Technologies, cat #06750), 100IU/mL penicillin and 100 µg/mL streptomycin (Eurobio, cat # CABPES010U), residual cells were collected, stained with anti-CD45 PerCPCY5.5 (Clone HI30, BD Biosciences cat #564105), anti-GPR56 PE (Clone CG4, BD Biosciences cat #567213) and anti-mSca1 FITC (Clone D7, BD Biosciences cat # 557405).

Viable CD45+/mSca1- cells were sorted into GPR56+ and GPR56- fractions and plated at $5x10^5$ cells /mL into 6, 12 or 24 well plates according to the number of cells on a 5 Gy-irradiated MS5 stromal layer and cultured in in MEMα medium (Thermo Fisher Scientific, cat #22571) supplemented with 12.5% FBS qualified for long-term hematopoietic culture (Stem Cell Technologies, cat #06150), 12.5% horse serum (Stem Cell Technologies, cat #06750), 100IU/mL penicillin and 100 µg/mL streptomycin (Eurobio, cat # CABPES010U), 7.5 µg/mL alphathioglycerol (Sigma-Aldrich, cat # M6145) and cytokines (SCF 25 ng/mL, TPO 50 ng/mL, IL-3 10 ng/mL, FLT3-L 50 ng/mL, IL-6 10 ng/mL, G-CSF 10 ng/mL, GM-CSF 10 ng/mL, EPO 10 ng/mL, all from Peprotech) for three weeks with weekly half medium renewal.

At day 21, cells were mechanically resuspended, washed in Phosphate Buffered Saline (PBS, Life Technologies) with 2% Bovine Serum Albumin (BSA, Sigma-Aldrich, cat # A9418) and stained with APC-H7 anti-human CD45 (clone 2D1, BD

Biosciences cat # 560178) and FITC anti-mouse Sca1 (Clone D7, BD Biosciences cat # 557405). Residual leukemic mSca1-/hCD45+ cells were sorted on an Aria III cell sorter (Beckton Dickinson), washed and resuspended in 800 µL MEMα. This cell suspension was added to 3.2 mL of methylcellulose (50% H4100 Methocult® [Stem Cell Technologies], 37.5% FBS [Stem Cell Technologies], 1.25% bovine serum albumin (BSA), 1% L-glutamine, and 7.5 µg/mL alphathioglycerol) supplemented with cytokines (SCF 25 ng/mL, TPO 50 ng/mL, IL-3 10 ng/mL, FLT3-L 50 ng/mL, IL-6 10 ng/mL, G-CSF 10 ng/mL, GM-CSF 10 ng/mL, EPO 10 ng/mL [all from Peprotech]) before seeding in triplicate at 1 mL per 60x19 mm culture dish. Colonies were counted after 14 days incubation in 20% O_2, 5% CO_2 at 37°C. The colony count was multiplied by 3 (triplicate methylcellulose dishes) and normalized to the number of sorted GPR56+ and GPR56- cells to derive the Long-Term Culture Initiating Cell (LTC-IC) frequency in each fraction.

Healthy CD34+ dose-response assays

Leukapheresis or cord blood samples from healthy donors were obtained after approval from the Saint-Louis Hospital Cell Therapy Department. Following gradient density centrifugation, CD34+ stem/progenitor cells were obtained by immunomagnetic enrichment (Macs; Miltenyi Biotec). 10,000 CD34+ cells per well were seeded in 96-well plates previously coated with hTERT-MSC-GFP cells and tested in triplicate in a dose-response assay in a 5-point, 3-fold dilution assay in a 72-hour culture in standard culture conditions (MEMα [Thermo Fisher Scientific, cat #22571] supplemented with 12.5% FBS qualified for long-term hematopoietic culture [Stem Cell Technologies, cat #06150], 12.5% horse serum [Stem Cell Technologies,

cat #06750], 100IU/mL penicillin and 100 µg/mL streptomycin (Eurobio, cat # CABPES010U) and 7.5 µg/mL alphathioglycerol (Sigma-Aldrich, cat # M6145). Half-maximal inhibitory concentrations (IC50s) were obtained by fitting a four-parameter dose-response curve with the least square method in Prism 8.0.1 (GraphPad). Inhibitory concentrations for alternative cell fractions (eg. IC40) were obtained based on IC50s and Hill's coefficients with the Quickcals online calculator (https://www.graphpad.com/quickcalcs/Ecanything1.cfm, GraphPad).

Systematic interrogation of pseudo-niche components in a mini-panel drug screening

To study the role of pseudo-niche components on basal culture outputs (ie without drugs), cell counts of total leukemic bulk, LSCs and differentiating cells were filtered on DMSO 0.1% vehicle wells (6 technical replicates per condition). Mixed effects models were carried for each output (total number of cells in the leukemic bulk, proportion of LSCs and proportion of differentiating cells in the bulk) after scaling for standardization of fixed effect coefficients, including terms for MSC (reference: no MSC), hypoxia (reference: 20% O_2), plasma-like amino acids (reference: standard culture medium) and plasma-like cytokines (reference: no cytokine addition), second order interaction terms between pseudo-niche components terms, and including a random effect for AML patients. The relative importance of each term in the model was determined based on the R^2 partitioned by averaging over orders (lmg metrics) using the *relaimpo* R package.[7]

To study the role of pseudo-niche components on drug activities, similar mixed effect models were carried including AUCs on the total number of leukemic cells, number of LSCs and number of differentiated cells, with fixed effects for pseudo-niche

components and random effects for patients. Differences across drugs were studies by performing subgroup analysis focusing on each individual drugs, while overall behaviors were investigated by adding a random effect term for drugs in the mixed effect models.

AML bone marrow plasmas and conditioned media

Plasma samples were collected at the time of diagnosis from BM aspirates in EDTA. Briefly, BM samples were centrifuged at 260g for 5 minutes. The platelet-rich plasma fraction was collected and further centrifuged at 1850 g for 15 minutes. Aliquots of the platelet-low plasma fraction were stored at -80°C.

Conditioned medium from h-TERT-MSC-GFP cells (n=1) or h-TERT-MSC-GFP and primary AML MNCs co-cultures (n=5) grown in MEMα (Thermo Fisher Scientific, cat #22571) supplemented with 12.5% FBS (Stem Cell Technologies, cat #06150), 12.5% horse serum (Stem Cell Technologies, cat #06750), 7.5 µg/mL alphathioglycerol (Sigma-Aldrich, cat # M6145), 100IU/mL penicillin and 100 µg/mL streptomycin (EUROBIO, cat # CABPES010U) for 48 hours. Supernatants were collected, centrifuged and stored as *supra*.

Targeted metabolomics

Aliquots of diagnostic AML BM plasma samples (n=24) or conditioned media from h-TERT-MSC-GFP cells (n=1) or h-TERT-MSC-GFP and primary AML MNCs co-cultures (n=5) underwent targeted metabolomics with the AbsoluteIDQ® p180 Assay (Biocrates, Innsbruck, Austria).

Multiplex cytokine levels assays

Aliquots of diagnostic AML BM plasma samples (n=21) and conditioned media from h-TERT-MSC-GFP cells (n=1) or h-TERT-MSC-GFP and primary AML MNCs co-cultures (n=5) underwent targeted cytokine profiling through electrochemoluminescence with a custom multiplex assay for EPO, FLT3L, G-CSF, GM-CSF, IL-3, IL-6, M-CSF, TPO (U-PLEX®) and a standard R-PLEX® SCF assay and processed on a Quickplex station (all from MesoScale Discovery, Rockville, Maryland) according to manufacturer's guidelines.

RNA-Seq

Cryopreserved AML MNCs from 4 patients were thawed and immediately processed for RNA extraction (primary AML) or cultured in 6-well plates at 500.000 cells/mL during 6 hours in niche-like or standard conditions, washed in PBS 2.5% EDTA (Invitrogen, cat # 15575-038) then processed for RNA extraction using RNeasy plus microkit (Qiagen cat # 74034). RNAs were assessed using a BioAnalyzer 2100 (Agilent). Only 9 of 12 samples passed QC. Libraries were generated from total RNA using the SMARTer Universal Low Input RNA Kit (Takara Bio) according to manufacturer's instructions and sequenced on Illumina NovaSeq6000 to (100 bp paired-end reads). RNA seq reads were aligned to the human reference genome using Salmon 1.0.0.[8] DESeq2 was used to obtain normalized expression values.[9] GSEA analysis was run with *fgsea* on pre-ranked genes using log-transformed fold change as ranking metric, using the 6 leukemic signatures from Van Galen et al. (**Supplementary Table 9**).[10]

DNA-Seq

Identification of gene mutations with a 43-gene panel on primary AML MNCs at diagnosis was done as previously described.[4] Viably frozen MNCs were thawed and cultured in 6-well plates at 500.000 cells/mL during 72 hours in niche-like or standard conditions as described supra. After 72 hours, cells were resuspended in PBS 2.5% EDTA (Invitrogen, cat # 15575-038), labeled with PE anti-CD45 (Beckman, cat # A07783) and 7-Aminoactinomycin D (7-AAD, Thermo Fisher Scientific cat # A1310) and viable CD45dim/SSClow blasts (median 3.75x105 cells, range 0.85 - 19.8 x10^5 cells) were sorted in BD FACSAria II (BD Biosciences). DNA extraction was done using DNeasy blood and tissue kit (Qiagen, cat # 69504) according to manufacturer's instructions. DNA concentrations were determined using a Qubit dsDNA BR Assay (Thermofisher Q32850) according to manufacturer's instructions. Primers (**Supplementary Table 8**) were designed and tested on bulk for all somatic mutations detected on bulk sequencing at diagnosis for the 7 patients. Targeted amplicons were generated from diagnosis and post-culture samples using a multiplexed PCR using KAPA2G Robust HotStart kit (Sigma Aldrich, cat KK5515) (number of cycles = 26 or 32), were purified using AMPure XP beads (Beckman, A63881) and quantified using Bioanalyzer High Sensitivity DNA kit (Agilent, 5067-4626). Library preparation was done using the Illumina DNA prep protocol (Illumina, cat 20018704) and Nextera DNA CD Indexes (Illumina, 20018707) using an initial input of 10 ng of purified PCR products. The final library was sequenced on a Miseq (Illumina, V2 chemistry, 150 bp x2, dual indexes). Fastq files were pre-processed using Fastp (version 0.20.1),[11] aligned with BWA (version 0.7.17),[12] on the Homo Sapiens reference Genome version hg38. Variant calling was done using Varscan (version 2.4.4) and Pindel (version 0.2.5b9) callers.

Single-cell RNA sequencing

After thawing, cryopreserved AML MNCs from patient SLS393 from the same vial were split into three aliquots of 2x10^6 cells each. The first was submitted to Dead Cell removal protocol (Dead Cell Removal Kit, Miltenyi Biotec, cat #130-090-101), followed by positive hCD45 immunomagnetic selection (CD45 MicroBeads human, Miltenyi Biotec, cat #130-045-801) following the manufacturer's instructions and submitted to scRNA-seq (pre-culture sample). The two others were cultured in 6-well plates at 500.000 cells/mL in niche-like or standard conditions for 72 hours, harvested and submitted to Dead Cell Removal and positive hCD45 immunomagnetic selection as *supra* before scRNA-seq.

For scRNA-seq library preparation, cells were processed using the Chromium platform and the 3' Gene expression v3.1 library preparation kit (10XGenomics). Libraries were pooled and sequenced on a Novaseq S4 platform (Illumina). Fastq files were processed using Cellranger V6, and counts data were analyzed using Seurat V4.[13] Counts were normalized the *SCTransform* method with regression on cell-cycle scores, then integrated using reciprocal principal component analysis. Differential expression testing was done on 500 progenitor cells for each condition using the MAST package.[14] For each contrast, only genes expressed in at least 10% of cells in at least one of the two groups were considered. GSEA analyses were run with *fgsea* on pre-ranked genes using log-transformed fold change as ranking metric, using hallmark pathways from MSigDB (https://www.gsea-msigdb.org/gsea/msigdb/).[15] Cell identity was based on expression of canonical markers (i.e *IL7R* and CD8A for T cells, *CD79A* for B cells, *NKG7* for NK cells, *CD14* for monocytes, *CST3* for dendritic cells, *IGFBP5* for stromal cells). Progenitor cells highly expressed *HOXA9*, *KIT*, *IL3RA* and *MYC*, and were enriched for HSPC-like

and Progenitor-like signatures derived from Van Galen et al.[10] Cell cycle scores were derived from Tirosh et al.[16]

Patient Derived Xenotransplants (PDX)

All mouse experiments were performed in accordance with protocols approved by the Comité d'Ethique en Expérimentation Animale Paris-Nord n°121 (project #8909-2017021413452743). 2.5 x10^6 *FLT3*-ITD, *NPM1*c, *DNMT3A*^{R882H}, and *IDH1*^{R132H} AML primary cells were transplanted by tail vein injection into 10-12 week-old sub-lethally irradiated recipient NOD.Cg-Prkdcscid IL2rgtm1Sug Tg(SV40/HTLV-IL3, CSF2) 10-7Jic/Jic Tac (hu NOG-EXL) males purchased from Taconic. Sample size was chosen according to historical experiments with this highly penetrant and consistent model. Blinded observers visually inspected mice for signs of distress (appetite loss, hunched posture or lethargy). Animals were to be excluded in case of distress. None of our mice had to be excluded because of distress without detectable blasts in the blood or bone marrow. Fourteen days after injection, engraftment was confirmed by measuring circulating hCD45-positive blasts in blood. Following randomization, mice were treated once daily by oral gavage with 40mg/kg ruxolitinib (Medchemexpress cat # HY-50858) diluted in 0.5% methylcellulose (Alfa Aesar, cat # 43147) or vehicle only for 14 days, once daily with chemotherapy (tail vein injection of 1mg/kg doxorubicin for 3 days and intraperitoneal injection of 50mg/kg cytarabine for 5 days), starting on day 2 of ruxolitinib or vehicle, or both chemotherapy and ruxolitinib.

Bone marrow biopsies were performed on anesthetized animals at indicated time points. Sample were lysed in Red blood cell lysing buffer (Sigma, cat # R7757), washed twice with PBS and resuspended in PBS 0.5% BSA (Sigma-Aldrich, cat # A7906), 2mM EDTA (Invitrogen, cat # 15575-038) prior to staining with APC-

conjugated anti-human CD45 (clone 2D1, BioLegend, cat # 368512). Samples were washed 3 times before analysis on a BD FACSCanto II (BD Biosciences) flow cytometer.

AML drug screening data analyses

Cell counts in each gate were normalized to negative controls (DMSO 0.1% vehicle wells). Drug activity was determined as the actual (trapezoidal) area over the curve (AOC) of cell counts (ie without fitting a dose-response curve) without truncation, using the *PhamarcoGx* R package.[17] With AOCs, higher values indicate greater drug activity. The 'Area Over the Curve'-type metric, integrating potency and efficacy was chosen for its robustness compared to conventional potency metrics such as IC50s.[18] Actual rather than fitted data was used because of the limited relevance of conventional dose response curve fitting for non-cytotoxic drugs.[19,20] Finally, cell counts were not truncated to account for the fact that some drugs may induce differentiation not captured by our differentiation markers (CD11b, CD14 and CD15), resulting in cell counts over the baseline of vehicle-treated wells in the blasts gate. Thus, drug activity metrics can fall below 0. For the differentiation activity metric, normalized differentiating cell counts (Diff) were transformed in each experiment as ~ $1 – (1/Diff_{max})$ to obtain activity metrics in the 0-1 range, with higher values indicating more potent differentiation.

Statistical analyses

Statistical analyses were conducted in Prism 8.0.1 (GraphPad) or R version 3.6.0 (https://www.R-project.org/). Group comparisons for continuous variables were

carried with student t tests with Welch's correction or Mann-Whitney's tests for technical and biological replicates, respectively. Survival analyses were done with Cox models. Adjustment for multiple testing was done with the Benjamini & Hochberg method.[21]

Supplementary References

1. Dohner H, Estey E, Grimwade D, et al. Diagnosis and management of AML in adults: 2017 ELN recommendations from an international expert panel. *Blood*. 2017;129(4):424-447.
2. Balsat M, Renneville A, Thomas X, et al. Postinduction Minimal Residual Disease Predicts Outcome and Benefit From Allogeneic Stem Cell Transplantation in Acute Myeloid Leukemia With NPM1 Mutation: A Study by the Acute Leukemia French Association Group. *J Clin Oncol*. 2017;35(2):185-193.
3. Boissel N, Nibourel O, Renneville A, et al. Prognostic impact of isocitrate dehydrogenase enzyme isoforms 1 and 2 mutations in acute myeloid leukemia: a study by the Acute Leukemia French Association group. *J Clin Oncol*. 2010;28(23):3717-3723.
4. Cerrano M, Duchmann M, Kim R, et al. Clonal dominance is an adverse prognostic factor in acute myeloid leukemia treated with intensive chemotherapy. *Leukemia*. 2020.
5. Frismantas V, Dobay MP, Rinaldi A, et al. Ex vivo drug response profiling detects recurrent sensitivity patterns in drug-resistant acute lymphoblastic leukemia. *Blood*. 2017;129(11):e26-e37.
6. Masse A, Roulin L, Pasanisi J, et al. BET inhibitors impair leukemic stem cell function only in defined oncogenic subgroups of acute myeloid leukaemias. *Leuk Res*. 2019;87:106269.
7. Groemping U. Relative Importance for Linear Regression in R: The Package relaimpo. *2006*. 2006;17(1):27.
8. Patro R, Duggal G, Love MI, Irizarry RA, Kingsford C. Salmon provides fast and bias-aware quantification of transcript expression. *Nat Methods*. 2017;14(4):417-419.
9. Love MI, Huber W, Anders S. Moderated estimation of fold change and dispersion for RNA-seq data with DESeq2. *Genome Biol*. 2014;15(12):550.
10. van Galen P, Hovestadt V, Wadsworth Ii MH, et al. Single-Cell RNA-Seq Reveals AML Hierarchies Relevant to Disease Progression and Immunity. *Cell*. 2019;176(6):1265-1281 e1224.
11. Chen S, Zhou Y, Chen Y, Gu J. fastp: an ultra-fast all-in-one FASTQ preprocessor. *Bioinformatics*. 2018;34(17):i884-i890.
12. Li H, Durbin R. Fast and accurate short read alignment with Burrows-Wheeler transform. *Bioinformatics*. 2009;25(14):1754-1760.
13. Hao Y, Hao S, Andersen-Nissen E, et al. Integrated analysis of multimodal single-cell data. *Cell*. 2021;184(13):3573-3587 e3529.
14. Finak G, McDavid A, Yajima M, et al. MAST: a flexible statistical framework for assessing transcriptional changes and characterizing heterogeneity in single-cell RNA sequencing data. *Genome Biol*. 2015;16:278.
15. Liberzon A, Birger C, Thorvaldsdottir H, Ghandi M, Mesirov JP, Tamayo P. The Molecular Signatures Database (MSigDB) hallmark gene set collection. *Cell Syst*. 2015;1(6):417-425.
16. Tirosh I, Venteicher AS, Hebert C, et al. Single-cell RNA-seq supports a developmental hierarchy in human oligodendroglioma. *Nature*. 2016;539(7628):309-313.
17. Smirnov P, Safikhani Z, El-Hachem N, et al. PharmacoGx: an R package for analysis of large pharmacogenomic datasets. *Bioinformatics*. 2016;32(8):1244-1246.
18. Fallahi-Sichani M, Honarnejad S, Heiser LM, Gray JW, Sorger PK. Metrics other than potency reveal systematic variation in responses to cancer drugs. *Nat Chem Biol*. 2013;9(11):708-714.
19. Ritz C, Baty F, Streibig JC, Gerhard D. Dose-Response Analysis Using R. *PLoS One*. 2015;10(12):e0146021.
20. Tyner JW, Tognon CE, Bottomly D, et al. Functional genomic landscape of acute myeloid leukaemia. *Nature*. 2018;562(7728):526-531.
21. Benjamini Y, Hochberg Y. Controlling the False Discovery Rate - a Practical and Powerful Approach to Multiple Testing. *J Roy Stat Soc B Met*. 1995;57:289-300.

Supplementary Tables

Available online.

Supplementary Figures

Supplementary Figure 1. Relative importance (lmg metrics) of each pseudo-niche component and their interactions on each readout in vehicle (DMSO 0.1%) treated cells based on the same mixed effect models as depicted in Figure 3C.

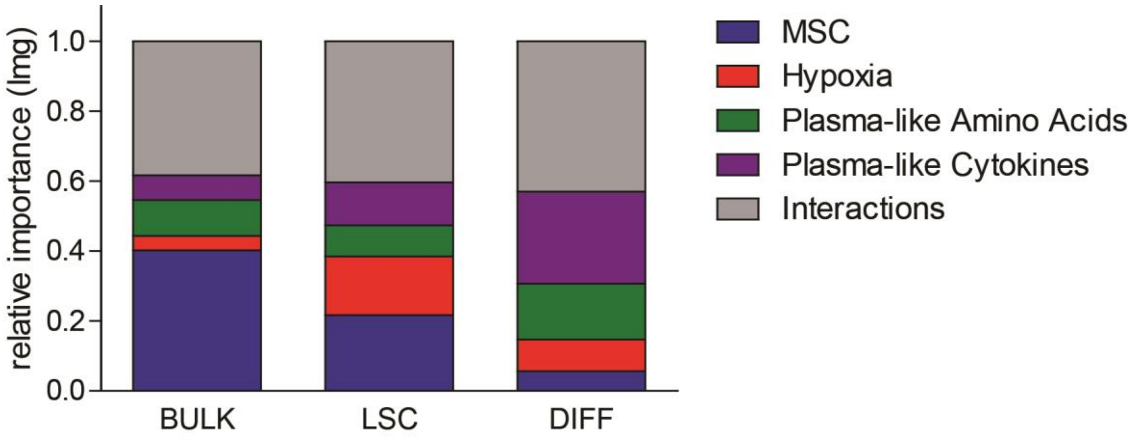

Supplementary Figure 2. Relative importance (lmg metrics) of each pseudo-niche component and their interactions on each readout of drug activity in mixed effect models including random effects for drugs and patients.

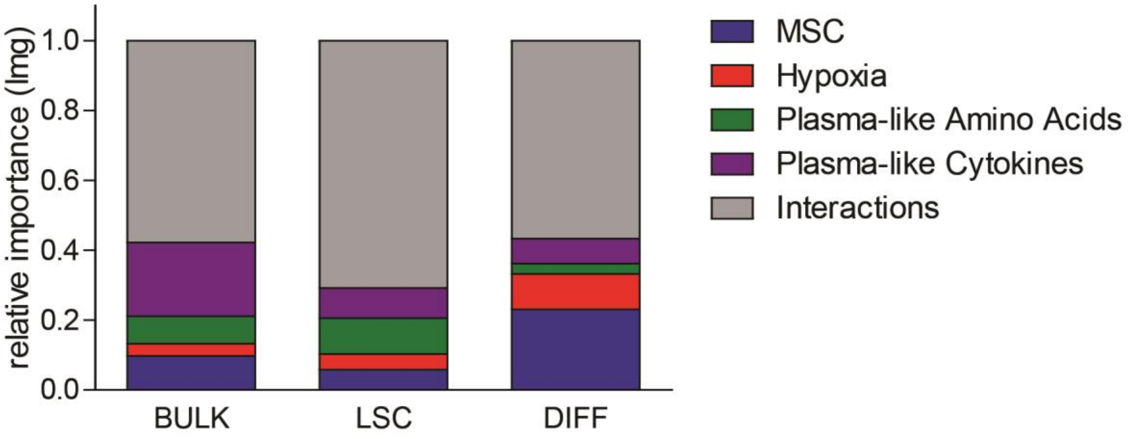

Supplementary Figure 3. Drug activities for each readout and each patient in standard versus niche-like conditions. P values from Wilcoxon signed rank tests.

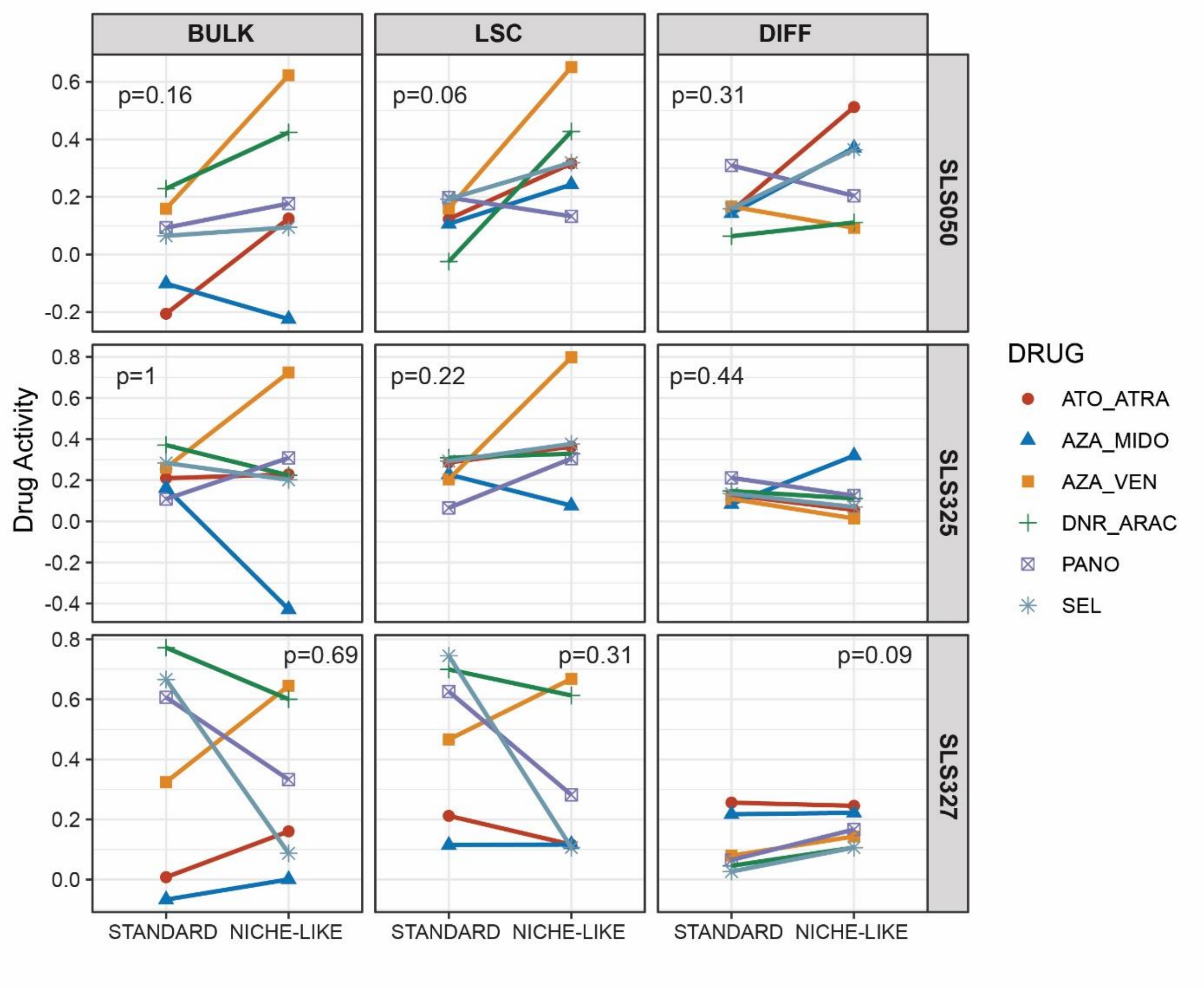

Supplementary Figure 4. Variant Allele Frequencies in primary AML MNCs and residual blasts after 72-hour culture in standard (no MSC, 20% O2, standard MEM-alpha medium) or niche-like (MSC co-culture, 3% O2, plasma-like amino acids and cytokines) culture. Circles are proportional to VAFs, normalizing circle diameter based on the highest VAF in each sample. Additional cases from **Figure 4A**. Detailed mutations are provided in **Supplementary Table 7**.

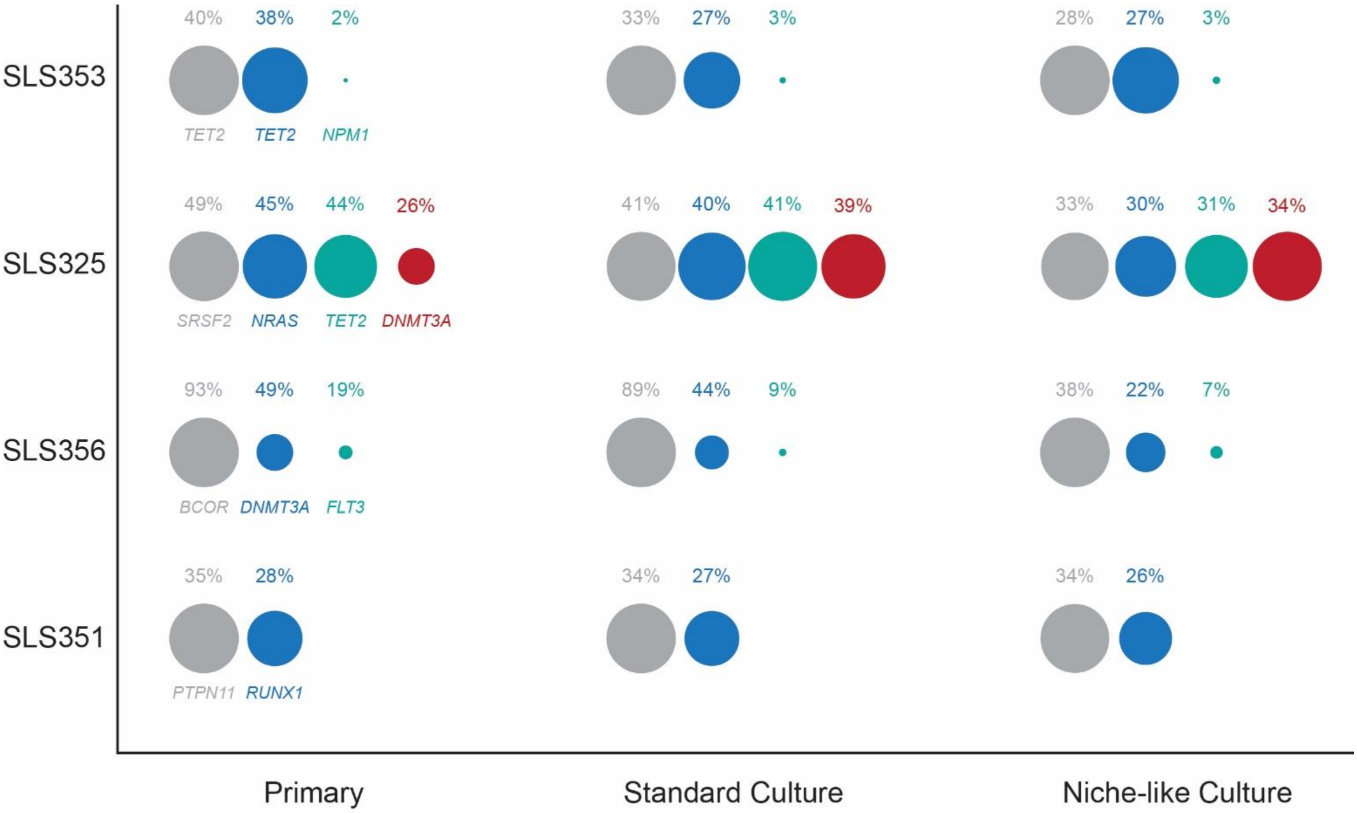

Supplementary Figure 5. A. Venn Diagramm of Differentially Expressed Genes (absolute fold change > 1.5 and FDR q-value <0.05) and **B.** Heatmap of Normalized Enrichment Scores (NES, if FDR q-value < 0.1) after Gene Set Enrichment Analysis on MSigDB pathways on average expression levels in 500 progenitor cells from SLS393 determined by sc-RNA-Seq pre-culture and after 72 hours of *ex vivo* culture in standard or niche-like conditions.

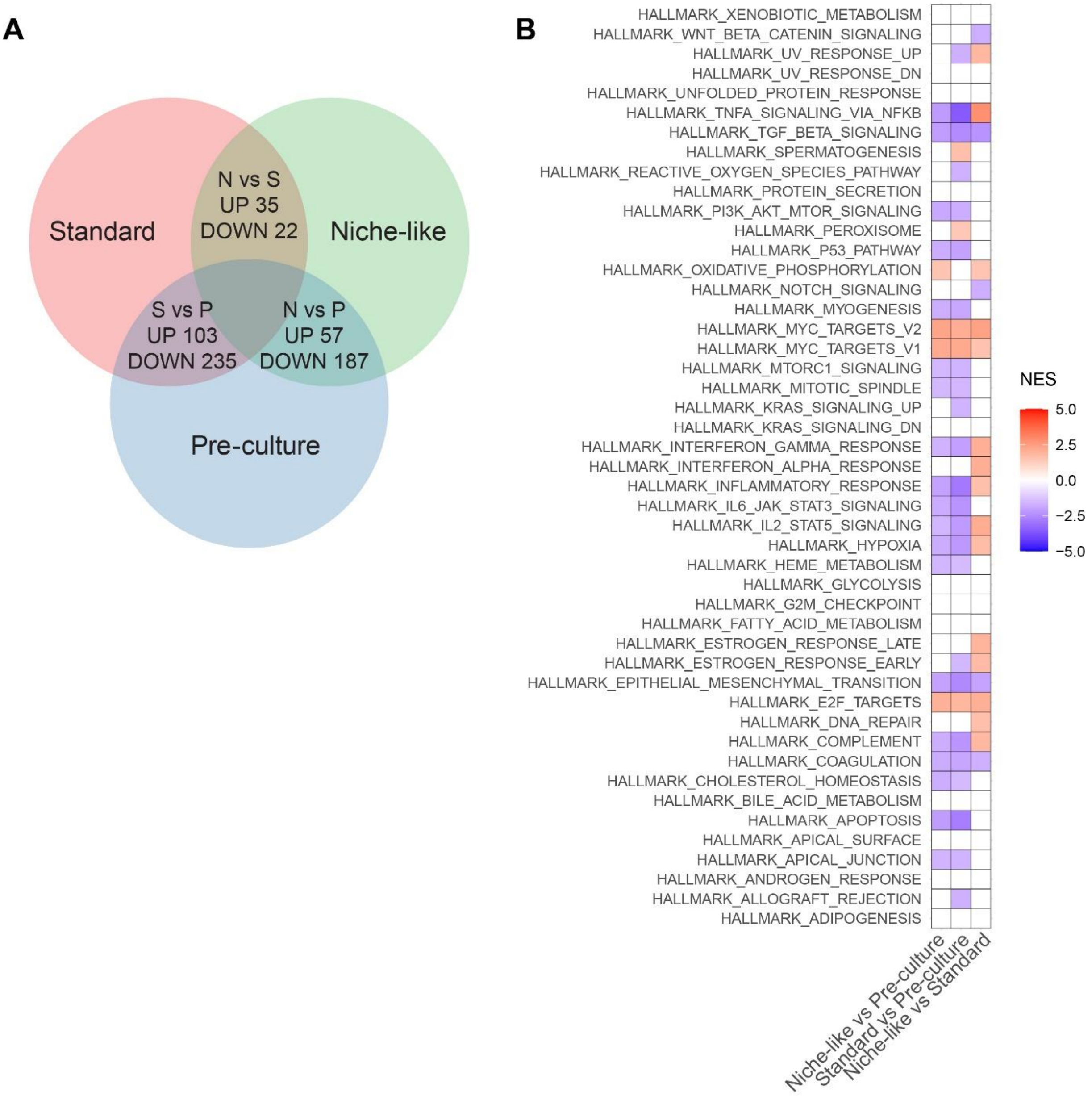

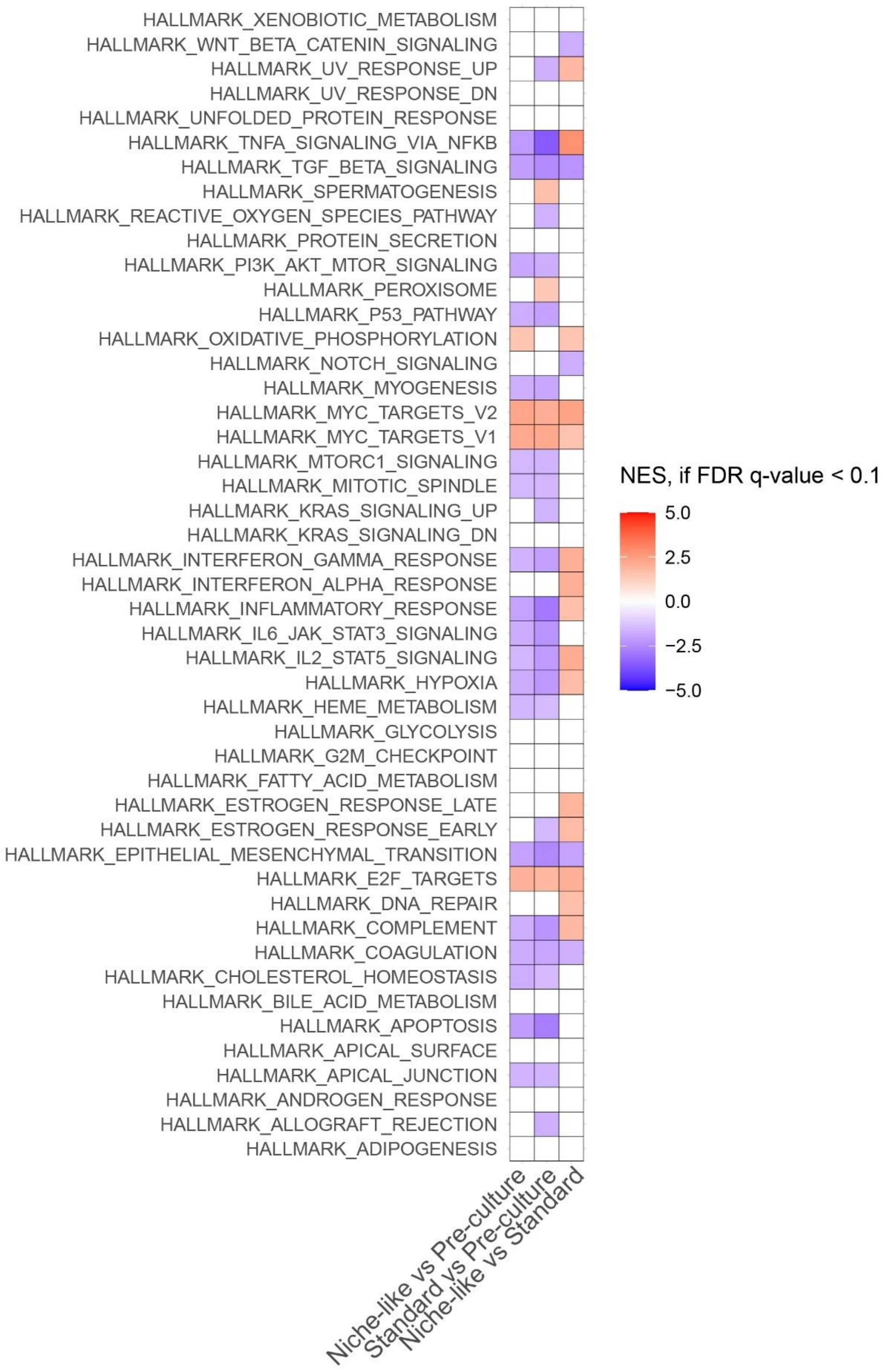
HALLMARK_XENOBIOTIC_METABOLISM
HALLMARK_WNT_BETA_CATENIN_SIGNALING
HALLMARK_UV_RESPONSE_UP
HALLMARK_UV_RESPONSE_DN
HALLMARK_UNFOLDED_PROTEIN_RESPONSE
HALLMARK_TNFA_SIGNALING_VIA_NFKB
HALLMARK_TGF_BETA_SIGNALING
HALLMARK_SPERMATOGENESIS
HALLMARK_REACTIVE_OXYGEN_SPECIES_PATHWAY
HALLMARK_PROTEIN_SECRETION
HALLMARK_PI3K_AKT_MTOR_SIGNALING
HALLMARK_PEROXISOME
HALLMARK_P53_PATHWAY
HALLMARK_OXIDATIVE_PHOSPHORYLATION
HALLMARK_NOTCH_SIGNALING
HALLMARK_MYOGENESIS
HALLMARK_MYC_TARGETS_V2
HALLMARK_MYC_TARGETS_V1
HALLMARK_MTORC1_SIGNALING
HALLMARK_MITOTIC_SPINDLE
HALLMARK_KRAS_SIGNALING_UP
HALLMARK_KRAS_SIGNALING_DN
HALLMARK_INTERFERON_GAMMA_RESPONSE
HALLMARK_INTERFERON_ALPHA_RESPONSE
HALLMARK_INFLAMMATORY_RESPONSE
HALLMARK_IL6_JAK_STAT3_SIGNALING
HALLMARK_IL2_STAT5_SIGNALING
HALLMARK_HYPOXIA
HALLMARK_HEME_METABOLISM
HALLMARK_GLYCOLYSIS
HALLMARK_G2M_CHECKPOINT
HALLMARK_FATTY_ACID_METABOLISM
HALLMARK_ESTROGEN_RESPONSE_LATE
HALLMARK_ESTROGEN_RESPONSE_EARLY
HALLMARK_EPITHELIAL_MESENCHYMAL_TRANSITION
HALLMARK_E2F_TARGETS
HALLMARK_DNA_REPAIR
HALLMARK_COMPLEMENT
HALLMARK_COAGULATION
HALLMARK_CHOLESTEROL_HOMEOSTASIS
HALLMARK_BILE_ACID_METABOLISM
HALLMARK_APOPTOSIS
HALLMARK_APICAL_SURFACE
HALLMARK_APICAL_JUNCTION
HALLMARK_ANDROGEN_RESPONSE
HALLMARK_ALLOGRAFT_REJECTION
HALLMARK_ADIPOGENESIS
Niche-like vs Pre-culture
Standard vs Pre-culture
Niche-like vs Standard
NES, if FDR q-value < 0.1
5.0
2.5
0.0
−2.5
−5.0

Supplementary Figure 6. Activity of the DNR-AraC combination alone or with fixed, low concentrations of each of 23 drugs (related to **Figure 5**) on the leukemic bulk of each of the 45 tested patients (**Supplementary Table 1**).

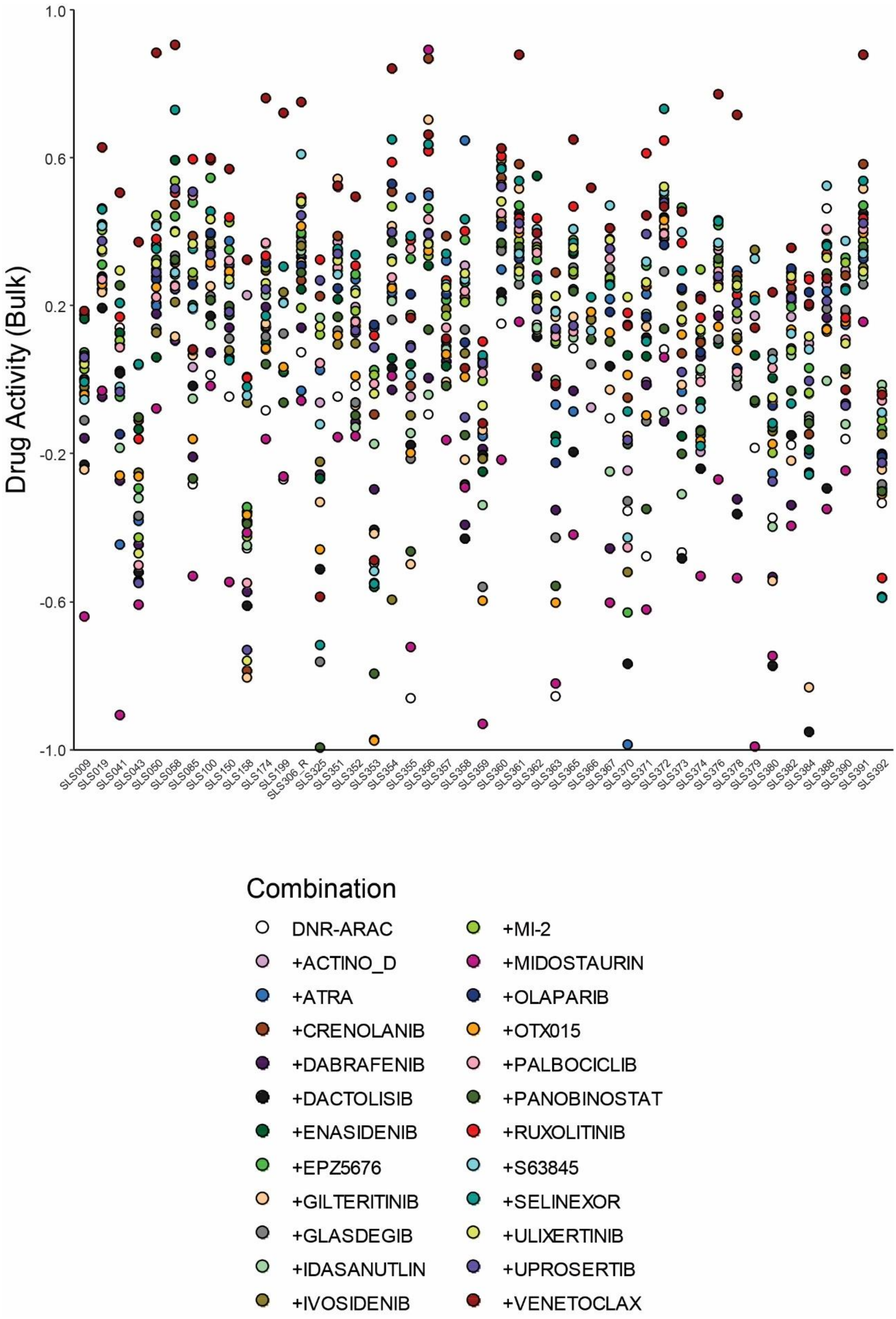

Supplementary Figure 7. Activity of the DNR-AraC combination alone or with fixed, low concentrations of each of 23 drugs (related to **Figure 5**) on LSCs of each of the 45 tested patients (**Supplementary Table 1**).

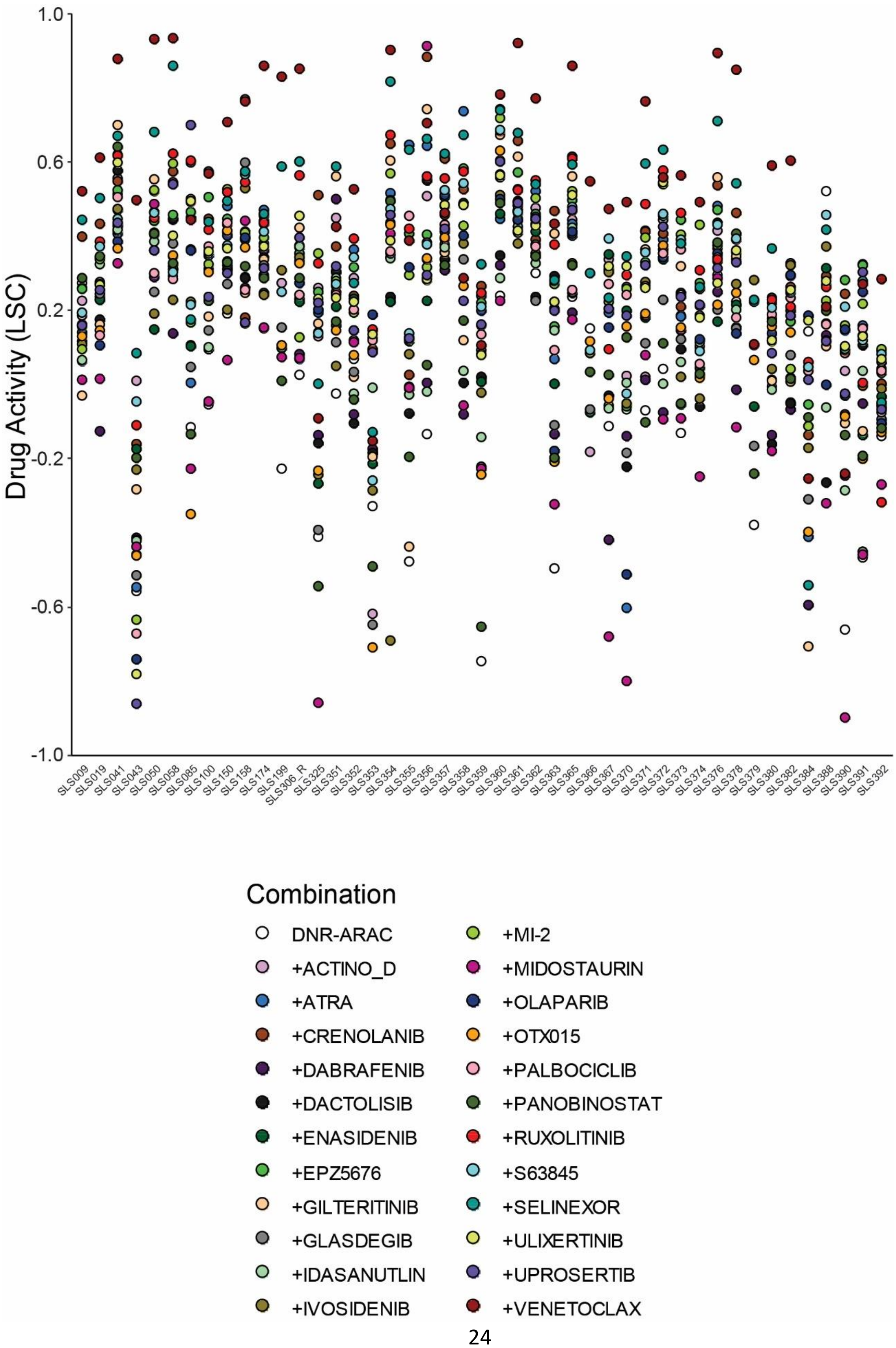

Supplementary Figure 8. Differentiating activity of the DNR-AraC combination alone or with fixed, low concentrations of each of 23 drugs (related to **Figure 5**) in each of the 45 tested patients (**Supplementary Table 1**).

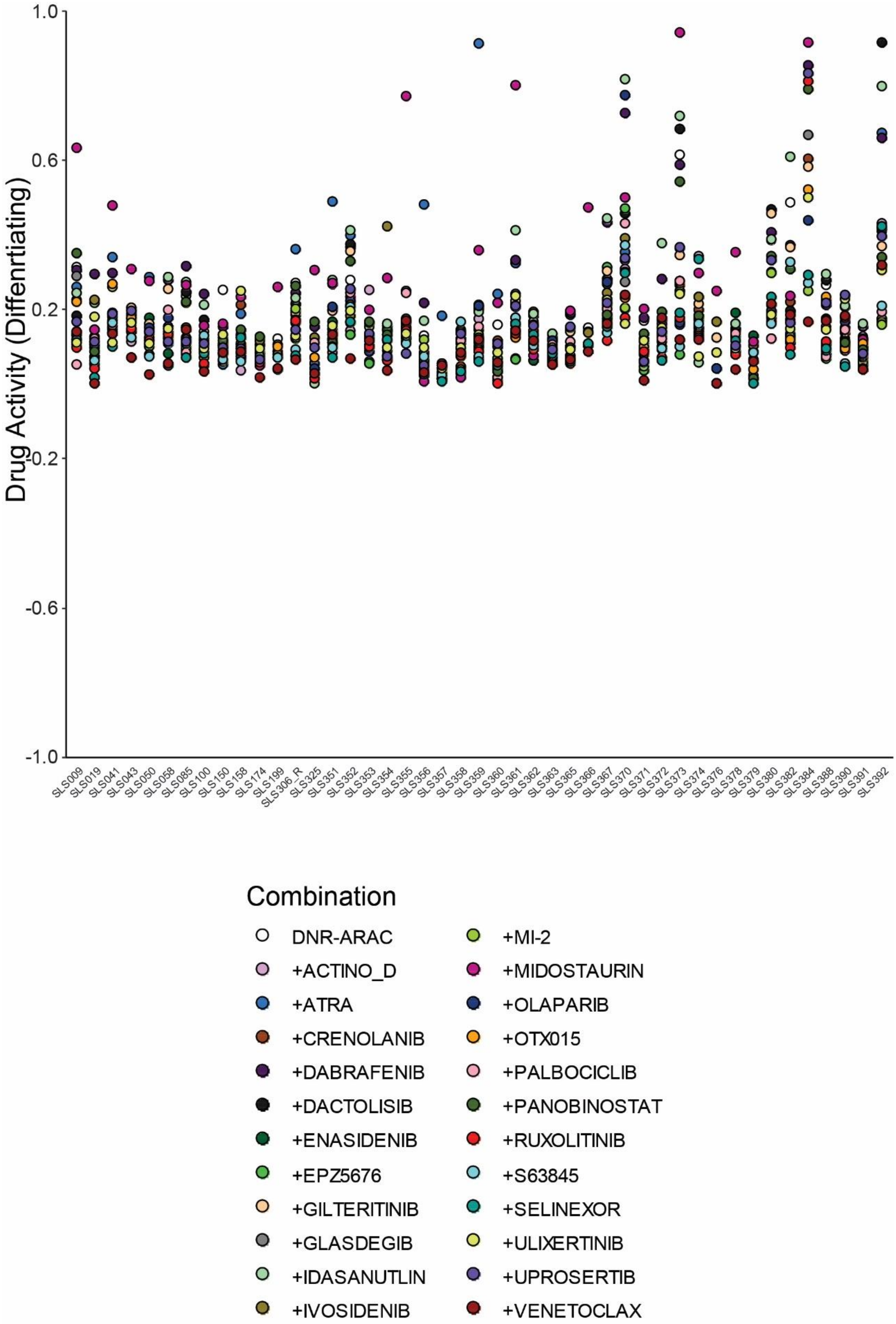

Supplementary Figure 9. Heatmap of chemogenomic tests from the DNR-AraC combination screen (**Figure 5**). Colors indicate the difference in the mean combination activity for each output between patients mutated and those wildtype for the given gene or gene group. *FDR q-value of t test < 0.05.

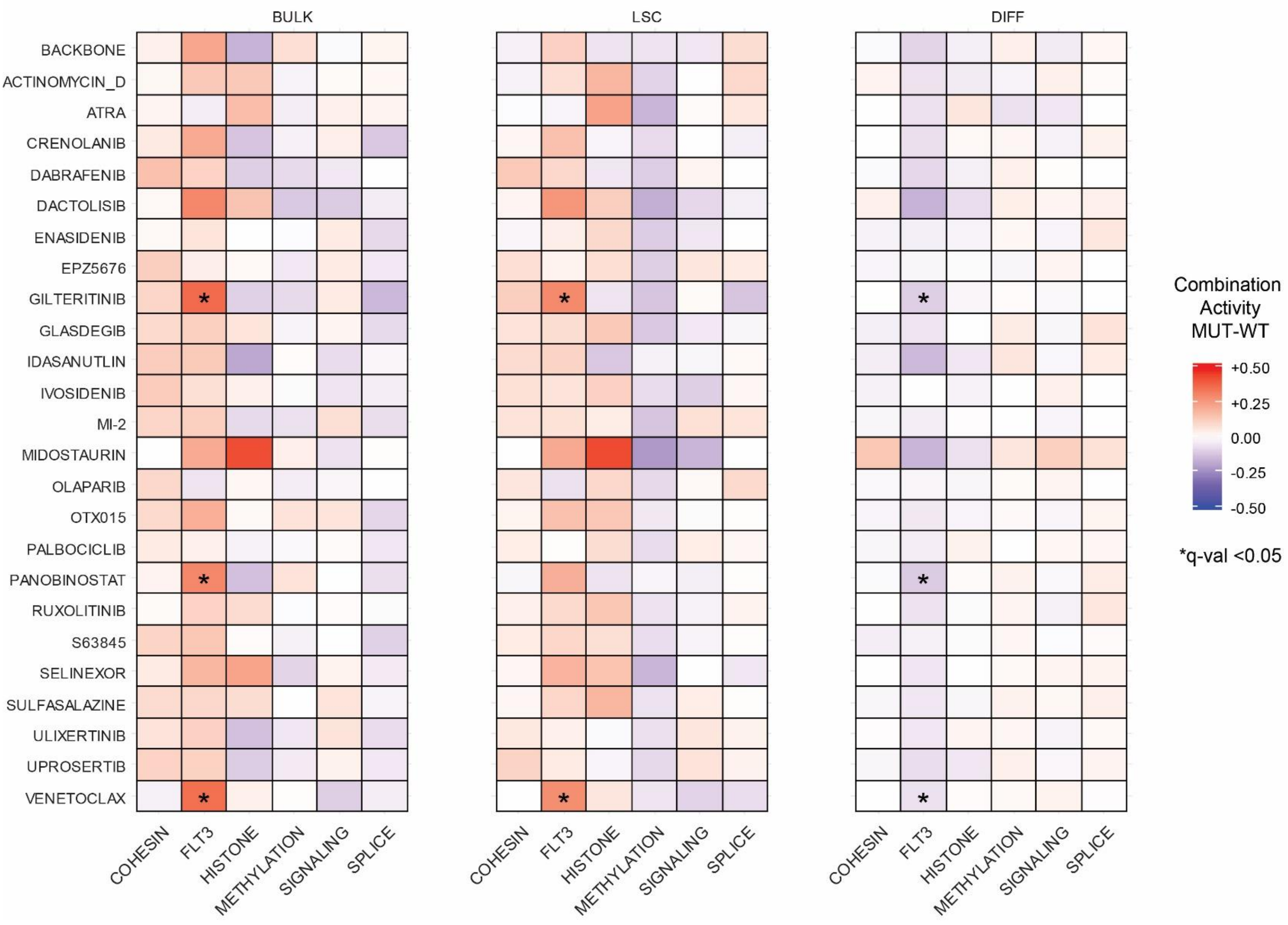

Supplementary Figure 10. Histogram of optimal combinations from the DNR-AraC screen (**Figure 5**) for activity against LSCs.

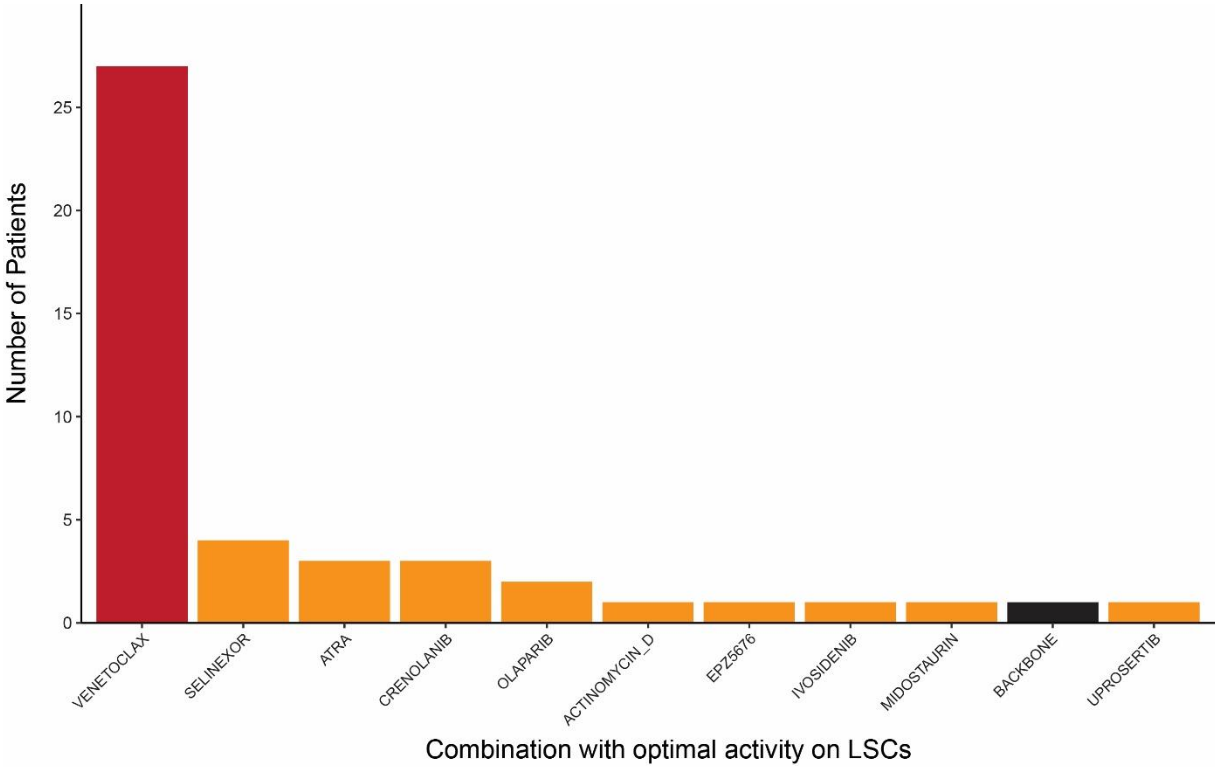

Supplement: Supplementary file 1 — Supplementary Methods and Figures (Clean) [file 41408_2022_689_MOESM1_ESM.pdf]
