# Supplementary material for: A multiparametric niche-like drug screening platform in acute myeloid leukemia
Source: Blood Cancer J. 2022 Jun 24;12(6):95. doi: 10.1038/s41408-022-00689-3 (PMC9232632; doi:10.1038/s41408-022-00689-3)
Supplement: Supplementary file 3 — Supplementary Table 4 [file 41408_2022_689_MOESM3_ESM.pdf]

Drug Response Pattern (Figure 2)

Plate_1

PBS	OTX015	AZA	DNR	AraC	PANO	DNR + AraC	AraC + VEN	AZA + MIDO	AZA + CRENO	AZA + ENA	
PBS	OTX015	AZA	DNR	AraC	PANO	DNR + AraC	AraC + VEN	AZA + MIDO	AZA + CRENO	AZA + ENA	
PBS	OTX015	AZA	DNR	AraC	PANO	DNR + AraC	AraC + VEN	AZA + MIDO	AZA + CRENO	AZA + ENA	
DMSO	ENA	AZA + VEN	MIDO	AZA + PANO	ActinoD	AZA + OTX015	VEN	ENA + CRENO	CRENO	MI-2 + EPZ	
DMSO	ENA	AZA + VEN	MIDO	AZA + PANO	ActinoD	AZA + OTX015	VEN	ENA + CRENO	CRENO	MI-2 + EPZ	
DMSO	ENA	AZA + VEN	MIDO	AZA + PANO	ActinoD	AZA + OTX015	VEN	ENA + CRENO	CRENO	MI-2 + EPZ	
Blasts Only	Blasts Only	Blasts Only	MSC only	MSC only	MSC only						

Plate_2

	DNR + AraC + CRENO		DNR + AraC + ENA		MI-2 + EPZ + OTX015		ATO + ATRA		PANO + OTX015		
	DNR + AraC + CRENO		DNR_C1 + AraC + ENA_C1		MI-2 + EPZ + OTX015		ATO + ATRA		PANO + OTX015		
	DNR + AraC + CRENO		DNR_C1 + AraC + ENA_C1		MI-2 + EPZ + OTX015		ATO + ATRA		PANO + OTX015		

Niche-like components mini-screen (Figure 3)

	no cytokines						plasma-like cytokines				
	DNR 2 + ARAC 20	DNR 0.2 + ARAC 2	DNR 0.02 + ARAC 0.2	DNR 0.002 + ARAC 0.02			DNR 0.002 + ARAC 0.02	DNR 0.02 + ARAC 0.2	DNR 0.2 + ARAC 2	DNR 2 + ARAC 20	
	AZA 50 + VEN 0.5	AZA 5 + VEN 0.05	AZA 0.5 + VEN 0.005	AZA 0.05 + VEN 0.0005	DMSO	DMSO	AZA 0.05 + VEN 0.0005	AZA 0.5 + VEN 0.005	AZA 5 + VEN 0.05	AZA 50 + VEN 0.5	
	AZA 50 + MIDO 20	AZA 5 + MIDO 2	AZA 0.5 + MIDO 0.2	AZA 0.05 + MIDO 0.02	DMSO	DMSO	AZA 0.05 + MIDO 0.02	AZA 0.5 + MIDO 0.2	AZA 5 + MIDO 2	AZA 50 + MIDO 20	
	ATO 25 + ATRA 10	ATO 2.5 + ATRA 1	ATO 0.25 + ATRA 0.1	ATO 0.025 + ATRA 0.1	DMSO	DMSO	ATO 0.025 + ATRA 0.1	ATO 0.25 + ATRA 0.1	ATO 2.5 + ATRA 1	ATO 25 + ATRA 10	
	SEL 2	SEL 0.2	SEL 0.02	SEL 0.002			SEL 0.002	SEL 0.02	SEL 0.2	SEL 2	
	PANO 0.02	PANO 0.002	PANO 0.0002	PANO 0.00002			PANO 0.00002	PANO 0.0002	PANO 0.002	PANO 0.02	

DNR-AraC Screen (Figure 5)

Plate_1

	DNR 2 + ARAC 40	DNR 0.2 + ARAC 4	DNR 0.02 + ARAC 0.4	DNR 0.002 + ARAC 0.04	DNR 0.0002 + ARAC 0.004	DNR 0.0002 + ARAC 0.004 + PANO 0.002	DNR 0.002 + ARAC 0.04 + PANO 0.002	DNR 0.02 + ARAC 0.4 + PANO 0.002	DNR 0.2 + ARAC 4 + PANO 0.002	DNR 2 + ARAC 40 + PANO 0.002	
	DNR 2 + ARAC 40 + GLASDE 0.01	DNR 0.2 + ARAC 4 + GLASDE 0.01	DNR 0.02 + ARAC 0.4 + GLASDE 0.01	DNR 0.002 + ARAC 0.04 + GLASDE 0.01	DNR 0.0002 + ARAC 0.004 + GLASDE 0.01	DNR 0.0002 + ARAC 0.004 + OTX015 0.02	DNR 0.002 + ARAC 0.04 + OTX015 0.02	DNR 0.02 + ARAC 0.4 + OTX015 0.02	DNR 0.2 + ARAC 4 + OTX015 0.02	DNR 2 + ARAC 40 + OTX015 0.02	
	DNR 2 + ARAC 40 + VEN 0.04	DNR 0.2 + ARAC 4 + VEN 0.04	DNR 0.02 + ARAC 0.4 + VEN 0.04	DNR 0.002 + ARAC 0.04 + VEN 0.04	DNR 0.0002 + ARAC 0.004 + VEN 0.04	DNR 0.0002 + ARAC 0.004 + RUXO 0.26	DNR 0.002 + ARAC 0.04 + RUXO 0.26	DNR 0.02 + ARAC 0.4 + RUXO 0.26	DNR 0.2 + ARAC 4 + RUXO 0.26	DNR 2 + ARAC 40 + RUXO 0.26	
	DNR 2 + ARAC 40 + MIDO 0.8	DNR 0.2 + ARAC 4 + MIDO 0.8	DNR 0.02 + ARAC 0.4 + MIDO 0.8	DNR 0.002 + ARAC 0.04 + MIDO 0.8	DNR 0.0002 + ARAC 0.004 + MIDO 0.8	DNR 0.0002 + ARAC 0.004 + SEL 0.4	DNR 0.002 + ARAC 0.04 + SEL 0.4	DNR 0.02 + ARAC 0.4 + SEL 0.4	DNR 0.2 + ARAC 4 + SEL 0.4	DNR 2 + ARAC 40 + SEL 0.4	
	DNR 2 + ARAC 40 + AG221 6	DNR 0.2 + ARAC 4 + AG221 6	DNR 0.02 + ARAC 0.4 + AG221 6	DNR 0.002 + ARAC 0.04 + AG221 6	DNR 0.0002 + ARAC 0.004 + AG221 6	DNR 0.0002 + ARAC 0.004 + ACT_D 0.004	DNR 0.002 + ARAC 0.04 + ACT_D 0.004	DNR 0.02 + ARAC 0.4 + ACT_D 0.004	DNR 0.2 + ARAC 4 + ACT_D 0.004	DNR 2 + ARAC 40 + ACT_D 0.004	
	DNR 2 + ARAC 40 + IVO 0.34	DNR 0.2 + ARAC 4 + IVO 0.34	DNR 0.02 + ARAC 0.4 + IVO 0.34	DNR 0.002 + ARAC 0.04 + IVO 0.34	DNR 0.0002 + ARAC 0.004 + IVO 0.34	DNR 0.0002 + ARAC 0.004 + S63845 0.0034	DNR 0.002 + ARAC 0.04 + S63845 0.0034	DNR 0.02 + ARAC 0.4 + S63845 0.0034	DNR 0.2 + ARAC 4 + S63845 0.0034	DNR 2 + ARAC 40 + S63845 0.0034	
					DMSO	DMSO					

Plate_2

	DNR 2 + ARAC 40 + IDASA 0.048	DNR 0.2 + ARAC 4 + IDASA 0.048	DNR 0.02 + ARAC 0.4 + IDASA 0.048	DNR 0.002 + ARAC 0.04 + IDASA 0.048	DNR 0.0002 + ARAC 0.004 + IDASA 0.048	DNR 0.0002 + ARAC 0.004 + DABRA 1.5	DNR 0.002 + ARAC 0.04 + DABRA 1.5	DNR 0.02 + ARAC 0.4 + DABRA 1.5	DNR 0.2 + ARAC 4 + DABRA 1.5	DNR 2 + ARAC 40 + DABRA 1.5	
	DNR 2 + ARAC 40 + DACTO 0.34	DNR 0.2 + ARAC 4 + DACTO 0.34	DNR 0.02 + ARAC 0.4 + DACTO 0.34	DNR 0.002 + ARAC 0.04 + DACTO 0.34	DNR 0.0002 + ARAC 0.004 + DACTO 0.34	DNR 0.0002 + ARAC 0.004 + GILTE 0.1	DNR 0.002 + ARAC 0.04 + GILTE 0.1	DNR 0.02 + ARAC 0.4 + GILTE 0.1	DNR 0.2 + ARAC 4 + GILTE 0.1	DNR 2 + ARAC 40 + GILTE 0.1	
	DNR 2 + ARAC 40 + UPRO 0.14	DNR 0.2 + ARAC 4 + UPRO 0.14	DNR 0.02 + ARAC 0.4 + UPRO 0.14	DNR 0.002 + ARAC 0.04 + UPRO 0.14	DNR 0.0002 + ARAC 0.004 + UPRO 0.14	DNR 0.0002 + ARAC 0.004 + MI-2 2	DNR 0.002 + ARAC 0.04 + MI-2 2	DNR 0.02 + ARAC 0.4 + MI-2 2	DNR 0.2 + ARAC 4 + MI-2 2	DNR 2 + ARAC 40 + MI-2 2	
	DNR 2 + ARAC 40 + OLA 0.012	DNR 0.2 + ARAC 4 + OLA 0.012	DNR 0.02 + ARAC 0.4 + OLA 0.012	DNR 0.002 + ARAC 0.04 + OLA 0.012	DNR 0.0002 + ARAC 0.004 + OLA 0.012	DNR 0.0002 + ARAC 0.004 + EPZ 2	DNR 0.002 + ARAC 0.04 + EPZ 2	DNR 0.02 + ARAC 0.4 + EPZ 2	DNR 0.2 + ARAC 4 + EPZ 2	DNR 2 + ARAC 40 + EPZ 2	
	DNR 2 + ARAC 40 + PALBO 0.02	DNR 0.2 + ARAC 4 + PALBO 0.02	DNR 0.02 + ARAC 0.4 + PALBO 0.02	DNR 0.002 + ARAC 0.04 + PALBO 0.02	DNR 0.0002 + ARAC 0.004 + PALBO 0.02	DNR 0.0002 + ARAC 0.004 + ATRA 1.6	DNR 0.002 + ARAC 0.04 + ATRA 1.6	DNR 0.02 + ARAC 0.4 + ATRA 1.6	DNR 0.2 + ARAC 4 + ATRA 1.6	DNR 2 + ARAC 40 + ATRA 1.6	
	DNR 2 + ARAC 40 + ULIX 0.56	DNR 0.2 + ARAC 4 + ULIX 0.56	DNR 0.02 + ARAC 0.4 + ULIX 0.56	DNR 0.002 + ARAC 0.04 + ULIX 0.56	DNR 0.0002 + ARAC 0.004 + ULIX 0.56	DNR 0.0002 + ARAC 0.004 + CRENO 0.1	DNR 0.002 + ARAC 0.04 + CRENO 0.1	DNR 0.02 + ARAC 0.4 + CRENO 0.1	DNR 0.2 + ARAC 4 + CRENO 0.1	DNR 2 + ARAC 40 + CRENO 0.1	
					DMSO	DMSO					
