# Supplementary material for: A multiparametric niche-like drug screening platform in acute myeloid leukemia
Source: Blood Cancer J. 2022 Jun 24;12(6):95. doi: 10.1038/s41408-022-00689-3 (PMC9232632; doi:10.1038/s41408-022-00689-3)
Supplement: Supplementary file 2 — Supplementary Tables 1–3 and 5-12 [file 41408_2022_689_MOESM2_ESM.pdf]

Supplementary Table 1. Patients samples and metadata

	Sex	Age, years	Stage	WBC, x109/L	Sourc	
**UPN**	**Clinical**					
SLS009	F	69	Diagnosis	1.5	BM	89
SLS014	F	56	Diagnosis	2.73	BM	60
SLS019	F	62	Diagnosis	35.9	BM	92
SLS020	F	28	Diagnosis	3.28	BM	63
SLS025	F	34	Diagnosis	4.2	BM	62
SLS032	M	70	Diagnosis	13.61	BM	68
SLS033	M	64	Diagnosis	68.01	BM	90
SLS036	M	29	Diagnosis	35.06	BM	46
SLS037	M	51	Relapse	0.31	BM	10
SLS041	F	59	Diagnosis	39	BM	93
SLS043	M	29	Diagnosis	39.8	BM	90
SLS048	F	46	Diagnosis	5.35	BM	41
SLS050	F	50	Diagnosis	142.6	BM	90
SLS058	F	75	Diagnosis	22	BM	65
SLS068	M	71	Diagnosis	96.73	BM	45
SLS070	M	35	Diagnosis	28.74	BM	73
SLS083	F	22	Diagnosis	20.47	BM	64
SLS085	F	60	Diagnosis	37	BM	90
SLS094	M	52	Relapse	1.2	BM	33
SLS095	F	46	Diagnosis	1.19	BM	7
SLS100	F	65	Diagnosis	107.4	PB	99
SLS107	M	22	Diagnosis	6.29	PB	63
SLS110	M	48	Diagnosis	16.39	BM	82
SLS134	M	60	Diagnosis	39.7	BM	67
SLS149	F	27	Diagnosis	21	BM	56
SLS150	F	76	Diagnosis	19.8	PB	19
SLS158	M	59	Diagnosis	4.1	BM	27
SLS174	F	52	Diagnosis	6.7	BM	74
SLS199	F	70	Diagnosis	97	PB	48
SLS244	M	50	Diagnosis	99.72	BM	80
SLS301	F	57	Relapse	4.41	PB	71
SLS302	F	59	Diagnosis	104.98	PB	79
SLS305	F	78	Relapse	11.29	PB	25
SLS306	F	47	Diagnosis	21.1	BM	73
SLS306R	F	47	Relapse	1.99	PB	64

SLS325	F	61	Diagnosis	78.7	BM	45
SLS327	M	64	Diagnosis	254.62	BM	92
SLS343	M	63	Diagnosis	88	PB	87
SLS345	F	25	Diagnosis	28.42	PB	59
SLS346	M	58	Diagnosis	81.26	BM	89
SLS351	F	16	Diagnosis	14.5	BM	60
SLS352	M	71	Diagnosis	4.2	BM	82
SLS353	F	78	Diagnosis	34	PB	69
SLS354	F	75	Diagnosis	117	PB	95
SLS355	M	70	Diagnosis	105.3	BM	26
SLS356	M	34	Diagnosis	125.7	BM	87
SLS357	M	56	Diagnosis	8.1	PB	47
SLS358	M	58	Diagnosis	88	BM	94
SLS359	M	41	Diagnosis	4.7	PB	20
SLS360	F	69	Diagnosis	39.7	PB	91
SLS361	M	55	Diagnosis	68.4	PB	82
SLS362	F	76	Diagnosis	69.7	PB	61
SLS363	M	72	Diagnosis	49	BM	77
SLS365	M	45	Diagnosis	53.4	BM	64
SLS366	F	20	Diagnosis	14.7	PB	58
SLS367	M	43	Diagnosis	59	BM	60
SLS370	M	51	Diagnosis	78	BM	81
SLS371	F	65	Diagnosis	23.5	PB	78
SLS372	M	73	Diagnosis	134	BM	48
SLS373	M	46	Diagnosis	19.8	BM	40
SLS374	M	60	Diagnosis	0.7	BM	20
SLS376	F	62	Diagnosis	136	PB	94
SLS378	M	68	Diagnosis	108	BM	94
SLS379	F	18	Diagnosis	37.7	BM	80
SLS380	F	37	Diagnosis	5.8	BM	48
SLS381	F	71	Diagnosis	49.01	BM	90
SLS382	F	78	Diagnosis	1.7	PB	43
SLS384	F	68	Diagnosis	64.7	BM	40
SLS388	F	69	Diagnosis	146	BM	97
SLS389	M	48	Diagnosis	27.6	BM	75
SLS390	F	64	Diagnosis	293	BM	81
SLS391	M	73	Diagnosis	143	PB	97
SLS392	M	54	Diagnosis	2.3	PB	28
SLS393	F	76	Diagnosis	120	BM	80

e

Blasts, %

FAB	CD14 Expression, %	CD15 Expression, %	Karyotype	*NPM1*	*FLT3*	
Differenciation Arrest			Genetic			
M1	0	37	46,XX	MUT	WT	NA
M2	0	13	9, add(11)del(	WT	WT	
M1	0	11	46,XX	MUT	ITD	1
M2	0	26	21)(q22;q22),	WT	WT	
M2	0	6	X,t(6;9)(p22;	WT	ITD	0.82
M0	0	1	Y,del(13)(q13	WT	WT	
M5	20	43	2?4;q2?2q2?	WT	ITD	0.3
M4Eo	3	8	Y,inv(16)(p13q	WT	WT	
NA	1	82	46,XY	WT	WT	
M5	5	87	46,XX	MUT	ITD	0.5
M1	0	11	46,XY	MUT	ITD & TKD	0.66
M4Eo	4	29	nv(16)(p13q2	WT	WT	
M1	0	60	46,XX	MUT	ITD	0.65
M2	0	8	46XX,+4	MUT	WT	NA
M4	1	8	46,XY	WT	ITD	0.48
M4Eo	0	54	Y,inv(16)(p13	WT	WT	
M4Eo	10	41	X,inv(16)(p13	WT	WT	
M5	44	87	46,XX	MUT	WT	NA
NA	2	42	1?3),-16,+ma	WT	WT	
NA	2	9	NA	WT	WT	
M1	0	1	46,XX	MUT	WT	NA
M5	3	98	,der(9)t(2;9)(	WT	WT	
M1	0	97	46,XY	WT	WT	
NA	10	0	46,XY	WT	WT	
M4Eo	0	0	X,inv(16)(p13	WT	WT	
M4	0	0	46,XX	MUT	TKD	NA
M2	0	0	46,XY	WT	WT	NA
M1	0	0	46,XX	MUT	ITD	0.1
M1	0	0	46,XX	MUT	ITD	1.5
M5	0	0	46,XY	MUT	WT	
M5	0	0	,del(15)(q1?4	MUT	WT	
M1	0	0	46,XX	MUT	WT	
M5	11	90	47,XX,+4	MUT	WT	
M2	0	0	46,XX	MUT	ITD	0.36
M1	0	0	del(5)add(5)(	MUT	ITD	8.7

M2	1	27	46,XX	MUT	WT	NA
M5	0	8	46,XY	MUT	ITD	0.49
M4	0	0	47XY, +8	MUT	ITD	0.58
M4	0	0	46,XX	MUT	ITD & TKD	0.05
M1	0	0	Y,del(20)(q11	WT	ITD	0.19
M2	0	15	46XX, t(5;11)	WT	WT	NA
M4	18	60	46,XY	WT	WT	NA
M1	0	7	46XX, t(6;9)	MUT	WT	NA
M1	0	6	46,XX	MUT	ITD	0.86
M2	0	0	46,XY	WT	WT	NA
M5	0	0	46,XY	WT	ITD	0.6
M2	1	2	16;21)(q24;q2	WT	WT	NA
M5	15	53	t(10;11); 47X	WT	WT	NA
M4	0	0	dd(7)(q3?2),-	WT	WT	NA
M1	0	0	46,XX	MUT	ITD	0.4
M2	1	0	46,XY	MUT	ITD	0.68
M4	0	0	46,XX	MUT	WT	NA
M1	1	21	46,XY	MUT	ITD	0.9
M4	0	26	46,XY	MUT	TKD	NA
M2	0	20	46,XX	MUT	WT	NA
M4	3	25	46,XY	MUT	WT	NA
M5	55	86	46XY, isoc 4p	MUT	TKD	NA
M2	0	5	46,XX	MUT	TKD	NA
M4	0	0	46,XY	MUT	WT	NA
M4	10	98	46,XY	MUT	WT	NA
M1	0	0	46XY, +Y	MUT	WT	NA
M1	0	0	46,XX	MUT	ITD	0.9
M1	0	0	46,XY	MUT	ITD	0.26
M5	2	35	46,XX	MUT	TKD	NA
M4	0	35	46,XX	MUT	WT	NA
M1	0	1	46,XX	MUT	WT	
M1	0	0	46,XX	MUT	WT	NA
M4	0	17	46,XX	MUT	WT	NA
M1	0	1	46,XX	MUT	ITD	0.55
M5	42	92	46,XY	MUT	WT	
M5	51	43	46,XX	MUT	WT	NA
M1	0	0	46,XY	MUT	ITD	0.02
M2	0	0	46XY, del17p	MUT	WT	NA
M4	NA	NA	46,XX	MUT	ITD	0.66

FLT3-ITD Allele Ratio	IDH1	IDH2	ELN 2017 Risk Category	EOI Evaluation	NPM1_MRD	EFS_Time (
			Outcome			
WT	WT	Fav	CR	0.000036	1234	0
WT	WT	Int				
MUT	WT	Int	CR	0.0001	731	1
WT	WT	Fav				
WT	WT	Adv				
WT	WT	Int				
WT	WT	Int				
WT	WT	Fav				
WT	WT	Fav				
WT	WT	Int	CR	NA	546	0
WT	R140Q	Int	CR	0.000001	159	0
WT	WT	Fav				
WT	WT	Int	CR	0.0012	1158	0
WT	WT	Fav	CR	NA	1182	0
WT	WT	Int				
WT	WT	Fav				
WT	WT	Fav				
MUT	WT	Fav	CR	NA	999	1
WT	WT	Adv				
WT	WT	NA				
MUT	WT	Fav	ED	NA	2	1
WT	WT	Int				
WT	WT	Fav				
WT	WT	Fav				
WT	WT	Fav				
WT	WT	Fav	CR	0.000001	835	0
WT	R140Q	Adv	MLFS	NA	49	1
WT	R140Q	Int	PIF	NA	79	1
WT	WT	Int	PIF	NA	47	1
WT	WT	Fav				
WT	WT	Int				
WT	R140Q	Fav				
WT	WT	Fav				
WT	WT	Fav	CR	0.0043	159	1
WT	WT	Int				

WT	WT	Fav	CR	0.000061	290	1
NA	NA	Fav				
WT	WT	Int				
WT	WT	Fav				
WT	WT	Int				
WT	WT	Adv	PIF	NA	70	1
WT	R140Q	Adv	PIF	NA	42	1
WT	WT	Fav	PIF	NA	63	1
MUT	WT	Int	CR	0.000001	469	1
WT	WT	Adv	PIF	NA	52	1
WT	WT	Adv	PIF	NA	37	1
WT	WT	Int	ED	NA	20	1
WT	WT	Adv	ED	NA	20	1
WT	WT	Adv	PIF	NA	44	1
WT	WT	Int	ED	NA	30	1
WT	WT	Int	CR	0.000001	203	0
MUT	WT	Fav	CR	0.00032	852	1
MUT	WT	Int	CR	NA	378	1
MUT	WT	Fav	CR	0.00006	870	1
WT	WT	Fav	CR	0.000001	686	0
WT	WT	Fav	CR	0.0046	171	0
WT	WT	Fav	CR	0.0075	142	0
WT	WT	Fav	CR	NA	335	1
WT	WT	Fav	CR	0.000001	517	1
WT	WT	Fav	CR	0.0016	397	1
WT	WT	Fav	CR	0.0063	1219	0
WT	WT	Adv	CR	NA	632	1
WT	R140Q	Fav	CR	NA	849	1
MUT	WT	Fav	CR	0.07	179	1
WT	WT	Int	CR	0.000001	295	1
NA	NA	Fav				
MUT	WT	Fav	CR	0.000001	588	1
WT	R140Q	Fav	CR	NA	335	1
WT	WT	Int	ED	NA	15	1
NA	NA	Fav				
WT	WT	Fav	ED	NA	22	1
WT	R140Q	Fav	ED	NA	24	1
WT	WT	Int	CR	0.000001	125	0
WT	WT	Int				

LSC_count_ex_vivo	LTC-IC	drug_response_patterns	plasma_amino_acids	plasma_cytokines	conditioned_medium_ami	nich
					**Experiments**	
			x	x		
			x	x		
	x		x	x		
	x		x	x		
			x			
			x	x		
			x	x		
			x	x		
			x	x		
					x	
			x		x	
			x	x		
			x	x	x	x
					x	
			x			
			x	x		
			x	x		
			x	x		
			x	x		
			x	x		
			x	x		
			x	x		
			x	x		
			x	x		
			x	x		
			x	x	x	
		x				
x						
		x				
		x				

(days) EFS_event

						x
						x
x						
x						
	x					

...ino_acids_cytokines

...ne-like_components_mini-screen

DNA-Seq	RNA-Seq	sc-RNA-Seq	DNR-ARAC_Screen
			x
			x
			x
			x
			x
			x
			x
			x
			x
x			x
			x
			x
	x		
			x

x			x
x			x
x			x
x			x
	x		x
x			x
x			x
			x
			x
			x
			x
			x
			x
			x
			x
			x
			x
			x
			x
			x
			x
			x
			x
			x
			x
	x		
			x
			x
			x
	x		
			x
			x
			x
		x	

Supplementary Table 2. Gene m

UPN	GENE
SLS361	*BRCC3*
SLS361	*NPM1*
SLS361	*TET2*
SLS361	*SRSF2*
SLS361	*PRPF8*
SLS380	*WT1*
SLS380	*WT1*
SLS380	*WT1*
SLS380	*FLT3*
SLS380	*NPM1*
SLS380	*DNMT3A*
SLS390	*NPM1*
SLS390	*RAD21*
SLS390	*DNMT3A*
SLS390	*CBL*
SLS325	*TET2*
SLS325	*SRSF2*
SLS325	*DNMT3A*
SLS325	*NRAS*
SLS325	*NPM1*
SLS325	*CEBPA*
SLS371	*SMC1A*
SLS371	*NPM1*
SLS371	*FLT3*
SLS373	*SMC3*
SLS373	*RIT1*
SLS373	*NPM1*
SLS352	*SRSF2*
SLS352	*IDH2*
SLS352	*ASXL1*
SLS367	*NRAS*
SLS367	*NPM1*
SLS370	*PTPN11*
SLS370	*TET2*
SLS370	*FLT3*
SLS370	*NPM1*
SLS384	*CEBPA*
SLS384	*NPM1*
SLS384	*IDH2*
SLS384	*SRSF2*
SLS379	*SMC3*
SLS379	*FLT3*
SLS379	*NPM1*
SLS379	*IDH1*
SLS376	*SMC1A*
SLS376	*TP53*
SLS376	*KRAS*

SLS305	*NPM1*
SLS009	*GATA2*
SLS009	*TET2*
SLS009	*TET2*
SLS009	*NPM1*
SLS354	*FLT3*
SLS354	*NPM1*
SLS354	*NF1*
SLS354	*DNMT3A*
SLS354	*IDH1*
SLS354	*KIT*
SLS014	*NO_MUTATION*
SLS019	*FLT3*
SLS019	*IDH1*
SLS019	*DNMT3A*
SLS019	*NPM1*
SLS019	*FLT3*
SLS020	*RAD21*
SLS020	*EZH2*
SLS020	*EZH2*
SLS020	*WT1*
SLS025	*FLT3*
SLS366	*WT1*
SLS366	*WT1*
SLS366	*NRAS*
SLS366	*NPM1*
SLS366	*TET2*
SLS032	*NRAS*
SLS032	*NRAS*
SLS032	*SF3B1*
SLS032	*EP300*
SLS032	*BCORL1*
SLS032	*STAG2*
SLS033	*DNMT3A*
SLS033	*FLT3*
SLS036	*NRAS*
SLS365	*PTPN11*
SLS365	*FLT3*
SLS365	*IDH1*
SLS365	*NPM1*
SLS041	*NPM1*
SLS041	*FLT3*
SLS041	*SF3B1*
SLS043	*FLT3*
SLS043	*NPM1*
SLS043	*IDH2*
SLS048	*NRAS*
SLS048	*WT1*
SLS050	*NPM1*
SLS050	*DNMT3A*

SLS351	*RUNX1*
SLS351	*PTPN11*
SLS058	*STAG2*
SLS058	*NPM1*
SLS058	*FLT3*
SLS357	*KRAS*
SLS357	*NRAS*
SLS357	*PTPN11*
SLS357	*SMC3*
SLS357	*PTPN11*
SLS357	*SF3B1*
SLS068	*FLT3*
SLS068	*DNMT3A*
SLS068	*SRSF2*
SLS083	*BCORL1*
SLS085	*FLT3*
SLS085	*KRAS*
SLS085	*PTPN11*
SLS085	*NF1*
SLS085	*IDH1*
SLS085	*DNMT3A*
SLS085	*NPM1*
SLS085	*RAD21*
SLS085	*DNMT3A*
SLS353	*NPM1*
SLS353	*TET2*
SLS353	*TET2*
SLS353	*DNMT3A*
SLS388	*FLT3*
SLS388	*NPM1*
SLS388	*TET2*
SLS100	*IDH1*
SLS100	*DNMT3A*
SLS100	*NPM1*
SLS107	*NO_MUTATION*
SLS374	*U2AF1*
SLS374	*BRAF*
SLS374	*NPM1*
SLS374	*STAG2*
SLS374	*PHF6*
SLS110	*TET2*
SLS110	*GATA2*
SLS110	*CEBPA*
SLS110	*CEBPA*
SLS363	*FLT3*
SLS363	*IDH1*
SLS363	*NPM1*
SLS363	*DNMT3A*
SLS358	*KRAS*
SLS358	*NRAS*

SLS134	*CEBPA*
SLS134	*TET2*
SLS134	*CEBPA*
SLS134	*CSF3R*
SLS378	*FLT3*
SLS378	*CSF3R*
SLS378	*SRSF2*
SLS378	*IDH2*
SLS378	*NPM1*
SLS149	*NRAS*
SLS149	*NRAS*
SLS150	*WT1*
SLS150	*FLT3*
SLS150	*FLT3*
SLS150	*NPM1*
SLS150	*DNMT3A*
SLS158	*DNMT3A*
SLS158	*IDH2*
SLS158	*SRSF2*
SLS158	*ASXL1*
SLS174	*FLT3*
SLS174	*NPM1*
SLS174	*IDH2*
SLS362	*PTPN11*
SLS362	*IDH1*
SLS362	*DNMT3A*
SLS362	*NPM1*
SLS382	*IDH1*
SLS382	*NPM1*
SLS392	*WT1*
SLS392	*PTPN11*
SLS392	*NPM1*
SLS392	*STAG2*
SLS199	*PHF6*
SLS199	*FLT3*
SLS199	*WT1*
SLS199	*NPM1*
SLS199	*FLT3*
SLS360	*FLT3*
SLS360	*RAD21*
SLS360	*EP300*
SLS360	*NPM1*
SLS360	*DNMT3A*
SLS356	*FLT3*
SLS356	*FLT3*
SLS356	*DNMT3A*
SLS356	*BCOR*
SLS359	*TP53*
SLS391	*NRAS*
SLS391	*FLT3*

SLS391	*FLT3*
SLS391	*NPM1*
SLS391	*SRSF2*
SLS391	*IDH2*
SLS355	*RUNX1*
SLS355	*EZH2*
SLS355	*SF3B1*
SLS355	*DNMT3A*
SLS355	*CBL*
SLS372	*CBL*
SLS372	*KRAS*
SLS372	*NPM1*
SLS372	*ASXL1*
SLS372	*TET2*
SLS372	*SRSF2*
SLS372	*TET2*
SLS372	*CBL*
SLS244	*EZH2*
SLS244	*PTPN11*
SLS244	*NRAS*
SLS244	*NPM1*
SLS244	*DNMT3A*
SLS306	*FLT3*
SLS306	*SMC3*
SLS306	*DNMT3A*
SLS306	*NPM1*
SLS302	*NPM1*
SLS302	*IDH2*
SLS306R	*FLT3*
SLS306R	*NPM1*
SLS306R	*SMC3*
SLS306R	*DNMT3A*
SLS070	*NO_MUTATION*
SLS037	*CEBPA*
SLS037	*CEBPA*
SLS095	*NO_MUTATION*
SLS094	*WT1*
SLS094	*WT1*
SLS094	*WT1*
SLS094	*NRAS*
SLS301	*RAD21*
SLS301	*CUX1*
SLS301	*NRAS*
SLS301	*DNMT3A*
SLS301	*KIT*
SLS301	*GATA2*
SLS301	*NPM1*
SLS343	*DNMT3A*
SLS343	*FLT3*
SLS343	*NPM1*

SLS345	*FLT3*
SLS345	*FLT3*
SLS345	*WT1*
SLS345	*FLT3*
SLS345	*NPM1*
SLS393	*TET2*
SLS393	*TET2*
SLS393	*DNMT3A*
SLS393	*FLT3*
SLS393	*NPM1*

utations identified by targeted sequencing

MUTATION
BRCC3:NM_024332:exon1:c.C47T:p.S16F
NPM1:NM_002520:exon11:c.859_860insTCTG:p.L287fs
TET2:NM_001127208:exon3:c.C1648T:p.R550X
SRSF2:NM_003016:exon1:c.C284T:p.P95L
PRPF8:NM_006445:exon10:c.T1376C:p.L459P
WT1:NM_024426:exon1:c.C338T:p.A113V
WT1:NM_024426:exon1:c.G337C:p.A113P
WT1:NM_024426:exon1:c.322_334del:p.F108fs
FLT3:NM_004119:exon16:c.G1992T:p.M664I
NPM1:NM_002520:exon11:c.861_862insTGTA:p.L287fs
DNMT3A:NM_175629:exon19:c.C2309T:p.S770L
NPM1:NM_002520:exon11:c.861_862insTGTA:p.L287fs
RAD21:NM_006265:splice
DNMT3A:NM_175629:exon18:c.T2147A:p.V716D
CBL:NM_005188:exon8:c.T1139C:p.L380P
TET2:NM_001127208:exon6:c.A3656G:p.H1219R
SRSF2:NM_003016:exon1:c.C284A:p.P95H
DNMT3A:NM_175629:exon8:c.T887G:p.V296G
NRAS:NM_002524:exon2:c.G35A:p.G12D
NPM1:NM_002520:exon11:c.863_864insCATG:p.L287fs
CEBPA:NM_004364:exon1:c.751dupA:p.S251fs
SMC1A:NM_006306:exon13:c.2082_2117del:p.694_706del
NPM1:NM_002520:exon11:c.859_860insTCTG:p.L287fs
FLT3:NM_004119:exon20:c.G2503T:p.D835Y
SMC3:NM_005445:exon15:c.1461_1463del:p.487_488del
RIT1:NM_001256820:exon3:c.G121A:p.A41T
NPM1:NM_002520:exon11:c.859_860insTCTG:p.L287fs
SRSF2:NM_003016:exon1:c.C284A:p.P95H
IDH2:NM_002168:exon4:c.G419A:p.R140Q
ASXL1:NM_015338:exon12:c.2254dupG:p.V751fs
NRAS:NM_002524:exon2:c.G35T:p.G12V
NPM1:NM_002520:exon11:c.861_862insTGCA:p.L287fs
PTPN11:NM_002834:exon3:c.A182C:p.D61A
TET2:NM_001127208:exon3:c.1553dupT:p.I518fs
FLT3:NM_004119:exon20:c.G2503T:p.D835Y
NPM1:NM_002520:exon11:c.861_862insTGCA:p.L287fs
CEBPA:NM_004364:exon1:c.201_202insCTAC:p.I68fs
NPM1:NM_002520:exon9:c.761_771del:p.S254fs
IDH2:NM_002168:exon4:c.G419A:p.R140Q
SRSF2:NM_003016:exon1:c.C284G:p.P95R
SMC3:NM_005445:exon13:c.A1133C:p.K378T
FLT3:NM_004119:exon20:c.2502_2504del:p.834_835del
NPM1:NM_002520:exon11:c.859_860insTCTG:p.L287fs
IDH1:NM_005896:exon4:c.G395A:p.R132H
SMC1A:NM_006306:exon4:c.G413A:p.G138D
TP53:NM_001126112:exon8:c.834_846delinsCTTTCGC:p.278_282delinsPFR
KRAS:NM_033360:exon2:c.G35A:p.G12D

NPM1:NM_002520:exon11:c.859_860insTCTG:p.L287fs
GATA2:NM_032638:exon5:c.1096_1097insCCAACG:p.G366delinsANG
TET2:NM_001127208:exon3:c.1606_1607del:p.K536fs
TET2:NM_001127208:exon7:c.G3866A:p.C1289Y
NPM1:NM_002520:exon11:c.859_860insTCTG:p.L287fs
3:NM_004119:exon14:c.1792_1793insGCCTTGATTTCAGAGAATATG:p.E598delinsGLDFR
NPM1:NM_002520:exon11:c.859_860insTCTG:p.L287fs
NF1:NM_001042492:exon42:c.C6247T:p.R2083C
DNMT3A:NM_022552:exon15:c.1742_1743delinsCT:p.W581S
IDH1:NM_005896:exon4:c.C394T:p.R132C
KIT:NM_000222:exon17:c.T2377A:p.L793M
NO_MUTATION
FLT3:NM_004119:exon20:c.2508_2510del:p.836_837del
IDH1:NM_005896:exon4:c.C394T:p.R132C
DNMT3A:NM_022552:intron19:c.2323-1G>C
NPM1:NM_002520:exon11:c.859_860insTCTG:p.L287fs
FLT3:NM_004119:exon14:c.1770_196dup:p.591_599dup
RAD21:NM_006265:exon2:c.98_99insTCCCTTTT:p.F33fs
EZH2:NM_004456:exon6:c.C619T:p.R207X
EZH2:NM_004456:exon20:c.2229_2230insCCCGGC:p.I744delinsPGI
WT1:NM_024426:exon9:c.1371_1372insGGTTCCGTCGT:p.R458fs
FLT3:NM_004119:exon14:c.1783_1821dup:p.595_607dup
WT1:NM_024426:exon9:c.C1372T:p.R458X
WT1:NM_024426:intron7:c.1249+1G>A
NRAS:NM_002524:exon2:c.G38A:p.G13D
NPM1:NM_002520:exon11:c.859_860insTCTG:p.L287fs
TET2:NM_001127208:exon3:c.C1648T:p.R550X
NRAS:NM_002524:exon2:c.G37C:p.G13R
NRAS:NM_002524:exon3:c.A182G:p.Q61R
SF3B1:NM_012433:exon15:c.C2098G:p.Q700E
EP300:NM_001429:exon26:c.T4242G:p.Y1414X
BCORL1:NM_021946:exon3:c.C2512T:p.Q838X
STAG2:NM_001042749:exon25:c.C2464T:p.Q822X
DNMT3A:NM_022552:exon23:c.C2644A:p.R882S
FLT3:NM_004119:exon14:c.1799_1800insCC:p.D600fs
NRAS:NM_002524:exon3:c.A183T:p.Q61H
PTPN11:NM_002834:exon3:c.A227G:p.E76G
FLT3:NM_004119:exon20:c.G2503T:p.D835Y
IDH1:NM_005896:exon4:c.G395A:p.R132H
NPM1:NM_002520:exon11:c.861_862insTGTA:p.L287fs
NPM1:NM_002520:exon11:c.861_862insTGCA:p.L287fs
3:NM_004119:exon14:c.1793_1794insCGTTGATTTCAGAGAATATGA:p.E598delinsDVDFR
SF3B1:NM_012433:exon14:c.G1998T:p.K666N
TGGAATGTGCCAAATGTTTCTGCAGCATTTCTTTTCCATTGGAAAATCTTTAAAATGCACG
NPM1:NM_002520:exon11:c.860_863dup:p.W288fs
IDH2:NM_002168:exon4:c.G419A:p.R140Q
NRAS:NM_002524:exon2:c.G34A:p.G12S
WT1:NM_024426:exon7:c.C1105T:p.R369X
NPM1:NM_002520:exon11:c.859_860insTCTG:p.L287fs
DNMT3A:NM_175629:exon23:c.G2645A:p.R882H

RUNX1:NM_001001890:exon6:c.1270_1271insGAACCAGAGCG:p.D424fs
PTPN11:NM_002834:exon13:c.C1520A:p.T507K
STAG2:NM_001042749:exon31:c.3391_3392del:p.R1131fs
NPM1:NM_002520:exon11:c.859_860insTTTG:p.L287fs
FLT3:NM_004119:exon14:c.A1718G:p.E573G
KRAS:NM_033360:exon2:c.G38A:p.G13D
NRAS:NM_002524:exon2:c.G38A:p.G13D
PTPN11:NM_002834:exon13:c.T1504C:p.S502P
SMC3:NM_005445:exon11:c.G931T:p.D311Y
PTPN11:NM_002834:exon3:c.A227G:p.E76G
SF3B1:NM_012433:exon15:c.A2098G:p.K700E
TACTTCTACGTTGATTTCAGAGAATATGAATATGATCTCAAATGGGAGTTTCCAAGAGAA
DNMT3A:NM_175629:exon18:c.C2141G:p.S714C
SRSF2:NM_003016:exon1:c.C284T:p.P95L
BCORL1:NM_021946:exon6:c.3985_4010delinsAGACCAGGAAAA:p.K1328fs
FLT3:NM_004119:exon20:c.G2503T:p.D835Y
KRAS:NM_033360:exon2:c.G38A:p.G13D
PTPN11:NM_002834:exon3:c.G178C:p.G60R
NF1:NM_001042492:exon14:c.1561delA:p.S521fs
IDH1:NM_005896:exon4:c.G395A:p.R132H
DNMT3A:NM_175629:exon23:c.G2645A:p.R882H
NPM1:NM_002520:exon11:c.859_860insTCTG:p.L287fs
RAD21:NM_006265:exon5:c.419delT:p.V140fs
DNMT3A:NM_175630:exon2:c.C55T:p.R19W
NPM1:NM_002520:exon9:c.758_771del
TET2:NM_001127208:exon3:c.2780dupT:p.V927fs
TET2:NM_001127208:exon3:c.1837dupG:p.P612fs
DNMT3A:NM_175629:exon18:c.C2099T:p.P700L
004119:exon14:c.1817_1818insTGAATATGATCTCAAATGGGAGTTTCC:p.P606delinsPEY
NPM1:NM_002520:exon11:c.860_863dup:p.W288fs
TET2:NM_001127208:exon3:c.971dupA:p.Q324fs
IDH1:NM_005896:exon4:c.C394T:p.R132C
DNMT3A:NM_175629:exon23:c.G2678C:p.W893S
NPM1:NM_002520:exon11:c.860_863dup:p.W288fs
NO_MUTATION
U2AF1:NM_001025203:exon6:c.G467A:p.R156H
BRAF:NM_004333:exon3:c.G475A:p.V159I
NPM1:NM_002520:exon11:c.859_860insTCTG:p.L287fs
STAG2:NM_001042749:exon6:c.346_356delinsC:p.D116fs
PHF6:NM_032458:exon9:c.902dupA:p.Y301_H302delinsX
TET2:NM_001127208:exon6:c.G3782A:p.R1261H
GATA2:NM_032638:exon4:c.936_956dup:p.312_318dup□
CEBPA:NM_004364:exon1:c.C970G:p.L324V
CEBPA:NM_004364:exon1:c.939_940insAAG:p.V314delinsKV
FLT3:NM_004119:exon20:c.G2503T:p.D835Y
IDH1:NM_005896:exon4:c.G395A:p.R132H
NPM1:NM_002520:exon11:c.859_860insTCTG:p.L287fs
DNMT3A:NM_175629:exon23:c.C2644T:p.R882C
KRAS:NM_033360:exon2:c.G35A:p.G12D
NRAS:NM_002524:exon3:c.A182G:p.Q61R

CEBPA:NM_004364:exon1:c.68_78del:p.P23fs
TET2:NM_001127208:exon7:c.A3907G:p.S1303G
CEBPA:NM_004364:exon1:c.A914C:p.Q305P
CSF3R:NM_156039:exon15:c.C1919A:p.T640N
_004119:exon14:c.1805_1806insTTTCAGAGAATATGAATATGATCTCAA:p.K602delinsNFR
CSF3R:NM_156039:exon17:c.2427dupC:p.S810fs
SRSF2:NM_003016:exon1:c.C283A:p.P95T
IDH2:NM_002168:exon4:c.G419A:p.R140Q
NPM1:NM_002520:exon11:c.859_860insTCTG:p.L287fs
NRAS:NM_002524:exon2:c.G34A:p.G12S
NRAS:NM_002524:exon3:c.A182G:p.Q61R
WT1:NM_024426:exon9:c.C1393T:p.H465Y
FLT3:NM_004119:exon20:c.2508_2510del:p.836_837del
FLT3:NM_004119:exon16:c.A1988G:p.K663R
NPM1:NM_002520:exon11:c.860_863dup:p.W288fs
DNMT3A:NM_175629:exon7:c.C745T:p.Q249X
DNMT3A:NM_175629:exon22:c.A2525G:p.Q842R
IDH2:NM_002168:exon4:c.G419A:p.R140Q
SRSF2:NM_003016:exon1:c.C284A:p.P95H
ASXL1:NM_015338:exon12:c.1927dupG:p.G642fs
FLT3:NM_004119:exon14:c.1797_1798insCTTACCCAATATGAATAT:p.D600delinsLTQYEYD
NPM1:NM_002520:exon11:c.859_860insTCTG:p.L287fs
IDH2:NM_002168:exon4:c.G419A:p.R140Q
PTPN11:NM_002834:exon3:c.A227G:p.E76G
IDH1:NM_005896:exon4:c.G395A:p.R132H
DNMT3A:NM_175629:exon23:c.G2645A:p.R882H
NPM1:NM_002520:exon11:c.859_860insTCTG:p.L287fs
IDH1:NM_005896:exon4:c.C394T:p.R132C
NPM1:NM_002520:exon11:c.860_863dup:p.W288fs
WT1:NM_024426:exon7:c.1134delT:p.L378fs
PTPN11:NM_001330437:exon13:c.G1520A:p.G507E
NPM1:NM_002520:exon11:c.859_860insTCTG:p.L287fs
STAG2:NM_001042749:intron17:c.1639-2A>G
PHF6:NM_032458:exon9:c.903delC:p.Y301fs
3:NM_004119:exon14:c.1773_1774insTCAGATAATGAGTACTTCTAC:p.V592delinsSDNEY
WT1:NM_024426:exon8:c.A1330T:p.R444X
NPM1:NM_002520:exon11:c.860_863dup:p.W288fs
FLT3:NM_004119:exon16:c.C2028A:p.N676K
M_004119:exon14:c.1793_1794insCTACGTTGATTTCAGAGAATATGA:p.E598delinsDYVD
RAD21:NM_006265:exon12:c.1585_1586insGA:p.K529fs
EP300:NM_001429:exon31:c.A5372G:p.Y1791C
NPM1:NM_002520:exon11:c.859_860insTCTG:p.L287fs
DNMT3A:NM_175629:exon23:c.C2644T:p.R882C
821_1822insAGGCTCAGAGAATATGAATATGATCTCAAATGGGAGTTTCCAAGA:p.E608d
n14:c.1783_1784insAGGGCTCCTCAGATAATGAGTACTTCTACGTTGATTTCA:p.R595delin
DNMT3A:NM_175629:exon23:c.C2644T:p.R882C
BCOR:NM_017745:exon14:c.4834dupC:p.L1612fs
TP53:NM_001126112:exon8:c.C916T:p.R306X
NRAS:NM_002524:exon2:c.G35A:p.G12D
FLT3:NM_004119:exon20:c.A2522T:p.N841I

FLT3:NM_004119:exon21:c.T2600G:p.I867S
NPM1:NM_002520:exon11:c.859_860insTCTG:p.L287fs
SRSF2:NM_003016:exon1:c.C284G:p.P95R
IDH2:NM_002168:exon4:c.G419A:p.R140Q
RUNX1:NM_001001890:exon6:c.1075_1076insTACCTGCCGCCGCCCTACC:p.P359fs
EZH2:NM_004456:exon17:c.G1978A:p.G660R
SF3B1:NM_012433:exon15:c.A2098G:p.K700E
DNMT3A:NM_022552:exon23:c.C2644T:p.R882C
CBL:NM_005188:exon8:c.1134_1135delinsAA:p.378_379delinsLK
CBL:NM_005188:exon9:c.G1243A:p.G415S
KRAS:NM_033360:exon2:c.C53A:p.A18D
NPM1:NM_002520:exon11:c.862_863insGCAG:p.W288fs
ASXL1:NM_015338:exon12:c.1927dupG:p.G642fs
TET2:NM_001127208:intron4:c.3500+1G>A
SRSF2:NM_003016:exon1:c.C284G:p.P95R
TET2:NM_001127208:exon10:c.4452delC:p.N1484fs
CBL:NM_005188:exon8:c.T1139C:p.L380P
EZH2:NM_004456:exon18:c.G2069A:p.R690H
PTPN11:NM_002834:exon3:c.G179T:p.G60V
NRAS:NM_002524:exon2:c.G35C:p.G12A
NPM1:NM_002520:exon11:c.859_860insTCTG:p.L287fs
DNMT3A:NM_022552:exon10:c.1160_1161insTG:p.C387fs
LT3:NM_004119:exon14:c.1807_1808insATGAATATGATCTCAAAT:p.W603delinsYEYDLK
SMC3:NM_005445:exon11:c.896_897insTAA:p.I299delinsIN
DNMT3A:NM_022552:exon23:c.G2645A:p.R882H
NPM1:NM_002520:exon11:c.859_860insTCTG:p.L287fs
NPM1:NM_002520:exon11:c.859_860insTCTG:p.L287fs
IDH2:NM_002168:exon4:c.G419A:p.R140Q
LT3:NM_004119:exon14:c.1807_1808insATGAATATGATCTCAAAT:p.W603delinsYEYDLK
NPM1:NM_002520:exon11:c.859_860insTCTG:p.L287fs
SMC3:NM_005445:exon11:c.896_897insTAA:p.I299delinsIN
DNMT3A:NM_022552:exon23:c.G2645A:p.R882H
CEBPA:NM_004364:exon1:c.717_718insCGCGCCC:p.A240fs
CEBPA:NM_004364:exon1:c.939_940insAAG:p.V314delinsKV
WT1:NM_024426:exon7:c.1170_1171insCTGCCCC:p.F391fs
WT1:NM_024426:exon7:c.1109_1110insC:p.R370fs
WT1:NM_024426:exon7:c.1195_1196insCACTGCA:p.N399fs
NRAS:NM_002524:exon3:c.A182G:p.Q61R
RAD21:NM_006265:exon8:c.867_872del:p.289_291del
CUX1:NM_001913:exon11:c.A1049G:p.K350R
NRAS:NM_002524:exon2:c.G34A:p.G12S
DNTM3A:NM_022552:exon23:c.G2645A:p.R882H
KIT:NM_000222:exon9:c.A1526T:p.K509I
GATA2:NM_032638:exon5:c.G1085A:p.R362Q
NPM1:NM_002520:exon11:c.859_860insTCTG:p.L287fs
DNMT3A:NM_175629:exon23:c.G2645A:p.R882H
AGATAATGAGTACTTCTACGTTGATTTCAGAGAATATGAATATGATCTCAAATGGGAGTT
NPM1:NM_002520:exon11:c.859_860insTCTG:p.L287fs

95_1796insGCTCCTCAGATAATGAGTACTTCTACGTTGATTTCAGAGAATATGAAT:p.Y599
GTACTTCTACGTTGATTTCAGAGAATATGAATATGATCTCAAATGGGAGTTTCCAAGAGAA
NM_024426:exon7:c.1143_1144insTACGGTCG:p.A382fs
FLT3:NM_004119:exon20:c.G2503T:p.D835Y
NPM1:NM_002520:exon11:c.860_863dup:p.W288fs
TET2:NM_001127208:exon6:c.G3785A:p.R1262Q
T2:NM_001127208:exon3:c.2069delA:p.Q690fs,TET2:NM_017628:exon3:c.2069delA:p.Q69
NMT3A:NM_153759:exon19:c.G2078A:p.R693H,DNMT3A:NM_022552:exon23:c.G2645A:p.F
91_1792insACCGGCTCCTCAGATAATGAGTACTTCTACGTTGATTTCAGAGAATAT:p.E598
_199185:exon10:c.772_773insTCTG:p.L258fs,NPM1:NM_002520:exon11:c.859_860insTCT(

VAF
4.01146
37.963
44.7989
44.9198
48.2759
4.24628
4.4586
4.73118
29.7333
38.7952
43.6594
42.3656
46.0459
48.6423
94.1019
41.8573
44.6367
44.6398
45.9821
46.6368
48.5584
24.1379
40.1042
42.3469
4.42177
15.6485
41.7513
38.2253
38.5475
40.2597
33.494
43.7282
7.17629
29.5608
32.6172
37.4257
6.77656
33.4016
44.27
45.0751
38.4314
42.7653
46.2185
48.0287
43.9
5
4.2

35.5491
43.6782
44.1319
45.5263
47.4227
20.5882
45.0704
45.5432
47
47.4783
49.6997
NA
2.52874
45.0397
46.4471
46.6981
NA
2.40964
3.90764
5.04386
13.3562
25
2.11726
22.043
23.9157
29.6296
63.6268
4.8474
37.7049
40.048
44.8161
84.6473
85.0622
48.6081
NA
43.1579
8.42857
30.2264
41.9134
46.114
33.7079
38
46.2209
22.4771
43.9394
46.5228
16.3265
41.5254
41.2698
48.324

34.7826
39.3417
41.9802
44.2029
48.6111
2.40964
2.88221
4.19162
5.9322
20.5607
43.2247
13.1868
46.8571
50
10
2.38636
9.40767
13.2836
23.0769
26.7068
43.4307
43.4944
44.9102
60
24
42.3423
43.6992
51.1364
9.45559
47.0199
92.8889
46.5753
48.9362
59.0361
NA
22.5191
36.7347
57.265
82
83.9695
14.0777
39
40.5
44.7205
10.3352
17.1233
41.5789
53.6388
7.10526
24.1379

40.3315
45.5587
46.2277
46.2633
3.61111
6.69856
46.6357
47.5709
48
2.01342
34.0659
9.25926
14.0496
25.8242
31.746
47.2527
3.15457
45.488
47.0588
49.1124
3.59589
44.6064
49.3506
33.8832
35.1082
39.071
43.9359
32.8947
33.8028
9.25926
10.5556
34.3066
75
13.3333
26.1905
36.5079
46.4789
57.9439
3.76884
43.7086
46.4164
47.1042
47.8088
30.0885
37.2093
43.75
88.7755
82.7119
3.58974
5.83153

18.4783
42.9658
47.5362
48.0851
16.4912
44.7423
51.3465
52.809
81
4.35897
6.86499
16.6667
39.6396
43.2494
45.5621
47.2477
80.5611
16.1426
16.3814
20.6823
43.9863
44.0129
14.8377
39.7541
46.2085
47.4048
43.0556
46.1219
39.2226
40.9836
41.8231
46.6667
33.333
13.0802
4.55635
16.338
36.4943
43.75
37.4408
52.6957
44.247
46.2704
2.28401
48.3333
46.9091
41.261
7.78626
43.4442

2.03095
8.0315
37.8534
8.79332
49.6552
46.1667
48.1918
42.8904
19.8895
41.9048

Drug	Provider	Catalog #	Acronym	Solvent
Azacitidine	Selleckchem	S1782	AZA	DMSO
Crenolanib	Selleckchem	S2730	CRENO	DMSO
Enasidenib	Selleckchem	S8205	AG221	DMSO
Venetoclax	MedChem Express	HY-15531	VEN	DMSO
MI-2	Selleckchem	S7618	MI-2	DMSO
Midostaurin	MedChem Express	HY-10230	MIDO	DMSO
Actinomycin D	MedChem Express	HY-17559	ActinoD	DMSO
Cytarabine	MedChem Express	HY-13605	AraC	DMSO
Daunorubicin	MedChem Express	HY-13062	DNR	DMSO
EPZ5676	Selleckchem	S7062	EPZ	DMSO
ATRA	SIGMA	R2625	ATRA	DMSO
Panobinostat	Selleckchem	S1030	PANO	DMSO
OTX015	MedChem Express	HY-15743	OTX015	DMSO
Arsenic Trioxyde	Sigma	72718	ATO	HCl 2%
Selinexor	Interchim	AG001ASN	SEL	DMSO
Idasanutlin	MedChem Express	HY-15676	IDASA	DMSO
Dactolisib	MedChemExpress	HY-50673	DACTO	DMSO
Uprosertib	MedChemExpress	HY-15965	UPRO	DMSO
Olaparib	MedChem Express	HY-10162	OLA	DMSO
Palbociclib	MedChemExpress	HY-50767	PALBO	H_2O
Ulixertinib	MedChemExpress	HY-15816	ULIX	DMSO
Ruxolitinib	MedChemExpress	HY-50856	RUXO	DMSO
Dabrafenib	MedChemExpress	HY-14660	DABRA	DMSO
Gilteritinib	MedChem Express	HY-12432	GILTE	DMSO
S63845	MedChem Express	HY-100741	S63845	DMSO
Glasdegib	MedChem Express	HY-16391	GLASDE	DMSO
Ivosidenib	MedChem Express	HY-18767	IVO	DMSO

NR: not reached

*arbitrary choice as PK data was not available and IC50 not reached in healthy CD34+ cells.

Stock Concentration (mM)	Pharmacokinetics Data		Healthy CD34	
	Cmax (µM)	Reference (PMID or DOI)	Top tested concentration (µM)	IC_{50} (µM)
100	3.1	15831784	3	NR
100	0.48	10.1182/blood.V126	0.5	NR
100	27.5	29320949	30	NR
100	2.1	28100580	3	0.31
100	N/A	N/A	10	NR
100	3.8	15345597	4	NR
100	0.16	24968986	0.2	0.041
100	55.13	23919448	10	2.73
100	0.19	27738808	4	0.19
100	N/A	N/A	10	NR
100	8	10388008	8	NR
100	0.29	25939707	0.3	0.015
20	0.274	26341814	1	0.2
5	4.56	28462082	5	0.66
100	2	28336527	2	NR
10	0.24	23808545	3	NR
20	1.7	28357727	1	NR
80	0.7	23795919	1	NR
100	0.06	30324586	1	NR
25	0.1	27402157	1	NR
100	2.79	29470718	10	NR
100	1.29	29162613	10	NR
50	7.56	27058232	10	NR
10	0.51	FDA	5	NR
10	N/A	N/A	10	0.029
10	3.03	29463550	30	1.038
100	1.69	30758648	20	NR

+ assay		Drug Response Pattern (Figure 2) Concentration (μM)	Niche Component Screen (Figure 3) Concentration (μM)	DNR-AraC S
IC_{40} (μM)	IC_{10} (μM)			Concentration (μM)
NR	NR	3	50	-
NR	NR	0.5	-	0.1
NR	NR	30	-	6
0.2	0.034	0.2	0.5	0.04
NR	NR	10	-	2
NR	NR	4	20	0.8
0.027	0.004	0.03	-	0.004
1.8	0.3	2	20	40
0.12	0.021	0.1	2	2
NR	NR	10	-	2
NR	NR	8	10	1.6
0.01	0.002	0.01	0.02	0.002
0.13	0.02	0.15	-	0.02
0.44	0.073	0.4	25	-
NR	NR	-	2	0.4
NR	NR	-	-	0.048
NR	NR	-	-	0.34
NR	NR	-	-	0.14
NR	NR	-	-	0.012
NR	NR	-	-	0.02
NR	NR	-	-	0.56
NR	NR	-	-	0.26
NR	NR	-	-	1.5
NR	NR	-	-	0.1
0.019	0.0034	-	-	0.0034
0.019	0.01	-	-	0.01
NR	NR	-	-	0.34

creen (Figure 5)
**Justification**
-
20% of Cmax
20% of Cmax
IC10
arbitrary*
20% of Cmax
IC10
arbitrary
20:1 AraC/DNR ratio
arbitrary*
20% of Cmax
IC10
IC10
-
20% of Cmax
20% of Cmax
20% of Cmax
20% of Cmax
20% of Cmax
20% of Cmax
20% of Cmax
20% of Cmax
20% of Cmax
20% of Cmax
IC10
IC10
20% of Cmax

Supplementary Table 5. Amino acid and hexose concentrations (μM) in patients bone m

UPN	Sample	Ala	Arg	Asn	Asp
SLS009	PLASMA	370	98.7	60	29.9
SLS014	PLASMA	459	85	70.4	4.63
SLS019	PLASMA	180	20	27.4	5.1
SLS020	PLASMA	425	77.2	56.7	22.1
SLS025	PLASMA	384	32.7	32.8	46.1
SLS032	PLASMA	292	62.2	33.1	13.3
SLS033	PLASMA	291	60.1	46.2	11
SLS036	PLASMA	76.6	13.5	11.8	10.2
SLS037	PLASMA	229	39.7	32.5	3.15
SLS043	PLASMA	428	62.1	53.8	117
SLS048	PLASMA	139	37.6	28.6	7.61
SLS050	PLASMA	434	139	55.2	75.2
SLS068	PLASMA	298	14.4	26.7	51.5
SLS070	PLASMA	172	78.8	37.6	5.33
SLS083	PLASMA	428	25.4	30.2	12.9
SLS085	PLASMA	287	95.8	41.1	25.2
SLS094	PLASMA	244	27.7	44.1	51.6
SLS095	PLASMA	551	46.9	69.6	47.8
SLS107	PLASMA	157	82.8	29.8	3.03
SLS110	PLASMA	274	34.3	31.9	6.7
SLS134	PLASMA	213	5.55	29	4.41
SLS149	PLASMA	218	24.6	22.9	16.5
SLS150	PLASMA	594	78.4	53.5	29.3
SLS158	PLASMA	563	18.3	48.1	47.9
SLS041	MSC w/AML CM	365	390	192	177
SLS043	MSC w/AML CM	375	363	209	213
SLS050	MSC w/AML CM	461	411	217	203
SLS058	MSC w/AML CM	456	457	210	213
SLS085	MSC w/AML CM	371	473	205	181
N/A	MSC w/o AML CM (ref.)	381	350	211	217

ıarrow plasma samples, MSC with or without AML co-culture

Cit	Gln	Glu	Gly	His	Ile	Leu
38.4	601	213	433	80.9	87	184
27.4	743	19.7	440	103	90	181
9.23	352	22.1	277	38.5	30.5	74.2
20.9	736	208	292	99	94.1	182
20.2	457	347	349	73.4	60.4	140
34.4	500	160	263	58.1	51.9	115
13.9	452	124	253	73.3	108	229
16.8	247	88.8	212	38.7	26.1	66.7
11.5	388	13.2	125	50.1	74.5	155
21	569	468	439	93.3	70.4	198
11.1	394	62.2	331	54.2	51.5	104
21.9	756	361	506	86.5	103	242
25.4	440	410	358	58.4	27.9	66.8
16.3	569	49	290	71.3	71.2	153
10.8	429	103	318	65.3	58.5	103
27.7	523	89.9	364	51	89.4	167
19.6	297	284	324	59	90	212
23.2	601	172	480	91.7	50.3	140
16.4	470	57	202	61	74.3	189
14.6	470	44.1	181	47.9	64.9	115
22.2	435	72.5	166	53.3	30	64.7
15	376	141	455	62	26.7	80.7
26.6	694	125	399	76.4	68.8	154
29	267	509	465	87.9	128	229
21.8	1580	609	618	138	285	347
23.3	1400	624	610	140	320	388
26.4	1410	609	705	158	347	443
26.3	1470	583	737	162	341	418
22.7	1570	657	689	153	348	418
23.4	1340	568	603	141	319	397

Lys	Met	Orn	Phe	Pro	Ser	Thr
275	22.8	144	73.6	245	133	137
295	32.1	126	74.3	310	127	168
117	12.4	84.8	67.7	152	60.2	44.2
236	30	95	77.2	278	133	139
218	20.9	135	78	245	112	121
169	12.4	98.8	53.7	176	59.6	109
215	36.7	129	142	109	59.5	85.2
69.4	6.52	70.7	51.6	93.3	22.8	51.9
132	20.3	147	152	161	65.6	59.7
340	29.2	197	134	265	128	106
149	10.5	48.1	32.4	110	78.4	57.2
332	29.6	176	114	241	167	139
140	8.39	88.3	72.3	131	13.7	74.3
187	18.9	80.2	60.4	150	88.1	87.5
163	13	88	57.4	185	33.6	84.1
322	30.1	113	62.9	184	77	113
219	22.9	109	73.1	161	120	95.9
193	27.7	128	86.3	510	177	150
151	15.2	84.5	91.2	138	119	78.5
112	10.2	102	98.3	164	63.5	53.9
109	9.68	99.7	68	119	29	46.9
113	7.73	69.3	45	130	26.4	53.7
224	17.5	123	97.7	334	79	107
291	32.8	109	92.9	351	106	124
331	60.2	50.3	149	281	203	294
348	63.5	57.6	153	279	217	319
378	73.8	85.9	182	341	255	329
353	74.1	84.9	182	351	240	318
347	68.6	54.6	166	313	229	311
367	66.1	54.6	152	296	228	325

Trp	Tyr	Val	Hexoses
67.5	84.1	264	5887
69.1	83.6	261	5423
46.1	64	130	6599
73.5	78.2	272	5818
56.6	78.5	233	4280
29.1	47.7	209	6062
61.6	89.6	282	3022
47.6	36.6	138	7332
43.3	70.3	269	8144
65	63.2	236	5254
48	30.3	176	5744
55.5	72.5	349	11364
32.6	46.6	135	3636
33.9	50.6	206	5495
48.3	50.1	221	3613
46.3	66.2	261	6365
27.5	62.3	255	3096
54.5	63.4	219	6257
44.7	51.2	275	6293
43.9	44.9	186	6786
52	49.1	133	5933
39.2	33	131	2571
52.1	66.5	265	7553
53.6	86.4	281	667
41	142	329	3562
38.7	148	343	5092
49.9	169	386	6417
53.4	168	400	6624
47.5	162	364	4592
45	148	346	5619

Supplementary Table 6. Cytokine concentrations (pg/mL) in patients bone marrow pla

UPN	Sample	SCF	TPO	M-CSF	IL-6
SLS009	PLASMA	166.67	1783.69	15.69	14.41
SLS014	PLASMA	213.90	645.51	6.71	0.36
SLS019	PLASMA	133.60	2146.91	4.16	17.44
SLS020	PLASMA	130.75	334.86	10.97	0.08
SLS032	PLASMA	155.20	428.57	12.75	1.98
SLS033	PLASMA	107.84	4345.05	26.08	10.66
SLS036	PLASMA	112.01	1034.08	6.57	2.79
SLS037	PLASMA	130.75	2553.45	24.20	58.73
SLS048	PLASMA	126.63	524.13	2.74	0.45
SLS050	PLASMA	134.13	1007.57	15.05	0.27
SLS070	PLASMA	139.29	710.62	4.75	1.35
SLS083	PLASMA	145.50	959.54	4.92	1.39
SLS085	PLASMA	165.13	964.15	6.08	1.68
SLS094	PLASMA	153.62	266.74	18.92	31.77
SLS095	PLASMA	154.36	1091.52	10.45	2.08
SLS107	PLASMA	136.02	2330.95	15.59	4.36
SLS110	PLASMA	153.05	641.32	29.71	19.55
SLS134	PLASMA	132.19	326.31	7.51	5.64
SLS149	PLASMA	111.40	1145.63	5.33	4.67
SLS150	PLASMA	151.22	274.32	8.28	2.11
SLS158	PLASMA	92.08	178.12	16.85	641.73
SLS041	MSC w/AML CM	371.52	33.02	4.34	4954.19
SLS043	MSC w/AML CM	547.41	86.44	22.98	4877.97
SLS050	MSC w/AML CM	336.94	16.51	3.69	424.66
SLS058	MSC w/AML CM	321.79	16.51	1.52	335.24
SLS085	MSC w/AML CM	335.98	158.68	15.61	5120.21
N/A	MSC w/o AML CM (ref.)	638.89	16.51	2.40	213.33

sma samples, MSC with or without AML co-culture

IL-3	GM-CSF	G-CSF	FLT3-L	EPO
10.74	1.96	26.12	11.53	3102.79
9.02	0.06	14.57	56.83	448.83
0.53	0.56	46.26	2.54	5295.89
0.53	0.05	3.62	40.92	894.58
0.53	0.16	40.58	6.02	1666.10
0.53	0.11	81.97	1.73	285.71
8.11	0.25	6.41	2.20	3832.85
0.53	0.71	2751.97	344.55	4201.56
0.53	0.14	8.18	14.21	1603.22
4.62	0.01	19.26	4.71	473.80
29.96	0.47	14.41	1.70	9474.90
6.12	0.21	20.39	2.12	1480.91
10.18	0.11	14.62	118.73	101.77
0.53	0.11	17.92	49.96	336.82
18.44	0.30	6.73	95.98	144.15
0.53	0.06	22.92	3.75	2641.16
9.61	1.07	163.23	2.74	19817.46
0.53	0.01	17.97	7.84	280.62
0.53	0.08	1.30	3.31	633.83
5.01	0.15	18.12	5.20	2788.55
0.53	0.02	15.93	2.58	385.63
0.54	4.83	224.69	1279.48	31.84
0.54	102.80	584.14	1415.06	36.63
0.54	8.56	1.30	914.46	22.20
0.54	5.09	1.30	323.99	21.13
18.66	1195.44	9458.90	1556.85	37.73
0.54	9.69	1.30	1749.51	34.36

Supplementary Table 7. Comparison of reference and plasma-like medium	MW	MEMα (Cat #22571)	
		mg/L	mM
**Anorganic salts**			
Calcium Chloride (CaCl2-2H2O)	147	264	1.796
Magnesium Sulfate (MgSO4) (anhyd.)	120	97.67	0.814
Potassium Chloride (KCl)	75	400	5.333
Sodium Bicarbonate (NaHCO3)	84	2200	26.190
Sodium Chloride (NaCl)	58	6800	117.241
Sodium Phosphate monobasic (NaH2PO4-H2O)	138	140	1.014
**Other components**			
D(+)-Glucose anhydrous	180	1000	5.556
Lipoic acid	206	0.2	0.001
Phenol Red	376.4	10	0.027
Sodium Pyruvate	110	110	1.000
**Vitamines**			
Ascorbic Acid	176	50	0.284
Biotin	244	0.1	0.000
Choline chloride	140	1	0.007
D-Calcium pantothenate	477	1	0.002
Folic Acid	441	1	0.002
myo-Inositol	180	2	0.011
Niacinamide	122	1	0.008
Pyridoxal hydrochloride	204	1	0.005
Riboflavin	376	0.1	0.000
Thiamine hydrochloride	337	1	0.003
Vitamin B12	1355	1.36	0.001
**Ribonucleosids**			
Adenosine	267	10	0.037
Cytidine	243	10	0.041
Guanosine	283	10	0.035
Uridine	244	10	0.041
**Deoxy-ribonucleosids**			
2'-Deoxyadenosine x H2O	251	10	0.040
2'-Deoxycytidine x HCl	264	11	0.042
2'-Deoxyguanosine	267	10	0.037
2'-Deoxythymidine	242	10	0.041
**Amino acids**			
Glycine	75	50	0.667
L-Alanine	89	25	0.281
L-Arginine hydrochloride	NA	0	0.000
L-Arginine	211	105	0.498
L-Asparagine-H2O	150	25	0.167
L-Aspartic acid	133	30	0.226
L-Cysteine hydrochloride-H2O	176	100	0.568
L-Cystine	313	31	0.099
L-Glutamic Acid	147	75	0.510
L-Glutamine	146	292	2.000
L-Histidine hydrochloride-H2O			
L-Histidine	155	31	0.200

L-Isoleucine	131	52.4	0.400
L-Leucine	131	52	0.397
L-Lysine hydrochloride			
L-Lysine	183	73	0.399
L-Methionine	149	15	0.101
L-Phenylalanine	165	32	0.194
L-Proline	115	40	0.348
L-Serine	105	25	0.238
L-Threonine	119	48	0.403
L-Tryptophan	204	10	0.049
L-Tyrosine	181	36	0.199
L-Valine	117	46	0.393
**Dialyzed FBS**		25%	
**Cytokines**		ng/ml	
TPO		0	
EPO		0	

Plasma-like Medium	
mg/L	mM
264.92	1.802
97.67	0.814
400	5.333
2200	26.188
6300	107.188
140	1.015
1000	5.556
0.2	0.001
10	0.027
110	1.000
50	0.284
0.1	0.000
1	0.007
1	0.002
1	0.002
2	0.011
1	0.008
1	0.005
0.1	0.000
1	0.003
1.33	0.001
10	0.038
10	0.041
10	0.035
10	0.041
10	0.040
11	0.042
10	0.038
10	0.041
32	0.430
22	0.250
0	0.000
35	0.160
8	0.050
8	0.060
100	0.570
31	0.100
33	0.220
144	0.990
0	0.000
18	0.120

19	0.140
25	0.190
0	0.000
45	0.240
6	0.040
20	0.120
30	0.260
12	0.120
20	0.170
11	0.050
19	0.100
33	0.280
25%	
ng/ml	
1	
2.5	

Supplementary Table 8. Variants and primers for targeted sequencing of primary samples a

UPN	Target	VariantCaller	Chr	Pos	REF
SLS356	BCOR_p.L1612fs	Varscan	chrX	40053925	A
SLS356	DNMT3A_p.R882C	Varscan	chr2	25234374	G
SLS356	FLT3_p.E608ITD	Pindel	chr13	28034097	C
SLS352	ASXL1_P.V751fs	Varscan	chr20	32434965	T
SLS352	CEBPA_p.P197delinsHPP	Varscan	chr19	33301825	G
SLS352	IDH2_p.R140Q	Varscan	chr15	90088702	C
SLS352	SRSF2_p.P95H	Varscan	chr17	76736877	G
SLS353	NPM1_p.A253fs	Pindel	chr5	171405390	ATAGAAAAA
SLS353	TET2_p.P612fs	Varscan	chr4	105235778	T
SLS353	TET2_p.V927fs	Varscan	chr4	105236721	G
SLS158	ASXL1_p.G642fs	Varscan	chr20	32434638	A
SLS158	DNMT3A_p.Q842R	Varscan	chr2	25235779	T
SLS158	IDH2_p.R140Q	Varscan	chr15	90088702	C
SLS158	SRSF2_p.95H	Varscan	chr17	76736877	G
SLS325	DNMT3A_p.V296G	Varscan	chr2	25247718	A
SLS325	NRAS_p.G12D	Varscan	chr1	114716126	C
SLS325	SRSF2_p.P95H	Varscan	chr17	76736877	G
SLS325	TET2_p.H1219R	Varscan	chr4	105243631	A
SLS351	PTPN11_p.T507K	Varscan	chr12	112489096	C
SLS351	RUNX1_p.D424fs	Varscan	chr21	34792226	T
SLS355	CBL_p.378_379delinsLK	Varscan	chr11	119278204	CC
SLS355	DNMT3A_pR882C	Varscan	chr2	25234374	G
SLS355	EZH2_p.G660R	Varscan	chr7	148810384	C
SLS355	RUNX1_p.P359fs	Varscan	chr21	34792421	G
SLS355	SF3B1_p.K700E	Varscan	chr2	197402110	T

nd residual cells after standard or niche-like culture

ALT	Forward Primer	Reverse Primer
AG	AGGACCAGAAGACCAGGATGA	GCATTGTGTGAGGTTCGTGG
A	CCTGCTGTGTGGTTAGACGG	CGCAAAATACTCCTTCAGCGG
GAGATCAT[illegible]	AAAGCCAGCTACAGATGGTACA	TCCATAAGCTGTTGCGTTCA
TG	ACAGAGGGCTACAGTTGGACT	GTGGTGCCAGACTCACATTCA
GGCGGGT	CGCTGGTGATCAAGCAGGA	TTGTCCACCGACTTCTTGGC
T	GCTGCAGTGGGACCACTATT	CAAGAGGATGGCTAGGCGAG
T	TTCGCCTTCGTTCGCTTTCA	CCCCTCAGCCCCGTTTACC
CGT	TCAGGGACAAGAATCCTTCAAGA	CAGTGCACTACGTTCCTTGC
TG	CTCGTTTTCACCAAGCGGAAT	TTGGGACTGCCCTTGATTCAT
GT	GCAAGATCTTCTTCACAGGTGC	GTGTGACTTCCTCCCTGGTC
AG	CGAGAGGTCACCACTGCCATA	AGGATAAGGCGGCAGTAGTTG
C	TGGTAGACGCATGACCAGTGT	GCACAGCAATCAGAACAGCC
T	GCTGCAGTGGGACCACTATT	CAAGAGGATGGCTAGGCGAG
T	TTCGCCTTCGTTCGCTTTCA	CCCCTCAGCCCCGTTTACC
C	TCGTCTCCCCGATTTTGCT	TGAGAATTTGCCGTCTCCGAA
T	AAGTACTGTAGATGTGGCTCGC	TCCGACAAGTGAGAGACAGGAT
T	TTCGCCTTCGTTCGCTTTCA	CCCCTCAGCCCCGTTTACC
G	ATGGAATGGTGATCCACGCAG	AAGTGCACGCTGAACTCTCTT
A	TTCGTAGGTGTTGACTGCGA	CCTGCTCAAAAGGAGAGCGT
GCTCTGGT	GGCTCCTACCAGTTCTCCATG	CTCAGTAGGGCCTCCACAC
AA	TGGAGCTTAAAATAGGACCCAGA[illegible]	GCTGTTTAGATCCGTACCTGC
A	CCTGCTGTGTGGTTAGACGG	CGCAAAATACTCCTTCAGCGG
T	TCACTCTGTGCGCTTTTGTG	TCTGGTCACCTCACTGACCT
GGCGGCGG[illegible]	CCCCCGCATGCACTATCC	CCCACCATGGAGAACTGGTA
C	GCTGACAGGCTATGGTTCATGT	GTTGGCGGATACCCTTCCATA

PRIMARY_VAF	STANDARD_CULTURE_VA	ICHE-LIKE_CULTURE_VAF
0.93	0.89	0.38
0.49	0.44	0.22
0.19	0.09	0.07
0.43	0.39	0.23
0.36	0.03	0.30
0.45	0.39	0.21
0.42	0.39	0.22
0.02	0.03	0.03
0.40	0.33	0.28
0.38	0.27	0.27
0.30	0.20	0.21
0.07	0.06	0.06
0.49	0.29	0.34
0.47	0.32	0.32
0.26	0.39	0.34
0.45	0.40	0.30
0.49	0.41	0.33
0.44	0.41	0.31
0.35	0.34	0.34
0.28	0.27	0.26
0.83	0.88	0.72
0.48	0.46	0.38
0.34	0.38	0.34
0.12	0.11	0.08
0.48	0.45	0.37

Supplementary Table 9. Gene signatures for leukemic states.

VANGALEN_GMP_LIKE	VANGALEN_HSC_LIKE	VANGALEN_PROGENITOR_LIKE	VANGALEN_
PRTN3	*NPTX2*	*CDK6*	*DEFB1*
MPO	*H1F0*	*HSP90AB1*	*RNASE2*
CALR	*EMP1*	*SPINK2*	*MS4A3*
CLEC5A	*MEIS1*	*EEF1B2*	*SERPINB10*
ELANE	*CALCRL*	*PCNP*	*SESN3*
POU4F1	*TPSD1*	*TAPT1-AS1*	*ZFR*
TRH	*TPT1*	*HINT1*	*MRPL33*
TSPOAP1	*CRHBP*	*LRRC75A-AS1*	*CTSG*
CEBPE	*CLNK*	*DSE*	*SLC44A1*
LINC01835	*TSC22D1*	*PEBP1*	*SLPI*
NUCB2	*DST*	*LOC107984974*	*FUT4*
CSF3R	*NRIP1*	*H2AFY*	*SRGN*
RUNX1T1	*ABCB1*	*EEF1A1*	*CD70*
CD38	*GABRA4*	*SMIM24*	*PRLR*
PLPPR3	*ZBTB20*	*PSME1*	*PLD3*
IGFBP2	*ABCA9*	*SOX4*	*LPL*
PRRT4	*TPSB2*	*LINC01623*	*RETN*
SNHG5	*KMT2A*	*EEF1G*	*TP53INP2*
FABP5	*FAM30A*	*EBPL*	*HSPA5*
LOC100419170	*MEF2C*	*EIF4B*	*RNASE3*
CLEC11A	*TMEM74*	*PARP1*	*CCL23*
SERPINB1	*PDZRN4*	*MEST*	*EMB*
AZU1	*ST3GAL1*	*TMEM70*	*ATP8B4*
FBN2	*XIRP2*	*TFDP2*	*CLU*
HNRNPDL	*RBPMS*	*ATP5G2*	*FAM107B*
HSPB1	*TMEM25*	*NAP1L1*	*KBTBD11*
RNA5-8S	*C20orf203*	*MSI2*	*CSTA*
THSD7A	*GNG11*	*TPM4*	*ANKRD28*
C12orf57	*SLC6A13*	*SPN*	*PIWIL4*
FGFR1	*HOPX*	*SELL*	*RNVU1-6*
LPO	*CMTM2*	*RACK1*	*F13A1*
MGST1	*HIST1H2BK*	*CTSC*	*PROK2*
C1QTNF4	*NPDC1*	*PRDX6*	*IL31RA*
HMGN1	*SCRN1*	*EGFL7*	*PLAC8*
SIPA1L2	*MECOM*	*SNHG6*	*ELF1*
DDOST	*CXCL2*	*NOP53*	*EP400NL*
PTGIR	*CCS*	*MRPL57*	*EAF2*
GATM	*FAM74A4*	*SELENOP*	*RFX8*

VAMP8	*UMODL1-AS1*	*CCDC152*	*PTGFR*
FAM46A	*CCDC18-AS1*	*SRSF6*	*JUN*
VAMP5	*TTLL10*	*SNHG8*	*FRMD3*
STAR	*CFAP61*	*B4GALT6*	*HOXB-AS3*
ANKRD18A	*PLCB4*	*HNRNPA1*	*PHACTR3*
TM7SF3	*SPON1*	*KIF2A*	*AK2*
CCND1	*LINC01770*	*HSH2D*	*ECRP*
ROBO1	*CACNB4*	*PTPRCAP*	*DDIT3*
GFI1	*CCDC144NL-AS1*	*NACA2*	*RFLNB*
DEFA4	*RNF217*	*PDLIM1*	*FEZ1*
CERS6	*ZPBP*	*CPA3*	*SERPINB2*
PTMA	*MPPED2*	*GNPTAB*	*CDKN2A*

_PROMONOCYTE_LIKE	VANGALEN_MONOCYTE_LIKE	VANGALEN_CDC_LIKE
FCN1	MRC1	
S100A12	HLA-DRB5	
MAFB	CST3	
VCAN	SAMHD1	
S100A9	NAPSB	
PLBD1	FCER1A	
SERPINA1	HLA-DRB1	
BCL2A1	JAML	
THBS1	PKIB	
PSAP	HLA-DRA	
S100A8	HLA-DRB6	
FPR1	CPVL	
C5AR1	HLA-DPB1	
CD14	HLA-DQA1	
NAMPT	HLA-DPA1	
VNN2	CLEC4A	
CTSS	TMSB10	
DUSP1	CAP1	
CEBPB	HLA-DQB1	
CR1	CRIP1	
NFKBIA	CLEC10A	
SLC11A1	GPX1	
LILRB3	ITGB7	
BCL6	HLA-DQB2	
CYP1B1	DBI	
TNFAIP2	FTH1P3	
MS4A10	ACTB	
AQP9	HLA-DQA2	
TLR4	S100B	
APOBEC3A	ALDH2	
LRRK2	GABARAP	
CD163	LY86	
LRP1	IGSF6	
S100A10	HLA-DPB2	
CD68	CD74	
MEGF9	PAK1	
NCF2	LGALS2	
LOC643802	CSF1R	

IFNGR1	*PALLD*
FTL	*CCR2*
SLC24A4	*GPR183*
CD300E	*GRB2*
CXCL8	*EPS8*
CLEC7A	*COTL1*
NEAT1	*DHRS9*
CLEC4E	*HLA-DMB*
CRISPLD2	*HLA-DMA*
MEFV	*PYCARD*
ACSL1	*LOC644936*
NCF1	*SH3BGRL3*

Supplementary Table 10. Differential gene expression averaged across 500 progenitor ce

Gene	log2FC	FDR q-value
ANXA1	-2.6974206	0
CD99	-2.2820004	0
MYADM	-2.733183	0
TAGLN2	-1.9249345	0
TSC22D3	-2.9993758	0
VIM	-3.6780996	0
LMNA	-3.6389994	4.10E-302
CRIP1	-4.0560232	1.92E-286
TUBA1A	-2.5670584	1.48E-269
RPS10	1.09409307	4.08E-252
RGCC	-3.3617637	3.69E-251
ADGRE5	-1.9630999	1.72E-245
EIF1	-1.2130854	1.46E-239
ATP2B1	-2.0437947	1.43E-237
UBC	-1.4030298	9.65E-216
RPS29	0.73806574	2.43E-215
JUND	-1.5843134	1.31E-213
RPL38	0.86553586	1.06E-207
AC020916.1	-2.0424889	3.84E-205
EMP3	-1.8603933	6.30E-203
TUBB4B	-1.6948327	1.23E-201
PLCG2	1.73889251	1.58E-201
TPSAB1	3.14224632	9.83E-194
MTRNR2L12	1.32960747	1.06E-189
LTC4S	1.45301171	2.62E-186
AHNAK	-2.0062326	9.35E-180
EMP1	-1.9737394	1.77E-179
RPS27	0.66449818	2.45E-179
TGFB1	-1.3589861	4.55E-178
CLIC1	-1.0599698	1.49E-177
CD44	-1.4139905	4.04E-177
SAT1	-1.810989	2.29E-174
MGST1	1.63918342	5.11E-173
CYTL1	2.30323256	7.35E-172
LRRFIP1	-1.3394138	3.80E-171
RPL31	0.73453269	2.51E-170
UCP2	-1.5624121	1.03E-169
MCL1	-1.5763477	1.50E-165
IRF1	-1.4870665	1.00E-154
SLC40A1	1.54519055	2.25E-152
SNHG6	0.96096112	7.02E-148
RTN4	-1.1290849	2.98E-145
MYL6	-0.9907065	3.40E-144
KLF6	-1.6926249	5.53E-144
CALM1	-1.3234723	3.69E-142
RPL37	0.58034541	2.06E-140
RAB11FIP1	-1.2910544	7.12E-140

PPP1R15A	-1.1996037	4.28E-129
TUBB6	-1.032931	3.49E-127
MMP2	1.04829923	1.03E-126
APOC2	1.29550691	2.08E-126
ANPEP	1.01018428	2.76E-122
GNA15	-1.0148793	4.63E-122
EFHD2	-1.036381	5.46E-122
RARA	-0.9494113	4.89E-119
PER1	-0.8811781	7.47E-119
HSPA5	-1.9722822	1.37E-118
PDLIM1	-0.9238836	5.95E-118
ORAI1	-0.9288289	1.88E-116
MAP2K3	-1.1052079	3.96E-116
CD52	-1.2972961	5.63E-116
ANKRD28	-1.0756378	9.73E-116
MIDN	-1.0952485	3.53E-114
MYB	1.00199086	8.06E-114
MYC	1.0027345	6.77E-113
TNFRSF12A	-0.9224185	1.37E-112
ZFAS1	0.86367636	2.31E-112
UQCR10	0.83424068	1.79E-109
CREB5	-0.9755295	4.24E-109
RPL39	0.49607268	9.89E-109
ZFP36	-1.3358433	1.07E-108
TPM4	-1.177647	1.47E-108
RPS9	-0.5469057	2.21E-108
SLC44A1	0.90458732	4.28E-108
GLIPR2	-0.8639548	1.15E-107
AIF1	-0.8685854	4.49E-107
SOX4	1.37021265	6.13E-107
CTSD	0.88867636	9.14E-107
VSIR	-1.1052289	1.23E-106
AKAP13	-1.0324252	3.71E-106
MT-ND4L	0.8833201	1.18E-105
JUNB	-1.4384736	3.23E-104
CRIP2	-0.7457232	7.85E-103
ZBTB7A	-0.9192055	8.75E-103
RPL34	0.49323596	7.91E-102
RPS20	0.65462472	9.50E-101
SEPTIN11	-0.9636516	1.23E-100
PLK3	-0.8735534	2.24E-100
S100A11	-1.3080064	3.09E-100
PWWP3A	0.57170648	2.47E-99
RASL10A	-0.6764697	2.78E-99
AL355922.1	0.7595787	4.44E-99
CIRBP	-0.8388411	1.10E-98
MTURN	-0.8639262	1.92E-98
H1FX	-1.6938784	2.86E-98
GABPB1-AS	1.05327393	4.17E-98
IDS	-0.9510931	5.98E-98

SORL1	1.03587789	2.43E-97
SH3BGRL3	-0.8522189	3.86E-97
H3F3A	-0.5900456	5.36E-97
PABPC1	0.74131603	5.87E-97
MT-ATP8	0.90572535	1.11E-96
BCL2	0.62212086	7.21E-96
FOSB	-1.1492029	9.80E-96
RPL35A	0.46701293	3.66E-95
LST1	-0.9579725	5.16E-95
NAIP	0.56997162	6.45E-95
LYN	-0.8471912	4.38E-94
PRR7	-0.6654126	2.45E-93
CDKN1A	-0.7299736	5.06E-93
KIT	0.79256751	8.91E-93
HES1	-1.1910289	1.69E-91
DSTN	-0.8761812	2.06E-91
LINC01226	0.57150607	2.09E-91
PRXL2C	-0.8187203	2.47E-91
RAP1B	-0.8573844	3.60E-91
TSC22D1	-1.1306032	4.85E-91
ICAM3	-0.7675359	2.86E-90
RASGRP3	0.81424717	5.62E-90
HMGN1	0.62865292	5.79E-88
LARS	0.73186766	7.66E-88
PHLDA2	-0.7588794	1.27E-87
CITED2	0.7934308	2.88E-87
RNASE2	1.12106751	6.84E-87
TXN	-0.8186493	4.64E-86
MIS18BP1	0.79515337	5.70E-86
FYB1	0.79924071	7.54E-86
KCTD9	-0.9768716	5.60E-85
HSD17B4	0.66595974	1.30E-84
ST8SIA6	0.68871887	2.59E-84
SLC9A3R1	-0.8955429	3.19E-84
SPIN4	0.61577528	7.29E-84
PCBP2	0.6507924	1.27E-83
RPS17	0.62535002	1.42E-83
PNP	-0.8954521	1.45E-83
ITGA5	-0.7613676	1.55E-83
LINC01268	0.62351376	1.97E-83
DNAJB6	-0.8460499	2.62E-83
ST3GAL6	0.43242129	2.86E-83
KMT2E	-0.7563822	3.49E-83
ANGPT1	0.98621889	5.80E-83
RBM38	-0.8088177	6.79E-83
MYCN	0.61163652	6.81E-83
AC245060.5	0.81220073	8.41E-83
CAPG	0.78009752	2.39E-82
CXXC5	-0.9560445	3.92E-82
PPIL4	-0.7341789	4.85E-82

IFITM10	0.35016314	5.28E-82
TPM3	-0.7436446	8.89E-82
MPC2	-0.6974314	1.40E-81
MAT2A	0.72902563	1.69E-81
HERC2	0.61914715	1.73E-81
IFT57	-0.7068963	2.74E-81
GTF2I	0.74163734	4.26E-81
MRC2	0.44876069	4.30E-81
TMSB10	-0.7078046	8.42E-81
JCHAIN	-1.7196723	1.49E-79
RGS18	0.57428502	1.81E-79
LRPAP1	0.77416893	4.56E-79
RASGEF1B	-0.6713388	5.58E-79
HIF1A	-0.8312706	5.67E-79
ACSM3	1.02637586	6.80E-79
URI1	0.69362026	9.82E-79
FAM30A	-0.9955689	6.08E-78
SERBP1	0.64884815	1.55E-77
TREM1	-0.7788406	2.15E-77
LMNB1	0.51367311	4.74E-77
KLF3	-0.8282717	7.83E-77
VMP1	-0.7918617	1.89E-76
RELB	-0.7677596	3.12E-76
FSCN1	-0.8029673	5.30E-76
NFATC1	-0.7100713	6.83E-76
KNOP1	0.52762706	1.62E-75
RPL37A	0.42703289	1.96E-75
MT-ND6	0.78521975	3.89E-75
PHF20	-0.7523418	1.02E-74
NDUFV2	-0.6473749	1.32E-74
HTRA3	0.45983633	1.92E-74
AL627171.2	0.67263484	1.99E-74
MBOAT7	-0.7476926	2.16E-74
NACA	0.43246715	4.73E-74
FRY	0.48752687	8.07E-74
YBX3	-0.8703873	2.60E-73
IRF2BP2	0.80679355	9.83E-73
NEAT1	-1.0198013	1.92E-72
KRAS	-0.7066414	2.10E-72
ARID4B	-0.7861258	2.24E-72
NDUFB1	0.64379731	2.44E-72
LY6E	-0.8388744	1.17E-71
MBP	-0.7438181	1.24E-71
DDAH2	-0.7432204	1.31E-71
S100A6	-1.1242583	2.74E-71
PIM3	-0.7530294	8.69E-71
RPS11	0.45045669	1.70E-70
TOP2B	0.74092227	1.87E-70
UBE2B	-0.6639425	1.88E-70
ID1	-1.4815564	2.06E-70

MEX3B	0.37006259	2.12E-70
HMGB1	0.64721509	7.19E-70
CD74	-0.8991125	9.00E-70
HLA-A	-0.6939658	9.34E-70
MANF	-0.7347408	9.71E-70
RALGDS	-0.7934938	1.05E-69
YPEL5	-0.8759533	1.90E-69
MRPL33	0.67135685	3.15E-69
YWHAZ	-0.8238131	3.22E-69
ARF6	-0.6964692	7.73E-69
PRKDC	0.6614071	9.53E-69
NARS	0.60693795	9.83E-69
CBX3	0.62336677	1.17E-68
VAMP8	0.60132389	1.37E-68
RNASE3	0.45055065	1.46E-68
R3HDM4	-0.6018552	1.57E-68
AL034397.3	0.58162962	1.58E-68
GAPT	0.66349024	1.96E-68
HCST	-0.9391117	2.78E-68
RSL1D1	0.61595434	3.22E-68
RPS28	0.3603081	3.37E-68
CSNK1A1	-0.6782394	4.31E-68
SH2B3	-0.5997514	4.85E-68
FBXO34	-0.7169805	6.23E-68
CD38	0.57689345	1.07E-67
CAT	0.67701936	2.16E-67
TMEM161B-/	0.72449433	2.35E-67
RALGAPA2	0.51707777	3.00E-67
BPTF	0.72538398	3.28E-67
RAC1	-0.5856071	5.32E-67
NAP1L1	0.69496898	8.50E-67
LINC02470	0.49777902	9.24E-67
C11orf54	0.42373154	1.32E-66
SERF2	-0.4831417	1.62E-66
ATP8B4	0.77213898	3.73E-66
CTR9	0.74448095	4.17E-66
TENT5A	0.55034298	8.85E-66
EZR	-0.8644081	2.20E-65
TP53	0.38162827	2.22E-65
SYNJ2	-0.7996188	5.34E-65
CD84	0.57809704	7.37E-65
CBX1	0.37186455	8.43E-65
RHOA	-0.6580268	1.31E-64
CYTH4	-0.7010823	1.51E-64
TIPARP	-0.815914	1.66E-64
UBB	-0.6211001	1.71E-64
OMA1	0.31449344	2.92E-64
JUN	-1.193584	4.58E-64
LSP1	-0.7621611	4.64E-64
ALOX5AP	-0.7016797	5.32E-64

CDH26	0.31467911	9.20E-64
TACC1	-0.7011355	1.34E-63
LRPPRC	0.53568613	1.51E-63
ATP2B1-AS1	-0.6923183	1.93E-63
MZB1	-0.7643753	2.25E-63
PLXDC2	0.57189016	2.78E-63
SGMS2	-0.5241783	3.20E-63
MT-ND3	0.55997443	3.22E-63
GNAI2	-0.6346656	3.71E-63
SPINK2	-0.754124	4.91E-63
TPPP	-0.4170585	6.53E-63
JMJD1C	-0.7956556	6.82E-63
RIPOR2	-0.6633149	7.30E-63
ZSCAN18	-0.558345	1.04E-62
ZNF66	0.3753942	1.09E-62
UPP1	-0.6251533	1.26E-62
C16orf54	-0.5636699	1.39E-62
DYNLT1	-0.591388	1.52E-62
AC244502.1	0.98911547	1.69E-62
CENPJ	0.47801633	2.14E-62
S100A10	-1.1494807	3.20E-62
PLP2	-0.6809263	5.29E-62
MAP7D1	-0.6241055	5.78E-62
SAMHD1	-0.5664814	6.33E-62
NPDC1	-0.6359328	7.79E-62
PPP3CA	0.5219	8.21E-62
GATD3A	0.40600984	8.86E-62
CMTM3	-0.6813344	1.11E-61
EXOSC5	0.38911419	1.54E-61
ACTN1	0.56560175	3.07E-61
RCSD1	0.50855888	3.53E-61
NINJ1	-0.6921484	3.89E-61
ELMSAN1	-0.7213132	5.39E-61
CD96	0.69873204	8.66E-61
YWHAQ	-0.5296341	9.98E-61
CYTH1	-0.7096039	1.06E-60
PNISR	0.59964212	1.12E-60
GSN	-0.6471872	1.57E-60
RBM12B	0.42491705	2.31E-60
NOL8	0.52921155	3.92E-60
ODF3L1	-0.3295742	4.26E-60
RELL1	-0.7014424	5.09E-60
LGALS1	-1.559372	5.31E-60
CHCHD2	-0.5033848	6.09E-60
UBTF	0.4709071	6.26E-60
CYTOR	-0.7095258	7.99E-60
HIPK1	-0.6285611	8.86E-60
RPL41	0.32568444	1.10E-59
KLF13	-0.6685914	1.18E-59
TMOD3	-0.5456386	1.46E-59

STAT1	0.41928879	1.52E-59
RHOG	-0.6499176	3.47E-59
THEMIS2	-0.6086422	3.78E-59
SPART	0.71432075	4.51E-59
PTP4A3	-0.5558287	4.95E-59
MTSS1	-0.4117939	7.44E-59
RPL15	-0.3880319	8.41E-59
PIM1	-0.7360319	1.24E-58
CFL1	-0.5908045	1.25E-58
CDC42	-0.5538094	1.31E-58
H3F3B	-0.8404185	1.45E-58
BCL3	-0.5968088	1.63E-58
ZNRF1	0.44115441	1.76E-58
HSP90B1	-1.086205	2.08E-58
SNX2	-0.6194247	2.10E-58
ARL4A	-0.6485114	2.47E-58
CYTIP	-0.6309972	3.71E-58
RPL23	0.50649505	4.70E-58
G3BP2	-0.63612	4.78E-58
CSRNP1	-0.5374179	4.90E-58
IFFO2	-0.4990381	5.05E-58
ASAH1	0.77253679	6.03E-58
TRIM73	0.46514366	6.36E-58
SET	0.54756166	6.72E-58
FTX	0.70136687	8.59E-58
MFSD1	0.54961003	1.02E-57
SRSF3	0.61357772	1.08E-57
ETF1	-0.6783039	1.64E-57
PPP1R18	-0.5680168	2.06E-57
GAS2L1	-0.3380015	2.07E-57
MDK	-0.7215902	2.30E-57
TUBB	-0.7745298	3.01E-57
GNAI3	-0.5423482	3.24E-57
PLPPR3	0.62929609	3.66E-57
ARPC5L	-0.492111	3.68E-57
ZBTB43	-0.5822644	3.85E-57
TSEN34	-0.5627648	5.40E-57
SMC2	0.44501494	6.40E-57
TASOR	0.42759447	1.79E-56
CAPZA2	-0.5965556	1.92E-56
RILPL2	-0.6670998	2.40E-56
RPLP2	0.31362797	2.91E-56
RPL30	0.32799883	3.31E-56
MTRNR2L8	0.51873569	3.44E-56
ZBTB44	0.56359957	4.58E-56
NPTX2	-0.427179	4.84E-56
TMBIM4	0.5720632	5.74E-56
LINC-PINT	-0.6253175	5.97E-56
NPC2	0.54679448	6.21E-56
NCF4	0.61980701	8.55E-56

SRSF7	0.67928953	1.03E-55
RPAP2	0.70503459	1.23E-55
C16orf74	0.33429027	1.44E-55
CFD	1.44848675	1.83E-55
AHR	-0.6304411	1.98E-55
IL1RAP	-0.6602295	3.01E-55
PARP4	0.44837764	3.07E-55
MTIF3	0.54576627	3.56E-55
TSTD1	-0.6180974	6.18E-55
WTAP	-0.6237599	6.91E-55
RPS21	0.34874188	7.29E-55
APAF1	0.34386366	8.34E-55
BIN1	-0.5183095	9.35E-55
SIK3	-0.5600025	9.40E-55
HIST1H4C	0.45046912	1.72E-54
LUC7L3	0.56820822	1.81E-54
WASHC1	0.44051304	1.99E-54
TTC3	0.68332996	2.79E-54
CDKN2D	-0.6083945	2.91E-54
WDFY2	-0.6644279	3.74E-54
ANTXR2	-0.63814	5.61E-54
PIGM	0.35370937	5.69E-54
KPNA4	-0.4954174	7.00E-54
UBE2N	-0.4696156	8.78E-54
NAMPT	-0.7531864	1.37E-53
ADAM17	-0.6651494	1.70E-53
SOCS3	-0.5223127	1.71E-53
IL1B	0.37911173	2.78E-53
TLE5	-0.547993	3.02E-53
EGFL7	-0.7117309	3.96E-53
KLF10	-0.6854863	4.13E-53
FCER1G	-0.6932999	4.59E-53
MAFF	-0.6196221	5.02E-53
DEPTOR	0.25036107	5.35E-53
GOLGA8R	0.30916071	6.19E-53
CCND3	-0.6159351	7.73E-53
SRSF5	0.50068455	7.96E-53
ARHGDIA	-0.5144051	8.01E-53
DBI	-0.6885679	8.66E-53
KDM6B	-0.6870278	8.93E-53
GOLGA8N	0.35684623	1.50E-52
SAP18	-0.6685033	1.57E-52
DLD	-0.4572061	2.00E-52
COX6B1	0.52557609	2.33E-52
FAM217B	0.33396983	2.50E-52
H19	-0.8199189	2.85E-52
CARD19	-0.5233279	4.93E-52
ZNF737	0.3537126	5.00E-52
PTPRC	-0.6716935	5.17E-52
RABAC1	-0.5387286	6.21E-52

TTC17	0.43976063	6.66E-52
TRAF4	-0.5701537	6.67E-52
WDR12	0.36695977	1.39E-51
SLC35E4	-0.5299522	1.79E-51
PTPRE	-0.7021456	2.22E-51
AC096667.1	0.29770173	2.60E-51
ETFB	-0.527388	2.66E-51
TFG	-0.4429358	2.85E-51
LAMP2	0.53438312	5.13E-51
GAS5	0.53242848	5.21E-51
RBBP4	0.51282673	6.39E-51
Z97192.3	0.38236176	6.43E-51
ATRX	0.66256601	7.17E-51
ASCL2	0.2387863	7.30E-51
RIOK3	-0.5773867	8.29E-51
STK17B	-0.6039626	1.03E-50
PPP4R3B	0.46142831	1.07E-50
PDCD11	0.33297852	1.12E-50
PRDX3	0.44743326	1.39E-50
LUZP1	-0.5649618	1.64E-50
TNFRSF1A	-0.4975975	1.92E-50
CNR2	0.30066005	1.97E-50
CNST	-0.6364985	2.98E-50
LMO4	0.53268735	5.87E-50
EDEM3	0.45931444	6.03E-50
TSPAN2	-0.4070613	6.73E-50
TIMP1	-0.6795239	8.26E-50
CITED4	0.30662019	1.11E-49
OIP5-AS1	0.55201478	2.20E-49
BCO2	-0.4905713	2.33E-49
ZNF680	0.27056724	2.40E-49
TMEM63A	-0.4840842	2.75E-49
PSMD12	-0.4424931	2.91E-49
LDHA	-0.6109836	3.41E-49
SMC4	0.66597704	3.83E-49
FUCA1	0.358464	4.02E-49
MAP3K8	-0.6038392	5.84E-49
CKAP2	0.36649424	7.30E-49
ARGLU1	0.60377438	9.29E-49
AGO2	-0.5488434	9.63E-49
TTC1	0.41651919	9.69E-49
UBE2D2	-0.542855	1.00E-48
ARL8A	-0.4502618	1.29E-48
GPR183	-0.8403182	1.52E-48
SPNS3	-0.5352891	1.58E-48
TSG101	-0.4130362	2.13E-48
GOLIM4	0.50064067	2.54E-48
DUSP6	-1.0025327	2.82E-48
H2AFJ	-0.5145425	3.29E-48
HNRNPUL1	-0.5233577	4.79E-48

LRRFIP2	-0.5866882	4.89E-48
CLEC2D	0.22140046	5.20E-48
MRPL34	0.43819287	5.74E-48
GAPDH	-0.4235196	6.88E-48
LRRK1	0.30239198	7.72E-48
S100A4	-1.0603466	7.98E-48
GCSH	0.37322959	8.66E-48
PAK2	0.46190937	8.89E-48
ITM2C	-0.6717356	1.10E-47
HCG18	0.49188063	1.15E-47
CHST12	0.40403766	1.38E-47
ITGA4	0.65000853	1.60E-47
TARBP1	0.47029131	1.63E-47
SYAP1	-0.7814528	2.13E-47
MIB1	0.42092073	2.29E-47
PTPN1	-0.5537686	2.42E-47
AL354733.3	0.24003295	2.66E-47
SETDB2	0.42995777	2.71E-47
CLEC2B	-0.5994896	3.15E-47
DENND11	0.37379695	3.54E-47
ARL4C	-0.4895534	4.17E-47
CYB5A	0.27974532	4.50E-47
AC104083.1	0.2336449	5.16E-47
RARA-AS1	-0.3833336	5.50E-47
ETV6	0.4963417	5.95E-47
RBM28	0.37269078	6.02E-47
PSTPIP2	0.46449906	6.51E-47
GMCL1	0.45857491	8.37E-47
HSPE1	0.61375354	9.33E-47
UBR5-AS1	0.44258269	9.86E-47
EHMT2	0.31182651	1.01E-46
ATM	0.45056494	1.06E-46
YWHAH	-0.5310725	1.20E-46
SOS1	0.36619817	1.71E-46
PRKD2	-0.362484	1.99E-46
VEGFB	0.33615326	2.13E-46
IQGAP1	-0.5555608	2.47E-46
RNASEH2B	0.61057672	2.50E-46
GALNT1	0.44026083	2.51E-46
FCHSD2	0.34337515	2.81E-46
IQCH-AS1	0.24880903	3.08E-46
LINC00342	0.62301144	3.60E-46
PLEC	-0.5899258	4.28E-46
PYGL	0.46206589	5.43E-46
ARHGAP27	-0.5310192	5.50E-46
YTHDC1	0.49210656	5.62E-46
TMA16	0.42855115	7.27E-46
CSNK1D	-0.4662808	9.16E-46
HLTF	0.35097218	1.07E-45
ZNF804A	0.25273918	1.27E-45

PPP1CB	-0.5835629	1.32E-45
SLC20A1	-0.5792477	1.33E-45
TWF2	-0.5166929	1.50E-45
CFP	-0.4207009	1.59E-45
SLC39A3	0.50341361	1.68E-45
PER2	-0.4463266	1.85E-45
EVI2B	0.54100643	2.53E-45
BAIAP2	-0.4504845	2.93E-45
ELF1	-0.6370083	3.03E-45
DENND4A	-0.5484726	3.27E-45
ARHGAP4	-0.4362004	3.89E-45
ST13	0.47253387	4.63E-45
RAD50	0.42476636	4.70E-45
NSA2	0.54919554	5.01E-45
EXOSC6	0.39798891	5.66E-45
LFNG	-0.4825168	5.94E-45
GARS-DT	0.36512357	6.21E-45
KCNE3	0.34876854	6.72E-45
YPEL3	0.48394051	7.10E-45
SUB1	-0.4933681	7.84E-45
SGO2	0.29419454	7.85E-45
GPATCH2	-0.468366	8.24E-45
RHOC	-0.5157534	1.10E-44
SEC61B	-0.4970211	1.12E-44
MRPS26	0.30750467	1.19E-44
AGL	0.26365403	1.24E-44
ACOT7	-0.3457832	1.26E-44
ZMYM4	0.3865584	1.26E-44
SLBP	-0.4115055	1.38E-44
S1PR4	-0.3699272	1.40E-44
PCF11	0.53140737	1.60E-44
ENSA	-0.4515399	1.64E-44
FDX1	-0.423047	2.20E-44
AC092683.1	0.40576851	2.23E-44
TNRC18	0.48907638	2.25E-44
TMX4	-0.6913336	2.34E-44
SPOP	-0.3752171	2.35E-44
IMPA2	0.25708345	3.07E-44
NR3C1	-0.4928003	3.50E-44
ST6GALNAC	-0.4003238	3.53E-44
MAN1A1	0.41704904	3.62E-44
CHD1	-0.6001375	3.80E-44
MITD1	0.38143262	3.98E-44
BOD1L1	0.57391623	4.00E-44
BAALC	-0.5821022	4.20E-44
ARID5A	-0.5085851	4.33E-44
RPL36A	0.3541521	4.95E-44
ADI1	0.41385641	5.75E-44
ARMCX3	-0.5998741	5.93E-44
STRAP	-0.449987	6.54E-44

MCRIP2	0.28436743	8.05E-44
SRSF6	0.58993264	8.64E-44
CASP8	-0.532206	9.40E-44
NDE1	-0.4160869	9.78E-44
TOMM7	0.42823291	1.05E-43
RAB1A	-0.4013122	1.05E-43
IGSF10	0.32240576	1.17E-43
COMMD6	0.43005374	1.25E-43
GPSM2	0.31160773	1.34E-43
JUP	-0.4751726	1.51E-43
TMX1	0.37365041	1.61E-43
TMCC1-AS1	0.31554746	1.69E-43
BMP2K	0.45696776	1.74E-43
SERTAD1	-0.5083785	1.89E-43
SFXN3	-0.4395623	2.00E-43
ZC3H8	0.30529221	2.12E-43
RBM14	-0.497172	2.22E-43
MAP1LC3B	-0.5329168	2.32E-43
TMEM173	-0.4706775	2.34E-43
MOB3A	-0.4640523	3.58E-43
AL021368.2	0.41010532	3.98E-43
ALG8	0.18011692	4.20E-43
HES4	-0.5224448	4.32E-43
LEPROTL1	-0.500181	4.51E-43
ZNF141	0.34656477	5.15E-43
CA5B	-0.4325605	5.30E-43
RPL13A	0.28380308	6.36E-43
HIC1	-0.4334861	6.49E-43
TPPP3	-0.3319023	6.61E-43
TNFRSF4	-0.7147495	7.29E-43
HSPD1	0.58782727	7.35E-43
PRR12	0.23987254	8.61E-43
DMAC1	0.31352167	9.24E-43
HOXA3	0.34674397	9.25E-43
PCMTD2	0.24595919	9.35E-43
PTER	0.21450207	1.29E-42
GOLGA8O	0.23032033	1.43E-42
LRMDA	0.3431264	1.45E-42
ATP2A2	-0.4184103	1.47E-42
APOLD1	0.28482292	1.60E-42
PLCB1	-0.5146646	1.63E-42
PPP6R1	-0.4225643	1.65E-42
AGPAT4	-0.5129637	1.82E-42
LPCAT2	0.6242482	2.16E-42
PRH1	0.22148805	2.49E-42
SARAF	-0.4983728	3.27E-42
HERPUD1	-0.5052043	4.65E-42
CMSS1	0.36216204	4.78E-42
SLC35A1	0.22855832	5.33E-42
NUDT1	-0.4458739	5.48E-42

KANK1	0.22195006	7.15E-42
CYBA	-0.499297	7.69E-42
VPS13C	0.52905761	8.55E-42
C15orf39	-0.4372682	9.69E-42
TNFRSF18	-0.4633804	1.02E-41
PPA2	0.33699615	1.04E-41
PHLDA1	-0.6597785	1.13E-41
CD37	-0.5559847	1.17E-41
POGLUT1	0.25382378	1.56E-41
BUD31	-0.3919105	1.90E-41
PNPT1	0.30150857	2.01E-41
AZU1	1.07642114	2.28E-41
SUDS3	0.22808034	2.34E-41
ARMC10	0.3296715	2.51E-41
NCL	0.52797347	2.72E-41
ABCA1	-0.3865762	2.77E-41
NRDC	0.47666865	2.82E-41
RXYLT1	0.3470553	2.93E-41
P2RY8	0.41477776	3.18E-41
HAT1	-0.3859598	3.70E-41
DAAM1	-0.5628998	3.82E-41
UBE2H	-0.5357236	4.08E-41
ABCC4	0.46339905	4.25E-41
RHOF	-0.457836	4.34E-41
ZSWIM7	0.34758384	5.14E-41
ANXA11	-0.4027883	5.59E-41
SPNS2	-0.4957706	7.44E-41
PHF14	0.4297929	7.65E-41
NUP42	0.30592131	8.46E-41
DLEU1	0.27177388	9.11E-41
ISOC2	0.25245273	9.42E-41
PPM1F	-0.3911713	1.02E-40
PCNA	0.06907133	1.07E-40
BRD7	0.45819573	1.18E-40
RRAGC	-0.394476	1.19E-40
RPL18A	-0.4424739	1.23E-40
AHSA1	0.29068033	1.25E-40
GPR27	0.30405613	1.29E-40
STOML3	-0.2589197	1.41E-40
LYRM7	0.40563125	1.56E-40
OCLN	0.29572835	1.74E-40
AK2	0.4740107	1.75E-40
HLA-C	-0.5735943	2.27E-40
SLC9A3R2	-0.5888081	2.50E-40
STAB1	0.43521658	2.53E-40
DUSP14	-0.4205599	2.81E-40
STARD4-AS	0.23804696	3.48E-40
LINC00926	0.21654448	3.75E-40
MT-ND2	0.44750305	3.79E-40
ACADM	0.42734197	4.03E-40

LINC00996	0.27359048	4.44E-40
OPN3	0.24525757	4.79E-40
MDN1	0.35446382	5.83E-40
CD300A	-0.4311023	6.41E-40
CMTM6	-0.4812539	7.33E-40
HLA-B	-0.5731347	7.80E-40
MANBA	0.36488385	8.02E-40
NT5C	-0.4187658	8.48E-40
PA2G4	0.52517069	8.58E-40
NUFIP2	0.50541541	8.99E-40
CKS2	0.28555584	1.05E-39
NSF	0.24976174	1.23E-39
HTR1F	0.24101942	1.41E-39
ZNF706	-0.4085254	1.45E-39
NEK9	0.1984628	1.50E-39
ATF3	-0.4680496	1.51E-39
CREB1	0.36159664	2.15E-39
CREBZF	0.32468376	3.01E-39
TMEM106B	0.31459987	3.11E-39
MRPS34	0.37378815	3.12E-39
BCL7B	-0.384161	3.70E-39
N4BP3	-0.3030482	3.96E-39
MTAP	0.2906581	4.39E-39
PDGFA	-0.3851197	4.63E-39
SVIL	-0.6000259	5.47E-39
VPS37B	-0.4500636	5.95E-39
UBAC1	0.21321365	6.23E-39
DENND6B	-0.429822	6.62E-39
UBE2D1	-0.4743703	6.80E-39
INKA1	-0.4596347	7.19E-39
PPP1R3E	0.34331282	7.50E-39
PDCD10	-0.4033523	7.74E-39
FAM177A1	-0.3571264	8.44E-39
RHEX	0.65824893	8.92E-39
TRA2B	0.54254375	1.02E-38
RNF126	-0.3392245	1.08E-38
TESPA1	0.45452135	1.15E-38
R3HDM2	0.4423998	1.18E-38
CKS1B	0.08918227	1.19E-38
GGCT	0.38186069	1.33E-38
PCBP1-AS1	0.29963527	1.34E-38
LACC1	0.32429495	1.49E-38
POLR2H	0.23621401	1.53E-38
AC005670.3	0.32926382	1.53E-38
DPYSL3	-0.2732731	1.67E-38
ZNF302	0.31489457	1.82E-38
TNFAIP2	-0.595829	2.19E-38
NREP	0.39705834	2.32E-38
LINC01409	0.24068514	3.33E-38
SLC35E2A	0.25565156	3.73E-38

TXNDC5	0.39224502	3.73E-38
CKLF	-0.7040361	3.79E-38
REXO2	0.31581939	4.12E-38
POLR3K	0.20973095	4.30E-38
CCN3	-0.3017872	4.44E-38
PSMG4	0.20287828	4.59E-38
PPP2CA	-0.3768363	4.88E-38
PDLIM2	-0.3746072	5.08E-38
AGPAT2	-0.3210446	5.12E-38
SDHAF3	0.2192507	5.14E-38
HOMER3	0.37014346	6.68E-38
TRIM25	0.23748351	7.59E-38
NOP56	0.46980239	8.15E-38
ZBTB20	0.54936087	8.48E-38
SMC1A	0.32176078	8.70E-38
MALT1	-0.3993893	9.38E-38
IL2RA	-0.5217613	9.38E-38
CD81	0.41563816	1.06E-37
ZNF891	0.28172164	1.09E-37
TNFRSF1B	-0.4179556	1.20E-37
EGR1	0.50730669	1.24E-37
DCTN6	-0.394063	1.26E-37
CDK6	0.4928239	1.26E-37
RSRC2	-0.5012935	1.42E-37
SKAP2	0.49767185	1.50E-37
PDXK	-0.3385889	1.54E-37
WDR3	0.22885903	1.55E-37
SNAI3	-0.4394156	1.65E-37
NOLC1	0.31664472	1.93E-37
ADPRHL2	-0.2363282	1.94E-37
CIAO2A	0.30283292	1.94E-37
SERPINE1	-0.3970334	2.26E-37
WDR70	0.35114716	2.29E-37
CFAP126	-0.2004369	2.48E-37
HSP90AB1	0.39234963	2.55E-37
KIF9	-0.4027681	2.57E-37
DDX10	0.30610775	2.64E-37
NTPCR	0.26630125	2.68E-37
CREG1	0.48957062	2.72E-37
UBE2V2	0.2836283	2.91E-37
CORO1C	-0.4508069	2.94E-37
FAM111A	0.31626877	3.43E-37
UQCRFS1	-0.3986275	3.49E-37
AKIRIN2	-0.4039419	3.50E-37
PSMB1	-0.4196938	3.68E-37
SUMO3	0.3005875	4.03E-37
ZFPM1	0.18218738	4.20E-37
HLA-DPA1	-0.5966477	4.58E-37
RRP12	-0.5146275	4.90E-37
RPL12	0.30303527	4.98E-37

ECHDC1	0.28485595	5.57E-37
ZMAT5	0.25247846	5.76E-37
HIST2H2BE	-0.5064936	6.15E-37
ME2	0.35116188	6.44E-37
XYLT1	0.22486037	7.32E-37
EIF5	-0.5187415	7.35E-37
SNHG25	0.35050672	7.66E-37
FMC1	0.28276665	7.69E-37
RBM17	-0.38888	1.04E-36
WASF2	-0.4788275	1.05E-36
NR1H2	-0.3434461	1.09E-36
EPM2AIP1	0.39005169	1.20E-36
PRKCQ-AS1	0.26760023	1.32E-36
POLE3	0.30884911	1.50E-36
MAPKAPK3	-0.3687337	1.93E-36
PRRG4	0.28161311	2.10E-36
WRNIP1	0.25427347	2.11E-36
MCPH1	0.21791065	2.27E-36
EMC3	-0.375686	2.33E-36
TOP1MT	0.30274538	2.65E-36
ARL5A	0.31922137	2.65E-36
LRRC75A	0.84821096	3.35E-36
FRAT2	0.21517789	3.63E-36
HDDC2	0.2777385	3.67E-36
CCDC6	-0.3550793	4.05E-36
FAM120A	0.37284104	4.06E-36
ENDOG	0.24530389	4.09E-36
CD302	0.4675068	4.11E-36
CAPNS1	-0.3705517	4.21E-36
ARL6IP1	0.43563402	4.54E-36
RPL22	0.25366721	4.70E-36
KCNK17	-0.3737008	4.88E-36
ABRAXAS1	0.25293721	4.89E-36
TMEM131	0.34286397	5.12E-36
ERGIC1	-0.3525549	5.19E-36
KAT6B	0.36277255	5.64E-36
PDCD4	0.50955304	5.72E-36
ADRB1	0.25455865	5.81E-36
ALDOA	0.33823301	6.12E-36
ARG2	-0.2440479	6.37E-36
UTP3	0.32554615	6.46E-36
DDIT4	-0.7759737	7.03E-36
IGHM	-0.6262608	8.20E-36
INKA2	-0.3167789	8.26E-36
PCYOX1	0.20583877	9.01E-36
TNFAIP8	0.55662482	9.49E-36
DNMT1	0.32415907	9.76E-36
FMNL1	-0.4057882	9.98E-36
DCTN3	-0.278569	1.27E-35
TERF2	-0.3107813	1.28E-35

DDX18	0.44697212	1.38E-35
TMX3	0.25807634	1.41E-35
CRLS1	0.16384256	1.42E-35
SMIM12	-0.2530326	1.50E-35
ACIN1	0.34186429	1.56E-35
HADHA	0.39176053	1.57E-35
TFAP4	0.16836993	1.67E-35
TNFSF13B	0.51630848	1.73E-35
CELF1	-0.4424132	1.96E-35
SEC14L1	-0.3666834	2.05E-35
PTPRA	0.36662341	2.16E-35
ZNF605	0.23181741	2.17E-35
GRK2	0.33028611	2.29E-35
PRKACB	0.5552489	2.31E-35
BRI3BP	0.38409632	2.35E-35
AC007384.1	-0.3740951	2.40E-35
LTB	-0.6495926	2.44E-35
TBCA	0.4796246	2.54E-35
CCDC66	0.25684431	2.56E-35
FAM122C	-0.3569966	2.72E-35
NT5DC1	0.20197131	2.77E-35
KRCC1	0.32101114	2.87E-35
AC026471.4	0.16500295	3.00E-35
POP5	0.26597297	3.11E-35
SLC39A10	0.40458712	3.27E-35
AKIRIN1	-0.3374896	3.66E-35
PIN4	0.27142341	3.86E-35
PAXIP1-AS1	0.20226124	4.05E-35
NDUFAF2	0.26687245	4.13E-35
RNF130	0.48371972	4.51E-35
ZNF770	0.25253931	4.72E-35
ADGRG1	-0.4810001	5.13E-35
TRADD	0.14185026	5.50E-35
ATF4	-0.4789425	5.50E-35
NCOA4	0.49209658	5.54E-35
SKI	0.21727344	5.59E-35
NOP14	0.27536444	5.76E-35
HIST1H1C	-0.9008035	6.18E-35
USP14	-0.4029549	6.47E-35
VPS41	0.25268461	7.31E-35
AGPAT5	0.32947276	8.50E-35
ZNF431	0.51454309	8.63E-35
PIK3CB	0.29206809	8.67E-35
SUPT16H	0.42354614	8.98E-35
CAMKK2	0.28936824	1.19E-34
SAMD9	0.2436484	1.20E-34
SDCBP	-0.4361988	1.21E-34
FAHD2A	0.17371829	1.21E-34
GRN	0.42787836	1.26E-34
WDR1	-0.4055254	1.30E-34

CFAP36	0.49193838	1.33E-34
MAT2B	0.30157585	1.33E-34
TNFRSF14	0.26873099	1.44E-34
CD2BP2	-0.2686509	1.49E-34
ALDH3B1	0.2377579	1.54E-34
SPINT2	-0.434893	1.59E-34
FIRRE	0.24985275	1.76E-34
EVA1B	-0.533964	1.77E-34
SMIM26	0.44019779	1.80E-34
MORF4L2	-0.3918882	1.81E-34
MIR22HG	-0.3768916	1.87E-34
APEX1	0.44178425	2.04E-34
SMIM19	0.26275469	2.08E-34
TMUB1	-0.2367677	2.57E-34
EIF2S3	0.45918726	2.58E-34
PARN	0.18933497	2.80E-34
TPCN1	0.19840403	2.80E-34
PPP4C	-0.2610316	3.18E-34
MAP1S	-0.3603957	3.40E-34
SMAD3	-0.4106882	3.59E-34
CYHR1	0.1453335	3.61E-34
VPS36	0.39832443	3.81E-34
SSH2	-0.3186235	4.07E-34
GOLGA8M	0.1241868	4.09E-34
RUNX3	-0.6649249	4.14E-34
C1orf162	0.44215448	4.16E-34
THYN1	0.24227594	4.48E-34
RPL36	0.27760395	4.80E-34
FKBP1A	-0.401556	5.09E-34
TREML2	-0.2266026	5.10E-34
THUMPD1	0.43378318	5.34E-34
VPS53	0.19126208	5.34E-34
TCF7L2	-0.421613	5.42E-34
BHLHE40	-0.5708044	5.71E-34
MTMR6	-0.3454016	5.91E-34
TBCB	-0.2628773	6.02E-34
WASIR1	0.14716291	6.07E-34
CLPTM1L	0.21718809	6.07E-34
METTL7A	0.2691589	6.41E-34
FAM216A	0.25248891	6.51E-34
ZNF493	0.35133005	6.52E-34
EIF2AK1	0.38082218	6.90E-34
MRPL15	-0.3093799	7.03E-34
NFKBID	-0.4253665	7.73E-34
MGA	0.34338199	8.32E-34
Z83843.1	0.3125532	9.56E-34
PSMA6	-0.4205256	1.01E-33
NPIPB4	0.18598702	1.11E-33
PAQR8	-0.3492511	1.15E-33
MSI2	0.62573365	1.16E-33

CEP63	0.37416927	1.35E-33
CSK	-0.3032555	1.41E-33
SBDS	-0.4051082	1.42E-33
VPS26A	-0.2822762	1.50E-33
QSOX1	0.24270244	1.52E-33
DBP	0.18345905	1.55E-33
CCDC144A	0.31530889	1.61E-33
KANSL1L	0.22063195	1.79E-33
KCNN4	-0.3887418	1.88E-33
APBB1IP	0.37897039	1.99E-33
AC090559.1	0.43237744	2.05E-33
U2SURP	0.4266526	2.18E-33
DHX40	0.21314231	2.23E-33
NHLRC2	0.31662362	2.40E-33
H6PD	0.17616428	2.40E-33
RNF7	-0.4078129	2.55E-33
GSEC	0.16318806	2.78E-33
SLC25A19	0.18721822	2.86E-33
DDX55	0.23412796	2.95E-33
STXBP4	0.18601866	2.97E-33
NFKBIA	-0.9491557	3.24E-33
RNF44	0.22944686	3.25E-33
GUK1	-0.3377141	3.91E-33
TACC3	0.24275695	4.00E-33
EMID1	0.20404658	4.12E-33
CDK9	-0.3380903	4.20E-33
AL450998.2	0.21274402	4.54E-33
GRK3	0.21586572	4.64E-33
SMAD5	0.27293334	4.67E-33
RGS19	-0.4948077	6.06E-33
UBE2A	-0.3286283	6.13E-33
DDX46	0.42422281	6.44E-33
SMARCAD1	0.25115275	7.14E-33
SRM	0.40466855	7.27E-33
TCAIM	0.24371247	7.30E-33
COPRS	0.1888101	7.48E-33
GLUL	-0.6454601	7.50E-33
SLC39A11	0.16720177	7.60E-33
TSSC4	-0.2049183	8.96E-33
TMEM251	0.16903598	9.85E-33
PAFAH1B2	-0.3253229	1.01E-32
CCDC12	-0.3762214	1.08E-32
NMI	0.36006278	1.12E-32
ATP2B4	0.26504365	1.13E-32
ZNF138	0.2235286	1.22E-32
ZRANB2	0.48473157	1.24E-32
USP4	-0.2833463	1.28E-32
TRAF5	-0.4160838	1.41E-32
SLC25A46	0.24268075	1.55E-32
OSER1	-0.241347	1.56E-32

OTUD6B-AS	0.30045076	1.62E-32
PTPN6	-0.4976453	1.64E-32
METAP2	0.4152693	1.82E-32
GTF2H2C	0.17766929	1.92E-32
ITGB1	0.25865716	1.94E-32
PIGF	0.22481742	1.95E-32
HP1BP3	0.41095963	2.03E-32
ARF1	-0.452891	2.08E-32
SPAG9	-0.4298882	2.20E-32
CD58	-0.4388751	2.20E-32
ARHGAP30	-0.411339	2.33E-32
RAB5A	-0.3548939	2.34E-32
MLF2	-0.3107131	2.78E-32
DNTTIP1	-0.2466679	3.04E-32
MRPL3	0.35253679	3.25E-32
XAF1	0.35361211	3.31E-32
PSMG1	0.15161963	3.61E-32
NMNAT3	0.16788638	3.72E-32
TTC14	0.38175541	3.72E-32
DOCK10	0.26132662	3.81E-32
DGKD	-0.3417781	3.87E-32
BLOC1S2	-0.2817986	4.05E-32
GNL3L	0.23122531	4.22E-32
ATIC	0.28483086	4.27E-32
MRPS31	0.29786647	4.40E-32
NXT2	0.23573053	4.56E-32
ARID3B	-0.3370941	5.06E-32
CNBP	0.38799433	5.15E-32
ATG101	-0.2778418	5.20E-32
ANO6	0.2009318	5.30E-32
PYURF	0.35992703	5.94E-32
ACTR1B	-0.3327144	7.02E-32
C11orf68	-0.2171116	7.65E-32
SCAND1	-0.3540333	7.79E-32
DDX1	0.30188381	8.57E-32
ARL11	0.25359929	8.67E-32
MYCBP2	0.4601664	9.00E-32
CENPX	0.19990258	9.25E-32
M6PR	0.37823952	9.30E-32
ADK	0.35750719	9.67E-32
KRT10	0.3390173	9.85E-32
SLC30A9	0.21905017	9.97E-32
TBC1D5	0.33563182	1.04E-31
NUDT4	0.18286428	1.12E-31
GRK6	-0.268668	1.12E-31
SELENOK	-0.2646196	1.14E-31
ALG3	0.13714574	1.22E-31
PPIB	-0.4526098	1.24E-31
FLYWCH2	0.16169244	1.27E-31
KRIT1	0.14906725	1.31E-31

SOD2	-0.5318999	1.39E-31
TTF2	0.20715632	1.40E-31
NSD1	0.26699321	1.43E-31
PET117	0.15099008	1.45E-31
TRIB1	-0.3645136	1.45E-31
SLC39A4	0.22104948	1.47E-31
TMEM80	0.16714081	1.50E-31
ATP6V0A2	0.3868928	1.50E-31
DNAJB11	-0.3075516	1.53E-31
TMA7	0.33995114	1.55E-31
SIGIRR	-0.3009514	1.62E-31
RAB14	-0.398415	1.63E-31
UBE2J1	-0.4419185	1.64E-31
RUVBL1	0.25413241	1.64E-31
GNG7	0.1802829	1.93E-31
CPSF2	0.27029075	1.95E-31
FAM228B	0.2126751	2.00E-31
ENTPD1-AS	0.16133921	2.04E-31
ARPC1A	-0.2686188	2.26E-31
COX15	0.13590214	2.42E-31
RRP9	0.14539807	2.51E-31
OCEL1	0.1871114	2.53E-31
SH3BP2	-0.4741738	2.67E-31
FAM210B	0.25770258	3.11E-31
GPATCH11	-0.4148099	3.28E-31
ATRN	0.16226595	3.73E-31
SLC43A3	-0.3552513	4.19E-31
TMCO1	0.39910732	4.23E-31
NRROS	0.06516747	4.48E-31
COMMD9	0.233016	4.48E-31
MRPL54	-0.2545425	4.60E-31
GLB1	0.1598275	4.61E-31
SYNE3	-0.4356416	4.72E-31
TMPO	0.30735745	4.81E-31
FHL1	-0.2860961	4.92E-31
IL12A-AS1	0.31823052	4.96E-31
CTNNB1	-0.4820023	4.99E-31
ZBTB16	0.44106187	5.20E-31
DEAF1	0.22445211	5.26E-31
HSD17B12	0.32218421	5.32E-31
SMIM8	0.24056646	5.36E-31
COA6	0.25654162	5.78E-31
THOC3	0.21734721	6.02E-31
ZNF480	0.22569096	6.35E-31
RNF11	-0.3204718	6.37E-31
ZMPSTE24	0.14418485	6.99E-31
CETN3	0.15066779	7.04E-31
CD53	-0.4659003	7.37E-31
VASP	-0.5096073	7.88E-31
RSAD1	0.14505582	8.15E-31

CARNMT1	0.23130069	8.21E-31
ASRGL1	0.22102529	8.32E-31
LY75	0.37342636	8.90E-31
N4BP2	-0.4326062	9.15E-31
SEH1L	0.2102059	9.35E-31
HCG11	0.21499849	9.39E-31
MCEE	0.14167312	1.03E-30
TARSL2	0.24877616	1.05E-30
GUCY1A1	0.45198963	1.12E-30
ANXA6	-0.2981276	1.15E-30
LZIC	0.19878461	1.17E-30
MAPRE1	-0.2623923	1.18E-30
OSBPL1A	0.22089058	1.20E-30
TES	-0.5489501	1.24E-30
SNRPE	0.38544699	1.25E-30
MRPS5	0.27555693	1.28E-30
TMEM192	0.25169203	1.32E-30
ARF3	-0.2863478	1.46E-30
MPV17L	0.15676584	1.55E-30
CBR1	0.23466982	1.60E-30
NPIPB5	0.20038006	1.61E-30
DCP1A	-0.3339461	1.63E-30
ELP4	0.13963194	1.70E-30
LSM1	-0.1958749	1.72E-30
PCID2	0.23525844	1.78E-30
RPS15A	0.25876722	1.80E-30
EHD1	-0.4275444	1.86E-30
SATB1-AS1	0.28222589	1.94E-30
ORMDL2	-0.3014493	1.94E-30
CSTB	-0.364303	2.07E-30
AC092747.4	0.24717893	2.25E-30
ZDHHC21	0.2782662	2.26E-30
LINC01355	0.17459916	2.42E-30
LINC01252	0.14679125	2.68E-30
TRIM8	-0.390283	2.69E-30
NKG7	-0.4439204	2.74E-30
TOX4	-0.3028705	2.78E-30
RIPK2	-0.3962663	2.81E-30
STX17	0.26275073	2.83E-30
C9orf139	0.39675624	2.88E-30
NUCB2	0.53232084	2.89E-30
PPT1	0.31112455	2.97E-30
CLEC12A	0.29753642	3.08E-30
CTNNBL1	0.20639487	3.33E-30
ENTR1	0.22062829	3.76E-30
HOXA6	0.18799357	3.85E-30
AGGF1	0.19596874	3.95E-30
SLC20A2	0.20277273	4.04E-30
LAIR1	0.43745806	4.21E-30
PTPN2	0.33432792	4.32E-30

GLUD1	-0.2667669	4.32E-30
DDX39A	0.19224445	4.52E-30
RHOT1	0.24020175	4.52E-30
EIF4A2	0.3733174	4.64E-30
POLR2J3	0.42301056	4.83E-30
EMD	-0.2671798	4.92E-30
NDUFV3	0.28708183	5.01E-30
MCM3AP-AS	0.1555578	5.28E-30
CEBPA	0.51440765	5.35E-30
NAA38	0.35147025	5.57E-30
RECQL	0.24499381	5.82E-30
KTN1	0.44885748	5.88E-30
TFEC	0.37373748	6.10E-30
MFSD14C	0.19485191	6.22E-30
SH3YL1	0.18118204	6.25E-30
QKI	0.36420142	6.39E-30
IDH1	0.30466422	6.87E-30
C1GALT1C1	0.20013205	7.02E-30
INTS8	0.17678257	8.08E-30
TST	0.22796363	8.97E-30
SPG21	0.18030904	8.98E-30
TRIM28	0.17377228	9.47E-30
IRF2BPL	-0.3386974	9.67E-30
SPG11	0.28297474	9.80E-30
LINC00665	0.23542832	9.93E-30
PMS1	0.1960397	9.95E-30
SF3B5	-0.3513859	9.99E-30
MRPS9	0.15243872	1.02E-29
OARD1	0.21512321	1.08E-29
CMPK1	0.27840592	1.08E-29
PLEKHO2	-0.3747331	1.12E-29
SNCA	0.19982107	1.18E-29
MAPKAP1	0.19296056	1.24E-29
OAZ2	-0.3392456	1.25E-29
AC016831.5	-0.3582611	1.26E-29
ANKRD27	0.19999606	1.28E-29
JAML	-0.3330054	1.30E-29
ZNF638	0.38503587	1.30E-29
NFATC2	-0.3131696	1.35E-29
SERINC1	-0.3599892	1.36E-29
ATP6V0C	-0.4408289	1.37E-29
RABEPK	0.12554757	1.40E-29
FOPNL	0.19956506	1.44E-29
APOL1	0.11625287	1.46E-29
HEXD	0.18553887	1.55E-29
TSC22D4	0.08691065	1.59E-29
METAP1D	0.13729962	1.75E-29
BRD3	0.18965078	1.75E-29
PYCR2	0.16696151	1.90E-29
EIF4G3	0.22799937	1.92E-29

NSUN6	0.17557685	1.94E-29
CYB5R3	-0.2808014	1.97E-29
MAPK1IP1L	-0.3836732	2.00E-29
STAU2	0.15268189	2.42E-29
IFITM3	-0.4200258	2.43E-29
MIER1	-0.403308	2.47E-29
PACSIN2	-0.3346349	2.51E-29
AIMP1	0.36013001	2.52E-29
ATG14	0.12883993	2.56E-29
HNRNPA1	-0.3843186	2.57E-29
ZNF117	0.23606841	2.78E-29
PUS7L	0.28044103	2.79E-29
TBCK	0.20435905	2.85E-29
RTF2	-0.2879348	2.88E-29
NDUFA13	-0.3681043	3.03E-29
LLPH	0.29585822	3.07E-29
BMI1	0.29543308	3.14E-29
CORO1A	-0.4596609	3.15E-29
CRTAP	0.27069672	3.26E-29
PBDC1	-0.334966	3.45E-29
MPHOSPH8	0.36723779	3.68E-29
COG2	0.16887414	3.91E-29
EEF1AKNMT	0.17646236	3.93E-29
ZNF708	0.22669362	3.94E-29
GGNBP2	-0.3391014	4.12E-29
CHIC2	-0.2971546	4.66E-29
ATP5ME	0.39144272	4.67E-29
IMP3	0.36064104	4.68E-29
GPX4	0.3619734	4.69E-29
ZNF322	0.17681241	4.71E-29
CSF2RB	0.56270538	4.73E-29
CEP290	0.38259095	4.83E-29
ZCCHC7	0.2986809	4.84E-29
PRKAA1	-0.3965226	5.19E-29
ZNF487	-0.306268	5.60E-29
VPS72	0.11610762	5.66E-29
SAMD1	0.17398517	5.81E-29
GPR132	-0.2941709	5.92E-29
PTPN22	0.21909319	6.02E-29
DEDD2	-0.3523087	6.11E-29
MARCKSL1	-0.5381294	6.13E-29
SNHG30	0.18494092	6.41E-29
MTHFD1	0.14597722	6.42E-29
THOC1	0.24826101	6.71E-29
MCM3	0.07971219	6.79E-29
DGCR6L	0.12924523	6.88E-29
SELENOW	-0.3926889	6.97E-29
PIGN	0.16618978	7.05E-29
ERCC6L2	0.21369078	7.07E-29
ZBTB21	-0.3205534	7.35E-29

FAM174C	0.23694098	7.52E-29
MFSD6	-0.391697	7.54E-29
ACTL6A	0.19633983	7.68E-29
KANTR	0.18191493	7.82E-29
AC025040.1	0.15731482	9.33E-29
REXO4	0.25057285	9.49E-29
TLN1	0.3810722	9.61E-29
SCAMP1-AS	0.14106403	9.77E-29
MPC1	0.22661555	1.05E-28
CD83	-0.4175293	1.06E-28
AC104041.1	0.11628115	1.06E-28
GLMN	0.21205777	1.08E-28
AC068587.4	0.26410398	1.09E-28
IRAK3	0.41699248	1.29E-28
PTPMT1	0.23547308	1.35E-28
FADD	-0.2642832	1.37E-28
THOC7	0.37346427	1.39E-28
PGM2	0.20572925	1.42E-28
BCLAF1	0.35103277	1.45E-28
CCDC107	-0.2466851	1.51E-28
BCKDHB	0.153623	1.52E-28
ABHD10	0.08538558	1.56E-28
PRMT3	0.15774348	1.82E-28
MTR	0.29876936	1.84E-28
SCAMP1	0.18318156	1.88E-28
DNPH1	0.32288829	1.94E-28
IPO8	0.2151322	1.99E-28
NFE2L2	-0.3737442	2.07E-28
NCBP2	0.32502507	2.19E-28
FKBP2	0.15802719	2.23E-28
CEBPG	-0.3195527	2.31E-28
SEC22B	0.24527071	2.33E-28
NFYB	0.22481053	2.41E-28
C15orf61	0.10833529	2.46E-28
EIF3I	-0.3821379	2.46E-28
EIF3E	0.34227199	2.48E-28
MGRN1	-0.2171695	2.65E-28
SCARB1	0.16932413	2.67E-28
KCNAB2	0.338701	2.78E-28
FKBP4	0.24519543	2.80E-28
EGLN2	-0.2591941	2.81E-28
SRBD1	0.22649854	2.87E-28
PPWD1	0.27599366	2.89E-28
CARF	0.18242557	2.95E-28
ARL2	0.19072806	2.95E-28
DPYD	0.1729577	3.13E-28
MTX2	0.16923194	3.20E-28
ARHGEF6	0.30147505	3.26E-28
TOB2	-0.3607305	3.28E-28
AC019205.1	0.14846977	3.29E-28

STX12	-0.2596322	3.40E-28
TXNDC9	-0.1865364	3.48E-28
PPP1R16B	-0.2528423	3.55E-28
TAF12	0.23816561	3.61E-28
TCTN3	0.15782682	3.64E-28
GTPBP10	0.16650379	3.77E-28
SPTSSA	0.20526657	3.82E-28
PM20D2	0.21421826	3.83E-28
SRP9	0.27871436	3.91E-28
SRPRB	0.17183681	4.07E-28
ORC2	0.16459697	4.32E-28
IFITM2	-0.3781125	4.35E-28
NECAP2	-0.273872	4.38E-28
RCN2	0.20161119	4.45E-28
RNF144A	0.2021617	4.89E-28
RPL36AL	-0.3300227	4.92E-28
PGM2L1	-0.328158	5.05E-28
PBXIP1	-0.2851914	5.05E-28
ABRACL	0.43462913	5.09E-28
TNNI2	-0.281252	5.58E-28
AHI1	0.20874833	5.78E-28
CDC42SE1	-0.3502598	5.86E-28
BRD8	0.28987694	5.89E-28
NSUN2	0.07572752	6.29E-28
LINC00899	0.27363663	6.38E-28
FASTKD1	0.21697684	6.55E-28
CDK11A	0.16095939	6.72E-28
MGAT1	-0.3385451	6.78E-28
OSGIN2	-0.3469419	6.96E-28
BAHCC1	0.38849137	7.02E-28
COA3	0.22804776	7.04E-28
ARFGAP2	-0.222146	7.13E-28
ATE1	0.1760175	7.26E-28
FAH	0.2452088	7.63E-28
THOC2	0.37043755	7.72E-28
SLC3A2	-0.3386881	8.13E-28
CCT5	0.36536667	8.28E-28
CDYL	0.23139559	8.73E-28
EAPP	-0.3041257	8.74E-28
RNF19B	-0.3022346	8.80E-28
TRUB2	0.16192076	9.56E-28
PLEKHF1	-0.2850341	9.63E-28
GFI1	0.2596344	1.09E-27
HSPA1A	0.10280119	1.11E-27
SSBP3	0.264116	1.15E-27
ITGB3BP	0.23733206	1.17E-27
PIBF1	0.20315581	1.18E-27
FBXO9	0.2152357	1.21E-27
AL592183.1	0.16637926	1.23E-27
PDCD6IP	0.34813116	1.30E-27

ELL2	-0.3327745	1.34E-27
TMEM14C	0.37121397	1.35E-27
CDC42EP3	-0.4422119	1.37E-27
PMVK	0.16593916	1.41E-27
C5orf24	0.23989095	1.42E-27
HUWE1	0.26553819	1.43E-27
MIA3	0.27083139	1.50E-27
TMEM189	-0.2049936	1.50E-27
ERMP1	0.18996647	1.51E-27
PPCDC	-0.2837469	1.65E-27
INTS10	0.21874849	1.65E-27
IAH1	-0.2713603	1.68E-27
WDR36	0.1676739	1.71E-27
MAPK1	-0.3534432	1.72E-27
POLR2F	0.26728655	1.75E-27
NUDCD2	0.24461899	1.75E-27
TRPS1	-0.3604156	1.85E-27
USP48	0.32889516	1.89E-27
FAM214B	-0.304161	2.01E-27
HLA-E	-0.4377006	2.02E-27
DUSP23	0.24555851	2.10E-27
CEP41	0.17662495	2.18E-27
SRSF1	0.30814129	2.40E-27
CALM3	-0.2679272	2.52E-27
SLC16A3	-0.3943807	2.67E-27
ASPSCR1	0.12370157	2.69E-27
LRIF1	0.24115076	2.69E-27
STK40	-0.30092	2.74E-27
NBPF19	0.19616831	2.75E-27
PAFAH1B1	-0.3169558	2.89E-27
CWC25	-0.2816155	2.93E-27
TTC37	0.33117218	2.99E-27
IFT27	0.13174274	3.14E-27
RNFT1	0.16416411	3.37E-27
EPRS	0.30558435	3.44E-27
ZMYM3	0.12467729	3.61E-27
DNAJA2	-0.3294618	3.62E-27
CHD2	-0.5046702	3.65E-27
FOSL1	-0.2468022	3.81E-27
PRKAG2-AS	0.15615789	3.94E-27
ACO1	0.14564138	3.94E-27
STAG3	0.12707878	3.96E-27
SLC33A1	0.16740341	3.97E-27
HIKESHI	0.24979082	4.03E-27
CDK2AP2	-0.0869448	4.20E-27
RPL28	-0.2694285	4.38E-27
EHMT1	0.24512621	4.42E-27
MFSD14A	-0.2252297	4.53E-27
ZNF121	0.19089171	4.57E-27
MCM7	-0.0438841	4.57E-27

UBE2L3	-0.3608081	4.63E-27
IRF5	0.15327948	4.68E-27
DPY19L4	0.22222626	4.91E-27
WASHC5	0.14447611	4.93E-27
ADGRA3	0.11514962	5.08E-27
CDS2	-0.2838769	5.17E-27
ZNF397	0.22260458	5.43E-27
PHKB	0.28404035	5.43E-27
ZNF780B	0.16959589	5.44E-27
ZNF622	-0.1918225	5.45E-27
RELT	-0.3372461	5.45E-27
DCXR	0.27401963	5.59E-27
NFKB1	-0.2933622	5.64E-27
FBRS	-0.2079029	5.68E-27
OCIAD1	0.35641981	5.75E-27
AKAP8L	-0.2621665	5.82E-27
RBM27	-0.3165796	5.86E-27
RALY	-0.2685076	5.89E-27
ZNF253	0.18342556	5.91E-27
HENMT1	0.19436105	5.98E-27
ZEB2	0.53770624	6.00E-27
STARD3NL	-0.2106993	6.60E-27
AC005261.1	0.16992995	6.70E-27
GFOD1	-0.422092	6.84E-27
GNS	0.17794184	7.01E-27
STXBP5	0.37395612	7.09E-27
ZNF718	0.14008174	7.39E-27
MAPRE2	-0.3341586	7.54E-27
CDK4	0.17582915	7.59E-27
IER2	-0.6213645	7.68E-27
NRG4	0.27886337	8.23E-27
BAIAP2-DT	-0.2246948	8.23E-27
DHPS	0.17023419	8.61E-27
PLXNC1	0.27741057	8.93E-27
C22orf34	0.13261186	9.66E-27
EXOSC2	0.12570261	9.78E-27
ATR	0.3387435	9.84E-27
STIM2	-0.2393211	9.92E-27
URM1	-0.158088	1.04E-26
TM9SF2	0.26629028	1.10E-26
LDHB	0.35677221	1.11E-26
ITPR2	0.33291618	1.19E-26
RHEB	-0.2653882	1.20E-26
RAB32	0.5259165	1.23E-26
MINCR	0.18264609	1.23E-26
PSMB8-AS1	0.15221293	1.23E-26
TM7SF3	0.44419647	1.26E-26
UQCC2	0.20120612	1.27E-26
SKA2	0.20592345	1.31E-26
MCOLN1	0.12429653	1.40E-26

ADRM1	-0.2021471	1.41E-26
HIBADH	0.16765498	1.43E-26
NSRP1	0.38325866	1.44E-26
CDK19	0.18572173	1.44E-26
DOK2	-0.3818436	1.48E-26
TMEM11	-0.1337068	1.49E-26
GADD45B	-0.4481215	1.53E-26
mars-02	0.09868124	1.53E-26
ACADVL	-0.2313394	1.59E-26
CAMLG	0.45372283	1.61E-26
ACOT13	0.21564325	1.67E-26
DIMT1	0.20998891	1.68E-26
ACOX1	0.12934168	1.70E-26
LNPEP	0.30536971	1.74E-26
NDUFB6	0.28290419	1.76E-26
IGFLR1	0.2344407	1.80E-26
PCMT1	0.28610306	1.83E-26
HDAC3	0.01084095	1.91E-26
SYNC	0.18989854	1.91E-26
PSMF1	-0.1502123	1.94E-26
ZNF511	-0.1442019	1.97E-26
GART	0.26831953	2.01E-26
HELB	0.22577874	2.02E-26
ZNF248	0.16826501	2.06E-26
EFTUD2	0.10881153	2.09E-26
XPOT	0.27764714	2.17E-26
FAM83G	-0.2137313	2.19E-26
PIN1	-0.1438636	2.22E-26
NCKAP1L	0.31236549	2.47E-26
PNMA1	-0.3009372	2.63E-26
SELENOF	0.26461864	2.65E-26
FKBP8	-0.3110841	2.66E-26
AIP	0.01556304	2.69E-26
PIGP	0.2297636	2.74E-26
SAMD4B	-0.21835	2.89E-26
LCOR	-0.2846748	2.91E-26
NBPF14	0.15223684	2.91E-26
STAMBP	0.16853311	2.99E-26
GNA12	0.29506593	2.99E-26
ACTG1	-0.3614134	3.04E-26
RUFY1	0.24982127	3.13E-26
RPARP-AS1	0.28276702	3.17E-26
ATG7	0.15592883	3.19E-26
ZNF26	0.26382218	3.27E-26
NUDCD1	0.16708363	3.28E-26
RAB8B	-0.3674627	3.36E-26
DCTN2	-0.2007948	3.46E-26
SLC5A3	-0.4041046	3.47E-26
CBL	-0.261548	3.49E-26
R3HDM1	0.22839607	3.62E-26

SMIM27	0.27217718	3.63E-26
SH3BP5	-0.2903014	3.74E-26
TIMMDC1	0.23938903	3.77E-26
WASHC2C	0.21486022	3.82E-26
MTIF2	0.2397714	3.89E-26
RPS27L	0.38637934	3.91E-26
TIA1	0.3241025	3.93E-26
N4BP2L2	0.4338013	4.07E-26
NARS2	0.13060118	4.31E-26
TCP1	-0.2593767	4.34E-26
SNRPD3	0.30682948	4.51E-26
TTN	0.10563367	4.59E-26
FAM172A	0.14107829	4.62E-26
PGRMC2	0.21877663	4.72E-26
GRPEL1	0.1555833	4.98E-26
DPH5	0.13405421	5.12E-26
ZNF439	0.17737254	5.18E-26
PECAM1	0.29617178	5.21E-26
MTHFS	0.17693646	5.27E-26
PLEKHJ1	-0.1827706	5.48E-26
PAM16	0.22153709	5.52E-26
ZMYM1	0.13367611	5.78E-26
PMS2	0.18460576	6.04E-26
FOXO3	-0.2936881	6.34E-26
EIF4H	-0.3212471	6.35E-26
SF3B3	0.19084631	6.68E-26
TCAF1	0.21946675	6.80E-26
TFPI	-0.4659557	6.83E-26
MRPS33	0.34336986	6.88E-26
ZNF814	0.19749205	6.92E-26
ABCG1	-0.2948183	7.01E-26
TUBGCP4	0.18483218	7.13E-26
ALPK1	0.15165445	7.39E-26
PRMT2	-0.2807796	7.41E-26
TNIP2	-0.1871373	7.41E-26
PPIL3	0.09847601	7.55E-26
LDLR	-0.3360241	7.84E-26
BDH2	0.15939491	8.03E-26
ARL17A	0.1376122	8.03E-26
TAF1	0.15692332	8.23E-26
CHROMR	0.24612376	8.30E-26
FES	0.1655555	8.37E-26
GCA	0.19092103	9.04E-26
SMIM30	0.19124556	9.06E-26
SELENOT	-0.2868267	9.46E-26
ARF4	-0.3444468	9.48E-26
TMEM245	0.20880135	9.49E-26
TINF2	-0.2506078	9.81E-26
IFNGR2	-0.1772552	1.04E-25
NPTN	0.09002395	1.06E-25

ERV3-1	0.24700038	1.06E-25
PRPF8	0.27501636	1.07E-25
SEC23IP	0.17087817	1.14E-25
ACYP2	0.15960254	1.18E-25
DERA	0.10257973	1.21E-25
IP6K2	-0.286738	1.32E-25
POLR3H	0.12876592	1.34E-25
CCM2	-0.3043813	1.35E-25
FAM207A	0.08412288	1.45E-25
LAP3	0.14464657	1.51E-25
PHF7	0.17599582	1.54E-25
UGGT1	0.17261267	1.58E-25
NDUFAF8	0.26103755	1.69E-25
LNPK	0.16503997	1.71E-25
AGTRAP	0.20011487	1.72E-25
NOTCH2	0.19161022	1.74E-25
MTOR	0.09489735	1.76E-25
ARL6IP6	0.06712474	1.78E-25
COA1	0.20084513	1.79E-25
ATF1	0.12992406	1.83E-25
IFRD2	0.15938805	1.85E-25
ERLIN1	0.25924646	1.85E-25
MCM3AP	0.19802909	1.91E-25
PHACTR2	0.26666271	1.92E-25
UTP18	0.17274651	1.92E-25
NEDD9	-0.3941597	1.92E-25
ODC1	-0.301421	2.04E-25
ANAPC13	0.16488874	2.06E-25
WIPI2	-0.2163534	2.15E-25
SCP2	0.3351521	2.22E-25
EIF4EBP1	0.01531633	2.26E-25
JOSD1	-0.2269994	2.29E-25
EBNA1BP2	0.22047883	2.30E-25
PIGX	0.19719209	2.33E-25
ZEB1	-0.3817869	2.36E-25
LINC00667	0.16971881	2.36E-25
EBAG9	0.15059281	2.40E-25
C6orf226	-0.2662763	2.58E-25
PRDM16	0.23250818	2.59E-25
TRMT13	0.22628158	2.67E-25
DHRSX	0.12430861	2.67E-25
ADAT2	0.14369239	2.84E-25
SNRNP40	0.1253528	2.97E-25
AC142472.1	0.15469071	3.04E-25
TMEM168	0.18127699	3.11E-25
ERAP1	0.15762097	3.13E-25
ABHD14A	0.12222193	3.15E-25
COX4I1	-0.363456	3.26E-25
MRPL57	-0.4090102	3.32E-25
POLR3GL	0.26333706	3.45E-25

LARP1	0.32405545	3.49E-25
NUDT16L1	-0.2869501	3.55E-25
DMTF1	-0.2256145	3.67E-25
GAS7	-0.2979246	3.71E-25
ZNF32	0.15299997	3.76E-25
TSGA10	0.18197664	3.81E-25
ARHGEF2	-0.27632	3.84E-25
GANAB	0.15896353	3.90E-25
ANXA2	-0.4344336	3.93E-25
NGDN	-0.028588	3.95E-25
SCNM1	-0.0339888	4.08E-25
ZNF83	0.16342699	4.22E-25
KIAA0100	0.20193736	4.30E-25
SNHG20	0.18181041	4.32E-25
MAN1A2	0.31339442	4.34E-25
CLCN7	0.20658343	4.50E-25
INPP5B	0.0507805	4.76E-25
EXOSC8	0.15515095	4.96E-25
RBIS	0.36694704	5.03E-25
ALAD	0.13590258	5.16E-25
C12orf45	0.30788435	5.30E-25
HBP1	-0.2811482	5.32E-25
SREBF1	-0.261431	5.35E-25
SMC6	0.19033394	5.37E-25
STRBP	0.2502546	5.45E-25
DHRS4	0.1235995	5.46E-25
MED10	-0.276387	5.49E-25
ILF3-DT	0.19046659	5.67E-25
SUCLG2	0.16763259	5.87E-25
RPL8	-0.3297323	5.96E-25
UBE3D	0.14585401	6.52E-25
HNRNPUL2	0.21588526	6.56E-25
RNF145	-0.2353054	6.56E-25
FBXW11	-0.183737	6.63E-25
NLN	0.16879102	6.64E-25
RTCA-AS1	0.25365158	6.69E-25
THAP5	0.22133055	6.89E-25
PNPLA8	-0.2704047	7.11E-25
LINC01278	0.14273525	7.12E-25
REPIN1	-0.1941257	7.20E-25
NDUFB3	0.27095477	7.21E-25
ARFGAP3	-0.3082867	7.23E-25
ADGRE2	-0.4104027	7.30E-25
LIAS	0.1530208	7.34E-25
MYL12A	-0.373	7.35E-25
TAF9	-0.4629003	7.36E-25
VPS33A	0.04203095	7.36E-25
USP6NL	-0.3712143	7.48E-25
MAPKAPK2	-0.2454257	7.59E-25
TEX30	-0.2725737	7.81E-25

CD68	-0.3591056	8.39E-25
FBXO25	0.16954278	8.40E-25
BLOC1S5	0.1902547	8.94E-25
CHCHD7	0.29873604	9.12E-25
TMEM208	-0.2280039	9.56E-25
PSMD7	-0.2778321	9.68E-25
PML	-0.1723306	9.74E-25
YPEL2	-0.2970527	9.77E-25
PIGS	0.13033935	1.02E-24
PDCD2	0.2624543	1.02E-24
FBXO11	-0.2437538	1.03E-24
YRDC	0.14010104	1.05E-24
SSR1	0.34309147	1.13E-24
HDAC2	0.28419358	1.17E-24
CDC123	-0.2573966	1.19E-24
EIF3B	0.23469343	1.20E-24
GSK3B	0.16983727	1.21E-24
HIST1H2BC	-0.349951	1.22E-24
PDCL3	0.17051241	1.23E-24
SNHG32	0.41293842	1.27E-24
ADD1	-0.3348678	1.29E-24
MKNK2	-0.3401718	1.30E-24
LINC01410	0.11419461	1.31E-24
ZC3H4	0.19137273	1.35E-24
PIGBOS1	0.20021163	1.38E-24
DIPK2A	0.13392104	1.42E-24
LARP4	0.16798762	1.45E-24
CBWD1	0.15037086	1.49E-24
IRF9	-0.2638109	1.50E-24
HIBCH	0.12610746	1.50E-24
INSR	0.15910853	1.55E-24
AC015813.1	0.17914111	1.55E-24
ARID3A	-0.3490397	1.63E-24
NDUFAF4	0.11723868	1.63E-24
MAN2B2	0.1453692	1.68E-24
TCOF1	0.21041009	1.73E-24
CTSC	0.41232767	1.73E-24
MNS1	-0.3101884	1.75E-24
ARRB2	-0.3330224	1.77E-24
AAMP	0.04005911	1.79E-24
STK11	0.09458564	1.85E-24
SRP14-AS1	0.1391694	1.88E-24
MSRB3	0.32726659	1.88E-24
SMIM20	0.14418381	1.90E-24
CPT1A	0.30238983	1.92E-24
DLST	-0.1556169	2.05E-24
FBL	0.33183045	2.09E-24
B3GALNT2	0.14940018	2.14E-24
BMS1	0.18071641	2.16E-24
NUDT7	0.10686133	2.21E-24

BRIX1	0.1523754	2.21E-24
ELP3	0.08645615	2.24E-24
ZMYND11	0.17820499	2.26E-24
PSMD9	-0.1152654	2.30E-24
MBD4	0.28898323	2.32E-24
TANK	-0.3103029	2.37E-24
CCDC91	0.19605256	2.52E-24
RNF20	0.19905199	2.54E-24
SS18L2	0.06647949	2.64E-24
CYLD	-0.3280029	2.65E-24
ATXN7L1	0.13689927	2.68E-24
FLT3	0.51331079	2.76E-24
IVD	0.20671867	2.77E-24
MRPL19	0.1922484	2.79E-24
AC004918.1	0.09214825	2.82E-24
PSMD11	-0.2210862	2.84E-24
ARID1B	0.43455957	2.85E-24
SNHG4	0.20714443	2.86E-24
SSBP4	-0.2217003	2.87E-24
SNRPC	-0.1058177	2.89E-24
CHMP6	-0.226852	3.04E-24
TRIM27	0.22184178	3.13E-24
CD46	0.34863864	3.25E-24
DUSP1	-0.5791203	3.38E-24
MRE11	0.25157657	3.43E-24
DHX37	0.16413676	3.51E-24
NAB2	-0.2536502	3.54E-24
LINC02245	0.12803876	3.57E-24
CUTC	0.09703205	3.64E-24
ICE2	0.19654157	3.74E-24
POLR2G	0.18083596	3.86E-24
DOK3	0.14555561	3.97E-24
CDC34	-0.1338445	4.10E-24
CEP44	0.14638693	4.13E-24
MKKS	0.17075523	4.14E-24
EWSR1	-0.3066314	4.15E-24
DYNC1LI1	-0.2687406	4.16E-24
ACBD3	-0.3406646	4.45E-24
TMEM41B	-0.179296	4.53E-24
TP53BP2	-0.2926484	4.73E-24
RNMT	-0.2861271	4.74E-24
ATP5PD	0.3695989	4.78E-24
MLH3	0.21202481	4.79E-24
SPAG7	-0.1800468	4.83E-24
ZNF514	0.11307103	4.85E-24
RPP40	0.10131002	4.85E-24
SIVA1	-0.1420813	4.87E-24
ABTB1	0.10526923	4.95E-24
TAS2R14	0.10286945	5.07E-24
TVP23B	0.08399509	5.11E-24

BLVRB	0.264667	5.23E-24
SH3KBP1	0.39044536	5.36E-24
LINC01089	0.08875565	5.43E-24
APMAP	0.12413722	5.59E-24
COQ8A	0.14648732	5.69E-24
CUL1	0.15341837	5.89E-24
SLC38A10	0.20520551	5.97E-24
ISG15	-0.3152387	6.12E-24
TOPBP1	0.2057664	6.22E-24
SLC36A4	0.17055423	6.29E-24
CHPT1	0.18466981	6.40E-24
RBM39	-0.4265455	6.61E-24
UBR2	0.09366971	6.67E-24
SULT1A1	0.15967091	6.69E-24
RAD51C	0.06263608	6.86E-24
GLYR1	0.24602762	7.04E-24
RANGRF	0.13752904	7.11E-24
SOS2	0.23323269	7.35E-24
DYNC1I2	0.28155723	7.35E-24
ZNHIT6	0.28673323	7.47E-24
ZNF609	0.22878251	7.77E-24
CCDC125	0.22984393	7.78E-24
EHBP1L1	-0.2963025	7.78E-24
NFKBIL1	0.0453498	7.79E-24
ARL17B	0.08963017	7.87E-24
TMUB2	-0.1779235	7.99E-24
TMEM150A	0.10524732	8.01E-24
SLC39A6	0.14910162	8.09E-24
UBA2	0.18035263	8.18E-24
ACADSB	0.14540235	8.22E-24
NSFL1C	-0.179474	8.29E-24
PRMT5	0.14684357	8.60E-24
MRPL1	0.18236073	8.67E-24
TRPV2	-0.1916616	9.10E-24
FOSL2	-0.4201222	9.33E-24
TMEM35B	0.19525855	9.61E-24
GBGT1	0.11877746	9.69E-24
PAXIP1-AS2	0.13705419	1.01E-23
UBE2M	0.18484366	1.02E-23
JMJD6	-0.1428812	1.05E-23
SKIDA1	0.19843522	1.06E-23
ANAPC5	0.37237488	1.07E-23
NR6A1	0.16688783	1.09E-23
CYB561D2	0.10267275	1.12E-23
FAM98B	0.11872694	1.15E-23
MCM5	-0.118994	1.21E-23
NDUFA3	0.35593124	1.22E-23
ANKRD10-IT	0.16817958	1.23E-23
CCDC18	0.1764958	1.29E-23
FAM160B1	-0.1474962	1.29E-23

VPS51	-0.0862983	1.30E-23
NFXL1	0.12522806	1.30E-23
AC010642.2	0.10699939	1.32E-23
AC090630.1	0.14901012	1.33E-23
MED30	0.03855029	1.36E-23
VWA5A	0.17234939	1.39E-23
ZNF69	0.14450457	1.39E-23
CMIP	0.20987721	1.44E-23
RABIF	-0.2277141	1.45E-23
SNRPA1	-0.2489403	1.49E-23
IDI1	-0.3469948	1.50E-23
TSPAN3	0.23544082	1.50E-23
MNAT1	0.11861773	1.56E-23
TP53I13	-0.1820718	1.57E-23
TMED3	0.17702439	1.60E-23
CAD	0.14745426	1.63E-23
CAMTA1	-0.2268994	1.66E-23
SGTA	-0.2603048	1.68E-23
RAB7B	0.19026113	1.68E-23
SURF6	0.16691227	1.72E-23
NHLRC3	0.1396925	1.79E-23
ZBTB8OS	0.13082022	1.81E-23
RPUSD3	0.14441151	1.86E-23
CHMP1A	-0.1644501	1.86E-23
CFAP20	-0.1913999	1.92E-23
KLF2	-0.4368644	2.05E-23
SUMO1	-0.2551113	2.06E-23
CNOT6	0.12146291	2.09E-23
MSH3	0.22601874	2.12E-23
AL157392.3	0.12504205	2.12E-23
RTN3	0.32316288	2.15E-23
HACD3	0.15395367	2.16E-23
PDE4DIP	0.18344377	2.18E-23
NFYC	0.0634133	2.19E-23
AC104530.1	0.18062539	2.24E-23
ARID4A	-0.3925024	2.29E-23
JKAMP	0.20555025	2.41E-23
SLC46A3	0.13185564	2.43E-23
CACYBP	0.28716456	2.44E-23
GLT8D1	0.11506356	2.67E-23
CNTRL	0.34565754	2.73E-23
DTYMK	0.12608947	2.75E-23
CCDC149	0.16434849	2.77E-23
LSM6	0.13528277	2.80E-23
RPS24	0.21629608	2.94E-23
PPP1R35	-0.2004975	2.96E-23
UBA1	0.12479116	3.01E-23
LINC00623	0.13771102	3.05E-23
UNG	0.06521848	3.07E-23
ERH	0.32007425	3.19E-23

GNPTG	0.20375574	3.25E-23
TESC	0.30871963	3.40E-23
SAR1A	-0.207081	3.42E-23
VPS11	0.10170898	3.47E-23
GTF2H3	0.14059003	3.48E-23
UBE2F	0.27160088	3.50E-23
GARS	-0.2637057	3.55E-23
PMF1	-0.2362399	3.55E-23
DHFR	0.01078737	3.61E-23
FBXL3	-0.2696633	3.67E-23
NOG	0.15392018	3.70E-23
METRNL	-0.2999763	3.74E-23
RPA3	-0.0068413	3.75E-23
UXS1	0.15426998	3.77E-23
CCDC14	0.25150092	3.89E-23
ITFG1	0.1125952	3.89E-23
GSAP	0.11285109	3.96E-23
DDA1	-0.1721253	4.00E-23
FARSA	0.28538465	4.01E-23
SLIRP	0.27204139	4.01E-23
ZXDC	0.1091913	4.10E-23
PDHA1	0.11892929	4.12E-23
C17orf67	0.18699435	4.16E-23
TIMP2	0.21218779	4.22E-23
SQOR	0.2766006	4.27E-23
RBL2	0.18738566	4.38E-23
CHCHD3	0.13226091	4.48E-23
HMGB2	-0.3950338	4.51E-23
SMIM29	-0.1533403	4.55E-23
PPIP5K2	0.3071251	4.57E-23
ZNF433-AS1	0.20660741	4.59E-23
SNRNP27	0.18592511	4.64E-23
AC007566.1	0.12118867	4.65E-23
ITGAX	-0.3127187	4.68E-23
NAPEPLD	0.16695622	4.92E-23
PRPF19	0.04083862	5.10E-23
FTH1	-0.4447276	5.11E-23
EEF1E1	0.18750385	5.24E-23
MEF2C	-0.4976114	5.27E-23
TUBA1C	-0.278717	5.43E-23
ITFG2	0.129853	5.46E-23
SESN1	-0.2510622	5.47E-23
ANAPC16	0.26410212	5.49E-23
SMAP2	-0.2923134	5.61E-23
OSGEP	-0.122676	5.75E-23
EEF1AKMT1	0.12580101	5.78E-23
LYPLA2	-0.1176565	5.84E-23
RBM4	-0.2752564	5.88E-23
API5	0.13188903	5.89E-23
UBA6	-0.2590357	5.95E-23

DPY30	0.14239334	6.02E-23
STK24	-0.3093684	6.08E-23
GNL1	-0.2514515	6.08E-23
HEBP2	-0.295338	6.22E-23
ARMC1	0.11019015	6.35E-23
CTDSPL2	-0.0708106	6.35E-23
AC009061.2	0.11592479	6.56E-23
PDCD5	0.29387515	6.74E-23
PLPBP	0.14421412	6.83E-23
PUS7	0.16774924	7.16E-23
LATS2	-0.3238896	7.29E-23
ALYREF	0.15176113	7.50E-23
PCNX4	0.34606713	7.66E-23
PKD2	0.21090979	7.69E-23
MRPL39	-0.1233388	7.72E-23
KDM5C	-0.0277981	7.75E-23
FAM32A	0.03197545	7.86E-23
ICAM1	-0.2673737	7.89E-23
N4BP1	-0.2688434	8.47E-23
CBWD2	0.14417675	8.66E-23
RBM6	0.1862261	8.66E-23
PSMC5	-0.0808497	8.69E-23
PRELID3B	-0.0876709	8.80E-23
HMGCL	0.05066793	8.80E-23
POLI	0.10329538	8.92E-23
QPRT	0.39918818	9.37E-23
PITHD1	-0.1157225	9.42E-23
GAB3	0.05454866	9.45E-23
MON1B	0.14177865	9.57E-23
BTN3A2	0.15335006	9.59E-23
FBH1	0.11463531	9.65E-23
DHX8	-0.0071585	9.68E-23
MRPS22	-0.2241185	9.72E-23
PAAF1	0.20065903	9.76E-23
TIMM17A	-0.2016424	9.78E-23
TSPO	-0.364696	9.81E-23
DNAJC10	0.35572545	9.93E-23
ARHGEF7	-0.3250664	9.98E-23
PIGK	0.14188171	1.01E-22
TMC6	-0.2899753	1.02E-22
PIK3C3	0.26134668	1.03E-22
MAP1LC3A	-0.2671704	1.03E-22
PDCD6	0.32214854	1.03E-22
TRAPPC3	0.15193314	1.04E-22
CPSF7	-0.1592396	1.06E-22
NISCH	0.10501497	1.15E-22
ERG28	0.02518303	1.16E-22
RMND1	0.15717406	1.17E-22
PRKCQ	0.09204326	1.17E-22
LRRC28	0.09024035	1.17E-22

AC026124.1	0.15127181	1.18E-22
RPP21	0.12624843	1.19E-22
CBX4	0.06504144	1.20E-22
PSMB7	-0.1521052	1.22E-22
RBPMS	-0.2858719	1.22E-22
FCF1	0.14026053	1.24E-22
FOS	-0.6056705	1.30E-22
TDRD3	0.15392838	1.37E-22
TIMM17B	-0.0104146	1.41E-22
PSMB2	-0.2195563	1.42E-22
KLHDC3	0.08094776	1.47E-22
BAZ2A	-0.2810468	1.51E-22
XRRA1	0.13717805	1.52E-22
SMARCC1	0.40646971	1.54E-22
ZNF276	-0.2431609	1.57E-22
CD244	0.12585747	1.59E-22
SAMM50	0.05059361	1.60E-22
B3GNTL1	0.16260994	1.60E-22
GCLC	0.16852522	1.63E-22
C1orf50	-0.1148676	1.64E-22
PPP1R10	0.19080654	1.64E-22
SHPRH	0.18281685	1.64E-22
RAB11A	-0.1646349	1.64E-22
FAM49B	-0.3003519	1.65E-22
KRT18	-0.4796737	1.65E-22
SPIDR	0.18527018	1.66E-22
POLR3A	0.11675252	1.79E-22
GLO1	0.19338996	1.79E-22
HILPDA	0.11265662	1.80E-22
APOOL	0.14543436	1.85E-22
ZNF790-AS1	0.12083673	1.88E-22
DPH7	0.12098976	2.00E-22
UBE2D3	-0.3533047	2.02E-22
PHF5A	0.06429132	2.04E-22
TMEM126B	0.21828264	2.05E-22
TPRKB	0.1410728	2.06E-22
DARS	0.31236279	2.07E-22
LASP1	-0.2322283	2.08E-22
NEU4	-0.24255	2.12E-22
ZC3H6	0.19888393	2.18E-22
EEF2K	0.07892121	2.19E-22
ZNF664	0.12741181	2.21E-22
WDR61	0.22741236	2.24E-22
TFAM	-0.3266154	2.27E-22
PRR11	0.1590856	2.28E-22
EMC2	0.15462711	2.28E-22
PLAUR	-0.3534428	2.28E-22
PCMTD1	0.25944285	2.32E-22
GNA13	-0.245019	2.33E-22
MACF1	0.433657	2.34E-22

MYBBP1A	0.12642333	2.36E-22
GMPR	0.20659203	2.40E-22
ROCK2	0.2813396	2.50E-22
ARSD	0.28188902	2.51E-22
CHID1	0.01405954	2.52E-22
F8A3	0.13114383	2.54E-22
DPM2	0.09843596	2.54E-22
KDM2A	-0.1783169	2.57E-22
NUP43	0.09690802	2.57E-22
NT5M	0.14913045	2.59E-22
AC006480.2	0.11877208	2.60E-22
YARS	0.03135156	2.66E-22
TGIF2	-0.2165386	2.66E-22
PYM1	-0.2244312	2.67E-22
RNF213	0.19672347	2.70E-22
PRKAG2	0.10020878	2.73E-22
IGFBP2	0.44646952	2.75E-22
NUP153	-0.0993949	2.79E-22
TRIP11	0.30615636	2.79E-22
TMEM218	0.21214722	2.82E-22
TUT7	0.19636384	2.86E-22
KPNA2	-0.1480509	2.90E-22
ZNF429	0.18019246	2.97E-22
FAM106A	0.06758413	3.07E-22
ST3GAL1	-0.2988476	3.10E-22
RUNX1	0.35930107	3.15E-22
ELOC	-0.1656151	3.22E-22
FUT10	0.05884309	3.23E-22
ECM1	-0.1923731	3.33E-22
RFLNB	0.39675266	3.33E-22
STAT2	0.21451538	3.34E-22
ZNFX1	-0.2808825	3.43E-22
LMO7	0.13219134	3.49E-22
ZNF518A	0.22660072	3.50E-22
PLBD2	0.07457198	3.57E-22
EMG1	-0.119765	3.62E-22
CIZ1	0.12379091	3.64E-22
RFX2	-0.2891605	3.70E-22
IMMP1L	0.1272376	3.71E-22
ZC3H7B	0.12535367	3.71E-22
ZNF714	0.1452296	3.84E-22
ZMYND19	0.10438777	3.96E-22
AFG3L2	0.18496512	4.03E-22
ZYX	-0.3193021	4.11E-22
RRAGA	-0.1567269	4.14E-22
CHST13	0.11960443	4.16E-22
GTF2H5	0.19559296	4.20E-22
G6PD	-0.2309914	4.27E-22
THAP6	0.10737114	4.27E-22
HNRNPAB	0.38088085	4.27E-22

HEATR3	0.10904095	4.32E-22
LRRC59	-0.1707486	4.34E-22
NOMO1	0.08410639	4.36E-22
ITGAE	0.2723992	4.39E-22
HMOX2	-0.1094048	4.41E-22
SDHAF2	-0.1918064	4.49E-22
SEC13	-0.0059498	4.51E-22
SMIM7	0.05361325	4.61E-22
CSF3R	0.43246885	4.66E-22
KDELR2	-0.1531186	4.68E-22
SDK2	0.08445554	4.86E-22
YIPF3	-0.1359299	4.93E-22
NUMB	-0.1891051	4.95E-22
TRMT10C	0.32275609	5.02E-22
XPC	0.18158191	5.06E-22
DOK1	-0.1719708	5.10E-22
LYSMD2	0.16553646	5.17E-22
TCF12	0.07675161	5.21E-22
DGLUCY	0.23174768	5.33E-22
ALDH6A1	0.14194795	5.35E-22
CMC1	0.17456702	5.62E-22
VEZT	-0.2343091	5.67E-22
ZNF529	0.11164469	5.79E-22
KCNMB3	0.10572743	6.28E-22
EXTL2	0.14518801	6.53E-22
HAUS1	0.13072606	6.66E-22
SARS	0.16715838	6.73E-22
CTSW	-0.3955029	6.73E-22
NDUFAF6	0.16363773	6.80E-22
MOSPD2	0.11187001	6.95E-22
SLC16A7	0.19034754	7.03E-22
TLR2	0.18386458	7.09E-22
GPR174	0.19808974	7.19E-22
MAP7	0.35448486	7.22E-22
CDCA7L	0.15825492	7.23E-22
MTA2	0.02025959	7.26E-22
NUP107	0.15476433	7.40E-22
CGGBP1	0.24575225	7.41E-22
HDAC7	-0.1769857	7.76E-22
F11R	-0.3078638	7.92E-22
HEBP1	0.19179433	8.06E-22
LIX1L	0.11883543	8.06E-22
MRPL47	-0.1925098	8.06E-22
NSMAF	0.14039582	8.12E-22
RAD17	0.15085537	8.35E-22
CNOT11	0.07368387	8.46E-22
ELMO1	-0.2630366	8.76E-22
BZW2	0.2959525	8.80E-22
LYAR	0.17323712	9.03E-22
TCEA2	-0.2559969	9.06E-22

HADH	0.13705956	9.19E-22
NAA50	-0.1782235	9.21E-22
MXI1	0.14665834	9.39E-22
CDKN1B	0.03753593	9.51E-22
DNAJC19	0.21682228	9.51E-22
MBD2	0.11276057	9.55E-22
SCAMP3	0.13664881	9.55E-22
RPP25L	0.11259469	9.68E-22
NARF	0.10082446	9.76E-22
CEP152	0.17276996	9.80E-22
TMEM41A	0.12682531	9.88E-22
ZC3H15	-0.3775468	9.93E-22
PFN1	-0.4545221	9.99E-22
XPNPEP3	0.09990353	1.05E-21
GLRX	-0.1301879	1.05E-21
ZNF652	0.15280221	1.09E-21
AP002748.4	0.07309481	1.09E-21
MFN1	0.09284482	1.11E-21
SHFL	-0.2206199	1.11E-21
NRAS	-0.2195358	1.11E-21
FMNL3	0.10054352	1.12E-21
TSEN2	0.12891419	1.12E-21
KIDINS220	0.13856229	1.14E-21
TRIM44	0.26576621	1.18E-21
GOSR1	-0.1639631	1.20E-21
ZNF785	0.17517207	1.20E-21
C2CD2	0.08682272	1.22E-21
HSPB11	0.10338493	1.27E-21
KAT7	0.10505045	1.32E-21
MRPL11	-0.0253093	1.34E-21
NDUFAF3	-0.0503098	1.34E-21
PSMA5	0.24680187	1.36E-21
ZNF181	0.12461986	1.37E-21
HYPK	0.09343755	1.37E-21
RASA4	0.11033725	1.37E-21
LTN1	0.18529515	1.38E-21
TMED10	-0.229669	1.38E-21
COL24A1	0.2436248	1.39E-21
KDM4A	0.20849547	1.39E-21
AL138756.1	0.13004712	1.39E-21
HOXB2	-0.3278537	1.41E-21
SLCO3A1	0.14196587	1.42E-21
GSPT2	0.16027079	1.43E-21
ING1	0.0379008	1.44E-21
ELMOD2	0.131175	1.45E-21
MRPL22	-0.1800743	1.52E-21
INTS6	-0.3701456	1.53E-21
MRPL50	0.11791001	1.54E-21
WDR41	0.00146593	1.54E-21
NUDCD3	0.12319491	1.55E-21

ALDH5A1	0.14315623	1.59E-21
ITGB1BP1	-0.1695358	1.59E-21
GPR108	0.06287469	1.61E-21
TTN-AS1	0.20146754	1.63E-21
NOC3L	0.24540415	1.65E-21
SMIM10L1	0.15460625	1.67E-21
SDHAF1	0.12854836	1.70E-21
DPPA4	0.29391763	1.71E-21
IFIH1	0.18851268	1.72E-21
ORC3	0.10214875	1.73E-21
PSMC4	-0.1356128	1.80E-21
ATG3	-0.2002368	1.81E-21
COG3	-0.2433897	1.83E-21
NAXE	0.12932352	1.88E-21
AL390728.6	0.16698824	1.91E-21
NDUFA8	0.13516337	1.91E-21
FUOM	-0.2207648	1.96E-21
UNC50	0.10438453	1.97E-21
DTX3L	0.14615295	1.98E-21
AHDC1	-0.3338153	1.99E-21
IRAK1	0.01596535	2.00E-21
DPF2	-0.0757249	2.03E-21
GLOD4	0.21100748	2.04E-21
UBAC2	0.05285023	2.12E-21
PPP1R12B	0.15848772	2.12E-21
CMBL	0.17788259	2.13E-21
C16orf58	0.05387277	2.14E-21
ACLY	0.1775366	2.15E-21
FRG1	0.33115543	2.16E-21
COMMD4	0.1287278	2.17E-21
UBALD2	-0.1932867	2.21E-21
FAN1	0.08207007	2.24E-21
ELAC2	0.07592457	2.24E-21
LINC01184	0.1373629	2.29E-21
KLHL18	-0.2523813	2.29E-21
ANKRD13D	-0.1610486	2.30E-21
HPRT1	-0.1557966	2.34E-21
ZNF224	0.03875749	2.36E-21
PPP1R21	0.14111741	2.38E-21
RNPEPL1	0.02928339	2.41E-21
C1orf174	0.0810125	2.43E-21
TMEM30A	0.11513957	2.46E-21
DCTPP1	0.0953315	2.50E-21
GPAT3	-0.3351894	2.56E-21
RAB2A	-0.3083416	2.59E-21
POLG2	0.10185933	2.68E-21
UBQLN1	-0.1941381	2.73E-21
THRA	0.05689324	2.78E-21
NME1	0.29096998	2.79E-21
BFAR	-0.1904729	2.84E-21

UMPS	0.02033302	2.86E-21
CYBRD1	0.11440444	2.86E-21
RBM8A	-0.3243903	2.88E-21
ZNRF2	0.09674686	2.93E-21
INTS11	-0.0392431	2.95E-21
GOLT1B	-0.0835071	2.99E-21
MRPS2	0.02681244	3.03E-21
METTL8	0.17034986	3.11E-21
NIPAL2	0.13852054	3.14E-21
AP3S1	-0.2002183	3.19E-21
AC097448.1	0.10492931	3.28E-21
TIMM9	0.2255444	3.30E-21
ERI2	0.12049093	3.30E-21
NEDD8	0.28045021	3.30E-21
CBWD5	0.15797631	3.34E-21
PPP4R1	-0.2305361	3.41E-21
ARRDC1	-0.0114279	3.46E-21
PPM1K	0.23890902	3.49E-21
MLEC	0.29132748	3.51E-21
RIDA	0.12700854	3.53E-21
RFC4	0.04620582	3.56E-21
LONP2	0.1630158	3.57E-21
RPGR	-0.3653573	3.57E-21
ADSS	0.1785548	3.57E-21
EEF1D	-0.282698	3.61E-21
SNTB1	0.12282211	3.62E-21
NCK1	-0.2059905	3.64E-21
TNRC6B	0.3222898	3.64E-21
FAM89B	-0.1104839	3.65E-21
SNRNP200	0.22263147	3.67E-21
ZNF37A	0.23428479	3.73E-21
GAS6-AS1	0.18784062	3.74E-21
TMEM248	-0.0048154	3.77E-21
GET1	-0.1444614	3.88E-21
TECR	0.03129803	3.89E-21
XXYLT1	0.10985448	3.92E-21
MCFD2	-0.2112815	3.99E-21
AC010883.3	0.13701424	3.99E-21
HMG20B	0.04207584	4.00E-21
NAGK	-0.0392446	4.03E-21
TRAPPC11	0.17198979	4.09E-21
EVI5	0.17728293	4.12E-21
GRAMD1A	-0.153612	4.19E-21
PHF6	0.20023225	4.21E-21
RNF13	0.149369	4.22E-21
GNL2	0.08325359	4.27E-21
ATP6V1B2	-0.1858346	4.28E-21
GIGYF2	0.11685194	4.37E-21
EIF4EBP2	0.17641023	4.39E-21
CEP57	0.19183547	4.46E-21

POLR2C	0.01284641	4.47E-21
ARPC5	0.22657403	4.52E-21
CEBPZOS	0.17154733	4.65E-21
TMEM209	0.12118368	4.67E-21
UHMK1	0.21363031	4.83E-21
AC004540.2	0.24587994	4.87E-21
LIG3	0.1323323	4.93E-21
PPP6R3	-0.2444663	4.96E-21
STX8	0.10673641	4.98E-21
PPP6R2	0.10185873	5.07E-21
MFSD3	0.10616554	5.20E-21
RCC2	0.1865898	5.33E-21
SUSD1	0.16000536	5.34E-21
SP1	0.1397442	5.37E-21
CD34	-0.2039321	5.44E-21
KDM1A	0.21111643	5.45E-21
SRRM1	0.32363521	5.49E-21
SENP7	0.16311934	5.50E-21
AC105285.1	0.12274403	5.57E-21
NOL12	0.01296683	5.71E-21
LIPT1	0.13841876	5.93E-21
OTUB1	0.02379627	5.94E-21
ORC4	0.08708927	5.97E-21
DTD2	0.12510848	6.05E-21
EPG5	0.09237027	6.07E-21
IFT122	0.0819314	6.11E-21
CTPS1	0.15341577	6.22E-21
P2RX4	0.11847495	6.27E-21
EXOC3	0.17612427	6.28E-21
YBX1	-0.3648213	6.28E-21
MAGOH	-0.1141486	6.31E-21
IARS	0.20448955	6.31E-21
SNHG9	0.16612749	6.39E-21
ATP6V1F	0.36460111	6.41E-21
ELP5	-0.1431722	6.43E-21
AUP1	-0.1441707	6.49E-21
GON7	0.1568704	6.53E-21
PLA2G12A	0.06221242	6.54E-21
C1D	-0.0309504	6.65E-21
TMEM97	0.11207123	6.76E-21
ALKBH3	0.08631312	6.77E-21
ACOT11	0.16504634	6.80E-21
TSPYL4	-0.0665622	7.09E-21
PLEKHM1	-0.1640074	7.14E-21
B3GALT6	0.09083608	7.17E-21
ADAL	0.13797741	7.51E-21
USF2	0.25059456	7.67E-21
SYNJ2BP	0.15552097	7.92E-21
RNF168	-0.3188537	8.01E-21
LRRC8C	-0.3685916	8.01E-21

UBE2G2	0.23440926	8.10E-21
ARV1	0.13249458	8.17E-21
ARHGAP15	0.18732727	8.19E-21
AC004951.1	0.1377092	8.33E-21
LAMP1	0.24405116	8.35E-21
NSD2	-0.0753468	8.61E-21
ERCC3	0.05530755	8.77E-21
ADIPOR1	-0.1043682	8.77E-21
DUS1L	0.14383909	8.88E-21
CZIB	0.10347435	8.92E-21
AP1G1	-0.2701343	9.09E-21
HRAS	-0.2144771	9.13E-21
HAX1	0.00447116	9.18E-21
TAB2	-0.0240807	9.20E-21
ZSWIM6	-0.2885376	9.24E-21
STARD7	0.17993987	9.26E-21
FER	0.13025483	9.46E-21
YES1	-0.1814721	9.49E-21
ARRDC5	0.09619235	9.52E-21
OSTM1	0.12698612	9.53E-21
DNAAF2	0.14660659	9.57E-21
RNF181	-0.1969818	9.70E-21
BAK1	-0.1759026	9.86E-21
AFTPH	-0.2048088	9.95E-21
JPX	0.39515667	1.02E-20
VPS8	0.08792867	1.03E-20
RC3H2	-0.2041722	1.04E-20
HMGA1	-0.4457159	1.04E-20
EAF1	-0.2124421	1.05E-20
MPHOSPH9	0.19942672	1.05E-20
EIF2B1	0.15676013	1.06E-20
CPTP	-0.1631902	1.09E-20
MRPL27	-0.1194402	1.11E-20
NFAT5	-0.1573219	1.12E-20
ZNF33A	0.22865223	1.13E-20
SAFB	0.18124113	1.15E-20
USP33	0.22108151	1.16E-20
ZNF587B	0.10484781	1.17E-20
ADH5	0.17768542	1.19E-20
BEND6	0.16333621	1.22E-20
ADD3	0.23234011	1.23E-20
COPS2	-0.2869119	1.25E-20
PWWP2B	-0.2154491	1.27E-20
KIF5B	-0.1945279	1.27E-20
RBX1	0.33899431	1.28E-20
TIMM44	0.05495218	1.32E-20
RNF5	0.15531839	1.32E-20
YLPM1	0.18663761	1.33E-20
AL513128.2	0.10842175	1.33E-20
GCN1	0.09291228	1.34E-20

HPCAL1	-0.1695912	1.34E-20
PLRG1	0.16144044	1.35E-20
TRABD	-0.1204821	1.37E-20
AC022087.1	0.07347173	1.39E-20
MRPL30	0.16872969	1.39E-20
EMC4	0.10495157	1.41E-20
SLC25A38	0.09716485	1.43E-20
CDC5L	-0.1180233	1.43E-20
LMAN2	-0.1771959	1.46E-20
C12orf66	0.10052442	1.49E-20
RNF169	0.12359737	1.50E-20
PPM1B	-0.1755773	1.51E-20
RRP7A	-0.0927551	1.52E-20
RNF216	0.06911827	1.54E-20
PPCS	0.34191732	1.57E-20
RNF125	-0.2762091	1.58E-20
EIF4E2	-0.0793507	1.59E-20
HDHD2	0.15553716	1.60E-20
PACC1	0.0778633	1.61E-20
AC099518.5	0.04294369	1.61E-20
PDIA4	0.11166322	1.69E-20
DOCK11	0.14641103	1.73E-20
CEP192	0.15817955	1.73E-20
CLPB	0.16181558	1.76E-20
ANKAR	0.08247169	1.76E-20
PIGC	0.16907665	1.82E-20
RNF114	-0.2080213	1.84E-20
FNDC3A	0.09103369	1.85E-20
FADS1	0.26989119	1.85E-20
EIF2B2	-0.2161235	1.85E-20
ARHGAP11A	0.1594309	1.87E-20
IMPACT	0.23188149	1.92E-20
CEP128	0.14083108	1.93E-20
ABT1	-0.1446481	1.94E-20
HS2ST1	0.12685352	1.96E-20
CXorf21	0.22868596	1.96E-20
APLP2	0.47636249	1.97E-20
SEPTIN9	-0.1658791	1.97E-20
UTP14A	0.2156935	2.10E-20
PHF23	-0.1666595	2.11E-20
PABPC4	0.28984793	2.11E-20
DIS3L	0.14647966	2.24E-20
NIF3L1	0.09177333	2.26E-20
RNF166	-0.2334611	2.26E-20
GATAD1	0.28961956	2.27E-20
MAGT1	-0.1622115	2.27E-20
WDR75	0.0851994	2.28E-20
MLLT1	0.16095847	2.29E-20
STX10	0.04288447	2.30E-20
RPRD1A	0.07207299	2.34E-20

TMEM242	-0.1229316	2.36E-20
RNF4	-0.1539846	2.37E-20
UBE2G1	-0.077867	2.39E-20
PAN3	0.09343541	2.40E-20
BAG6	-0.0330689	2.44E-20
B4GALT1	-0.1429815	2.45E-20
PRPF6	0.20536948	2.47E-20
MGMT	-0.1315551	2.53E-20
METTL15	0.11881907	2.57E-20
BCL7C	-0.1382373	2.57E-20
UBN2	0.20469717	2.57E-20
SLC39A8	0.14499467	2.59E-20
LIMD1	-0.1652297	2.60E-20
BCCIP	0.15202268	2.62E-20
KIF1BP	-0.228454	2.65E-20
PGP	0.14076518	2.76E-20
IFT52	0.09054578	2.78E-20
RABGAP1	0.19520819	2.82E-20
KIAA1143	0.1944679	2.85E-20
PLEKHA3	0.05396352	2.91E-20
SPATA13	-0.219856	2.95E-20
EMC7	-0.1762621	2.96E-20
CCNY	0.18378987	2.97E-20
RPAIN	0.19746944	2.97E-20
ICOSLG	-0.2141947	3.02E-20
L3HYPDH	0.08632219	3.14E-20
ALG6	0.08927206	3.16E-20
UBE2S	-0.2245021	3.16E-20
CALCOCO1	0.0158474	3.17E-20
BOLA2B	0.11482804	3.17E-20
ZNF844	0.09794288	3.18E-20
GGPS1	0.14598382	3.18E-20
VKORC1L1	0.06884409	3.24E-20
GMPS	0.00073859	3.26E-20
SNRPA	-0.1991579	3.28E-20
ISOC1	0.06169258	3.32E-20
DLG1	0.12496052	3.40E-20
PRPS2	0.08814131	3.40E-20
NUDT6	0.15183927	3.43E-20
ROBO3	-0.1929639	3.44E-20
THUMPD2	0.14948186	3.45E-20
BLMH	0.09091686	3.49E-20
DRAM2	0.10099667	3.53E-20
GALNT7	0.2712981	3.53E-20
SLC43A1	0.12343721	3.56E-20
KHNYN	-0.221171	3.58E-20
TIFA	0.10733612	3.61E-20
PFDN6	0.14455783	3.64E-20
VRK3	-0.040908	3.65E-20
TM9SF4	0.09137884	3.69E-20

HDAC8	0.19706074	3.71E-20
DCTN5	0.03969715	3.72E-20
SH3GL1	-0.1595951	3.81E-20
SRSF4	-0.2068775	3.81E-20
GPI	0.17030678	3.82E-20
RIN3	-0.2474251	3.82E-20
IVNS1ABP	-0.2752349	3.86E-20
AC138649.1	0.12893339	3.86E-20
ACAT1	0.16848092	3.89E-20
TMEM69	0.06517557	3.89E-20
PLAU	-0.3335107	3.96E-20
TXNDC17	-0.1467141	3.97E-20
CSPP1	-0.2682046	3.98E-20
SNRNP25	0.11178997	4.05E-20
ASPH	0.19228362	4.09E-20
CCL5	-0.5850506	4.15E-20
FAM13A	0.01779094	4.21E-20
FBXL20	0.1167149	4.22E-20
RPS19BP1	-0.1634733	4.27E-20
KANSL2	0.03549829	4.29E-20
POLR1B	0.10762169	4.30E-20
CEBPZ	0.24857512	4.36E-20
CACUL1	-0.2034311	4.42E-20
SYK	0.18955626	4.49E-20
GNB2	-0.1729874	4.50E-20
CCDC102B	0.14715344	4.53E-20
TBC1D10B	-0.1395407	4.54E-20
FERMT3	-0.2555725	4.57E-20
CYB5R4	0.17568622	4.60E-20
COMMD10	0.08629712	4.63E-20
UBASH3B	-0.2531059	4.65E-20
TRAPPC4	-0.1795738	4.70E-20
CAPN2	-0.2178505	4.88E-20
IDE	0.13940399	4.92E-20
COX18	0.1488021	4.95E-20
RPL27	0.18468795	4.95E-20
ZC3HAV1L	0.07505022	4.98E-20
STX1A	-0.1481038	5.00E-20
HCFC1R1	-0.0694191	5.06E-20
AL109811.2	0.11308917	5.10E-20
PTDSS1	0.14310961	5.25E-20
NIFK-AS1	0.11551835	5.26E-20
RNF113A	0.10860158	5.30E-20
ZNF43	0.22416433	5.31E-20
NOTCH3	-0.1786949	5.41E-20
PLEKHM3	0.13131791	5.54E-20
ZNF490	0.12010659	5.56E-20
METTL4	0.10386625	5.57E-20
SNX18	0.11935758	5.62E-20
WDR89	0.08719585	5.68E-20

LINC02256	0.27840931	5.71E-20
SSRP1	0.09845125	5.73E-20
OSGEPL1	0.12254511	5.79E-20
PSAP	0.1612718	5.81E-20
NCOA3	-0.3448999	5.86E-20
MAP3K4	0.1664489	5.93E-20
FKBP5	-0.1855132	5.99E-20
DCTD	0.0132194	5.99E-20
C1orf56	-0.1930371	6.00E-20
GPALPP1	0.19880997	6.14E-20
GDF11	0.19793582	6.14E-20
RBM4B	0.05625267	6.19E-20
TMEM259	-0.1421672	6.22E-20
UBAP1	-0.1994785	6.29E-20
MAGOHB	0.11411207	6.41E-20
RRP36	-0.0859247	6.43E-20
CD300LF	0.05391879	6.58E-20
MSH2	0.15343937	6.65E-20
GLI4	0.10884223	6.80E-20
SACM1L	0.0085069	6.83E-20
UBE2Z	-0.3211069	6.87E-20
PPP6C	-0.227053	6.88E-20
CLASP2	0.13635072	6.90E-20
DLGAP4	-0.2173727	7.03E-20
UBL7	0.09873721	7.03E-20
NDFIP1	-0.2756995	7.05E-20
CTNS	0.09434396	7.18E-20
NCOA1	0.16595854	7.21E-20
HMGN3	0.3001399	7.22E-20
KDM6A	-0.1310638	7.29E-20
THAP11	-0.1000756	7.34E-20
RRP15	0.18386455	7.42E-20
WDSUB1	0.15023219	7.43E-20
NELFA	-0.0388531	7.59E-20
CTSA	0.24891262	7.62E-20
ZNF146	0.16061534	7.64E-20
SGK3	0.29241955	7.64E-20
DERL2	-0.1740941	7.81E-20
RNF138	-0.1787193	7.90E-20
LIPA	0.05545403	7.96E-20
XBP1	-0.4362229	8.00E-20
SLC30A5	0.04223066	8.06E-20
VDAC1	0.34891984	8.06E-20
WDR13	-0.2082666	8.14E-20
AC079781.5	0.10614216	8.16E-20
THAP12	-0.1566267	8.24E-20
ZBTB46	-0.2067821	8.29E-20
RPUSD4	0.0777405	8.61E-20
SZRD1	-0.2217361	8.94E-20
MGAT2	0.04564773	8.95E-20

EMC6	-0.1098076	8.97E-20
TOR1AIP1	0.20511127	8.98E-20
STMN1	-0.1120732	9.07E-20
RAPGEF1	0.09582265	9.17E-20
PIH1D1	-0.0633832	9.17E-20
RPL7L1	0.22873404	9.27E-20
LAGE3	0.12334846	9.31E-20
SDF2L1	-0.0243498	9.55E-20
IL27RA	-0.3132657	1.00E-19
PSMD6	-0.2332477	1.00E-19
MRPL32	0.11140584	1.01E-19
HEATR1	0.13765573	1.05E-19
CLTB	-0.1084042	1.05E-19
PPP2R2A	-0.2469305	1.06E-19
CAPN1	-0.224428	1.06E-19
POLDIP3	-0.0874604	1.08E-19
POU2F2	-0.3314734	1.10E-19
RPF2	0.19131266	1.10E-19
POLR2E	-0.0893064	1.11E-19
LRIG2	0.06820264	1.11E-19
DTNBP1	0.123619	1.13E-19
EDC4	-0.0613526	1.15E-19
CLDND1	-0.3511057	1.17E-19
PREPL	0.15432542	1.17E-19
CD2AP	-0.3280308	1.18E-19
SPNS1	-0.01358	1.18E-19
MSL3	0.04861882	1.18E-19
TRIM38	0.24294274	1.19E-19
RNF214	0.14124584	1.20E-19
ANKRD49	0.12896172	1.20E-19
NOP10	-0.3217244	1.21E-19
SLC35C2	0.0867018	1.21E-19
FANCA	0.09188049	1.23E-19
SLC26A2	0.25904392	1.24E-19
C6orf89	0.03309752	1.27E-19
GORASP2	-0.1778622	1.28E-19
MAPKAPK5-	0.08612202	1.28E-19
ING4	0.14900458	1.31E-19
EIF3J-DT	0.1611829	1.32E-19
QARS	0.08622088	1.33E-19
ZNF169	0.06822564	1.33E-19
RUNX2	0.1562863	1.36E-19
RB1	0.19552192	1.37E-19
RPL29	-0.2830992	1.38E-19
GCFC2	0.1073461	1.39E-19
CDKN2AIPN	0.04866869	1.40E-19
N6AMT1	0.08691208	1.41E-19
DUSP10	-0.2029874	1.43E-19
MRPS28	0.10329993	1.44E-19
SLU7	-0.2153877	1.45E-19

RFNG	0.09774799	1.47E-19
TMEM128	0.07806876	1.48E-19
CSGALNAC⁻	-0.2181765	1.48E-19
CNOT2	-0.2477321	1.48E-19
NXT1	-0.0976961	1.48E-19
AP1AR	0.14055475	1.50E-19
AL096870.8	0.13923119	1.52E-19
DERL1	-0.2189093	1.55E-19
KYAT3	0.09313314	1.55E-19
ZNF675	0.10843986	1.56E-19
NPRL2	0.14854995	1.58E-19
MGST2	0.19346445	1.59E-19
NUDT9	0.07424199	1.60E-19
MPLKIP	0.17073491	1.61E-19
ZNF106	0.26623035	1.63E-19
PARG	0.06923013	1.65E-19
ZNF667	-0.2917871	1.65E-19
AC246817.1	0.12558239	1.65E-19
SIN3A	-0.1257667	1.65E-19
SLC22A16	0.10502461	1.67E-19
COA4	0.26179635	1.68E-19
MYNN	-0.0296254	1.69E-19
DCAF17	0.10989408	1.70E-19
ERP44	-0.211355	1.70E-19
USP11	0.08320235	1.74E-19
UVRAG	0.03599057	1.74E-19
RGL4	-0.2016449	1.77E-19
ERCC5	0.19927	1.81E-19
SETD5	-0.15903	1.82E-19
SNX17	-0.0638398	1.82E-19
DNAJC3	-0.2606061	1.83E-19
NUCKS1	0.31344637	1.86E-19
PLGLB2	0.10929548	1.89E-19
PTTG1IP	0.24283159	1.89E-19
ZNF573	0.07832628	1.90E-19
HSPH1	0.20982202	1.92E-19
TBC1D1	0.12347657	1.94E-19
AAMDC	0.12905892	1.94E-19
RPA2	0.2147492	1.96E-19
RIPOR1	-0.2548153	2.01E-19
MANEA	0.11493438	2.02E-19
MTMR12	-0.2538777	2.03E-19
PDE3B	0.16553587	2.05E-19
FUCA2	0.15216042	2.05E-19
IST1	-0.2096276	2.06E-19
SPRYD7	0.10681721	2.06E-19
TEX264	0.04501026	2.07E-19
L3MBTL2	0.08774243	2.09E-19
NBR2	0.09536598	2.11E-19
CEP78	0.16554762	2.11E-19

C20orf27	0.06875556	2.15E-19
GPR89A	0.08413944	2.20E-19
IBTK	0.15831592	2.21E-19
PIKFYVE	0.24833615	2.24E-19
UROD	0.06727977	2.24E-19
NRBP1	-0.1510029	2.26E-19
COQ5	0.06691472	2.26E-19
SERPINB9	-0.3004108	2.27E-19
NUP133	0.10329146	2.27E-19
PDAP1	0.16858438	2.30E-19
ALKBH4	0.05497265	2.30E-19
METAP1	0.12732339	2.33E-19
TRIO	0.03986631	2.34E-19
KIAA1109	0.13841044	2.36E-19
TRIAP1	0.06400342	2.37E-19
C11orf98	0.09474551	2.39E-19
CBFB	0.12331518	2.40E-19
U2AF2	0.11952567	2.40E-19
SDC4	-0.1783187	2.44E-19
KLF4	-0.1945662	2.47E-19
CBFA2T2	0.13054669	2.49E-19
PAXBP1	0.27018793	2.49E-19
PTK2B	-0.095395	2.51E-19
WDR48	-0.1437887	2.53E-19
AC018638.7	0.06888721	2.56E-19
CYTH2	-0.217852	2.60E-19
AK6	-0.0038332	2.63E-19
HECA	-0.1443647	2.65E-19
DCP2	0.16492233	2.68E-19
MRS2	0.10490012	2.68E-19
SP110	0.11084889	2.69E-19
DCPS	0.11814968	2.70E-19
BRMS1	-0.0042382	2.74E-19
METTL5	0.16501622	2.76E-19
B4GALT6	0.13477419	2.84E-19
ZNF24	-0.0610079	2.85E-19
KTI12	0.08245111	2.91E-19
MRFAP1L1	0.10368735	2.91E-19
DDOST	-0.1434527	2.91E-19
ZNF524	0.05703094	2.93E-19
MLX	-0.1097378	2.93E-19
TAMM41	0.0679023	2.98E-19
CCDC28A	0.03063118	3.00E-19
BCAS3	0.10071648	3.01E-19
RCN1	0.20263653	3.06E-19
RFXANK	0.04580164	3.07E-19
SPATA2L	-0.2028571	3.12E-19
SNUPN	0.18333078	3.16E-19
LZTS2	-0.0900868	3.19E-19
CWF19L2	0.19405039	3.19E-19

DOCK2	0.30688264	3.20E-19
FHL3	-0.2157501	3.20E-19
ZNF195	0.20251196	3.22E-19
TBP	0.06150874	3.22E-19
EHBP1	0.10281152	3.26E-19
LYPLAL1	0.11966817	3.29E-19
SCLT1	0.09112642	3.31E-19
SHOC2	-0.2466472	3.32E-19
CAPRIN1	0.28525418	3.37E-19
SERPING1	-0.239881	3.41E-19
IFT172	0.06857906	3.42E-19
DCAF12	0.08365335	3.42E-19
RAE1	-0.1594258	3.43E-19
AP3B1	0.16176656	3.45E-19
DYRK1B	-0.2193499	3.46E-19
HDAC6	0.10276217	3.47E-19
SLC35A3	0.10678424	3.48E-19
QSER1	0.09509181	3.50E-19
PGAM5	0.07510468	3.53E-19
PIP4K2B	0.12970693	3.54E-19
ZNF124	0.11171216	3.58E-19
RNF34	-0.1654844	3.58E-19
COPB1	-0.1859447	3.60E-19
TMEM14B	0.30084722	3.62E-19
RPIA	0.14289645	3.63E-19
SNHG1	0.18490934	3.63E-19
SSU72	0.24983521	3.65E-19
ANXA4	0.16059216	3.68E-19
TOMM6	0.30360752	3.71E-19
TRAPPC12	0.11707439	3.71E-19
SOCS7	0.07735641	3.75E-19
COMMD1	0.07230135	3.81E-19
XPO1	0.27801984	3.85E-19
NNT	0.17828552	3.86E-19
C21orf91	0.14248763	3.91E-19
ZNF512	0.13564456	3.92E-19
CDIPT	0.01303064	3.94E-19
HCFC1	0.07333687	3.98E-19
CCDC117	0.14096258	4.01E-19
S100A9	-0.3500587	4.07E-19
MICU1	0.13614424	4.10E-19
SH3TC1	-0.1966903	4.14E-19
FAF2	-0.1856381	4.32E-19
TRIM5	0.16124546	4.34E-19
RIT1	-0.2441739	4.35E-19
TMEM59	-0.3215659	4.38E-19
SDHB	0.03070258	4.39E-19
RBM26-AS1	0.10201362	4.47E-19
HIF1AN	0.02249214	4.48E-19
PDS5B	0.12309595	4.53E-19

STK32C	0.17950169	4.53E-19
MRPL14	0.07858781	4.58E-19
KAT8	0.07430856	4.62E-19
TOMM40	0.10545	4.66E-19
TUBGCP3	0.09868503	4.66E-19
JPT1	-0.1611854	4.71E-19
BUD23	-0.0034841	4.71E-19
ILF2	-0.3288369	4.75E-19
MATR3.1	0.1506435	4.79E-19
SMARCAL1	0.09138827	4.79E-19
DNM1L	0.01007693	4.82E-19
MTREX	0.08593793	4.84E-19
ZC3H7A	0.078956	4.86E-19
BX890604.2	0.26565553	4.94E-19
APIP	0.17037641	4.95E-19
GFM1	0.0901554	4.98E-19
RNF40	0.01190697	5.02E-19
AL356599.1	0.09872471	5.03E-19
ATMIN	0.07405872	5.03E-19
PCNT	0.1623192	5.05E-19
FBXL15	0.06072222	5.12E-19
IL16	0.1657568	5.22E-19
USP3	-0.1567693	5.35E-19
AC123768.4	0.06143831	5.47E-19
TNIP1	-0.2129506	5.48E-19
GPR146	-0.2935092	5.51E-19
SLC37A1	-0.0818648	5.52E-19
BLCAP	-0.1219317	5.55E-19
ZNF618	0.11767396	5.78E-19
DENND3	-0.2697113	5.84E-19
SETD1B	-0.0375152	5.89E-19
WDR26	-0.0665944	5.89E-19
NSMCE3	0.08192672	5.99E-19
SRF	-0.0503964	6.02E-19
IKBKB	0.05240234	6.05E-19
MAPK9	0.14540013	6.10E-19
ALG10B	0.1380086	6.14E-19
USP22	-0.176238	6.15E-19
NR1D2	0.15531255	6.16E-19
PPIH	0.03629628	6.22E-19
RTN4R	-0.2144186	6.27E-19
HIST1H2AC	-0.2183726	6.43E-19
SLC50A1	-0.2094851	6.45E-19
ERVK3-1	-0.1591942	6.52E-19
ALAS1	-0.2058132	6.55E-19
YIF1A	-0.0909108	6.57E-19
MTCH1	-0.2072283	6.57E-19
CTCF	-0.0489108	6.59E-19
DENND1B	0.20698423	6.60E-19
MIR17HG	0.08699515	6.62E-19

PJA2	-0.2899329	6.63E-19
WASHC4	0.19577786	6.70E-19
COIL	0.07891048	6.72E-19
ECPAS	0.11223373	6.86E-19
ST3GAL4	0.15307786	6.90E-19
TFCP2	0.14374164	6.90E-19
SDHAF4	0.11533283	6.95E-19
NINJ2	0.12931762	6.96E-19
UPF3A	0.26703306	6.97E-19
LENG8	-0.2070491	7.01E-19
CISD1	0.08355526	7.16E-19
VIRMA	0.003402	7.19E-19
AGPS	0.10275545	7.27E-19
MID1IP1	-0.2153277	7.28E-19
C2orf69	0.12606083	7.29E-19
ZFP14	0.15112484	7.31E-19
IDH2	-0.1571242	7.34E-19
GATC	0.12359485	7.36E-19
EIF1B-AS1	0.11826637	7.39E-19
NCF2	-0.2743797	7.40E-19
RAB4B	0.12651989	7.42E-19
LINC00205	0.12442833	7.52E-19
GNB4	-0.2084001	7.60E-19
FAM76B	0.10246948	7.65E-19
RC3H1	-0.244934	7.70E-19
CTSZ	-0.1770404	7.81E-19
GNPDA2	0.08842858	7.86E-19
VPS26C	-0.208907	8.06E-19
SNAP29	-0.1971523	8.08E-19
CHMP2B	-0.2291924	8.12E-19
COPE	-0.2854619	8.13E-19
DPM1	-0.1945483	8.14E-19
DTWD2	0.10134686	8.15E-19
TSHZ1	0.06837751	8.18E-19
AASDH	0.09472683	8.23E-19
ACTR10	-0.0929773	8.29E-19
SFT2D1	0.02113812	8.30E-19
ZNF727	0.16535761	8.33E-19
ZNF101	0.08616199	8.41E-19
AQR	0.15129065	8.41E-19
TCF15	0.09832779	8.42E-19
ARMCX6	-0.1670753	8.44E-19
IP6K1	-0.1952223	8.47E-19
NELFE	-0.0336041	8.47E-19
PPTC7	-0.1924132	8.51E-19
FAM192A	0.18675449	8.59E-19
TOR1AIP2	-0.043332	8.70E-19
KBTBD7	0.10740468	8.71E-19
SART1	-0.0065867	8.94E-19
MSRB1	-0.0205236	9.06E-19

OGA	-0.2360231	9.09E-19
TMED8	0.05241199	9.12E-19
RAB22A	-0.2585552	9.13E-19
TOR4A	-0.1606238	9.19E-19
TAPT1	-0.1970934	9.25E-19
ADAM15	-0.196911	9.47E-19
EML3	0.06622805	9.55E-19
SETD3	0.03432924	9.55E-19
NANS	-0.0984274	9.87E-19
SMAP1	0.13439844	1.02E-18
ACTR1A	-0.2352921	1.02E-18
ATP11A	0.09995623	1.02E-18
CLTC	-0.1307152	1.03E-18
PDCL	0.10859807	1.03E-18
MMACHC	0.09484415	1.03E-18
LRCH4	-0.1023716	1.04E-18
PFKFB3	-0.2948193	1.08E-18
NTHL1	-0.0389776	1.08E-18
ATP6V1D	-0.1550487	1.09E-18
NOL10	0.09062428	1.10E-18
PDLIM7	-0.0599799	1.12E-18
LIG4	0.13967389	1.13E-18
ZNF681	0.18631677	1.13E-18
ARPC4	-0.1464992	1.13E-18
RRBP1	-0.3109896	1.16E-18
NDUFAB1	0.1457379	1.18E-18
CSNK2A2	0.03150964	1.18E-18
CAST	-0.3503422	1.18E-18
RHOH	-0.2014369	1.19E-18
MRFAP1	-0.0716678	1.19E-18
SAFB2	0.10483492	1.20E-18
TWNK	0.13127717	1.20E-18
PLXNB2	-0.226373	1.22E-18
CHPF2	-0.125578	1.27E-18
KLHL24	0.27619794	1.28E-18
PHTF1	-0.1288862	1.28E-18
SHKBP1	-0.1668155	1.31E-18
UBE2R2	-0.2343177	1.32E-18
MYPOP	0.0780871	1.32E-18
TMEM43	-0.1774446	1.33E-18
PDXDC1	0.08261833	1.35E-18
GMFB	-0.1449101	1.35E-18
PELP1	0.10006352	1.40E-18
AGK	0.10260263	1.40E-18
ALCAM	0.24956189	1.42E-18
TAF2	0.05690013	1.42E-18
MYO9A	0.14542823	1.43E-18
VRK2	0.15124588	1.43E-18
LSG1	0.13612609	1.43E-18
CHST14	0.08358106	1.43E-18

NOSIP	0.0356124	1.44E-18
EIF3M	0.26339228	1.49E-18
MDM1	0.12901623	1.50E-18
CYSLTR1	0.16034598	1.50E-18
EPN1	-0.1944036	1.51E-18
TMEM273	-0.2502519	1.53E-18
TRPM7	0.16181895	1.54E-18
MMP24OS	-0.1330411	1.55E-18
NUP54	-0.1047896	1.56E-18
PPP1R15B	-0.1366577	1.56E-18
AC022098.1	0.07425246	1.58E-18
ITSN2	-0.3035324	1.59E-18
TSR3	-0.0865885	1.59E-18
TET1	0.08585043	1.59E-18
COLGALT1	0.09983203	1.60E-18
SLC25A40	0.10217929	1.61E-18
GPBP1	-0.1912368	1.61E-18
TRPM2	0.10807692	1.61E-18
GBP4	0.21663377	1.62E-18
RELA	-0.1856384	1.63E-18
IRF2BP1	-0.0079175	1.63E-18
AMPD3	0.08115745	1.65E-18
ATP10D	0.17330729	1.68E-18
DNAJC24	0.10725948	1.70E-18
DTWD1	0.23644085	1.72E-18
CDK11B	0.11944533	1.73E-18
LZTFL1	-0.0492977	1.74E-18
PDLIM4	-0.1929052	1.75E-18
FECH	0.10527672	1.76E-18
CCDC86	0.07681339	1.76E-18
TSN	0.12212923	1.76E-18
EEF1AKMT4	0.08983779	1.78E-18
RAB6A	-0.1751836	1.78E-18
C1GALT1	0.00354339	1.79E-18
PPM1A	-0.1342257	1.79E-18
ZNF84	0.18726461	1.80E-18
IPO7	0.20046541	1.81E-18
STAT6	0.0935229	1.81E-18
CPNE8	0.1622085	1.83E-18
KCTD20	0.09325532	1.83E-18
TXNIP	-0.4732926	1.84E-18
STK10	-0.1904172	1.87E-18
SPATC1L	0.138341	1.88E-18
NSUN3	0.10042173	1.91E-18
TIAL1	0.28261026	1.92E-18
CBFA2T3	-0.0739155	1.94E-18
PHF21A	0.09652227	1.94E-18
ETFRF1	-0.2189487	1.95E-18
MCTS1	0.20938742	1.99E-18
ZNF808	0.05881858	2.00E-18

AC009950.1	0.07661127	2.06E-18
TSNAX	0.19940843	2.06E-18
TUFM	-0.2031393	2.08E-18
SND1	0.02866712	2.09E-18
G2E3	0.09657967	2.11E-18
NUTF2	-0.1550117	2.12E-18
ABCF2	0.08989843	2.15E-18
FBXL12	-0.1647826	2.16E-18
SCRN3	0.08269712	2.18E-18
METTL23	0.0452888	2.18E-18
LMBRD2	0.10771343	2.19E-18
ATP23	0.12496751	2.23E-18
ACTN4	-0.2649568	2.24E-18
ZNF362	0.07688069	2.25E-18
NMRAL1	0.04703636	2.26E-18
SLC35E2B	0.04516109	2.27E-18
RABL2A	0.0889695	2.28E-18
MAD2L1	0.10628681	2.31E-18
C1orf43	-0.1097668	2.33E-18
MRPS27	0.08729384	2.33E-18
FDX2	-0.1511603	2.36E-18
CARD6	0.13568921	2.36E-18
RBM41	0.17926201	2.37E-18
ERI3	0.0563437	2.37E-18
CAPN7	0.13773492	2.37E-18
CDC37	-0.1429994	2.38E-18
SEMA4D	0.0566722	2.38E-18
VBP1	0.08556268	2.43E-18
DHODH	0.1020816	2.43E-18
GNAQ	0.29730427	2.44E-18
NUDC	-0.0955269	2.48E-18
DMXL1	0.15139065	2.49E-18
CASP1	0.03155451	2.50E-18
ANKRD40	-0.0523746	2.50E-18
TESK2	0.09894792	2.52E-18
PPP3CB-AS	0.13429269	2.52E-18
WASHC3	0.07687054	2.53E-18
SLC39A1	-0.0692199	2.58E-18
ALS2	0.20484348	2.64E-18
ZNF561	0.07453216	2.65E-18
YY1AP1	-0.2034082	2.66E-18
GDE1	0.03062698	2.69E-18
MTFR1	0.08069893	2.71E-18
DIP2A	0.10305133	2.72E-18
GTPBP4	-0.253799	2.75E-18
FADS2	0.08123571	2.77E-18
ATG12	0.22145592	2.79E-18
RRP1	0.06713597	2.81E-18
GDI2	0.34120762	2.83E-18
FXN	0.13106925	2.83E-18

AC106791.1	0.13193702	2.85E-18
TRMT10B	0.15061956	2.86E-18
AUH	0.07285387	2.87E-18
H2AFY2	0.09932133	2.90E-18
RAD23B	0.12999206	2.94E-18
MAP3K5	0.04183259	2.96E-18
DNAJC11	0.06200161	3.03E-18
MAPK12	0.11152621	3.07E-18
C1orf131	0.11894189	3.10E-18
MGST3	-0.2420339	3.13E-18
AUXG01000(	0.11626061	3.13E-18
ZNF280D	0.20292459	3.14E-18
GINM1	-0.2406146	3.15E-18
METTL2B	0.09769739	3.17E-18
LDLRAD4	0.15477765	3.18E-18
MTRF1	0.10098449	3.18E-18
MRPS10	0.07225329	3.19E-18
AC108519.1	0.10270129	3.22E-18
NDUFA5	0.11113131	3.22E-18
TMEM60	0.1196011	3.23E-18
PNKP	-0.1404021	3.26E-18
MIR4435-2H	-0.1835854	3.27E-18
FKTN	0.10977068	3.29E-18
BCKDHA	0.11921824	3.30E-18
CPSF4	0.06203357	3.31E-18
HACL1	0.09880224	3.31E-18
COPS5	0.1430862	3.34E-18
UCHL5	0.16099237	3.39E-18
NT5C3B	0.08111403	3.41E-18
NXPE3	0.11052167	3.46E-18
PAGR1	0.01536438	3.46E-18
PPP1CC	-0.0089989	3.46E-18
AC083798.2	0.07950013	3.48E-18
CTBP1	0.25869751	3.49E-18
CXorf56	0.029139	3.55E-18
MRPS30	0.1777208	3.67E-18
MPST	0.17646731	3.68E-18
NR2C1	0.01850097	3.68E-18
UTP6	0.19043051	3.70E-18
MRTO4	0.16525594	3.72E-18
AGFG1	0.07454774	3.73E-18
ZCCHC8	0.03519561	3.73E-18
AC093627.4	0.06698832	3.74E-18
MRPL44	-0.0176934	3.75E-18
C3	0.10275865	3.84E-18
GABARAPL2	0.21257421	3.90E-18
AL359504.2	0.08568601	3.91E-18
CCDC30	0.14192275	3.93E-18
MOSMO	0.10631819	3.94E-18
CLK1	-0.3031525	4.00E-18

CLN5	0.08480553	4.01E-18
C19orf25	-0.0335185	4.06E-18
RAB37	0.20342287	4.08E-18
FAM120AOS	-0.1064731	4.11E-18
POMGNT1	0.06184018	4.13E-18
DDX56	-0.0605702	4.18E-18
EXOC4	0.07621785	4.21E-18
TMEM165	0.13290902	4.24E-18
SURF1	-0.0001133	4.28E-18
STOML2	-0.0210417	4.29E-18
CALHM2	-0.2075263	4.31E-18
CCNK	-0.1305215	4.33E-18
CERK	-0.1180696	4.34E-18
ELP1	0.0916707	4.35E-18
CHD4	0.32139124	4.37E-18
KMT2B	0.07663759	4.38E-18
MRPL10	-0.1451578	4.38E-18
COL9A2	0.07858497	4.48E-18
GNL3	0.1758943	4.50E-18
SLC6A6	-0.2055884	4.50E-18
TYW1	0.11290916	4.55E-18
GTF3C1	-0.0082427	4.57E-18
HEATR5B	-0.1104767	4.59E-18
RFX7	0.08038592	4.63E-18
AHCY	0.00216558	4.67E-18
AL138963.4	0.06393578	4.67E-18
MOK	-0.1687636	4.68E-18
YTHDF3	0.01556108	4.68E-18
EIF4E	-0.1260542	4.76E-18
RSRC1	0.18533739	4.77E-18
ZNF566	0.14556732	4.79E-18
IFT20	-0.0831466	4.80E-18
EFR3A	-0.040349	4.83E-18
CHTOP	-0.0492089	4.85E-18
TMEM156	0.14316975	4.85E-18
P4HA1	0.19888219	4.93E-18
ARID2	0.02777836	4.95E-18
SUCNR1	0.41617177	4.97E-18
RASAL3	0.16550579	5.00E-18
ATP5MPL	0.33555147	5.03E-18
SLC29A1	0.12363313	5.05E-18
AASDHPPT	0.02021863	5.11E-18
AGO1	0.12497327	5.13E-18
ARCN1	-0.0235499	5.19E-18
MAF1	-0.1411756	5.21E-18
TNFAIP8L1	-0.1286218	5.23E-18
AC138150.1	0.10681893	5.24E-18
PLD3	-0.1178028	5.26E-18
ALDH9A1	0.03511962	5.27E-18
FAM50B	0.062639	5.29E-18

NOTCH1	-0.2419003	5.31E-18
ALG11	0.09709956	5.33E-18
UAP1	-0.0452793	5.36E-18
STXBP3	0.20051266	5.36E-18
USO1	0.21241832	5.41E-18
MARCH3	0.11726063	5.47E-18
SNX13	0.05197226	5.53E-18
PDHX	-0.0053278	5.55E-18
GPN1	-0.0342716	5.70E-18
CCS	-0.0899174	5.71E-18
AP2B1	0.09471519	5.73E-18
WARS2-AS1	0.10714477	5.77E-18
C6orf62	0.1511343	5.82E-18
SAP30BP	-0.2048105	5.82E-18
ATP8B2	-0.2342259	5.94E-18
TMEM220	0.19241002	6.08E-18
TNPO3	-0.0396606	6.10E-18
USP28	0.11013653	6.12E-18
TRMT61B	0.0605163	6.41E-18
DNASE2	0.12352168	6.46E-18
SUPT20H	0.15519476	6.52E-18
AHCYL1	0.16790245	6.53E-18
C3orf38	0.00411355	6.54E-18
AC060780.1	0.2265475	6.60E-18
CASP2	0.17725095	6.65E-18
LENG1	-0.1011314	6.71E-18
ITPRID2	-0.2580989	6.72E-18
SHISA5	-0.0875448	6.87E-18
CIITA	0.2098754	6.88E-18
C16orf87	0.08628148	6.98E-18
C1RL	0.10798558	7.10E-18
PTGER2	-0.2551558	7.22E-18
RCOR1	0.07581485	7.39E-18
DHX33	0.14184276	7.50E-18
NUP50	0.0397835	7.51E-18
RNF19A	-0.2075167	7.55E-18
CBLB	-0.2060866	7.61E-18
RIOK2	0.05391701	7.62E-18
TGIF1	5.9487E-05	7.67E-18
HMGXB4	-0.136771	7.79E-18
GNPTAB	0.3400728	7.80E-18
SLC43A2	-0.3030415	7.84E-18
CKAP5	0.10046525	7.94E-18
MED27	-0.0568558	7.96E-18
IK	-0.0011118	7.98E-18
PHYKPL	-0.162003	8.08E-18
SCAMP4	-0.0271268	8.13E-18
NQO2	0.04524015	8.15E-18
UBE2W	0.06686307	8.16E-18
KDM3B	0.10227171	8.21E-18

UFD1	-0.0272735	8.22E-18
MRPL21	-0.1274457	8.23E-18
ATXN2L	-0.0313136	8.24E-18
TALDO1	0.29881937	8.25E-18
ADAMTSL4	-0.2222042	8.39E-18
GPS2	0.09326814	8.42E-18
EMC8	-0.1703719	8.44E-18
CPD	0.11253425	8.50E-18
SNAP23	0.22044044	8.87E-18
NSMCE1	0.09534531	8.91E-18
MR1	0.09538074	8.93E-18
NBAS	0.0748058	8.94E-18
LRRC8A	-0.2979917	8.96E-18
DGKA	-0.2389339	9.11E-18
PDHB	0.03747842	9.12E-18
BAD	0.03317902	9.14E-18
ZNF746	0.01570632	9.32E-18
CEP76	0.10699964	9.44E-18
C12orf10	-0.1485279	9.49E-18
AC025171.2	0.22665257	9.53E-18
FANCF	0.08532842	9.54E-18
TMEM131L	-0.2173726	9.54E-18
FAAP20	-0.0264889	9.66E-18
ZBTB1	0.15547187	9.68E-18
WEE1	-0.2157368	9.73E-18
COQ9	0.0681723	9.77E-18
TRPC4AP	-0.1250034	9.83E-18
COL4A5	0.16629609	9.87E-18
AL499604.1	0.07728617	1.01E-17
ZMAT3	0.10478971	1.02E-17
PHF1	-0.1704464	1.02E-17
KIAA1586	0.09835586	1.03E-17
PRKX	0.04494007	1.04E-17
STOM	-0.2308605	1.05E-17
CD99L2	0.08017717	1.05E-17
DAZAP2	0.18603366	1.06E-17
PCBP1	0.24431065	1.07E-17
MCM6	-0.0433094	1.07E-17
CCDC71L	-0.0731832	1.07E-17
PNPO	0.06218081	1.08E-17
CPSF3	0.13057602	1.09E-17
MPV17	0.10866761	1.09E-17
MOB4	-0.0592753	1.10E-17
MGLL	0.01088101	1.10E-17
RGS16	0.15837737	1.11E-17
ATG4C	0.13728082	1.13E-17
CCZ1	-0.0075033	1.13E-17
ADRB2	0.20717823	1.13E-17
POLR2I	0.13654946	1.14E-17
NDUFS2	0.19531461	1.16E-17

TGFBR2	-0.1335017	1.17E-17
NCSTN	0.07859905	1.17E-17
WBP11	-0.2188635	1.17E-17
TM4SF1	-0.2358781	1.18E-17
C20orf96	0.08345179	1.18E-17
BTBD2	0.06325174	1.19E-17
MAZ	0.27839034	1.20E-17
CERS2	0.1608052	1.20E-17
CRYL1	0.10944398	1.21E-17
YIPF6	-0.079047	1.22E-17
PAQR3	0.08503764	1.23E-17
NFIL3	0.14871603	1.23E-17
MTX1	-0.1396118	1.23E-17
SPTLC1	0.10448359	1.23E-17
SNRK	-0.0463948	1.23E-17
ANGEL2	0.18259623	1.24E-17
TBC1D8	0.07082158	1.24E-17
HSDL2	0.15587013	1.26E-17
WDR46	0.09761397	1.27E-17
CDK7	0.01064245	1.27E-17
CSKMT	0.17927123	1.29E-17
MAFK	-0.1814283	1.30E-17
PFDN1	0.08562171	1.30E-17
MIS18A	0.10036956	1.31E-17
NUP155	0.09618203	1.32E-17
NFIC	0.22605435	1.34E-17
PXK	0.25990186	1.35E-17
GTPBP6	0.11631475	1.35E-17
CDC42EP2	-0.1459412	1.35E-17
NKIRAS2	-0.108147	1.35E-17
NAGPA	0.09362442	1.35E-17
DEF6	-0.0385568	1.36E-17
TAP1	0.00635639	1.37E-17
ADGRG6	0.15833569	1.37E-17
LSM14B	-0.0228691	1.37E-17
PLEKHA5	0.19504683	1.38E-17
LINC01684	0.17425668	1.38E-17
ORMDL1	0.33563991	1.38E-17
PKD1	0.07768789	1.39E-17
PXN	-0.162602	1.41E-17
SPPL3	-0.1836573	1.41E-17
S100PBP	0.16041297	1.43E-17
EEF1A1	-0.2558085	1.43E-17
NTMT1	-0.1218875	1.43E-17
HK2	0.09667166	1.44E-17
PAXX	-0.0543782	1.45E-17
ZNF585A	0.05703916	1.45E-17
PWWP2A	0.0963281	1.47E-17
SF3B4	-0.0930923	1.49E-17
SLC35B3	0.12229535	1.53E-17

MAP3K12	0.07927674	1.57E-17
ATP6V1A	-0.1670351	1.58E-17
UPF2	0.28786267	1.59E-17
ZNF91	0.23126145	1.59E-17
EBPL	0.26922426	1.59E-17
ZNF346	0.05719883	1.60E-17
ARPP19	-0.2639029	1.60E-17
ZNF667-AS1	-0.0919715	1.61E-17
EIF2AK4	0.14305006	1.61E-17
HSPB1	-0.2639556	1.62E-17
RCBTB1	0.15478139	1.62E-17
RAD54L2	0.04206881	1.63E-17
MRPS14	0.04515367	1.65E-17
INTS13	0.11040141	1.66E-17
FAM222B	-0.0901272	1.68E-17
TMEM42	0.14941703	1.72E-17
HDLBP	0.16471442	1.76E-17
DMAC2L	0.19511496	1.76E-17
ZSCAN16-AS	0.22022797	1.80E-17
ABHD17B	0.06164458	1.83E-17
SOX12	0.09761862	1.84E-17
KIAA2013	0.12674638	1.87E-17
PSMB9	-0.0486252	1.88E-17
TXNDC12	0.1600748	1.88E-17
NSMCE4A	0.02460034	1.89E-17
DFFB	0.10397506	1.90E-17
AC004540.1	0.12281927	1.91E-17
PLSCR1	-0.0034995	1.92E-17
SPECC1	0.21054162	1.94E-17
LTBP3	-0.1852152	1.95E-17
COPA	-0.1661254	1.96E-17
RPS6KA4	-0.1128405	1.97E-17
FAM136A	0.19037508	1.98E-17
SNX19	0.04136277	1.98E-17
MYDGF	-0.0917016	1.99E-17
MBNL3	0.16032807	2.00E-17
ZNF598	-0.155901	2.00E-17
CCDC106	0.09545263	2.02E-17
MFAP1	-0.1312312	2.03E-17
PPP1R7	0.11338914	2.03E-17
ATP6AP2	0.07311831	2.04E-17
DYRK3	-0.2716847	2.06E-17
KIAA1958	0.15465299	2.08E-17
DDX23	0.16341738	2.11E-17
SNX27	0.11068402	2.14E-17
XPO5	0.10488649	2.14E-17
CFL2	0.10403856	2.16E-17
EPHB6	0.09914491	2.17E-17
TMEM258	0.27936667	2.21E-17
SLC2A4RG	0.13429902	2.22E-17

UIMC1	-0.1308326	2.24E-17
PSMA4	0.25763454	2.24E-17
TTBK2	0.17395663	2.24E-17
TGFBR1	-0.1632704	2.26E-17
RPL11	0.16235664	2.27E-17
GMPR2	0.13919585	2.29E-17
RPL7A	-0.2288007	2.29E-17
CRLF3	0.07538147	2.29E-17
GEMIN6	0.11023323	2.29E-17
MCUR1	0.05649275	2.30E-17
DAP3	0.08526021	2.30E-17
FTSJ3	0.10189577	2.31E-17
ADAM10	0.17420268	2.32E-17
CD63	-0.3128594	2.32E-17
NR2C2	0.01205595	2.32E-17
HIST1H4E	0.1565309	2.33E-17
SUPT5H	0.03629234	2.33E-17
TM2D1	0.18029333	2.34E-17
ALDH3A2	0.10138739	2.36E-17
PSMB6	-0.13957	2.36E-17
TFB2M	-0.0388409	2.37E-17
CSNK1G3	-0.1670012	2.37E-17
VAMP5	0.14163567	2.40E-17
AFF4	-0.1620474	2.40E-17
AC012615.1	0.06514685	2.40E-17
PPFIBP1	0.08239513	2.41E-17
CASD1	0.0907238	2.41E-17
IFNAR2	0.14940751	2.42E-17
CFLAR	0.33904266	2.44E-17
MCRIP1	-0.1418618	2.45E-17
SF3A3	0.05076501	2.47E-17
VAPA	-0.3585618	2.50E-17
C11orf1	0.17290293	2.50E-17
ARRB1	0.22028307	2.52E-17
ASH2L	0.12419974	2.53E-17
ZADH2	0.08511201	2.54E-17
GPATCH4	0.21632522	2.55E-17
DESI2	-0.0981696	2.56E-17
SLC25A39	-0.1050769	2.57E-17
LACTB	-0.1862526	2.61E-17
DBT	0.17036883	2.72E-17
KLHL9	-0.0705902	2.72E-17
SIPA1	0.05699067	2.72E-17
C7orf50	0.1160351	2.76E-17
CLNS1A	0.3062549	2.76E-17
MRPL28	-0.034205	2.81E-17
TSR1	0.10731689	2.85E-17
NADK2	0.13553278	2.89E-17
HSF1	-0.1343543	2.91E-17
ARRDC2	-0.1963049	2.91E-17

DBNL	-0.0852171	2.94E-17
RNPC3	0.19101141	2.96E-17
PPID	0.15557067	2.98E-17
PHKG2	0.05961416	3.00E-17
FUT4	0.15075675	3.00E-17
CHMP2A	-0.1976796	3.08E-17
RASSF5	-0.1385004	3.08E-17
ITGB2	-0.2701459	3.12E-17
NUP58	-0.0082618	3.12E-17
TMEM230	0.20508584	3.15E-17
HARS	-0.000572	3.16E-17
B3GAT3	-0.0456169	3.16E-17
SAT2	0.01567687	3.17E-17
LIME1	-0.284138	3.21E-17
ZRANB1	-0.1696187	3.22E-17
WDR83	0.06294583	3.23E-17
TMEM170B	0.09438521	3.25E-17
SLC11A2	0.0674253	3.27E-17
CREB3L2	0.12750408	3.31E-17
ZDHHC2	0.09376586	3.32E-17
LSM4	0.0502598	3.35E-17
HPS6	-0.0786214	3.38E-17
AC015802.6	0.08488573	3.41E-17
VSIG10	0.03406707	3.41E-17
VWF	0.32999698	3.44E-17
TSFM	0.04868088	3.44E-17
EFCAB14	0.16989442	3.45E-17
NEK7	0.09622174	3.47E-17
NDUFV1	-0.1323811	3.49E-17
UQCC3	0.08066175	3.53E-17
ALKBH5	0.09046522	3.55E-17
RO60	-0.0874499	3.58E-17
AL022311.1	0.01349357	3.61E-17
FRYL	0.12754209	3.62E-17
PPM1H	0.11586438	3.63E-17
RNF135	0.07616757	3.64E-17
COPZ1	-0.0626944	3.65E-17
MAP3K1	-0.2445479	3.66E-17
SMPD2	-0.1667492	3.68E-17
RCHY1	-0.1330317	3.70E-17
ICAM2	-0.1699702	3.87E-17
ZNF830	0.21060088	3.88E-17
ZNF333	0.05392392	3.90E-17
STT3A	0.11561908	3.90E-17
ZNF414	-0.1400228	3.90E-17
TOR1A	0.04094501	3.91E-17
CYP4V2	0.09280725	3.93E-17
CCDC90B	0.03585528	3.94E-17
GPN3	0.04708852	3.99E-17
EXOC2	0.09325791	4.03E-17

TARS	-0.1182525	4.04E-17
RPL26L1	0.13035008	4.09E-17
HEMK1	0.10969733	4.11E-17
CMPK2	0.11698144	4.13E-17
WDR74	0.0910495	4.15E-17
C5orf63	0.1181828	4.20E-17
C21orf62-AS	0.12944998	4.21E-17
TBC1D15	-0.1492884	4.21E-17
HIVEP1	-0.2920864	4.27E-17
RPL24	0.17697574	4.30E-17
HERC4	0.22303149	4.31E-17
DLG5	0.05960062	4.32E-17
BMF	-0.1699014	4.32E-17
BUB3	0.11446205	4.37E-17
PPP2R2D	0.00518974	4.39E-17
CNOT9	-0.0923263	4.43E-17
C14orf119	-0.0667592	4.45E-17
TRPT1	0.12759187	4.46E-17
CRKL	-0.0588665	4.46E-17
ATG16L2	0.05319809	4.49E-17
PLEKHA2	-0.0835368	4.49E-17
TPP1	0.12262112	4.52E-17
SON	0.24771987	4.53E-17
HPF1	0.18290226	4.58E-17
OSCAR	-0.2002884	4.66E-17
CCP110	-0.0055511	4.68E-17
ANKRD17	0.20042258	4.68E-17
FAM76A	-0.1646215	4.69E-17
ADO	0.03773414	4.71E-17
CSNK1E	-0.1030229	4.83E-17
PPP2CB	-0.1033395	4.83E-17
MAP10	0.07208945	4.84E-17
RHBDF2	-0.2313485	4.91E-17
CCDC61	0.05446678	4.91E-17
SERGEF	0.06513876	4.92E-17
PIGL	0.06794535	4.92E-17
RAF1	-0.0690791	4.94E-17
MORC2	0.1858589	4.96E-17
DNAJC4	-0.0113909	5.03E-17
SEPHS2	0.12184654	5.03E-17
NUP160	0.04874038	5.05E-17
MZT1	0.07199507	5.10E-17
MAPK6	0.09824411	5.17E-17
MCM4	-0.0300262	5.17E-17
PLIN3	-0.2369003	5.17E-17
MIIP	0.06206574	5.19E-17
CFAP97	0.2282173	5.19E-17
AC005332.6	0.06686327	5.20E-17
C17orf80	0.09566188	5.24E-17
CTU1	0.00236809	5.24E-17

PRSS21	-0.1624209	5.29E-17
SLC35E3	0.20890993	5.32E-17
POLR1A	0.10270315	5.33E-17
KCMF1	-0.2494449	5.34E-17
BRD9	-0.0103453	5.35E-17
MKRN1	-0.1203428	5.36E-17
PINK1	0.0547981	5.37E-17
TM9SF1	-0.1067748	5.38E-17
C19orf48	-0.0079314	5.39E-17
SERTAD2	-0.1434673	5.40E-17
NUP85	0.07578868	5.43E-17
SERINC5	0.1546483	5.46E-17
UTP20	0.14881254	5.46E-17
DHRS7B	0.05588463	5.52E-17
POC5	0.11458725	5.53E-17
SELENOO	-0.0092056	5.62E-17
SCO2	-0.1367031	5.64E-17
RDH14	-0.0520074	5.69E-17
RANBP6	0.11562529	5.69E-17
SPON2	-0.1319138	5.75E-17
PSMD14	-0.0901895	5.75E-17
TTC32	0.10154485	5.79E-17
PPP1R9B	0.02645237	5.83E-17
ATP5PF	0.27802891	5.84E-17
GYG1	0.00868273	5.96E-17
ALKBH2	0.09522968	5.97E-17
MARC2	0.07125261	6.03E-17
ASXL2	0.07829559	6.03E-17
TIMM23	-0.1074313	6.05E-17
CLN8	-0.1607016	6.06E-17
EDEM2	0.1198707	6.09E-17
NUTM2A-AS	0.17347483	6.12E-17
PPP2R5D	0.05974913	6.14E-17
THOC6	0.10058877	6.25E-17
IL4I1	-0.1592196	6.25E-17
NELFB	-0.0049584	6.32E-17
RBCK1	-0.1397994	6.32E-17
DGKE	0.19589327	6.36E-17
SAR1B	0.06391213	6.37E-17
OSBPL5	0.09785951	6.40E-17
RTCB	-0.0626774	6.43E-17
USP39	0.03149524	6.45E-17
RCC1	0.06203394	6.49E-17
CDKL3	0.07627298	6.52E-17
FAM92A	0.12093486	6.57E-17
RFFL	0.06621901	6.61E-17
GIGYF1	0.04292768	6.66E-17
B9D2	-0.1446241	6.66E-17
MSRA	-0.0615231	6.73E-17
CUL4B	0.14256523	6.78E-17

TMTC4	0.04273588	6.85E-17
COA5	0.11023509	6.86E-17
NLRP2	0.06972265	6.90E-17
STRADB	0.10166111	7.03E-17
AL355488.1	0.07420886	7.17E-17
BTN3A1	0.07912014	7.22E-17
SLC30A1	-0.2138863	7.26E-17
INTS3	0.04286374	7.29E-17
DNAH14	0.13459639	7.34E-17
CCDC58	0.1149338	7.36E-17
SIPA1L3	0.10864025	7.36E-17
AC009107.2	0.04563396	7.49E-17
EIF4A3	0.06279136	7.49E-17
RPS6KA5	-0.2703403	7.55E-17
SLC30A7	0.09029228	7.55E-17
KHSRP	0.14256534	7.67E-17
ALKBH7	0.36222556	7.67E-17
UBXN6	-0.0230023	7.73E-17
MBTD1	0.14365136	7.73E-17
LINC00909	0.17760322	7.80E-17
SLC30A6	0.07953169	7.81E-17
EIF2D	0.01702263	7.89E-17
RFX8	0.08906864	7.95E-17
CARD9	0.11222223	7.97E-17
UPK3BL1	0.00034121	8.13E-17
RRS1	0.13726386	8.13E-17
TRRAP	0.11112437	8.16E-17
ASB16-AS1	0.09007626	8.28E-17
SDE2	-0.2197634	8.28E-17
USP36	-0.0809491	8.39E-17
PCIF1	-0.2406183	8.46E-17
CDADC1	0.12609881	8.47E-17
OXLD1	0.01531825	8.68E-17
AFMID	0.14199787	8.71E-17
TOR2A	-0.0438754	8.71E-17
MTRES1	0.0536907	8.78E-17
ZNF226	0.06288815	8.78E-17
DUSP18	-0.2347388	8.87E-17
ERAP2	0.0925745	8.87E-17
MYO15B	-0.0954061	8.94E-17
USP47	-0.1828539	8.99E-17
MGAT4A	0.05534209	9.04E-17
ATXN3	0.11296062	9.07E-17
FLI1	0.06059594	9.07E-17
PRPF18	0.09124246	9.10E-17
EIF2A	0.22459931	9.11E-17
TNFSF14	0.07575928	9.15E-17
NDUFS1	0.19765765	9.23E-17
TBL1XR1	0.20484993	9.30E-17
C12orf65	-0.2036137	9.30E-17

CDK14	0.12309234	9.32E-17
TAPBP	-0.2670678	9.32E-17
DNAJC9	-0.0579654	9.35E-17
COPB2	0.03205804	9.36E-17
MOSPD1	0.04710588	9.38E-17
POLR2D	0.14393969	9.38E-17
TRAPPC6A	0.09900673	9.50E-17
ARNT	0.05570614	9.57E-17
GET3	-0.0091753	9.59E-17
MARS	-0.1773252	9.77E-17
RPL22L1	0.36822357	9.86E-17
PDIK1L	0.06976968	9.93E-17
VAMP3	-0.0945796	9.93E-17
STRN4	-0.1178589	1.00043E-16
USP15	-0.3244444	1.00578E-16
WRN	0.13141102	1.01721E-16
BCAP31	-0.2365327	1.02264E-16
SASH3	-0.1057925	1.03538E-16
UBA3	0.16468198	1.04603E-16
TRAPPC8	0.01154945	1.0502E-16
DNAJC21	0.22083412	1.07184E-16
IREB2	-0.0161584	1.07248E-16
LMAN1	0.15604357	1.08942E-16
SLC10A3	-0.1497645	1.09854E-16
CAP1	-0.3227314	1.10262E-16
CNPPD1	-0.0635328	1.10329E-16
TCP11L2	-0.2909254	1.10462E-16
TMEM87B	0.03135882	1.12842E-16
EEA1	0.21741892	1.12911E-16
NDUFA10	0.06842883	1.13102E-16
AC036176.1	0.0577031	1.14016E-16
TPST2	-0.1831059	1.14183E-16
MED6	-0.1083777	1.14196E-16
C19orf12	-0.1291266	1.14764E-16
PPP1CA	-0.1546844	1.15922E-16
PEF1	-0.1961253	1.161E-16
GLRX3	0.10577109	1.16423E-16
GABPA	0.10274318	1.16762E-16
GOPC	0.12398484	1.17411E-16
BLOC1S4	0.00864722	1.18088E-16
PAIP2	-0.3411229	1.18986E-16
PGBD4	0.1072056	1.19103E-16
PTBP1	0.0220207	1.21907E-16
PTMA	0.24871891	1.2232E-16
WDR73	-0.0956035	1.25719E-16
UBE2Q2	-0.2306744	1.28869E-16
SLC35B1	-0.1059468	1.30055E-16
APEH	0.09783722	1.30253E-16
BCL2L12	0.03806651	1.33574E-16
TBC1D2B	-0.0802371	1.33601E-16

NDUFA9	-0.0825037	1.34024E-16
INPP4A	-0.0482881	1.35535E-16
MED21	0.12855842	1.35734E-16
SNX20	0.05462602	1.35984E-16
LCP2	-0.0045349	1.36434E-16
NUP98	-0.1650544	1.37393E-16
YWHAG	-0.2618268	1.38027E-16
CCDC115	0.10863049	1.39204E-16
AC026471.1	0.05861392	1.39231E-16
ELP2	0.1216542	1.40608E-16
AC005562.1	-0.0149206	1.42186E-16
CFAP44	0.09393224	1.42573E-16
CHMP4B	0.01613122	1.42782E-16
TERF1	0.12472111	1.43021E-16
PITPNA-AS1	0.01985326	1.44428E-16
ATAD1	-0.0032182	1.46183E-16
SIRT7	-0.0621453	1.46445E-16
SFT2D3	0.04971294	1.47088E-16
GPSM1	-0.1513654	1.47418E-16
PLA2G4A	-0.0884414	1.47531E-16
TTC5	0.10922595	1.48268E-16
TIMM13	0.24959047	1.4838E-16
NNT-AS1	0.14678724	1.48865E-16
TPT1-AS1	0.09810161	1.51691E-16
ERF	-0.255667	1.51856E-16
TENT4B	-0.0146265	1.53086E-16
SLC2A3	-0.2950955	1.53963E-16
SMARCA5	0.25970332	1.55101E-16
CEPT1	0.08998547	1.57753E-16
SYMPK	0.02072501	1.58709E-16
TTC28-AS1	0.10231907	1.59148E-16
EBLN3P	0.12530791	1.59262E-16
SCARB2	-0.204654	1.62521E-16
HDAC1	0.16201558	1.64268E-16
LEMD3	-0.0886608	1.64762E-16
CHML	0.25073382	1.68008E-16
RASA2	-0.2176007	1.68144E-16
CBR4	0.13085633	1.69798E-16
BRCC3	0.12777948	1.70825E-16
CDCA4	-0.1628419	1.73027E-16
CXXC1	0.11579858	1.73347E-16
SLC17A5	-0.1692437	1.74847E-16
ANKRD10	0.0734108	1.79084E-16
FBXO42	-0.0192173	1.7962E-16
DROSHA	0.06623665	1.79645E-16
SENP5	-0.0569881	1.7989E-16
SOCS5	0.05814537	1.80744E-16
GIMAP2	0.06007246	1.80931E-16
TTLL3	0.00415128	1.8116E-16
GOLGA8A	0.10881629	1.81301E-16

TXLNG	0.05051185	1.82556E-16
TLNRD1	-0.1827147	1.84342E-16
HADHB	0.04918564	1.85191E-16
RETREG2	0.11771108	1.86927E-16
LEO1	0.02814103	1.88321E-16
ZBTB38	0.13290338	1.8885E-16
TLK2	-0.0110324	1.90108E-16
MRPL9	-0.0494898	1.90744E-16
DKC1	0.22556759	1.93553E-16
C12orf29	0.11475695	1.94618E-16
PRRC2A	0.04998571	1.94673E-16
FAM219B	-0.1550321	1.94742E-16
ACO2	-0.0087163	1.95275E-16
TAGAP	-0.1350771	1.96475E-16
RAB5C	-0.2444686	1.97259E-16
SAC3D1	0.13545862	1.98218E-16
HAUS4	0.0333947	1.99837E-16
SUPT7L	-0.0760341	2.00175E-16
RAB1B	-0.0452366	2.0252E-16
LRRC41	-0.0352931	2.0376E-16
PLEKHH3	-0.1618151	2.04939E-16
RPE	0.10864172	2.06629E-16
POLR2B	-0.0623001	2.08032E-16
PAN3-AS1	0.14257006	2.09324E-16
DDX19A	0.07434368	2.09586E-16
CASP7	-0.1565091	2.11941E-16
BACE1-AS	0.08912617	2.12847E-16
SPRTN	0.07291879	2.13029E-16
SGPL1	-0.1480652	2.15326E-16
ERVK13-1	0.04993084	2.16128E-16
DRG1	-0.0053769	2.18395E-16
LINC02693	0.07255777	2.20788E-16
RAD51-AS1	0.0956303	2.21201E-16
EEFSEC	0.10274867	2.21421E-16
MAP7D3	0.13849414	2.22385E-16
VPS45	0.10169997	2.26794E-16
DCUN1D4	0.03111362	2.27723E-16
CCNC	-0.0488208	2.28914E-16
HINT3	0.08319856	2.29339E-16
KDM5B	-0.3669082	2.31759E-16
FRA10AC1	0.15122497	2.32077E-16
TMC3-AS1	0.07819651	2.36642E-16
MRPL16	0.10043602	2.3784E-16
PEX3	0.04307563	2.39196E-16
MRPL41	-0.0862053	2.40255E-16
ZDHHC3	0.02442431	2.40565E-16
CPNE3	0.2150852	2.41132E-16
THAP4	-0.0115183	2.43192E-16
PUF60	-0.1570228	2.43663E-16
SERPINB1	0.35377559	2.46172E-16

RASSF2	-0.1351193	2.46718E-16
SUZ12	0.1315651	2.51054E-16
PARP8	0.03263057	2.51405E-16
RLIM	-0.2189729	2.54444E-16
PAXIP1	0.06044301	2.5517E-16
USP19	0.06751086	2.55232E-16
SPIN1	0.14502845	2.5721E-16
RNF10	-0.2544743	2.59757E-16
PAPSS1	0.09691034	2.61245E-16
PRSS2	-0.7246676	2.61487E-16
FIP1L1	-0.0922302	2.63782E-16
ALG13	0.11841122	2.64126E-16
ERICH1	0.15280777	2.65322E-16
MRPS12	0.18503872	2.66465E-16
TMED1	-0.0533575	2.66583E-16
GLTP	-0.147258	2.6758E-16
ANKIB1	0.13536276	2.69099E-16
PPP1R8	-0.0669048	2.73831E-16
CNOT1	-0.1103463	2.73961E-16
DHX15	-0.0671503	2.76733E-16
MRPS18A	-0.1273038	2.79163E-16
CASC3	0.02192075	2.79851E-16
NAF1	0.02378634	2.80332E-16
TFDP1	0.08208455	2.81648E-16
AC093673.1	-0.1345516	2.81714E-16
LETM1	0.07228694	2.81929E-16
L2HGDH	0.11844394	2.82627E-16
TBC1D20	0.02223437	2.89617E-16
SMARCB1	0.03616815	2.89659E-16
MRPL40	0.13693736	2.90697E-16
ZNF639	0.06765057	2.91943E-16
MAPKAPK5	0.03676948	2.94427E-16
GUCY1B1	0.12651216	2.95208E-16
SKP2	0.07858885	2.96424E-16
TBRG4	-0.0410986	2.97973E-16
CNPY3	-0.235001	2.97975E-16
FAM91A1	0.00479607	3.0237E-16
YBEY	0.06336426	3.03929E-16
ZNF350	-0.0079385	3.04193E-16
LDAH	0.11643578	3.10921E-16
TMED9	-0.0530749	3.11573E-16
C5orf15	0.0125614	3.12252E-16
ZNF428	-0.1126536	3.12652E-16
LANCL1	0.035534	3.13091E-16
MED8	-0.149841	3.16909E-16
FAM20B	0.13349045	3.17391E-16
PHACTR1	0.15363785	3.19218E-16
JAK1	0.16263668	3.22862E-16
ZBTB11	0.00612653	3.25586E-16
EIF2S1	-0.1504777	3.27101E-16

ISCA1	-0.0957329	3.27975E-16
RP2	-0.1507405	3.37261E-16
CRK	-0.2556508	3.3785E-16
KAT14	0.10361379	3.39423E-16
GPAA1	-0.0820215	3.46493E-16
PAQR7	0.08046927	3.50357E-16
MPHOSPH1(	0.1463003	3.52403E-16
DEXI	-0.0122114	3.54331E-16
DPY19L2	0.13963926	3.56779E-16
IL6R	-0.1950104	3.58992E-16
RABGEF1	-0.2114532	3.62264E-16
INAFM2	0.0928818	3.6571E-16
METTL3	0.06407283	3.66419E-16
GCNT1	0.22210206	3.66557E-16
ZNF410	-0.1475417	3.67296E-16
ADNP2	-0.2172654	3.70643E-16
KLHL23	0.05596606	3.74845E-16
CORO7	-0.1785223	3.76813E-16
ANGPTL6	0.07366121	3.79774E-16
C5orf51	0.0298635	3.80227E-16
LRG1	0.09786556	3.84477E-16
CFAP298	-0.0193124	3.86421E-16
RRP1B	0.14307928	3.86443E-16
TATDN1	0.22350815	3.93221E-16
PACRGL	0.0847678	4.00607E-16
CLK4	-0.0713075	4.01673E-16
HPS3	0.11767533	4.04599E-16
RBM22	-0.1205435	4.07432E-16
WDTC1	0.0521769	4.07497E-16
ACBD6	0.12052046	4.07826E-16
SCFD1	-0.1372464	4.09543E-16
UBAP2L	0.08823441	4.1024E-16
OTUD4	-0.0390095	4.12886E-16
SLC35F2	0.05808546	4.13624E-16
PIWIL4	0.09817537	4.17419E-16
RASA3	0.16302243	4.1823E-16
HTATSF1	-0.0963548	4.20679E-16
TMEM9B	-0.0092606	4.24039E-16
NASP	0.19850689	4.24388E-16
CISD2	0.12870178	4.34539E-16
SIGMAR1	0.09904888	4.36102E-16
RIC8A	-0.1066197	4.37042E-16
COPS3	-0.1130556	4.38935E-16
MACROD1	0.05901191	4.40659E-16
ATG10	0.08668518	4.4698E-16
SCHIP1	0.15248493	4.47942E-16
PPP3R1	-0.2425951	4.52229E-16
GM2A	0.15545637	4.52581E-16
GUF1	0.10373154	4.52697E-16
LETMD1	0.11029028	4.54471E-16

HSPA14.1	0.05796238	4.54521E-16
RANBP2	-0.2539788	4.5601E-16
SLC25A33	-0.0121418	4.62366E-16
CEP170	-0.2768373	4.62802E-16
MED12L	0.10611221	4.65714E-16
PPP4R2	-0.2284523	4.69727E-16
LARP4B	0.09836673	4.72101E-16
RAB3GAP2	-0.005836	4.7631E-16
ANTKMT	0.04651767	4.81034E-16
CAAP1	0.11002784	4.85174E-16
RIOX2	0.17787535	4.86509E-16
IRF3	0.1365526	4.87296E-16
CHKA	0.078507	4.89265E-16
VPS13B	0.11038712	4.91248E-16
ACBD5	0.06667934	4.93294E-16
AC245297.3	0.18514894	4.94011E-16
ZNF766	0.14527599	4.96287E-16
POLD2	0.09708823	4.98114E-16
CLIP2	-0.0741743	4.98505E-16
GNPAT	-0.0211632	4.99112E-16
TMEM18	0.16712372	5.00147E-16
PIP4P1	-0.1394474	5.0185E-16
PTGES2	0.00024864	5.04344E-16
SLC52A2	-0.0487569	5.0519E-16
ATP9B	0.08750974	5.09368E-16
ZNF260	0.09080998	5.10527E-16
TEX10	0.08754466	5.14172E-16
FHOD1	-0.015937	5.14625E-16
NOP2	0.07793534	5.20909E-16
GCSAML	0.22065949	5.23908E-16
GLG1	0.09807629	5.25836E-16
DGKZ	-0.127744	5.30692E-16
CNIH4	-0.1367355	5.37466E-16
RAP1A	-0.2616625	5.38992E-16
PRPSAP2	0.05669918	5.41031E-16
TMEM68	0.07961864	5.41693E-16
PNPLA4	0.08274676	5.49285E-16
RSPRY1	-0.1216006	5.49759E-16
ZBTB26	0.04253049	5.54675E-16
FOXK1	-0.0099557	5.55635E-16
PEPD	0.07406672	5.56195E-16
AC006504.5	0.12755866	5.60802E-16
CEP162	0.17502582	5.61575E-16
ATXN10	0.07803449	5.62529E-16
KATNA1	-0.1579643	5.66121E-16
C1orf109	0.07758151	5.70138E-16
TAPBPL	-0.1676687	5.70894E-16
SRP68	-0.0232138	5.74727E-16
TPD52L2	-0.0319599	5.84387E-16
CASC4	0.09813696	5.89359E-16

C4orf46	0.08655594	5.90738E-16
MCCC2	0.11332646	5.9092E-16
USP20	0.07322946	5.9994E-16
PHC2	-0.039205	6.07919E-16
HAUS3	0.08182413	6.07945E-16
BAG2	0.11478479	6.09349E-16
SLAIN2	-0.0289775	6.09508E-16
KAT6A	-0.2305938	6.1404E-16
MAP4	-0.061268	6.21191E-16
TBC1D10C	-0.1221669	6.26974E-16
ZNF587	0.09655416	6.53743E-16
WAC-AS1	0.2257366	6.58195E-16
VPS4A	-0.075159	6.58225E-16
TIMM10	0.02714123	6.63167E-16
ZNF720	0.14295729	6.6602E-16
TRIM24	-0.0062911	6.68567E-16
GALK1	0.07434895	6.68576E-16
GAPVD1	-0.0100386	6.69666E-16
KDSR	0.13036936	6.74385E-16
MRPS11	0.13052122	6.8979E-16
FGFRL1	0.03418373	7.03177E-16
CD164	0.3743306	7.04966E-16
RALB	-0.0817665	7.07985E-16
RABL6	0.05661807	7.14116E-16
DNMT3A	0.13567855	7.14801E-16
MRPS16	0.00144485	7.16436E-16
EVL	-0.0828714	7.18901E-16
ANKLE1	0.1034054	7.20659E-16
SAP30L	0.01299559	7.26788E-16
RHOQ	-0.0990309	7.31587E-16
CCDC82	-0.1624481	7.3193E-16
ATP11B	-0.0755755	7.34354E-16
CREB3	-0.167579	7.38039E-16
AL135925.1	0.07341328	7.39464E-16
EPB41L4A-A	0.20053677	7.39915E-16
PGS1	0.10073393	7.40044E-16
LARP1B	0.08589331	7.49427E-16
PHACTR4	0.14065757	7.50223E-16
AP2S1	0.04604549	7.58621E-16
ADPRM	0.07409233	7.72641E-16
C4orf48	-0.2866492	7.73036E-16
AC092329.4	0.02115051	7.75965E-16
RB1CC1	-0.282056	7.77167E-16
ALG2	-0.1375711	7.80505E-16
DNAJB4	0.09534944	7.87304E-16
BABAM1	-0.0033417	7.91102E-16
ILK	-0.0414786	7.98229E-16
RASSF1	-0.0385537	8.03281E-16
RAB11B	-0.0570944	8.04048E-16
SCAI	0.12824115	8.06581E-16

MED12	0.11377559	8.07966E-16
TFE3	-0.1013102	8.11888E-16
GTF3C3	0.07263725	8.1316E-16
ZNF519	0.0887622	8.14289E-16
ATL2	0.11859864	8.19302E-16
DEGS1	-0.1665675	8.24176E-16
ETFA	0.08319163	8.30682E-16
PAICS	0.16644051	8.44966E-16
ELK4	0.0197088	8.45685E-16
MTMR9	-0.1297285	8.51522E-16
DCAF8	0.06325762	8.52196E-16
BCAS2	-0.1590043	8.54068E-16
JARID2	-0.1568585	8.55369E-16
SEC22C	-0.1643233	8.56587E-16
RSAD2	0.09615348	8.59315E-16
ELOF1	-0.0860309	8.66549E-16
SH3BP1	-0.1356337	8.75557E-16
NOA1	0.09736982	8.81235E-16
ERGIC2	0.08833368	8.81543E-16
PRPSAP1	0.1348705	8.88561E-16
ZNF600	0.03290971	8.89796E-16
ATP2A3	0.15613696	8.90388E-16
TMEM33	0.06898521	8.93783E-16
MVB12A	0.02883585	8.95463E-16
BTD	0.07674387	9.02701E-16
COA7	0.0784205	9.03752E-16
FBXW7	-0.1603895	9.06723E-16
PTBP2	-0.1991708	9.16916E-16
NCBP2AS2	0.13013754	9.31086E-16
PITRM1	0.05639827	9.32643E-16
GGA2	0.10659058	9.40288E-16
ZBTB37	0.16233054	9.45068E-16
PSEN1	-0.0839123	9.48726E-16
LGALS3BP	-0.2492657	9.69135E-16
LATS1	0.05971495	9.7075E-16
COMMD2	-0.0223467	9.80811E-16
AARSD1	0.03216169	9.89431E-16
ZNF761	0.08796834	9.94283E-16
ZNF551	0.06589817	1.00662E-15
CEP104	0.10650878	1.00685E-15
CCDC18-AS	0.17361094	1.00977E-15
CLPTM1	-0.035599	1.01171E-15
REEP4	-0.0312215	1.01697E-15
RNF141	-0.0082572	1.02654E-15
GALNT4	-0.2047301	1.0295E-15
TRAPPC2	0.059584	1.04409E-15
FAM89A	0.0794064	1.04483E-15
BIRC2	0.0649387	1.04831E-15
SSNA1	0.00396443	1.05577E-15
KLHDC1	0.08191699	1.06087E-15

ID3	-0.2619272	1.06426E-15
MMAB	0.07792401	1.06932E-15
AGO4	0.05803536	1.08734E-15
MFF	0.05772579	1.09E-15
NFATC2IP	-0.0403145	1.09854E-15
AF117829.1	0.10264977	1.1076E-15
WBP1L	-0.1547889	1.11294E-15
RUFY3	-0.01842	1.12523E-15
TRIM4	0.08671061	1.13419E-15
NCAPD3	0.07985583	1.13452E-15
ZBED1	0.11588248	1.13588E-15
GCDH	0.06631214	1.14456E-15
TJAP1	0.00923281	1.14639E-15
ARFGEF1	0.06078401	1.14692E-15
NOL6	0.08135185	1.14698E-15
GMNN	-0.0596528	1.14793E-15
SLC66A2	-0.1207974	1.15491E-15
CSF1R	0.12773984	1.16917E-15
DNAJB12	-0.0948985	1.17114E-15
ADARB1	0.10321234	1.17194E-15
SMIM24	0.41996367	1.17715E-15
PRPF4	-0.0414824	1.1776E-15
IRAK4	0.16133194	1.18019E-15
C8orf33	0.11276939	1.18112E-15
USP10	-0.0219159	1.18205E-15
MTHFD1L	0.09682004	1.18652E-15
GTF3C6	-0.100717	1.18807E-15
ADSL	0.07949761	1.19435E-15
ACAD8	0.03038144	1.19457E-15
ARL6IP4	-0.2974512	1.20317E-15
KIAA2026	0.10072229	1.20786E-15
MRPL12	0.09153424	1.20916E-15
ABCB7	0.13367768	1.21309E-15
TAF7	0.29720349	1.21652E-15
DDX52	0.0348418	1.21686E-15
CTDSP1	-0.085777	1.22029E-15
NMT1	0.06494948	1.23361E-15
UCHL3	-0.0180909	1.23767E-15
C22orf39	0.00200545	1.23987E-15
SNHG15	-0.2056157	1.24914E-15
UBR5	-0.188365	1.25584E-15
CHP1	0.01806849	1.25652E-15
AKAP1	0.13161151	1.26069E-15
ARSB	0.08948305	1.26196E-15
SOAT1	0.12495336	1.2641E-15
AAK1	0.21928497	1.26672E-15
PAK1IP1	0.1735383	1.27065E-15
CUX1	0.24062381	1.27183E-15
NSUN5	0.10553078	1.28123E-15
NFATC3	0.08793653	1.29447E-15

CHAMP1	-0.1085899	1.29522E-15
GTF2E2	0.06051915	1.29782E-15
PARL	0.09503005	1.31297E-15
HDHD3	0.00721934	1.31607E-15
STAU1	-0.1339726	1.32872E-15
FAM214A	-0.0222594	1.33568E-15
ZNHIT3	-0.018792	1.35708E-15
RSRP1	0.32942683	1.36667E-15
SMARCC2	0.23205447	1.36972E-15
SMYD3	0.15908857	1.37176E-15
GOT2	0.05214001	1.4011E-15
STN1	-0.026654	1.40493E-15
PDRG1	-0.1498175	1.41543E-15
MAN2C1	-0.0490377	1.41671E-15
DNPEP	0.07481631	1.41719E-15
EAF2	-0.1930962	1.41976E-15
GMDS-DT	0.11794454	1.43729E-15
DPY19L3	0.12783204	1.44823E-15
CLK2	0.02717078	1.45224E-15
ARIH1	-0.1678491	1.45241E-15
AIFM1	0.14428996	1.45617E-15
CD151	-0.2070876	1.46562E-15
CTNND1	0.13696499	1.50037E-15
MFSD12	-0.1283639	1.50113E-15
RNF149	-0.2251094	1.50827E-15
PLIN2	0.1650069	1.50854E-15
ZFP64	0.06784733	1.51084E-15
ST8SIA4	0.0482048	1.5204E-15
TNPO1	0.09592795	1.53156E-15
SDF2	-0.0616464	1.53574E-15
MKRN2	-0.091975	1.54264E-15
NCK1-DT	0.14238102	1.54815E-15
ZBED3	0.00596232	1.55206E-15
EIF3G	-0.329236	1.55619E-15
TMCC1	0.0590354	1.55982E-15
CNP	0.07393117	1.56658E-15
UBE2L6	-0.0250272	1.56871E-15
AL009179.2	0.1011555	1.57117E-15
STAT3	-0.1590523	1.57152E-15
ZNF546	0.08828265	1.57175E-15
CLUAP1	-0.0190772	1.5982E-15
RICTOR	-0.211511	1.60858E-15
KMT2C	-0.0934119	1.62112E-15
MVP	-0.1805818	1.63243E-15
LAT2	0.31911498	1.63723E-15
XPO4	0.03732921	1.65532E-15
HNRNPH2	-0.1379052	1.66735E-15
CTSB	0.06064241	1.67007E-15
RASAL2	0.11475962	1.68996E-15
PAF1	-0.2207017	1.69592E-15

CES2	0.04555534	1.69895E-15
RABGGTB	0.10177728	1.6993E-15
UPF3B	0.18036006	1.69993E-15
SPSB3	-0.0616081	1.70283E-15
NIN	0.1867412	1.70431E-15
OTUD5	-0.1098538	1.71374E-15
MRPL24	0.16630823	1.71471E-15
KCNQ5	0.13718661	1.73609E-15
NPEPPS	-0.2211563	1.7532E-15
FTCDNL1	0.1138686	1.75356E-15
CRCP	0.02706825	1.75442E-15
ENDOV	-0.0966677	1.75479E-15
AC090825.1	0.08710884	1.76292E-15
KIAA1549	0.07376052	1.76307E-15
MIF4GD	0.06372019	1.76323E-15
SLF2	0.09400423	1.78424E-15
MIGA1	0.0066945	1.78675E-15
ZCCHC17	-0.0212836	1.8248E-15
ANAPC1	0.09925183	1.82924E-15
ITCH	-0.1349411	1.83115E-15
ZNF70	0.07528493	1.83254E-15
CPOX	0.06352032	1.83489E-15
PBX3	-0.2634824	1.83519E-15
S100A1	-0.264238	1.83541E-15
MYL6B	-0.0509238	1.8406E-15
SNX15	0.04558605	1.86952E-15
MRPL36	0.02538488	1.87576E-15
SFSWAP	-0.0321684	1.87705E-15
ZNF738	0.03559894	1.90027E-15
TEN1	-0.054084	1.91031E-15
FARSB	0.12374495	1.91764E-15
CCDC126	0.06802659	1.9368E-15
CREBBP	0.0101399	1.94318E-15
FBXO3	-0.0801224	1.94763E-15
ARHGEF9	0.05463422	1.96917E-15
TLR4	0.08698294	2.02367E-15
AATF	-0.0004605	2.02452E-15
PPP2R3C	0.0778472	2.02494E-15
C1orf52	-0.1268395	2.0276E-15
GALT	0.0397844	2.0322E-15
MGME1	0.05863956	2.03805E-15
FAM118A	-0.1206563	2.05087E-15
HACD2	0.09125615	2.06028E-15
DNMT3B	-0.0685212	2.06653E-15
HCCS	-0.1340697	2.07025E-15
FBXO22	0.23463008	2.08585E-15
C2orf74	0.12142164	2.1115E-15
SNAPC3	0.09791024	2.13341E-15
SMG7	-0.0742711	2.14391E-15
LIN7C	0.09607692	2.16451E-15

TAF15	0.21900334	2.17025E-15
SRGN	0.69685572	2.1777E-15
NLK	0.15983414	2.1812E-15
LAMTOR3	-0.054192	2.18316E-15
OBI1	0.06673638	2.18894E-15
ABHD13	0.10704978	2.20483E-15
FLOT2	-0.0424801	2.20875E-15
BCOR	-0.2319799	2.22133E-15
DPAGT1	0.06110948	2.22502E-15
UQCRC1	-0.0729858	2.2554E-15
AP1B1	0.00496353	2.2601E-15
MIEN1	0.05745282	2.27272E-15
BCL11A	0.27962981	2.28236E-15
PPIF	-0.2344733	2.301E-15
OAF	-0.1688434	2.30159E-15
HLA-DRA	-0.3623848	2.37376E-15
TRA2A	0.00038463	2.39595E-15
NOB1	0.1401348	2.40882E-15
MRRF	0.06837364	2.41495E-15
PIGH	0.07714122	2.44189E-15
SDAD1	0.12932446	2.45964E-15
CELF2	0.32567993	2.46039E-15
MCM2	-0.0135475	2.47507E-15
KIAA0586	0.12574618	2.4842E-15
NDUFA6	-0.20295	2.50363E-15
TMEM256	0.15651503	2.50948E-15
VDAC3	0.16512338	2.51263E-15
MAP4K3-DT	-0.0373232	2.51738E-15
COPG1	0.03853701	2.53356E-15
ARID5B	0.13006099	2.55634E-15
ABHD8	0.09343494	2.58845E-15
IPO5	0.10231171	2.59246E-15
ERCC6	0.09812002	2.59476E-15
TMEM50B	-0.034574	2.60966E-15
HIVEP2	-0.2223518	2.62349E-15
COPS7A	-0.0170141	2.62484E-15
TMEM127	-0.0742271	2.63737E-15
UBQLN2	0.07870699	2.65156E-15
CCNT2	-0.0721677	2.67199E-15
TOMM20	0.2921861	2.68336E-15
FAM43A	0.21648394	2.70195E-15
MRPL58	0.03761822	2.70357E-15
PIAS2	0.17091339	2.706E-15
SDHC	0.21833863	2.71154E-15
TMEM144	0.08152278	2.72493E-15
GPSM3	-0.3211325	2.7568E-15
BAG4	-0.011689	2.794E-15
EXOC7	0.01567086	2.80072E-15
LSM2	0.27129038	2.80319E-15
PRICKLE1	-0.0749864	2.83194E-15

SPAST	0.08354966	2.83937E-15
HIST2H2AC	0.12866771	2.85073E-15
RIOK1	0.05106246	2.86037E-15
RNF2	-0.1473924	2.88794E-15
SCOC-AS1	0.12426232	2.90659E-15
GGA1	-0.1497647	2.92951E-15
INTS4	0.06554295	2.93141E-15
SLC25A43	0.05540217	2.97536E-15
GPX7	0.04373848	2.98941E-15
SRC	-0.2180757	2.99653E-15
CYB5D2	0.09560572	3.00666E-15
PIEZO1	-0.0704804	3.01429E-15
SCAP	0.07065431	3.0464E-15
MESD	0.12409765	3.06453E-15
COMMD8	0.14341102	3.07728E-15
DENND10	-0.0016321	3.11196E-15
SNHG10	0.04478924	3.12327E-15
TMEM184C	0.10152626	3.13269E-15
SEC23B	0.03792301	3.1491E-15
ISCA2	-0.0226995	3.16447E-15
MBLAC2	0.07788401	3.17155E-15
AC074117.1	0.07905013	3.17255E-15
EXOSC10	0.05283832	3.20035E-15
COX11	0.11484451	3.22122E-15
MEA1	0.03128567	3.22197E-15
MTF2	-0.1451638	3.23634E-15
UHRF2	-0.0967791	3.25247E-15
PLEKHB2	-0.0649044	3.25401E-15
CTDNEP1	-0.0430702	3.28486E-15
TIMM8B	0.18314647	3.29321E-15
PBX2	0.03362997	3.29433E-15
ACYP1	0.00036218	3.30916E-15
ZFX	0.15620017	3.32349E-15
AC092069.1	0.12273134	3.35571E-15
CNPY2	0.20116282	3.35633E-15
RING1	0.02486205	3.37677E-15
COMMD7	-0.2594583	3.37801E-15
DDX60L	0.14443729	3.38541E-15
ZNF800	-0.1586216	3.42402E-15
MRPS15	0.1121011	3.43577E-15
IFNGR1	-0.2050331	3.45024E-15
ZNF561-AS1	0.10359067	3.48731E-15
ABHD3	0.03719689	3.49118E-15
SIRPA	0.09893393	3.52494E-15
RFC2	-0.0386914	3.53878E-15
ETHE1	-0.1378695	3.56714E-15
RAB3D	-0.0243167	3.56728E-15
CTNNBIP1	-0.0011007	3.57104E-15
BIN3	-0.1617549	3.60724E-15
AC007262.2	0.09867323	3.63088E-15

CHMP4A	0.14349702	3.64256E-15
PNPLA6	-0.116731	3.64352E-15
VTI1A	-0.0613278	3.65544E-15
METTL25	0.09026324	3.66807E-15
KATNBL1	0.24134859	3.68409E-15
TET3	0.06258763	3.68576E-15
WWC3	0.09296179	3.69172E-15
GTF2B	-0.1047104	3.71518E-15
CCZ1B	0.06580897	3.71774E-15
ZNF273	0.03416517	3.79085E-15
FBXO4	0.07054208	3.79773E-15
MFSD4B	0.18095824	3.80163E-15
TMEM260	0.15025395	3.85207E-15
TWF1	0.02984551	3.87252E-15
CLK3	-0.1672582	3.89815E-15
RMI1	0.11358034	3.95219E-15
PSMG3	-0.0183381	3.95298E-15
PTCD3	0.15045396	3.95591E-15
RPN2	-0.1099206	3.95606E-15
KLHDC4	0.03101053	3.96052E-15
NDC1	0.07680411	3.96913E-15
USP7	-0.0562338	3.96996E-15
SRFBP1	-0.0303498	3.98435E-15
RPL3	-0.2847137	4.03892E-15
UQCRB	0.24680414	4.04829E-15
B3GNT8	-0.2010102	4.08388E-15
SLC25A11	0.02006035	4.10007E-15
NBDY	-0.209663	4.10304E-15
TMEM177	0.06087129	4.11185E-15
FUBP3	0.08589914	4.12465E-15
AP002449.1	0.05929646	4.13414E-15
ARHGEF19	0.06545344	4.14489E-15
MXRA7	0.06678534	4.15089E-15
SMU1	0.06006404	4.15761E-15
MIRLET7BH(	-0.1470407	4.18113E-15
ROCK1	-0.3477789	4.1868E-15
NFE2L3	0.18277185	4.2424E-15
PURB	-0.0148992	4.24289E-15
AC021097.2	0.06596607	4.25893E-15
KANSL1	-0.0427281	4.28806E-15
ZSCAN30	0.1046176	4.30671E-15
DOCK5	-0.1364162	4.32441E-15
CASS4	-0.1896211	4.35766E-15
HIST1H2AK	0.11205501	4.35786E-15
DOCK1	-0.1857668	4.38357E-15
ADAM28	0.33330774	4.38864E-15
SLC35F5	0.03573468	4.41265E-15
RPL9	0.16300865	4.43018E-15
THUMPD3-A	-0.1932895	4.45506E-15
LYRM2	0.07775448	4.47911E-15

HOXA7	0.00659614	4.52865E-15
ASF1A	0.10902124	4.56158E-15
ANKLE2	-0.1504545	4.57599E-15
YEATS4	0.00755367	4.59358E-15
MPDU1	-0.0980249	4.59557E-15
SLC7A6OS	-0.0144663	4.65974E-15
DR1	-0.0155011	4.67815E-15
SLC2A5	0.11158551	4.69213E-15
DMAC2	0.04073743	4.72462E-15
PRKAG1	0.04180446	4.75783E-15
COG5	0.09160063	4.80805E-15
WDR45	-0.035145	4.82191E-15
SLC25A37	0.22670786	4.83635E-15
TNFRSF25	-0.1848212	4.85322E-15
ZBTB40	0.09211831	4.90986E-15
ARL16	-0.0596814	4.92952E-15
ANP32E	0.24729398	4.93558E-15
ZNF3	-0.1626581	4.9373E-15
DMAP1	-0.0016586	4.98454E-15
ACADS	-0.0190595	5.04034E-15
GID8	-0.0417267	5.09412E-15
GAK	-0.0204535	5.10256E-15
ZNF579	-0.1790186	5.11077E-15
AC002310.1	0.06505353	5.11308E-15
PNKD	0.17125214	5.18882E-15
CDC42SE2	-0.2422183	5.19951E-15
EXOC5	-0.0550918	5.25527E-15
RFXAP	0.0820963	5.25675E-15
EBP	0.05947863	5.35614E-15
CARD16	-0.016626	5.41623E-15
FAM122B	0.04126515	5.42255E-15
NUCB1	-0.1055229	5.42761E-15
YAF2	-0.0765688	5.43171E-15
FTSJ1	0.0183914	5.49177E-15
CYP7B1	0.12341853	5.49772E-15
ACAD10	0.06733039	5.50728E-15
NEK6	0.0676805	5.52913E-15
C9orf43	0.06681382	5.55581E-15
WHRN	-0.0884413	5.5596E-15
ZNF506	0.20857591	5.57644E-15
HAVCR2	-0.0662762	5.5831E-15
TMEM134	-0.0473338	5.61934E-15
GID4	0.0859174	5.62184E-15
UBE2E3	-0.0236527	5.65527E-15
SMCR8	0.08690653	5.68024E-15
COA8	-0.0037771	5.76555E-15
UBALD1	-0.0664166	5.7977E-15
TRANK1	0.15843092	5.88601E-15
MED4	-0.1123761	5.94962E-15
CCDC127	-0.1593701	5.97296E-15

CTBP1-DT	0.10610804	6.01377E-15
CXCR4	0.16384154	6.07745E-15
SPN	0.33622112	6.07957E-15
MPG	-0.2200888	6.08155E-15
ARL1	0.03703389	6.12291E-15
RGS14	-0.1636345	6.15423E-15
RAD51D	0.07591938	6.15962E-15
CCDC22	-0.0021097	6.16485E-15
TMEM19	0.08383097	6.18017E-15
PTCD1	0.063687	6.19521E-15
PPFIA1	0.10591813	6.19753E-15
NAA10	-0.1043634	6.24387E-15
HBS1L	0.038469	6.2507E-15
LONRF1	-0.2173031	6.25181E-15
HAUS2	-0.1510977	6.37248E-15
TCFL5	0.08093901	6.37865E-15
CDK10	0.00130923	6.38396E-15
MSL1	0.11321522	6.38519E-15
RMDN1	0.09980558	6.39235E-15
BIN2	0.1421532	6.49077E-15
ZNF74	0.09953003	6.51355E-15
MBIP	-0.0150075	6.53559E-15
WDR82	-0.1007275	6.53893E-15
EFCAB2	-0.0021696	6.55156E-15
PDE6D	0.06555307	6.5752E-15
RNASEL	0.10606332	6.60528E-15
STX4	-0.0694921	6.61463E-15
HESX1	0.07699747	6.61746E-15
FANCB	0.03849233	6.62916E-15
RBM34	0.05200672	6.69429E-15
FBXW5	-0.0447579	6.70931E-15
PTGES3	0.2840756	6.72757E-15
MAPKBP1	-0.2465089	6.74303E-15
SLC35B4	0.08068011	6.84937E-15
GPS1	-0.0062861	6.86863E-15
TAF4	0.02799587	6.92589E-15
AC027601.6	-0.1638127	6.97814E-15
THUMPD3	-0.0401499	6.98013E-15
MRNIP	-0.0052528	6.98503E-15
GTF2IRD2	0.07387915	6.98573E-15
SHARPIN	-0.0206376	7.0011E-15
SNF8	-0.0487898	7.05558E-15
NKX2-3	0.05534973	7.12078E-15
PIK3CD	0.06484539	7.15024E-15
AKR1A1	0.01378477	7.17652E-15
COX7C	0.20850406	7.42884E-15
TMEM203	0.06847118	7.43874E-15
ST6GALNAC	-0.1462343	7.44079E-15
KLC1	-0.1312799	7.45256E-15
ENG	0.08366902	7.53256E-15

AKT1	-0.0029444	7.6053E-15
NUP62	-0.0693298	7.63819E-15
HPS1	0.11726138	7.77062E-15
WDR81	0.01853248	7.77337E-15
ZC3H14	0.09898804	7.79835E-15
SLC48A1	0.04587788	7.8001E-15
FAM117A	0.25944431	7.8596E-15
EPC2	0.0626933	7.88789E-15
RNF167	-0.1217714	7.89208E-15
GDAP2	0.00587583	7.8938E-15
ERO1A	-0.0185992	8.05801E-15
RFK	-0.0554872	8.09636E-15
LINC01138	0.08428139	8.10957E-15
ANAPC7	0.06047262	8.12541E-15
CYB561A3	-0.0029274	8.22133E-15
ANKHD1	0.15550696	8.22686E-15
TMEM216	-0.0255998	8.24496E-15
PHKG1	0.09651651	8.28111E-15
CSTF2T	0.0857424	8.28742E-15
POT1	0.0819316	8.31472E-15
SNRNP70	0.06414584	8.35768E-15
SMCHD1	0.23321483	8.43905E-15
IMP4	-0.0217839	8.48581E-15
STX5	-0.1273414	8.57567E-15
PDIA3	-0.2592972	8.57789E-15
RIC8B	0.06282914	8.77725E-15
ABI2	-0.2247831	8.80577E-15
SS18	-0.0493491	8.81268E-15
SLA2	-0.0312018	8.82088E-15
NECAP1	0.01375348	8.87218E-15
FOXJ3	-0.0732275	8.88223E-15
BAG1	0.15912662	8.89839E-15
AKT2	-0.0713284	8.92706E-15
CCDC174	-0.1304888	8.96591E-15
SERTAD3	-0.2137333	8.99052E-15
CNN2	-0.0718581	9.23757E-15
SF3A1	0.02481169	9.25073E-15
BUD13	-0.0721525	9.25354E-15
KIF22	0.08691993	9.27082E-15
VAPB	-0.0896866	9.37815E-15
RMND5A	0.04122951	9.39493E-15
USP12	-0.2377196	9.46629E-15
HINT2	0.01068595	9.47014E-15
NUP93	0.00328902	9.4716E-15
GOLGA7	0.07281613	9.5097E-15
WDFY1	0.16420037	9.51098E-15
TAZ	-0.1412146	9.51312E-15
CSNK1G2	0.07491505	9.53567E-15
FZR1	-0.2105781	9.54751E-15
APPBP2	-0.1027108	9.56362E-15

RPN1	0.01414314	9.59923E-15
MAPK14	-0.0084975	9.62473E-15
TTC21B	0.05263756	9.64787E-15
VAMP4	0.08627831	9.68449E-15
PSMC6	-0.0700914	9.75599E-15
RILP	-0.0083888	9.75621E-15
PPP2R5A	0.0172357	9.78092E-15
CSNK2B	-0.2412857	9.84303E-15
SRRT	0.0622498	9.85057E-15
NFKBIE	-0.1140984	9.91752E-15
MBD5	0.03626073	1.01119E-14
SDHD	0.15080563	1.01857E-14
OSTF1	0.10676152	1.01929E-14
CNOT7	0.14833103	1.02092E-14
ABCD4	-0.045217	1.03101E-14
TXNDC15	0.04343948	1.03211E-14
ZNF557	0.0768493	1.03379E-14
IER3	-0.4788171	1.04403E-14
PTPN11	0.02034211	1.04525E-14
USP9X	-0.1481521	1.04558E-14
ZNF48	-0.1509467	1.04562E-14
GCH1	-0.2835053	1.04848E-14
TMEM106A	0.03825144	1.06988E-14
RRM1	0.07299171	1.08451E-14
TRIQK	0.07930377	1.08554E-14
TMEM147-A!	0.1064527	1.08571E-14
DNAJC13	0.1380103	1.08775E-14
NAPG	-0.1933475	1.09292E-14
PACS1	0.02603938	1.101E-14
ZBTB33	0.09858904	1.11136E-14
SIKE1	0.05299031	1.11525E-14
HOOK2	0.0380028	1.11631E-14
RAP2C	-0.0377697	1.11778E-14
CCNH	-0.0109747	1.13995E-14
SORD	0.15785269	1.14372E-14
HINFP	0.01113353	1.14688E-14
WDR18	-0.041943	1.14853E-14
KPNA6	-0.1635268	1.15079E-14
TMSB4X	0.37834258	1.15744E-14
PTEN	0.27166731	1.16631E-14
TBC1D9B	0.11259185	1.1754E-14
WASIR2	0.05763862	1.19058E-14
ZNRD2	-0.085198	1.20335E-14
AC016831.1	-0.1832734	1.20544E-14
NHP2	0.22693723	1.22205E-14
SMIM1	0.1016845	1.22266E-14
ENOSF1	0.19530033	1.2273E-14
MIEF1	0.08797759	1.23197E-14
SIL1	-0.1711724	1.24087E-14
TMEM147	0.14874997	1.24227E-14

ELOVL1	-0.0714646	1.24238E-14
WWP1	0.06255574	1.2501E-14
PDIA6	-0.0936062	1.25272E-14
RBM42	-0.1257726	1.25378E-14
EIF2B3	0.10789576	1.25549E-14
NSL1	0.08501854	1.27356E-14
ARHGEF1	-0.1358918	1.29057E-14
MDC1	0.02304134	1.30904E-14
GSK3A	-0.1306777	1.32489E-14
NPM1	0.26037806	1.33236E-14
BOP1	0.0356915	1.34271E-14
TFPT	0.05982347	1.34515E-14
MED1	0.0389618	1.34583E-14
ANKRD39	0.04079364	1.35492E-14
SPRED2	-0.1602572	1.35802E-14
EIF2B5	0.02572028	1.35806E-14
TDG	0.12426284	1.35896E-14
NCKAP5L	-0.1487018	1.37693E-14
POM121	0.0382422	1.37728E-14
BNIP2	-0.2479391	1.37758E-14
CUEDC2	0.0148135	1.38707E-14
DPY19L1	0.10542661	1.39551E-14
DCUN1D1	0.08200133	1.39558E-14
RARS2	-0.0230908	1.40655E-14
LINC00339	0.07796273	1.41044E-14
GOLPH3	-0.1276406	1.42404E-14
GPRC5D-AS	0.05395322	1.43382E-14
BDH1	0.12576046	1.45076E-14
RNF123	0.07259642	1.45359E-14
LINC00662	0.1078284	1.45988E-14
SH2D2A	-0.2217536	1.46653E-14
AC006213.2	0.0788914	1.47604E-14
ATP6V0D1	-0.1415161	1.48194E-14
LINC02802	0.11262089	1.48349E-14
SMNDC1	0.12121343	1.48932E-14
ARMC5	0.08183466	1.49445E-14
BTBD6	0.06138201	1.49632E-14
DNAJC5	-0.1287997	1.50199E-14
APH1A	-0.1182226	1.51143E-14
MYLK-AS1	0.07642811	1.51205E-14
MRPL52	0.07533608	1.52137E-14
DCK	0.17673966	1.53609E-14
CNNM3	0.09379903	1.53746E-14
PRKAR1A	-0.1436694	1.55344E-14
CDC16	0.09052919	1.55559E-14
AC139887.2	-0.0933748	1.55915E-14
DNAJB14	-0.1568336	1.56041E-14
NF2	0.05355373	1.5708E-14
RPA1	0.11303342	1.57825E-14
EPB41L3	0.08703813	1.60115E-14

RAD18	0.09529391	1.60845E-14
DGKQ	0.09585343	1.61658E-14
TUG1	0.04201691	1.63352E-14
CD109	-0.2912889	1.63699E-14
PATZ1	0.0612569	1.6477E-14
STK38	0.12169162	1.66493E-14
TNK2	-0.1203372	1.66868E-14
HGS	-0.1374631	1.68048E-14
CEP70	0.10962116	1.68526E-14
ZNF182	0.04817278	1.69785E-14
YTHDF2	-0.0089654	1.70724E-14
RABEP1	0.05208007	1.72344E-14
ZNF426	-0.002462	1.72942E-14
MAST3	0.08828882	1.74139E-14
ZDHHC20	0.05755423	1.75561E-14
BCKDK	-0.0863734	1.76136E-14
ATG4B	-0.039157	1.76901E-14
RNF187	0.12598443	1.81287E-14
AMN	-0.1870232	1.8145E-14
C1orf122	-0.0696437	1.81909E-14
GIT1	-0.006652	1.82255E-14
CHST11	-0.1111635	1.82358E-14
CCNJ	0.06593453	1.82769E-14
PRPF31	0.04865086	1.83145E-14
RBM23	0.10535674	1.84206E-14
TCEAL9	0.16875696	1.84737E-14
HTATIP2	0.11844034	1.85249E-14
BOD1	0.0510392	1.85398E-14
ACTR6	0.11623656	1.87392E-14
VPS13B-DT	0.07579823	1.88693E-14
BTK	0.16786529	1.88877E-14
SFR1	0.08930076	1.90114E-14
SLC35E1	-0.2108713	1.90761E-14
MTLN	0.09438939	1.91516E-14
CCNDBP1	0.14541127	1.93404E-14
LRBA	-0.1071501	1.93588E-14
ESRRA	0.02941209	1.94479E-14
ZNF585B	0.09358215	1.96078E-14
ENOPH1	0.06477817	1.96331E-14
ZNF655	-0.0897697	1.97023E-14
FAM50A	0.0786595	1.97492E-14
RNF220	0.1795456	1.99286E-14
PTGER4	0.13249614	2.00822E-14
ESYT2	-0.145951	2.02028E-14
SIN3B	0.04141118	2.03891E-14
CARD8-AS1	-0.0046523	2.04358E-14
PLPP5	0.02462762	2.04516E-14
CPXM1	-0.1356161	2.05426E-14
PPIE	0.09177305	2.05675E-14
PRORP	-0.0985719	2.0689E-14

ZNF28	0.0762323	2.07225E-14
TBC1D13	0.08176068	2.10237E-14
FBXL5	-0.0040948	2.12782E-14
FBXW8	0.05911571	2.13416E-14
PUM2	0.13462937	2.15016E-14
FIS1	-0.1454596	2.16101E-14
COX10-AS1	0.06668509	2.17705E-14
COASY	0.04149464	2.18719E-14
ATAD2	-0.0057861	2.19938E-14
ARHGAP25	-0.233762	2.19939E-14
TMEM167B	-0.095922	2.2141E-14
BCL2L13	-0.0418109	2.22598E-14
LRRC58	0.12800417	2.22991E-14
PRKCD	-0.101366	2.25631E-14
SLC25A25-A	0.06179662	2.25897E-14
NOC2L	0.0195759	2.2787E-14
FLYWCH1	0.06769496	2.28422E-14
TXK	0.07032233	2.29216E-14
CLPP	-0.0282854	2.30111E-14
UCK2	-0.0478709	2.3096E-14
MDH1	0.124963	2.32478E-14
SLC38A7	0.07559965	2.3266E-14
BEX4	-0.1610052	2.35905E-14
ATP8A1	0.12923274	2.36583E-14
RABL2B	0.11529535	2.38621E-14
TFIP11	-0.1218809	2.4066E-14
NIT1	0.07334158	2.40719E-14
WDR7	0.10977918	2.42992E-14
LSM10	0.02555038	2.43409E-14
PPME1	0.02031235	2.4585E-14
NFRKB	0.09028327	2.45949E-14
ZNF787	-0.0615377	2.46342E-14
NUP210	0.08293489	2.47126E-14
GFOD2	-0.1191863	2.47268E-14
AC016735.1	0.07038457	2.50964E-14
EGLN1	0.11048784	2.51642E-14
CYP20A1	0.07002507	2.5333E-14
SNRPG	-0.1309283	2.53533E-14
HSH2D	0.08574236	2.54603E-14
SNHG8	0.24085249	2.5604E-14
SUSD6	-0.0926787	2.56169E-14
UBQLN4	0.06536995	2.56694E-14
CEP95	0.02827724	2.58214E-14
GUCD1	0.01254497	2.6411E-14
INF2	-0.1754528	2.6814E-14
SEC24B	-0.1237346	2.73834E-14
AC244090.1	0.03025875	2.74225E-14
UBN1	0.01798457	2.74863E-14
RAB20	-0.1139012	2.75839E-14
NABP2	0.07739256	2.76439E-14

MRPL23	0.15983366	2.76502E-14
STAT5A	0.1257396	2.79058E-14
PLAA	-0.0590619	2.79126E-14
RNF146	0.0178463	2.79915E-14
CHD8	-0.0933865	2.81565E-14
UBE2T	0.04523822	2.82495E-14
GSKIP	-0.1348667	2.84539E-14
CDK16	-0.0671688	2.85181E-14
WDPCP	0.11242342	2.86076E-14
MNDA	0.11622948	2.86134E-14
WDR92	0.06558923	2.86461E-14
PADI2	0.06209169	2.87818E-14
AC084198.2	0.05583598	2.89016E-14
RANBP9	-0.0190075	2.92579E-14
E2F3	0.06727864	2.93165E-14
TMEM115	-0.124632	2.9423E-14
SLC38A9	0.07834998	2.94242E-14
ANKRD11	0.12313116	2.95758E-14
TRAP1	0.06616149	2.96573E-14
NAE1	0.09291743	2.96575E-14
TIGD2	0.03961373	2.97035E-14
HMG20A	-0.0080744	2.97562E-14
PRR13	0.21319225	2.98168E-14
NIFK	0.26074472	3.00007E-14
SVIP	-0.0679829	3.00398E-14
UTF1	-0.2322456	3.01709E-14
SRPRA	0.00692532	3.0187E-14
NPLOC4	-0.0108479	3.02205E-14
PLAAT4	-0.1502992	3.02474E-14
RFTN1	0.09157985	3.05197E-14
INPPL1	0.01055183	3.06871E-14
SPTAN1	-0.2129856	3.11808E-14
SDSL	0.10105953	3.12108E-14
TRIM59	0.14872464	3.14889E-14
PARP11	0.11915408	3.15315E-14
EPHA1-AS1	0.10155734	3.16274E-14
NEU1	-0.0466929	3.16592E-14
CRTC2	-0.1419735	3.16796E-14
SLC8B1	0.04155744	3.16878E-14
PYROXD1	0.06383287	3.18245E-14
GRASP	-0.1832118	3.21381E-14
PI4KB	-0.0939688	3.22332E-14
AC004148.1	0.05912791	3.23159E-14
MRTFB	0.05218011	3.29192E-14
ATF7	-0.0879325	3.29865E-14
SFT2D2	-0.0480782	3.31178E-14
APOBR	0.03428964	3.31684E-14
AC100810.1	-0.1049528	3.32539E-14
NAA20	-0.030894	3.32687E-14
WASHC2A	0.12654776	3.3401E-14

SMARCD2	-0.0695564	3.3421E-14
ASB8	-0.1576969	3.34712E-14
ZNF793	0.11075776	3.35082E-14
HSD17B10	-0.0229491	3.35306E-14
SOGA1	0.08797503	3.35494E-14
AC019069.1	0.07291091	3.37418E-14
ALDH1B1	0.051499	3.37482E-14
FHIT	0.07289099	3.38888E-14
RAB3GAP1	-0.0018063	3.40024E-14
BTBD7	-0.1601043	3.41062E-14
GTF2A2	0.16101037	3.45092E-14
ACAP2	-0.0692559	3.46179E-14
OAT	0.15601965	3.46457E-14
MZT2A	-0.1513338	3.50233E-14
PPP2R1A	-0.0220495	3.50898E-14
TMED2	-0.0428762	3.57894E-14
ODF2L	-0.0579159	3.71224E-14
EFCAB11	0.07259206	3.72383E-14
CPLANE1	0.0924353	3.74385E-14
COQ10B	0.04427116	3.746E-14
BBS2	0.02707001	3.7462E-14
KRT17	-0.3324185	3.77552E-14
THAP2	0.09948812	3.78333E-14
PUM3	0.2060603	3.84769E-14
MTPN	-0.3136273	3.87504E-14
ATRAID	0.02597087	3.88645E-14
RENBP	0.07732885	3.88731E-14
SNX29	0.05485184	3.88897E-14
TIGD1	0.05799335	3.96918E-14
C5orf22	0.03707581	4.01476E-14
SKP1	0.30020415	4.02626E-14
MRPL43	0.00738992	4.03208E-14
RPF1	-0.0025452	4.03903E-14
ZNF277	0.09970499	4.05028E-14
TYW1B	0.0843247	4.11114E-14
MMRN1	0.11240849	4.11554E-14
TAF4B	0.08344705	4.13244E-14
LYRM4	0.01662509	4.18437E-14
SORT1	0.00196529	4.18586E-14
TSR2	-0.1000816	4.20966E-14
NIT2	0.17604067	4.23555E-14
ZNF227	0.04596842	4.30078E-14
FAM13A-AS1	0.03920073	4.31375E-14
RPS6KA1	0.02950378	4.31865E-14
GAMT	-0.0682526	4.32755E-14
N4BP2L1	-0.0340695	4.33277E-14
ZNF337	-0.009596	4.35194E-14
BRWD3	0.06915192	4.43278E-14
STMP1	0.3061774	4.44675E-14
STRIP1	-0.0243765	4.46474E-14

PATL1	-0.0702903	4.53222E-14
NPM3	0.04411391	4.53944E-14
HIGD1A	-0.0518473	4.55169E-14
CIR1	-0.2755684	4.58495E-14
MAGED1	-0.1052919	4.59366E-14
UQCC1	0.07880172	4.59675E-14
ST3GAL3	0.09743726	4.62359E-14
FUT8	0.07552368	4.63372E-14
KIAA0930	0.16452403	4.64148E-14
PSMB5	0.02813864	4.67063E-14
SCO1	0.08507809	4.69883E-14
SQSTM1	-0.4424014	4.72285E-14
SP3	-0.035326	4.74349E-14
HELQ	0.0749995	4.8021E-14
ERLIN2	0.04878224	4.81746E-14
DUSP11	-0.0957229	4.82453E-14
SPATA5	0.1111282	4.83686E-14
RBFA	0.07139733	4.85994E-14
MAP3K11	-0.2185657	4.87607E-14
PGGT1B	0.12756348	4.91089E-14
SH3D21	0.10568767	4.91899E-14
TM2D3	-0.1204722	4.91911E-14
RANBP1	0.10305841	4.95548E-14
EIF2B4	0.06801585	4.97183E-14
TYMS	-0.3073828	4.97532E-14
ATF6B	-0.0006057	5.03707E-14
UTP4	0.07214004	5.05096E-14
TTC7A	0.01792512	5.0561E-14
KAT5	-0.127501	5.0836E-14
PPP1R11	0.01163379	5.11623E-14
C5orf56	0.03418375	5.14574E-14
SHQ1	0.08580422	5.16154E-14
ZBED4	0.05209965	5.18478E-14
CYB5B	-0.0358482	5.20639E-14
P3H1	0.06203772	5.21861E-14
WBP2	-0.0240841	5.22086E-14
NME4	0.09310056	5.24067E-14
GALNT11	0.1433823	5.2855E-14
ZNF740	0.04679129	5.2915E-14
CUL5	-0.1390596	5.29369E-14
OSBPL9	-0.0562164	5.29843E-14
PKM	-0.1826462	5.30671E-14
AP1S1	0.09686642	5.32528E-14
QRSL1	0.10565045	5.32983E-14
AP002387.2	0.09983612	5.33464E-14
ETFDH	0.03991162	5.36673E-14
CYFIP1	0.1251749	5.38193E-14
CHMP1B	0.01064091	5.38725E-14
FADS3	-0.1890856	5.4095E-14
CCL1	-0.2017087	5.44234E-14

ACAD9	0.04062184	5.47879E-14
MAP2K4	-0.1151223	5.47892E-14
RAB40C	0.07909716	5.48401E-14
UBE3C	0.10524906	5.51577E-14
NUDT21	-0.0016108	5.57786E-14
RBM33	-0.115954	5.58474E-14
TOR3A	0.0323122	5.67644E-14
ACAP1	-0.2203532	5.72512E-14
CATSPER2	0.11415573	5.75837E-14
RHOT2	-0.0282407	5.79637E-14
TMEM120B	-0.0954157	5.7964E-14
AP1M1	0.00129918	5.81694E-14
PGRMC1	-0.1111477	5.83534E-14
MRPL17	-0.0109989	5.87269E-14
GTDC1	0.13127083	5.991E-14
SYTL1	-0.1782685	6.01605E-14
NOL11	0.0298249	6.01633E-14
FNTA	0.05156157	6.01679E-14
TSTA3	-0.1149344	6.06684E-14
STIP1	0.06955209	6.08079E-14
FGR	-0.1728293	6.09098E-14
VAV3	0.01317523	6.12799E-14
PIP4K2A	0.1457204	6.16191E-14
UBE2J2	-0.0981369	6.2132E-14
PREP	0.09550642	6.23403E-14
PRNP	-0.1746266	6.25474E-14
MPHOSPH6	0.05543247	6.35171E-14
PUS3	0.11787122	6.37753E-14
PSMD10	0.02428974	6.38542E-14
JRKL	0.12045573	6.48804E-14
TOP3B	0.03523551	6.51696E-14
GEMIN4	0.07991604	6.56415E-14
NCLN	-0.0503529	6.5715E-14
TMEM160	0.04997347	6.59028E-14
GTF3C2	0.02168409	6.60177E-14
TATDN3	-0.0173177	6.60676E-14
NAP1L4	0.1086972	6.68902E-14
HIPK2	-0.063267	6.7274E-14
PCNX1	0.09537823	6.73046E-14
DST	0.18258658	6.73841E-14
TP53INP1	0.11440841	6.77473E-14
DELE1	0.04711097	6.77908E-14
ZNF358	0.0912358	6.78668E-14
EID1	-0.2171273	6.78719E-14
COX16	0.16388024	6.78948E-14
FTL	0.29249858	6.79734E-14
GDI1	-0.1288736	6.81048E-14
HK1	-0.1394437	6.85793E-14
PRR14	-0.0888918	6.88841E-14
ARHGAP1	0.03111499	6.91593E-14

MMGT1	0.06425498	6.98415E-14
SDR39U1	-0.0747011	7.00081E-14
ZNF354A	0.10758873	7.00538E-14
EIF2AK2	0.13993749	7.04715E-14
CD47	-0.2727573	7.0788E-14
CYSTM1	-0.2230952	7.17963E-14
BRAP	-0.049566	7.22475E-14
TNFAIP3	0.01358385	7.2814E-14
ZSCAN29	0.06416537	7.28158E-14
AL359644.1	0.05212455	7.29271E-14
NAT14	-0.1147698	7.29876E-14
ZNF562	0.06241246	7.30758E-14
UBL7-AS1	0.06170193	7.37633E-14
GUSB	-0.10623	7.42172E-14
AC007383.2	0.07340967	7.4264E-14
DDB1	0.01216512	7.47117E-14
ELOA-AS1	0.06196235	7.49527E-14
FAM102B	-0.170224	7.51456E-14
TUBB2A	-0.1456387	7.51801E-14
AP2A1	0.01918083	7.64329E-14
EXT2	0.02539983	7.69842E-14
TXLNA	0.0509899	7.71566E-14
STUB1	0.0344558	7.73705E-14
PRDM8	-0.1634624	7.83409E-14
KXD1	0.06523355	7.86355E-14
ZKSCAN2-D'	0.09588723	7.87543E-14
CERT1	-0.2421775	7.88009E-14
LMF2	-0.1509284	7.88581E-14
RAI1	-0.1056638	7.90223E-14
PKP4	0.12624074	7.90837E-14
DDX49	-0.0400504	7.91777E-14
ZNF621	0.04967839	7.96712E-14
VAMP2	-0.0545024	7.97624E-14
ZNF92	0.10865776	7.97872E-14
SETMAR	0.06069647	7.98822E-14
WNK1	0.07411525	8.03859E-14
PAIP1	0.06981658	8.04352E-14
PREX1	0.05054531	8.05257E-14
LTA4H	0.26069432	8.08406E-14
RNASEH1	-0.0501736	8.09377E-14
PARP1	0.21631186	8.10117E-14
UBL4A	-0.1485992	8.13608E-14
PSMC2	-0.031146	8.14356E-14
GANC	0.06797852	8.14519E-14
RNF41	-0.0719049	8.23227E-14
TIMM10B	-0.1347453	8.27347E-14
ITPK1	0.09715464	8.28707E-14
AC011447.3	0.0897938	8.29692E-14
VEZF1	-0.1824548	8.30279E-14
LGALS9	0.09458835	8.31256E-14

FIBP	-0.0466858	8.34254E-14
ZCCHC3	0.0724582	8.35211E-14
GEMIN7	0.00753643	8.35652E-14
FAM78A	-0.0131889	8.42563E-14
CDC14A	-0.2201357	8.45808E-14
ZNF641	0.07271794	8.52745E-14
NDRG3	0.04406375	8.54539E-14
ZNF354B	0.09145872	8.5618E-14
IFNAR1	-0.0079087	8.60368E-14
SDHA	0.02374873	8.64646E-14
USP53	-0.0490212	8.65174E-14
MFNG	0.01185703	8.68449E-14
KLHDC8B	0.0517654	8.71536E-14
SMG6	-0.0106946	8.72354E-14
MRPS17	0.10111305	8.76202E-14
NUSAP1	0.05853321	8.80643E-14
PGBD2	0.06030515	8.86361E-14
TNPO2	0.03686445	8.88651E-14
RNF111	-0.1109035	8.95684E-14
EPM2A	0.08389579	9.05702E-14
TP53I11	-0.1545833	9.23451E-14
CBLL1	0.01741071	9.2461E-14
SEC61A1	-0.0624641	9.27881E-14
ZNF606	0.06423472	9.28334E-14
HLA-DMA	-0.2477491	9.30062E-14
SRA1	-0.1647808	9.46318E-14
BCAP29	0.02252752	9.54104E-14
MARCH5	-0.0277123	9.59159E-14
ILVBL	0.01641888	9.6854E-14
MED15	-0.1367073	9.69728E-14
SURF4	-0.0801247	9.81052E-14
HIF1A-AS3	0.06752033	9.826E-14
PRRC1	0.01146169	9.8366E-14
AC011043.1	0.08132531	9.92191E-14
GNG10	-0.0082227	1.0132E-13
P2RY11	0.04903044	1.01505E-13
SCAF1	-0.0594768	1.01773E-13
FAM234A	0.06406135	1.01814E-13
AK9	0.12555768	1.02161E-13
JPT2	-0.0376191	1.02553E-13
NUDT14	0.112541	1.02635E-13
BICRAL	0.11330217	1.03093E-13
ARIH2	0.0105484	1.03607E-13
OSTC	0.22582381	1.03901E-13
CARHSP1	-0.1671726	1.05029E-13
MT1X	0.05214465	1.05733E-13
AC016773.1	0.03309961	1.08249E-13
HECTD4	0.0374082	1.08539E-13
SNHG19	0.109259	1.09185E-13
TOLLIP	-0.1028748	1.0945E-13

TUBA1B	-0.5753349	1.10063E-13
ARHGEF40	0.03143339	1.10091E-13
FDPS	0.05131494	1.10201E-13
NDST2	-0.1632286	1.10313E-13
AC118549.1	0.11191957	1.11734E-13
MTFR1L	0.05654245	1.11793E-13
SYNGR2	0.10765651	1.12047E-13
PPP2R5C	-0.0526194	1.12579E-13
CPVL	0.26837848	1.13411E-13
E2F4	0.02414028	1.13798E-13
SNRNP35	-0.0421066	1.15843E-13
AL354740.1	0.08029869	1.15875E-13
OXNAD1	0.06639756	1.16265E-13
PARP10	0.04451666	1.1668E-13
NOM1	0.07204193	1.16737E-13
BNIP3L	-0.1452091	1.17127E-13
PRIMPOL	0.09719379	1.17685E-13
FOXRED1	0.0305463	1.17719E-13
TCERG1	0.08838957	1.18027E-13
RAP2B	0.0117344	1.19191E-13
ATF2	-0.0719995	1.19399E-13
ZNF528-AS1	0.11911773	1.19403E-13
CBWD3	0.09472143	1.20296E-13
SPEN	-0.0244304	1.20358E-13
METTL21A	-0.0097947	1.20414E-13
LDLRAD2	-0.2065411	1.20677E-13
ZNF594	0.0786962	1.20717E-13
CCDC124	-0.0160375	1.21022E-13
ACAP3	-0.1335732	1.21369E-13
SIRT2	-0.1207895	1.22132E-13
IDH3B	-0.0418917	1.22603E-13
TOMM70	0.06696637	1.2328E-13
SEC63	0.13046869	1.23659E-13
REX1BD	-0.0625362	1.2381E-13
PRMT7	0.09000009	1.24595E-13
PCYT2	0.08106948	1.25337E-13
CENPW	-0.0405913	1.2573E-13
SASS6	0.13199536	1.25767E-13
SUGT1	0.13111934	1.26274E-13
DCAF16	0.03364603	1.29415E-13
TSPAN32	-0.169367	1.30389E-13
ZNF274	-0.0606997	1.30425E-13
SPRYD4	0.07122819	1.317E-13
FUNDC2	0.0327637	1.31821E-13
SF3B1	-0.2952477	1.32568E-13
GABPB1-IT1	0.0165011	1.32594E-13
ATP6AP1	-0.0157152	1.33334E-13
SCARF1	-0.1321504	1.33671E-13
POLR2M	0.04405368	1.34593E-13
TEX2	0.05512818	1.35039E-13

HSPBAP1	0.06434027	1.35735E-13
ZNF717	0.06960577	1.36589E-13
TRIM11	-0.0713315	1.37156E-13
CDC27	-0.0876762	1.38771E-13
HTRA2	-0.0015093	1.3891E-13
CNOT10	-0.0110994	1.4114E-13
KMT5B	-0.0423354	1.41244E-13
CDK12	-0.0529024	1.41672E-13
RAB8A	-0.0093265	1.41783E-13
GPANK1	-0.0144007	1.41992E-13
CAB39	-0.0418895	1.42159E-13
SDCCAG8	0.23329529	1.4357E-13
MCTP2	-0.0600767	1.43852E-13
CDK13	0.15700316	1.44046E-13
MIB2	-0.0282416	1.44065E-13
ZNF654	-0.0253317	1.44142E-13
MAVS	-0.0674561	1.45451E-13
ISY1	0.04691047	1.46077E-13
DGUOK	-0.0272739	1.46289E-13
KIFAP3	-0.0588313	1.47609E-13
PTCD2	0.07859295	1.47849E-13
ACAA1	-0.1128119	1.47959E-13
ZNF394	-0.1246398	1.48149E-13
NBEAL1	0.08624582	1.4824E-13
POC1B	-0.0759025	1.49481E-13
IRAK2	-0.1412225	1.50149E-13
ZNF529-AS1	0.07149698	1.51002E-13
AZIN1	0.00759966	1.51258E-13
CTSF	0.03320955	1.52159E-13
TIPRL	-0.022093	1.53004E-13
NDUFB10	0.20400883	1.5311E-13
SMYD4	0.07535861	1.53962E-13
NIPSNAP3A	0.02696425	1.54417E-13
VPS25	0.03060371	1.56734E-13
MRPL2	0.05017225	1.5719E-13
GLRX2	-0.0737724	1.57933E-13
TFB1M	0.04031263	1.58055E-13
MOB2	-0.0703059	1.5866E-13
NEDD1	-0.0013105	1.59755E-13
CERS5	-0.0167198	1.6112E-13
SNRNP48	0.0274845	1.62199E-13
NCOA5	0.06872069	1.64472E-13
AC027097.1	0.0983742	1.64986E-13
AC016747.1	-0.1409826	1.65328E-13
ELAVL1	-0.0279689	1.65383E-13
LIN54	-0.0005573	1.65535E-13
AC093157.1	0.08749837	1.66463E-13
CRELD2	-0.1236213	1.6737E-13
ELOA	0.02359925	1.68043E-13
FUNDC1	0.10043108	1.69225E-13

TDP2	-0.0063673	1.69506E-13
FAM53C	0.05081012	1.69795E-13
CCDC57	0.04574582	1.69842E-13
FAM210A	-0.0480048	1.70225E-13
TMLHE	0.02646909	1.70622E-13
TMEM91	-0.161491	1.72491E-13
OCIAD2	-0.1319563	1.73026E-13
COPS7B	0.00221927	1.73416E-13
COX19	-0.0608811	1.74079E-13
DSTYK	-0.2288822	1.74195E-13
TRIOBP	-0.0929805	1.78434E-13
TMEM159	0.09406658	1.78572E-13
UGGT2	0.06379277	1.78612E-13
DIPK1B	0.08000447	1.79245E-13
HELLPAR	0.15336909	1.79614E-13
INPP5D	0.15746403	1.83697E-13
CISD3	0.07835741	1.8376E-13
CTBP2	-0.1030832	1.84734E-13
USPL1	0.06427595	1.85332E-13
CHMP3	-0.0238271	1.86658E-13
ZNF345	0.06592154	1.88363E-13
TMC8	0.0867055	1.89079E-13
TCEAL1	0.1176391	1.89261E-13
RAB11FIP2	0.11830057	1.90065E-13
IDNK	0.14606689	1.90168E-13
ABHD17A	-0.1846955	1.90572E-13
ELF4	-0.1781763	1.90958E-13
ZNF395	-0.1983623	1.90998E-13
GLE1	0.07664445	1.91135E-13
GSDMD	0.0148063	1.94375E-13
MAP2K5	0.03564526	1.94858E-13
THADA	0.15089595	1.96451E-13
RAB9A	0.07690665	1.97335E-13
SRPK2	0.10176923	1.98159E-13
JAGN1	-0.0795786	1.99871E-13
ARL5B	0.05328591	2.00022E-13
ST3GAL2	-0.0154231	2.01831E-13
KRBA2	0.05631934	2.04539E-13
HMBOX1	0.08334949	2.0523E-13
KIF20B	0.20479943	2.05547E-13
CUL3	-0.1581633	2.06836E-13
PHC3	0.04113897	2.07578E-13
SARNP	-0.1402836	2.08337E-13
SUN2	0.00195103	2.09303E-13
WIZ	0.01294986	2.09643E-13
EEF1AKMT3	0.06750803	2.10127E-13
CROT	0.10870158	2.10725E-13
PQBP1	0.12658412	2.11455E-13
CDK5	0.04850318	2.12956E-13
RFWD3	0.01497084	2.13535E-13

GPBP1L1	-0.0817599	2.14525E-13
R3HCC1	-0.0079111	2.14987E-13
LRRCC1	0.13285812	2.16133E-13
YKT6	-0.037974	2.16921E-13
WDR43	0.25511194	2.17103E-13
SUPV3L1	-0.0894474	2.17562E-13
LINC00863	0.05559363	2.17735E-13
IRF2	-0.0020987	2.18079E-13
NUDT5	-0.0274482	2.18562E-13
CINP	0.00524002	2.18828E-13
AC093297.2	0.07855306	2.20641E-13
DYM	0.10614087	2.22113E-13
DNM2	-0.0006657	2.23796E-13
ACSL5	0.09390898	2.24479E-13
STX7	0.23126162	2.25439E-13
TCHP	0.08378256	2.2583E-13
TMEM167A	-0.1266181	2.26146E-13
CCNG2	0.04168798	2.27325E-13
DCAF6	0.00522926	2.28411E-13
CYP1B1	0.25167635	2.28512E-13
STK26	0.22705579	2.30574E-13
ADPGK	0.02819132	2.31347E-13
USP34	0.16145876	2.34451E-13
AEBP2	-0.0214758	2.34452E-13
NIP7	-0.0520945	2.34938E-13
NDST1	0.06567016	2.35452E-13
TIMM50	-0.0018558	2.35817E-13
MBD6	-0.0040261	2.40102E-13
MACO1	-0.0409716	2.40368E-13
PIGU	0.04685892	2.44164E-13
NOCT	0.06723541	2.4752E-13
AP2M1	-0.1553241	2.51774E-13
AC008267.5	0.06300599	2.5184E-13
CWC22	-0.1197953	2.53817E-13
MBD3	0.09408287	2.55163E-13
FAM120C	0.04639872	2.55861E-13
PSENEN	0.02247019	2.56036E-13
TUBD1	0.03258084	2.56661E-13
SLC25A28	-0.0358318	2.57134E-13
DCUN1D5	-0.0685497	2.57682E-13
GAR1	0.1277519	2.58207E-13
CHD1L	0.09270418	2.5883E-13
HDAC10	-0.0021462	2.59475E-13
SLC45A3	0.18524554	2.61542E-13
ELF2	-0.1365739	2.62147E-13
MRPL55	0.1363107	2.62193E-13
LRRC8D	0.0077418	2.62207E-13
TPR	0.2678547	2.64591E-13
IFT22	-0.1523293	2.65164E-13
AC010618.3	0.08627332	2.66522E-13

UBE2E1	-0.0442392	2.66731E-13
YIPF1	0.04948544	2.67276E-13
ATP1B3	0.04064315	2.69918E-13
BCL7A	0.05180695	2.69923E-13
POP7	-0.0074898	2.70251E-13
INO80D	0.08108604	2.70322E-13
AKAP7	0.10244196	2.70423E-13
LTBP4	0.08448457	2.71283E-13
BRD2	-0.1154625	2.71526E-13
ELP6	0.05179232	2.72816E-13
PMPCA	-0.064367	2.73863E-13
RPP38	-0.0526904	2.76935E-13
JDP2	-0.0036327	2.79387E-13
ZFAND1	0.15027563	2.85299E-13
IMPDH1	0.00857822	2.87409E-13
RPP14	0.06426672	2.89101E-13
TRAPPC2L	0.03589096	2.89911E-13
NCAPD2	0.08464682	2.90045E-13
NRM	-0.1511418	2.90372E-13
AL133453.1	0.03837913	2.91394E-13
WBP1	-0.1071128	2.92934E-13
ZNF875	0.03738206	2.94791E-13
PLTP	-0.1349272	2.98785E-13
PINX1	0.09814375	2.99313E-13
FAM117B	0.08560205	2.99712E-13
COX17	0.06748085	3.00404E-13
LINC02019	0.02819482	3.02292E-13
ABRAXAS2	-0.1171004	3.04031E-13
GATM	0.06662142	3.05425E-13
NUTM2B-AS	0.13914361	3.06582E-13
NOL9	0.03763649	3.09321E-13
LINC00539	0.05574905	3.15557E-13
DHRS4-AS1	0.08985817	3.15559E-13
CAPRIN2	0.07920386	3.19903E-13
ZFR	-0.1549881	3.22235E-13
HSCB	0.14915918	3.22983E-13
ETNK1	0.1555098	3.24387E-13
ATP6V1H	-0.0308567	3.25734E-13
EFNB1	-0.1366007	3.258E-13
MXD1	0.14769825	3.27247E-13
AP3D1	0.05424363	3.28577E-13
ADAR	0.00163464	3.30514E-13
HSPA4	0.00678785	3.3107E-13
RNF217	0.06087523	3.33922E-13
ZNF567	0.05487024	3.349E-13
SNU13	-0.2280901	3.36081E-13
ELMOD3	0.06496818	3.38103E-13
YIPF5	-0.0767781	3.40115E-13
PKN2	-0.1512533	3.404E-13
GBF1	0.00525252	3.42554E-13

RALGPS2	0.06497058	3.4438E-13
PTS	-0.1756516	3.45602E-13
CUL9	0.01770918	3.46544E-13
MLLT6	0.06832346	3.46822E-13
SNHG17	0.11095618	3.51705E-13
SLC39A7	0.00011738	3.52158E-13
INO80B	0.05591862	3.55213E-13
BX664615.2	0.04800197	3.555E-13
TIMM29	-0.0102203	3.57478E-13
BBS10	0.06086794	3.59381E-13
C2orf68	0.03419967	3.59776E-13
ZNF689	0.00238534	3.61633E-13
PLD1	0.13236187	3.62168E-13
CSTF3	0.07578777	3.63257E-13
TSEN15	0.05197386	3.64586E-13
MBTPS1	0.09729588	3.68466E-13
AC005921.2	-0.002752	3.68482E-13
AIMP2	0.06305163	3.71449E-13
DRG2	0.05921594	3.73698E-13
LPIN1	-0.1515989	3.74199E-13
LPCAT4	0.03291032	3.74686E-13
CUTA	0.23005028	3.76314E-13
TPRG1L	-0.0754272	3.79851E-13
TMEM101	0.09656255	3.81664E-13
ESF1	0.22906277	3.82836E-13
TMED4	-0.1053412	3.82974E-13
CAMK2G	0.03645795	3.83639E-13
HAUS7	0.07545208	3.84549E-13
AAAS	0.04899442	3.88655E-13
RBM15B	-0.0861156	3.89193E-13
TRIM47	-0.1007209	3.90507E-13
RAB35	-0.1223225	3.92151E-13
IER5	-0.2323981	3.94535E-13
PSMD3	0.00794696	3.98897E-13
PIGT	-0.1245349	4.04231E-13
PRKCB	-0.0547827	4.05261E-13
N4BP2L2-IT2	0.07409724	4.05753E-13
GABARAPL1	-0.1083877	4.0707E-13
KCNC4	0.13476909	4.07796E-13
SETX	-0.0778135	4.08617E-13
SUPT6H	-0.0993565	4.09981E-13
RAB27A	0.20424564	4.12799E-13
TGS1	0.02450256	4.14844E-13
ZDHHC24	-0.011168	4.15059E-13
VHL	-0.0141478	4.15799E-13
YIPF2	-0.0436423	4.17685E-13
THOC5	0.06667455	4.23349E-13
TAS2R4	0.09523177	4.23785E-13
CD55	-0.2511275	4.29943E-13
NAGLU	0.01488465	4.32139E-13

HMOX1	0.0777334	4.3299E-13
BRAF	-0.2149013	4.3326E-13
PRDM16-DT	0.05783883	4.36309E-13
CCDC32	0.01774153	4.44533E-13
GOLGA3	0.06699591	4.45935E-13
MTERF1	0.06949672	4.5002E-13
BORCS5	0.08411627	4.50381E-13
POLR2J	0.09538313	4.512E-13
MRPS7	-0.0445162	4.51829E-13
NEPRO	0.06543582	4.53661E-13
TMEM179B	0.02558369	4.62947E-13
DDRGK1	-0.0375277	4.6377E-13
ZNHIT1	0.1433248	4.64658E-13
ICE1	0.00035538	4.65566E-13
KLHDC2	-0.0283151	4.66618E-13
SMDT1	-0.1020753	4.76245E-13
MINPP1	0.08860504	4.76897E-13
MYCBP	0.09370102	4.7842E-13
HMGN4	-0.0442823	4.80462E-13
BMT2	0.03299747	4.84321E-13
ANKS1A	-0.0510949	4.88374E-13
FOXK2	0.02254798	4.90973E-13
ALG10	0.05267261	4.91419E-13
PEX1	0.06889594	4.96257E-13
LUC7L2	-0.1404276	4.98114E-13
PAPOLG	-0.0011523	5.17587E-13
DDX59	0.00631003	5.17977E-13
PDE4D	-0.0643748	5.18129E-13
ZDHHC4	0.05669549	5.22214E-13
NEMP1	0.0920509	5.22862E-13
FBXL4	0.07243602	5.33776E-13
POLR2J2	0.03321479	5.3513E-13
PIK3CA	-0.1694777	5.36291E-13
PARVB	-0.1445637	5.39901E-13
COQ7	0.01712495	5.39922E-13
FBXO7	-0.1506696	5.40908E-13
TCF3	0.09416529	5.45898E-13
FBXO38	-0.0522426	5.45958E-13
GGA3	0.02182192	5.46782E-13
VMA21	0.10796972	5.46855E-13
SNHG5	0.25310934	5.47236E-13
KIN	-0.0282447	5.48216E-13
NAA60	-0.100602	5.49613E-13
TYK2	0.00690631	5.49649E-13
ATP7A	0.12685822	5.50019E-13
MXD4	-0.2064178	5.53801E-13
EPDR1	-0.0351694	5.54403E-13
FIG4	0.07359565	5.54866E-13
FAM162A	0.15216601	5.58063E-13
RPP30	0.03045499	5.58576E-13

POLG	-0.0095758	5.59819E-13
PDXP	0.08035536	5.61538E-13
NR2F6	0.08333174	5.62216E-13
ZFPL1	-0.0254608	5.63418E-13
FASTK	-0.0814034	5.63949E-13
ZDHHC7	-0.0930272	5.65406E-13
SNIP1	-0.0289151	5.66626E-13
HDDC3	0.08419336	5.73587E-13
GRAMD4	0.06870772	5.77356E-13
EPC1	0.12282513	5.82727E-13
MPRIP	0.05802476	5.85961E-13
PALB2	0.07145098	5.86793E-13
FPGS	-0.0364712	5.91588E-13
NKAP	0.09354424	5.9214E-13
TCEANC2	0.13211597	5.95025E-13
DFFA	0.13906161	5.9698E-13
CCDC138	0.0437054	5.9711E-13
CCDC180	0.07863539	5.97121E-13
LINC01500	0.05725514	5.99072E-13
AC093227.1	0.0583501	6.14376E-13
NFKB2	-0.2266762	6.18922E-13
GATAD2B	0.02505537	6.19522E-13
SIRT5	0.08371604	6.21402E-13
POLR2K	0.03843679	6.23961E-13
CHORDC1	0.07470471	6.26382E-13
TRIM65	0.05105401	6.28648E-13
FAM114A2	0.10993851	6.38775E-13
TRMT1	0.00494319	6.40757E-13
PTPN18	0.08262347	6.40838E-13
MROH6	0.07149969	6.43493E-13
ZFAND2B	0.02992377	6.44968E-13
FSD1L	-0.180001	6.46501E-13
TMEM250	-0.0407684	6.46603E-13
FBXO45	-0.040176	6.52225E-13
CCDC173	-0.1349127	6.53341E-13
TEDC1	-0.1554658	6.53562E-13
EI24	-0.0359646	6.56645E-13
BCR	-0.0625734	6.58438E-13
CLSTN3	-0.023463	6.62555E-13
SWI5	0.06793983	6.64658E-13
ZKSCAN3	0.03823113	6.68852E-13
SAP30	0.03992917	6.69101E-13
GLCE	0.08560877	6.69699E-13
LEMD2	-0.0181138	6.71866E-13
LPCAT1	0.07885061	6.76943E-13
IFT43	-0.0400869	6.78493E-13
SLC25A13	0.00762784	6.81112E-13
SLC25A16	0.01337425	6.82889E-13
MTMR14	-0.0350129	6.84423E-13
MDM2	0.09299944	6.8644E-13

C12orf75	-0.2448742	6.87139E-13
ING2	-0.018416	6.92989E-13
STX18	0.03101304	6.93507E-13
CLASP1	0.0973771	6.95263E-13
PXN-AS1	0.07745539	6.96885E-13
MRPS35	0.00246038	6.98057E-13
TRIM33	-0.1021364	6.99674E-13
MYO1F	-0.0955477	7.01261E-13
LSM12	-0.0587999	7.06156E-13
GATB	0.05304898	7.09633E-13
CHMP5	0.22623867	7.10937E-13
ZNRD1	0.09691385	7.11705E-13
FAS	-0.1468085	7.14667E-13
HYAL3	0.09998423	7.15056E-13
MRPS23	0.05398507	7.17434E-13
SCCPDH	0.19818953	7.18915E-13
CLCN3	-0.0039764	7.19164E-13
PTGIR	-0.1941603	7.26122E-13
MDP1	-0.0035464	7.26616E-13
CCDC28B	-0.1374492	7.30409E-13
MHENCR	-0.1278709	7.3208E-13
COX5B	-0.2255594	7.37484E-13
LNX2	-0.0723078	7.39684E-13
UBR3	0.08694165	7.39904E-13
SLC7A1	-0.0663577	7.4063E-13
DCAF15	-0.1325448	7.45247E-13
ANKRD16	0.08946153	7.46978E-13
C2orf49	0.05811235	7.52695E-13
ERGIC3	0.05810161	7.53485E-13
ZNF687	-0.0099512	7.58085E-13
FUT7	0.08022007	7.58728E-13
TWISTNB	-0.0685116	7.62409E-13
IL9R	0.05933731	7.68175E-13
NMD3	0.00389408	7.68907E-13
NUP188	0.08000303	7.73981E-13
BICD2	-0.1408923	7.78071E-13
PPIL2	0.0160695	7.79966E-13
ANAPC10	-0.0163635	7.80129E-13
ASAP1	0.10874654	7.85198E-13
ARRDC3	-0.2316946	7.87813E-13
METTL18	0.13151955	7.89229E-13
MFSD10	-0.1688162	7.96174E-13
ZBTB6	0.05910597	7.96482E-13
FAM133B	-0.2045984	7.98137E-13
TRAF6	-0.0741835	7.98613E-13
TAF8	-0.002892	8.09289E-13
ZC3H12C	0.01942602	8.09722E-13
SYNRG	0.09827264	8.10982E-13
AC107464.3	-0.0476578	8.12346E-13
FANCL	0.08467685	8.15565E-13

RFC5	0.05208149	8.15674E-13
TMEM71	-0.1091116	8.31313E-13
TLR1	0.03107511	8.43988E-13
RABGAP1L	0.02638318	8.44034E-13
KDM3A	-0.0588862	8.48485E-13
COX14	-0.0580379	8.50597E-13
APOE	0.06891385	8.52057E-13
NUDT22	0.04246582	8.54004E-13
FRMD4B	-0.2555382	8.55423E-13
UNC119	0.00388183	8.59053E-13
CNIH1	-0.0586011	8.59966E-13
BRD4	-0.086425	8.6216E-13
AP3M1	0.05483619	8.63424E-13
ZBTB14	0.07490753	8.68941E-13
OXA1L	0.06591394	8.70068E-13
FKBP7	0.06206835	8.71334E-13
ISYNA1	-0.0650138	8.71777E-13
DDX60	0.07000053	8.72879E-13
ATP5PB	-0.2250561	8.77353E-13
COPS6	-0.0995433	8.82601E-13
MRPS24	0.15218387	8.84007E-13
MGAT4B	-0.168622	8.85738E-13
IFIT5	0.04679389	8.88738E-13
AC090114.2	-0.1185096	8.91388E-13
POLR1E	0.09851908	8.92866E-13
CRNDE	0.13916425	9.00267E-13
DDX41	0.07415858	9.04286E-13
ZNF160	-0.1169297	9.09065E-13
LRRC47	0.07364103	9.09675E-13
BRD1	-0.1046209	9.21878E-13
CMAS	0.0160632	9.25217E-13
VRK1	0.10422024	9.26626E-13
UBL3	-0.0378328	9.29855E-13
MRPL42	0.18904735	9.33578E-13
CLIC4	-0.1645161	9.37591E-13
NEK1	0.08717637	9.4744E-13
POLDIP2	0.00735843	9.51584E-13
PCBD2	0.13056244	9.68113E-13
VAV1	0.02506202	9.71792E-13
RMDN3	0.02879834	9.79685E-13
MLF1	-0.1130795	9.85907E-13
BRPF1	-0.0590553	9.86306E-13
SPOPL	-0.0099138	9.86709E-13
BLOC1S6	0.12838297	9.90393E-13
DNAJA3	0.0752682	9.93237E-13
GSR	0.07766473	9.95847E-13
BCL2L2	0.07120503	9.98593E-13
TMBIM1	-0.1715725	9.99335E-13
DDB2	-0.1295915	1.00348E-12
MFSD14B	0.03959687	1.00604E-12

POLB	-0.0190618	1.00808E-12
GLA	0.03128279	1.01333E-12
DCTN4	-0.1635711	1.01339E-12
ETAA1	0.08066665	1.01659E-12
DIP2B	0.08201156	1.02873E-12
SPOUT1	0.07969066	1.02968E-12
RAB5B	0.0381868	1.03531E-12
SLC4A1AP	0.0740079	1.0371E-12
SUMF1	0.0638296	1.03714E-12
SLC35D1	0.03447654	1.04162E-12
ARHGAP45	-0.0834669	1.042E-12
EDRF1	0.05988546	1.04584E-12
MOAP1	-0.0603723	1.0478E-12
FAM104A	-0.0260157	1.05019E-12
SLK	-0.1072003	1.05303E-12
XIST	0.34373772	1.05595E-12
XPNPEP1	-0.0762032	1.06221E-12
XAB2	-0.1023194	1.08056E-12
C12orf76	-0.0384994	1.08341E-12
TDP1	0.06071433	1.08374E-12
SPRY2	0.07525368	1.09701E-12
PHAX	-0.0341092	1.09985E-12
MCTP1	-0.0698494	1.10058E-12
ZNF616	0.09127881	1.10597E-12
TBXAS1	0.08908481	1.10659E-12
HNRNPDL	-0.2828899	1.1167E-12
CERCAM	0.04091757	1.11948E-12
FAM102A	0.07988045	1.12313E-12
PEX5	0.04838341	1.12395E-12
MUS81	0.04845865	1.12897E-12
PPIL1	0.05963336	1.13249E-12
KIAA0319L	0.10706599	1.1344E-12
CENPB	0.05242431	1.1347E-12
C11orf71	-0.0344595	1.13884E-12
RCC1L	-0.0021943	1.1413E-12
TRAPPC10	0.02219625	1.14938E-12
FLCN	-0.0178616	1.15298E-12
NEXN	0.15795132	1.15715E-12
CCDC69	-0.1415234	1.16325E-12
FBRSL1	0.08608884	1.17212E-12
RPRD1B	-0.1841799	1.17397E-12
KCTD10	-0.1283353	1.17955E-12
AHCYL2	0.06309266	1.18215E-12
PCCB	0.00832419	1.18321E-12
FAM241A	-0.1272421	1.18678E-12
BECN1	0.03358794	1.20208E-12
TMEM9	0.05099365	1.20329E-12
TK2	0.03627634	1.20504E-12
ZNF12	-0.0220262	1.21086E-12
IPO9	0.08060698	1.23353E-12

TNFSF12	0.08491252	1.23848E-12
PHTF2	-0.1134148	1.23912E-12
AP3S2	-0.0543396	1.24106E-12
POM121C	0.00864306	1.25413E-12
USP24	0.09381604	1.26689E-12
THNSL1	0.06178618	1.28097E-12
HOPX	-0.3744752	1.28177E-12
TEC	0.01412289	1.28324E-12
HNRNPL	0.00497796	1.288E-12
MBD1	0.03209937	1.29976E-12
ABI1	-0.1863545	1.30518E-12
INTS14	-0.0065098	1.30581E-12
MAP2K7	0.01312346	1.31055E-12
NSD3	0.15976839	1.32056E-12
SNRPB	-0.2603002	1.32087E-12
ZNF385A	-0.1982696	1.32363E-12
HELZ	-0.1447394	1.32819E-12
SLC25A32	-0.0118022	1.33446E-12
EXOSC3	-0.0735932	1.3428E-12
MPV17L2	0.03215068	1.3491E-12
GOLGA5	-0.0043884	1.3493E-12
THAP9-AS1	-0.0976778	1.35452E-12
YIF1B	-0.061677	1.36718E-12
FLAD1	0.05165238	1.38558E-12
DAXX	-0.0884645	1.39602E-12
SETDB1	-0.0127607	1.3983E-12
UBA5	0.12166455	1.41642E-12
STK11IP	-0.0406511	1.41918E-12
COG6	0.11820007	1.42004E-12
RPS6KC1	0.0708733	1.42501E-12
STK38L	-0.0394154	1.42836E-12
NIPBL-DT	0.1693515	1.43583E-12
BSDC1	-0.108515	1.43815E-12
ZNF85	0.1191294	1.44895E-12
AC103591.3	-0.3175608	1.45752E-12
BICD1	0.08103513	1.45846E-12
NFE2L1	-0.1448919	1.46631E-12
CEP57L1	0.1534502	1.47203E-12
SAP30L-AS1	0.05290661	1.4763E-12
TMEM70	-0.0401092	1.47829E-12
TPSB2	0.07999213	1.48967E-12
KLF3-AS1	-0.1791326	1.49549E-12
LIMK1	-0.089845	1.50447E-12
STRN3	-0.0297609	1.51311E-12
PSTPIP1	-0.1903923	1.51927E-12
ZMIZ2	-0.0042516	1.52047E-12
TMEM170A	-0.0623636	1.52262E-12
YAE1	0.05225805	1.54545E-12
PLCB2	-0.0132724	1.54986E-12
TAX1BP3	0.09505311	1.55476E-12

ZC3H18	0.03157755	1.5598E-12
RPRD2	0.03706084	1.55985E-12
CABIN1	-0.1478789	1.56166E-12
DBF4	0.12098751	1.56213E-12
NPHP3	0.03502771	1.5663E-12
NDUFA12	-0.087776	1.57662E-12
PCED1A	0.00188309	1.57665E-12
QTRT1	-0.1074056	1.57772E-12
ATPAF2	0.00804583	1.57989E-12
SCAF4	-0.0662846	1.59455E-12
KIAA0232	-0.2382493	1.5957E-12
PPP1R14B	-0.2798378	1.61687E-12
LITAF	-0.1628837	1.62338E-12
SNHG3	0.18020637	1.62989E-12
RNF8	0.03191037	1.63123E-12
SRP72	0.23453779	1.63321E-12
TEFM	0.09045557	1.63375E-12
SURF2	0.01242704	1.64504E-12
RBBP5	0.04479284	1.65078E-12
CLIP4	0.0857661	1.65119E-12
DNAAF5	0.07021451	1.65487E-12
GVQW3	-0.0107002	1.65737E-12
TARS2	0.09711589	1.67193E-12
UQCRHL	0.07676085	1.67742E-12
CNOT3	-0.0158332	1.68282E-12
POLR2A	-0.0418375	1.68754E-12
SNAPIN	0.02479358	1.68824E-12
TMCO3	-0.0482864	1.69845E-12
B3GALNT1	0.08049527	1.6991E-12
LGALS8	0.09258845	1.71616E-12
BACH1	0.00577711	1.71957E-12
HGSNAT	0.01380205	1.72966E-12
AKNA	-0.0493769	1.73501E-12
PPM1G	-0.0153527	1.73906E-12
ALG5	-0.0575239	1.74262E-12
AC009403.1	0.0680445	1.7663E-12
UBE2O	0.0386381	1.77218E-12
DPEP2	0.02136788	1.77225E-12
C18orf25	-0.0805287	1.77307E-12
YARS2	-0.0355795	1.78643E-12
AC144521.1	0.04512025	1.78709E-12
TRMT6	-0.0302131	1.79823E-12
COQ4	-0.0441441	1.80031E-12
RAD23A	-0.0151905	1.81042E-12
PRPF38A	-0.089854	1.81283E-12
STMN3	-0.0787872	1.83047E-12
PRKCH	-0.1564626	1.83206E-12
SLC66A3	0.07465556	1.84122E-12
ATG2A	0.03198574	1.84351E-12
NUMA1	0.12400675	1.85697E-12

IDH3A	0.06388626	1.86001E-12
DCUN1D3	-0.0622811	1.86446E-12
QDPR	0.10376956	1.86844E-12
UMODL1-AS	0.08161265	1.87084E-12
CENPU	-0.0491963	1.9209E-12
DYRK1A	-0.2211922	1.92325E-12
ARL3	-0.1132635	1.94042E-12
EXOC1	0.02570313	1.95298E-12
TMEM219	-0.0080763	1.95353E-12
APOBEC3G	-0.0338405	1.96191E-12
DUSP3	0.14595321	1.96807E-12
CBX6	0.0399649	1.97651E-12
PAPOLA	-0.1654614	1.9819E-12
VPS13D	0.05810818	1.98516E-12
KCTD21-AS1	0.08942749	1.9868E-12
ZNF574	-0.070595	1.98976E-12
MAP3K2	-0.0325652	2.0186E-12
OSBP	-0.1190802	2.03077E-12
SGMS1-AS1	0.06839351	2.0491E-12
ZNF692	0.07473469	2.05912E-12
SUCLG1	0.03887049	2.06386E-12
POLR3C	0.09075232	2.06482E-12
OTUD6B	0.15621832	2.06515E-12
EEPD1	-0.1388512	2.06691E-12
ERLEC1	0.06243631	2.07593E-12
CDK17	-0.1275816	2.08724E-12
AC019080.1	0.0656224	2.08949E-12
SNAPC5	0.0409376	2.10421E-12
ATXN2	-0.246071	2.10919E-12
ABL2	-0.0425682	2.11198E-12
QRICH1	-0.0280328	2.13099E-12
OTULIN	0.10385319	2.13115E-12
FBXW2	-0.0151283	2.14127E-12
EPB41	0.22232469	2.14602E-12
AL133243.3	-0.1274205	2.14675E-12
HAUS6	0.05155397	2.15516E-12
PDPR	0.03916476	2.15903E-12
SFI1	-0.0758544	2.17006E-12
LTV1	0.04736182	2.17182E-12
ANKRA2	0.06606251	2.23256E-12
TMEM164	0.0524608	2.23776E-12
KEAP1	-0.0262539	2.24822E-12
NPIPB2	0.0837159	2.25088E-12
PCCA-DT	0.0610706	2.26712E-12
SLC25A26	0.04054286	2.3036E-12
EZH1	-0.0222361	2.32969E-12
TUBGCP5	0.04061024	2.33171E-12
LUC7L	-0.0121762	2.33566E-12
CERNA2	0.05968655	2.35297E-12
SENP6	0.23300023	2.35611E-12

DHX32	0.09880003	2.3564E-12
ERO1B	0.077143	2.36322E-12
UBFD1	0.03922077	2.38256E-12
LINC01082	0.06118091	2.38713E-12
ZNF460	0.05857132	2.393E-12
PRIM2	0.04326336	2.41083E-12
AC104596.1	0.05748705	2.41478E-12
GIT2	0.07771329	2.42416E-12
AC110285.2	0.02673292	2.44194E-12
ATP1B1	-0.2662107	2.45828E-12
NUP88	0.02792499	2.4673E-12
RAB18	-0.0322723	2.49221E-12
GPCPD1	-0.0573277	2.49669E-12
RIOX1	-0.0064819	2.52349E-12
ECI2	0.07025713	2.54692E-12
RAB44	0.10586092	2.54966E-12
NFKBIB	-0.1548451	2.55367E-12
NAB1	-0.1499642	2.56545E-12
ATG4D	-0.1369974	2.60594E-12
COBLL1	-0.2685171	2.61506E-12
PSMB3	-0.146774	2.62442E-12
UBXN7	-0.1720853	2.62501E-12
ST6GAL1	0.08527082	2.65273E-12
CALU	0.08756742	2.65315E-12
SMC5	-0.0618979	2.65608E-12
CIC	-0.1092069	2.6667E-12
RSU1	0.12514894	2.6705E-12
SUCLA2	0.03584076	2.68544E-12
OS9	0.08830727	2.70273E-12
ZNF559	0.08987511	2.70469E-12
PARP9	0.00706698	2.7062E-12
RAB34	-0.1457236	2.70787E-12
SMS	0.10427766	2.71804E-12
BORCS7	-0.0162931	2.73083E-12
MRPL49	-0.1001249	2.74136E-12
TTC9C	-0.0100716	2.75121E-12
SNHG21	-0.0620695	2.77188E-12
ZNF281	0.06887998	2.77732E-12
PROSER1	0.01480098	2.78066E-12
VPS29	-0.0444334	2.79263E-12
PIP4P2	-0.0126234	2.7933E-12
SRPK1	0.07443072	2.79652E-12
UCKL1	-0.0574726	2.80634E-12
P4HB	-0.1108083	2.82564E-12
AC022973.5	0.08315965	2.8611E-12
ATP11C	0.08608749	2.86542E-12
SACS	-0.016665	2.87724E-12
YTHDF1	-0.0218871	2.87873E-12
ZBTB25	0.01872696	2.91562E-12
ATP2C1	0.13702631	2.92226E-12

CETN2	-0.0799386	2.94804E-12
RWDD4	-0.0476382	2.97174E-12
EMILIN2	-0.0592923	2.99396E-12
IER3IP1	0.01314777	3.00349E-12
OPA1	-0.1365262	3.01961E-12
LENG9	0.09344461	3.03434E-12
BCL2A1	-0.1131524	3.05938E-12
DDX39B	-0.0646048	3.0619E-12
ZDHHC9	0.04804387	3.07188E-12
GTF2H2	0.11321366	3.07668E-12
DUT	0.05841408	3.08533E-12
ZER1	-0.0361493	3.09209E-12
ECHS1	0.13677983	3.09327E-12
CAND1	-0.0897506	3.11378E-12
VPS16	0.01147284	3.13065E-12
FN3KRP	0.06734355	3.1495E-12
SLC26A1	0.07145579	3.155E-12
POMP	-0.2091611	3.17106E-12
ATXN7	0.0080853	3.17263E-12
FOXN3	-0.0302392	3.17487E-12
MIR222HG	-0.0237594	3.17551E-12
SH2D3C	-0.1801148	3.19457E-12
DCAF11	0.07518563	3.19543E-12
EP400	0.14678017	3.21148E-12
AC242426.2	0.05268039	3.22824E-12
CRYZ	0.05002253	3.22859E-12
MOSPD3	0.04389126	3.23419E-12
PLGRKT	0.12863935	3.24477E-12
RHBDD1	0.05155581	3.25636E-12
LPGAT1	0.04698682	3.26061E-12
TELO2	0.02408374	3.29386E-12
KMT5A	0.00792308	3.32929E-12
ZNF660	0.12282535	3.32939E-12
ZNF184	0.07460606	3.35909E-12
LHPP	-0.0348309	3.37646E-12
ZNF257	0.08088623	3.39202E-12
RITA1	0.07850924	3.39563E-12
FBXO28	-0.0838872	3.39818E-12
MPPE1	0.13881293	3.40611E-12
VPS39	0.02438239	3.41447E-12
CNOT8	-0.0028163	3.44055E-12
PIK3IP1	0.05695922	3.48348E-12
TRNT1	0.00830407	3.48926E-12
EFHC1	-0.0076768	3.50937E-12
CSTF1	0.01930753	3.51069E-12
SGSM3	-0.0540022	3.53953E-12
BTBD1	0.04221767	3.5427E-12
TASP1	-0.0643154	3.57963E-12
MOCS2	0.09129075	3.58195E-12
YDJC	0.00827079	3.58951E-12

TSIX	0.08950785	3.5946E-12
SAMD11	0.06988164	3.61301E-12
DOCK8	0.13881477	3.61739E-12
MIPEP	0.07161615	3.6614E-12
TAF11	-0.0789493	3.66514E-12
MED17	-0.0329777	3.70334E-12
ZNF33B	0.15711944	3.72863E-12
PRPS1	-0.0478423	3.74792E-12
PSMC3	-0.0327929	3.74897E-12
ZNF213-AS1	0.09453689	3.75155E-12
GLRX5	0.03452988	3.76117E-12
GTF2F1	-0.0682219	3.76459E-12
COQ6	0.02412182	3.80125E-12
MSL2	0.11961383	3.82913E-12
AIDA	-0.1326028	3.85724E-12
IQCB1	-0.0857125	3.87882E-12
EIF6	-0.0954496	3.88834E-12
ACSS1	-0.052532	3.9208E-12
PEX26	0.14011034	3.92919E-12
ETV3	-0.0310126	3.9311E-12
CCDC136	0.1169537	3.93267E-12
DDX54	0.04772746	3.93716E-12
TMEM123	0.24336853	3.9468E-12
GHDC	0.10321215	3.95314E-12
GRIPAP1	-0.0842645	3.96649E-12
PHF8	0.05375051	3.98497E-12
NRIP1	-0.0360262	4.00137E-12
PIAS1	-0.0967785	4.01366E-12
PRPF39	0.0460687	4.02221E-12
ENOX2	0.10545662	4.04797E-12
CCDC97	-0.121427	4.05908E-12
TMEM161B	0.07894954	4.07623E-12
CDC40	-0.0985545	4.11113E-12
UNKL	0.06385772	4.11287E-12
LSM5	0.1619734	4.13094E-12
VPS50	0.0732099	4.13825E-12
CCDC88C	-0.1412485	4.19318E-12
MAML1	0.06389914	4.19907E-12
MRTFA	-0.1452873	4.20451E-12
MYSM1	0.13401564	4.21328E-12
PDGFC	-0.0817973	4.25839E-12
ZBED5	0.03760204	4.26138E-12
ZNF701	0.04351478	4.26403E-12
TMEM107	0.00853588	4.27012E-12
WDFY4	0.09509889	4.27415E-12
TUT4	0.17971513	4.27724E-12
SNX10	-0.1528859	4.28655E-12
NME6	0.04408519	4.29193E-12
LAMTOR2	0.1125787	4.29779E-12
XIAP	-0.042615	4.30918E-12

VPS18	-0.0482776	4.31637E-12
CCDC25	0.09615632	4.31881E-12
NUFIP1	0.06572602	4.33192E-12
LYSMD3	0.03183192	4.35884E-12
CCDC7	0.05498295	4.39411E-12
STIMATE	-0.0394362	4.40196E-12
ZNF441	0.04096533	4.41767E-12
C6orf120	-0.0423388	4.42118E-12
SLC2A1	-0.2321485	4.42597E-12
RNF6	0.0139763	4.45053E-12
ZNF593	0.07637309	4.45234E-12
AC007038.2	0.08848751	4.47395E-12
DDX28	-0.0118268	4.4913E-12
DYSF	-0.0568704	4.50582E-12
CWC27	0.10699907	4.55637E-12
MSANTD4	0.07282887	4.59285E-12
YOD1	0.09392925	4.60966E-12
METTL16	0.00285137	4.63193E-12
COX7A2L	0.17744535	4.6779E-12
MKNK1	-0.0537058	4.69671E-12
PSMC1	-0.1008205	4.70814E-12
DGKG	0.03782658	4.7237E-12
HERPUD2	-0.0651492	4.74904E-12
ZNF326	-0.205078	4.79569E-12
DENND5A	-0.0145152	4.83753E-12
EID2	0.05800429	4.87543E-12
MTM1	0.09938988	4.89186E-12
PDPK1	-0.0507465	4.93468E-12
MSRB2	0.17646832	4.94124E-12
AL391069.2	0.1382753	4.97503E-12
DYNLRB1	0.20586102	5.03801E-12
DCAF13	0.04851766	5.07017E-12
USE1	0.0426216	5.11014E-12
CCNT1	-0.0366932	5.12753E-12
LINC01637	0.09031133	5.14434E-12
ASXL1	0.08522821	5.15057E-12
ADCY7	-0.0811228	5.15393E-12
C19orf18	0.05818434	5.17288E-12
RESF1	0.14163428	5.22199E-12
FUT11	0.00620268	5.22984E-12
MAN2A2	0.14641026	5.2805E-12
ATAD2B	-0.0440356	5.28894E-12
NCOR2	0.06559986	5.30641E-12
CAPZB	-0.2033666	5.36138E-12
CHEK2	0.09720118	5.36636E-12
CYCS	-0.0738962	5.43303E-12
SEC23A	-0.0566128	5.43342E-12
SLC12A7	-0.1899085	5.46228E-12
BANF1	0.13282742	5.48402E-12
ZNF564	0.03954806	5.4951E-12

RCE1	-0.0779853	5.5189E-12
AC016876.1	0.04576822	5.58773E-12
ZNF550	0.05431254	5.65204E-12
ADIPOR2	-0.1046135	5.67682E-12
TRIM22	0.11626579	5.68964E-12
KBTBD2	-0.1378503	5.74732E-12
RPL10	-0.1897156	5.82036E-12
EED	-0.0344294	5.84905E-12
SLC25A30	0.04342799	5.88986E-12
BIK	0.06180862	5.89498E-12
CRIPT	0.07604054	5.89884E-12
SMARCA4	0.10631023	5.9084E-12
MYO5A	0.06842173	5.91491E-12
PIK3C2A	0.06080584	5.95453E-12
C15orf40	0.12831039	5.98858E-12
CEBPB	-0.1146623	6.00176E-12
NUP205	0.03790243	6.06603E-12
AVL9	-0.0427491	6.09184E-12
ILF3	0.04005423	6.1628E-12
THEM6	0.07146282	6.18276E-12
EIF4E3	0.13954526	6.19813E-12
SPATS2	0.10684129	6.20209E-12
WASL	-0.0213568	6.2257E-12
PTRH2	0.08770182	6.25653E-12
BLZF1	-0.0934412	6.28633E-12
ANKMY2	-0.0455979	6.30862E-12
USP32	-0.0314609	6.34932E-12
POLK	-0.1306165	6.35104E-12
SDF4	-0.0562575	6.38447E-12
ZFAND5	0.07819263	6.46995E-12
RPAP3	0.07634255	6.5011E-12
RAB2B	-0.0031251	6.50594E-12
VCP	-0.1763884	6.53919E-12
MRPL37	-0.1041322	6.54145E-12
RER1	-0.0043508	6.54426E-12
CARS2	0.04684545	6.56442E-12
ZNF736	0.06315265	6.59533E-12
MAD2L2	-0.0408913	6.60666E-12
CDT1	-0.2642342	6.6569E-12
NAPRT	-0.1170793	6.69217E-12
LINC01341	0.05278222	6.71376E-12
IARS2	0.03971916	6.76617E-12
TENT2	-0.0051448	6.76937E-12
RYBP	-0.1203357	6.82998E-12
DNAJC16	0.10110291	6.90904E-12
ZNF577	0.08303286	6.97993E-12
SLC27A5	0.06447421	6.98891E-12
HSPG2	-0.0712245	6.99167E-12
ZNF623	0.04169481	7.02245E-12
TMED7	-0.0042958	7.02773E-12

ASB3	0.05110737	7.0282E-12
AL390728.5	0.06116953	7.04242E-12
LINC00685	0.05840699	7.0848E-12
DDX50	0.1292742	7.08909E-12
MIR762HG	0.05779467	7.12568E-12
CDKN2AIP	0.04496211	7.19053E-12
AL035530.2	0.07656734	7.20172E-12
CYFIP2	-0.1913802	7.31955E-12
ZNF888	-0.0606912	7.33734E-12
UBA7	-0.0812413	7.37688E-12
CXorf38	-0.0562589	7.4062E-12
SUOX	0.05274862	7.43404E-12
LCORL	0.15069001	7.46065E-12
PPDPF	-0.2253554	7.46517E-12
TIMM23B	-0.06108	7.53169E-12
KIZ	0.08169593	7.5444E-12
TMEM87A	-0.0083028	7.63803E-12
LINC00847	0.00158243	7.65157E-12
PLEKHA8	0.06215118	7.6712E-12
PARP14	0.01559352	7.71084E-12
MIA2	0.13766472	7.72513E-12
PLPP6	0.05377338	7.82256E-12
TRNAU1AP	-0.0942872	7.90232E-12
AC119396.1	0.03431147	7.91922E-12
DPM3	0.12147087	7.93116E-12
AC096711.2	0.04533056	7.93628E-12
DENND4B	-0.0166282	7.93838E-12
GRWD1	-0.0082677	7.94757E-12
AC108863.2	0.04083705	8.09741E-12
WWP2	-0.0152713	8.14911E-12
EEF2	0.20637158	8.152E-12
C16orf72	-0.1260468	8.15394E-12
SPATA33	-0.099279	8.1803E-12
PPP4R3A	0.0776245	8.19618E-12
ADNP	0.05523487	8.29991E-12
GPT2	-0.0570673	8.30686E-12
CD82	-0.2563206	8.3178E-12
MITF	0.08774614	8.31831E-12
ZNF234	0.05036417	8.33271E-12
PITPNB	-0.0114068	8.39203E-12
SPPL2A	-0.0870296	8.3926E-12
SLC9A3-AS1	0.04495941	8.45817E-12
AC044849.1	-0.1877788	8.48457E-12
A1BG	-0.2212501	8.51831E-12
ZNF263	0.00224719	8.64075E-12
NDUFS4	0.15302446	8.66419E-12
GRHPR	-0.1499192	8.71337E-12
ULK1	-0.0263372	8.79431E-12
DHRS1	-0.0394395	8.79597E-12
MANBAL	-0.0903773	8.81365E-12

UBA6-AS1	0.06844199	8.82015E-12
MESP1	0.07771744	8.84557E-12
GMIP	-0.0618283	8.86629E-12
BABAM2	0.01646412	8.98897E-12
PCLAF	-0.4395713	8.99436E-12
MCCC1	0.03337058	9.04654E-12
ZBTB41	0.08410362	9.12955E-12
ANKRD46	0.09240477	9.16348E-12
FAM135A	0.09379348	9.28598E-12
UBXN4	-0.2109829	9.3083E-12
HAGH	0.07654858	9.36442E-12
ARID1A	0.01912467	9.37081E-12
INTS6-AS1	0.07313508	9.4138E-12
ANKRD13A	0.03578091	9.4826E-12
SF3B6	-0.2291002	9.49413E-12
ATP1A1	-0.0982421	9.55244E-12
SQLE	-0.1882582	9.57196E-12
AC211476.6	0.05197836	9.57465E-12
KDM1B	0.05789258	9.716E-12
AURKAIP1	0.1005424	9.74787E-12
BTBD8	0.05419951	9.82677E-12
FH	0.06779022	9.84188E-12
TPRA1	0.03900507	9.8918E-12
MT-CO3	-0.2081649	1.00453E-11
CMC2	0.01328916	1.00665E-11
PRMT1	0.00625878	1.00885E-11
NAAA	-0.1318499	1.01761E-11
TFRC	-0.2237818	1.02E-11
POLR3D	0.09545356	1.02462E-11
CAVIN1	-0.1608341	1.03719E-11
AC104825.1	0.03630602	1.04075E-11
C9orf78	0.1314214	1.04505E-11
MRPS25	0.11653042	1.05033E-11
LHFPL2	-0.0803727	1.05584E-11
NAT10	0.13351353	1.05915E-11
WDR34	-0.0699697	1.06701E-11
BX284668.5	0.07723867	1.07123E-11
FOXN2	-0.1221735	1.07275E-11
AC016831.7	-0.1417277	1.07317E-11
PPP1R26	0.05318474	1.07337E-11
ZNF268	0.03512809	1.07675E-11
NOTCH2NL/	0.04183997	1.0787E-11
AC008969.1	0.11945683	1.08778E-11
SAMD8	-0.1334185	1.0886E-11
POLE4	0.04650778	1.09204E-11
MRPS6	-0.093777	1.09313E-11
RASSF7	-0.0318765	1.09428E-11
TMEM38B	-0.0760831	1.10571E-11
BRAT1	0.01809372	1.1076E-11
CASP8AP2	0.09010896	1.11314E-11

DYNC2LI1	0.06570512	1.12242E-11
CENPH	0.0074018	1.13234E-11
OSBPL8	0.24928597	1.13402E-11
DHX38	0.02320519	1.13802E-11
BISPR	0.07196123	1.15751E-11
CASP3	-0.158339	1.16113E-11
LMBRD1	0.0460522	1.16233E-11
MARCH7	-0.0008465	1.16277E-11
LRP5	0.07559371	1.16887E-11
GNB5	0.10060734	1.17729E-11
REL	-0.0817994	1.19433E-11
CMTR1	0.03733705	1.19541E-11
KDM4B	0.0247742	1.19594E-11
KATNB1	-0.1055776	1.19899E-11
COQ2	0.06372878	1.19914E-11
ZNF783	0.05796769	1.21036E-11
WDR45B	0.14103655	1.21384E-11
PIP5K1A	-0.0316108	1.21424E-11
NCBP3	0.17535025	1.2228E-11
Z99127.4	0.03100599	1.22699E-11
ECHDC2	-0.0054396	1.22923E-11
ANAPC15	-0.0890244	1.23138E-11
ZC3H11A	0.05630759	1.23218E-11
ZNF417	0.07188763	1.23981E-11
DDX20	-0.0438073	1.24199E-11
PEX16	-0.0859392	1.24322E-11
TRIM21	-0.0193873	1.24869E-11
NSUN4	0.01841323	1.25082E-11
NUP37	0.06773573	1.26259E-11
POFUT1	0.0857913	1.27646E-11
ARSA	-0.0799617	1.28854E-11
C16orf91	-0.0075767	1.29003E-11
OBSCN	0.04764035	1.29095E-11
TIMM8A	0.02530373	1.29584E-11
APOBEC3C	0.09934821	1.29874E-11
MEGF8	0.07328754	1.29941E-11
CCDC85B	0.16531519	1.31256E-11
RAMAC	0.02675268	1.31555E-11
ENTPD3-AS	0.0582913	1.32282E-11
GOLGA8Q	0.07377703	1.32542E-11
RNF115	-0.0009749	1.32675E-11
AHCTF1	-0.016774	1.33368E-11
CRYZL1	-0.0518113	1.33592E-11
PROK2	0.07446069	1.34808E-11
C1QBP	0.27674558	1.35117E-11
TEX261	0.0202933	1.37478E-11
PRKCSH	0.03298036	1.37575E-11
PLCXD1	-0.0145129	1.3759E-11
LINC01215	-0.1505312	1.39019E-11
SLC19A1	0.04898475	1.39621E-11

ANKRD26	0.12903149	1.39779E-11
RAP1GDS1	0.01252587	1.39888E-11
BCS1L	0.01956689	1.40111E-11
KBTBD3	0.05683923	1.40966E-11
TCIRG1	-0.1221001	1.4103E-11
PPP2R5E	0.05097674	1.41506E-11
UBR7	0.00969051	1.41575E-11
BTBD3	-0.0472533	1.41864E-11
KIF9-AS1	0.00819533	1.41901E-11
PDK3	0.03568817	1.46759E-11
STXBP2	-0.0435893	1.47218E-11
ERCC2	0.0631004	1.47551E-11
MTRR	0.04039683	1.48591E-11
RRM2B	0.0413072	1.4862E-11
TICAM1	-0.1716846	1.48774E-11
SMN1	0.06258113	1.49003E-11
SCRN1	-0.2032488	1.49171E-11
ZNF765	0.07927751	1.49448E-11
RBM48	0.01378275	1.49574E-11
MRPL4	0.07673541	1.50568E-11
SAMSN1	0.17772758	1.50773E-11
TRIM37	0.04633964	1.52656E-11
EIF4EBP3	0.1154105	1.52669E-11
CANT1	-0.0432486	1.54369E-11
GPATCH8	-0.0313898	1.54451E-11
ZMYM6	0.0829051	1.55089E-11
APPL2	0.03616575	1.5515E-11
DYRK4	0.03362514	1.55397E-11
SLC41A3	-0.0126448	1.55555E-11
NBR1	0.1291348	1.55715E-11
ZNF93	0.11125897	1.56886E-11
EHD4	-0.1356477	1.57257E-11
SYNE1	0.08160628	1.58621E-11
TRIM58	0.00599771	1.58871E-11
PSMD13	-0.1057701	1.59604E-11
MOCS3	0.01768667	1.59732E-11
TRAF3	-0.1491836	1.60392E-11
L3MBTL1	0.0673098	1.60773E-11
FAR2	0.01599504	1.61504E-11
MOGS	0.03990843	1.63296E-11
WAC	-0.1575979	1.63418E-11
SNN	-0.1492956	1.64973E-11
FAM53B	0.07767878	1.66738E-11
NAPA	-0.1027425	1.67421E-11
SLC35A4	-0.0122739	1.67775E-11
MTMR4	0.05810467	1.68665E-11
SYT11	-0.1978197	1.68723E-11
REPS1	0.0496679	1.71056E-11
MAMDC2	-0.1315375	1.71396E-11
SPHK1	-0.1458265	1.7277E-11

REEP3	0.08105054	1.74658E-11
LINC00476	0.10489091	1.75049E-11
MTA1	-0.0256126	1.75539E-11
RAB7A	-0.1033296	1.75668E-11
RERE	-0.0216243	1.75906E-11
ACPP	0.00610392	1.77657E-11
ZNF548	0.0361149	1.80172E-11
GPD2	0.0522661	1.80798E-11
ZFP90	0.06731918	1.83252E-11
AL031595.3	-0.1357874	1.88189E-11
GTF2F2	0.00659955	1.88235E-11
GCAT	0.0781692	1.88308E-11
WAPL	-0.0264367	1.89334E-11
TBC1D2	0.03397397	1.89699E-11
CR381653.1	0.04527767	1.89893E-11
COPS9	0.23057624	1.934E-11
MROH1	0.05513363	1.95036E-11
PLEKHM2	-0.0107691	1.9511E-11
NGRN	-0.1363052	1.95152E-11
MADD	0.0478206	1.9557E-11
MEX3C	-0.0976862	1.96497E-11
NCOA7	-0.0006572	1.96868E-11
DUSP22	-0.0076553	1.9687E-11
SENP2	-0.0390864	1.96885E-11
VOPP1	0.01345322	1.96979E-11
FIGNL1	0.0595734	1.98559E-11
TBCE	0.01781331	1.98571E-11
RASGRP2	-0.1095976	1.99494E-11
NAA30	-0.0042316	2.00945E-11
CFDP1	0.22255325	2.0101E-11
UBE2K	0.01467283	2.02038E-11
FAM3C	-0.0688058	2.03903E-11
AC008124.1	-0.0076303	2.04275E-11
ATG5	-0.0603553	2.08564E-11
DEDD	-0.1551847	2.09729E-11
CYBC1	0.04164241	2.10026E-11
FAM41C	0.03319573	2.12091E-11
LRMP	0.00905503	2.12346E-11
SPECC1L	0.0500783	2.13159E-11
CHCHD4	0.05903389	2.13978E-11
CCND2	0.00396443	2.15762E-11
PUM1	-0.1552451	2.17268E-11
SAP130	0.05834166	2.18059E-11
IRAK1BP1	0.06480417	2.1824E-11
LRP10	-0.1964051	2.19029E-11
GPKOW	-0.0316516	2.20675E-11
GEMIN5	0.07594673	2.22258E-11
P4HTM	-0.0773061	2.2274E-11
PICALM	-0.0372883	2.24483E-11
SCRN2	-0.0258764	2.2503E-11

EEF1AKMT2	0.0682202	2.25078E-11
BAZ1B	0.04033026	2.25881E-11
TMEM126A	0.02836254	2.26845E-11
RALA	-0.1286017	2.26942E-11
SLC25A15	0.04467431	2.27034E-11
VTI1B	0.04073639	2.27486E-11
FASTKD3	0.04787517	2.2844E-11
SHMT1	0.07300478	2.2865E-11
PRTN3	0.31154861	2.29243E-11
NCOA6	0.08968284	2.30635E-11
SEL1L	-0.0411092	2.31288E-11
FAM122A	0.02114556	2.33096E-11
CHD6	0.04885351	2.36342E-11
NDUFS6	0.17650052	2.36414E-11
DEGS2	0.057246	2.3715E-11
RNF24	0.18276143	2.39116E-11
CRACR2A	0.04580464	2.4238E-11
TBK1	0.00118572	2.44435E-11
CHRAC1	0.03739748	2.44691E-11
SMAD7	0.01481793	2.44947E-11
BATF	0.02478772	2.47374E-11
TSPAN33	-0.0277122	2.47884E-11
UNC5B	-0.0412579	2.48096E-11
PSPH	0.07215564	2.48565E-11
OGFOD3	0.05344636	2.48901E-11
PSMG2	-0.086481	2.50037E-11
ZNF544	0.10235779	2.52162E-11
VMAC	-0.0255564	2.52798E-11
CLPX	0.07067393	2.52945E-11
TMED5	-0.0633134	2.55119E-11
IMMT	0.06327568	2.55191E-11
IFIT3	0.05961756	2.56123E-11
IMMP2L	0.06993099	2.5619E-11
CAPN10	-0.0450229	2.57733E-11
PTK7	0.04267359	2.58348E-11
H2AFZ	-0.1605948	2.58365E-11
STX3	-0.1270109	2.59929E-11
ATP13A3	0.00255697	2.60022E-11
TNFSF13	-0.1179819	2.61225E-11
IFT74	0.13658634	2.62167E-11
KARS	0.0768396	2.63161E-11
UMAD1	0.05246434	2.63202E-11
BRF2	-0.0096943	2.66546E-11
ZFP62	0.06214621	2.67508E-11
BROX	0.14177507	2.68713E-11
NAA15	-0.0731883	2.68946E-11
USP1	-0.0381171	2.70212E-11
FO393401.1	0.02388591	2.74688E-11
MYH11	0.063115	2.77435E-11
TNRC6A	-0.0293612	2.78678E-11

LINC01578	-0.2367868	2.80932E-11
RTN2	0.06718085	2.81122E-11
RNH1	0.08268716	2.83762E-11
MRPS18B	0.02571498	2.83804E-11
AKAP11	0.09092796	2.85969E-11
TRAPPC2B	-0.0379881	2.87074E-11
ABITRAM	0.04676279	2.87274E-11
SNAPC4	0.0663436	2.88236E-11
CDC73	-0.0868499	2.89009E-11
GATAD2A	-0.0803358	2.89572E-11
DIS3	-0.0467781	2.95884E-11
CCDC137	-0.0836169	2.98275E-11
MTPAP	0.03842669	2.99511E-11
SHLD2	0.0081896	2.99928E-11
LENG8-AS1	0.0950686	3.00163E-11
RBM12	-0.0519081	3.0114E-11
ELK1	-0.0304383	3.01299E-11
TADA2A	0.06550961	3.04151E-11
D2HGDH	-0.0450239	3.06107E-11
ERC1	0.04994348	3.07597E-11
APOL6	-0.1200125	3.09063E-11
OPA3	0.01401934	3.11851E-11
WDR33	0.0190096	3.13365E-11
TOMM5	0.21774659	3.15774E-11
ADCY3	0.04445786	3.16266E-11
TIGD5	0.00805085	3.1714E-11
ZNF581	-0.0479214	3.18582E-11
NIPSNAP2	0.10283934	3.19238E-11
MFSD5	-0.0399274	3.20038E-11
HEIH	-0.0439486	3.21105E-11
VPS26B	0.03317834	3.24443E-11
AC012146.1	0.13864162	3.2497E-11
HYMAI	-0.0451208	3.25059E-11
SAE1	0.03430896	3.25096E-11
ZYG11B	0.10574991	3.26266E-11
MLXIP	0.07553528	3.26303E-11
FAM200B	0.05630951	3.29698E-11
HEXB	0.07826523	3.30537E-11
SECISBP2L	-0.1054581	3.33376E-11
MRPL45	0.12872141	3.35149E-11
SCMH1	0.04397556	3.40844E-11
EVI2A	0.16852575	3.42363E-11
PSMD2	0.10115925	3.42913E-11
TFEB	-0.1418244	3.46051E-11
ACSF3	0.14606309	3.49837E-11
SHMT2	0.03121331	3.51196E-11
POU5F2	0.06578612	3.52699E-11
GRSF1	0.07459713	3.52986E-11
AC092279.1	0.03300183	3.53024E-11
ZNF174	0.04431705	3.53712E-11

PARVG	0.13309754	3.55395E-11
RCCD1	0.08638201	3.55422E-11
TP53RK	0.03008597	3.55792E-11
ELOVL5	0.01025008	3.60326E-11
TLE3	-0.0467412	3.61635E-11
ABCA2	0.01166896	3.6199E-11
SUN1	-0.1697952	3.63604E-11
RUVBL2	0.02224472	3.64148E-11
MCM9	0.06578627	3.65149E-11
FAM184B	0.05249141	3.67156E-11
SERINC3	0.08836348	3.68969E-11
SLC7A6	-0.1094501	3.74621E-11
FEM1B	-0.0427796	3.74696E-11
RAB12	0.02599654	3.75627E-11
MKLN1	-0.1042212	3.77958E-11
TRAF7	0.03157964	3.81585E-11
ARHGAP17	-0.0020468	3.81781E-11
DPP3	0.01668591	3.83119E-11
PDS5A	0.11149142	3.90611E-11
TPGS1	-0.0058646	3.96437E-11
MRPL35	0.08930895	3.96683E-11
SLC38A1	-0.2928006	3.97066E-11
PTPN9	-0.0618926	4.01846E-11
WRAP73	0.03234583	4.02358E-11
NIBAN1	0.2748004	4.03068E-11
USP25	0.0741336	4.03534E-11
ASCC2	0.08452082	4.03927E-11
VPS54	0.086076	4.0487E-11
TNKS2	-0.0585298	4.05781E-11
IKBKE	-0.0746879	4.06313E-11
CIAO1	0.08287497	4.06773E-11
ACTR2	0.19081139	4.07324E-11
PLCL2	0.04397289	4.08589E-11
CASTOR2	0.07397532	4.09492E-11
SNAP47	-0.0440473	4.11671E-11
C1orf216	-0.0053072	4.1272E-11
NECTIN2	-0.1518922	4.14576E-11
HERC3	-0.0457596	4.14742E-11
DDX31	0.06320376	4.15489E-11
TMEM64	0.04413452	4.18082E-11
ILKAP	0.01849052	4.20311E-11
FLII	-0.0197646	4.22182E-11
ZP3	0.06019024	4.23451E-11
ACACA	-0.0142086	4.25716E-11
TRIB3	-0.1542503	4.28528E-11
NUB1	0.08833364	4.29014E-11
TMX2	0.06063713	4.29783E-11
STK3	0.08580536	4.36995E-11
CRBN	-0.1783721	4.3899E-11
UPF1	-0.0717736	4.39403E-11

KBTBD6	0.02535017	4.40179E-11
ZNF230	0.05182598	4.42992E-11
FBXO46	0.03424529	4.43071E-11
XRN1	0.04955207	4.49209E-11
MBNL2	0.03331696	4.50121E-11
ABHD16A	-0.083585	4.50795E-11
FASTKD2	0.01300627	4.51473E-11
AC004687.1	0.25967247	4.58319E-11
ZNF816	-0.0030016	4.62821E-11
MPP5	0.0220919	4.63412E-11
SCML2	-0.1256002	4.70332E-11
MED31	-0.1001018	4.72491E-11
VWA8	0.06708286	4.72771E-11
HMBS	0.05441453	4.74164E-11
TBC1D7	0.00982779	4.74645E-11
SOX18	-0.1918914	4.79051E-11
CDK5RAP2	-0.0611125	4.86329E-11
PSMB4	-0.0740174	4.90833E-11
GABPB2	0.10493522	4.94642E-11
ZCCHC9	0.06906206	4.97926E-11
MEMO1	0.06911373	4.97944E-11
EPS15L1	0.09060875	5.01234E-11
AL121933.2	0.03733911	5.04604E-11
ZNF665	0.09776308	5.05385E-11
PDSS2	0.07412286	5.09039E-11
GOLGA8B	0.06889456	5.09137E-11
SRP54	-0.0204624	5.09402E-11
SLC38A2	0.0482157	5.15452E-11
CLN6	0.06261007	5.192E-11
ZNF267	-0.0721027	5.19798E-11
DCAF7	0.13097279	5.19997E-11
GEMIN2	0.02865143	5.24748E-11
TOPORS	0.06316039	5.27458E-11
SMAD4	-0.0695752	5.28582E-11
CDC26	0.03738545	5.29905E-11
AC007038.1	0.07156482	5.35097E-11
JADE2	-0.0641344	5.36557E-11
TSPOAP1	0.07425965	5.40818E-11
VPS35L	0.06888386	5.41863E-11
AMPD2	-0.0966847	5.42566E-11
FAM160B2	-0.0996724	5.46793E-11
CXorf40B	-0.0368251	5.47744E-11
SLC12A6	0.10505125	5.49915E-11
SPIRE1	0.06143789	5.50993E-11
NFU1	0.07204262	5.51163E-11
ITPR1	0.15452702	5.53152E-11
SRCAP	0.026102	5.58849E-11
PSMD1	0.05669327	5.59326E-11
IDH3G	0.00672158	5.60628E-11
MPZL1	0.10392121	5.64249E-11

UNC45A	-0.030172	5.67202E-11
ACTR3B	0.05881248	5.68459E-11
SRI	-0.0989073	5.68664E-11
PMPCB	0.15119011	5.69011E-11
INTS2	0.08714381	5.70987E-11
ZNF383	0.08166887	5.74903E-11
RUNDC1	-3.37E-05	5.95596E-11
RIMKLB	-0.1287357	5.99145E-11
HOXA4	0.04715637	6.06694E-11
WDR37	-0.0285042	6.0833E-11
ZFAND2A	-0.1677919	6.09161E-11
ZBTB24	0.02154058	6.14252E-11
CENPQ	0.06013309	6.14903E-11
IL4R	-0.1685692	6.17091E-11
FNIP1	0.13270563	6.17298E-11
CLMN	-0.0899617	6.18746E-11
AC025171.4	0.06646563	6.19211E-11
DHX9	0.17790122	6.20137E-11
RGS3	-0.1563857	6.20872E-11
SLC1A5	-0.1687942	6.23326E-11
AC024075.1	0.04278444	6.24189E-11
MARCH6	0.04594709	6.30582E-11
DENND1A	0.02728854	6.32474E-11
HSPBP1	-0.0363555	6.37232E-11
ING3	0.0394607	6.37334E-11
ACY1	0.00888795	6.3858E-11
LSM14A	0.02772637	6.38638E-11
VAMP7	-0.0100482	6.38761E-11
ZSCAN32	-0.0023948	6.41453E-11
LTBR	-0.1383232	6.43525E-11
TMEM8A	-0.0037559	6.45787E-11
TADA3	0.06365857	6.46117E-11
LARP7	0.17324302	6.47348E-11
NRBF2	0.01967833	6.49504E-11
KIAA1191	0.02895705	6.53767E-11
LAS1L	0.06735292	6.59547E-11
ZFP3	0.06505498	6.63331E-11
BRCA1	0.00068738	6.65254E-11
AL133330.1	-0.1679473	6.81598E-11
CREM	-0.2476051	6.88696E-11
NBEAL2	-0.2083072	6.89853E-11
BLM	0.09310107	6.94082E-11
PTPN4	0.03298222	6.94305E-11
FNTB	0.08725405	6.9579E-11
SLC23A2	0.02197905	7.00233E-11
ATF7IP	0.13717074	7.13583E-11
ECSIT	0.02188313	7.15528E-11
TSPAN31	-0.0038899	7.17958E-11
HM13	-0.0769584	7.18034E-11
VPS37A	-0.0080559	7.21509E-11

KIF2A	-0.1659946	7.22062E-11
CSE1L	0.05063527	7.23854E-11
ZNF404	0.06128133	7.26844E-11
ARHGAP9	-0.1157882	7.41941E-11
SMIM4	0.11223019	7.42818E-11
CRISPLD1	0.06364801	7.43508E-11
NDUFS7	0.13762262	7.44639E-11
LINC01150	0.03936983	7.44849E-11
AC092171.5	0.05291664	7.52439E-11
PHF12	-0.0436252	7.54163E-11
RDX	-0.100579	7.64435E-11
NCBP1	0.08845615	7.67279E-11
RPS6KB2	-0.0614372	7.72518E-11
RORA-AS1	0.07942625	7.72677E-11
ACP5	0.04997034	7.78446E-11
CYB5RL	0.05966646	7.78573E-11
ZNF586	0.04073332	7.82014E-11
TMEM129	-0.0294126	7.85777E-11
INTS7	0.0361965	7.8862E-11
GPATCH2L	0.17767703	7.89431E-11
STK25	-0.0132027	7.89462E-11
ZNF721	0.10770112	7.90347E-11
ACAT2	0.0511795	7.93461E-11
PRDX4	-0.0815781	7.95656E-11
ARL2BP	-0.1440557	7.95989E-11
ZNF485	0.05700755	8.07668E-11
ECI1	-0.0264082	8.14263E-11
MEN1	0.04510289	8.14286E-11
MEGF6	0.04078608	8.20106E-11
PXMP4	0.07931998	8.20883E-11
CCDC62	0.06999363	8.25428E-11
XPA	0.09845228	8.30751E-11
METTL1	0.08180806	8.37141E-11
TRIM23	0.0094546	8.3753E-11
LSM3	-0.031798	8.54841E-11
NUDT18	-0.072132	8.58932E-11
CYREN	-0.0015741	8.59895E-11
XPO7	0.05876418	8.63067E-11
FABP5	0.27206144	8.68995E-11
ENTPD6	0.00870374	8.70857E-11
ERCC1	-0.0237378	8.75892E-11
ARSK	0.04605301	8.76878E-11
KRR1	-0.0051968	8.77855E-11
NCK2	0.02002894	8.85379E-11
BTBD10	-0.1532175	8.8868E-11
SETD2	-0.1610933	8.90315E-11
GBE1	0.00530806	9.00054E-11
PLEKHF2	-0.0378558	9.00069E-11
LINC00910	0.06828145	9.03339E-11
ZZEF1	0.01857917	9.12227E-11

ATPSCKMT	0.09492154	9.1235E-11
RSBN1L	-0.1860826	9.13022E-11
PTGR2	0.10266693	9.15409E-11
EPGN	0.06224211	9.17137E-11
CCAR2	-0.0144347	9.20695E-11
LIMA1	0.07389266	9.22592E-11
KRT8	0.05823726	9.24897E-11
EDEM1	-0.0425059	9.24974E-11
CD7	-0.0833485	9.25073E-11
TMEM223	-0.0619651	9.26581E-11
WDR20	-0.085075	9.33056E-11
STAM2	0.00351023	9.35001E-11
TRMT2A	0.03582729	9.5137E-11
BX842570.1	0.03848205	9.61451E-11
GNPNAT1	-0.1168768	9.7142E-11
CHERP	-0.0107741	9.77313E-11
ZNF669	0.02561555	9.77748E-11
ATP6V1C1	-0.027533	9.79042E-11
PIGG	0.02376234	9.79562E-11
NAA25	0.02989633	9.89079E-11
SGF29	0.1117314	9.9004E-11
SF3A2	-0.0895803	9.9971E-11
CCDC171	0.06720532	1.00744E-10
AC139099.2	-0.1386346	1.02397E-10
AC018362.1	0.0887283	1.03591E-10
FAM98C	0.01491377	1.03961E-10
DNAJC7	-0.1532845	1.04884E-10
MAGEF1	0.05219052	1.05197E-10
RNF25	-0.108365	1.05306E-10
PISD	-0.0986508	1.06984E-10
TESMIN	0.09811817	1.07364E-10
CCT4	-0.0360651	1.07631E-10
TAF6	-0.0317496	1.07659E-10
LIMK2	-0.0463679	1.07743E-10
MAIP1	-0.0350584	1.07954E-10
IFI6	-0.0325372	1.07985E-10
GBP3	0.06229016	1.09007E-10
ZNF629	0.00964086	1.09163E-10
ZNF90	0.09495553	1.09612E-10
KMO	0.0478248	1.10593E-10
SNAPC1	0.06794725	1.11365E-10
CGRRF1	-0.1311143	1.1274E-10
ATP6V0E1	0.20335114	1.12831E-10
COG4	0.06296689	1.13141E-10
HNRNPA1P4	-0.1042691	1.13179E-10
MRPL46	-0.0118923	1.13624E-10
LRCH3	0.01108522	1.14482E-10
TCEAL3	-0.2157236	1.1504E-10
DNAJB1	0.01602694	1.15362E-10
POLR3E	0.05170018	1.15869E-10

RXRA	-0.0727186	1.16219E-10
ZNF512B	-0.0480524	1.1626E-10
FAR1	-0.1758392	1.16278E-10
FBXO41	0.03651552	1.16704E-10
PSMA3-AS1	0.22053106	1.17172E-10
ALOX12-AS1	0.05765356	1.1725E-10
CHCHD5	-0.0510089	1.17537E-10
RPL19	-0.1918924	1.17941E-10
P2RY10	0.05976079	1.19672E-10
RSBN1	0.04626003	1.20321E-10
ACP6	0.06566618	1.20425E-10
SELENOP	0.30744656	1.21233E-10
COPS4	-0.1696193	1.21566E-10
NIPA2	-0.0435337	1.21956E-10
C3orf33	0.0563232	1.22033E-10
USF1	-0.0724459	1.23054E-10
MYO9B	-0.0064775	1.23702E-10
PRRC2B	0.06634714	1.2452E-10
TMTC2	0.06741524	1.24876E-10
DPP7	-0.1436528	1.25036E-10
UNC93B1	-0.0848712	1.25121E-10
VPS28	-0.0872519	1.26473E-10
MICOS10	0.11944713	1.27482E-10
ACD	-0.0376221	1.29563E-10
XPR1	0.0814629	1.308E-10
ELK3	0.08765874	1.30973E-10
PSMD5	0.00420808	1.31118E-10
TRIM52	0.06318009	1.31356E-10
DTD1	0.0092149	1.31542E-10
CAPN15	-0.020308	1.31591E-10
HOXB4	0.0535815	1.31689E-10
MMADHC	0.0573938	1.31817E-10
RFT1	0.06892302	1.31859E-10
UGP2	0.13741478	1.32054E-10
WT1	0.04169829	1.32527E-10
FAM13B	0.01754189	1.32783E-10
SFXN5	0.08821352	1.3289E-10
U91328.1	0.04218511	1.33219E-10
CCDC189	0.03849674	1.33965E-10
RBMXL1	0.032849	1.33984E-10
WT1-AS	0.07040602	1.34053E-10
DERL3	0.08865254	1.34409E-10
ZNF649	0.05484016	1.35306E-10
LRRC69	0.05586764	1.3553E-10
ANAPC4	0.04677433	1.35863E-10
SMAGP	0.03456422	1.3638E-10
PFKP	-0.0665548	1.36469E-10
STAM	0.05266374	1.38159E-10
MAN1B1	-0.0230624	1.38314E-10
RPS26	0.20453107	1.38751E-10

ZNF518B	0.08839077	1.40226E-10
METTL17	-0.0172972	1.40503E-10
TMEM109	0.04506632	1.40809E-10
MCAT	-0.0889576	1.41717E-10
NUBP1	-0.073407	1.4179E-10
ZNHIT2	-0.0905886	1.42122E-10
ARHGEF3	-0.1247529	1.42494E-10
EXOC6	0.02232143	1.43072E-10
AC080038.1	0.05010932	1.45981E-10
AC002558.3	0.00175992	1.46145E-10
SMIM15	0.02678456	1.46756E-10
PHRF1	0.00204588	1.48744E-10
B3GNT5	-0.066874	1.49044E-10
RCOR3	0.15321587	1.50596E-10
TXNRD1	0.05658295	1.50938E-10
ERCC4	0.08929652	1.51022E-10
PGPEP1	-0.0779423	1.51965E-10
PPAN	-0.0963831	1.51976E-10
FEM1C	-0.0866595	1.52919E-10
ZNF264	-0.1059389	1.5399E-10
THEM4	0.06967697	1.54114E-10
AC102953.2	0.04154382	1.54448E-10
NPAT	0.03967495	1.57945E-10
RLF	-0.0294275	1.58672E-10
HNRNPLL	0.07465695	1.59504E-10
ZMIZ1	-0.0732348	1.59772E-10
COTL1	0.04890755	1.60093E-10
DAP	-0.0555195	1.60276E-10
LRRC27	0.07519802	1.60792E-10
MARC1	0.05350905	1.61057E-10
TMEM44	0.01218515	1.61172E-10
GRINA	0.01520048	1.61457E-10
AGO3	0.11824617	1.61579E-10
C1orf35	-0.0815491	1.61651E-10
CORO1B	0.04675732	1.62188E-10
AL162595.1	0.05804088	1.62383E-10
GON4L	-0.1442353	1.63046E-10
DCAF5	-0.0162357	1.63625E-10
MECP2	-0.0490605	1.63646E-10
SP140L	0.04826112	1.65222E-10
KDM2B	0.06042033	1.65634E-10
STX11	-0.1821135	1.66589E-10
MALSU1	-0.0043246	1.66901E-10
ITPA	0.06932746	1.67182E-10
POLE	0.01888506	1.67374E-10
DIDO1	-0.0439444	1.68093E-10
BOLA2	0.03265924	1.6996E-10
SMARCD1	0.02263744	1.70482E-10
ZFAND6	-0.1258615	1.70765E-10
HIPK3	-0.1350368	1.71582E-10

PTOV1	-0.0657498	1.73152E-10
ANKRD13C	-0.0012008	1.73201E-10
CLEC11A	0.13871773	1.73903E-10
MED16	-0.0177822	1.74185E-10
LCT	-0.1225769	1.74543E-10
KLF16	-0.129339	1.7474E-10
RTCA	-0.0656065	1.75174E-10
PCYT1A	-0.0884756	1.75841E-10
BTAF1	-0.0435491	1.76166E-10
TMEM184B	-0.0697193	1.76585E-10
ZC3H12A	-0.1549924	1.76599E-10
ADAM8	-0.1883603	1.76714E-10
RAB30-DT	0.06610767	1.77389E-10
ANKRD36C	0.1289763	1.77579E-10
PDLIM5	0.04022718	1.80425E-10
GAB2	-0.1335093	1.80611E-10
AC004865.2	-0.0271587	1.82145E-10
FAHD2B	0.04474888	1.82197E-10
CLIC2	0.06914808	1.82306E-10
LNCAROD	0.1018785	1.82369E-10
CTDP1	-0.0958844	1.83868E-10
OLA1	0.14350071	1.84362E-10
PEX2	-0.0236784	1.84521E-10
MED29	-0.0544011	1.84944E-10
FAM98A	0.06156982	1.85246E-10
RTTN	0.0550912	1.8528E-10
SPAG1	-0.1521403	1.85712E-10
ASB13	0.08612682	1.86286E-10
C11orf49	0.0347383	1.86325E-10
SPDYE3	0.06012655	1.87001E-10
AP000766.1	0.05794659	1.87149E-10
SLC1A4	0.05565377	1.88113E-10
FTO	0.07976829	1.88921E-10
AC097376.3	0.06839718	1.89923E-10
PDE12	0.0082159	1.91776E-10
LBHD1	0.03180002	1.93119E-10
SUPT4H1	-0.0583631	1.93674E-10
FUS	0.27035642	1.94838E-10
STAT4	-0.1197421	1.97018E-10
ITGAV	0.05196853	1.97374E-10
RPL21	0.14901322	1.97537E-10
SKIL	-0.0818801	1.98071E-10
DNAJC15	-0.1672823	1.98148E-10
ERMARD	0.0504457	1.98654E-10
KLHDC10	-0.0759283	1.98983E-10
DNTTIP2	-0.0305586	1.99056E-10
LINC00963	0.03147189	1.99114E-10
PPP1R2	-0.092882	1.99374E-10
NKRF	0.00168237	2.0135E-10
ATOX1	-0.1294992	2.01956E-10

PIK3AP1	0.13292494	2.01994E-10
ODR4	0.04880972	2.04036E-10
UBXN8	0.04861914	2.04629E-10
HYOU1	-0.0007666	2.05753E-10
HELLS	-0.1459924	2.0645E-10
TGFBRAP1	-0.0622251	2.06985E-10
AZIN1-AS1	0.04248813	2.07791E-10
KLHL5	-0.0297004	2.08341E-10
AAGAB	-0.0728517	2.08423E-10
DGCR2	0.03567167	2.10459E-10
HSD17B7	0.0405077	2.10793E-10
MNT	-0.1089995	2.1122E-10
SLC25A29	-0.0859763	2.1138E-10
GAA	-0.008875	2.12216E-10
TTPAL	-0.0299215	2.12869E-10
TPGS2	-0.0235672	2.13355E-10
SMG5	-0.0244336	2.13363E-10
COPS8	-0.0823154	2.15666E-10
AC091607.2	0.06747234	2.16391E-10
G3BP1	0.26241138	2.16769E-10
XPO6	0.03098575	2.17194E-10
CEP120	0.09121528	2.18422E-10
FBXL17	0.08143694	2.18921E-10
STAP1	-0.1419743	2.19536E-10
METTL14	-0.0175192	2.23206E-10
RBM18	0.01060778	2.2435E-10
ZFC3H1	0.13962569	2.24535E-10
PRKRIP1	-0.0403758	2.24673E-10
AP1S2	0.12758747	2.25224E-10
PTPN7	-0.1212676	2.25843E-10
CDK2	0.00036636	2.25887E-10
EXOSC7	0.04225437	2.26936E-10
ERMAP	0.05078073	2.27059E-10
LEPROT	0.09130126	2.27509E-10
ABL1	-0.0307271	2.2782E-10
LINC01003	-0.0876028	2.29119E-10
MRPL20	0.1201803	2.29292E-10
IL10RB	0.00600834	2.31143E-10
CPNE2	-0.1129517	2.33923E-10
ANKRD54	-0.0500976	2.35179E-10
SLC10A7	0.04389656	2.35705E-10
ANGPT2	0.05744818	2.35984E-10
NDUFS8	-0.0301016	2.37088E-10
AP1G2	-0.0424688	2.39945E-10
NFE2	0.17381365	2.4181E-10
QTRT2	-0.0073069	2.43316E-10
GSTM2	0.03056174	2.45125E-10
GLCCI1	0.06607793	2.4544E-10
MAP1A	-0.2432424	2.45758E-10
NCOA2	0.04876379	2.45935E-10

COG8	0.05446018	2.46182E-10
SELENON	0.04325885	2.49352E-10
ZNF768	0.01320474	2.50498E-10
CAVIN2	-0.030682	2.51571E-10
IMPA1	0.03611765	2.51633E-10
RGP1	-0.0313731	2.53043E-10
RASSF3	0.00098517	2.54729E-10
MAD1L1	0.04311213	2.55442E-10
RBBP7	0.03788038	2.55589E-10
AC127502.2	-0.0281786	2.56264E-10
RETREG3	-0.0415853	2.58154E-10
BCAT2	0.06496302	2.58582E-10
AC002454.1	0.03220939	2.60055E-10
ZFYVE21	-0.0262466	2.60266E-10
RNF38	-0.116856	2.62887E-10
PFKL	-0.0188197	2.63085E-10
RPSA	-0.2735422	2.65108E-10
CEP97	0.08479318	2.66713E-10
TMEM183A	0.0008101	2.66906E-10
PHPT1	-0.0114157	2.69479E-10
AP5B1	0.07151929	2.71035E-10
BRWD1	0.10134128	2.73847E-10
RBKS	0.06490311	2.74509E-10
MRPL13	-0.0844268	2.74728E-10
HIST1H2AH	0.05623177	2.74879E-10
HCFC2	-0.1230957	2.75291E-10
RIF1	0.07484119	2.75516E-10
ABCC5	0.06818166	2.77281E-10
SZT2	0.0423989	2.7782E-10
CSRP1	0.0542086	2.79027E-10
NUP214	0.09515855	2.80137E-10
NRSN2-AS1	0.05529728	2.82948E-10
TSTD3	0.04525022	2.84191E-10
BCL10	-0.044359	2.85136E-10
CEP85	0.06678685	2.86444E-10
GTF2H1	0.02532826	2.87404E-10
ZCWPW1	0.05946672	2.88164E-10
FXR2	-0.045529	2.89842E-10
TMEM222	-0.0303902	2.90204E-10
PRCP	0.02778801	2.91399E-10
MAEA	-0.0552846	2.91463E-10
BIRC6	0.0882705	2.9184E-10
UBE3A	-0.1001477	2.93774E-10
OGFR	0.01728285	2.93805E-10
TNKS	0.02443843	2.94253E-10
THAP7	0.00312706	2.95034E-10
AL354822.1	0.06010406	2.95666E-10
CGAS	0.1084111	2.96793E-10
RAMP1	0.0896127	2.98207E-10
ZNF589	0.04375963	2.99504E-10

HDGFL2	0.01139506	3.00358E-10
MARVELD1	0.05524332	3.01602E-10
SPIN2B	0.05637425	3.04615E-10
NDUFA1	0.21184422	3.07682E-10
GTF3C5	-0.0698248	3.08953E-10
DHRS11	0.08538517	3.09998E-10
KCTD18	0.04351991	3.10869E-10
UBE2Q1	0.11021316	3.1679E-10
DHRS4L2	0.08087955	3.19373E-10
CCDC77	0.05393708	3.22676E-10
RFC1	-0.137852	3.23817E-10
SCAMP2	-0.1251578	3.25345E-10
CDC37L1	0.00877567	3.25477E-10
NT5C2	0.00352677	3.25908E-10
ARHGAP35	0.03848219	3.26344E-10
SNX5	0.01351389	3.27634E-10
MEAF6	-0.0096976	3.2842E-10
CXCL8	-0.8631588	3.29502E-10
FAM193B	-0.0055032	3.30622E-10
ADGRE1	0.08062728	3.31519E-10
ZNF337-AS1	0.05966482	3.32067E-10
EXOC8	0.0502434	3.33972E-10
SRP19	-0.1276986	3.34171E-10
RALGAPA1	-0.1336445	3.35692E-10
MTFP1	-0.0098308	3.37657E-10
DCAF1	-0.0042498	3.39785E-10
CCDC43	0.00656818	3.45754E-10
TAB3	-0.0174282	3.46043E-10
MINDY2	-0.1451217	3.46999E-10
SCOC	0.02411715	3.52489E-10
CACNA2D4	0.04372655	3.5322E-10
NMB	0.02353335	3.54388E-10
CCT6B	0.05604542	3.56785E-10
ZDHHC14	0.05324769	3.57428E-10
COP1	-0.0102804	3.58096E-10
PHKA2	0.09474486	3.59084E-10
CEP164	0.04966015	3.61072E-10
ZKSCAN8	0.04205407	3.62101E-10
CYB561	-0.1075125	3.64225E-10
CENPC	-0.0254234	3.6431E-10
AC025171.1	0.12600683	3.71289E-10
MAP4K5	0.0205697	3.72072E-10
KLHL20	-0.0206863	3.72454E-10
SMARCA2	0.19305131	3.74093E-10
DICER1	0.15630691	3.83664E-10
NDUFAF1	0.04500164	3.89376E-10
FYCO1	0.03814839	3.9392E-10
NFX1	-0.0617261	3.95802E-10
CDKN1C	0.25287265	3.9599E-10
XRCC1	0.00058611	4.00859E-10

KCTD2	-0.030142	4.02926E-10
POGK	0.07536135	4.06216E-10
KLHL8	0.09105871	4.13153E-10
MTG1	0.04011014	4.14524E-10
POLR1C	0.05144587	4.14552E-10
PGM1	0.07872242	4.18607E-10
NIBAN2	-0.1095427	4.19494E-10
AL109615.4	0.06227389	4.19636E-10
ZNF7	0.02614859	4.20329E-10
RRP8	-0.0220411	4.24961E-10
MRGBP	0.04796605	4.25463E-10
NSMCE2	0.01994866	4.25581E-10
USP16	-0.1701177	4.25887E-10
PITPNA	-0.052863	4.28787E-10
ZNF419	-0.0023455	4.29558E-10
SIAH1	-0.0208942	4.31473E-10
C11orf21	-0.082588	4.32428E-10
DENND4C	0.01304636	4.3301E-10
SEC24B-AS1	0.04484614	4.34123E-10
MEF2C-AS1	0.04043846	4.40323E-10
MAD2L1BP	-0.1142808	4.41527E-10
METTL9	-0.0581832	4.41608E-10
AP5Z1	-0.0534128	4.47762E-10
ARMC8	-0.0220271	4.50872E-10
NUBP2	0.05150883	4.51043E-10
ISCU	0.21775463	4.53863E-10
SPCS3	-0.1494784	4.5732E-10
SUMF2	0.13668767	4.58094E-10
TUBE1	-0.0119352	4.644E-10
SEPTIN1	-0.1090976	4.65939E-10
WDR27	0.07453948	4.68689E-10
ZDHHC18	-0.1193083	4.7425E-10
LYPLA1	0.13135128	4.74376E-10
ZNF45	0.0112878	4.74961E-10
ATN1	-0.0167881	4.79615E-10
ZDHHC23	-0.0554508	4.79715E-10
SCD5	-0.1193153	4.80088E-10
HEXA	-0.0878792	4.85955E-10
PMEPA1	-0.1783589	4.90065E-10
PI4KA	0.118849	4.90618E-10
PMM2	0.09318019	4.94777E-10
PREB	0.03798101	4.9508E-10
TMEM267	0.0856365	4.96821E-10
DUSP12	-0.0513229	5.01688E-10
AGA	0.05352684	5.04531E-10
GFM2	0.05889733	5.06081E-10
ITPRIPL2	0.01790451	5.06733E-10
KPNA3	-0.0314861	5.06918E-10
RSF1	-0.1860459	5.07636E-10
URB1	0.10834011	5.07809E-10

ZNF646	0.01985842	5.07846E-10
SETD4	-0.0275885	5.07982E-10
C12orf73	0.04752317	5.08766E-10
APTX	0.01693424	5.09043E-10
AC025159.1	0.02939966	5.1162E-10
TMEM9B-AS	0.07902731	5.11926E-10
ZKSCAN5	0.02062403	5.16372E-10
HERC1	-0.0097118	5.17275E-10
CHRNB1	0.01577414	5.20835E-10
TBPL1	-0.0458709	5.22795E-10
NUP35	0.04063344	5.23769E-10
L3MBTL3	0.07779613	5.24758E-10
FAM168B	-0.0697354	5.26178E-10
BZW1	-0.1919417	5.2634E-10
DAPK1	-0.1126031	5.27751E-10
NOC4L	-0.019395	5.28763E-10
GSE1	-0.0195382	5.31253E-10
PRDX5	-0.2424344	5.34118E-10
NACC1	-0.1037246	5.3486E-10
BAG5	0.042242	5.35E-10
SFXN4	0.102279	5.37675E-10
CCDC34	0.12076406	5.40509E-10
RNGTT	0.00796939	5.41466E-10
PEX13	-0.1150539	5.4181E-10
ALG9	0.02656482	5.45402E-10
POR	0.03354057	5.45544E-10
JAK3	-0.1292981	5.50499E-10
CHKB-DT	0.05818065	5.50778E-10
YEATS2	0.03250949	5.51495E-10
PLCE1	0.05659442	5.5163E-10
TAF3	0.05806272	5.52867E-10
B3GNT2	0.08510693	5.52965E-10
TBXA2R	-0.1306729	5.54073E-10
ZDHHC5	-0.0097093	5.57143E-10
HPS4	0.02071297	5.59964E-10
PPP3CB	0.06080488	5.60303E-10
MRPS18C	0.14988658	5.60419E-10
KPNA1	-0.0632983	5.61012E-10
RBBP8	0.03641792	5.61093E-10
BIVM	0.07259716	5.63494E-10
TTLL12	0.04551203	5.64589E-10
MED13	-0.0383143	5.65396E-10
ANKRD42	-0.0606225	5.66081E-10
HDAC5	-0.1118823	5.66333E-10
ZNF507	0.0993411	5.68352E-10
SECISBP2	0.00577333	5.69398E-10
BCAT1	-0.0199034	5.75322E-10
POLRMT	0.07065696	5.8093E-10
PDCD7	0.0786502	5.82755E-10
PRRC2C	-0.2675474	5.84131E-10

CD59	-0.0922978	5.85122E-10
AKR7A2	-0.0485759	5.85129E-10
EXOSC9	0.02269941	5.901E-10
MED19	-0.1317572	5.94289E-10
SPSB2	-0.0677835	5.94391E-10
CD33	-0.1133728	5.94692E-10
ATP13A2	-0.0560871	5.98046E-10
TOM1L2	-0.0547458	6.07739E-10
TKFC	-0.0075825	6.17444E-10
MCMBP	0.07081385	6.2563E-10
TYSND1	0.04732284	6.30501E-10
LRP8	-0.0682308	6.40644E-10
ZNF791	0.03195593	6.45062E-10
H1F0	-0.0832729	6.46421E-10
MYO18A	0.11180823	6.48389E-10
TCTN1	-0.0534102	6.52699E-10
KIF23	0.06487962	6.55783E-10
SLC16A1	0.05988951	6.56113E-10
NDUFA7	0.01215629	6.59942E-10
TRIP4	0.06948766	6.64849E-10
CNTROB	0.023474	6.70181E-10
GBA	0.02610369	6.72155E-10
AL512625.3	-0.034323	6.72597E-10
SMIM3	-0.2198149	6.74129E-10
NBPF26	0.03988364	6.74347E-10
IL2RG	-0.3322644	6.7448E-10
ZNF331	-0.0007078	6.74595E-10
SHC1	-0.0426611	6.77448E-10
PEX6	0.05056607	6.78551E-10
POGZ	-0.0641421	6.80216E-10
AC245060.6	0.0503279	6.80715E-10
MAML3	0.09545356	6.85074E-10
UROS	0.20217822	6.90477E-10
RFX5	0.03331468	6.91443E-10
TPX2	0.02703252	6.96E-10
TRIM2	0.07680585	7.03435E-10
CCDC102A	-0.0203808	7.04417E-10
ATXN1L	-0.0872935	7.06514E-10
TMEM141	-0.017367	7.12102E-10
EIF1AD	-0.0804058	7.22047E-10
NDRG1	0.11714243	7.28699E-10
MCRS1	0.01753718	7.41837E-10
TASOR2	0.11745983	7.44669E-10
CTDSP2	-0.0930972	7.45693E-10
IFI27L2	0.10300639	7.5023E-10
STRADA	0.00032464	7.52011E-10
CCNL2	-0.1119296	7.54268E-10
GNRH1	-0.1107409	7.54599E-10
SNHG16	0.21912847	7.60431E-10
ZNF266	0.00128657	7.6171E-10

IFFO1	0.03954323	7.61978E-10
BYSL	-0.0085042	7.69501E-10
TSC2	0.01832669	7.70268E-10
CUL4A	0.04209293	7.70914E-10
HOMEZ	0.04013707	7.73696E-10
CD3EAP	0.08789477	7.75474E-10
RABGGTA	0.04358158	7.79904E-10
ZNF724	0.06882558	7.92496E-10
NANP	0.01327254	7.97675E-10
NAA35	-0.0110876	7.98907E-10
C12orf57	0.13464608	8.04032E-10
DET1	0.05717628	8.04784E-10
DPP9	-0.0276348	8.05459E-10
GPR89B	0.05004959	8.08662E-10
IMPAD1	-0.0148643	8.09534E-10
TTC27	0.06741642	8.10598E-10
HNRNPF	-0.1991311	8.12432E-10
HS6ST1	0.09853107	8.13382E-10
GPR180	0.06320742	8.13665E-10
CHST10	0.03852441	8.17966E-10
CTPS2	0.00890424	8.18221E-10
USF3	0.03284283	8.18791E-10
GTF3C4	-0.018957	8.21556E-10
PRMT9	0.02775992	8.29938E-10
GPATCH3	0.00312614	8.4514E-10
AC092338.3	0.04162353	8.52082E-10
SLC1A3	0.05827533	8.5316E-10
MEF2D	-0.092228	8.56376E-10
NUDT15	0.04418954	8.68136E-10
LCMT1	0.00508502	8.68493E-10
USP42	-0.0474321	8.79025E-10
RGL2	-0.1391293	8.81879E-10
TTF1	-0.05193	8.82498E-10
SPTY2D1	-0.1402332	8.85735E-10
NELFCD	-0.0163854	8.86003E-10
AC058791.1	-0.1438958	8.86519E-10
COQ8B	-0.0225353	8.90967E-10
CCT2	0.03115377	8.92883E-10
NAT9	-0.0641202	8.94589E-10
POLD4	-0.0076122	8.9634E-10
RAVER1	-0.0091336	8.9858E-10
HPS5	-0.0015653	8.99829E-10
DCP1B	0.0037233	9.06108E-10
RGPD5	-0.0270383	9.11511E-10
CAMKMT	0.05632507	9.14705E-10
DCTN1	0.03157393	9.15523E-10
SREK1IP1	-0.0019981	9.17948E-10
PTGDS	0.43985925	9.27181E-10
FCGRT	0.10710294	9.2794E-10
FGD4	0.04162462	9.37676E-10

SLC31A2	-0.0986315	9.46329E-10
MORN2	0.00249563	9.58413E-10
ABHD18	0.0497516	9.60513E-10
ANKRD33B	0.07381435	9.6113E-10
NME3	0.05951103	9.62275E-10
MMP17	0.04395292	9.66695E-10
ARHGAP22	0.16315618	9.69286E-10
CARD8	0.14321985	9.7116E-10
TBKBP1	-0.0697283	9.72929E-10
CS	0.08532859	9.73944E-10
PHF10	0.04060663	9.74028E-10
PRDX6	-0.2121058	9.8238E-10
LINC02482	0.07859956	9.8535E-10
CLDND2	-0.1562057	9.90477E-10
VKORC1	-0.0199855	9.94095E-10
DYNC1LI2	-0.1093396	9.95593E-10
FBXO6	0.03687151	1.00066E-09
AL391684.1	0.04336894	1.00678E-09
VPS13A	0.10322127	1.01348E-09
SLC39A9	0.08946882	1.014E-09
PGBD1	0.03252068	1.02185E-09
AUTS2	-0.0320383	1.02825E-09
MARK3	-0.067709	1.04134E-09
TUBGCP2	-0.0659546	1.04641E-09
TXNDC11	-0.0550181	1.04928E-09
HSBP1L1	-0.0110527	1.06169E-09
FAM149B1	0.0169288	1.06627E-09
IGHMBP2	-0.0846903	1.07797E-09
MIR181A1H(	0.02373159	1.08017E-09
RBMS1	-0.1748591	1.08489E-09
CCNQ	-0.0892753	1.08675E-09
HSPA13	-0.0864262	1.0909E-09
HIST1H4J	0.07362882	1.09214E-09
UTP23	0.05398344	1.10011E-09
ASMTL	-0.1374843	1.10416E-09
MRPL38	0.03174347	1.10606E-09
AC020915.2	0.06180247	1.10607E-09
POLH	0.0815912	1.10627E-09
LMBR1L	-0.0725803	1.11667E-09
C12orf4	0.06365929	1.12028E-09
AP5M1	0.11313787	1.12598E-09
TRAF3IP1	0.1255342	1.13797E-09
CTSS	0.20302514	1.14467E-09
NF1	0.06891558	1.16194E-09
FANCM	0.07457493	1.16794E-09
MILR1	-0.0553486	1.16827E-09
JTB	0.14134158	1.1791E-09
OXSM	-0.0065259	1.18236E-09
PSIP1	-0.0076947	1.18557E-09
TM2D2	0.02086536	1.18902E-09

ZCCHC2	0.00160684	1.18906E-09
CPT2	0.02920377	1.19268E-09
PHYH	0.06289445	1.1953E-09
REXO5	0.06736576	1.19787E-09
AKIP1	-0.0056146	1.21387E-09
MANEA-DT	0.03418423	1.21461E-09
DIAPH1	-0.0512255	1.2175E-09
WDR6	-0.0065026	1.22512E-09
PPP1R3D	0.07032907	1.22887E-09
UHRF1BP1L	-0.0085893	1.23361E-09
SNX6	-0.0226314	1.24109E-09
IGF2BP2	-0.0994334	1.24522E-09
ZNF805	-0.0188163	1.25643E-09
WIPF2	-0.1118634	1.2589E-09
ABCF1	-0.0394096	1.2623E-09
MAN2B1	0.02579588	1.26741E-09
CU638689.4	0.0598661	1.27008E-09
AC015871.4	0.03879471	1.27533E-09
MVB12B	0.03123622	1.2777E-09
ATP6V1E1	0.03053189	1.28349E-09
LRRC34	0.06304064	1.2894E-09
NUDT16	0.05252704	1.29364E-09
TAF5L	-0.0492293	1.30231E-09
GALNS	0.00280541	1.30497E-09
LMAN2L	0.04856692	1.31672E-09
AC026979.2	-0.1826358	1.31825E-09
CREB3L4	0.09265113	1.31834E-09
TGOLN2	0.15548284	1.3191E-09
DNAJB9	-0.0282446	1.32775E-09
ZFP82	0.04761342	1.32988E-09
MUL1	-0.0611856	1.33345E-09
KRBOX4	0.01777024	1.33617E-09
TRGC1	-0.0174429	1.35376E-09
DTX2	-0.0049398	1.35714E-09
TUSC2	-0.0786283	1.36456E-09
PSMG3-AS1	0.03512154	1.3656E-09
PUS1	0.0663181	1.3732E-09
LSM7	-0.251323	1.37802E-09
GPR107	-0.070563	1.38398E-09
LRRC8C-DT	-0.026352	1.38429E-09
P2RX5	0.12987123	1.38945E-09
F8A1	0.06696622	1.40175E-09
CYC1	-0.0069155	1.40581E-09
MLC1	-0.0627082	1.41002E-09
SNX16	0.04303514	1.41048E-09
TLR6	0.0516216	1.41079E-09
MTRF1L	-0.136277	1.41087E-09
ROMO1	0.08807559	1.41746E-09
SPICE1	0.07870325	1.42391E-09
ARMC6	0.06119948	1.4245E-09

ZNF576	0.00732826	1.43819E-09
NVL	0.01982347	1.44803E-09
CCNI	-0.2573469	1.4534E-09
NEMP2	0.03948827	1.45512E-09
ZDHHC13	0.0704699	1.4589E-09
METTL6	0.03173981	1.45971E-09
UNC13D	-0.0301536	1.46532E-09
PXMP2	-0.0105348	1.46565E-09
SOCS4	0.11829211	1.47437E-09
AL158152.1	0.03837146	1.48073E-09
CASP10	-0.1477866	1.48157E-09
COMMD5	0.01494745	1.48695E-09
MTF1	-0.0289952	1.49174E-09
ZSWIM9	0.06202509	1.49636E-09
GSTCD	0.06718013	1.4979E-09
RDH11	0.035033	1.51415E-09
PKN1	-0.0046412	1.51699E-09
MYLPF	0.13395898	1.52213E-09
FBXL19	0.02530452	1.52551E-09
LYZ	0.42146657	1.53503E-09
NADK	-0.0235927	1.5551E-09
ZNF407	-0.0101193	1.55615E-09
AC027097.2	0.05765096	1.56472E-09
LMBR1	0.01062886	1.57728E-09
TESK1	-0.1047618	1.58192E-09
TMEM39A	-0.032728	1.58897E-09
ADAP1	0.03618518	1.58927E-09
ACP1	0.02264097	1.60373E-09
ILRUN	-0.0232727	1.61212E-09
CLIP1	0.04975404	1.6288E-09
TERF2IP	-0.1735479	1.64801E-09
CYB5R1	0.01507947	1.67141E-09
UBR4	0.02843932	1.67464E-09
DDX27	0.13075561	1.6771E-09
NPRL3	0.07868475	1.69072E-09
PABPN1	-0.0855071	1.70127E-09
TACO1	-0.0854789	1.7117E-09
GATA2	0.46185512	1.71363E-09
CDK2AP1	0.02765946	1.71976E-09
RARS	0.07320605	1.74795E-09
INTS12	0.02058352	1.74929E-09
ZNF318	0.05879188	1.77287E-09
ANKRD36B	0.04243677	1.77728E-09
OSBPL2	0.02651574	1.77735E-09
GYPC	-0.2069752	1.77881E-09
MAPK8	-0.0936119	1.81761E-09
FLNA	0.04073369	1.82588E-09
GALM	0.08398307	1.82922E-09
ZNF8	0.07664964	1.83898E-09
MON2	0.04803008	1.84625E-09

LINC01857	0.07732926	1.84996E-09
DYNC1H1	-0.1270679	1.87065E-09
CEP250	0.0726881	1.87525E-09
ICA1	0.02155981	1.88124E-09
AC008105.3	-0.0510084	1.88768E-09
TRMU	-0.0099396	1.88792E-09
ORC5	0.0268323	1.88848E-09
AMACR	0.03219787	1.89818E-09
FUBP1	0.15704942	1.9302E-09
FAM104B	0.04983433	1.95667E-09
SNX4	-0.0725604	1.97629E-09
ZNF607	0.00907667	1.98472E-09
HDAC4	0.02397054	1.99409E-09
MECR	0.05708146	2.00426E-09
AL161457.2	0.05973695	2.01623E-09
ZNF16	0.04141775	2.02275E-09
VPS4B	0.06049853	2.02379E-09
DCLRE1C	0.00850487	2.04325E-09
RHPN1	-0.0549286	2.05543E-09
HIST1H3D	-0.1222862	2.05848E-09
TMEM185B	0.03849374	2.06021E-09
GPR135	0.06579383	2.06366E-09
NT5DC3	-0.1508852	2.07199E-09
AC073389.1	0.02885998	2.09967E-09
WDR60	0.13084768	2.10868E-09
SUPT3H	0.02060163	2.11396E-09
CFAP410	0.03207143	2.14721E-09
SLC17A9	-0.0318706	2.15272E-09
MMP19	-0.1793188	2.18485E-09
ORAI3	0.03215636	2.19866E-09
AEBP1	0.09210132	2.20606E-09
FNDC3B	0.12262283	2.21371E-09
PUSL1	-0.0084881	2.21581E-09
ACSL1	-0.017086	2.2171E-09
RXRB	0.01131138	2.22667E-09
IL17RA	0.0656212	2.23058E-09
FAM215B	0.0160811	2.23502E-09
NOL4L	-0.0803801	2.23713E-09
STX18-AS1	0.06908349	2.25186E-09
TMEM39B	0.03128436	2.25629E-09
DNAJC3-DT	0.08884069	2.26639E-09
MDH2	-0.0074329	2.27211E-09
PROC	-0.1269695	2.28627E-09
AC024060.2	0.06764102	2.28889E-09
LPCAT3	-0.1264053	2.28906E-09
PGD	0.12375492	2.28945E-09
DCUN1D2	0.02361476	2.29258E-09
ZNF571	0.00717592	2.30324E-09
COG1	-0.0413692	2.30711E-09
CCDC59	0.08730533	2.31021E-09

STYX	0.04719196	2.33456E-09
C20orf194	-0.0869285	2.34496E-09
NDUFB5	0.19426571	2.34754E-09
C8orf76	-0.1011918	2.35536E-09
SERPINA1	0.00926446	2.37282E-09
KANK2	-0.1416156	2.37725E-09
NUS1	-0.0073743	2.38774E-09
THAP1	-0.0151932	2.38947E-09
STK17A	0.00236219	2.40555E-09
NRDE2	0.10485363	2.42141E-09
ZNF430	-0.0583206	2.43143E-09
AC103769.1	0.03614611	2.43278E-09
ZHX1	0.06655301	2.4467E-09
RPL5	0.14507684	2.45298E-09
JMY	0.04801351	2.45884E-09
GGH	-0.0841198	2.46479E-09
DDHD2	0.02461641	2.47158E-09
OGFOD2	0.05065605	2.47487E-09
GPATCH1	0.08381661	2.49521E-09
LPIN2	-0.0858461	2.51169E-09
MARCH2	-0.0267804	2.52074E-09
EMC1	0.08842418	2.54574E-09
ATP1A1-AS1	0.07356619	2.56736E-09
SCFD2	0.05721422	2.56979E-09
MAGEH1	0.05256137	2.58479E-09
TMEM51	-0.0763939	2.59228E-09
HTT	0.01090295	2.6015E-09
AC002464.1	0.04390102	2.60626E-09
PALD1	0.0905863	2.62033E-09
NRF1	-0.0047203	2.66611E-09
C6orf47	-0.0885601	2.67427E-09
NDUFS3	0.04926956	2.67594E-09
PDE7A	0.04891697	2.68109E-09
AATK	-0.1336601	2.69269E-09
AC008915.2	0.03596161	2.69721E-09
AC159540.2	0.07982343	2.69797E-09
GIPC1	-0.0040562	2.71066E-09
SETD6	0.05306447	2.7222E-09
DISC1	0.06197085	2.74082E-09
DHX29	-0.0191065	2.75846E-09
TRAF3IP3	0.02754032	2.75925E-09
SPTBN1	0.00067561	2.77105E-09
STK16	-0.0137487	2.77503E-09
SSH1	-0.0530014	2.7772E-09
REV1	-0.0262373	2.7835E-09
ZKSCAN4	0.04759285	2.8162E-09
FBXW4	-0.0201957	2.82001E-09
TXN2	-0.1023762	2.84418E-09
LINC02591	0.03105015	2.84646E-09
XRN2	0.16096848	2.86829E-09

PLAGL2	-0.0292745	2.88621E-09
PGAP2	0.03470984	2.88628E-09
APOL2	0.06401618	2.89067E-09
CSNK2A1	-0.0548048	2.89446E-09
RNF14	-0.0119343	2.89891E-09
DOT1L	-0.0874718	2.90781E-09
ZNF100	0.09098723	2.9177E-09
DHX34	0.01427906	2.92663E-09
ZPR1	0.01323121	2.93371E-09
SLC25A17	0.04608768	2.93413E-09
ENTPD7	-0.0239977	2.94864E-09
MTERF4	0.16022993	2.99031E-09
DSE	-0.0240746	3.02139E-09
PCYOX1L	0.05675372	3.03223E-09
KLHL6	-0.0034649	3.04665E-09
PYCR1	0.05898014	3.05094E-09
TRAF3IP2	-0.0287853	3.0753E-09
IQGAP2	0.16161276	3.07611E-09
TTC33	0.02786985	3.08329E-09
LIMD2	-0.2450983	3.08494E-09
ZDHHC17	0.03423753	3.098E-09
VARS	0.01010372	3.10212E-09
C11orf74	0.11456618	3.10646E-09
C7orf26	0.03626177	3.11087E-09
PIK3CG	-0.0326913	3.11481E-09
PIK3R2	0.06153145	3.12088E-09
CREBRF	-0.1998729	3.13519E-09
RMC1	-0.0416644	3.15305E-09
MAP2K6	0.05573166	3.16937E-09
TMEM234	-0.1198058	3.17482E-09
OPRL1	0.02729866	3.19547E-09
SATB1	0.12275212	3.19943E-09
TTL	-0.0158239	3.25511E-09
SSBP2	0.04703346	3.27064E-09
URGCP	0.03876734	3.29254E-09
PBRM1	0.10266664	3.30295E-09
ZNF286A	0.092406	3.33932E-09
SVBP	-0.0657926	3.34582E-09
ABHD2	0.18646967	3.36003E-09
MORC3	-0.0267861	3.36212E-09
CC2D1A	-0.0022863	3.37101E-09
PDE6G	-0.0683541	3.39008E-09
HACD4	-0.01296	3.40079E-09
TBL2	-0.0897648	3.4047E-09
UBTD2	-0.0397656	3.4176E-09
RPS25	0.15695868	3.46378E-09
ZKSCAN1	0.11369752	3.48034E-09
POP4	-0.016571	3.49282E-09
PRKCI	0.00525937	3.50405E-09
AGAP9	0.03223829	3.55357E-09

B4GALT5	-0.049	3.55467E-09
PANK3	-0.006008	3.55903E-09
AC012358.3	0.05724217	3.59703E-09
PTRHD1	0.08291417	3.62801E-09
LRRC37A2	0.04880603	3.6601E-09
TMEM182	0.06350857	3.67332E-09
MED7	0.02656106	3.69683E-09
PFAS	0.05742409	3.70954E-09
RPL26	0.13452538	3.73613E-09
CHCHD1	-0.0420621	3.74891E-09
CNOT6L	0.03027436	3.74924E-09
ANKRD50	0.08122555	3.76377E-09
ZSCAN12	0.03838845	3.76596E-09
BCL2L11	0.00122335	3.7683E-09
BBIP1	-0.0072184	3.77832E-09
AZI2	0.08496104	3.78232E-09
ITGA6-AS1	0.05664126	3.81747E-09
LPP	0.04106214	3.82252E-09
CCT7	0.16838356	3.85251E-09
CARM1	0.04857367	3.86453E-09
ITFG2-AS1	0.06361145	3.87044E-09
MAST4	-0.0459263	3.93994E-09
CLUH	0.02537334	3.95596E-09
C18orf21	-0.041673	3.9598E-09
AL049840.4	-0.0551215	3.9712E-09
ABCA7	0.03565122	3.97162E-09
RREB1	-0.0253515	3.98009E-09
TAF5	0.0294184	4.0085E-09
ANKS3	0.04635944	4.01223E-09
GALNT10	0.0368517	4.02491E-09
IFI35	-0.0666246	4.07863E-09
INPP5A	0.04687183	4.08983E-09
MPND	0.04922063	4.10048E-09
MMS19	0.00864729	4.10967E-09
AC108134.3	0.035493	4.13919E-09
ARMH3	-0.0399339	4.15914E-09
UTP11	-0.0360294	4.23232E-09
SBNO1	0.10104059	4.25553E-09
ANO10	0.00013577	4.28827E-09
ALDH18A1	0.06663336	4.29678E-09
CDK5RAP3	-0.0003852	4.34111E-09
ATP6AP1L	0.04632001	4.34976E-09
DEFB1	0.08728394	4.35128E-09
MOB1B	-0.1287161	4.35985E-09
SDCBP2-AS	0.05677649	4.36972E-09
ZNF280C	0.02506119	4.37484E-09
STX16	0.00546424	4.41531E-09
PICK1	-0.0174949	4.42013E-09
HDHD5	0.06400263	4.42687E-09
ICMT	0.03778561	4.45884E-09

ZSCAN9	-0.00383	4.48811E-09
RP9	0.01172345	4.51597E-09
SAV1	0.04194371	4.52436E-09
ZNF408	-0.03931	4.59517E-09
CCNB1IP1	0.10351497	4.62704E-09
CAPZA1	0.02998118	4.62732E-09
LYRM1	-0.0264388	4.63164E-09
ULK4	-0.147712	4.63925E-09
SWSAP1	0.06924603	4.64095E-09
PPARGC1B	0.08424898	4.64603E-09
VTA1	-0.031085	4.65514E-09
CIB1	-0.0439506	4.74478E-09
QPCTL	0.02870111	4.76186E-09
CCDC88B	-0.13296	4.82883E-09
TTC4	0.04761943	4.83562E-09
RNASEH1-A	0.0515903	4.8687E-09
GTF2IRD2B	0.07535223	4.86929E-09
PARPBP	0.01588557	4.8899E-09
SLC2A13	0.05571845	4.9587E-09
EIF4ENIF1	-0.0180425	4.96758E-09
ZNF10	-0.001368	4.99591E-09
ITGB2-AS1	-0.1108631	5.00163E-09
EP300	0.10280162	5.04664E-09
FAM199X	-0.008528	5.05511E-09
ZNF709	-0.0235377	5.05588E-09
WIPF1	0.09252991	5.05685E-09
SCLY	0.06169583	5.08693E-09
G6PC3	-0.1063423	5.11092E-09
PFDN2	-0.0887436	5.11498E-09
AKAP17A	-0.0638928	5.14253E-09
PIK3R4	-0.0623935	5.14881E-09
AC067930.8	0.04237605	5.20957E-09
AL133243.2	-0.0799157	5.2572E-09
ZSCAN21	0.0270575	5.29149E-09
HACE1	0.04628764	5.31201E-09
PGAM1	0.03951294	5.31313E-09
CCDC169	0.08756687	5.35799E-09
RBM25	-0.275985	5.36946E-09
PTBP3	-0.0456768	5.39498E-09
RAB5IF	0.14165712	5.44684E-09
SEC16A	0.0305817	5.46321E-09
XKR8	-0.0822471	5.47837E-09
PSMA2	-0.0877486	5.48639E-09
PDK1	0.02732759	5.51596E-09
CEP83	0.11520155	5.52113E-09
IFT80	-0.0017181	5.53949E-09
ZNF688	-0.0750824	5.55007E-09
PCBP2-OT1	0.04270907	5.57267E-09
C2orf76	0.03777037	5.58298E-09
ASH1L	0.05008754	5.59108E-09

RYK	0.07708728	5.60607E-09
CEP350	-0.0646016	5.63413E-09
MX2	0.05562712	5.65119E-09
Z93930.2	0.05184165	5.65351E-09
DDX19B	-0.0404284	5.70267E-09
STYXL1	0.04968189	5.73926E-09
IFI27L1	-0.0494016	5.76283E-09
FAM229B	-0.1073276	5.77438E-09
PHF11	0.13546708	5.7773E-09
GMEB1	-0.0845674	5.8174E-09
SBNO2	-0.0678978	5.89212E-09
TRMT44	0.05467356	5.92929E-09
AC008764.8	0.05838391	5.94013E-09
PPRC1	-0.0407905	5.94499E-09
TPK1	-0.0348986	6.03893E-09
PLD6	0.06335031	6.04094E-09
SLC25A12	-0.0276391	6.05307E-09
PPHLN1	-0.1270373	6.06633E-09
RBM45	0.06543422	6.1183E-09
PHF2	0.07657046	6.16822E-09
MINDY3	-0.0594077	6.25223E-09
ZNF615	0.03570935	6.25432E-09
DVL3	0.0786326	6.25532E-09
GBP2	-0.0734591	6.26377E-09
HOXB-AS3	-0.0255155	6.29941E-09
ZNF580	0.00931284	6.37878E-09
ABHD12	0.01565502	6.38475E-09
ERAL1	0.02614154	6.42669E-09
IGLL1	0.24432888	6.46217E-09
ABCC1	0.06794285	6.57244E-09
RCL1	0.01337525	6.63365E-09
DGCR8	0.01547505	6.64404E-09
SLC22A18	-0.0763546	6.66853E-09
TTC19	0.14240476	6.67063E-09
AXIN1	0.00806783	6.70355E-09
ZC3HC1	-0.0335046	6.73446E-09
U2AF1L4	-0.0268594	6.75173E-09
SLC5A6	0.04461305	6.75988E-09
TLR9	-0.0762323	6.76059E-09
MIS12	0.03766785	6.78366E-09
CLEC16A	0.06136945	6.8E-09
TBC1D22A	0.05146458	6.89897E-09
TRAPPC6B	0.0053904	6.90201E-09
ZNF250	0.00872896	6.94253E-09
ARHGAP23	-0.1368461	6.95304E-09
EML2	0.02882927	6.96755E-09
EIF3C	0.06099674	7.01364E-09
GOSR2	-0.0066398	7.01747E-09
FBXO30	-0.1192435	7.04525E-09
PARK7	0.14126032	7.04642E-09

CTSH	0.14542314	7.091E-09
SEPHS1	0.03331596	7.12537E-09
RNF170	0.01436979	7.1612E-09
PYCARD	-0.189234	7.25268E-09
MTERF3	-0.0122584	7.26573E-09
PSME3	0.00988321	7.30187E-09
RPS6KB1	-0.053406	7.34136E-09
DDIT3	-0.2462179	7.36195E-09
WHAMM	-0.0259818	7.40019E-09
APOBEC3D	0.04893643	7.41359E-09
CKMT2-AS1	0.07453854	7.42092E-09
CMTM7	-0.0352384	7.44953E-09
MIATNB	0.05879871	7.48033E-09
ATP5MC1	0.07804349	7.48897E-09
NLRC3	-0.1405595	7.54234E-09
NOTCH2NL(	0.04742587	7.56279E-09
HIST1H1B	-0.1581569	7.63291E-09
MIER2	-0.0579944	7.63985E-09
SOCS1	-0.2128901	7.64365E-09
SLC16A5	-0.0220703	7.67615E-09
STARD9	0.0994421	7.72168E-09
CHFR	-0.0009221	7.73686E-09
MFSD8	-0.0145505	7.73711E-09
MFSD11	-0.0096486	7.76764E-09
TRAK1	-0.0676118	7.82375E-09
LINC00641	0.0750522	7.85047E-09
DDI2	-0.0831925	7.85717E-09
AC136944.2	0.05074645	7.87158E-09
PLEKHO1	-0.2503397	7.89425E-09
SEC31B	0.04459022	7.89953E-09
CANX	0.22092413	7.9135E-09
AL138724.1	-0.0482385	7.91427E-09
FOXRED2	0.05036128	7.92464E-09
CIAPIN1	-0.0077033	7.93928E-09
OSER1-DT	0.03711914	7.96312E-09
IFRD1	-0.0736266	7.97692E-09
UBE4A	0.04210946	8.02296E-09
DLAT	0.03181493	8.02528E-09
Z97192.1	0.0528377	8.03413E-09
CCDC130	-0.0571755	8.0599E-09
TAF13	-0.0316345	8.10124E-09
SUGP1	0.04967558	8.1823E-09
MICOS13	0.17521623	8.20938E-09
ORAI2	0.25487773	8.2885E-09
PUS10	0.03335432	8.35787E-09
BAX	0.14176949	8.39896E-09
FEN1	-0.0522028	8.43455E-09
CCAR1	-0.1088242	8.46559E-09
ORC6	-0.0460255	8.46772E-09
C10orf95	0.07450029	8.47865E-09

TNFAIP8L2	-0.019801	8.50167E-09
SLFN12	0.06739298	8.54314E-09
VCPIP1	0.0127223	8.55926E-09
CALCOCO2	-0.0640653	8.56953E-09
EFL1	0.06943124	8.5707E-09
PIK3R1	-0.13636	8.72888E-09
RELCH	-0.0120073	8.74454E-09
RRAGB	0.03817542	8.76664E-09
LINC00467	0.08535538	8.78869E-09
GPM6B	0.0367376	8.8238E-09
GTF2A1	0.07925119	8.84144E-09
CCT3	0.16894637	8.90619E-09
IL15RA	-0.0136201	8.91252E-09
LINS1	0.04943798	8.9454E-09
CHUK	0.0123323	8.9496E-09
LINC02615	0.02436003	9.05897E-09
PTTG1	-0.0406935	9.12936E-09
WDR59	0.02412013	9.34511E-09
MRPS36	0.10816051	9.39101E-09
MAP3K13	-0.044204	9.49416E-09
MICAL3	0.03886821	9.50458E-09
SAAL1	0.02808499	9.54859E-09
CDC23	0.01524199	9.56848E-09
PLXNA3	-0.1854005	9.58162E-09
HEXD-IT1	0.03458249	9.59623E-09
MRM2	-0.0213797	9.64691E-09
MED9	-0.0548521	9.69802E-09
MFN2	-0.0073792	9.74585E-09
ZNF516	-0.1237394	9.76787E-09
MRI1	0.08484153	9.7999E-09
FARS2	-0.0264561	9.82172E-09
SLC29A3	0.03295573	9.88386E-09
RBM15	-0.0048276	9.88494E-09
FAM160A2	-0.0370435	1.00084E-08
ZNF671	-0.0238678	1.00445E-08
HLA-F-AS1	0.03975267	1.00629E-08
ZNF418	0.01765661	1.02132E-08
MED23	-0.0300663	1.02367E-08
ZNF568	0.07249971	1.04249E-08
RCAN1	0.0450763	1.05361E-08
AL589863.1	0.05358218	1.05944E-08
RNF103	-0.1027882	1.06171E-08
MATK	0.05833341	1.06237E-08
ZNF558	0.03505548	1.08126E-08
UQCR11	0.19323267	1.08253E-08
SLX4IP	0.0409128	1.08459E-08
COPG2	0.08917126	1.0847E-08
TBC1D23	-0.0588853	1.09117E-08
SMURF2	-0.0549192	1.09147E-08
SYNGR1	-0.1573815	1.1029E-08

DMTN	0.09525804	1.10976E-08
CHSY1	0.09762966	1.11083E-08
GOT1	-0.0271326	1.11492E-08
STK19	-0.1095297	1.11703E-08
ABHD14B	-0.0247701	1.1235E-08
GFPT1	-0.0540739	1.1276E-08
UBIAD1	0.04567412	1.12935E-08
ZC3H10	0.04263923	1.13831E-08
LACTB2	0.09724008	1.13929E-08
JRK	0.0951225	1.13932E-08
PTPN12	-0.0278029	1.13941E-08
ZSCAN26	0.06244518	1.1483E-08
WDR54	-0.149816	1.15041E-08
DBR1	0.02519404	1.1547E-08
FGFR1OP	0.03390641	1.15533E-08
USP27X	-0.1205699	1.16297E-08
AGPAT1	0.02883451	1.16798E-08
DHX16	-0.0114041	1.16838E-08
EDC3	-0.0457528	1.16877E-08
GTPBP3	0.0710595	1.16991E-08
EIF3D	-0.1971914	1.17047E-08
PCBD1	-0.0687776	1.18185E-08
RPGRIP1L	0.05608093	1.18532E-08
AOAH	0.04042427	1.18703E-08
MAN2A1	-0.0326319	1.18707E-08
TSC22D2	0.01250064	1.19745E-08
DNAJB2	-0.0176975	1.20444E-08
ZNF500	0.03757098	1.20572E-08
KTN1-AS1	0.04787753	1.20799E-08
CCDC47	0.03720876	1.22075E-08
TBL3	0.04369489	1.23004E-08
SIGLEC6	0.09278692	1.23334E-08
ABCB10	0.06192029	1.23404E-08
ISG20L2	0.09688776	1.23415E-08
CASP9	0.01851519	1.25115E-08
CNOT4	0.03514203	1.25492E-08
ANKRD52	0.01232894	1.26553E-08
FAIM	0.07354858	1.27584E-08
AC064807.1	0.03592381	1.27933E-08
SIRT1	-0.0410679	1.27971E-08
NONO	0.04075932	1.29439E-08
CRYGD	0.04126348	1.3043E-08
SETD9	0.04883236	1.305E-08
PDE6B	0.07039065	1.3062E-08
AC005726.1	0.05281314	1.31265E-08
MICU2	0.06309258	1.32955E-08
KIF27	0.08840428	1.33157E-08
AC096992.2	0.04475131	1.333E-08
TBC1D24	0.06541641	1.34144E-08
LMNB2	-0.044137	1.34236E-08

TAP2	0.03617045	1.34722E-08
BNIP1	-0.0775462	1.34815E-08
MSN	-0.250401	1.35625E-08
TOGARAM1	-0.1041464	1.36049E-08
ZNF782	0.05838722	1.36138E-08
ZNF384	0.09077907	1.36487E-08
MYD88	0.00808296	1.36491E-08
PEX14	-0.0228026	1.36926E-08
AK3	0.05403502	1.37131E-08
APLF	0.02463	1.3796E-08
SLC9B2	0.05811337	1.38035E-08
AC093690.1	0.06874286	1.38367E-08
MTHFD2	-0.1446731	1.38828E-08
LONP1	0.03510862	1.40242E-08
ADAMTS6	0.06585877	1.40824E-08
BRI3	0.23409113	1.40975E-08
UTRN	0.02426476	1.41018E-08
JAK2	0.07097725	1.41641E-08
SLMAP	-0.1434846	1.41733E-08
INSIG1	-0.2123325	1.4238E-08
AC004889.1	-0.0385562	1.42429E-08
XRCC4	0.08645546	1.42611E-08
CCDC93	-0.0580565	1.42782E-08
CA2	0.12024108	1.43463E-08
AC009961.4	0.0079576	1.43787E-08
CDK8	0.05892553	1.45539E-08
SPG7	0.00886438	1.46274E-08
TIGAR	0.00496719	1.47022E-08
YWHAB	0.22522606	1.4714E-08
PHC1	-0.0302968	1.48722E-08
STX6	0.00012492	1.49122E-08
POLL	0.01862677	1.49469E-08
JADE1	0.07050069	1.49629E-08
FKBP14	0.0771373	1.50309E-08
ANKZF1	0.02197961	1.50444E-08
AL080317.2	0.06958599	1.50755E-08
PSMD4	0.13654607	1.51392E-08
SMG8	0.04615172	1.53408E-08
PRKAR2A	-0.0573988	1.54098E-08
ERI1	-0.0426056	1.54405E-08
PRELID1	-0.2421798	1.54512E-08
RIPK1	0.00496297	1.54675E-08
MTMR10	-0.0726787	1.56928E-08
CEP295	0.07409439	1.56985E-08
WDR5B	0.08476011	1.58052E-08
ZNF254	-0.0141815	1.60055E-08
ITGA3	-0.0852296	1.60352E-08
RDH5	0.03513782	1.60565E-08
ATF5	-0.1204906	1.60964E-08
AL592435.1	0.03507348	1.61605E-08

ZNF780A	0.0549496	1.62604E-08
RABL3	-0.0911605	1.63952E-08
DAB2	0.08585822	1.64768E-08
DAZAP1	-0.0447632	1.65709E-08
DAGLB	-0.0550907	1.65919E-08
ARMH1	-0.0605536	1.66562E-08
AP000845.1	0.02689812	1.67365E-08
KRI1	0.00454144	1.68268E-08
GLMP	0.03266295	1.71148E-08
EXOSC1	-0.0315087	1.71255E-08
ERG	0.16198772	1.73079E-08
RSKR	0.05637949	1.73758E-08
SLC9A6	-0.0154348	1.76491E-08
AGPAT3	0.03235038	1.76708E-08
CCDC121	0.04713696	1.77287E-08
ATG13	-0.0367473	1.78528E-08
TLK1	0.06059417	1.78906E-08
TBC1D17	0.07376768	1.80375E-08
ULK3	-0.0466959	1.80966E-08
AMD1	-0.1700649	1.82569E-08
MCPH1-AS1	0.04188431	1.84018E-08
REV3L	-0.1152182	1.84778E-08
SCRIB	0.01781414	1.84793E-08
PTRH1	-0.0466567	1.85766E-08
DNAJC2	-0.0139551	1.87039E-08
NADSYN1	0.05477731	1.88078E-08
AC120053.1	0.08283088	1.8825E-08
SLTM	0.28614699	1.89171E-08
ZNF468	-0.0017165	1.91266E-08
EIF4G1	0.08886405	1.92257E-08
ZC3HAV1	-0.1267502	1.94147E-08
TNS3	-0.1011901	1.95215E-08
UQCRH	0.16431135	1.9545E-08
PLEK	-0.2585944	1.95472E-08
PILRB	0.01908632	1.9625E-08
AL353807.5	0.05487095	1.96581E-08
ZDHHC6	0.00061825	2.00584E-08
TRIM14	0.04777058	2.0074E-08
MZF1	0.02991674	2.01209E-08
ARHGAP18	0.03044951	2.01889E-08
IWS1	-0.0394326	2.02699E-08
C7orf31	0.04522828	2.02838E-08
DLGAP1-AS	-0.0522956	2.03068E-08
COMMD3	0.09556524	2.06055E-08
IRS2	-0.0088647	2.06057E-08
FAHD1	0.04670058	2.09814E-08
SPART-AS1	0.0521266	2.10614E-08
STON2	-0.102122	2.11492E-08
DHX30	0.02648722	2.11543E-08
PLA2G15	-0.1169179	2.13163E-08

TBCC	0.08758257	2.13538E-08
FAM120B	0.04413838	2.15229E-08
SYTL2	0.05562829	2.16519E-08
XYLT2	0.04885343	2.16608E-08
MAGED2	-0.0098573	2.17688E-08
KDM4A-AS1	0.06419688	2.18239E-08
FGFBP3	0.02622832	2.18565E-08
TIMM21	0.07298605	2.18774E-08
WDR49	-0.1345466	2.19435E-08
MPP1	0.1526182	2.20372E-08
EXOSC4	-0.0130328	2.21726E-08
ABCE1	0.12930531	2.24262E-08
TANGO2	0.00156752	2.26539E-08
GADD45A	-0.0670042	2.28826E-08
UBE4B	0.06885207	2.29197E-08
C5orf30	-0.0581586	2.2969E-08
TFDP2	0.06884708	2.32169E-08
DPP8	-0.017103	2.3251E-08
MPI	0.03806135	2.33014E-08
ZBTB4	-0.0645967	2.33191E-08
CUL2	-0.0466442	2.33216E-08
AC016727.1	0.01672067	2.33629E-08
RELA-DT	0.03361594	2.35316E-08
CHD9	0.12511414	2.356E-08
DNAL4	-0.0548425	2.36237E-08
GTPBP1	-0.0747811	2.37684E-08
MAPK3	-0.0717606	2.37957E-08
ALG1	0.0414322	2.37996E-08
LINC02035	0.03677105	2.38151E-08
RAB4A	0.05633876	2.38708E-08
CCDC51	-0.0091238	2.39862E-08
SYS1	-0.0355754	2.41202E-08
GOLGA2	-0.0111088	2.42193E-08
ARFIP1	0.04461138	2.44332E-08
SPPL2B	-0.0736321	2.44459E-08
JAG1	-0.1281558	2.45323E-08
DECR1	-0.0387713	2.46889E-08
SPI1	-0.1611108	2.4741E-08
ZFP91	-0.0835233	2.47899E-08
PWP1	0.02578225	2.48114E-08
DDT	-0.2016472	2.48633E-08
TMEM205	-0.1710406	2.5027E-08
KCTD5	0.00101068	2.50564E-08
CPSF1	0.04348934	2.50726E-08
CLN3	0.01948496	2.51328E-08
GK5	0.11563059	2.51493E-08
BTN3A3	0.05555376	2.53454E-08
UNK	-0.0517443	2.56999E-08
PCGF6	0.06462398	2.57559E-08
HOXA10	0.11360561	2.58747E-08

WDR55	-0.0183147	2.59822E-08
HMCES	-0.1197087	2.61993E-08
ASB7	-0.0198472	2.62019E-08
ASNSD1	0.06459293	2.62369E-08
A1BG-AS1	-0.0335517	2.63615E-08
CALHM6	0.2174475	2.64414E-08
ADHFE1	0.05090423	2.65192E-08
ENC1	0.04840255	2.65646E-08
RNASET2	-0.1323929	2.66071E-08
ZNF316	-0.0478191	2.66311E-08
PELO	-0.0498331	2.66465E-08
PIP5K1C	-0.0133721	2.66835E-08
TRMT11	0.01977522	2.67514E-08
AC055822.1	0.05030585	2.68001E-08
EEF2KMT	0.07918785	2.68302E-08
RAVER2	0.04303441	2.68656E-08
GTF2H4	-0.0074441	2.68783E-08
EXOG	0.04539018	2.71483E-08
ZNF283	0.05163464	2.72389E-08
PKIG	0.0291612	2.73162E-08
ZMYM5	-0.0790351	2.7337E-08
RAD21	-0.1076211	2.73498E-08
CIAO3	-0.0034905	2.74549E-08
C21orf58	0.09891457	2.75043E-08
FGD3	0.01774476	2.76058E-08
CATSPERG	0.07534752	2.76096E-08
RFC3	0.00475131	2.76368E-08
AHRR	0.05579528	2.76669E-08
CREBL2	0.01978095	2.7738E-08
CARS	-0.1350298	2.77536E-08
MIR99AHG	0.07363423	2.77615E-08
AKAP10	0.05280075	2.77927E-08
FOXP1-IT1	0.00592427	2.79018E-08
DDX51	0.0729799	2.80257E-08
HERC5	0.04863021	2.80546E-08
RAD1	0.00171397	2.82124E-08
COMTD1	-0.1051261	2.82975E-08
HCK	-0.0364384	2.8871E-08
DANCR	0.14377591	2.903E-08
MSH6	-0.0261566	2.91425E-08
GIMAP6	-0.1394484	2.92499E-08
ABI3	-0.1042179	2.92747E-08
LINC01678	0.07926203	2.92872E-08
MAP4K3	-0.0286544	2.93732E-08
IRGQ	-0.1080085	2.94171E-08
COX10	0.00869285	2.9638E-08
MAFG	0.00256366	2.96454E-08
ZFHX2-AS1	0.01730339	2.97247E-08
AC092821.3	0.03439399	2.98839E-08
MYO19	0.03182484	2.99605E-08

VGLL4	-0.1421311	3.00089E-08
ZNF451	-0.0429841	3.0241E-08
NUDT2	0.02741792	3.0359E-08
SNW1	-0.1618097	3.03854E-08
MED28	0.08356549	3.06306E-08
OXSR1	-0.0429929	3.06805E-08
DUS3L	-0.0396375	3.11585E-08
INPP4B	0.11299219	3.11697E-08
FAM221A	0.06318918	3.1675E-08
NDUFA2	0.16809465	3.19822E-08
EIPR1	0.04569058	3.20566E-08
FEZ2	-0.0166081	3.23087E-08
BDP1	-0.0126487	3.23497E-08
BBC3	-0.1208199	3.24236E-08
DPH6	0.05142752	3.2743E-08
OGT	-0.1531346	3.27703E-08
UBE2D4	0.06219294	3.31896E-08
TCF20	0.05768605	3.33935E-08
FAM3A	-0.0337834	3.37389E-08
UFL1	-0.0907933	3.40471E-08
DEPDC5	0.05756156	3.431E-08
ZC3H3	-0.018561	3.43226E-08
AP006621.3	0.038414	3.44387E-08
TOMM34	-0.0245346	3.46017E-08
IGBP1	-0.0217877	3.46133E-08
NCOR1	0.12610159	3.47148E-08
HNRNPA3	-0.2648412	3.49689E-08
MTO1	0.05317936	3.49922E-08
SLC35A5	0.04174753	3.51696E-08
CSNK1G1	-0.0745712	3.56078E-08
ARL14EP	0.07264976	3.57961E-08
UFM1	0.11956708	3.60159E-08
CABLES1	0.06993711	3.62305E-08
PHLPP1	0.00054605	3.64324E-08
MED25	-0.1561471	3.64348E-08
POU2F1	-0.0935796	3.64769E-08
CNNM2	0.0751271	3.68346E-08
LINC02384	-0.0780248	3.68844E-08
IGFBP7	0.10146882	3.71284E-08
NDUFB7	-0.0039054	3.71504E-08
TBRG1	0.01289849	3.71927E-08
RAB21	-0.0757731	3.72645E-08
CENPF	0.06286579	3.7792E-08
UQCRC2	0.04318682	3.79093E-08
RPTOR	0.02195741	3.80242E-08
DESI1	-0.015273	3.81214E-08
TMEM243	-0.0460253	3.83832E-08
RAC2	-0.2331062	3.90688E-08
RHBDD3	0.01870038	4.01206E-08
AMMECR1L	-0.039452	4.03668E-08

AL590764.1	0.02360281	4.05821E-08
TOMM40L	-0.0666719	4.05967E-08
B4GALT4	0.00348608	4.06044E-08
SLC25A20	0.05339332	4.1014E-08
MRPS21	0.13845173	4.11078E-08
STAG1	0.08550788	4.11714E-08
NEDD4	0.0515436	4.12423E-08
SMAD2	-0.0097524	4.14672E-08
CARMIL2	-0.0117389	4.1492E-08
CIPC	0.01101032	4.18496E-08
GALK2	0.11480003	4.23272E-08
ABCA5	0.06038467	4.25697E-08
CCSER2	-0.0059524	4.31941E-08
MEIS1-AS2	-0.042807	4.36944E-08
AMFR	0.00188335	4.39172E-08
SMAD6	0.04803529	4.39872E-08
USP37	-0.0633457	4.39876E-08
ZNF134	0.00408583	4.41371E-08
SNHG12	0.10755098	4.41859E-08
TGDS	0.00877269	4.42868E-08
PLXND1	0.04201737	4.44939E-08
CBY1	-0.0579495	4.47998E-08
TMEM214	0.02513911	4.49941E-08
PGK1	-0.2019455	4.54838E-08
MIR155HG	-0.0093244	4.6421E-08
MLLT10	0.1056644	4.65516E-08
TRIM68	0.05137465	4.66769E-08
SREBF2	0.00420351	4.67053E-08
AL645568.1	0.03881101	4.67393E-08
PHB2	-0.1267104	4.69821E-08
ARL8B	-0.0441999	4.71075E-08
ENTPD5	0.04635711	4.72632E-08
HUS1	-0.0126676	4.73434E-08
AC010623.1	0.02477397	4.73664E-08
SNX14	0.04181321	4.85509E-08
EIF5A	-0.1722358	4.87818E-08
PPARA	0.04323382	4.9164E-08
ZBTB34	-0.0728491	4.96668E-08
MDM4	0.1358191	4.97116E-08
ZNF496	0.03550976	4.98848E-08
MED11	0.01479263	4.98878E-08
NBN	0.0171145	5.03357E-08
PHOSPHO2	-0.0144924	5.05873E-08
TIMM22	-0.0717791	5.10221E-08
ODF2	0.0571239	5.10355E-08
DDX58	0.0888529	5.13423E-08
PHF20L1	0.02383434	5.14481E-08
FAM200A	0.03543611	5.20063E-08
RFX1	-0.0904127	5.2482E-08
MRPL20-AS:	-0.0411326	5.28571E-08

IRF8	-0.2401729	5.32754E-08
LINC02352	0.06494515	5.33229E-08
INPP5F	-0.0256772	5.45387E-08
B3GALT4	-0.1535138	5.45427E-08
ZNF420	0.03016515	5.45808E-08
NMNAT1	0.04644429	5.49085E-08
XRCC5	0.19229449	5.49526E-08
ATP13A1	0.0038676	5.52201E-08
PPIP5K1	0.06871399	5.53574E-08
TXNL4A	-0.0025869	5.53976E-08
KAT2A	-0.0102567	5.54197E-08
TXNL1	-0.0123415	5.55272E-08
BCL9L	-0.0936688	5.56172E-08
KANSL3	-0.0946907	5.56213E-08
SEC11C	-0.0255063	5.56234E-08
SOCS2	0.05015496	5.62316E-08
KLHL11	0.03886073	5.63901E-08
NGLY1	0.03610875	5.77369E-08
SMG1	0.20448112	5.78633E-08
SCYL3	0.03048723	5.79098E-08
ST7L	0.06857667	5.82259E-08
ZMYM2	0.06657936	5.8394E-08
TMEM44-AS	0.02745629	5.86444E-08
E2F5	-0.0447551	5.87241E-08
TMEM181	0.04955318	5.93079E-08
SLC27A1	0.01566302	5.93216E-08
AC135050.6	-0.0009925	5.96864E-08
EPB41L2	0.09896255	5.97714E-08
LRRC42	-0.1004598	5.99491E-08
VEGFA	-0.1304275	6.00655E-08
PIP5K1B	0.0511682	6.044E-08
ARAP1	0.0530799	6.06461E-08
IRF7	-0.0358769	6.09242E-08
IQCG	0.01565594	6.13359E-08
HIRA	0.03076813	6.13751E-08
DDX24	-0.1890944	6.23377E-08
GTF2E1	-0.0445435	6.24708E-08
SLC15A4	-0.1003948	6.29412E-08
DHRS12	-0.0705675	6.30536E-08
CASP4	0.20761657	6.30894E-08
BCORL1	0.01977078	6.32527E-08
AFG1L	0.04916114	6.34235E-08
CASP6	0.16115441	6.34438E-08
SFXN2	0.05646098	6.36975E-08
KCTD6	-0.0526242	6.37039E-08
ZFAND3	0.04967646	6.50624E-08
AC124248.1	0.0716488	6.50993E-08
AL360012.1	0.03475592	6.52663E-08
CRAMP1	0.04448312	6.53593E-08
MUTYH	0.00247185	6.59785E-08

DACH1	0.07826472	6.61389E-08
KIF1B	-0.1020623	6.64129E-08
CNTLN	0.05008287	6.67843E-08
WDR25	0.02429937	6.70655E-08
AL353622.1	0.04671063	6.73378E-08
CDIP1	-0.0107908	6.73567E-08
NDUFB8	-0.1510803	6.75719E-08
PPP1R3F	-0.0076903	6.78634E-08
PTPN23	0.0103371	6.87415E-08
PRKRA	-0.0115233	6.89466E-08
ZRSR2	0.04642826	6.89698E-08
THOP1	-0.0413016	6.90925E-08
SLFN11	0.07756231	6.93566E-08
ARAP2	0.01146758	6.94732E-08
SS18L1	-0.0106223	6.96681E-08
ABO	0.10645116	6.98703E-08
COQ10A	0.05102424	7.00163E-08
TRIM16	0.06187572	7.10861E-08
MTMR3	0.01875334	7.11774E-08
SLC25A24	-0.0186286	7.1259E-08
CALCRL	-0.2265355	7.18736E-08
CERS6	0.06308085	7.23357E-08
PPP3CC	-0.0873331	7.23602E-08
STAT5B	0.06017886	7.27271E-08
ATAT1	0.01054483	7.37441E-08
RNF139	-0.0239185	7.46062E-08
ZBTB8A	-0.1921626	7.47491E-08
BCDIN3D	0.03396839	7.5518E-08
ZNF140	-0.0340025	7.71479E-08
BRK1	-0.2148994	7.76065E-08
DRAM1	-0.0827957	7.76073E-08
CISH	-0.0908626	7.78766E-08
DNAJC14	0.0250532	7.82134E-08
NCEH1	0.04559732	7.8907E-08
CDV3	0.17402107	7.98079E-08
SRRD	-0.0037511	8.01678E-08
AL049840.2	-0.0747658	8.02486E-08
REXO1	-0.0864068	8.03982E-08
PRADC1	0.03596321	8.0481E-08
NDUFAF7	0.04910252	8.07541E-08
ZNF382	0.04080232	8.12807E-08
C6orf136	-0.0182912	8.20317E-08
TPT1	0.13079169	8.24744E-08
FRS2	-0.0097916	8.2704E-08
ATL3	0.04231638	8.28636E-08
DAPP1	-0.1465515	8.29764E-08
MTFMT	0.05379045	8.33819E-08
ZNF836	-0.025868	8.38685E-08
WBP4	-0.1273605	8.3942E-08
HIP1	-0.1631541	8.41594E-08

CMTR2	0.00355992	8.42528E-08
TMF1	-0.1026566	8.44587E-08
SKIV2L	0.01073426	8.46776E-08
SNX12	-0.004605	8.50052E-08
UNC119B	-0.0546313	8.51309E-08
MAP2K1	0.01094351	8.59495E-08
GGCX	0.07318337	8.59879E-08
LIN37	-0.0491227	8.64487E-08
E4F1	-0.0675691	8.68652E-08
PRIM1	0.01930856	8.77898E-08
ATAD5	0.06152062	8.81155E-08
ZNF595	0.03083825	8.85119E-08
MTHFD2L	0.01220634	8.88666E-08
AC007406.5	0.05355808	8.88869E-08
ZNF334	0.00946157	8.8979E-08
ZSCAN16	-0.0677533	8.9239E-08
SLC38A5	0.012351	9.06088E-08
CPEB4	0.09584015	9.1217E-08
CTNNA1	0.07234779	9.22612E-08
DENND1C	0.00376772	9.23913E-08
ARHGAP32	0.05359377	9.25822E-08
METRN	0.05828417	9.31388E-08
RNF208	0.0447359	9.32047E-08
ZNF131	0.03580292	9.36098E-08
NOP16	0.11402067	9.38975E-08
CNDP2	-0.0590662	9.42168E-08
IL3RA	0.08020842	9.43346E-08
AC110769.2	0.03827759	9.47014E-08
NOMO3	0.01719491	9.50552E-08
PSME4	-0.1354615	9.51002E-08
STARD4	-0.0193152	9.52755E-08
MAP3K20	-0.0163657	9.5643E-08
HOXB6	-0.0700508	9.60383E-08
TCTEX1D2	-0.0038627	9.63373E-08
AC093627.5	0.0527525	9.65321E-08
TRMT112	0.11164629	9.81785E-08
ZNF445	0.11024279	9.87356E-08
C1RL-AS1	-0.0132192	9.96233E-08
FAM168A	-0.0097979	9.99573E-08
MARCH8	-0.0674878	1.00054E-07
AC005838.2	0.03221504	1.00357E-07
PAQR6	-0.0189905	1.01836E-07
UAP1L1	0.01846268	1.03481E-07
KSR1	-0.1495875	1.03925E-07
RGS10	0.2760998	1.04064E-07
FDFT1	-0.0470128	1.04961E-07
ZNF330	-0.00636	1.05182E-07
RPUSD1	0.05654684	1.05434E-07
DAD1	0.05173359	1.05965E-07
GPR160	0.05358523	1.06859E-07

PRPF3	0.07307304	1.07586E-07
TADA1	0.01758558	1.07634E-07
ZDHHC12	-0.0372031	1.07732E-07
MMUT	0.02229249	1.08134E-07
BST1	-0.0912529	1.08241E-07
MAU2	0.04894492	1.08526E-07
DIS3L2	0.00572265	1.08582E-07
AC124016.2	0.04362203	1.09351E-07
SRD5A3	0.15847473	1.09703E-07
FAM193A	-0.0429564	1.09828E-07
PLSCR3	0.00111943	1.10634E-07
STBD1	-0.0962271	1.1116E-07
SCYL2	0.00214479	1.11693E-07
AC245884.8	0.03707198	1.11973E-07
RCAN3	-0.1247323	1.12859E-07
DPH3	-0.0638605	1.13599E-07
WARS2	-0.0329621	1.14444E-07
B4GALT3	-0.0697049	1.1486E-07
HSD17B11	0.15610913	1.14898E-07
TRMO	-0.0410071	1.1492E-07
ABHD15	-0.0282067	1.15942E-07
MMS22L	0.09593494	1.16505E-07
NOP9	0.00333724	1.17023E-07
TP53BP1	0.13210933	1.17307E-07
AC008875.3	-0.027038	1.17453E-07
BTG3	-0.0774409	1.18306E-07
RNASEH2C	0.05792523	1.18473E-07
BSG	-0.0328632	1.19647E-07
HS1BP3	-0.0429449	1.19866E-07
UQCRQ	0.16683637	1.20364E-07
INO80E	0.00725755	1.20417E-07
THRIL	0.03879299	1.20677E-07
KLHL42	0.04050112	1.20679E-07
CCT8	0.13781266	1.21197E-07
WAS	0.00541622	1.21238E-07
RBMX2	0.04482436	1.2143E-07
LAPTM4A	0.00654336	1.22455E-07
KIAA1841	-0.1255669	1.22569E-07
AC007336.1	0.08319649	1.23159E-07
SLC22A5	0.03688168	1.23168E-07
FCHO2	0.0290637	1.23964E-07
RETSAT	0.02313401	1.25072E-07
METTL26	-0.0240739	1.25258E-07
GMDS	-0.0101252	1.25286E-07
BSCL2	0.00706357	1.26009E-07
TMEM187	0.02707061	1.26487E-07
FASN	0.07099633	1.26627E-07
TTI2	0.01944741	1.26736E-07
TOM1	-0.0161633	1.27159E-07
PIK3R6	-0.0240894	1.27758E-07

AC092171.3	0.03550154	1.28122E-07
EBLN2	-0.021202	1.28435E-07
CRY2	-0.0468967	1.28642E-07
AC018521.5	-0.0227322	1.29546E-07
KHDC4	0.1030954	1.29766E-07
ZNF398	-0.0201555	1.31038E-07
ALDH16A1	-0.0530119	1.31536E-07
ICK	0.0651811	1.31545E-07
STRN	0.00793272	1.31758E-07
GMEB2	-0.1137349	1.31829E-07
THG1L	0.02713254	1.32581E-07
RPS6KA3	-0.0180213	1.33216E-07
APC	-0.0922743	1.33385E-07
IFI30	-0.1993573	1.33445E-07
PI4K2B	-0.050691	1.33764E-07
PHGDH	-0.1344594	1.33812E-07
KLHL28	-0.0834825	1.34078E-07
NMRK1	0.03246923	1.35834E-07
AL031595.2	0.03800854	1.36172E-07
TMEM14A	0.04269389	1.37324E-07
AC087473.1	0.06083637	1.39431E-07
RBM7	-0.0279075	1.40107E-07
CEP19	0.04970279	1.40333E-07
WARS	-0.0940334	1.41183E-07
LRR1	0.0202345	1.42123E-07
ASCC3	0.04986311	1.42168E-07
ZNF107	0.10084389	1.42761E-07
UTP25	0.07228731	1.43244E-07
FBF1	0.01721076	1.43685E-07
STK35	-0.0799667	1.43845E-07
CLASRP	-0.0056328	1.44627E-07
CD79A	-0.1076138	1.44951E-07
ENY2	-0.0891965	1.45489E-07
MED14	0.01029205	1.46263E-07
ARL6IP5	0.23409071	1.47419E-07
RFX3	0.04944445	1.48055E-07
AL590652.1	0.04750319	1.49319E-07
SNX11	0.01614629	1.51612E-07
TRAK2	0.01745711	1.52608E-07
DENR	0.1470734	1.52962E-07
AKAP9	0.38039752	1.53079E-07
DDHD1	-0.0531475	1.53473E-07
SNX24	0.03946531	1.54281E-07
RAB13	-0.0843711	1.5441E-07
IKZF5	-0.053122	1.55673E-07
ATRIP	0.02137036	1.56332E-07
AGAP2	-0.0534631	1.57369E-07
SPCS2	0.02996015	1.57581E-07
AL451085.2	0.01039663	1.60516E-07
GCLM	0.03404837	1.60994E-07

NORAD	0.1251643	1.63697E-07
PCCA	0.0326307	1.6411E-07
ZNF284	0.04770568	1.64887E-07
NEK3	0.02058122	1.65034E-07
PDE1B	0.01508109	1.65936E-07
CHCHD10	0.18346064	1.66353E-07
RAB11FIP3	0.03023967	1.66416E-07
DUS4L	0.03481371	1.66458E-07
SYNGAP1	-0.0707934	1.68079E-07
BTF3L4	0.04413664	1.68877E-07
POC1B-AS1	0.06054067	1.71655E-07
RBBP9	0.05499633	1.72667E-07
UTP15	-0.0025401	1.73002E-07
MED18	0.00016559	1.75086E-07
ZGPAT	3.8908E-05	1.75511E-07
ACTR3C	0.04782763	1.76147E-07
APH1B	-0.0392292	1.7638E-07
HMGXB3	0.03059641	1.78066E-07
LINC00893	-0.028129	1.78452E-07
ZFYVE1	-0.0005746	1.80103E-07
BANP	-0.0261397	1.8088E-07
CENPV	0.055843	1.81591E-07
AC015712.2	0.01556211	1.84601E-07
TSPYL1	-0.0354436	1.86035E-07
TLE4	-0.1125625	1.8708E-07
FOCAD	0.0473044	1.87272E-07
AL591895.1	-0.0409682	1.87367E-07
INTS5	0.00463751	1.87644E-07
LRRC23	0.05292599	1.89926E-07
ZBTB42	-0.0503441	1.90063E-07
JOSD2	-0.0387963	1.9121E-07
OGFOD1	0.02014065	1.94231E-07
AC073111.4	0.01707322	1.94358E-07
CMC4	-0.0215864	1.96271E-07
ESYT1	-0.0002201	1.97657E-07
AC005523.2	0.03316916	1.98603E-07
CD79B	-0.0838886	1.99548E-07
TMEM161A	0.01940143	1.99571E-07
AC078846.1	0.04777033	2.00503E-07
AC008014.1	0.02523411	2.00676E-07
FKBP11	-0.0645114	2.0207E-07
RWDD1	0.12183839	2.02881E-07
BRF1	-0.0974968	2.03815E-07
ZNF813	0.05350902	2.04905E-07
GIMAP1	-0.0722562	2.06384E-07
AGAP5	0.04308439	2.06788E-07
HOXA5	-0.0136606	2.07162E-07
WDR77	-0.0061455	2.08113E-07
CENPT	0.0064005	2.08239E-07
SND1-IT1	0.04679863	2.09402E-07

PET100	0.12495568	2.11757E-07
CDK5RAP1	0.01393243	2.12387E-07
ANAPC2	0.06097083	2.14799E-07
AC027020.2	0.04913739	2.15073E-07
LINC02175	0.04969951	2.15292E-07
C2CD3	0.00297998	2.16665E-07
OXR1	0.10349093	2.16947E-07
TP53TG1	0.08140493	2.17346E-07
RNPEP	-0.0389448	2.17412E-07
PAK4	-0.0131046	2.17581E-07
DHTKD1	0.08544002	2.18639E-07
TATDN2	-0.0492199	2.19536E-07
MARF1	0.03253243	2.19727E-07
ECD	-0.0661407	2.199E-07
ZNF44	-0.0432765	2.20925E-07
RASGRP4	-0.1211544	2.21306E-07
TTC28	0.0375532	2.21528E-07
KCTD3	-0.0062674	2.23945E-07
CCPG1	0.03439799	2.24579E-07
UBP1	-0.0351348	2.24888E-07
SLC25A36	-0.1550296	2.26245E-07
APRT	0.17668628	2.26746E-07
DHX57	0.0484314	2.29502E-07
STK4	-0.185683	2.30713E-07
TAF1B	-0.0107691	2.30957E-07
PRR14L	0.09878225	2.31962E-07
ZNF543	0.04797824	2.38471E-07
ZNF469	0.02037063	2.38561E-07
RBM3	-0.1902251	2.38804E-07
AC097634.1	0.0218885	2.38987E-07
AC004803.1	0.04181961	2.40711E-07
FPGT	0.05681174	2.42694E-07
JMJD8	-0.0375548	2.43216E-07
VASH1	0.07709024	2.45171E-07
TRUB1	0.02791561	2.45536E-07
LRRC8B	-0.0921322	2.46014E-07
MPZ	0.02734912	2.47052E-07
RANGAP1	0.04126873	2.47127E-07
DUBR	-0.1195027	2.48203E-07
MYL5	-0.0409451	2.49539E-07
GBA2	-0.0536548	2.49655E-07
HECTD3	-0.0161678	2.50477E-07
PCGF1	-0.0246343	2.53716E-07
PCGF5	-0.006661	2.54883E-07
AIG1	-0.0471192	2.55389E-07
TAOK1	0.04457566	2.57243E-07
SETD1A	0.01312911	2.58662E-07
SSX2IP	-0.1060093	2.59322E-07
MERTK	0.06259678	2.59791E-07
IFI16	0.25006213	2.60021E-07

MARK2	-0.0111986	2.61439E-07
NUDT19	0.03872375	2.65477E-07
SELPLG	-0.0348334	2.66206E-07
RBM5	0.01106888	2.69676E-07
AL157938.3	0.04589249	2.708E-07
CLSTN1	0.04923045	2.71198E-07
LTB4R	-0.0943508	2.731E-07
SLC12A2	0.02924709	2.74171E-07
AC147067.1	0.10550707	2.7428E-07
SART3	-0.0416536	2.75301E-07
DYNLT3	-0.0319148	2.75818E-07
USP46	-0.0074086	2.77176E-07
TRIP12	0.17852614	2.7719E-07
ZNF197	0.06193717	2.78099E-07
DDX47	0.02495843	2.78668E-07
RRNAD1	0.05885197	2.7897E-07
ZNF592	-0.0522304	2.82679E-07
TMEM135	0.00638839	2.86259E-07
TSPOAP1-A:	0.01329376	2.86688E-07
YME1L1	0.04174735	2.86925E-07
KDM4C	0.0464146	2.87275E-07
MAP2K2	-0.0299449	2.8873E-07
ARHGEF18	0.00705419	2.89425E-07
LY86	0.00232823	2.91761E-07
HEATR5A	0.04754319	2.92266E-07
GPAM	0.04972416	2.92818E-07
AL731563.3	0.04078607	2.93608E-07
RUFY2	-0.1030229	2.93792E-07
SLC37A4	0.04050818	2.95177E-07
KIF21B	0.02585189	2.95688E-07
POLM	-0.0016329	2.9578E-07
IL6ST	-0.0954684	2.98212E-07
TMEM265	0.06413375	2.98568E-07
RRAGD	0.03440912	2.98957E-07
HLCS	0.04206878	2.99513E-07
URB2	0.01838128	3.00891E-07
SMPD1	-0.0099041	3.02544E-07
PHF19	-0.1495994	3.02875E-07
KIF16B	0.03850495	3.04812E-07
MRM3	-0.0063319	3.05138E-07
AC108488.3	0.0533272	3.06013E-07
ADGRG3	-0.0839137	3.07375E-07
TCTEX1D1	0.06921619	3.08032E-07
CSF2RA	0.09579786	3.09641E-07
DCAF10	0.08592564	3.11046E-07
FYTTD1	0.01133847	3.12017E-07
ADA	0.08976373	3.12228E-07
DBNDD2	-0.0837067	3.12823E-07
CWC15	-0.1085639	3.1314E-07
DDX42	0.04835227	3.1472E-07

STIM1	0.00401845	3.17005E-07
KIAA1522	-0.0850986	3.18371E-07
ZNF486	0.10910038	3.24476E-07
NT5C3A	-0.1121187	3.24963E-07
DNAJC25	0.03295043	3.25292E-07
PSME2	0.06549196	3.26022E-07
RALGAPB	-0.0196107	3.32007E-07
GOLPH3L	-0.0249393	3.368E-07
UPRT	-0.0012737	3.45917E-07
RBAK	0.04248622	3.46261E-07
ZFYVE19	-0.0414091	3.46575E-07
PHB	-0.0545731	3.47628E-07
AC005072.1	-0.0571673	3.49061E-07
PSMA3	-0.1610096	3.50801E-07
CEP135	-0.0357965	3.51651E-07
LINC00672	-0.0783976	3.51803E-07
AC078883.1	0.02050328	3.53799E-07
DNAL1	-0.0539462	3.56011E-07
AC012360.3	0.06656583	3.66496E-07
ZNF510	0.03835041	3.68599E-07
SHANK3	-0.0990022	3.68789E-07
DYTN	0.03969384	3.71055E-07
MTG2	0.00094379	3.71808E-07
TCN2	-0.0853292	3.74841E-07
MSANTD3	0.02947841	3.76001E-07
BICRA	0.01566576	3.76437E-07
ZNF185	-0.0526619	3.78526E-07
ATF7IP2	-0.036592	3.78911E-07
RTL10	-0.0049654	3.81974E-07
SIAH2	-0.0707112	3.82533E-07
RCBTB2	0.06091045	3.84355E-07
HIC2	-0.0068401	3.84439E-07
AKR1B1	-0.1024258	3.85565E-07
RINL	0.04508686	3.901E-07
AC008443.5	0.07054373	3.92641E-07
MPP6	0.03588518	3.92696E-07
NFYA	0.03349593	3.94958E-07
NATD1	0.01249702	3.98332E-07
AP2A2	0.01142091	3.99532E-07
DNASE1	-0.0123186	4.00217E-07
AC073957.3	0.03409354	4.01919E-07
HYAL2	0.01141384	4.02541E-07
CCDC159	0.00258931	4.08427E-07
ZNF292	-0.1692654	4.08614E-07
NEMF	0.11380597	4.1118E-07
DOCK7	0.00156671	4.19436E-07
ABCD3	0.0497021	4.22069E-07
KIF18A	-0.1303634	4.23151E-07
PABPC1L	-0.0420515	4.24563E-07
CMTM2	-0.1401964	4.28074E-07

DCAKD	0.02763704	4.3007E-07
SEMA4A	0.00083511	4.33002E-07
UBXN11	-0.0619544	4.3918E-07
ACSL4	0.14426556	4.54209E-07
SUCO	0.01637825	4.60242E-07
BET1	0.05809291	4.61398E-07
OGDH	-0.0124158	4.63539E-07
SNRPD2	0.18732865	4.71151E-07
MKS1	0.02129833	4.72841E-07
SERPINB6	0.10950272	4.74866E-07
TBCCD1	0.04580008	4.75284E-07
RPS27A	0.11857048	4.76924E-07
PLOD3	-0.0695532	4.77661E-07
PCNP	-0.0258729	4.79374E-07
LIPE-AS1	0.0445687	4.8132E-07
ZNF829	0.03409088	4.81608E-07
CD9	-0.1258816	4.82783E-07
CLTA	-0.1765788	4.83556E-07
AC103691.1	0.02740383	4.85179E-07
C1QTNF6	0.05213651	4.89701E-07
FAM174A	0.02313826	4.96269E-07
AC005674.2	-0.0045354	4.97874E-07
CEP89	-0.0206947	5.00292E-07
IPMK	-0.0201197	5.10698E-07
AC011477.2	0.03264409	5.1716E-07
PIAS3	0.0193873	5.19954E-07
BMP1	-0.0390483	5.21069E-07
MRM1	0.04319329	5.21456E-07
SIDT2	0.01042818	5.22014E-07
SELENOS	-0.0773264	5.30853E-07
GZF1	-0.0041688	5.33282E-07
PNO1	0.07694607	5.35788E-07
PRDM11	0.00225253	5.42315E-07
TRMT10A	0.04310183	5.45269E-07
IKBIP	0.10651533	5.46026E-07
PEX19	0.00589351	5.46141E-07
CIAO2B	-0.049997	5.49659E-07
SNAPC2	-0.0790447	5.49807E-07
MLST8	0.02878336	5.55629E-07
PPM1M	0.03212888	5.55677E-07
ZCRB1	0.02410801	5.56725E-07
CTSO	0.07722218	5.60197E-07
PRKACA	0.05000886	5.61089E-07
DHDDS	-0.0015428	5.64314E-07
CXorf40A	-0.0522345	5.65155E-07
NIPSNAP1	0.0418343	5.65741E-07
BRMS1L	0.04809647	5.69531E-07
ZNF23	0.06100193	5.69591E-07
TBC1D16	0.04549696	5.70795E-07
SLC66A1	-0.0207413	5.79216E-07

ZNF850	0.03824439	5.86871E-07
PAG1	-0.057902	5.8808E-07
RPUSD2	-0.0316492	5.96084E-07
MT-CYB	0.21804094	5.96983E-07
UBAP2	0.04852662	5.97182E-07
ORMDL3	0.02906423	5.99046E-07
UBE2V1	0.04607518	6.01219E-07
HYI	0.03028746	6.0911E-07
WAKMAR2	0.00417203	6.09529E-07
ZNF845	0.02100395	6.16213E-07
INPP1	-0.0557268	6.16436E-07
SHLD1	-0.0804964	6.19305E-07
AC009506.1	0.02632586	6.19809E-07
TRIM26	-0.0614835	6.20232E-07
PEA15	-0.0583388	6.22956E-07
RGS12	0.05731896	6.2491E-07
FBXL6	-0.0594119	6.26073E-07
HIST1H1E	0.19149773	6.28222E-07
BACE1	-0.0189879	6.29539E-07
TOR1B	-0.0173478	6.32304E-07
ZNF217	0.03575914	6.36288E-07
SNHG26	0.10520478	6.36318E-07
COX20	0.06081597	6.38173E-07
MBTPS2	0.03264109	6.3869E-07
AC068888.1	0.02660796	6.38757E-07
AC007325.4	0.01655286	6.40934E-07
PRCC	-0.0731347	6.42919E-07
LDB1	0.05409075	6.43602E-07
PAFAH1B3	-0.006404	6.44682E-07
AC240274.1	0.0480767	6.45027E-07
WDR11	0.10022244	6.45441E-07
ZBTB48	-0.0073349	6.47834E-07
FSBP	0.03764487	6.48408E-07
MS4A7	-0.1276104	6.4955E-07
AMZ2	0.04355924	6.52113E-07
ZNF668	0.0271395	6.52467E-07
NDUFB9	-0.125216	6.54692E-07
HLA-DPB1	-0.2900944	6.58461E-07
SMN2	0.03802827	6.7291E-07
ZNF432	0.0298883	6.75535E-07
CENPP	0.02797954	6.85066E-07
KLHL36	-0.0288186	6.90835E-07
GATD1	0.00352882	6.90964E-07
MAP4K2	-0.0625407	6.95911E-07
RHNO1	0.00125955	7.00106E-07
MRPL48	0.04818157	7.00764E-07
ABHD4	-0.0093125	7.03047E-07
ZBED5-AS1	0.0755004	7.08532E-07
MTCL1	0.0534919	7.09434E-07
CLCN6	-0.0424282	7.09851E-07

ZCCHC10	0.00044618	7.1145E-07
PSMA7	-0.1548572	7.11893E-07
TBC1D31	0.01350032	7.12581E-07
CHAF1A	-0.1020038	7.13001E-07
AARS	-0.0065635	7.13604E-07
C2CD5	0.01637827	7.14753E-07
AC146944.4	0.03770434	7.24339E-07
IFIT2	0.00479632	7.25962E-07
ZBTB10	-0.0081576	7.29354E-07
SNX1	0.00475073	7.31662E-07
INVS	0.03225538	7.32551E-07
GALC	0.02470749	7.38181E-07
ZNF75D	0.05561317	7.44075E-07
LINC01134	0.05287137	7.48853E-07
SLC25A1	-0.0247657	7.65237E-07
SREK1	0.12934007	7.71429E-07
GPD1L	0.04132925	7.7967E-07
ZNF644	0.00520889	7.85229E-07
ELMO2	-0.0652451	7.8783E-07
AP4B1	-0.0260146	7.92269E-07
PPP5C	0.02703666	8.01736E-07
B4GALT7	-0.0446024	8.10937E-07
SLC15A2	0.01493635	8.1193E-07
TMEM185A	-0.0891873	8.17058E-07
ANKRD44	-0.0102428	8.23549E-07
MED24	0.02654575	8.25908E-07
ZNF852	0.05743552	8.28425E-07
SLC35G2	-0.0739641	8.34342E-07
AC073896.3	0.03145781	8.37877E-07
ZBTB39	0.02529092	8.47137E-07
ALDOC	0.07549073	8.51528E-07
FGD5-AS1	0.03332611	8.54829E-07
FKBP3	0.05546333	8.57137E-07
AC009779.2	0.02248169	8.65893E-07
SLC45A4	-0.1044627	8.68506E-07
PARD6A	-0.0669146	8.69297E-07
VIM-AS1	-0.2343824	8.70472E-07
GALNT2	0.12390077	8.80547E-07
RBM10	0.02406641	8.80865E-07
HELZ2	-0.0964718	8.82319E-07
GFER	-0.0216955	8.88721E-07
SIRT3	-0.028245	9.07643E-07
ANKRD36	0.06601705	9.08526E-07
CHMP7	-0.0076623	9.17759E-07
RBBP6	0.17883842	9.19088E-07
TRIM13	0.03657664	9.20893E-07
MAP3K2-DT	0.07818379	9.23624E-07
ANXA2R	-0.0779191	9.24199E-07
FAM153CP	0.03218811	9.27349E-07
LCLAT1	0.02968883	9.27457E-07

ANKRD44-IT	-0.0176491	9.27895E-07
C15orf65	-0.0964507	9.39378E-07
ZNF324B	0.00541784	9.48287E-07
CBX5	0.11902953	9.49247E-07
UVSSA	0.07200648	9.59871E-07
INO80	-0.0225194	9.67623E-07
ZEB1-AS1	-0.0697366	9.67729E-07
ERCC8	0.03601897	9.70426E-07
ZNF251	0.01899894	9.73642E-07
FAF1	0.09380411	9.76056E-07
SF3B2	0.12608756	9.77211E-07
PVT1	0.06650041	9.83671E-07
CA3-AS1	0.04518119	9.8406E-07
AC246817.2	0.03440073	9.97305E-07
ETS2	-0.3114997	1.00247E-06
GSS	0.03926376	1.00561E-06
SLITRK4	0.04963036	1.01696E-06
PKNOX1	0.0051565	1.01736E-06
SEC31A	0.07007427	1.02265E-06
ABCF3	-0.0332063	1.02443E-06
TECPR2	0.03510325	1.03922E-06
USP38	0.00035458	1.04141E-06
KPNA5	-0.029631	1.04319E-06
UGDH	0.04655172	1.04663E-06
ZC3H13	0.13985218	1.04919E-06
WDR19	0.03561474	1.06722E-06
SNX30	0.07192695	1.07503E-06
RBSN	-0.0250655	1.07585E-06
H2AFV	0.17887563	1.07642E-06
GCC1	-0.0085178	1.07698E-06
NEFH	0.06823765	1.07803E-06
PYROXD2	0.04001341	1.08399E-06
HAGHL	0.06223685	1.08492E-06
FUZ	-0.0899826	1.0974E-06
SMPD4	-0.0303966	1.09937E-06
PMAIP1	-0.2798938	1.10372E-06
ZNF329	-0.035855	1.10386E-06
TMEM67	-0.1009796	1.10858E-06
PRPF38B	0.19629373	1.11302E-06
PATL2	0.00308278	1.11377E-06
TTC31	0.00884322	1.13167E-06
ERBIN	-0.130794	1.15912E-06
GPAT4	-0.0537982	1.16001E-06
CCDC84	0.00881816	1.16004E-06
CPQ	-0.0368434	1.16577E-06
ZNF180	0.0168366	1.16857E-06
FEM1A	0.04627764	1.18409E-06
ATG4A	0.01762377	1.1877E-06
BOLA3	-0.0016915	1.1914E-06
NPEPL1	-0.002586	1.19394E-06

SH3RF1	-0.0890663	1.20163E-06
PLD4	0.12607238	1.20512E-06
ZNF790	0.02621116	1.2106E-06
SIK2	-0.0216545	1.21297E-06
AC010745.5	0.0641189	1.22422E-06
MTHFSD	0.03477525	1.22598E-06
ZFAT	0.03332537	1.23514E-06
RANBP3	-0.0292478	1.24683E-06
POMT1	0.03773598	1.24762E-06
PROCA1	0.00431837	1.24865E-06
SLC4A7	0.08620552	1.25528E-06
RPS5	-0.2735328	1.25623E-06
RNF26	0.02151578	1.27827E-06
TMEM238	0.0378645	1.27967E-06
NBPF9	0.01623977	1.2817E-06
PNPLA2	-0.030371	1.28335E-06
MACC1	0.02497925	1.29642E-06
SCYL1	-0.0529783	1.30621E-06
ATXN7L3B	0.05505004	1.31054E-06
GEMIN8	-0.0117608	1.31545E-06
WDR44	-0.059634	1.3188E-06
TRIM34	0.07650267	1.31907E-06
WDR53	0.006665	1.32064E-06
ACAD11	-0.0028733	1.32927E-06
DIAPH2	0.15973636	1.33218E-06
KLC4	0.01951125	1.33582E-06
RNF144B	0.0503772	1.34626E-06
UBE3B	0.01348532	1.34648E-06
AL513550.1	0.04419868	1.35028E-06
KATNAL1	-0.0137255	1.35038E-06
AL157895.1	0.31377099	1.35851E-06
NDEL1	-0.1114225	1.36257E-06
C10orf25	0.05038562	1.40766E-06
BTG2	-0.2459652	1.41155E-06
PIAS4	-0.1237058	1.41348E-06
DUSP2	-0.0652514	1.42646E-06
ZNF627	-0.0114418	1.43346E-06
FLOT1	-0.0412362	1.4504E-06
NAALADL1	0.0515544	1.46498E-06
MSTO1	0.00695087	1.47124E-06
PPBP	-0.3462567	1.47609E-06
TCF25	0.07795	1.48397E-06
AL132709.8	0.05219163	1.48459E-06
SMYD2	-0.0198199	1.49221E-06
TEX22	0.01840184	1.49499E-06
GOLGA1	0.02152361	1.49653E-06
SH2B2	-0.1334127	1.50585E-06
TYROBP	0.30697534	1.51566E-06
FAAP100	-0.00158	1.51662E-06
ZSCAN31	0.03930811	1.52604E-06

KBTBD4	-0.0423431	1.53548E-06
DOP1B	0.02650157	1.54027E-06
TOP3A	0.02067846	1.55297E-06
AC027682.4	0.04227755	1.55774E-06
ESCO1	-0.0943455	1.55876E-06
SLC35D2	0.00607985	1.56273E-06
IDUA	0.02235973	1.57632E-06
UBL5	0.22154426	1.58161E-06
TOB1	-0.0569163	1.60087E-06
CTNNAL1	-0.0038008	1.60472E-06
NPR3	0.0280881	1.60518E-06
CASTOR1	0.01152219	1.61477E-06
FMR1-IT1	0.03550132	1.62099E-06
AC005520.2	0.02051312	1.62878E-06
BCL2L1	-0.0112592	1.6342E-06
DHFR2	0.05418384	1.6345E-06
BORCS6	-0.0214442	1.67642E-06
NICN1	-0.0018134	1.68313E-06
NIPSNAP3B	0.06573569	1.7092E-06
INPP5K	0.01401633	1.71005E-06
FCHO1	0.06602087	1.73833E-06
ZNF211	-0.0437767	1.73966E-06
ADAT1	0.00925918	1.75862E-06
PSMB10	0.05738509	1.77842E-06
NLRP1	-0.1384631	1.78115E-06
MIER3	-0.0181569	1.78282E-06
SLC35A2	-0.0871058	1.78824E-06
DYNLL2	-0.0615646	1.79338E-06
ARMC7	-0.0248605	1.79835E-06
SNX32	0.03722585	1.80756E-06
UBR1	0.00025413	1.80771E-06
XRCC6	-0.0796679	1.81128E-06
C16orf70	0.01828374	1.81518E-06
CYTH3	-0.0390912	1.82271E-06
LINC00884	0.04570636	1.83187E-06
SMCO4	0.0030511	1.83456E-06
TENT5C	0.09229095	1.84165E-06
NTAN1	-0.053033	1.87045E-06
ATP5MG	0.16503711	1.89751E-06
CD36	-0.1692232	1.90508E-06
DNASE1L1	-0.0764353	1.90778E-06
NSDHL	-0.0435456	1.90934E-06
TMEM186	0.01063945	1.93062E-06
SEC24C	-0.0547497	1.93188E-06
STAC3	0.05460534	1.93355E-06
AC104162.2	-0.0015677	1.93602E-06
NPIPA1	0.06392652	1.96937E-06
OSBPL3	-0.0352547	1.97007E-06
TRMT1L	0.06971541	1.97665E-06
ACAA2	0.00782293	1.97706E-06

CHM	0.09957947	1.9921E-06
C3orf62	-0.0387269	2.01555E-06
AC010615.2	0.05873377	2.01557E-06
MZT2B	-0.2229599	2.02097E-06
NUP50-DT	0.03536409	2.05123E-06
PES1	0.01272201	2.07024E-06
NUDT3	0.08008014	2.07787E-06
HOOK3	-0.0896804	2.07981E-06
TTC39C	-0.1045243	2.08621E-06
ARFIP2	-0.0200519	2.09698E-06
HIST1H2AE	-0.1070697	2.11607E-06
SBF1	-0.0525206	2.13009E-06
TMEM65	0.04298548	2.13639E-06
MRPL51	-0.0036526	2.15059E-06
BID	-0.0516504	2.15156E-06
TMEM191B	0.025149	2.16332E-06
NDUFB2	0.13051367	2.18061E-06
AGBL3	0.04710274	2.18158E-06
AC135507.1	0.01584339	2.18971E-06
ACOT8	-0.0085163	2.19264E-06
PDP1	-0.0360886	2.20842E-06
TRMT5	0.04491886	2.21754E-06
TRIM66	0.05807012	2.22982E-06
C15orf41	0.03808413	2.23345E-06
AC021054.1	0.04385889	2.27232E-06
PDCD4-AS1	0.09278363	2.30906E-06
TUBGCP6	-0.0090804	2.33755E-06
MDFI	0.04749653	2.34892E-06
RUSC1	-0.0297848	2.38004E-06
CHD3	0.09202431	2.41114E-06
NR4A1	-0.1291456	2.43003E-06
CSAD	0.0060371	2.45195E-06
KLHL21	-0.0119173	2.4679E-06
AC016588.2	0.03902169	2.4689E-06
ADGRG5	-0.0797996	2.48845E-06
TRAPPC9	-0.0051331	2.50007E-06
TYW3	0.04482628	2.50871E-06
MED13L	-0.0861763	2.51478E-06
ZUP1	-0.0947521	2.51629E-06
TCF4	-0.2122001	2.52555E-06
ZFYVE26	0.02972508	2.52825E-06
PLOD1	0.0455892	2.54599E-06
BLOC1S3	-0.0354088	2.55619E-06
U2AF1L5	0.03319775	2.55959E-06
STT3B	-0.1290601	2.56082E-06
IQCE	0.02843779	2.57558E-06
LRSAM1	0.02276358	2.5901E-06
PEX7	0.04541344	2.59179E-06
RAD9A	-0.0358111	2.59407E-06
RBM26	0.15738	2.59676E-06

PDCD2L	0.05113896	2.60123E-06
GPRC5C	-0.0722371	2.60409E-06
PRUNE1	0.00214623	2.61272E-06
VANGL1	-0.2011486	2.62472E-06
TOMM22	0.05624118	2.6943E-06
LIN52	0.02312864	2.69527E-06
TRPV1	0.04343938	2.69823E-06
DNAJC12	0.06907448	2.70262E-06
ZNF672	0.0225741	2.71396E-06
AC145207.5	0.04607919	2.72198E-06
SCAF8	-0.0490474	2.76417E-06
STAG2	-0.0129429	2.77498E-06
ZBTB5	-0.0139559	2.79497E-06
LRRC40	-0.0190828	2.81709E-06
U2AF1	0.02813302	2.82551E-06
EIF3J	0.11502476	2.86229E-06
CC2D1B	-0.0744584	2.88921E-06
DZIP3	0.00853569	2.89011E-06
SPTY2D1OS	0.04832794	2.91703E-06
APTR	-0.0582283	2.92844E-06
TMEM199	0.02224377	2.93383E-06
PPP1R37	-0.0222443	2.94705E-06
SYVN1	-0.13472	2.97302E-06
SPTLC2	-0.1020855	2.98493E-06
DEK	0.11309777	2.98909E-06
TARDBP	-0.2068674	3.00658E-06
OAS2	0.02871441	3.01366E-06
MVD	-0.0729048	3.01845E-06
AL353804.1	0.03010144	3.02948E-06
TMED2-DT	0.06883649	3.06997E-06
AC087190.1	-0.0057437	3.07042E-06
PDK2	0.02120529	3.08984E-06
RAB28	0.0385915	3.09307E-06
ITPKB	-0.0518145	3.12411E-06
ROGDI	-0.0458299	3.13038E-06
AC093323.1	0.07490034	3.20483E-06
KLHL12	-0.0174299	3.22253E-06
FLVCR1-DT	0.06357233	3.24474E-06
AL162458.1	0.01390306	3.27863E-06
PITPNM1	0.04290829	3.2911E-06
ZNF354C	0.04531839	3.29336E-06
AC008443.1	0.00940098	3.29391E-06
GSTM4	-0.0056918	3.30452E-06
WSB1	0.05864967	3.30967E-06
ABHD11	-0.0132151	3.32943E-06
DNAJC8	0.13481333	3.34951E-06
SMC3	-0.0264374	3.37739E-06
DNM1	-0.0046312	3.40667E-06
EME2	-0.0056451	3.41099E-06
BAP1	-0.0089164	3.41217E-06

ACTR8	0.02195757	3.4484E-06
TRIM35	-0.0391474	3.46058E-06
PCSK7	-0.0563123	3.46864E-06
ABR	-0.036439	3.52429E-06
S100B	-0.113648	3.5486E-06
AC009404.1	0.0469811	3.56061E-06
LDLRAP1	-0.0301543	3.56113E-06
BBS4	0.04328639	3.56352E-06
RBM19	0.03228704	3.58706E-06
PLAGL1	0.02965906	3.58787E-06
C2orf42	0.03537925	3.59039E-06
AP002360.1	0.04122171	3.62021E-06
CCNYL1	-0.0502501	3.64111E-06
ZNF865	0.04101474	3.64789E-06
WDR91	-0.005676	3.65793E-06
AL096865.1	0.0104213	3.69016E-06
WWOX	0.01475761	3.7348E-06
KMT2D	0.04735285	3.73714E-06
CYB561D1	0.04847108	3.75467E-06
AC025171.3	-0.016283	3.81453E-06
TRIM56	0.18963918	3.81747E-06
MAST2	-0.004249	3.84049E-06
LINC01023	-0.0933249	3.86197E-06
ZSWIM1	0.04094782	3.86303E-06
ALKBH6	0.01399175	3.86635E-06
IKZF4	0.0434973	3.877E-06
IFI44	-0.0432147	3.88373E-06
ZNF317	-0.00111	3.88568E-06
HNRNPR	0.17235496	3.88921E-06
AGTPBP1	0.03090276	3.90163E-06
HSDL1	0.0249626	3.90621E-06
HSBP1	-0.0269062	3.91593E-06
CCNB1	0.02166736	3.91938E-06
TRAF3IP2-A	0.01313877	3.94617E-06
TTI1	0.0228781	3.94964E-06
ARAF	-0.0350129	3.95253E-06
MAP3K3	-0.0700521	3.98408E-06
ATAD3A	0.00721722	4.0265E-06
KPTN	-0.0901247	4.0431E-06
ENGASE	0.03094586	4.06993E-06
GNE	0.02354881	4.1259E-06
TPRN	0.04227592	4.13714E-06
ZNF775	-0.1083746	4.13836E-06
NECAB3	0.01521458	4.14109E-06
PTDSS2	-0.0040653	4.15299E-06
ATP5F1E	0.20384198	4.16072E-06
WDR5	0.00771539	4.16581E-06
AL161421.1	0.03916101	4.16691E-06
CLHC1	0.08340443	4.16836E-06
AOPEP	0.00273421	4.2605E-06

AP001059.3	-0.0100854	4.28816E-06
AKR1C3	-0.0104048	4.31046E-06
TRAFD1	0.00993004	4.31229E-06
UEVLD	0.02340874	4.3146E-06
ZNF22	0.10749949	4.33183E-06
C16orf86	-0.0102727	4.33357E-06
TSC1	-0.0342977	4.34317E-06
KDM5A	-0.0586094	4.34968E-06
TMEM102	-0.0857162	4.42914E-06
TOP1	-0.0121172	4.43854E-06
HNRNPH3	0.05890668	4.46945E-06
HSPA14	0.01237887	4.60483E-06
THAP3	-0.0301228	4.62837E-06
NT5DC2	0.04713845	4.63721E-06
GORASP1	-0.0046483	4.66671E-06
NBPF11	0.0376118	4.68816E-06
ZNF136	-0.0044372	4.68856E-06
AC006064.4	0.02920763	4.70967E-06
ERICH6-AS1	-0.1101976	4.75654E-06
DHRS7	0.0671494	4.79671E-06
SENP3	0.01180293	4.7982E-06
NFS1	0.02578708	4.81734E-06
ABHD5	0.04082698	4.82177E-06
MTCH2	5.2102E-05	4.82509E-06
GTF2IRD1	0.04487984	4.84265E-06
ARF5	0.06650903	4.85605E-06
SV2A	-0.0424775	4.88059E-06
C18orf32	0.05838651	4.91084E-06
AC234772.2	0.01414145	4.91972E-06
RUSC2	-0.0424411	4.9333E-06
SEC24A	-0.047355	4.94556E-06
PSMA1	0.09546315	4.96102E-06
EIF2S2	-0.1519864	4.96598E-06
DNAAF4	0.06609026	4.98102E-06
NR1D1	0.04431833	4.98794E-06
TXNDC16	0.03808963	5.00937E-06
ACCS	0.0102739	5.03952E-06
SIMC1	0.03840554	5.04143E-06
ZNF677	0.04574412	5.1035E-06
ANAPC11	0.1349827	5.11588E-06
MAP4K4	0.06861237	5.11617E-06
RAP2A	0.01994554	5.14095E-06
DNAJC18	-0.0663807	5.23375E-06
TCEAL4	0.14728477	5.2366E-06
TRAPPC13	-0.0112912	5.2436E-06
LIN9	0.05834313	5.27583E-06
SNRPD1	0.05306514	5.2794E-06
TRMT9B	0.05379422	5.32019E-06
SMAD1	-0.0345632	5.33255E-06
RAP2C-AS1	-0.0062894	5.37704E-06

SAYSD1	0.0365779	5.38238E-06
MLYCD	0.06817443	5.51786E-06
ENTPD4	0.0091391	5.52036E-06
AC090204.1	0.05251412	5.54904E-06
GRK4	0.01322614	5.56211E-06
LINC01534	0.03460357	5.56739E-06
ICA1L	-0.03403	5.68275E-06
PANK2	-0.0931983	5.6865E-06
ZNF786	0.01062084	5.69649E-06
TAF10	-0.0423837	5.7055E-06
TOE1	0.05908092	5.77272E-06
TRAM1	-0.0377052	5.81941E-06
AL158212.3	0.03888957	5.83069E-06
AL355377.3	0.03229676	5.86916E-06
SUGP2	0.0929749	5.87378E-06
SINHCAF	-0.0718614	5.88997E-06
NAA40	0.00778064	5.90905E-06
SMUG1	0.03332207	5.91662E-06
TPMT	0.05288669	5.95121E-06
DDX6	-0.0023275	5.96938E-06
MTDH	-0.2098308	5.97333E-06
TECPR1	0.0035594	5.97732E-06
AC107214.1	0.03424185	6.01889E-06
HOTAIRM1	0.0044286	6.10117E-06
TRIR	-0.1663445	6.12776E-06
NME2	0.18374248	6.17659E-06
VPS33B	-0.048856	6.19031E-06
MTMR2	-0.032053	6.20461E-06
SRSF10	0.05987208	6.21019E-06
INTS1	-0.0423068	6.30443E-06
GTPBP2	0.02334443	6.31359E-06
ZNF781	0.04320618	6.33262E-06
VCL	-0.1004392	6.36938E-06
SNRPB2	0.09249303	6.39178E-06
PEX10	-0.0100968	6.47331E-06
LAMB2	-0.0676953	6.47855E-06
MEF2A	-0.1228026	6.49453E-06
MTA3	-0.036469	6.50364E-06
ACOT2	0.01235116	6.51686E-06
PPP1R12A	-0.1694146	6.56325E-06
LYST	0.26891155	6.59309E-06
LINC00921	0.01501752	6.63357E-06
ZNF212	-0.0335919	6.65251E-06
KLHL26	-0.0831601	6.68216E-06
NIPAL3	-0.0249045	6.69037E-06
PDSS1	0.01069328	6.75325E-06
DUSP5	-0.1385656	6.79111E-06
YJU2	-0.0565128	6.83935E-06
RAPGEF6	-0.0579989	6.84525E-06
CDKAL1	0.03920873	6.85289E-06

AC008771.1	0.03865706	6.89726E-06
ASH1L-AS1	-0.0456428	6.92832E-06
AGMAT	0.04559823	6.93928E-06
STARD3	-0.0325734	6.96404E-06
RHBDD2	-0.112042	6.97076E-06
EPS15	0.12211439	6.99359E-06
PRPF40A	-0.1915979	7.03975E-06
ZG16B	-0.1194803	7.05451E-06
SEPSECS-A	0.06378275	7.06418E-06
SEC24D	-0.0030527	7.06582E-06
ELANE	0.27672771	7.08399E-06
CLIC3	-0.1275297	7.09651E-06
HNRNPM	0.16956113	7.10665E-06
CPEB2	-0.0370047	7.13044E-06
OPTN	-0.1278077	7.14972E-06
AC104532.2	0.01091389	7.27853E-06
TTYH3	-0.0383389	7.36991E-06
LINC00923	0.04509176	7.40807E-06
LBR	-0.0973212	7.51351E-06
ACOX3	0.01925643	7.52254E-06
TBCE.1	-0.0064128	7.53237E-06
NBPF1	0.02873219	7.55601E-06
PCTP	0.04278156	7.60693E-06
PSMB8	-0.0224632	7.6087E-06
TAF6L	0.02137351	7.61774E-06
GLIDR	0.03518745	7.7023E-06
GPN2	-0.0241588	7.85291E-06
AL135791.1	0.03628155	7.89182E-06
KLHL2	-0.042384	7.89224E-06
ZNF525	0.05003052	7.90563E-06
CRAT	0.02653724	8.01196E-06
TAOK2	-0.0102293	8.01436E-06
SH3BP5L	-0.0338365	8.0494E-06
PRPF4B	-0.1351656	8.05888E-06
NUBPL	0.05717582	8.16566E-06
GIMAP8	0.02370887	8.21099E-06
ZNF789	-0.0245345	8.29296E-06
FAM8A1	0.00339096	8.32579E-06
GNB1	0.02422981	8.39724E-06
RABEP2	-0.127627	8.39802E-06
ATP5MF	0.14485629	8.49673E-06
AL356019.2	0.05090969	8.50925E-06
EID3	0.02299294	8.51103E-06
UBE2I	-0.1714829	8.5657E-06
NBPF12	0.06017058	8.60929E-06
RASGEF1A	-0.0155707	8.61131E-06
PNMA6A	0.03652671	8.66762E-06
AC010680.4	0.0295968	8.70596E-06
PSMD8	-0.1410979	8.78456E-06
AC006141.1	0.11361993	8.79605E-06

SSH3	0.00926381	8.84828E-06
LPXN	0.00954476	8.88769E-06
KIF13A	-0.0010421	8.93325E-06
CLOCK	0.05207663	9.06064E-06
TXNL4B	-0.0017192	9.06216E-06
TTLL1	0.050355	9.09579E-06
LARS2	-0.0027854	9.12259E-06
RAB33B	-0.0068445	9.13441E-06
ZNF347	0.05877699	9.18767E-06
SOD1	-0.1504386	9.19646E-06
TPP2	0.04749435	9.25167E-06
CENPL	0.02427912	9.27852E-06
FMR1	-0.0008368	9.29942E-06
SELENOI	-0.0147431	9.30428E-06
ATXN7L3	0.02294954	9.32448E-06
SIRT6	-0.0086226	9.33162E-06
PIGZ	0.03933063	9.44848E-06
AC243960.1	-0.0041684	9.5545E-06
NFIX	0.05429995	9.5692E-06
MEPCE	-0.0043901	9.58462E-06
MARCH1	-0.1430509	9.64711E-06
DALRD3	-0.1216236	9.661E-06
RAB31	-0.1839014	9.67643E-06
PARP6	-0.0509322	9.68048E-06
GSTK1	-0.1708846	9.80838E-06
SMG9	-0.0040762	9.90025E-06
MFHAS1	0.03962298	9.94194E-06
AGXT	0.05365051	9.9939E-06
PROSER3	-0.0502013	1.01211E-05
CCL28	0.04642595	1.01228E-05
C19orf54	0.01841899	1.01749E-05
DUSP7	-0.0669505	1.02284E-05
FGFR1OP2	-0.0021303	1.02328E-05
PPM1L	-0.0037404	1.02707E-05
RACGAP1	0.03604875	1.02939E-05
GADD45GIP	-0.1111182	1.03494E-05
TRDMT1	-0.0243492	1.03516E-05
LRCH1	-0.0445748	1.03839E-05
ZNF133	0.01319087	1.04474E-05
ZMAT2	0.02334408	1.04478E-05
ANGEL1	0.03040408	1.06116E-05
TTC24	-0.0120697	1.0653E-05
RAB10	-0.0985097	1.07031E-05
PARP15	0.01659519	1.0752E-05
AC124283.1	0.01326701	1.07811E-05
SWT1	0.01758616	1.08763E-05
AC015871.7	0.01281313	1.0881E-05
ZNF219	0.01899571	1.10272E-05
HCG25	0.06266013	1.11036E-05
ANKFY1	0.03392902	1.11425E-05

IL18	0.22378245	1.11483E-05
AGBL5	-0.0451502	1.11787E-05
AC009120.2	0.0623932	1.12015E-05
BBX	0.0082872	1.12345E-05
C6orf163	0.04201708	1.12883E-05
AC008982.2	0.03621809	1.13028E-05
SLF1	0.00914871	1.13524E-05
MAP4K1	-0.0135861	1.16005E-05
CASZ1	-0.0727667	1.17097E-05
CCDC88A	0.14431418	1.17232E-05
ZNF436-AS1	-0.0164447	1.17749E-05
NET1	0.06078711	1.17821E-05
AC103702.1	-0.0885285	1.17996E-05
TNFAIP1	-0.0781404	1.1814E-05
AC009118.3	0.03472805	1.1816E-05
USP21	0.01938477	1.18191E-05
AC003681.1	0.04848906	1.18339E-05
LMLN	0.0325022	1.1834E-05
ISG20	-0.1381946	1.19333E-05
ATP5F1B	-0.2003549	1.19344E-05
DHX35	0.01424175	1.19745E-05
LY6G5B	0.02010882	1.21062E-05
FRMD8	0.0108444	1.22251E-05
HIST1H2AM	0.04923694	1.22914E-05
NEU3	0.04606657	1.25655E-05
P2RY1	0.04711557	1.25931E-05
DVL2	0.01950291	1.26486E-05
SHPK	0.02676454	1.26521E-05
FBXL18	0.03524596	1.26798E-05
NDUFC2	0.15082716	1.27126E-05
PTX3	-0.2040082	1.27159E-05
RBPJ	-0.0548527	1.27348E-05
MAK16	0.03389234	1.27666E-05
BPNT1	0.02973038	1.27777E-05
VCPKMT	-0.0832298	1.2857E-05
AC012306.2	0.03420436	1.29507E-05
KMT2A	-0.1882047	1.30428E-05
ZNF335	-0.0337957	1.30686E-05
SHLD3	0.03182233	1.31064E-05
BPGM	0.03699151	1.31335E-05
MAFIP	0.04626683	1.31391E-05
EYA3	-0.0742231	1.31427E-05
ZNF501	0.01470151	1.32333E-05
GHITM	0.10006415	1.33745E-05
GIHCG	0.22196142	1.34029E-05
CCDC24	-0.0814328	1.35638E-05
AC022150.4	0.03904393	1.36473E-05
FNBP4	-0.1577243	1.3778E-05
CWF19L1	-0.0128992	1.40482E-05
OSBPL7	0.01594702	1.41173E-05

DHX36	0.0027027	1.4581E-05
GMPPA	0.00781736	1.46463E-05
PRELID2	0.02418456	1.46851E-05
NR1H3	-0.0312162	1.47609E-05
INIP	0.04823261	1.48062E-05
LIG1	-0.015224	1.48761E-05
AC009005.1	0.04853803	1.48842E-05
LINC00174	0.0615596	1.4936E-05
CYP51A1	0.04870695	1.50855E-05
SCN1B	-0.1475694	1.51392E-05
LIMS1	-0.0410633	1.52456E-05
AC009093.2	0.06198776	1.52696E-05
GYS1	0.0680283	1.53477E-05
GXYLT1	0.01154755	1.54449E-05
MICAL1	-0.0853068	1.54571E-05
NIPA1	-0.0499946	1.55252E-05
NLE1	-0.0075115	1.5546E-05
SRXN1	-0.0102333	1.56277E-05
CRNKL1	-0.0304439	1.56279E-05
AL590064.1	0.02750099	1.56288E-05
RTL6	-0.0036658	1.56941E-05
TAB1	0.02064788	1.57752E-05
RPP25	0.03154586	1.57842E-05
BORA	0.0507424	1.58801E-05
STPG1	-0.0631927	1.59621E-05
TMEM120A	0.008915	1.59848E-05
NOP14-AS1	-0.0529844	1.60851E-05
PRAF2	-0.0623505	1.61001E-05
ARFGEF2	-0.0495352	1.62086E-05
GAB1	0.00577047	1.63877E-05
KLHL15	0.03816121	1.6656E-05
AL357079.1	0.06192965	1.67184E-05
HCLS1	0.13243373	1.68795E-05
CSTA	0.14078684	1.70143E-05
PPOX	-0.0172961	1.71648E-05
AC046134.2	0.02685736	1.71928E-05
SPDL1	0.10412411	1.73546E-05
CAPN11	-0.0925509	1.74932E-05
ZFYVE27	0.01927169	1.74964E-05
MAP3K7	0.01807172	1.75112E-05
KLF7	0.02283395	1.7544E-05
RAB38	0.04850858	1.76574E-05
CHI3L1	0.07357357	1.77019E-05
SAMD9L	-0.0265217	1.79123E-05
LRRC61	-0.0199882	1.80807E-05
ARHGAP19	0.00566244	1.80825E-05
TRAPPC5	0.15711069	1.86013E-05
AL391832.2	-0.0018404	1.87325E-05
ZDHHC8	-0.050476	1.89105E-05
EEF1G	-0.184697	1.89401E-05

BCLAF3	-0.0430428	1.89929E-05
TCF7	0.02917591	1.89968E-05
AK5	-0.077286	1.90748E-05
S1PR2	-0.0710283	1.94873E-05
BLOC1S1	-0.0070204	1.95878E-05
ING5	0.044054	1.96102E-05
BDNF-AS	0.05783509	1.96558E-05
ITGA6	0.08872206	1.98098E-05
PNRC1	0.14521095	1.98529E-05
ZNF549	-0.0073265	1.99182E-05
PHETA1	-0.0792334	2.00328E-05
C1QTNF3	0.03897338	2.00994E-05
ZNF30	0.03595072	2.01089E-05
ZKSCAN7	0.03675396	2.02412E-05
PSPC1	0.00847852	2.04047E-05
PIGB	0.04216613	2.05548E-05
CCHCR1	0.03345355	2.05617E-05
PDP2	0.01328397	2.05683E-05
SRGAP2	-0.0138582	2.06952E-05
NEK4	0.07337569	2.08247E-05
CNBD2	0.0090696	2.11476E-05
AC017083.1	0.01204404	2.12856E-05
RAD52	0.05461117	2.13684E-05
SGMS1	-0.0573297	2.13923E-05
H2AFX	-0.0568132	2.16547E-05
PFDN4	-0.0489476	2.16641E-05
UBXN2B	0.05914501	2.19408E-05
ZNF620	0.04935829	2.20004E-05
RBMX	0.14157009	2.20038E-05
TEP1	-0.0475205	2.20594E-05
DNAJC28	0.05223018	2.21973E-05
NEIL2	-0.0170128	2.222E-05
MTHFR	0.03669782	2.25318E-05
ZNF611	0.0146473	2.26207E-05
ARMT1	-0.0109097	2.29536E-05
BTG1	-0.0225556	2.2969E-05
ZNF236	-0.0234285	2.31221E-05
FOXJ2	0.00357543	2.33182E-05
PTAR1	0.02383046	2.3342E-05
ZNF324	-0.0242403	2.34458E-05
SEC61G	-0.0779015	2.38534E-05
VAC14	0.04645476	2.40975E-05
AL391121.1	-0.0603283	2.41426E-05
TP53I3	-0.0713602	2.43236E-05
DNMBP	-0.114956	2.43248E-05
TYW5	0.0217539	2.44167E-05
ACE	-0.1187194	2.47127E-05
NDUFAF5	-0.0135051	2.48875E-05
HPDL	0.03340027	2.49619E-05
C4orf33	0.02360655	2.52057E-05

AC024560.4	0.02926445	2.52241E-05
FASTKD5	0.04523466	2.52788E-05
ATG16L1	-0.0113316	2.5283E-05
DDX5	-0.2736301	2.52875E-05
PRC1	-0.053488	2.54098E-05
TMPRSS9	0.06973501	2.58014E-05
ALKBH8	0.00415442	2.60543E-05
PPP2R1B	-0.0418949	2.62512E-05
ACSL3	-0.0947776	2.6312E-05
MN1	-0.1303296	2.6329E-05
BOLA1	0.02882199	2.64648E-05
CTBS	0.10857462	2.66375E-05
RWDD3	-0.0080155	2.67692E-05
SLC2A11	0.03481805	2.68764E-05
TMEM138	0.02655948	2.69699E-05
UHRF1BP1	0.02547146	2.70206E-05
ZNF696	0.02327306	2.70681E-05
TMEM154	0.02114392	2.707E-05
AC127521.1	0.00168915	2.71814E-05
P2RX7	0.02379683	2.75272E-05
DLC1	0.04972889	2.77073E-05
CCNG1	-0.0115966	2.7741E-05
AC027644.3	-0.0355872	2.77431E-05
AC074032.1	0.05006128	2.77474E-05
EMC10	-0.0084883	2.79889E-05
SLC9A7	-0.038994	2.85082E-05
AL591845.1	0.04217555	2.87061E-05
LEPR	0.05590153	2.91889E-05
AC008957.1	0.02752264	2.94379E-05
SPATA5L1	-0.0067223	2.95497E-05
RAB40B	0.03753993	2.97238E-05
DONSON	-0.0034479	2.97308E-05
TRMT2B	0.0686103	3.03707E-05
KIAA1328	0.013968	3.05532E-05
FLVCR1	0.04480801	3.05729E-05
ZNF747	0.00707985	3.098E-05
AC067852.2	-0.0121526	3.1019E-05
RINT1	0.0174929	3.13503E-05
AC009812.1	0.04632407	3.15555E-05
FARP2	0.02044919	3.15749E-05
CD27-AS1	-0.084526	3.16061E-05
CLYBL	0.07273808	3.16902E-05
RPL10A	-0.1904751	3.19537E-05
AL080317.1	0.04359324	3.19793E-05
WRAP53	0.01874719	3.20231E-05
ZNF527	0.02613522	3.22858E-05
OBSL1	0.01313431	3.23767E-05
PHF13	-0.0882957	3.23786E-05
TUBG1	-0.0423728	3.26102E-05
CTIF	0.03026744	3.27234E-05

PCGF3	-0.0825177	3.28597E-05
SLC9A8	-0.0053995	3.29215E-05
MIOS	-0.0237152	3.29424E-05
HAUS5	-0.0050875	3.29856E-05
DDX17	0.17767578	3.31715E-05
ZFP28	-0.0647456	3.35581E-05
CRTC1	-0.0246805	3.38319E-05
LINC01238	0.03615243	3.40103E-05
CEP68	-0.0134833	3.41309E-05
PEAK1	-0.1229766	3.44795E-05
PYGO2	-0.0206311	3.48599E-05
DNAJC30	-0.0634016	3.51034E-05
KLHL7	-0.0612669	3.52369E-05
C19orf44	0.03018384	3.5474E-05
JMJD7	-0.0312319	3.58618E-05
CDCP1	-0.063668	3.61192E-05
RNF31	-0.003471	3.6198E-05
AC073896.2	0.02084945	3.7032E-05
CPA3	0.25099663	3.70369E-05
PRKAR2B	-0.01116	3.71175E-05
NABP1	-0.152731	3.7454E-05
PINLYP	0.02443566	3.77849E-05
GRB10	-0.0524379	3.78789E-05
PORCN	0.03717719	3.79137E-05
ZNF296	-0.083173	3.83757E-05
TAFA2	-0.0714031	3.85329E-05
MICU3	-0.0294374	3.86741E-05
TYMSOS	0.02520464	3.90941E-05
WDYHV1	0.00914017	3.93843E-05
AC099343.3	-0.0283413	3.93914E-05
BAZ1A	-0.0671572	3.96418E-05
PRKAB1	-0.0760416	3.99879E-05
NETO2	0.03141106	4.01772E-05
CCT6A	0.14610243	4.0191E-05
SSR3	0.10449759	4.02922E-05
AL035587.1	0.01825146	4.03767E-05
RBL1	0.05942506	4.04594E-05
SGTB	-0.0701785	4.04665E-05
AL021155.5	0.00775691	4.06243E-05
AC011939.2	-0.0610649	4.06603E-05
LINC02762	-0.1089761	4.07585E-05
TM7SF2	0.03200307	4.10284E-05
AL157834.1	0.04763003	4.17629E-05
AL109628.2	0.04256595	4.19817E-05
DHCR7	0.04754716	4.22351E-05
ZNF614	0.0105958	4.23139E-05
PLEKHG2	-0.0636762	4.25471E-05
AC006427.2	0.04119572	4.26683E-05
ALMS1	0.01629901	4.34752E-05
TTLL4	-0.0048378	4.35352E-05

DOP1A	0.00993963	4.36905E-05
LBH	0.16454734	4.39117E-05
PPT2	-0.0257734	4.41003E-05
INTS6L	-0.0125418	4.42612E-05
CCDC9	-0.0804672	4.43037E-05
TVP23C	0.02482685	4.44705E-05
MIR29B2CH(	-0.0809222	4.45887E-05
TNNT3	-0.0908761	4.46137E-05
RNPS1	-0.0112188	4.50699E-05
SH3PXD2A	0.07382371	4.54188E-05
LSM11	0.04153652	4.55979E-05
MDGA1	0.02326261	4.59559E-05
TXNRD2	0.00558994	4.61593E-05
DENND6A	-0.0209198	4.62012E-05
AC004076.2	0.02099856	4.6489E-05
RHOBTB2	0.02453711	4.6549E-05
TMEM175	-0.0254975	4.65761E-05
SLC38A6	0.04260542	4.66536E-05
RMDN2	0.03934851	4.68535E-05
KIAA0753	-0.0084523	4.70352E-05
CENPK	0.00559911	4.75155E-05
PSTK	-0.0101134	4.90849E-05
LRRC37B	-0.0561944	4.91239E-05
SPACA9	-0.0903908	4.94985E-05
PURA	0.05872392	4.98298E-05
C19orf81	0.05706447	5.1258E-05
FAM234B	0.02563402	5.16538E-05
AL117336.2	0.04453275	5.18276E-05
GSTZ1	-0.0295563	5.18481E-05
SMYD5	0.0314621	5.19147E-05
ADCK2	-0.0010503	5.19409E-05
EML4	-0.1131334	5.26328E-05
AC021097.1	0.04296112	5.28536E-05
CNEP1R1	-0.0682708	5.3036E-05
OFD1	0.06683487	5.36712E-05
GOLGA4	-0.0304622	5.39117E-05
ELL	-0.1132629	5.48971E-05
TMEM50A	-0.049264	5.54651E-05
NME7	0.05350305	5.65566E-05
ACER3	0.0266731	5.67917E-05
UFSP2	-0.0692645	5.72614E-05
ADGRA2	0.04318888	5.81341E-05
KNTC1	-0.0310513	5.9609E-05
AC139530.1	0.00714635	5.98119E-05
RANBP10	-0.0220756	5.98912E-05
TDRD7	-0.0132789	6.01333E-05
ZNF569	0.01356203	6.05287E-05
HARS2	-0.0281738	6.06533E-05
C12orf43	-0.0322128	6.08287E-05
FANCI	0.01273808	6.08752E-05

LAMTOR1	0.17381993	6.27012E-05
AMZ1	-0.0857386	6.29723E-05
NOL7	-0.1322947	6.38306E-05
HSPA9	0.00462733	6.3854E-05
TTC38	0.00749477	6.55021E-05
KIF11	0.04984356	6.63794E-05
DECR2	0.030942	6.65409E-05
HSD17B8	0.01433742	6.72195E-05
STK39	0.03075849	6.7466E-05
TRMT12	0.02140658	6.82494E-05
TAS2R30	0.01506106	6.86184E-05
MALRD1	0.01016325	6.87184E-05
CLCC1	0.01736125	6.89852E-05
CAHM	0.01945494	6.92573E-05
AC083862.3	-0.0421735	6.93623E-05
SEPTIN2	0.01645382	6.95728E-05
IQSEC1	-0.0967495	6.9837E-05
EZH2	-0.1257901	7.00348E-05
AC009831.3	-0.0426273	7.06916E-05
TBC1D32	0.0448588	7.07163E-05
ZNF207	0.09081134	7.12448E-05
AL035587.2	0.03612667	7.14775E-05
C9orf72	0.00872368	7.16413E-05
ADAMDEC1	0.06555656	7.22583E-05
SEC62	-0.0902219	7.26165E-05
PRICKLE3	0.01195759	7.27768E-05
YIPF4	0.10572105	7.27845E-05
SPATA20	-0.0326814	7.27861E-05
ZNF76	0.03141166	7.29044E-05
MIR1915HG	-0.0002958	7.42058E-05
TRGC2	0.02398376	7.48165E-05
SUFU	0.01014399	7.49484E-05
NDUFV2-AS	0.01263571	7.55331E-05
EXD3	-0.0519355	7.56465E-05
TUT1	0.00526135	7.59461E-05
USP8	0.02166233	7.60539E-05
VPS52	0.01245551	7.67764E-05
SCAPER	0.04138989	7.75577E-05
FAM131A	-0.0984905	7.83833E-05
KCNQ1OT1	0.43780418	7.84921E-05
ZNF225	0.01239201	7.86035E-05
CCDC167	-0.0237728	7.93886E-05
AC010680.3	0.03336398	7.96493E-05
HNRNPD	0.10268508	7.96728E-05
RWDD2B	0.06336918	8.14293E-05
AARS2	0.05899537	8.14501E-05
AC108047.1	0.02223454	8.24208E-05
NHEJ1	0.0424815	8.37292E-05
ZNF713	0.03626453	8.4276E-05
MYO1G	-0.1093133	8.48461E-05

PTGS1	0.08168945	8.53712E-05
RNF121	0.03388017	8.55359E-05
SH3GLB2	-0.0332786	8.56865E-05
AC004034.1	0.01616003	8.62889E-05
SOCS6	-0.0136872	8.70728E-05
TMOD2	0.02841888	8.7113E-05
FKBP15	-0.0219331	8.72922E-05
MEST	0.01285796	8.74434E-05
PDIA5	0.0091452	8.81326E-05
MARK4	-0.0184838	8.81505E-05
ARFRP1	-0.0702357	9.01263E-05
SEC11A	-0.0635331	9.16988E-05
AC107375.1	0.00910511	9.22368E-05
E2F3-IT1	0.009841	9.51452E-05
ACTR3	-0.1254237	9.60901E-05
ZNF678	0.05493024	9.65369E-05
XRCC2	0.04348158	9.66953E-05
BSPRY	-0.1225044	9.67534E-05
AC106707.1	0.02749329	9.70211E-05
INSIG2	-0.0150292	9.77871E-05
TMTC3	0.01193513	9.86868E-05
EFCAB7	0.04598637	9.89683E-05
GNPDA1	0.05660506	9.93376E-05
SLFN12L	0.05769353	9.95064E-05
MORF4L2-A:	0.04800644	0.000101569
C10orf143	0.02142599	0.00010286
AC005076.1	0.05126147	0.00010391
USP30-AS1	-0.0380052	0.000104412
ZSWIM8	0.0278432	0.000104463
MEGF9	-0.0001777	0.000104828
TTLL5	0.02575175	0.000104936
SLC24A1	0.05768349	0.000105159
CCDC71	-0.0254174	0.000107151
THBS1	-0.197301	0.000107796
PRR34-AS1	-0.0451458	0.000108211
URB1-AS1	-0.03572	0.000109165
DEPDC7	0.02395493	0.000111604
ASCC1	-0.053718	0.000113012
FRAT1	0.00838592	0.000113446
UBE2E2	-0.0010453	0.000114537
KPNB1	0.06925361	0.000114721
LINC01806	-0.0634973	0.000115075
SHF	-0.065375	0.000115259
NOP58	0.17414251	0.000115709
PARP12	-0.0161645	0.000116695
SFPQ	0.16631042	0.000116861
SIAE	0.0046739	0.000116987
CYP2R1	0.0170135	0.000117011
LTO1	-0.0580261	0.000117211
ILDR2	0.02481285	0.000118759

FBXO33	-0.0896355	0.000119637
AC023157.3	-0.0586915	0.000119922
ZBTB22	-0.0245302	0.000120427
POGLUT3	0.00211206	0.000120587
POLD1	-0.0101199	0.000121234
PPP1R3B	-0.062929	0.000121407
KCTD13	0.03667385	0.000122586
SESTD1	-0.0684086	0.000123131
DAPK1-IT1	-0.0572922	0.000123356
LINC01002	0.02875425	0.000124356
LRFN4	0.0308318	0.0001252
CAMKK1	-0.0931472	0.000126431
DENND2D	0.03733205	0.00012659
AC005726.5	0.03736608	0.000126798
MYLIP	0.05928699	0.000127424
ZNF700	0.02882843	0.000127633
SYTL4	-0.104053	0.000128483
TMEM254	-0.0063814	0.000128725
CCDC15	0.05958369	0.0001291
AC024075.2	0.04403893	0.000129278
AL450384.2	0.03931341	0.000129328
BTN2A1	-0.0188623	0.000129418
MED22	-0.031901	0.000131206
IKZF1	-0.0773588	0.000131341
HLA-F	-0.0834256	0.000131472
TEPSIN	0.00087191	0.000131694
VENTX	-0.0275791	0.00013193
APOBEC3F	0.01029766	0.000132581
SEC61A2	-0.0839934	0.000132693
SPHK2	0.04044946	0.000133477
AC007952.4	-0.006892	0.000133932
APBA3	0.01979018	0.000134982
ACTA2	-0.036063	0.000136578
LINC01122	0.04043383	0.000137546
NPM2	-0.0461676	0.000138107
AL035071.1	0.01883331	0.000138944
NACC2	-0.0807754	0.000138946
SMARCE1	0.00508654	0.000139095
PHLDB3	0.00710196	0.000139233
CRTC3	-0.0588929	0.000140357
HMGN2	0.07151587	0.00014191
RRN3	0.01569365	0.000142442
ZNF619	0.03447432	0.000142443
RBFADN	0.04679514	0.000143057
LSM8	0.08327885	0.000143205
LINC02193	0.02262664	0.000143578
MT2A	-0.2943068	0.000143937
FBXL8	0.04885201	0.000144333
ZNF143	0.07572661	0.000144406
IPO11	-0.0689489	0.000145327

C10orf88	0.05510813	0.000146749
HIST1H2AL	0.04376994	0.000147634
ACOT9	-0.0345896	0.000147845
DEF8	-0.0790095	0.000147849
LRRC57	-0.0593297	0.000148155
FURIN	-0.0516438	0.000149348
EARS2	0.02595724	0.00015149
TBC1D19	-0.0076805	0.000152336
SH3BGRL	0.18694126	0.000154588
PPIG	-0.141738	0.000155096
ATF6	-0.1237785	0.00015543
PACS2	-0.0099516	0.000156375
WDR47	-0.0591436	0.000157537
AC090517.2	0.03080653	0.000159607
HIRIP3	-0.028585	0.000161409
TSPAN14	-0.0179794	0.000161985
PCNX3	-0.0015485	0.000162572
CCSAP	0.01515389	0.000163194
NAXD	-0.01312	0.000163332
DPH2	0.04572273	0.000164593
AC079922.2	-0.0033315	0.000167271
PJA1	-0.0096587	0.000167987
FDXR	0.02432875	0.000168239
FBXO32	0.04938058	0.000170014
NRAV	-0.0349281	0.000170478
GIN1	0.0581127	0.000170794
ZNF75A	0.02955647	0.000171619
LRWD1	0.02447254	0.000171868
HECTD1	-0.0046988	0.000172674
GABPB1	-0.0897012	0.000172973
LINC01572	0.03105481	0.000173128
AC132872.1	-0.0217714	0.000173882
SLC22A4	-0.0655438	0.00017823
THBS3	-0.0831677	0.000179483
ZHX3	0.02725406	0.000180196
PRR3	0.0288472	0.000180789
CLDN15	0.00379705	0.000185624
FRMD4A	-0.0247733	0.000186233
PFDN5	0.15767725	0.000186932
ATG2B	0.01050842	0.000187462
C9orf85	0.01656854	0.00018836
EOGT	0.02262095	0.000188882
PHIP	-0.0345459	0.000189618
MOV10	-0.0541759	0.000191437
P2RX1	-0.0469328	0.000193134
PANX2	0.02064693	0.000195035
AC026401.3	-0.1006358	0.000196226
DNHD1	0.02771826	0.000196304
AC024560.5	0.01336095	0.000198079
ASB6	-0.0844151	0.000198183

ZNF71	-0.059731	0.000199932
RNASEH2A	0.035068	0.000200331
ARHGAP10	0.06359886	0.000200848
DHX58	0.02714787	0.000201054
GORAB	-0.0193872	0.000201172
PELI1	-0.1161923	0.000201615
TYMP	-0.0042937	0.000202817
CENPBD1	0.02589903	0.000204183
AL359643.3	0.0378091	0.000205954
UBLCP1	0.05640827	0.000207645
FOXO4	0.03610104	0.000209591
RAB27B	-0.1815952	0.000210247
IMPDH2	0.11517465	0.000216199
TIPIN	0.05484532	0.000218762
AC090559.2	-0.0022571	0.000220947
CST3	0.3692186	0.000222472
UBXN2A	-0.0609214	0.000222481
PCF11-AS1	0.01214906	0.000222519
ARMC9	0.05677386	0.000223612
LINC00683	0.02292248	0.000223659
SF1	-0.2275391	0.00022409
RECK	-0.0446152	0.000225148
TMEM94	0.02195102	0.000226599
LINC00653	0.03525602	0.000228476
STEAP3	0.02438617	0.00022899
ABCB8	-0.0613898	0.000231209
NKAPD1	-0.044607	0.000231579
GTPBP8	-0.0292351	0.000232253
RDH13	-0.0449471	0.000232488
MIR646HG	0.01381449	0.000236413
PARP16	0.03987934	0.000239538
DCAF4	0.03526593	0.000240003
ZNF764	-0.0086433	0.000240479
PEX11B	-0.0425961	0.000241372
TRAM2-AS1	0.05897312	0.000242597
AAR2	-0.0276199	0.000243232
ZNF282	-0.0693372	0.000245504
MBOAT1	-0.0566802	0.000247473
POLR3G	0.05710136	0.000247511
NBPF10	0.05071349	0.000248623
AL390195.2	0.03065508	0.000251588
VAT1	-0.0040814	0.000251742
SLC25A51	-0.0239409	0.00025177
ITGAL	-0.1563206	0.000251923
C19orf53	0.03150986	0.000256123
OTUD3	0.01076735	0.000258491
ZNF552	0.01890784	0.000259746
PEMT	0.04540417	0.000261885
ECH1	-0.00743	0.000262069
SAP25	0.01505885	0.000262595

DARS2	0.03517809	0.000262964
BORCS8	-0.03617	0.000266037
IL17RC	-0.0613058	0.000266059
WDR35	0.00717995	0.000267481
IKBKG	-0.0514783	0.000273941
AC008393.1	0.02646534	0.000276403
UBXN1	-0.1051729	0.000277251
NBPF3	0.05220069	0.000280358
IGSF8	-0.0020053	0.000280447
CIP2A	0.03264031	0.000285031
AC083799.1	-0.0380818	0.000285874
AC068888.2	0.02717178	0.000286881
ZRANB3	0.03724925	0.000288607
SMPDL3B	0.01823738	0.000292129
ZNF879	0.03047377	0.000292855
EMC1-AS1	0.03140802	0.000294188
SGSM2	-0.0806914	0.000294667
SPSB1	-0.0457113	0.000295108
ZBTB2	-0.0749009	0.00029653
ZNF699	-0.0418102	0.000297099
TRIM71	-0.029817	0.000299341
AL353194.1	-0.014389	0.000301416
RPS13	0.11042327	0.000301708
NCDN	0.03791495	0.000302356
AC069544.1	0.01695232	0.000303597
HMGCS1	-0.1726952	0.000303657
AL162253.2	0.0347824	0.000306035
RTP4	0.01112813	0.000311713
AL133410.1	0.02028979	0.000311852
LIN7B	0.04608101	0.00031334
WDR76	-0.0245572	0.000313629
FAM110A	-0.1012465	0.000314198
BEND5	0.00035714	0.000314634
PRSS57	0.15106042	0.000316726
MSH5	0.00914798	0.00032118
TTC7B	0.05350289	0.000326607
NAIF1	-0.0067644	0.000327603
TIRAP	0.0112142	0.000330415
HDGF	0.11192936	0.000333006
AP002495.1	0.02455789	0.000333605
EGFL8	0.00845104	0.000336095
KCTD11	0.00979477	0.000337794
WASF1	0.06467688	0.000341083
EID2B	0.00357894	0.000343902
VIPAS39	-0.0070565	0.000345433
BET1L	-0.0182912	0.000347902
AMBRA1	-0.0808819	0.000349895
OGFRL1	0.03640314	0.000350554
ZNF35	0.00193207	0.000356469
TTC9	-0.0681322	0.000357213

PIGW	0.01482609	0.000359776
FANCG	-0.0036792	0.000360955
LY6G5C	0.01931212	0.000362495
COA6-AS1	-0.0154505	0.000364363
AC103703.1	0.01576273	0.000367977
PARP2	0.03413389	0.00036828
IL12A	-0.0166987	0.00036853
HBEGF	0.04379215	0.000373489
APOL3	-0.020656	0.000374976
HMGN5	0.08392629	0.000377424
GSPT1	0.12332899	0.00038148
TRIM41	-0.0249912	0.000383013
AMER1	0.03486203	0.000383918
DNAJC27	-0.0468094	0.000387991
MICAL2	-0.0860444	0.000390153
PPAT	0.03119354	0.000391001
ZNF776	0.01671828	0.000391721
AC069243.1	0.02456462	0.000392654
DLEU2	0.0073459	0.000393359
HIP1R	-0.004197	0.000397929
UCN	0.02566138	0.00039874
AC040977.1	0.03209508	0.000401888
AC006059.1	0.05314588	0.000406687
MTMR1	-0.029268	0.000408089
CCDC122	0.06141725	0.00041232
TMEM106C	-0.0505489	0.000413049
INO80C	0.03864719	0.00041347
C18orf54	0.05949887	0.000413626
PPP2R3B	0.02201192	0.000417784
BEX3	-0.0714655	0.000419474
HSD17B1	0.02394695	0.000421699
C20orf197	0.02311554	0.000422528
HIST1H2BN	0.00667574	0.000423075
C4orf3	0.07454876	0.00042627
SLC25A42	0.01713961	0.000426989
CPNE1	-0.0006625	0.000430993
SMURF1	-0.104509	0.00043215
PRDX2	-0.0210682	0.000435288
SEL1L3	-0.051999	0.000438424
ESD	0.12775981	0.000441515
AFF1	-0.0877619	0.00044161
ZNF570	-0.0322394	0.000442358
RNF185	-0.0121707	0.000446897
CROCC	0.0292153	0.000447275
WDCP	0.03570885	0.000449819
ASTE1	-0.0539328	0.000453192
ZNF555	0.02353935	0.000457948
MROH7	0.0309863	0.000458338
ATP6V0B	0.10686241	0.000460234
SLCO4A1	-0.0491318	0.00046537

UBOX5	-0.0281714	0.000466996
AC016394.1	0.02965056	0.00046864
AC064805.2	-0.0138213	0.000472086
SLC35B2	-0.0067015	0.000473272
GKAP1	0.03618086	0.000473274
AC009133.1	0.03058562	0.000473987
AC004847.1	0.01645924	0.000474372
TCEAL8	0.05014587	0.000479208
PRAM1	-0.082771	0.000487453
HIST1H2AD	0.02702035	0.000488449
RPL32	0.08889489	0.000488697
CASTOR3	0.01875322	0.000494234
FAM118B	-0.0079503	0.000510052
TRAM2	0.01312645	0.000511815
TCEA1	0.06355136	0.000512608
ZBTB47	0.02660013	0.000514439
KIAA0355	0.02603551	0.00051883
DNAJC1	-0.1768092	0.000523358
BBS9	0.01304843	0.000538399
ZSCAN5A	0.02703201	0.000553859
LINC01943	-0.0125167	0.000554838
SWAP70	0.06589327	0.00055713
GEN1	0.05618052	0.000557446
KMT5C	-0.0660398	0.000566433
ZRANB2-AS1	0.04747424	0.000576523
ZNF599	0.02631531	0.000577205
HIST1H2BF	-0.0053183	0.000578535
HOXA10-AS	0.09522362	0.000583202
BARD1	0.01273147	0.000586784
MORC2-AS1	0.04251745	0.000587404
INCENP	0.02789406	0.000588349
TWSG1	-0.0613463	0.000590728
MSMO1	-0.046179	0.000592039
DMXL2	-0.0186063	0.000592091
AL392172.1	0.0353042	0.000593798
SP4	-0.0021782	0.00060167
GAS8	-0.0054635	0.000602887
AKT1S1	-0.0469412	0.000608362
TFR2	0.02644199	0.000609336
AC016065.1	-0.002405	0.000617999
RAB29	-0.0659573	0.000618342
DTNB	0.05726243	0.000630238
POFUT2	-0.0950619	0.000631693
RIC1	0.02029353	0.000631939
HIVEP3	-0.0243646	0.000635393
TNFRSF10B	-0.0667374	0.000637432
NME8	0.02052884	0.000643439
TMEM104	-0.102255	0.000643648
HMGCR	-0.1134098	0.000648182
AP003307.1	-0.0127973	0.000648469

ZNF467	-0.0849876	0.000649804
ZNF827	-0.0068476	0.000649804
BEST1	0.026321	0.000651032
TMEM201	0.01924319	0.000654213
POLD3	-0.0171515	0.000660918
PYGB	0.02159022	0.000666021
PUDP	0.0107006	0.000672165
RTF1	-0.0874852	0.000675132
LINC00894	0.0569026	0.00067519
CUL7	0.00914114	0.00067927
TSPAN4	-0.043305	0.00068182
TMEM53	-0.0275649	0.000693542
ZNF658	0.03604217	0.000695258
ZNF154	0.02707087	0.000699828
MEIS1	-0.1281705	0.000700224
PIK3R5	-0.0669504	0.000705963
E2F6	0.01228209	0.000709098
PADI4	-0.0306424	0.000709737
MOB1A	0.00700049	0.000710292
CSDE1	-0.140413	0.000710456
SYNJ1	-0.0403429	0.000712093
PIGV	0.02293544	0.000713012
ACSS2	-0.0109093	0.000728962
ZMAT1	-0.0135985	0.000736332
AC011462.5	-0.0495756	0.000738764
EXD2	0.02609859	0.000739985
COG7	-0.0298433	0.000743452
TTLL10	-0.0799827	0.000758254
SPATA2	-0.0286808	0.00076423
NHSL2	-0.0012978	0.000772316
USB1	0.02583035	0.000786831
MAML2	-0.0717862	0.000788242
AC007878.1	0.01235926	0.000790544
SNX8	-0.0680675	0.000794495
AC068205.2	0.0339057	0.00079726
KCNK5	-0.0504519	0.000800433
DUS2	0.00294001	0.000800952
ZNF484	0.01310205	0.000804993
NIPBL	0.04212565	0.000806593
ARHGAP5	-0.024789	0.000841849
UCK1	-0.0258769	0.000844972
AC007066.2	0.04086205	0.00085089
LYRM9	0.03411173	0.000855551
LAPTM4B	0.07356878	0.000858304
C8orf37	0.05288768	0.000871603
TSEN54	0.00202086	0.000876931
ATP5MC2	0.13949439	0.00088275
MICALL2	-0.0298346	0.000887659
WDR24	0.02089039	0.000889806
CPPED1	-0.0576609	0.000898462

AL096803.2	0.01470673	0.00090405
AP4E1	0.01948226	0.000914201
C14orf93	0.00956638	0.000914632
AKAP8	-0.0265793	0.000921989
PPM1D	0.02034729	0.000950716
TRMT61A	0.03940345	0.000951382
DCLRE1A	0.03468519	0.000953088
PARD6B	0.02241327	0.000964903
AC092944.1	0.05022794	0.000974056
AL121603.2	-0.0077371	0.000988069
SFXN1	0.0654732	0.001006283
SLX4	-0.0651198	0.001012901
SLC39A13	-0.0335489	0.00101467
AC097382.3	-0.0021876	0.001016836
CRYBG1	-0.1118554	0.001020111
ZXDB	-0.0055153	0.001020282
EMSY	0.04612203	0.001026444
CCDC92	-0.0114931	0.0010335
SLC27A2	0.03553487	0.001038374
ZNF583	-0.0100233	0.001065971
FAM47E	-0.0777765	0.001079129
CHKB	-0.0372213	0.001084196
SMIM6	-0.0154708	0.001085202
AC025181.2	-0.0501296	0.001093363
ARNTL2	-0.0035265	0.001110434
ZNF839	-0.0081779	0.00111088
ZNF148	-0.0223788	0.001126548
PRPF40B	0.04784647	0.001133559
CELF6	0.02164963	0.001142263
TRIM16L	0.03018765	0.001142516
KRTCAP2	-0.0766992	0.001173202
ESS2	-0.0738492	0.001189149
PCM1	0.07826577	0.001200844
C3AR1	-0.0396302	0.001214393
SLC49A4	0.00668597	0.001217575
ZNF280B	0.04926914	0.001232078
FAM126B	-0.0941725	0.00123999
ZNF749	-0.0008753	0.001241845
ZNF554	0.02928689	0.001249203
ZNF175	0.03183137	0.001251146
AL355336.1	0.02736535	0.001252508
CDC42-IT1	0.01845892	0.001256994
MFSD9	0.00289838	0.001260139
AP000873.2	0.01212235	0.001269381
DVL1	0.02574636	0.001273892
ZNF461	0.0095048	0.001279623
DYRK2	-0.0343081	0.00128191
HOXB3	0.01642948	0.001298226
REST	-0.0052735	0.001321436
ARRDC1-AS	-0.0186158	0.001325161

MAPK8IP3	-0.0651676	0.001356263
RPL4	-0.23642	0.001360694
MARCKS	-0.1758711	0.001360994
CDYL2	-0.0295735	0.001374121
ANKMY1	0.03753738	0.001381255
TPI1	-0.0088981	0.001389105
ZNF624	-0.0079462	0.001406315
PROM1	0.16848861	0.001410351
SEC22A	-0.0321831	0.001414722
SH2B1	0.00012814	0.001420295
SRRM2	0.0984454	0.001430729
AC026124.2	-0.0245134	0.001437699
MYH9	0.06057897	0.001438571
USP5	0.04806352	0.0014515
RHOBTB1	-0.0281825	0.001452231
OLIG1	-0.0145298	0.001453856
AL592295.5	0.01687288	0.001462519
YTHDC2	-0.025024	0.001490531
ELAC1	0.02045221	0.001492415
MYO5C	-0.0835179	0.001494086
POLA1	0.0546698	0.001499568
TNRC6C	0.04129974	0.001504964
SRSF2	-0.1201134	0.001506435
CCDC146	0.0268424	0.001513353
AC010969.2	0.00622493	0.001520487
CDCA7	-0.0195431	0.001527869
ACOT4	0.02365288	0.001554883
ZKSCAN2	0.00661905	0.001588225
ETV5	-0.0536361	0.001596858
CD48	-0.0720253	0.001620837
ATP8B3	0.04109207	0.001620892
TMEM217	-0.0996439	0.001629415
POLA2	0.0053668	0.001645357
PYCARD-AS	-0.0490174	0.00165739
YWHAE	0.15152164	0.001657721
PLEKHG4	0.03507252	0.00166423
OTUD1	0.0178588	0.001666627
WDR4	0.01129534	0.001675445
UXT	0.11627867	0.001677267
DSN1	0.00525508	0.00171557
SLFN13	0.02442046	0.00172321
CPB2-AS1	0.02984699	0.00173434
PRKAB2	-0.0245576	0.001742611
DIABLO	0.05142978	0.001763785
WDR31	0.02782607	0.0017677
TCP11L1	0.01518935	0.001774839
DOLK	0.00743975	0.001796155
AC048341.1	-0.0025186	0.001813654
RBMS2	-0.0330713	0.001814409
AC009113.1	0.01556753	0.001820707

ARL13B	-0.0548572	0.001835719
HBD	-0.039624	0.001839991
EIF4G2	0.04571099	0.001860429
GRIK5	-0.0480893	0.001874799
PNRC2	-0.0795785	0.001898729
DGAT1	0.03427085	0.001906466
APPL1	-0.0600972	0.00191836
RNF139-AS1	-0.0095063	0.00192633
AC141424.1	0.01251087	0.001952216
LINC00513	-0.0748232	0.001962705
C1orf54	-0.0469781	0.0019746
FBXL13	0.02317527	0.001976861
SSB	0.15334747	0.001995076
TIGD7	0.03688153	0.002042964
ZNF440	0.0384904	0.002044771
SCAF11	0.0986403	0.002045972
MINDY1	-0.0965802	0.002047075
SEPTIN7	-0.057004	0.002075895
IPO13	0.02126086	0.002085177
LETM2	-0.0996351	0.002089664
FAU	-0.1163487	0.002090792
ITPKC	-0.053247	0.002105404
PGM3	0.03972794	0.002108164
AC105339.6	0.03341272	0.002121759
CHST7	-0.0702726	0.002140703
ZFP1	-0.0501264	0.002177168
ZNF837	0.0044375	0.002195392
C8orf82	0.03270171	0.002204485
ASL	-0.0492142	0.002205387
LINC01515	0.0423881	0.002209078
LINC00528	0.00726669	0.002261598
RAPGEF2	-0.0844913	0.002271523
RTL8A	-0.0040113	0.002294994
BBS1	-0.0066641	0.002314027
C2orf92	-0.0448116	0.002318843
METTL2A	0.03010688	0.002358714
CBR3-AS1	0.06610385	0.002362059
RAB11FIP4	-0.0852619	0.002372302
AL035446.1	-0.1249753	0.002390321
TAPT1-AS1	0.01579523	0.002394017
GK	0.00512501	0.002397216
NDUFC1	0.08009359	0.002401506
NAPB	-0.0357698	0.002418522
LMF1	0.04346925	0.0024338
COMT	0.08522666	0.002453569
SRP14	-0.1717555	0.00249213
ZWILCH	0.01815826	0.002492242
UGCG	0.01892983	0.002508007
ABAT	0.01970169	0.00253414
EXOC3-AS1	0.03572231	0.00253876

HSF2	-0.0458885	0.002539374
TBCEL	0.02241398	0.002627597
SPINDOC	-0.0132901	0.002630654
IL11RA	0.04240216	0.002663004
C9orf40	0.02538806	0.002691971
AP4M1	-0.0134218	0.00273718
ZSCAN25	0.03561948	0.00275835
F2R	-0.1441728	0.002901397
SH3GLB1	-0.1378078	0.002940106
AL121652.1	0.02762609	0.002949852
AVEN	0.05801328	0.002999553
GRAP	-0.0576301	0.00301908
RASA1	0.012969	0.003035638
TAX1BP1	0.09395597	0.003042217
AIF1L	-0.1430909	0.003043672
AL078587.2	0.02531725	0.003045804
SLC25A22	0.01741374	0.003126556
HSPA8	0.20190829	0.003150231
CAMK1D	-0.1318798	0.00316407
MPZL3	-0.0738752	0.003183084
LSS	-0.0081673	0.003191396
ZSCAN2	-0.0057655	0.003209765
AC006449.6	0.01074265	0.003231629
AC021016.2	0.02525143	0.003235345
VPS35	-0.0662372	0.003254435
TPCN2	-0.0285829	0.003304034
PRR5	-0.0544992	0.003307455
STAR	0.02191146	0.003315039
SMCR5	-0.1054124	0.003340092
TARBP2	0.03087121	0.003341173
PRDM4	-0.017146	0.003393128
EPS8L2	0.02040547	0.003397583
SRGAP2C	0.03574021	0.003399293
KNSTRN	0.01823133	0.003402259
USP40	0.0418311	0.003430547
TSTD2	0.00859863	0.00344988
FAM126A	0.02096125	0.003470935
CPSF6	0.01703939	0.003475918
ZNF777	-0.0137791	0.003489531
CCDC186	-0.0935073	0.003508831
ZNF81	0.02012938	0.00353671
AL080250.1	0.04678131	0.003538249
ZNF213	-0.009565	0.003544647
MIF	-0.2357454	0.00355045
GCHFR	0.00087157	0.003554549
AC004241.1	-0.0407996	0.003584705
TRAF2	-0.0441754	0.003610922
AC016949.1	-0.0364575	0.003687361
MTBP	0.02969354	0.003687415
SERPINF2	0.02181973	0.003767108

TM9SF3	-0.0031023	0.003796059
CDC7	0.02443446	0.003898675
ZNF18	-0.0080937	0.003947868
FKBPL	-0.0261352	0.003951287
ZNF626	0.04129125	0.003997827
ZNF319	-0.0219129	0.004189068
LINC00630	0.00606807	0.0042675
SYPL1	0.09285314	0.004275396
EMC9	-0.0027949	0.004287523
RRAS	-0.0640548	0.004397662
LINC01176	0.03915595	0.004416928
AC127035.1	0.03053033	0.004423229
SH3BP5-AS1	0.02659316	0.004499235
MED26	-0.0367825	0.004501195
MCM8	0.03041338	0.004515713
HSD11B1L	0.02731161	0.004529834
HSP90AA1	-0.2574514	0.004567346
TMEM25	-0.0483158	0.004639351
TRAPPC1	-0.0018528	0.004669477
JMJD4	-0.021173	0.004674368
CLSPN	-0.1176304	0.004686015
IPO4	0.0036818	0.004739871
FAM185A	0.01255373	0.004809564
DLG4	-0.0511559	0.004810882
ZNF846	0.03197696	0.004886297
PRMT6	0.00904266	0.00489382
NUMBL	-0.059583	0.00493008
KIAA0513	0.00810462	0.004964401
RPAP1	0.05778424	0.004979374
DHRS13	0.01499395	0.004998416
TIAM2	0.03836306	0.005022973
PTPA	-0.0271466	0.00502761
LINC01473	0.0310156	0.005102751
TBL1X	-0.0046932	0.005130804
AL135999.1	0.00379239	0.005244392
NXF1	-0.0009839	0.005270509
ZNF670	0.0349749	0.005302366
MPP7	-0.0895093	0.005355419
TRG-AS1	-0.2271891	0.00536216
SRSF8	0.04371551	0.005393588
OXCT1	-0.0881637	0.005459226
SLC4A5	0.02345734	0.005461868
ZNF77	0.01702358	0.005462344
ZCCHC14	0.00259592	0.005468847
HOXA9	0.22001917	0.005505731
APOO	-0.0026793	0.005672394
RHBDF1	-0.0844334	0.005821145
CXCL2	-0.0540547	0.005866122
BTN2A2	0.00394889	0.005884616
IL10RB-DT	0.02137004	0.005918541

MBNL1-AS1	-0.007362	0.005953892
C8orf58	-0.0507505	0.005978849
BST2	-0.1279967	0.006079571
CBX2	0.01456732	0.0061417
HRH2	0.008087	0.006315804
ZNF470	0.02136854	0.006316526
AC253572.2	-0.0717165	0.006460074
CA13	0.00148313	0.006519078
SPRYD3	-0.0416349	0.006545734
FBXW9	-0.0179812	0.006613605
ZBTB11-AS1	-0.0122125	0.006670193
SBF2	-0.0093609	0.006696778
RTN4IP1	0.01242376	0.006957179
PIGA	-0.0218581	0.006998062
RMND5B	-0.0233288	0.00701266
GSTM5	-0.007224	0.007146528
NENF	-0.0035611	0.007235741
MYCBPAP	0.02480564	0.007319336
CRYBG3	0.05171818	0.00737926
CTTNBP2NL	-0.0739261	0.00747256
KANSL1-AS1	0.02098071	0.007478763
ARIH2OS	0.0210503	0.007482622
NANOS3	-0.0414586	0.007491528
TOM1L1	-0.0147439	0.007514002
ZBED3-AS1	0.0389477	0.007536994
INTU	0.0489349	0.007758659
HDAC4-AS1	-0.0169305	0.00776342
ZNF275	-0.059648	0.007764193
ATP5F1A	0.135779	0.00783028
ZNF674	0.02129189	0.007845997
MFAP3	0.01000583	0.007910078
ACBD4	0.01975195	0.007994082
LCP1	-0.1535172	0.008118456
SLC7A5	-0.1257197	0.00813786
TBCD	0.01086057	0.008155415
MCU	0.02785376	0.008175552
S100Z	0.0189372	0.008258125
NLRP3	-0.0524305	0.008272693
MIR3936HG	0.02072362	0.008294779
SLC31A1	-0.0342005	0.008354966
AREL1	-0.0329583	0.008405608
EDF1	-0.0826832	0.008440224
BAHD1	-0.046642	0.008495458
EIF4B	-0.0146374	0.008526492
CHN2	0.01190993	0.008565704
SUSD3	0.01758454	0.008595764
AC008074.2	0.00228593	0.008613359
ZDHHC16	0.0122332	0.008678162
AP1S3	-0.0584745	0.00880016
SARS2	-0.0111284	0.008805201

SPAG16	0.02569227	0.008918503
ANXA7	-0.0279751	0.009234022
BRD3OS	0.00443556	0.009248219
ZBTB18	-0.0084056	0.009312181
SBNO1-AS1	-0.0302236	0.009378942
HHLA3	0.02115707	0.009408788
CDAN1	0.0156358	0.009527888
IGFBP6	-0.1010554	0.009792351
AL355001.2	-0.0015892	0.009826219
LAMTOR4	0.12808484	0.009854233
ZNF34	0.00576378	0.009973729
VWA1	-0.0861065	0.009986832
FBXO21	-0.0717065	0.010280529
PIDD1	-0.0406063	0.010398932
FXR1	0.07368216	0.010511571
BRPF3	0.02432424	0.010552879
TRIM52-AS1	-0.0230254	0.010668027
FNBP1	0.01688731	0.010733003
SSR2	-0.0710281	0.010857934
SNHG11	-0.006078	0.010945679
ZW10	0.00804463	0.011127373
DNAJC17	-0.0141855	0.011171941
FIZ1	-0.0092736	0.011178748
TBC1D10A	-0.0652044	0.011254095
SULT1C4	0.01064257	0.011391517
PIGO	-0.0015631	0.011476963
RECQL5	0.04539839	0.011486271
C3orf35	0.02698063	0.01149579
AC147651.4	-0.0565487	0.011554162
GNAS	-0.1736728	0.011606802
FMO5	0.01792636	0.011782723
QSOX2	-1.843E-05	0.01178564
ITGB7	-0.0948526	0.011820645
LINC02021	0.0300103	0.011830253
SERF1A	0.02665486	0.011891449
GINS2	-0.0739869	0.011956483
NKIRAS1	0.0163284	0.012016319
SLC25A44	-0.03603	0.012161253
CCDC191	-0.0083817	0.012221459
CTSG	0.08806393	0.012305673
MOB3C	-0.0395402	0.012345603
SYNCRIP	0.01781491	0.012436991
AL365272.1	-0.0120002	0.012451663
HIST1H2BK	0.02730957	0.012495724
CIDEB	-0.0120975	0.012501964
NAA80	0.04478577	0.012634405
AC007390.2	0.0334506	0.012637888
SEPSECS	0.042118	0.013034385
WDHD1	0.03688954	0.013141324
CACTIN	0.00536328	0.01332588

MT1F	-0.0562164	0.013491527
AL442125.1	-0.0223504	0.013511376
IQCK	0.00272986	0.013551397
AP4S1	-0.0625084	0.01393197
AC018647.2	0.02998644	0.014053955
NDUFB11	-0.0380862	0.014178678
SC5D	0.00453185	0.014446627
ZNF25	0.04105219	0.014454071
LANCL2	-0.0515777	0.014497064
IFT140	0.03364501	0.014538111
EXO5	-0.0071753	0.014654721
FAM204A	0.02878146	0.014963344
HSPA1B	-0.052891	0.015095865
HEATR6	0.01409496	0.015156895
PGLS	-0.1090405	0.015232524
OAS3	-0.0540395	0.01524413
CAMSAP1	0.02737997	0.015416448
AEN	-0.0138271	0.015422295
WSB2	-0.008316	0.015538181
TRBVB	-0.0440659	0.015632148
ANP32A	0.06836807	0.015719913
MMAA	0.01243764	0.015756833
RPS4X	-0.1341316	0.015807061
MAP3K14	-0.064841	0.015990711
TMCO4	0.03011112	0.016031241
CRELD1	0.00381802	0.016046232
OR2L2	0.031656	0.016272984
VDAC2	0.11086491	0.016438508
GOLGB1	-0.0133064	0.01653688
EML2-AS1	0.02967791	0.016595091
SLC25A35	0.02099346	0.016784379
BNIP3	0.04732907	0.017101122
C7orf25	-0.000356	0.017409294
SLC5A10	-0.0388383	0.017630024
NUDT8	-0.0357533	0.017630771
RAB9B	0.03971817	0.01779063
ZFYVE16	0.01997568	0.017856376
ZNF17	0.00902087	0.017934413
NAPA-AS1	-0.0246937	0.018218466
AL033543.1	-0.0761991	0.018252697
KAT2B	-0.0229773	0.018269776
NR2C2AP	0.02667578	0.018578611
ARHGAP12	0.00804499	0.019020308
PNN	0.15411016	0.019156801
AL662844.4	-0.0050106	0.019276587
CSTF2	-0.0007017	0.019308995
ZFP30	0.01307821	0.0200634
AC008035.1	0.03179864	0.020066059
DEPDC1B	0.02673548	0.020333244
POGLUT2	0.00305545	0.020361497

MED20	-0.0137864	0.020664335
ZCCHC4	0.01537348	0.020779577
YJEFN3	0.0058537	0.020902938
SPCS1	-0.0959848	0.021181684
ST3GAL5	-0.0979528	0.021486448
USP30	-0.0449782	0.021588741
TMEM263	-0.0106009	0.021918387
HOXB5	-0.079757	0.022001285
ZNF772	0.01194541	0.022389149
SLC27A3	-0.0062597	0.022405425
C1orf112	0.02348247	0.022551597
MX1	0.03003088	0.022647043
SPATA7	0.01304155	0.022755122
AL645728.1	0.02578247	0.02299933
BUB1B	0.00150419	0.023112033
AC133550.3	0.0315816	0.023276151
SORBS3	-0.0807792	0.023497496
SARM1	0.03620497	0.023984263
LRRC45	0.00518315	0.023988408
B2M	-0.1679842	0.023998138
MPO	-0.2437788	0.024332776
HHEX	0.02267112	0.024610811
KIF3B	-0.036573	0.024612938
ZNF674-AS1	-0.0032787	0.024635857
NAT1	0.01232569	0.024751339
NKTR	-0.0659016	0.024791935
ARRDC3-AS	0.03537632	0.024800547
CHEK1	-0.0742154	0.024865255
ZBTB45	-0.0480315	0.025158691
OCRL	-0.0087725	0.025873474
STARD8	-0.0681304	0.025974822
BTRC	0.01049221	0.026085351
MMP25-AS1	0.01098348	0.026194612
PSD4	0.03249356	0.026893978
MLH1	-0.0319239	0.027460714
A2M-AS1	0.01631822	0.027485878
ALG1L2	0.03863045	0.027507817
CALML4	0.01051224	0.027535953
FNIP2	-0.0838829	0.027806838
TBC1D22B	-0.0244288	0.028026844
AC020910.5	0.02657998	0.028162826
C2CD2L	-0.0587834	0.028240333
BPHL	0.01484375	0.028530891
HIST1H2BG	-0.1003024	0.028651536
TET2	-0.0250396	0.029472055
MRPL53	0.03852919	0.029602231
ZNF726	-0.021155	0.029607516
LXN	0.15536834	0.030077596
TTYH2	0.00881263	0.030991641
AGAP3	-0.0675406	0.031035011

SH3BP4	-0.0378071	0.03127147
ARHGEF17	-0.0650426	0.031430788
TMEM62	-0.0014998	0.031513196
TIPARP-AS1	0.01538071	0.032151206
GRB2	0.08713825	0.033127794
RUBCN	0.01328363	0.033164372
AP3M2	-0.0165473	0.03415351
HNRNPC	0.10052574	0.03457462
MYCNOS	0.02048308	0.034911293
ARFGAP1	-0.047005	0.035293506
HVCN1	-0.0320783	0.035344816
ITM2A	-0.1360218	0.035927757
REPS2	0.0178286	0.036304165
C12orf49	0.01798012	0.037224688
ERN1	-0.0305263	0.037388125
AC020656.1	0.10986849	0.037702807
ZNF235	0.02687206	0.037861778
ENPP4	0.00571653	0.037880978
ALG12	0.02070369	0.038180984
KIF13B	-0.0132642	0.038349046
ZNF200	0.01504945	0.03868007
ZFP36L2	0.16494797	0.038923594
SLC12A9	-0.0739913	0.039977124
ATP5PO	-0.0103913	0.040038323
APEX2	-0.0039455	0.040165296
AC093525.6	-0.0244359	0.040227142
SOCS2-AS1	-0.007245	0.040455781
SNRPF	0.10788319	0.04054818
AP001816.1	-0.0656605	0.0406414
RMI2	-0.0464718	0.041390325
FYN	-0.0526151	0.041520473
HNRNPU	-0.2458393	0.042860967
ELN	-0.1092611	0.043100399
ATP5IF1	-0.05896	0.043157747
ZNF778	0.0242491	0.043494466
JADE3	0.00817239	0.04352883
ASAP2	-0.0033179	0.043617892
DOHH	-0.0319325	0.044866618
AC090517.5	0.02473339	0.046083973
GBP5	0.02210355	0.046180517
PPP1R12C	-0.0213379	0.04627666
COX6A1	-0.1603081	0.046405938
GSTM3	0.03055974	0.046490796
DNA2	0.02327188	0.046528184
TBC1D25	-0.0298402	0.046684665
TBC1D14	-0.001118	0.047103316
NEK8	-0.0220102	0.04772889
POLR3F	-0.036812	0.047784112
DPCD	-0.0523077	0.049570944
TRIT1	-0.0237352	0.049637763

FCHSD1	-0.0289263	0.051257043
VNN1	-0.0959303	0.051774151
C11orf24	-0.081132	0.051884472
LINC01137	0.00851053	0.051974422
ARMCX1	-0.0760443	0.053132814
AP001059.2	-0.0338058	0.053324238
NDUFS5	-0.0645934	0.053359706
ZBTB3	-0.000399	0.053669161
KDELR1	0.05820785	0.053682981
INAFM1	-0.037751	0.053728583
NKD1	0.05490397	0.053923264
TCTA	-0.0545153	0.054146148
PDZD11	0.00627322	0.054454307
CCSER1	0.05763423	0.055151127
KHDRBS1	-0.106943	0.055335615
IQSEC3	0.02306226	0.055436598
MIGA2	-0.0019739	0.056449257
ATPAF1	-0.0070079	0.056904068
HIST1H1D	0.2760215	0.057207659
MIR1-1HG-A	0.00498058	0.057948699
CDPF1	0.00268763	0.058758068
AP001505.1	0.03439951	0.059100759
AC109460.2	0.01993581	0.060889798
INTS9	0.04014145	0.062132212
AC007114.1	0.04531198	0.062242452
DUSP28	0.00418836	0.063843078
SLC25A53	-0.0105931	0.064286731
HYLS1	0.06028937	0.064622082
CTC1	-0.0336197	0.064781569
PSMC3IP	-0.0788768	0.06521745
AC020658.5	-0.0018561	0.065544608
RPL13	-0.1269127	0.066239443
LZTR1	-0.0015547	0.070628805
CLP1	-0.0082812	0.071063686
SRR	0.02117498	0.071669219
CYSLTR2	-0.1239695	0.07182238
AL022162.1	0.02061086	0.072066443
FAM189B	-0.0063696	0.072205625
LINC00173	-0.0300898	0.072214698
SP2	-0.0472054	0.072511693
SBF2-AS1	0.02870246	0.072868295
ATP6V1E2	0.03156591	0.072877729
PRKAR1B	0.03826647	0.073006538
AP5S1	-0.0325114	0.073193486
C17orf75	-0.0066306	0.073318377
FAM107B	0.12330073	0.073536035
TMEM241	0.02627962	0.07373876
PSKH1	-0.0516264	0.074685171
FBXO8	-0.0350406	0.076010598
AC005332.3	0.03029175	0.076671906

LAMTOR5	-0.0821261	0.076920788
SLC25A14	-0.0327102	0.077640664
ATP5MC3	-0.1513589	0.078030458
ATP5MD	0.0771542	0.078047251
BRCA2	-0.0829334	0.078504316
CNPY4	0.01166875	0.079626612
AL136038.5	0.03465854	0.079968769
ZBTB7B	0.01084273	0.080130198
S100A13	-0.0715318	0.08030092
TMBIM6	-0.0290024	0.082497595
GPR137	-0.032378	0.082597419
MFSD13A	0.0278412	0.082953139
RAB24	0.01084985	0.083411812
KLC2	-0.0536976	0.085604745
RALBP1	-0.0800079	0.087426743
ZNF792	-0.00154	0.087978172
AC092428.1	-0.0553886	0.088179951
PHF3	-0.0092392	0.088816924
NAA16	0.00723684	0.089502355
SLA	0.04845824	0.090388543
CD200	0.00326192	0.091427093
TRIP6	-0.0281534	0.09148792
OSBPL11	-0.0673773	0.092259767
DAPK3	-0.0238374	0.092761767
MMD	0.01748736	0.092989909
YPEL1	0.03418589	0.093981769
USP51	0.0454644	0.093998408
AIFM2	0.01112919	0.094002121
RPS3	-0.1505244	0.094303891
CNNM4	-0.0107622	0.095424259
MRPL18	-0.035601	0.097761684
FLT3LG	-0.0242554	0.099738963
TNRC6C-AS	-0.0036455	0.099878028
NCAPH2	-0.0282373	0.10013407
GALE	-0.0544861	0.100204565
UBTD1	-0.0617182	0.100919898
MEAK7	-0.0247322	0.101340174
ATG9A	-0.0514241	0.10161207
RPS23	0.05748097	0.102912438
LINC00294	-0.0085533	0.103690367
MLKL	-0.0766463	0.10408055
TNFRSF10A	-0.0695615	0.104411266
LTK	-0.07655	0.10518796
SRGAP2B	0.00953187	0.10540032
Z95331.1	-0.039475	0.10644855
VAMP1	-0.0545983	0.106881357
IFT46	-0.0054632	0.108072715
SULF2	-0.058763	0.108097
RPL27A	0.07853261	0.108798814
ZBTB17	-0.0459041	0.109093999

PLB1	-0.0532679	0.109507129
SELENOH	0.14559091	0.110498554
LINC01006	0.02847042	0.110697405
ATP6V0A1	0.00291106	0.111757027
HERC6	0.02759766	0.11344849
PRDM15	0.02402082	0.114219397
MMP28	-0.0576375	0.115203318
SLC27A4	0.03357584	0.115555278
BGLAP	-0.0259024	0.11719286
TENT4A	-0.0241036	0.118198211
CCL23	-0.0159671	0.120553662
AC114980.1	-0.0262502	0.122971084
STK32B	0.0351719	0.126056264
CD180	-0.0739144	0.126827175
RNF217-AS1	0.022691	0.129242601
MON1A	0.01285167	0.129926991
SSBP1	0.07277853	0.131374498
COX7B	0.06279901	0.131492936
AP002381.2	-0.0017319	0.134876578
AC010359.3	-0.0499049	0.135501582
DYNLL1	-0.0302439	0.136847721
CEACAM21	-0.0396543	0.137142347
AC092164.1	0.01042298	0.140020068
NDUFA11	-0.0192118	0.141715116
ZNF223	-0.0095053	0.144173486
FGL2	0.11573239	0.14806191
FBXO5	0.01208247	0.148440113
PROSER2	0.00029057	0.149957514
SNX3	0.14073619	0.150270998
LINC02848	0.00406536	0.150743476
ARMCX5	-0.0017692	0.152382719
AL080276.2	0.02290207	0.154042399
MIEF2	-0.0373266	0.154915696
PIGQ	-0.0085362	0.156142046
USP49	0.03072781	0.156395715
CHCHD6	0.01286019	0.156422463
AC004943.2	0.01798881	0.157060556
STARD7-AS	0.027685	0.158165749
DNAJA4	-0.0229536	0.15964101
AMMECR1	0.02750275	0.165634832
REEP5	-0.0023208	0.16655604
GLS	-0.0361021	0.169815784
LMO2	0.1422747	0.171364212
ZNF710-AS1	0.05160686	0.171840827
LINC02328	-0.0114049	0.172588858
HIGD2A	0.04649658	0.173794763
TSPAN17	0.00609376	0.175219191
RHOBTB3	0.03783993	0.176892328
SRSF9	-0.069036	0.177546338
NLRC5	-0.0204412	0.178836313

PEX11A	0.01389323	0.179716983
ARRDC4	-0.0247169	0.179840522
WDR83OS	-0.035051	0.180116098
SENP1	0.00141677	0.18270639
AC011603.2	0.02512513	0.182731425
ARPC1B	-0.1574212	0.185263395
LINC02361	-0.074983	0.185380343
MINK1	0.01713739	0.186913222
HIST1H2BE	0.01654988	0.187452204
CAMTA2	-0.0059915	0.188241781
CAPS	-0.0132724	0.189736814
PRR5L	-0.0381481	0.191194523
KBTBD11	0.02777503	0.193576391
CEACAM4	-0.0562375	0.194281165
NAALADL2	0.02843657	0.195685792
NCALD	0.00551552	0.196146744
CLINT1	-0.0389374	0.196477814
RPS12	0.08500723	0.199899253
TAF1A	0.00096507	0.201820819
HMGA1P4	-0.0137069	0.204530774
METTL22	0.00044226	0.208942873
ZNF662	0.02382301	0.209637754
ATAD3C	0.03714122	0.209896153
HGH1	0.03298622	0.2103612
PRDM2	-0.0805087	0.210394625
ACTR5	0.01093184	0.210502055
RARG	-0.057264	0.216071037
NPW	-0.053079	0.216539744
MEF2B	-0.0047925	0.217570139
ADAM9	-0.0423263	0.218768901
RACK1	0.09445763	0.219014422
RIPK3	-0.0466849	0.228607843
PAN2	0.00752486	0.229584902
ZNF473	0.01628031	0.230096224
RAB11B-AS1	0.00785567	0.230682732
VILL	0.01483606	0.231294942
TCTN2	0.01226503	0.232623644
CYB5D1	-0.0257229	0.237413469
ZNF880	-0.0844424	0.241446529
MGAT5	0.00910931	0.242942464
ZNF684	-0.0191755	0.244669465
R3HCC1L	-0.0199628	0.252555759
AP003486.1	0.02150796	0.254015057
CKM	-0.0136693	0.25446351
NOD1	0.00345964	0.25793064
CRADD	0.01544237	0.25933439
LINC01004	0.029604	0.259727075
KHDC1	0.02749737	0.264935782
THRAP3	-0.0250863	0.26591314
FAM161B	-0.0114577	0.26605814

HOMER1	-0.0195042	0.267410351
ADPRH	-0.0004242	0.269334967
DICER1-AS1	0.04562021	0.269391821
ZNF189	-0.0023701	0.275196422
SESN2	-0.0745576	0.280533576
ZBTB9	0.00289963	0.281667445
AMN1	-0.0029528	0.286870803
BAZ2B	0.0227067	0.297735715
TCTE3	0.01622189	0.298917922
CACNA1A	0.00094085	0.299013662
GLYCTK	-0.0713927	0.303423722
HHAT	0.01547181	0.306001578
AL139260.1	0.02946119	0.306607889
EMB	-0.1274742	0.308072352
MLLT11	-0.0798737	0.320230326
TANC2	0.02817977	0.32027923
NYNRIN	-0.0168366	0.321556031
GNAI1	-0.0486159	0.322718224
BUB1	0.02343374	0.327775259
AL133523.1	0.01010332	0.32809973
C1QTNF4	-0.1326889	0.328281854
ZNF773	-0.0126703	0.329572142
IL12RB1	-0.015107	0.340371701
AL050309.1	0.02713332	0.343058015
AC093827.4	-0.0105262	0.353742764
AC099778.1	0.01931922	0.356592371
IPPK	-0.0194169	0.365523347
LCMT2	0.04363691	0.3695756
KMT2E-AS1	-0.0067862	0.377610199
KIAA0556	-0.0369594	0.381762502
SPARC	-0.0642033	0.390589941
TMEM116	0.02439491	0.39770215
AC091057.6	0.02234679	0.399985251
SRSF11	0.01320158	0.409905025
GNB1L	0.03846708	0.412119376
EIF3A	0.08221454	0.413351277
SVOPL	0.02771942	0.415906775
TIE1	-0.0250776	0.41736303
BIRC3	-0.0625763	0.420046397
IL13RA1	0.05129218	0.428077442
AMT	-0.0053146	0.430234033
FAM24B	-0.0059899	0.434994947
C16orf46	-0.01207	0.445644617
ZNF142	0.00945183	0.45540405
CFAP70	0.01190484	0.462087222
SULT1A3	0.01576116	0.476164844
ENKD1	-0.0154004	0.492840725
ZNF526	-0.0363874	0.506900884
SEMA6B	-0.0300435	0.509471979
ASB1	-0.0322806	0.512135376

TMEM268	-0.013282	0.524563477
ARMC2	0.01334535	0.528588923
PAK1	0.01168765	0.52874133
AC245452.1	0.01000182	0.55671449
RGPD2	-0.0001937	0.557804253
DENND5B	0.04227873	0.564506426
FAAP24	0.02883365	0.567578158
SP100	0.08662982	0.573840469
ZNF14	-0.0036681	0.575452088
GATA2-AS1	-0.0510046	0.576922889
CENPS	-0.0230747	0.578889571
MAEL	0.02887052	0.580855665
IFT88	-0.0675172	0.60399185
PIM2	-0.0056537	0.630689179
AC130895.1	-0.0163221	0.64396366
ABLIM1	0.01622415	0.64716688
COX5A	0.0212079	0.658483003
CAPN10-DT	0.01426664	0.663408892
AC026979.4	0.02451833	0.664958139
MVK	-0.0157771	0.669311973
TAF1C	-0.0100416	0.675321741
CD69	-0.1221422	0.682664922
BBS7	0.0528852	0.695602947
MORF4L1	-0.1662403	0.702062671
ZFHX3	0.00080164	0.743003205
AC093849.4	0.02742316	0.746955714
GCC2	-0.0326777	0.754425665
TNFSF4	-0.0034373	0.768845813
AL356804.1	0.0468084	0.776087908
DNAJC27-AS	0.00417506	0.785827182
TTC8	0.01049073	0.797048582
CCDC142	-0.0189218	0.801638829
ZNF444	-0.0191704	0.844527784
PTAFR	-0.012322	0.85872754
HOXB-AS1	0.01502649	0.927146889
HNRNPK	0.10947862	0.943447396
AC012020.1	-0.0204054	0.969837095
SLC16A1-AS	0.00783629	0.977698652
ZMYND8	0.06546638	0.986619233
ZNF799	0.00255903	1
HDAC9	-0.1090862	1
DZANK1	0.0099097	1
DSCC1	0.01377353	1
TMEM86B	-0.0738291	1
KYNU	0.01791814	1
LRP12	0.00281914	1
MT-CO2	0.11294877	1
TMEM237	0.02468482	1
SLC4A2	-0.0222978	1
UFC1	0.06914938	1

ZNF513	-0.0148841	1
NAGA	0.01474142	1
IFT81	0.00579694	1
ZNF691	-0.0083265	1
GAS2	0.0520269	1
MICALL1	-0.027055	1
PTP4A2	0.08469515	1
HLX	-0.0004827	1
TRIM46	-0.0045928	1
AC009962.1	0.02793996	1
SLC44A2	-0.0686447	1
DDX11	0.02959645	1
AC000065.2	-0.0057497	1
ABHD17C	-0.008768	1
ABCC10	-0.0047891	1
NFKBIZ	0.04268288	1
NDUFB4	-0.0513618	1
AL117332.1	0.01019226	1
AACS	0.02319893	1
OSM	-0.0581365	1
MYO1B	-0.0562461	1
SLC35C1	-0.0250966	1
AC027279.1	0.01001222	1
AQP3	0.08788621	1
IGF2BP3	-0.1020266	1
SMIM13	-3.159E-05	1
HLA-DRB5	-0.1932009	1
MYO7B	0.01154928	1
FANCD2	0.00589566	1
FGGY	-0.0451962	1
AL049840.5	-0.0553579	1
POLR2L	-0.0383981	1
SEPTIN6	-0.0068009	1
VARS2	0.01796183	1
VPS9D1	-0.046058	1
SSR4	-0.1351864	1
KIFC3	-0.0944833	1
INPP5E	-0.0499289	1
RBM43	0.05475558	1
DNAJA1	-0.0182024	1
AC007686.3	-0.0532569	1
CLDN10	0.02430361	1
RNASEK	0.06957685	1
ATAD3B	-0.0251442	1
GPR141	0.02154663	1
C9orf16	0.05485025	1
CCNL1	-0.1161786	1
MICB	-0.0085765	1
LPAR2	-0.0447798	1
HIST1H2BD	-0.0553523	1

GTF3A	0.06529835	1
CTU2	0.02318163	1
AC040970.1	-0.0109837	1
GDPD1	-0.0522539	1
DDX21	0.05817662	1
EIF2AK3	-0.0017622	1
ATP5F1C	0.00770546	1
ECT2	0.00121241	1
HACD1	0.01883289	1
RPL7	0.06967126	1
ICAM4	-0.0024145	1
PIP4K2C	0.02518909	1
NCF1	0.0351639	1
PPA1	0.15728562	1
DBF4B	0.02831039	1
SLC25A5	0.11155792	1
MAX	-0.0401558	1
AC004812.2	-0.0282799	1
DSC2	0.0055145	1
C3orf80	-0.0537624	1
SFMBT2	-0.0172613	1
EIF5B	-0.0360856	1
FKRP	-0.0299493	1
CCDC26	0.03157574	1
PASK	0.03489661	1
FAM81B	-0.0291284	1
FBXO31	-0.0389071	1
AC091729.3	0.02024347	1
AL683813.1	-0.0065302	1
ZNF625	0.03834421	1
GSTO1	0.05606904	1
EHD2	-0.0546146	1
HAUS8	0.00586225	1
ACVR1	0.03383093	1
SUV39H2	0.024459	1
SERAC1	-0.0055448	1
SERPINI1	0.06408874	1
ARHGAP26	-0.0301395	1
ACTB	-0.1668166	1
MT-ND1	-0.1599368	1
DDX3X	-0.1202084	1
AL591623.1	-0.0542436	1
ITGA2B	-0.0654317	1
HEXIM1	0.0104806	1
AL050341.2	-0.0160851	1
ATP6V1G1	-0.0714352	1
AURKA	0.04627555	1
GABARAP	-0.1435718	1
SAMD10	-0.026337	1
TIGD6	0.00374453	1

GMFG	-0.1226918	1
PRDM10	0.02013326	1
PITPNC1	0.00380223	1
AP003068.2	-0.0361648	1
RPS14	-0.1191103	1
LAMTOR5-A	0.00485502	1
KLF11	-0.012428	1
AL451165.2	-0.0156129	1
LYL1	0.03986071	1
HCP5	0.02061123	1
ZNF771	-0.0029602	1
AL358780.1	-0.007772	1
ERP29	0.03552852	1
YY1	-0.0074533	1
GALNT6	-0.0205116	1
NPC1	-0.0107164	1
FAM27C	-0.0008933	1
SMIM14	0.03147824	1
REC8	-0.0087908	1
HIST2H2AA4	0.01353217	1
GDPGP1	-0.007156	1
RETN	0.00076058	1
CST7	-0.0847614	1
FCSK	0.01560508	1
AC095057.3	-0.0118818	1
CCNA1	0.0164413	1
AL118516.1	-0.0923167	1
CEBPD	0.0686605	1
PRXL2B	0.02288888	1
MICA	0.00542928	1
MYOF	-0.0175966	1
EIF1B	-0.1489676	1
ZSWIM4	-0.0684277	1
USP54	0.00837368	1
CLU	-0.213906	1
CYP2U1	0.02574711	1
NCAPG2	0.01278674	1
ZNF682	-0.0064172	1
TEX9	0.01424452	1
ENPP2	-0.0481738	1
LGALS3	0.0149058	1
ZNF528	0.03095351	1
AC009318.1	0.06147499	1
IBA57	-0.0230067	1
MAP11	-0.0103903	1
AL121944.1	-0.0729022	1
GLIPR1	-0.1102398	1
COX6C	0.01983409	1
HLA-DMB	-0.0501246	1
AC015982.2	0.02159164	1

HIST1H4H	0.00880784	1
AC092620.1	0.01135856	1
SUMO2	-0.107271	1
FOXP1	-0.0237637	1
AC069185.1	0.04013275	1
DXO	-0.0429775	1
TKT	0.08470644	1
AC015871.1	0.02163779	1
ACSF2	-0.0089837	1
TAOK3	0.07763065	1
RAN	-0.0683784	1
AC132192.2	-0.0069638	1
APOM	0.00088964	1
CDR2	0.01364658	1
HLA-DRB1	-0.0278334	1
PDZD8	0.05032531	1
ZNF763	0.00669468	1
TSPAN13	-0.0112114	1
AC009065.4	0.00960605	1
CCDC112	0.03208218	1
COX7A2	-0.0197939	1
GAN	-0.0029619	1
ZNF584	-0.0310823	1
MYL12B	-0.1244596	1
DCLRE1B	-0.0011998	1
DANT2	0.02640479	1
ARHGDIB	-0.1340634	1
DRAP1	0.0120317	1
MIR4453HG	-0.0207897	1
HNRNPH1	0.11286884	1
CHURC1	0.01011732	1
CALM2	-0.0443601	1
ALDH2	-0.0034232	1
ARF4-AS1	0.00999503	1
GNG5	0.01543249	1
CACNA1C-A	0.0105722	1
RPS15	-0.1114795	1
CDC37L1-DT	0.01651216	1
SYF2	0.02455719	1
TULP3	0.01458954	1
COX8A	-0.0307931	1
RHOB	-0.0668405	1
YTHDF3-AS1	-0.0435689	1
DOCK8-AS1	-0.0034905	1
EIF3L	0.04913183	1
KIF3A	0.00068649	1
TAF1D	0.07450647	1
BX640514.2	-0.0042425	1
ELOB	-0.0535173	1
RPS3A	0.06920187	1

SEM1	-0.0471444	1
EIF3F	-0.1201136	1
HLA-DQA2	-0.01632	1
C17orf49	-0.0555131	1
PTGS2	-0.1101245	1
HNRNPA2B1	0.03101633	1
AC091271.1	-0.0499616	1
HNRNPA0	-3.175E-05	1
RPL17	0.02270395	1
BASP1	-0.0285878	1
TSPYL2	-0.0052183	1
ACRBP	-0.0392624	1
RPS16	0.04210237	1
SNHG29	0.03824203	1
ACY3	-0.0017865	1
RAB3IP	0.00614192	1
C11orf95	0.00010702	1
NSMF	-0.0384774	1
SELL	-0.035311	1
CALR	0.00245673	1
DGCR6	-0.0388785	1
RPL23A	0.03807511	1
STX2	-0.0279644	1
MALAT1	-0.0047043	1
SLC25A45	0.00893193	1
HIST1H2AG	-0.0155675	1
AC127496.5	0.01484006	1
RTRAF	0.05204629	1
HLA-DQB1	-0.0386621	1
AFF3	-0.0245504	1
AL355075.4	-0.0512313	1
JHY	-0.0308195	1
C11orf58	-0.010692	1
SNHG7	0.15102709	1
POLR1D	0.00334631	1
MT-ND4	-0.0842852	1
ANKRD12	-0.0689046	1
SERPINF1	-0.0218818	1
ATP5F1D	0.04143848	1
RSPH3	-0.019895	1
NQO1	-0.0088753	1
GSTP1	-0.0813084	1
MBNL1	-0.066606	1
HMGB3	-0.0505126	1
PHYHD1	0.01467014	1
SLC25A3	-0.0694091	1
FAM161A	-0.0137515	1
MFGE8	0.01431552	1
IER5L	-0.0403669	1
OAZ1	-0.1003634	1

RPL14	0.04000178	1
TSC22D1-AS	0.01602652	1
PCK2	-0.041861	1
HIST2H2BF	0.01015347	1
GPR35	-0.0424168	1
PRDX1	-0.0026512	1
AREG	-0.0507654	1
POP1	0.01210092	1
ARPC3	0.0342907	1
CKB	-0.0451086	1
PSME1	0.0705655	1
PPIA	-0.0650312	1
PLAC8	0.23314372	1
PEBP1	-0.0666826	1
RAD51B	0.00715022	1
SERP1	0.07327529	1
ZNF710	-0.0089589	1
CSRNP2	-0.0175447	1
RPLP1	-0.0458409	1
HLA-DQA1	-0.0262931	1
RPS7	-7.214E-05	1
LINC00211	0.00385705	1
ARPC2	-0.0578358	1
LAPTM5	0.00697289	1
RPLP0	-0.0060576	1
AC002467.1	-0.004919	1
ZNF165	-0.0578164	1
EIF4A1	-0.0919315	1
RPL6	-0.0971659	1
MT-ATP6	-0.04199	1
LAT	-0.0156077	1
EIF3H	-0.0284062	1
FXYD5	0.03598181	1
HIST1H3H	-0.0127967	1
RPS2	0.00082084	1
ITM2B	-0.1114254	1
EIF3K	-0.0283007	1
AC087239.1	-0.0266292	1
RPS8	-0.0576144	1
SLFN5	0.00421172	1
RPL18	-0.0199209	1
ID2	0.02060094	1
OST4	-0.0886735	1
NOP53	-0.0181293	1
EIF1AX	0.02580248	1
SLC25A6	-0.0410395	1
H2AFY	-0.1855225	1
MT-ND5	-0.044906	1
RPS19	-0.064991	1
RPL35	-0.0259534	1

RSL24D1	-0.0287695	1
UBA52	-0.0234031	1
EEF1B2	-0.0748628	1
RPS18	-0.0547155	1
NDUFA4	0.05027671	1
BTF3	-0.02879	1
RPS6	-0.0370153	1
ANP32B	-0.0461142	1
ENO1	-0.0284091	1
MT-CO1	0.00344187	1
HINT1	-0.0073061	1

lls after standard culture versus pre-culture (reference). Only genes expressed in >10%

% of single cells are reported. Log2 Fold Change (FC) and FDR-adjusted p-value.

Supplementary Table 11. Differential gene expression averaged across 500 progenitor

Gene	log2FC	FDR q-value
ANXA1	-2.3375846	0
MYADM	-2.8060444	0
TAGLN2	-1.9334824	0
VIM	-3.4069547	0
TSC22D3	-2.8462124	2.77974178057077e-318
LMNA	-3.6404171	6.02E-308
CRIP1	-3.8948268	2.00E-286
RGCC	-3.2919423	1.84E-250
EMP3	-1.8746542	3.26E-216
ADGRE5	-1.7790391	4.78E-213
ATP2B1	-1.9134165	2.81E-211
CD99	-1.4483342	6.52E-176
EMP1	-1.8965051	1.51E-162
UBC	-1.1812184	2.12E-161
LRRFIP1	-1.3110344	1.00E-160
IRF1	-1.5670636	2.34E-156
TGFB1	-1.2590533	2.40E-156
TUBA1A	-1.8150254	2.47E-156
EGR1	1.4080353	2.83E-148
S100A11	-1.8170291	9.76E-146
SAT1	-1.5612057	9.75E-140
UCP2	-1.4462797	2.12E-139
CLIC1	-0.9032945	1.87E-138
ANGPT1	1.4088588	3.38E-137
TUBB4B	-1.3572877	3.30E-136
DNAJB6	-1.1722718	1.26E-135
IDS	-1.2086669	1.05E-128
CD44	-1.121522	3.12E-127
EIF1	-0.7630081	9.35E-124
RARA	-0.9502257	3.64E-120
PLCG2	1.0773338	2.43E-118
MYC	1.3047563	1.45E-114
RAB11FIP1	-1.1289048	1.06E-113
ITGA5	-1.0911463	4.75E-113
TNFRSF12A	-0.9242382	1.25E-112
RUNX3	-1.2366208	2.79E-112
AHNAK	-1.4763885	6.31E-111
MAP2K3	-1.0552356	4.56E-109
MIDN	-1.0976542	1.78E-108
TIPARP	-1.2088022	9.93E-108
HES1	-1.2048366	2.04E-106
CRIP2	-0.7447854	2.19E-105
AC020916.1	-1.4169928	1.54E-103
PER1	-0.8352433	2.45E-102
MYL6	-0.8125559	2.70E-102
RELB	-0.8180123	3.13E-101
RASL10A	-0.6792563	2.60E-100

TUBB6	-0.9245059	4.68E-100
RTN4	-0.9604063	1.38E-98
HSPA5	-1.8358802	1.43E-97
CXXC5	-1.0838766	2.11E-97
RALGDS	-1.0280196	2.02E-94
RAP1B	-0.9034915	3.04E-93
AKAP13	-0.9864108	3.85E-93
KMT2E	-0.8690247	1.51E-91
SERBP1	0.7647731	1.90E-89
GABPB1-AS	1.0452194	2.63E-89
ZBTB7A	-0.9680911	1.34E-88
CREB5	-0.9505609	2.01E-88
RPS27	0.4148093	6.83E-88
TMSB10	-0.7329173	8.06E-88
RBM38	-0.8145607	4.06E-86
SLC40A1	0.9754483	2.11E-85
TPSAB1	1.3237574	2.15E-85
SKIL	-0.871665	6.27E-85
CDKN1A	-0.7174621	1.82E-84
PHLDA2	-0.7528447	1.06E-83
MGST1	1.086087	2.94E-83
SH3BGRL3	-0.7578811	3.61E-83
DSTN	-0.8899183	1.89E-82
PRXL2C	-0.8148852	1.13E-81
KCTD9	-0.9431098	1.74E-81
FSCN1	-0.7832123	1.94E-81
GNA15	-0.7784873	2.42E-81
PLK3	-0.8303839	3.21E-81
NFATC1	-0.7414664	4.40E-80
PLP2	-0.8008794	8.13E-80
GPR183	-1.1209152	3.31E-79
EFHD2	-0.8720996	3.69E-79
FLT3	1.0137087	4.33E-79
DDIT4	-0.8930427	6.91E-79
FYB1	0.8234275	4.22E-78
RHOG	-0.7679749	2.27E-77
H1FX	-1.4189585	2.04E-74
LARS	0.7769379	2.14E-74
ARID4B	-0.8675474	4.61E-74
FTH1	-0.8614378	6.69E-74
CALM1	-0.89153	9.37E-74
URI1	0.7396917	3.01E-73
MTURN	-0.7854517	2.69E-72
PDLIM1	-0.6918546	3.65E-72
KLF13	-0.8309398	5.95E-72
ARF6	-0.7281482	6.56E-72
JUND	-0.870247	1.31E-71
GLIPR2	-0.7445711	2.69E-71
NCL	0.8140783	3.14E-71
RPS29	0.4217363	3.66E-71

CTSC	0.769119	1.13E-70
NINJ1	-0.8286644	2.94E-70
RPS17	0.6291208	4.73E-70
SLC9A3R1	-0.880831	6.31E-70
EXOSC5	0.4991194	6.99E-70
PPP1R15A	-0.8623278	1.29E-69
PLEC	-0.7650252	2.69E-69
RASGEF1B	-0.6399277	3.83E-69
ELMSAN1	-0.8180367	8.56E-69
RHOA	-0.7152543	9.04E-69
ADAM17	-0.8321232	2.90E-68
MT-ND6	0.8328146	4.36E-68
NAP1L1	0.6942548	7.12E-68
TRA2B	0.7431242	8.16E-68
RILPL2	-0.7280735	1.02E-67
HIC1	-0.4801841	1.19E-67
SAMHD1	-0.5561217	1.31E-67
TNFSF13B	0.7386027	3.37E-67
LINC02470	0.5493023	1.09E-66
SNHG6	0.5946499	2.51E-66
S100A10	-1.1342465	4.20E-66
YWHAZ	-0.8344214	7.69E-66
RGS18	0.6505081	8.56E-66
PPP1CB	-0.7346874	1.34E-65
HSP90AB1	0.5774752	2.15E-65
SQSTM1	-0.8276299	2.69E-65
SLC16A3	-0.760693	8.38E-65
HIPK1	-0.7511448	9.53E-65
CIRBP	-0.6498825	1.20E-64
LGALS1	-1.6152859	2.18E-64
NUCB2	0.7411786	2.26E-64
CITED2	0.7506561	5.02E-64
IL1B	0.6108414	8.06E-64
MT-ND3	0.5625418	8.15E-64
SYNJ2	-0.8020373	1.01E-63
CDC42	-0.5961551	1.35E-63
RSL1D1	0.676936	1.36E-63
C16orf54	-0.693213	1.53E-63
HSPD1	0.8049305	1.94E-63
CTNNB1	-0.7862111	2.08E-63
CD74	-0.8299169	1.93E-62
FOS	1.7253516	3.02E-62
BCL3	-0.6176758	3.27E-62
MYB	0.7631585	4.92E-62
TPPP	-0.4144221	1.24E-61
NACA	0.4265483	1.78E-61
WTAP	-0.7029793	2.47E-61
SEPTIN11	-0.7584018	2.75E-61
HSPE1	0.7400543	3.96E-61
EZR	-0.8252969	6.32E-61

RPL23	0.5565163	9.15E-61
UBB	-0.6368248	1.01E-60
PPIL4	-0.6866952	1.04E-60
RAC1	-0.5814325	1.04E-60
AL034397.3	0.6876035	1.54E-60
TREM1	-0.7461672	3.45E-60
MAT2A	0.691087	6.06E-60
CSNK1A1	-0.6472702	6.95E-60
NAIP	0.4795289	1.68E-59
PRKDC	0.7490084	2.30E-59
MAP7D1	-0.6579252	2.33E-59
SRSF7	0.8146123	2.74E-59
FBXO34	-0.7228079	3.52E-59
ID1	-1.4518007	4.51E-59
RELL1	-0.6900161	5.26E-59
SGMS2	-0.5088485	1.06E-58
BHLHE40	-0.7521365	3.09E-58
PRR7	-0.5902877	2.15E-57
BCO2	-0.4949324	7.10E-57
KNOP1	0.5388451	7.16E-57
CD69	0.7925926	7.78E-57
KLF10	-0.7750911	8.33E-56
KLF3	-0.7688005	8.92E-56
GUCY1A1	0.8399423	1.27E-55
TSC22D1	-0.9992725	1.67E-55
PHF20	-0.7070596	2.56E-55
CYTH1	-0.714906	9.51E-55
TES	-0.7359663	9.51E-55
UBE2B	-0.6184324	1.01E-54
SARAF	-0.5948516	3.10E-54
ARID5A	-0.5980679	3.11E-54
PIM3	-0.7034108	3.54E-54
MPC2	-0.5922254	4.69E-54
RPL38	0.3892273	8.56E-54
PA2G4	0.705504	1.12E-53
KRAS	-0.6880215	1.40E-53
SMC4	0.8153522	1.87E-53
SH2B3	-0.5788013	5.42E-53
NDUFB1	0.5518209	6.31E-53
ARL4A	-0.7156848	7.64E-53
CFL1	-0.5831536	1.78E-52
DDAH2	-0.7207221	2.46E-52
UPP1	-0.6458077	2.59E-52
SRSF3	0.6365869	9.95E-52
G3BP2	-0.6379984	3.30E-51
CAPZA2	-0.6842469	3.42E-51
GPR132	-0.3693264	3.87E-51
KIT	0.6593851	4.41E-51
SPART	0.5780663	6.56E-51
CDKN2D	-0.5647668	1.31E-50

NOTCH1	-0.6077643	1.36E-50
LSP1	-0.7275904	1.69E-50
MBP	-0.6240107	2.46E-50
ICAM3	-0.6248732	2.88E-50
VAMP8	0.5308186	3.66E-50
ATP6V0C	-0.6077886	6.96E-50
ARGLU1	0.64963	7.03E-50
HIST1H4C	0.6885248	9.54E-50
ODF3L1	-0.3286139	1.31E-49
SLC35E4	-0.5338172	3.39E-49
TSPAN2	-0.4020165	4.35E-49
VSIR	-0.7551198	6.02E-49
CSRNP1	-0.4984515	7.21E-49
KPNA4	-0.5623653	7.56E-49
MAN1A1	0.5253519	8.49E-49
KDM6B	-0.7210531	1.23E-48
SAP18	-0.6208584	1.72E-48
CNBP	0.4960513	2.58E-48
LYN	-0.664876	3.83E-48
CD81	0.5694394	4.70E-48
DBI	-0.6873553	5.27E-48
BAIAP2	-0.487717	6.22E-48
JMJD1C	-0.6935987	1.41E-47
SIK3	-0.5901243	1.47E-47
IER3	-0.7125024	2.14E-47
AGPAT4	-0.5362766	2.61E-47
PNP	-0.6890413	3.10E-47
BPTF	0.6601391	3.37E-47
CYTH4	-0.6137942	3.41E-47
ORAI1	-0.5627058	3.47E-47
ZSCAN18	-0.5418669	3.48E-47
SORL1	0.5818594	6.11E-47
ANTXR2	-0.6214411	7.18E-47
IGLL1	1.0592249	8.24E-47
HMGN1	0.483043	1.30E-46
ZBTB43	-0.5959507	1.85E-46
SMAD7	-0.4343352	2.08E-46
AC245060.5	0.635308	2.11E-46
ZFAS1	0.6328955	3.30E-46
DENND4A	-0.5889258	5.39E-46
RGS19	-0.6468079	5.99E-46
ABCA1	-0.3850176	1.92E-45
PDGFA	-0.3855108	2.02E-45
DYNLT1	-0.525485	2.21E-45
MBOAT7	-0.6093199	3.31E-45
LUC7L3	0.5494009	3.57E-45
NPTX2	-0.4220788	3.80E-45
CA5B	-0.4670887	4.01E-45
IFT57	-0.5271033	4.25E-45
GAS2L1	-0.3297031	7.15E-45

MOB3A	-0.5207653	1.23E-44
SUCNR1	0.9348603	1.99E-44
TACC1	-0.6530757	2.70E-44
TMOD3	-0.5756527	2.78E-44
YPEL5	-0.8023338	3.37E-44
HIF1A	-0.699186	3.53E-44
VMP1	-0.755802	3.88E-44
CD302	0.5718772	1.14E-43
MTSS1	-0.3884714	1.24E-43
SRSF5	0.5584836	1.44E-43
RARA-AS1	-0.3752558	1.58E-43
PPP1R18	-0.4920837	1.72E-43
CD82	-0.6218657	1.89E-43
CMTM3	-0.5960847	3.50E-43
CMSS1	0.4330819	6.05E-43
TMBIM4	0.5583937	6.48E-43
NAMPT	-0.7123379	7.39E-43
DENND6B	-0.5192026	1.85E-42
R3HDM4	-0.5630016	2.13E-42
LRRFIP2	-0.6058263	2.61E-42
LFNG	-0.4474044	3.46E-42
TXN	-0.5770073	4.36E-42
RPS11	0.3771038	6.84E-42
TTC1	0.4037497	7.09E-42
PLEKHO1	-0.5914082	7.21E-42
H3F3A	-0.3697933	7.36E-42
PHLDA1	-0.6492804	8.53E-42
CD9	-0.6701429	8.55E-42
ARL4C	-0.4732219	1.27E-41
IGFBP2	0.6256122	1.49E-41
MIR22HG	-0.3995892	1.52E-41
IFFO2	-0.4692469	1.66E-41
APBB1IP	0.4692767	1.72E-41
CSNK1D	-0.5216966	2.48E-41
NOL8	0.4964878	3.09E-41
KLF6	-0.8423068	3.16E-41
TARBP1	0.5478536	3.80E-41
TRAF4	-0.5047161	3.81E-41
LINC01226	0.3313578	4.87E-41
ARHGDIA	-0.5202408	5.58E-41
TTC3	0.5874334	5.59E-41
HSD17B4	0.4407155	8.07E-41
AGO2	-0.5428538	9.87E-41
NFKBIA	-1.0302803	1.77E-40
IQGAP1	-0.5223736	1.85E-40
ETF1	-0.6363113	1.89E-40
DPYSL3	-0.2703327	2.14E-40
LINC01268	0.4363587	2.35E-40
TPM4	-0.6541821	2.54E-40
TPPP3	-0.3329547	6.06E-40

SERPINE1	-0.3938265	1.07E-39
ARL8A	-0.4947702	1.37E-39
TASOR	0.4046515	1.40E-39
STOML3	-0.2589197	1.84E-39
NFE2L2	-0.4922044	2.29E-39
MAP3K8	-0.5802278	3.07E-39
UBE2H	-0.5532945	4.19E-39
PPP1R16B	-0.2507074	5.43E-39
LRPPRC	0.4630461	5.50E-39
SEC61B	-0.4817619	9.94E-39
SLC20A1	-0.583456	1.13E-38
ARL2	0.4362942	1.20E-38
PNISR	0.5366203	1.67E-38
RPL37	0.3040759	2.49E-38
CYTIP	-0.5332449	2.52E-38
RAD50	0.5171434	3.31E-38
VAPA	-0.5576836	3.34E-38
HERPUD1	-0.5087419	3.89E-38
DNPH1	0.4577625	4.01E-38
TMX4	-0.7296808	6.72E-38
MYL12B	-0.4590654	7.63E-38
TNFRSF1A	-0.4818092	8.60E-38
FMNL1	-0.5296618	9.31E-38
PTPN6	-0.5940786	9.60E-38
UBE2D1	-0.5116769	1.27E-37
RCSD1	0.5138097	1.49E-37
MRPS34	0.5094458	2.27E-37
REXO2	0.4197182	3.60E-37
CHCHD2	-0.4481873	4.52E-37
ACTG1	-0.4514185	4.53E-37
LST1	-0.558072	5.20E-37
CFAP126	-0.2016711	6.97E-37
LDHB	0.4925257	7.07E-37
CD84	0.4651556	8.41E-37
SET	0.4700029	9.39E-37
MAFF	-0.5515908	1.76E-36
NPDC1	-0.4636966	1.81E-36
CDC42SE1	-0.567147	3.00E-36
ACTN1	0.4413068	3.28E-36
MCL1	-0.6960384	5.42E-36
H3F3B	-0.7132198	5.55E-36
MARCKSL1	-0.5994089	8.46E-36
YWHAQ	-0.4941178	8.69E-36
MTIF3	0.547542	9.48E-36
ZFP36	-0.8025204	1.04E-35
PYGL	0.4817426	1.32E-35
GAPT	0.5213686	1.74E-35
PSTPIP2	0.4871112	1.81E-35
HSP90B1	-0.9279393	2.32E-35
TNFAIP2	-0.6430352	2.32E-35

SYAP1	-0.8175588	2.38E-35
IL1RAP	-0.5659633	2.53E-35
THEMIS2	-0.5169115	3.21E-35
HIST2H2BE	-0.5126519	3.53E-35
TENT5A	0.4173048	4.40E-35
MT-CYB	0.4187455	4.72E-35
ARF4	-0.4743567	4.73E-35
ZMYM4	0.4181813	4.87E-35
PTP4A3	-0.4382202	5.41E-35
SFXN3	-0.4349944	5.96E-35
ATRX	0.5699843	6.02E-35
RNF19B	-0.3470818	7.79E-35
MIS18BP1	0.5468829	1.10E-34
HES4	-0.5173576	1.35E-34
RPL31	0.3294293	1.47E-34
ABRACL	0.4305895	1.54E-34
FDX1	-0.4625654	1.66E-34
SERTAD1	-0.4641028	2.75E-34
GPATCH2	-0.4610001	3.25E-34
AL355922.1	0.3269544	3.26E-34
SPNS2	-0.4572435	3.32E-34
PM20D2	0.3489306	4.28E-34
SRSF6	0.5074028	4.45E-34
SUB1	-0.4585105	6.77E-34
TFG	-0.4586121	7.59E-34
RNASE2	0.6309031	1.05E-33
S100A6	-0.840855	1.21E-33
BLVRB	0.4367964	1.38E-33
RPAP2	0.5562298	1.54E-33
LDHA	-0.546952	1.60E-33
TRIM73	0.3863822	1.89E-33
RNASEH2B	0.6327498	2.41E-33
NARS	0.4755526	2.53E-33
FUS	0.5113834	2.54E-33
CMTM6	-0.4841485	2.64E-33
PRKAG2-AS	0.2337129	2.97E-33
PDCD11	0.3383597	3.00E-33
LINC00342	0.5418145	3.22E-33
MAP1LC3B	-0.5662637	3.22E-33
SNX2	-0.5570395	3.52E-33
OMA1	0.2188811	3.64E-33
SH3BP5	-0.3082628	4.22E-33
GNAI3	-0.5241011	4.82E-33
PRDX3	0.4401641	4.91E-33
FAM216A	0.3776377	5.63E-33
CYTL1	0.8360609	6.42E-33
AC007384.1	-0.3467484	6.65E-33
CHMP6	-0.3455898	7.05E-33
PER2	-0.4423763	9.28E-33
GTF2I	0.5269173	1.12E-32

RSRC2	-0.5368078	1.48E-32
EHD1	-0.4379772	1.49E-32
UTP3	0.3767315	1.70E-32
ACOT7	-0.3129868	1.73E-32
PACSIN2	-0.4366821	2.09E-32
TSG101	-0.4411351	2.25E-32
CLEC12A	0.3442791	2.44E-32
YWHAH	-0.5392277	2.63E-32
BCL2	0.37034	2.90E-32
HLA-B	-0.4646339	4.10E-32
CBX3	0.4284236	4.73E-32
ISOC2	0.3254437	4.86E-32
LEPROTL1	-0.4672052	5.19E-32
SLC39A3	0.4788312	6.32E-32
CD83	-0.4297129	6.53E-32
NR3C1	-0.4779215	6.71E-32
SPIN4	0.4338707	8.77E-32
PTGDS	0.8703575	1.03E-31
SUPT16H	0.4904348	1.87E-31
NAA38	0.416509	1.90E-31
GCSH	0.4030037	1.94E-31
MSI2	0.5698156	2.02E-31
NOP56	0.5826406	2.51E-31
GPATCH11	-0.468114	2.59E-31
MRPL33	0.4266489	2.90E-31
GNAI2	-0.4855576	3.42E-31
PABPC1	0.4220794	3.80E-31
C1QBP	0.4851443	4.19E-31
RIOK3	-0.4870801	4.76E-31
BCL7B	-0.4183492	6.61E-31
RHOF	-0.4850367	6.61E-31
SRM	0.5217015	8.95E-31
TRIM8	-0.4917448	8.97E-31
CCT5	0.4538808	1.15E-30
PARP4	0.3920982	1.44E-30
ARG2	-0.2553397	1.68E-30
VASP	-0.4865699	1.82E-30
NRDC	0.4463067	2.05E-30
FAM214B	-0.3230689	2.41E-30
TSEN34	-0.4138541	3.77E-30
PPP2CA	-0.4058733	4.36E-30
FIRRE	0.2836104	4.52E-30
MRPL3	0.4020159	4.61E-30
MCRIP2	0.351326	6.62E-30
ALOX5AP	-0.5452218	6.80E-30
UBE2D2	-0.4162559	7.32E-30
RFX2	-0.4163138	7.46E-30
IFI16	0.6099459	7.83E-30
GPR174	0.3533733	8.01E-30
GAPDH	-0.3211698	9.60E-30

H19	-0.7186365	1.08E-29
HNRNPUL1	-0.4987217	1.10E-29
MTMR6	-0.3674691	1.13E-29
ZNF487	-0.2999361	1.31E-29
HERC2	0.3853607	1.77E-29
ANXA11	-0.398664	1.89E-29
YTHDC1	0.3850192	2.16E-29
LDLR	-0.3339928	2.48E-29
WDR3	0.2504053	2.55E-29
HNRNPA1	-0.4072769	2.67E-29
PFKFB3	-0.3498834	3.28E-29
NPC2	0.4063589	4.26E-29
NEDD8	0.3873428	4.72E-29
ELF1	-0.5173013	6.27E-29
TMEM14C	0.3997617	8.17E-29
RHOC	-0.4354427	9.91E-29
CASP8	-0.483866	1.02E-28
UBTF	0.3813886	1.05E-28
CHD2	-0.551567	1.13E-28
RBM3	-0.419865	1.26E-28
DAAM1	-0.5089886	1.29E-28
RUFY1	0.3928805	1.32E-28
WDFY2	-0.6144689	1.40E-28
NREP	0.4225223	1.44E-28
VEGFB	0.3162413	1.47E-28
MPHOSPH8	0.4034055	1.54E-28
LIMD2	-0.466651	1.59E-28
TMEM161B-	0.4935299	1.80E-28
MAPK1IP1L	-0.4563797	2.10E-28
UQCR10	0.3914008	2.22E-28
CD53	-0.4325378	2.26E-28
HNRNPAB	0.5298004	2.63E-28
PYURF	0.4043256	2.73E-28
RBM8A	-0.389449	2.97E-28
CDK6	0.4285245	3.56E-28
ARL6IP5	0.5473924	4.18E-28
CST7	0.5428958	4.37E-28
U2SURP	0.4476832	5.01E-28
RRAGC	-0.4258962	5.06E-28
RBM28	0.3921197	5.07E-28
PNMA1	-0.3113951	6.10E-28
EPRS	0.42992	6.38E-28
ATP8B4	0.4874078	6.67E-28
PDCD5	0.4396378	6.84E-28
RNF11	-0.3630342	7.03E-28
OPN3	0.2485311	7.30E-28
UBE2N	-0.4316951	8.50E-28
LAPTM5	-0.4853274	1.03E-27
PARP1	0.4437509	1.07E-27
IFI30	-0.6519375	1.12E-27

FAM122C	-0.3486064	1.22E-27
TRMT10C	0.3934618	1.33E-27
INKA2	-0.2911967	1.33E-27
PRKD2	-0.3399275	1.35E-27
GAS5	0.4593563	1.52E-27
RPL36	0.2490545	1.59E-27
MAPK1	-0.425922	1.64E-27
PLEKHF1	-0.3251175	1.64E-27
CD300A	-0.3897043	1.67E-27
MAP1S	-0.4238352	1.80E-27
EDEM3	0.3385141	1.84E-27
LUZP1	-0.4816989	1.88E-27
HOXB2	-0.5177517	2.25E-27
CYLD	-0.453583	2.26E-27
PPP4R3B	0.4312014	2.89E-27
ALG8	0.1897058	2.98E-27
DAB2	0.2605743	3.70E-27
MTAP	0.3206512	4.53E-27
RPL35A	0.2578472	5.24E-27
DOK2	-0.3961579	5.81E-27
GPATCH4	0.3622565	6.05E-27
UBE2D3	-0.3700903	7.61E-27
PPP6R1	-0.3981321	7.66E-27
CITED4	0.2357935	8.52E-27
ELL2	-0.3606303	8.98E-27
ENSA	-0.4024998	9.23E-27
YBX3	-0.594525	1.19E-26
TMPO	0.4413042	1.28E-26
SPI1	-0.5094172	1.39E-26
OSGIN2	-0.3931956	1.54E-26
NUCKS1	0.4782493	1.56E-26
TMCC1-AS1	0.3224042	1.63E-26
RAB14	-0.4296358	1.67E-26
MT-CO3	0.3002419	2.47E-26
MLF2	-0.2913322	2.53E-26
DUSP14	-0.4091606	2.66E-26
RRP12	-0.4740455	2.80E-26
USP48	0.3556346	2.86E-26
PRKCQ-AS1	0.3008669	3.12E-26
N4BP3	-0.2850269	3.20E-26
EHMT2	0.2936873	3.39E-26
CD58	-0.4423505	3.59E-26
PUS7L	0.2577772	3.65E-26
EXOSC6	0.337063	3.68E-26
TWF2	-0.467065	3.92E-26
GGCT	0.433065	4.29E-26
AC104083.1	0.2298882	5.14E-26
CDC42EP3	-0.479515	5.71E-26
RUVBL1	0.3459339	6.16E-26
HTR1F	0.2420243	6.32E-26

APRT	0.3745146	6.35E-26
CFP	-0.3482519	8.25E-26
AGPAT5	0.3777904	9.90E-26
MKNK2	-0.4170284	1.02E-25
WDR12	0.3290065	1.02E-25
BZW2	0.4159864	1.03E-25
RIPOR2	-0.443415	1.10E-25
PAK2	0.3527014	1.22E-25
RPL13A	0.2397577	1.28E-25
AC007952.4	0.4587509	1.41E-25
ST13	0.3802106	1.56E-25
JUP	-0.4043263	1.75E-25
M6PR	0.387853	1.89E-25
IRF8	-0.3640664	1.92E-25
CEP290	0.3955645	2.02E-25
MFSD1	0.3868786	2.22E-25
RPL37A	0.2744683	2.64E-25
RPS27L	0.4456116	2.67E-25
SKAP2	0.508441	2.99E-25
LRRC8C	-0.3996379	3.12E-25
PTPRC	-0.4872549	3.46E-25
RBM14	-0.4296195	3.77E-25
TOP2B	0.5177091	4.23E-25
ZMIZ1	-0.457919	4.36E-25
DDX46	0.4155784	4.62E-25
DLD	-0.4701308	4.78E-25
ARHGAP22	0.2987403	5.15E-25
SMAD3	-0.3877674	5.44E-25
VPS37B	-0.3986036	5.71E-25
RPS9	-0.2419936	5.84E-25
MALT1	-0.3915809	7.17E-25
ACADM	0.413549	7.20E-25
RABAC1	-0.3641373	9.77E-25
CBX1	0.3217032	1.10E-24
RPL34	0.2460945	1.12E-24
CYTOR	-0.4735267	1.21E-24
SNAI3	-0.4524734	1.39E-24
MT-ATP6	0.2741588	1.57E-24
MYCBP2	0.4532995	1.70E-24
OCIAD1	0.3798974	1.82E-24
THUMPD3-A	-0.358573	1.93E-24
NEU4	-0.2445856	2.17E-24
MRPS12	0.3611981	2.31E-24
RPS28	0.2264395	2.81E-24
TMEM63A	-0.3782149	3.10E-24
TLE5	-0.4351137	3.21E-24
KANK1	0.190365	3.43E-24
RNF10	-0.3737844	3.78E-24
APEX1	0.4226282	3.90E-24
MYCN	0.2758208	4.15E-24

FHL3	-0.3598817	4.18E-24
FN1	0.1256373	4.24E-24
CHD1	-0.559504	4.28E-24
PCF11	0.4403186	4.47E-24
SNRPE	0.3887782	4.63E-24
NOLC1	0.3704232	4.80E-24
CNTRL	0.4100993	4.87E-24
OSER1	-0.336417	5.14E-24
PTPRA	0.3543579	5.18E-24
NPM1	0.3400377	5.33E-24
MFSD6	-0.3895309	5.94E-24
TTC17	0.3907198	6.21E-24
LYAR	0.2784104	6.30E-24
POLR3K	0.2401213	6.94E-24
FCER1G	-0.510657	7.92E-24
CYBA	-0.3783595	8.10E-24
RBM12B	0.331219	8.41E-24
PLEKHO2	-0.3781697	9.28E-24
MRPS26	0.3669248	1.02E-23
ARHGEF7	-0.3304538	1.08E-23
SMC2	0.3435904	1.22E-23
TRAF5	-0.3780219	1.22E-23
C11orf68	-0.26845	1.34E-23
FRAT2	0.2439079	1.39E-23
CAT	0.4467759	1.40E-23
SERF2	-0.2736622	1.41E-23
ICAM1	-0.2627676	1.51E-23
VDAC3	0.3599857	1.53E-23
STRAP	-0.3871868	1.75E-23
RIPK2	-0.390782	1.77E-23
MGST2	0.3910179	1.92E-23
EMC3	-0.3915373	2.11E-23
LINC-PINT	-0.4424347	2.17E-23
XAF1	0.3163798	2.34E-23
TPM3	-0.3951619	2.90E-23
NCOA3	-0.4786993	3.06E-23
FAM217B	0.3011496	3.09E-23
H2AFJ	-0.4449077	3.33E-23
TFAP4	0.1608797	3.47E-23
SH2D2A	-0.2408885	3.49E-23
RPL36AL	-0.3015458	3.66E-23
RPL30	0.2085428	3.67E-23
FABP5	0.5013611	3.79E-23
STK24	-0.3675779	3.80E-23
EIF5	-0.478268	3.82E-23
HMGB1	0.4267276	4.06E-23
SLC25A19	0.2002508	4.13E-23
DUSP23	0.3067648	4.21E-23
N4BP2	-0.4709413	4.65E-23
MIER1	-0.4270949	4.79E-23

RPS20	0.3272493	5.16E-23
PIGM	0.2666526	5.20E-23
HIST1H1C	-0.8248726	5.35E-23
MAP7	0.385792	5.60E-23
STK40	-0.3195348	5.72E-23
FAM83G	-0.205696	6.05E-23
IGSF10	0.3203951	6.55E-23
SOX4	0.7791989	6.78E-23
TFPI	-0.4229401	6.82E-23
ERF	-0.3653253	6.91E-23
CHST12	0.3828709	7.07E-23
SLC39A4	0.2614693	7.25E-23
ARHGAP27	-0.4353999	7.30E-23
BOD1L1	0.4815339	7.30E-23
DDX1	0.3243626	7.48E-23
YRDC	0.2284378	7.64E-23
PDCD10	-0.4145055	7.90E-23
TCF7L2	-0.3708399	8.02E-23
TBCA	0.3528546	8.18E-23
PSMD12	-0.4183853	8.19E-23
KRT10	0.3702136	8.39E-23
NOP10	-0.3679934	8.99E-23
ICOSLG	-0.2515752	9.54E-23
EBPL	0.379804	1.17E-22
LYRM7	0.361022	1.20E-22
USP14	-0.4132619	1.24E-22
PTPN1	-0.4528043	1.44E-22
NUFIP2	0.4333327	1.68E-22
GFOD1	-0.3791466	1.70E-22
CAPNS1	-0.4042827	1.99E-22
FTX	0.4899256	2.02E-22
PPP6R3	-0.296617	2.08E-22
FHL1	-0.2714512	2.97E-22
ARID3A	-0.4789802	3.02E-22
PTPRE	-0.4398522	3.04E-22
HLA-A	-0.3737763	3.16E-22
DMAC1	0.3808127	3.56E-22
ST6GALNAC	-0.3176061	3.69E-22
FOSL2	-0.4562634	3.70E-22
CHD4	0.3896321	3.85E-22
RPL12	0.2516844	4.08E-22
C11orf54	0.2946912	4.20E-22
CDC42EP2	-0.1447938	4.45E-22
GTF2A2	0.3789462	4.85E-22
HMGA1	-0.4583345	5.36E-22
MRPL34	0.3968592	5.74E-22
COMMD6	0.3862432	6.32E-22
ATF3	-0.5274778	6.36E-22
EIF4A2	0.395858	6.75E-22
FAM177A1	-0.3572201	7.32E-22

ATM	0.3767314	7.45E-22
SLC9A3R2	-0.4889479	8.54E-22
AIMP1	0.3673205	8.56E-22
TMA16	0.3709976	9.83E-22
FKBP4	0.3486638	1.07E-21
SLBP	-0.3154728	1.11E-21
SELENOP	0.5083611	1.17E-21
IQCH-AS1	0.2002427	1.27E-21
PDXK	-0.3333023	1.38E-21
CSTB	-0.3816028	1.43E-21
CEP63	0.3216339	1.46E-21
CELF1	-0.4278301	1.48E-21
CNOT2	-0.3065695	1.61E-21
ZBTB44	0.3547212	1.69E-21
KRCC1	0.4101959	1.78E-21
ERG	0.4063182	1.83E-21
C16orf74	0.2025817	2.03E-21
NDUFB3	0.3116833	2.09E-21
CCDC12	-0.3123557	2.29E-21
ACIN1	0.3482844	2.30E-21
Z97192.3	0.2718019	2.37E-21
COA6	0.2904616	2.39E-21
CEBPG	-0.3583098	2.41E-21
IRAK2	-0.1486419	2.72E-21
STK17A	-0.3553945	2.85E-21
ARRDC2	-0.3399825	3.11E-21
ST8SIA6	0.3293706	3.17E-21
TMEM14B	0.3314447	3.21E-21
SPAG9	-0.4126671	3.40E-21
NUDCD1	0.2305308	3.45E-21
INSIG1	-0.459844	3.64E-21
ENDOG	0.380108	3.70E-21
ZRANB2	0.3961963	3.72E-21
FOSL1	-0.2427579	3.78E-21
HDDC2	0.2660292	3.79E-21
POP5	0.2808732	4.87E-21
TREML2	-0.2000654	5.08E-21
NME1	0.5013788	5.75E-21
CFAP97	0.3569079	5.79E-21
KCNN4	-0.3652151	5.83E-21
C6orf226	-0.2832212	6.47E-21
ITM2C	-0.4767116	6.58E-21
PRMT2	-0.3532179	8.35E-21
MSRB3	0.3889059	8.62E-21
IP6K2	-0.3237663	8.83E-21
HLTF	0.3180711	9.74E-21
CCDC85B	0.4250601	1.04E-20
WASF2	-0.3444825	1.05E-20
ZFPM1	0.2270353	1.10E-20
NTPCR	0.2356354	1.14E-20

LMAN1	0.2886224	1.22E-20
SDCBP	-0.4935934	1.24E-20
PAQR8	-0.3270942	1.27E-20
TMCO1	0.387972	1.33E-20
EBNA1BP2	0.3266279	1.44E-20
HBP1	-0.3573504	1.45E-20
ZNF286A	0.1700283	1.45E-20
DDX18	0.3780656	1.47E-20
SLC44A1	0.3031831	1.56E-20
ALDH5A1	0.2117262	1.73E-20
RIN3	-0.3456474	1.76E-20
CENPJ	0.2921682	1.80E-20
DEPTOR	0.1746712	1.97E-20
RNF166	-0.430477	1.98E-20
HP1BP3	0.4047067	2.14E-20
SPON2	-0.3313726	2.34E-20
ZNF706	-0.3238128	2.41E-20
CORO1C	-0.3647115	2.57E-20
MEX3B	0.2292399	2.67E-20
HPF1	0.2566697	2.73E-20
DCTN6	-0.377484	2.88E-20
ARID3B	-0.3357898	2.94E-20
NXT2	0.2285857	3.10E-20
ARF1	-0.3720396	3.16E-20
NDUFAF2	0.2853167	3.28E-20
ZNRF1	0.3586576	3.29E-20
IRF2BPL	-0.3840874	3.87E-20
PSMB8-AS1	0.2445635	3.91E-20
PTER	0.1887132	4.07E-20
IL4I1	-0.1588595	4.25E-20
PLAUR	-0.3507594	4.37E-20
ZNF804A	0.2035091	4.63E-20
CREM	-0.2892761	4.65E-20
LMNB1	0.3288285	4.71E-20
TM7SF3	0.4529408	5.05E-20
STX1A	-0.1486448	5.11E-20
EEF1A1	-0.2377427	5.27E-20
IVNS1ABP	-0.302702	5.30E-20
SERINC1	-0.3847555	5.78E-20
CD164	0.5649357	5.78E-20
TRIM25	0.2206703	5.90E-20
MSRB2	0.3136392	6.16E-20
GUCY1B1	0.2233195	6.23E-20
RXYLT1	0.2874018	6.77E-20
GALNT1	0.3045592	7.40E-20
MRPS33	0.4026617	7.76E-20
CSF1R	0.2115503	7.90E-20
MAPKAPK2	-0.3236897	8.16E-20
AKIRIN2	-0.345272	8.66E-20
ARPP19	-0.3664807	8.67E-20

PPA2	0.3194576	8.86E-20
CKS1B	0.1974897	9.51E-20
RBIS	0.3008234	9.61E-20
HIVEP1	-0.3338444	1.02E-19
DCP1A	-0.3169916	1.04E-19
UBE2V2	0.2655042	1.06E-19
RAB5A	-0.3590012	1.07E-19
APOL4	0.145934	1.12E-19
IDH1	0.3363838	1.26E-19
MDN1	0.332447	1.27E-19
MRPS31	0.2729298	1.29E-19
NFKBID	-0.3613041	1.33E-19
IQGAP2	0.3843312	1.33E-19
DDX10	0.29967	1.37E-19
RHOH	-0.3232676	1.44E-19
CMBL	0.2660367	1.44E-19
HACD3	0.2566404	1.73E-19
PAXIP1-AS1	0.2357775	1.76E-19
SAMD9	0.2753064	1.96E-19
SETDB2	0.321035	1.96E-19
CBR1	0.3155954	2.22E-19
MAP1LC3A	-0.264213	2.33E-19
ID3	-0.2798301	2.37E-19
NR1H2	-0.3202514	2.59E-19
RAB8B	-0.3379146	3.54E-19
MFSD14A	-0.2668074	3.67E-19
EWSR1	-0.3861001	3.70E-19
GGNBP2	-0.3465782	4.10E-19
TMX1	0.3482	4.28E-19
TESC	0.3294924	4.39E-19
ARPC5L	-0.3702926	4.68E-19
NDE1	-0.3530326	5.03E-19
TFEC	0.3727728	5.06E-19
FAM174C	0.284635	5.14E-19
METAP2	0.3438671	5.20E-19
MGAT1	-0.3571149	5.64E-19
SLC43A3	-0.3038141	5.65E-19
MORF4L2	-0.3760059	5.76E-19
ATP5PD	0.3304772	5.83E-19
PRKACB	0.4336884	6.19E-19
MTMR12	-0.2603512	6.54E-19
LRMDA	0.2483863	6.73E-19
EIF3I	-0.3132967	7.13E-19
MCOLN1	0.1469446	7.60E-19
SLC6A6	-0.3356802	8.28E-19
PLCB1	-0.3991311	8.95E-19
ZNF480	0.2335256	9.35E-19
TIMM13	0.3611069	9.98E-19
GADD45B	-0.4539738	1.12E-18
ZNF737	0.2408324	1.28E-18

LRPAP1	0.3732531	1.28E-18
NDUFB10	0.3240295	1.28E-18
METTL5	0.2719683	1.29E-18
ZBTB21	-0.3147465	1.33E-18
SMIM12	-0.252236	1.43E-18
ITSN2	-0.3204479	1.52E-18
ZNF138	0.196127	1.62E-18
PSMA6	-0.3041649	1.63E-18
CRK	-0.2958233	1.70E-18
TOB2	-0.3365098	1.72E-18
FGR	-0.2400005	1.76E-18
ARRB1	0.2413929	2.01E-18
STXBP5	0.356117	2.05E-18
NSA2	0.3539068	2.15E-18
OSBPL1A	0.2613075	2.38E-18
HCG18	0.3888863	2.45E-18
WDR1	-0.3363316	2.50E-18
GAS7	-0.3221601	2.61E-18
PLEKHH3	-0.1841	2.98E-18
RIPOR1	-0.2680471	3.00E-18
MRPL40	0.2270617	3.06E-18
PDIA3	-0.3345087	3.14E-18
SPNS3	-0.4118006	3.26E-18
SMARCC1	0.3788324	3.41E-18
FADD	-0.2577909	3.94E-18
GMFG	0.2788221	4.17E-18
HADHA	0.3446274	4.45E-18
HMGN3	0.2990627	4.52E-18
HAT1	-0.3426658	4.54E-18
ZNF605	0.1745437	4.57E-18
ADGRG6	0.2609946	4.95E-18
ASAH1	0.4259613	5.06E-18
PFN1	-0.4515162	5.10E-18
ACSM3	0.3921876	5.56E-18
USP6NL	-0.3394786	5.85E-18
ZNF891	0.2440662	6.37E-18
DHX33	0.1745686	6.75E-18
ATP2A2	-0.3647974	7.01E-18
TMEM106B	0.294086	7.28E-18
SH3BP2	-0.4219726	8.08E-18
MTIF2	0.2472185	8.16E-18
RNF130	0.3405472	8.25E-18
RGL4	-0.1834243	8.92E-18
UBE2J1	-0.3600694	9.03E-18
PIGF	0.2685368	1.11E-17
PAIP2	-0.3672404	1.13E-17
DNAJB11	-0.2668887	1.17E-17
C1orf56	-0.4204909	1.18E-17
SH3GLB1	-0.3925231	1.29E-17
TOMM5	0.3510053	1.32E-17

DKC1	0.3325311	1.42E-17
LSM2	0.333478	1.45E-17
CDK9	-0.3401041	1.55E-17
NFKB2	-0.2744538	1.55E-17
AC027601.6	-0.3317267	1.58E-17
TUBB2A	-0.1598446	1.59E-17
SLC5A3	-0.4188395	1.60E-17
TNFAIP8L1	-0.235016	1.68E-17
APAF1	0.2370623	1.82E-17
MGA	0.2755897	1.84E-17
TP53	0.2567551	1.87E-17
IST1	-0.3427937	1.87E-17
RPL5	0.2050314	1.88E-17
ETFB	-0.3585218	2.01E-17
NCF4	0.3756405	2.02E-17
SPATA2L	-0.2028305	2.07E-17
ANKLE1	0.1139983	2.22E-17
DDIT3	-0.3319824	2.26E-17
TAPBP	-0.3550463	2.30E-17
REPIN1	-0.321182	2.38E-17
MLH3	0.248683	2.48E-17
PARN	0.1758089	2.54E-17
SGTA	-0.2776564	2.59E-17
TXNDC5	0.2391429	2.60E-17
FAM49B	-0.3066641	2.67E-17
AK2	0.3272528	2.67E-17
APOLD1	0.1770496	2.70E-17
DEDD2	-0.3720239	2.81E-17
GRASP	-0.1872928	2.82E-17
LYZ	0.3652751	2.94E-17
GRK3	0.1975886	2.98E-17
C15orf39	-0.3234058	3.19E-17
RIT1	-0.3043854	3.40E-17
RB1CC1	-0.3686699	3.56E-17
TIMMDC1	0.2925588	3.80E-17
ERH	0.3376241	3.92E-17
LRRC8A	-0.3057234	4.07E-17
TOMM7	0.3277133	4.08E-17
DEK	0.3530254	4.11E-17
PDLIM4	-0.1908432	4.22E-17
UQCC2	0.294825	4.33E-17
USP4	-0.3250888	4.50E-17
CYP7B1	0.1809007	4.65E-17
RBBP4	0.3572701	4.68E-17
SUDS3	0.2521929	4.71E-17
ARRDC3	-0.3466379	4.93E-17
RBM17	-0.3115882	5.03E-17
HSPA8	0.4183792	5.07E-17
SRC	-0.217969	5.76E-17
CCL5	-0.5567656	6.15E-17

ENTR1	0.2156918	6.56E-17
TOMM6	0.3572791	6.57E-17
THOC7	0.3350589	7.78E-17
SLC39A10	0.3032509	7.81E-17
CFAP36	0.3883647	8.05E-17
PPCDC	-0.2499983	8.10E-17
KIF9	-0.3157065	8.19E-17
MGRN1	-0.2958776	8.21E-17
COL24A1	0.3674742	8.31E-17
ATP5PF	0.3010343	8.63E-17
UNC5B	-0.2668532	8.71E-17
ZNF141	0.2964151	8.86E-17
CYB5A	0.2389748	9.79E-17
CAD	0.1606013	9.94E-17
AL627171.2	0.3474129	1.036E-16
CRTAP	0.319809	1.068E-16
PPP1R10	0.4080217	1.11E-16
RC3H1	-0.3767139	1.11E-16
MESP1	0.1192694	1.129E-16
ITGB3BP	0.2342694	1.13E-16
ME2	0.3658673	1.289E-16
PWWP3A	0.2378857	1.319E-16
TMEM131	0.2341205	1.329E-16
NDUFAF8	0.3579435	1.35E-16
ADRB1	0.2442236	1.441E-16
RNF126	-0.3111259	1.489E-16
CLEC2B	-0.4369636	1.528E-16
DUSP18	-0.2676561	1.539E-16
DUSP10	-0.3078919	1.677E-16
CIAO2A	0.2869661	1.704E-16
C11orf74	0.2834003	1.707E-16
PHF14	0.3522155	2.032E-16
FAM102B	-0.2393607	2.068E-16
IMPACT	0.1991677	2.072E-16
TRIB1	-0.3056451	2.133E-16
CAPZB	-0.2491752	2.257E-16
UBE2Q2	-0.3593331	2.279E-16
ARID4A	-0.4382921	2.36E-16
ZNF66	0.247188	2.429E-16
KCNE3	0.2296369	2.534E-16
UQCRFS1	-0.3350916	2.557E-16
RHEX	0.4012363	2.689E-16
ARMCX3	-0.4418261	2.744E-16
COA4	0.3783722	2.813E-16
NOP14	0.3239902	2.849E-16
TBCK	0.1636512	2.86E-16
TNFRSF1B	-0.2993906	2.92E-16
TESPA1	0.3395122	2.958E-16
VPS13C	0.3877718	3.038E-16
RPS21	0.1937834	3.058E-16

YWHAG	-0.3800985	3.266E-16
B3GNT8	-0.2188084	3.331E-16
PCMTD1	0.3029866	3.445E-16
SDHAF3	0.2148215	3.499E-16
CYSLTR1	0.3431927	3.752E-16
ZNF276	-0.2961648	4.026E-16
NAB2	-0.2404592	4.22E-16
ADAM8	-0.3477243	4.352E-16
BRAF	-0.3203897	4.369E-16
GPSM2	0.2111491	4.483E-16
RFXAP	0.1226111	4.513E-16
F11R	-0.3580706	4.862E-16
BRD7	0.3525557	5.854E-16
NEDD9	-0.3377711	6.009E-16
CD37	-0.3380507	6.069E-16
RPL36A	0.2062346	6.121E-16
FAM210B	0.2124634	6.33E-16
GLUL	-0.4961193	6.945E-16
CCDC6	-0.325588	7.413E-16
SIGIRR	-0.2958714	7.836E-16
HSDL2	0.2249582	7.882E-16
LIPT1	0.1654945	8.661E-16
PAM16	0.2777356	8.717E-16
PCBP1-AS1	0.2257565	8.931E-16
CARD19	-0.3983021	9.244E-16
DARS	0.3476683	9.727E-16
POLR2H	0.2952523	9.767E-16
PJA2	-0.3496701	1.049E-15
SELENOK	-0.3573414	1.137E-15
PSMA5	0.2570691	1.145E-15
RPL39	0.2189678	1.283E-15
FKBP1A	-0.367298	1.295E-15
PHACTR2	0.256041	1.344E-15
XRCC5	0.3325268	1.423E-15
AC016831.5	-0.2921893	1.5E-15
ACADSB	0.1221217	1.522E-15
SBDS	-0.3509921	1.532E-15
PPP4R1	-0.2741293	1.558E-15
NLN	0.165716	1.76E-15
ADGRE2	-0.3755013	1.829E-15
IMPA2	0.2468452	1.851E-15
SSH2	-0.3361132	1.931E-15
NEK9	0.1909076	2.034E-15
ABRAXAS1	0.2299233	2.058E-15
CREB1	0.2014817	2.089E-15
MACF1	0.4292064	2.326E-15
HDDC3	0.1916207	2.357E-15
PPP1R3E	0.1967912	2.477E-15
ALG3	0.1955853	2.728E-15
RNF149	-0.2603774	2.738E-15

ELN	-0.1488573	2.759E-15
AHSA1	0.323513	2.765E-15
DNAJC3	-0.2857325	2.943E-15
TAF12	0.2379522	3.001E-15
VPS26A	-0.2780381	3.141E-15
BTN3A2	0.1942511	3.316E-15
TLN1	0.352025	3.414E-15
IFITM2	0.3437555	3.994E-15
DNAJA2	-0.3789865	4.151E-15
RPLP2	0.1804769	4.318E-15
NMNAT3	0.1670555	4.323E-15
HRAS	-0.1865198	4.469E-15
FZR1	-0.2159936	4.587E-15
MNS1	-0.2872185	4.665E-15
MOK	-0.1784529	4.686E-15
MCPH1	0.1687759	4.721E-15
CRYL1	0.202453	4.724E-15
NFKBIZ	0.2511502	4.757E-15
SMIM26	0.3778711	5.143E-15
SLC3A2	-0.341591	5.212E-15
CD200	0.2365244	5.266E-15
FAM120A	0.3283532	5.333E-15
BIN1	-0.2526061	5.389E-15
LNPK	0.1405865	5.744E-15
SFPQ	0.2737002	5.973E-15
RAB1A	-0.3688299	6.016E-15
EHBP1L1	-0.3141069	6.092E-15
CKAP2	0.2322898	6.341E-15
R3HDM2	0.303905	6.711E-15
CYHR1	0.2586492	7.583E-15
ECHDC1	0.2505556	7.999E-15
ZCCHC7	0.2680064	8.011E-15
ZSWIM7	0.242751	8.03E-15
CALHM6	0.3776572	8.151E-15
AKIRIN1	-0.3703536	8.161E-15
TANK	-0.3928088	8.212E-15
TP53BP2	-0.3138899	8.464E-15
STX12	-0.2501942	8.481E-15
PSMB1	-0.3184142	8.631E-15
BMI1	0.2235797	8.719E-15
SLC43A2	-0.2891192	8.869E-15
AC096667.1	0.1433596	9.056E-15
GNPTAB	0.3511416	9.562E-15
ALKBH7	0.3856455	9.625E-15
CCM2	-0.3496231	1.037E-14
PKP4	0.1910447	1.054E-14
IMPDH2	0.372106	1.104E-14
LINC01578	-0.3509923	1.106E-14
ATG101	-0.232349	1.111E-14
GSN	-0.3832144	1.155E-14

TMBIM1	-0.2968108	1.171E-14
CFD	0.6040564	1.174E-14
EIF3B	0.3082769	1.356E-14
AC090559.1	0.2986701	1.361E-14
NSF	0.2146259	1.459E-14
VDAC1	0.3933058	1.522E-14
ARHGAP30	-0.2820165	1.598E-14
TRIP11	0.2756799	1.605E-14
OAZ2	-0.3138748	1.73E-14
PCID2	0.2682411	1.731E-14
EEF1AKMT4	0.1184579	1.788E-14
CTSW	-0.3692966	1.856E-14
BAZ2A	-0.332454	2.007E-14
SF3B5	-0.3101133	2.061E-14
FASTKD1	0.1751678	2.061E-14
BAALC	-0.3984577	2.084E-14
S100Z	0.3574205	2.087E-14
PPIF	-0.3534691	2.096E-14
RRP9	0.1659124	2.152E-14
TMEM189	-0.2331561	2.18E-14
TMEM192	0.201696	2.249E-14
CCNL1	0.3467992	2.344E-14
AL354733.3	0.1163882	2.429E-14
POGLUT1	0.2037607	2.48E-14
PPP4C	-0.2837206	2.651E-14
TMUB1	-0.1760518	2.694E-14
JUNB	-0.6546799	2.798E-14
NUDT7	0.1132667	2.841E-14
MCM3AP-AS	0.1534861	2.842E-14
MRPL24	0.2248693	2.878E-14
DCK	0.3176813	2.934E-14
HCG11	0.1609751	2.974E-14
COG3	-0.2831482	3.102E-14
UBE2A	-0.3286445	3.125E-14
RPS27A	0.1511003	3.132E-14
KDM5B	-0.410198	3.172E-14
TERF2	-0.294041	3.342E-14
PMS1	0.2280098	3.618E-14
SH3YL1	0.176934	3.694E-14
AGTRAP	0.2097061	3.75E-14
ZC3H8	0.2435108	3.756E-14
OTUD6B-AS	0.3433222	3.929E-14
GOLIM4	0.3228946	3.93E-14
UBAC1	0.2250298	4.13E-14
NME2	0.29367	4.154E-14
SEC14L1	-0.3098818	4.202E-14
TRABD	-0.2836819	4.627E-14
DSTYK	-0.2258238	4.647E-14
RPL21	0.1861991	4.785E-14
EBAG9	0.1998466	5.124E-14

VPS36	0.3409793	5.153E-14
FBXL3	-0.2792826	5.512E-14
GNAS	-0.3089874	5.677E-14
EEF1AKMT1	0.1228929	5.829E-14
CPSF2	0.2507088	5.875E-14
TNNI2	-0.2157031	5.985E-14
IAH1	-0.2823091	5.992E-14
KTN1	0.3234723	6.193E-14
CDC42SE2	-0.3453211	6.296E-14
GCNT1	0.2345619	6.8E-14
ATF4	-0.4024623	6.867E-14
OIP5-AS1	0.3367337	6.9E-14
HIST1H2AC	-0.4141355	6.959E-14
GTF2H5	0.2425676	7.09E-14
PCIF1	-0.2825189	7.969E-14
SOS1	0.2763129	8.107E-14
PROC	-0.2507142	8.155E-14
HOPX	0.4139576	8.53E-14
CREG1	0.3137007	8.58E-14
ELMO1	-0.3168123	8.956E-14
TNK2	-0.256205	9.046E-14
COA3	0.2601112	9.071E-14
THYN1	0.2564968	9.378E-14
ILF2	-0.3200225	9.69E-14
MITD1	0.2776292	9.717E-14
CSK	-0.286675	9.72E-14
TNFRSF18	-0.3417153	1.09E-13
OSTC	0.2178126	1.146E-13
ZC3H12A	-0.1637638	1.151E-13
ABCC4	0.2573732	1.164E-13
AL450998.2	0.1794446	1.223E-13
HIKESHI	0.257537	1.247E-13
RAP1A	-0.3326649	1.27E-13
DGKD	-0.2513538	1.304E-13
INPP4B	0.2487666	1.31E-13
SPG11	0.2337157	1.311E-13
TMEM218	0.2014185	1.332E-13
C12orf45	0.2943909	1.347E-13
SMIM8	0.18665	1.357E-13
SREBF1	-0.2266553	1.382E-13
STX17	0.2139809	1.396E-13
TCP11L2	-0.3020984	1.396E-13
POLR3H	0.166532	1.42E-13
PMVK	0.2106124	1.446E-13
PAFAH1B1	-0.3127339	1.488E-13
TBC1D5	0.2677823	1.513E-13
ZSWIM6	-0.2656472	1.586E-13
NOC3L	0.258719	1.593E-13
PSMD7	-0.2378582	1.767E-13
ACBD3	-0.2956895	1.783E-13

PSMG4	0.2735729	1.802E-13
NDUFA8	0.2079578	1.817E-13
AATK	-0.1419569	1.865E-13
SOCS2	0.300273	1.888E-13
DLEU1	0.2597595	1.924E-13
SERPINB1	0.34765	1.948E-13
DYNC1H1	-0.281893	1.961E-13
RPGR	-0.3437669	1.961E-13
CHCHD7	0.2746527	1.963E-13
TMC6	-0.3175302	2.098E-13
CDV3	0.2871176	2.203E-13
ITGA4	0.4042556	2.215E-13
ALAS1	-0.2764578	2.24E-13
CLK1	-0.3830135	2.284E-13
NDUFA3	0.2657368	2.343E-13
CLPTM1L	0.2023603	2.393E-13
IRF9	-0.2311689	2.44E-13
PAFAH1B2	-0.3406115	2.484E-13
NFATC2	-0.25511	2.486E-13
UBE3D	0.1268604	2.521E-13
PGM2L1	-0.3352276	2.553E-13
PTBP2	-0.2633899	2.566E-13
NUP42	0.265382	2.696E-13
TRUB2	0.1874819	2.729E-13
GARS-DT	0.2841331	2.757E-13
PSMG1	0.2138835	2.818E-13
RNF168	-0.3659027	3.085E-13
ARHGEF6	0.2821595	3.213E-13
SKA2	0.2102613	3.463E-13
AL359504.2	0.1229828	3.497E-13
SPOP	-0.3163424	3.643E-13
PUS7	0.1881535	3.689E-13
IFNGR2	-0.3655233	3.863E-13
VNN1	0.3209641	4.05E-13
DENND3	-0.2907744	4.14E-13
ABHD14A	0.2331259	4.143E-13
LATS2	-0.2490254	4.337E-13
ZNF253	0.1738128	4.615E-13
LZIC	0.2185456	4.676E-13
ARV1	0.1311055	4.727E-13
AGPAT2	-0.2954245	4.902E-13
CIITA	0.2592258	4.933E-13
CACUL1	-0.2478108	4.964E-13
CMC1	0.2179648	5.165E-13
SNCA	0.2581317	5.247E-13
KLHDC8B	0.1303633	5.412E-13
VEZF1	-0.2830408	5.55E-13
NDUFB2	0.2542602	5.662E-13
MAP3K2	-0.2818558	5.75E-13
UBA6	-0.2777324	5.972E-13

AGL	0.1748204	6.134E-13
ANKRD13D	-0.2090679	6.438E-13
PLAC8	0.4239892	6.692E-13
PPP6C	-0.2394463	6.746E-13
SATB1-AS1	0.2998224	6.75E-13
ZNF681	0.1569077	6.928E-13
ARPC1A	-0.2488457	6.998E-13
PPP3R1	-0.3404365	7.431E-13
ACTR1A	-0.266004	7.495E-13
ZBTB46	-0.1969409	7.679E-13
CCL1	-0.1909476	7.845E-13
SLC2A1	-0.2767395	7.876E-13
DBP	0.157998	7.889E-13
N4BP1	-0.2992175	7.986E-13
AL133330.1	-0.1630457	8.15E-13
TPR	0.3267108	8.399E-13
EIF4G3	0.244842	8.439E-13
WBP11	-0.286942	8.772E-13
XPO1	0.313428	8.834E-13
MIR155HG	0.250986	8.962E-13
TCAIM	0.1540071	9.303E-13
STX11	-0.2164386	9.333E-13
GART	0.2554073	9.371E-13
AP1G1	-0.2971685	9.56E-13
PCYOX1	0.1972073	9.718E-13
EMB	0.3829161	9.99E-13
ACTB	-0.3149714	1.004E-12
MANF	-0.3929346	1.02E-12
TRADD	0.1659403	1.039E-12
PIGBOS1	0.1876362	1.046E-12
ARHGAP4	-0.3029963	1.088E-12
RPL24	0.1855889	1.13E-12
RNF20	0.1692082	1.148E-12
RASGRP3	0.2672623	1.156E-12
ADK	0.1990442	1.156E-12
ADPRHL2	-0.2304555	1.165E-12
AKAP1	0.1417309	1.176E-12
STXBP4	0.1132329	1.196E-12
CLNS1A	0.3417519	1.204E-12
TINF2	-0.2283326	1.218E-12
BANF1	0.2589946	1.292E-12
SGK3	0.2458177	1.292E-12
SMIM19	0.228699	1.296E-12
PTPN2	0.2901883	1.311E-12
THOC2	0.3578631	1.328E-12
ZNF780B	0.1817711	1.342E-12
IMP3	0.3248247	1.351E-12
BUD31	-0.3351422	1.401E-12
SV2A	0.1424199	1.412E-12
ZC3H15	-0.3549967	1.506E-12

AL499604.1	0.1797117	1.514E-12
GNPDA1	0.1616494	1.515E-12
ROCK2	0.307619	1.518E-12
PLPPR3	0.2415993	1.623E-12
GCH1	-0.2863598	1.629E-12
CD7	-0.3017283	1.654E-12
PRDX5	-0.3241902	1.663E-12
ZNF248	0.1453319	1.737E-12
SMARCA5	0.3316633	1.818E-12
CCDC34	0.1766435	1.826E-12
MDK	-0.3928551	1.901E-12
FLYWCH2	0.1994653	1.93E-12
ATP5MC1	0.3064293	1.937E-12
PPIB	-0.3359285	1.998E-12
KRT17	-0.4246644	2.009E-12
LENG8	-0.2652532	2.084E-12
ANXA2	-0.386653	2.095E-12
BCL11A	0.4618399	2.099E-12
NR2F6	0.1243398	2.139E-12
RECQL	0.2272617	2.188E-12
UBE2L3	-0.2670409	2.25E-12
TALDO1	0.2455574	2.271E-12
AKAP8L	-0.2913619	2.272E-12
KHNYN	-0.2383969	2.318E-12
MSH2	0.2279008	2.328E-12
COL4A5	0.1923452	2.366E-12
MIB1	0.2486463	2.487E-12
TTC37	0.2732986	2.518E-12
VPS41	0.2422996	2.529E-12
BCLAF1	0.2866394	2.584E-12
LRRK1	0.191563	2.584E-12
CSGALNAC	-0.2933429	2.755E-12
EAPP	-0.2950841	2.766E-12
XIRP2	0.1778389	2.797E-12
SRP72	0.3096732	2.798E-12
NDUFV3	0.2493167	2.809E-12
CLDND1	-0.2977825	2.864E-12
NPEPPS	-0.3829873	2.979E-12
RABEPK	0.146636	3.023E-12
GMPR2	0.1948788	3.174E-12
SGO2	0.2049141	3.254E-12
SH3GL1	-0.2155841	3.297E-12
SLITRK4	0.1649024	3.419E-12
ATIC	0.2686235	3.473E-12
TCEAL4	0.2515711	3.609E-12
CCND3	-0.3362803	3.618E-12
RNF114	-0.2824296	3.717E-12
ANKRD28	-0.396865	3.808E-12
SRBD1	0.2284553	3.843E-12
ALDH3A2	0.1068085	3.883E-12

RNASE3	0.1099587	4.105E-12
RASSF5	-0.2324262	4.302E-12
ATXN7L1	0.1134687	4.358E-12
S100A4	-0.6372423	4.397E-12
ZBTB38	0.1081293	4.512E-12
ARL5A	0.2479442	4.731E-12
ZEB1	-0.3208037	4.795E-12
R3HDM1	0.2111396	4.897E-12
CKS2	0.2060567	4.961E-12
PRDM16	0.1710744	5.107E-12
LINC00665	0.2437834	5.115E-12
PPM1K	0.213276	5.14E-12
DNMT1	0.303548	5.177E-12
ST6GAL1	0.0823115	5.25E-12
EGLN2	-0.2404503	5.557E-12
FRY	0.1256105	5.809E-12
NMI	0.3158874	5.929E-12
ALDH3B1	0.2300141	5.966E-12
POLR3GL	0.2806892	6.027E-12
ORMDL2	-0.2455119	6.414E-12
CPTP	-0.1602506	6.5E-12
UTP18	0.264446	6.553E-12
SMC1A	0.298815	6.976E-12
NSRP1	0.3080173	7.226E-12
RNF145	-0.3105225	7.417E-12
OCLN	0.2119358	7.78E-12
RPL22L1	0.3776643	7.8E-12
DYRK1B	-0.2209072	7.944E-12
C1GALT1C1	0.177827	8.069E-12
RIDA	0.1434256	8.348E-12
UBE2M	0.2617469	8.567E-12
RRAGA	-0.1940869	8.776E-12
AHR	-0.3868904	8.91E-12
TCOF1	0.2452219	9.162E-12
SMIM30	0.1781462	9.269E-12
ABI3	-0.1142143	9.451E-12
HIBADH	0.1373331	9.515E-12
CDK4	0.2936784	9.749E-12
SLC10A3	-0.1785023	9.772E-12
PAICS	0.2746375	9.83E-12
CPNE3	0.3045036	9.965E-12
ZEB2	0.293197	1.004E-11
UXS1	0.2194509	1.008E-11
FBXW11	-0.2095644	1.015E-11
GDF11	0.2486823	1.017E-11
UBR5-AS1	0.2609897	1.032E-11
RNF214	0.1705387	1.057E-11
UBE2Z	-0.282711	1.139E-11
FAM76A	-0.2091813	1.192E-11
DPM1	-0.202435	1.224E-11

PCBP2	0.2702832	1.269E-11
RNMT	-0.317321	1.313E-11
TNFSF14	-0.2279171	1.343E-11
NUDT6	0.1355458	1.356E-11
MAP3K4	0.158332	1.36E-11
KAT6A	-0.2998141	1.375E-11
RHEB	-0.3280623	1.386E-11
SLC30A1	-0.2348607	1.392E-11
TARSL2	0.2003538	1.402E-11
SLC43A1	0.1354929	1.421E-11
HMGCS1	-0.303874	1.445E-11
PPWD1	0.1973452	1.516E-11
NOTCH3	-0.1522106	1.516E-11
DNAJC19	0.3003146	1.565E-11
NFKB1	-0.3087593	1.586E-11
RSAD1	0.1289753	1.632E-11
ELF4	-0.2188405	1.632E-11
RYBP	-0.3404254	1.646E-11
ACTR1B	-0.2778638	1.678E-11
AC019205.1	0.0749992	1.711E-11
COX4I1	-0.271469	1.827E-11
PBDC1	-0.2846209	1.857E-11
IDI1	-0.3229219	1.88E-11
ALDH1B1	0.0811845	1.903E-11
BCKDHB	0.11322	1.904E-11
CEBPB	-0.3704952	1.94E-11
YPEL3	0.3417984	1.94E-11
MMACHC	0.067942	1.952E-11
NT5C	-0.3131261	2.015E-11
PDCD6	0.2434253	2.114E-11
NHP2	0.3268414	2.169E-11
NEAT1	-0.4831507	2.175E-11
PNPT1	0.2242567	2.263E-11
EIF3E	0.2842428	2.284E-11
IL16	0.2197465	2.304E-11
LRIF1	0.2209501	2.325E-11
LINC00899	0.3046463	2.344E-11
GOPC	0.2111144	2.433E-11
NFYB	0.1977834	2.461E-11
MMP19	-0.2030603	2.483E-11
ZNF326	-0.2886368	2.493E-11
ADD3	0.2389884	2.501E-11
CIR1	-0.3528789	2.595E-11
TRIM16	0.2273693	2.619E-11
DEAF1	0.2179028	2.621E-11
BCOR	-0.3286024	2.621E-11
ACAT1	0.1633707	2.688E-11
ERMP1	0.1960726	2.758E-11
BDH2	0.1137448	2.785E-11
ACTL6A	0.2304834	2.809E-11

PRR12	0.1854009	2.831E-11
MID1IP1	-0.2671152	2.873E-11
ARMC10	0.2268743	2.955E-11
PDXP	0.0997397	2.961E-11
ZNFX1	-0.2871689	3.02E-11
RPUSD3	0.1663748	3.032E-11
ACOT13	0.2488429	3.161E-11
PCMTD2	0.1920188	3.198E-11
CARNMT1	0.1825893	3.214E-11
SRP14-AS1	0.1850806	3.219E-11
FES	0.1928925	3.224E-11
SLC16A7	0.240334	3.417E-11
CLPB	0.1450398	3.467E-11
SLC35E1	-0.203197	3.523E-11
TOR4A	-0.1973532	3.621E-11
MAPKBP1	-0.2237256	3.782E-11
RNF7	-0.272436	3.795E-11
BLOC1S2	-0.2877328	3.86E-11
STK17B	-0.3481988	3.873E-11
PPTC7	-0.1948735	3.946E-11
PRH1	0.1759016	4.035E-11
RPF2	0.1779273	4.144E-11
POLR2F	0.2896197	4.19E-11
NBAS	0.1564161	4.247E-11
S1PR2	-0.104311	4.308E-11
CNPY2	0.2758428	4.363E-11
DUSP2	0.2857886	4.497E-11
CNNM3	0.0932098	4.514E-11
ARFGAP3	-0.2311265	4.655E-11
RNF213	0.24281	4.664E-11
ENTPD1-AS	0.1770313	4.851E-11
SELENOT	-0.2906887	4.958E-11
SELENOW	-0.2223001	5.005E-11
SOCS3	-0.2547573	5.238E-11
GMPR	0.1807482	5.365E-11
PPA1	0.2970998	5.731E-11
NIFK	0.2777546	5.966E-11
TOX4	-0.2759947	6.002E-11
WRNIP1	0.1949258	6.029E-11
FBL	0.3282788	6.234E-11
WEE1	-0.2315745	6.663E-11
TST	0.1973623	7.059E-11
SASH3	-0.2401188	7.171E-11
MTR	0.2875989	7.358E-11
RBM27	-0.2466104	7.366E-11
C20orf27	0.188746	7.553E-11
ADAL	0.1011933	7.632E-11
KCMF1	-0.304577	7.941E-11
MLEC	0.287683	8.042E-11
TIMP2	0.2455314	8.104E-11

ELP4	0.1230552	8.132E-11
LIMD1	-0.158594	8.17E-11
TNFRSF4	-0.4375919	8.175E-11
CD68	-0.232077	8.257E-11
SSR1	0.2525678	8.295E-11
MED13L	-0.3069563	8.38E-11
CTR9	0.2748548	8.948E-11
CHIC2	-0.2944492	9.173E-11
DUT	0.2803905	9.213E-11
NUMB	-0.2401958	9.315E-11
HEATR5B	-0.193025	9.346E-11
RAB5C	-0.2797172	9.611E-11
SLC22A16	0.1498823	9.827E-11
RNASEL	0.1100408	1.005E-10
BAIAP2-DT	-0.2417821	1.06E-10
EIF4H	-0.3335639	1.068E-10
ERLIN1	0.2489148	1.075E-10
ZNF74	0.0865286	1.095E-10
YPEL2	-0.2872454	1.117E-10
MRTO4	0.2082272	1.119E-10
GLUD1	-0.2742431	1.124E-10
SORD	0.2125199	1.131E-10
CPNE8	0.1885245	1.136E-10
LINC00909	0.1978045	1.146E-10
SOD2	-0.4694238	1.167E-10
MYBBP1A	0.1312574	1.183E-10
SMAD5	0.1748929	1.218E-10
FAM89B	-0.2568481	1.235E-10
CDCA7L	0.2409246	1.265E-10
GCSAML	0.2862361	1.28E-10
SDC4	-0.1669029	1.342E-10
ITPRID2	-0.32705	1.37E-10
SLA	0.1035488	1.377E-10
ZNF32	0.1492986	1.4E-10
NME4	0.2263399	1.461E-10
SNHG15	-0.2966313	1.472E-10
CLEC2D	0.1527662	1.517E-10
TOMM20	0.305562	1.547E-10
OARD1	0.2258584	1.561E-10
AZU1	0.5047002	1.574E-10
PLAU	-0.3421229	1.593E-10
JCHAIN	-0.1843023	1.596E-10
MAPRE1	-0.2999552	1.599E-10
PAPOLA	-0.2601473	1.602E-10
MAP3K1	-0.2810036	1.635E-10
NT5DC1	0.151448	1.639E-10
SPATC1L	0.1783657	1.655E-10
TARDBP	-0.2840871	1.677E-10
MSH3	0.1825977	1.71E-10
ATRN	0.1220089	1.738E-10

TSEN2	0.1375026	1.761E-10
ATP1A1-AS1	0.0727452	1.766E-10
ARHGEF2	-0.3276115	1.766E-10
SAMD4B	-0.2325752	1.799E-10
AAMDC	0.1550183	1.808E-10
ZNHIT6	0.2648778	1.81E-10
DDX55	0.1850163	1.894E-10
PPCS	0.2945128	1.926E-10
TLR4	0.0767765	1.95E-10
MRPL19	0.2322205	2.016E-10
DNTTIP1	-0.1893106	2.05E-10
CHCHD3	0.1728735	2.077E-10
SSU72	0.2524891	2.131E-10
CCDC144A	0.1849575	2.168E-10
SERTAD3	-0.2068054	2.204E-10
RPL41	0.1386464	2.23E-10
TMED3	0.2257041	2.251E-10
RPL11	0.140873	2.325E-10
CENPX	0.224339	2.354E-10
AC026471.1	0.1096809	2.372E-10
NDC1	0.0886044	2.5E-10
ZNF516	-0.2462819	2.502E-10
LY75	0.2786659	2.536E-10
SNN	-0.1559591	2.552E-10
MRE11	0.2415068	2.553E-10
PPP3CA	0.2353749	2.566E-10
RTCA-AS1	0.2219861	2.592E-10
BRCC3	0.1666641	2.639E-10
SETD5	-0.2234196	2.656E-10
B4GALT6	0.1110812	2.668E-10
FAM111A	0.2502656	2.689E-10
GALK1	0.1483978	2.74E-10
COPS2	-0.2834303	2.904E-10
TRPV2	-0.1240817	3.142E-10
KIF1BP	-0.1906471	3.144E-10
MTLN	0.1389662	3.173E-10
PGRMC2	0.2349824	3.19E-10
SHOC2	-0.3487045	3.203E-10
FAH	0.2172392	3.224E-10
PVT1	0.1241479	3.281E-10
PARK7	0.2799379	3.283E-10
ZBTB20	0.2875064	3.288E-10
CEP57	0.2342979	3.301E-10
ERVK3-1	-0.1970213	3.348E-10
RICTOR	-0.3245155	3.391E-10
GSEC	0.1119089	3.403E-10
PRKAA1	-0.3308964	3.466E-10
CLK3	-0.2201671	3.513E-10
INTS13	0.1917777	3.687E-10
BBC3	-0.1034751	3.694E-10

ZMYND19	0.0962102	3.707E-10
MDFI	0.1552521	3.772E-10
INTS10	0.2356858	3.78E-10
MRPL1	0.1808051	3.807E-10
AC016831.1	-0.1633907	3.841E-10
GPBP1	-0.2255809	3.897E-10
RELA	-0.2431445	4.059E-10
GLIPR1	0.5364934	4.165E-10
CAPRIN1	0.2504831	4.238E-10
EVI2A	0.2813223	4.247E-10
FADS1	0.2685061	4.34E-10
SMARCAD1	0.189602	4.378E-10
RIOX2	0.1928149	4.6E-10
SCARB1	0.1484694	4.612E-10
LINC00996	0.1536751	4.854E-10
TSSC4	-0.1460937	4.859E-10
GABARAPL1	-0.1954528	5.036E-10
RFLNB	0.2542849	5.089E-10
ALPK1	0.1456058	5.159E-10
RAB2A	-0.2948323	5.255E-10
CHEK2	0.1099771	5.474E-10
CRYGD	0.2861977	5.546E-10
ZYX	-0.3006459	5.581E-10
YY1AP1	-0.2099105	5.624E-10
ZNF770	0.199794	5.638E-10
CCDC18	0.1629259	5.801E-10
TLR2	0.1372013	6.18E-10
ARID1B	0.326376	6.227E-10
IDE	0.1110905	6.326E-10
LIMA1	0.1450614	6.333E-10
PTPN22	0.2002002	6.502E-10
UPF2	0.3516269	6.624E-10
HIST1H2BC	-0.336176	6.63E-10
TIMM8B	0.2358166	6.779E-10
VIM-AS1	-0.2325129	6.849E-10
PAF1	-0.1970306	6.861E-10
ZC3H6	0.1850654	7.277E-10
FRA10AC1	0.1710584	7.291E-10
JARID2	-0.2494694	7.383E-10
FOXO3	-0.2521645	7.441E-10
ZNF667	-0.2738686	7.562E-10
FAM207A	0.1916038	7.689E-10
UBL5	0.1893652	7.744E-10
ERCC6L2	0.1666927	7.82E-10
CALCRL	-0.2533387	7.913E-10
RPS8	0.135565	8.31E-10
DCTPP1	0.2332604	8.414E-10
EMD	-0.2406156	8.456E-10
ORMDL1	0.2310661	8.521E-10
CUL1	0.1478133	8.569E-10

RBBP6	0.3081329	8.735E-10
CSPP1	-0.2696818	8.955E-10
EMC2	0.1530958	9.019E-10
RASA2	-0.2291337	9.606E-10
CALHM2	-0.1728707	1.043E-09
SHFL	-0.2557538	1.077E-09
TMEM123	0.1970038	1.084E-09
RTN2	0.1209287	1.088E-09
RAB32	0.3545762	1.091E-09
PRPSAP1	0.1554253	1.106E-09
SNHG1	0.2131427	1.108E-09
LAMTOR4	0.2299943	1.113E-09
UNG	0.151321	1.152E-09
ADGRA3	0.0732116	1.152E-09
COMMD4	0.1812294	1.177E-09
CEP41	0.1477677	1.18E-09
Z83843.1	0.1835995	1.187E-09
PHTF1	-0.188241	1.192E-09
MRPS24	0.2459588	1.194E-09
NT5M	0.1670641	1.206E-09
HIST1H1D	0.4642936	1.213E-09
SERPINB6	0.233187	1.216E-09
SMPD2	-0.1719257	1.23E-09
COPRS	0.192717	1.23E-09
UQCRH	0.189995	1.234E-09
TMEM258	0.2488568	1.239E-09
CHST13	0.1009841	1.247E-09
PML	-0.1481122	1.27E-09
FUCA2	0.1979427	1.285E-09
CAPG	0.2349596	1.315E-09
HADH	0.2500937	1.345E-09
LAMTOR1	0.2704464	1.372E-09
NUTF2	-0.2086707	1.405E-09
ENOSF1	0.2443439	1.427E-09
LCOR	-0.3060887	1.47E-09
ZNF680	0.1520735	1.478E-09
LACTB	-0.2760441	1.482E-09
LINC01184	0.1683631	1.487E-09
CABIN1	-0.3049724	1.533E-09
MPLKIP	0.204026	1.538E-09
TUBGCP4	0.1721682	1.589E-09
RABGEF1	-0.2273336	1.602E-09
CTNND1	-0.2561439	1.612E-09
WDR89	0.1167752	1.624E-09
LINC02256	0.2124183	1.658E-09
IL18	0.3277505	1.706E-09
SYNE3	-0.299028	1.778E-09
TCAF1	0.2232046	1.83E-09
HILPDA	0.1383488	1.88E-09
DHPS	0.1554193	1.89E-09

TSPAN3	0.2495654	1.95E-09
ARHGAP15	0.24072	2.019E-09
ST3GAL3	0.1121354	2.042E-09
B4GALT1	-0.1966744	2.053E-09
VEZT	-0.2208475	2.068E-09
ARHGEF3	-0.1510499	2.071E-09
ULK4	-0.1629036	2.084E-09
ZNF546	0.0815668	2.093E-09
AC092683.1	0.2842811	2.131E-09
ECI2	0.1586708	2.3E-09
STARD3NL	-0.1841149	2.302E-09
ZNF37A	0.2597857	2.333E-09
MYL12A	-0.2450249	2.34E-09
NFAT5	-0.2259414	2.341E-09
GPR35	-0.1745197	2.461E-09
RASSF2	-0.2265078	2.498E-09
NUDT16L1	-0.3405884	2.524E-09
TAPT1	-0.2334733	2.542E-09
REXO4	0.1803815	2.603E-09
TRIM44	0.2802965	2.651E-09
GLRX	-0.0072122	2.694E-09
TESK2	0.0614927	2.695E-09
ATP2B4	0.1758338	2.711E-09
HBD	0.866736	2.731E-09
WIPI2	-0.1841569	2.74E-09
TRIM28	0.1822072	2.773E-09
TLNRD1	-0.1781633	2.784E-09
MTX2	0.1486844	2.87E-09
UTP14A	0.2165891	2.925E-09
PNPLA8	-0.2845245	2.937E-09
USP28	0.078778	2.957E-09
CCDC86	0.0711114	2.959E-09
GCA	0.2257062	2.999E-09
TOMM40	0.1893493	3.065E-09
CYB5R3	-0.2246047	3.148E-09
CD2BP2	-0.2025362	3.405E-09
TMEM168	0.1515918	3.407E-09
CCT3	0.3000935	3.419E-09
PTBP3	0.2328522	3.605E-09
HSD17B12	0.229657	3.613E-09
NR6A1	0.1778008	3.648E-09
CD46	0.219203	3.693E-09
LINC01409	0.1135084	3.811E-09
NARS2	0.1438797	3.881E-09
POU2F2	-0.2635573	3.882E-09
CAMLG	0.3466064	4.046E-09
NCK1	-0.2104696	4.059E-09
PRKAR1A	-0.2415855	4.15E-09
mars-02	0.1056967	4.15E-09
SF3B3	0.2065909	4.201E-09

TPCN1	0.0956918	4.222E-09
PLIN3	-0.2287982	4.272E-09
GTF2H3	0.1562826	4.274E-09
ATP5MC2	0.174042	4.275E-09
NLRC3	-0.2468527	4.28E-09
SAR1A	-0.2510245	4.29E-09
SYT11	-0.1934141	4.305E-09
ITGA3	-0.0910552	4.346E-09
ATP5ME	0.2803275	4.383E-09
RNASEH2C	0.2730843	4.41E-09
ZNF518A	0.2076902	4.513E-09
GSPT2	0.1502354	4.516E-09
C22orf34	0.1153013	4.823E-09
AC021097.2	0.0539957	4.825E-09
RALY	-0.2196373	4.87E-09
LNPEP	0.2115877	4.881E-09
SKI	-0.2104098	4.897E-09
CRTC2	-0.1741831	4.98E-09
GNA13	-0.2737629	4.98E-09
DUS1L	0.1992879	5.017E-09
MINCR	0.116292	5.145E-09
PLA2G12A	0.1364923	5.159E-09
KLHL23	0.1640705	5.251E-09
BRI3BP	0.2763482	5.328E-09
AMZ1	-0.1051997	5.364E-09
ARL11	0.2323382	5.383E-09
SMIM1	0.1194618	5.451E-09
ZNF718	0.085694	5.491E-09
ITGB1	0.1917231	5.494E-09
TRMT13	0.2265017	5.602E-09
PAAF1	0.1691357	5.627E-09
AC019069.1	0.0971403	5.648E-09
BICRAL	0.0990862	5.712E-09
CEP170	-0.2664124	5.718E-09
GRPEL1	0.1173956	5.75E-09
DIMT1	0.1765984	5.786E-09
COG6	0.0866654	5.878E-09
CAVIN1	-0.146043	6.147E-09
SRPRB	0.154252	6.182E-09
CORO1A	-0.3179143	6.322E-09
CDC123	-0.2284883	6.73E-09
GCFC2	0.0973414	6.919E-09
PMEPA1	-0.1923737	6.955E-09
MANEA	0.1362787	7.298E-09
NCK1-DT	0.1414336	7.309E-09
ICE2	0.1717109	7.461E-09
AC012146.1	0.1389388	7.663E-09
CCN3	-0.2432701	7.806E-09
TWISTNB	0.1242096	8.152E-09
CAP1	-0.2463076	8.161E-09

GPR146	-0.2420947	8.308E-09
TGIF1	-0.178862	8.583E-09
GOLGA8R	0.1390722	8.733E-09
STRBP	0.2038779	8.749E-09
PIGP	0.2406829	8.991E-09
RCAN1	0.0641145	9.203E-09
RPS6KA5	-0.3251834	9.364E-09
PDCD6IP	0.2273438	9.79E-09
GTF2H2C	0.0981308	9.822E-09
KMT2C	-0.1985833	9.878E-09
DOCK2	0.2660972	9.926E-09
MICOS13	0.2938388	1.002E-08
C5orf63	0.1029998	1.005E-08
PLGRKT	0.1296526	1.007E-08
TEX30	-0.2233732	1.033E-08
LIMK1	-0.1458877	1.054E-08
FOXRED2	0.0755192	1.064E-08
IFRD2	0.2212427	1.099E-08
FBXL12	-0.1646833	1.102E-08
JOSD1	-0.244563	1.118E-08
RPL27A	0.1563176	1.157E-08
PIP4P1	-0.1914008	1.159E-08
BDH1	0.1143922	1.196E-08
BIN3	-0.1854402	1.213E-08
SAP30BP	-0.2376706	1.223E-08
P4HA1	0.1780729	1.238E-08
SMAP2	-0.2313492	1.244E-08
PRPF6	0.2111733	1.253E-08
AC009061.2	0.1046597	1.28E-08
AL021368.2	0.2038654	1.282E-08
ARRB2	-0.1948434	1.286E-08
ZNRF2	0.1077029	1.293E-08
NDUFB6	0.2439011	1.298E-08
EPHA1-AS1	0.1216366	1.308E-08
RBM39	-0.262567	1.338E-08
FMC1	0.1764588	1.38E-08
GLYR1	0.1728387	1.396E-08
GAB2	-0.1476313	1.405E-08
DPY30	0.2001906	1.414E-08
CCDC14	0.1798172	1.448E-08
STARD4-AS	0.1420914	1.462E-08
CYSTM1	-0.2265782	1.466E-08
HSF1	-0.1347653	1.492E-08
SPINT2	-0.251311	1.521E-08
TMEM41B	-0.2152676	1.559E-08
UBQLN1	-0.247874	1.579E-08
NASP	0.3196372	1.592E-08
VRK2	0.1582828	1.678E-08
INKA1	-0.2275549	1.685E-08
PPP1R35	-0.198206	1.7E-08

TMSB4X	-0.2742747	1.804E-08
FAM118A	-0.1802294	1.811E-08
LASP1	-0.2288679	1.812E-08
KCNC4	0.1162496	1.838E-08
TAF9	-0.4003154	1.98E-08
CCDC88C	-0.1977622	1.983E-08
CEP78	0.1700593	2.037E-08
MAN1A2	0.2368292	2.055E-08
AKAP7	0.1668331	2.063E-08
NSD1	0.1772984	2.072E-08
NCF1	-0.1922068	2.107E-08
CCNI	-0.2803041	2.109E-08
FAM136A	0.2388778	2.125E-08
AMD1	-0.2853207	2.139E-08
ABCG1	-0.2135686	2.156E-08
B4GALT5	-0.177412	2.158E-08
PBXIP1	-0.2721245	2.159E-08
EMC8	-0.1646861	2.249E-08
ATXN2	-0.2998299	2.416E-08
GEMIN5	0.1100123	2.455E-08
MIR4435-2H	-0.185784	2.46E-08
NHLRC2	0.2107737	2.48E-08
RPAIN	0.1998366	2.49E-08
DYNC1LI1	-0.2393999	2.538E-08
Z99127.4	0.0788668	2.563E-08
RP2	-0.1940359	2.573E-08
HSPH1	0.2338208	2.578E-08
HECTD1	-0.193696	2.603E-08
ANKRD16	0.1306127	2.618E-08
CHMP1A	-0.1834834	2.62E-08
RREB1	-0.2908267	2.622E-08
LTA4H	0.2250516	2.736E-08
MKKS	0.1610282	2.737E-08
METTL7A	0.1478089	2.761E-08
BICD1	0.1341261	2.82E-08
ZFAND6	-0.2561531	2.829E-08
ZNF664	0.125878	2.896E-08
UBAP1	-0.1892088	2.926E-08
GRK6	-0.1825396	2.942E-08
FKBP8	-0.2504657	2.98E-08
CNIH4	-0.1863066	3.005E-08
ATG4D	-0.1363477	3.011E-08
KIF5B	-0.2538617	3.028E-08
S1PR4	-0.1627793	3.111E-08
ARIH1	-0.2179934	3.175E-08
FAM162A	0.1870153	3.381E-08
CYTH2	-0.1959044	3.398E-08
NCKAP5L	-0.1566117	3.404E-08
RPS16	0.1694958	3.406E-08
SRRM1	0.2558946	3.415E-08

DPY19L2	0.1266943	3.458E-08
COX6B1	0.2288207	3.508E-08
TM9SF2	0.228081	3.519E-08
EMC7	-0.1842347	3.525E-08
MRPS9	0.1777943	3.529E-08
DTD2	0.119814	3.541E-08
IFT27	0.1331981	3.561E-08
HAGHL	0.1312699	3.573E-08
MORF4L1	-0.2535122	3.681E-08
MAFG	-0.2313616	3.789E-08
GOLGA8N	0.1659469	3.8E-08
RHBDF2	-0.2140639	3.837E-08
RNF138	-0.1914161	3.864E-08
LACTB2	0.1260826	3.896E-08
RMND1	0.1449652	3.969E-08
MTRNR2L12	0.300964	4.026E-08
ZNF551	0.0541321	4.035E-08
METRN	0.1295507	4.102E-08
PKD2	0.1723636	4.201E-08
PIGK	0.1649283	4.246E-08
ESF1	0.2344146	4.323E-08
ATP8B2	-0.1998997	4.337E-08
ZNF485	0.0636036	4.374E-08
WDR70	0.2276807	4.417E-08
STAMBP	0.1319628	4.601E-08
MBD2	0.1865865	4.619E-08
NFE2L1	-0.2356711	4.648E-08
PKN2	-0.2175022	4.807E-08
MXD4	-0.2721048	5.015E-08
CORO7	-0.1522294	5.019E-08
AHI1	0.1724688	5.038E-08
FAF2	-0.1571966	5.183E-08
DYRK1A	-0.2558048	5.234E-08
KDM2A	-0.2230744	5.239E-08
NEFH	0.0844391	5.306E-08
COQ5	0.1197222	5.331E-08
PDCD2	0.2785906	5.334E-08
SEH1L	0.150508	5.458E-08
DUSP3	0.2161002	5.505E-08
WDR7	0.0864841	5.549E-08
CSNK2B	-0.2481303	5.608E-08
SHKBP1	-0.182012	5.684E-08
ST6GALNAC	-0.1474721	5.721E-08
ATP5F1D	0.2083998	5.866E-08
IARS	0.2299931	5.872E-08
ZNF181	0.1162614	5.935E-08
SPAG16	0.1065538	5.942E-08
PPIP5K2	0.2646082	6.055E-08
NUDCD2	0.2347941	6.057E-08
BRIX1	0.1877754	6.067E-08

RBX1	0.2196322	6.077E-08
ACO1	0.1144391	6.104E-08
DNAH14	0.136274	6.195E-08
SUGT1	0.171471	6.214E-08
AC015813.1	0.1396083	6.215E-08
DPH7	0.124233	6.382E-08
GNRH1	-0.1065046	6.491E-08
KPNA6	-0.1864426	6.573E-08
NDUFS6	0.2922165	6.675E-08
ADD1	-0.2794876	6.738E-08
SLC17A5	-0.2139503	6.776E-08
DEDD	-0.1608114	6.835E-08
KRT18	-0.391762	6.848E-08
FSD1L	-0.1843386	6.867E-08
AC103702.1	-0.2187072	7.095E-08
LIME1	-0.2009265	7.12E-08
PSMA4	0.245795	7.396E-08
NDUFV2	-0.2415212	7.603E-08
ISCU	0.3422796	7.614E-08
RPS15A	0.1361959	7.619E-08
UBASH3B	-0.2038598	7.709E-08
CLEC11A	0.2179415	7.836E-08
DNAAF2	0.1843121	8.13E-08
FAM89A	0.1601755	8.187E-08
RBM26-AS1	0.0706168	8.196E-08
DCAF12	0.1253332	8.288E-08
SNW1	-0.2195456	8.352E-08
EIF2B2	-0.2195971	8.371E-08
DMTF1	-0.2126481	8.383E-08
RANBP2	-0.2688135	8.445E-08
FBXO11	-0.2776679	8.686E-08
POLR1B	0.1134923	8.754E-08
AC108463.3	0.0694043	8.763E-08
CDYL	0.1524275	8.951E-08
PDCD4	0.2704302	9.271E-08
AC147067.1	0.0955687	9.336E-08
NEDD4	0.1135239	9.504E-08
CNST	-0.3310159	9.812E-08
PHF7	0.1621381	9.948E-08
TTF2	0.2454322	1E-07
MED10	-0.2119503	1.005E-07
RPL18	0.1401101	1.009E-07
PAXIP1	0.0536073	1.013E-07
MAT2B	0.1486707	1.02E-07
ATP6V0D1	-0.1923859	1.03E-07
CBLB	-0.195452	1.041E-07
PPP4R2	-0.2798609	1.067E-07
ACE	-0.1339218	1.068E-07
MOSMO	0.1176575	1.071E-07
ZNF439	0.1843554	1.077E-07

DYNC1I2	0.1835727	1.079E-07
BIRC3	-0.2128035	1.082E-07
PRIM1	0.1097752	1.084E-07
MYO1G	-0.2321302	1.093E-07
MIA3	0.2027395	1.098E-07
DOK1	-0.1485003	1.101E-07
MYO1F	-0.2233117	1.114E-07
LIAS	0.1483324	1.125E-07
OGA	-0.2327947	1.149E-07
CLIC3	-0.1391903	1.155E-07
DTYMK	0.1879683	1.196E-07
CFDP1	0.2647203	1.203E-07
EMID1	0.1807864	1.224E-07
RNF169	0.1544027	1.24E-07
IKZF1	-0.2554123	1.242E-07
GCAT	0.1203743	1.27E-07
USP12	-0.2983969	1.275E-07
AURKAIP1	0.2041295	1.292E-07
TICAM1	-0.172001	1.308E-07
TWNK	0.1386008	1.31E-07
DDX60L	0.142436	1.319E-07
ACP5	-0.1557651	1.36E-07
PROM1	0.3144353	1.366E-07
GUK1	-0.2344412	1.373E-07
FAM160B1	-0.2033596	1.384E-07
TIMM9	0.2628821	1.397E-07
GRN	0.170667	1.411E-07
OPTN	-0.1231019	1.431E-07
PHKB	0.1971505	1.439E-07
WASHC3	0.1501669	1.503E-07
NDUFAB1	0.2483484	1.511E-07
TEDC1	-0.1390895	1.515E-07
ZC3H4	0.1429813	1.515E-07
C21orf62-AS	0.0938235	1.518E-07
LINC02802	0.0817793	1.555E-07
MS4A7	-0.150476	1.563E-07
NDST2	-0.1857246	1.605E-07
BAK1	-0.1968714	1.613E-07
AC244502.1	0.4256238	1.628E-07
GTPBP10	0.1163126	1.639E-07
RCN1	0.236028	1.646E-07
ST3GAL6	0.106695	1.651E-07
IER5	-0.2960734	1.652E-07
MECR	0.0752908	1.666E-07
FBXO9	0.1604183	1.683E-07
THUMPD1	0.2513563	1.696E-07
TMUB2	-0.1787468	1.708E-07
CDS2	-0.2679177	1.71E-07
GPX4	0.1467868	1.742E-07
TMEM220	0.2099348	1.754E-07

MAMDC2	-0.1098317	1.795E-07
ADSS	0.1532688	1.811E-07
IVD	0.2261893	1.827E-07
NDUFAF6	0.1487383	1.829E-07
GATAD1	0.2458795	1.847E-07
RTF2	-0.2428118	1.864E-07
IFNAR2	0.1740028	1.92E-07
HSPG2	-0.1974189	1.92E-07
SCFD2	0.0722131	1.947E-07
ANKRD27	0.1083342	1.948E-07
TMEM60	0.1539853	1.955E-07
HRH2	0.1182445	1.955E-07
CISD1	0.1589048	2.018E-07
IRF2BP2	0.3103783	2.029E-07
BIN2	0.2562982	2.081E-07
PRMT3	0.1226979	2.101E-07
HOMER3	0.1933325	2.109E-07
SEC22C	-0.1724932	2.11E-07
AC104041.1	0.1364973	2.111E-07
RALGAPA1	-0.1897485	2.168E-07
TMEM97	0.1293036	2.203E-07
INTS8	0.1717681	2.229E-07
L3HYPDH	0.0787884	2.254E-07
ZNF493	0.2185689	2.298E-07
CEP152	0.1371128	2.309E-07
C15orf61	0.1444849	2.311E-07
GINM1	-0.2429377	2.474E-07
OCEL1	0.1698709	2.479E-07
TNIP1	-0.2614089	2.5E-07
PREPL	0.116509	2.507E-07
IL6R	-0.2028505	2.53E-07
NOP58	0.2499435	2.574E-07
CCDC66	0.2074629	2.6E-07
AP1S1	0.1648947	2.616E-07
LAP3	0.1804776	2.673E-07
C19orf48	0.1482021	2.674E-07
ADAT2	0.1255657	2.715E-07
HDLBP	0.1740758	2.749E-07
ANO6	0.1385622	2.759E-07
LINC01684	0.2177977	2.826E-07
ADCY7	-0.1535477	2.828E-07
VANGL1	-0.2050494	2.864E-07
ZNF638	0.3236087	2.883E-07
CARD6	0.1416647	2.91E-07
GPSM3	-0.2528467	2.939E-07
DCXR	0.2200965	2.946E-07
SLC35A1	0.146273	2.947E-07
TNFAIP8	0.2668712	2.994E-07
ZNF573	0.0743116	3.07E-07
PRPF8	0.2441186	3.127E-07

MPST	0.195259	3.162E-07
ZMYM1	0.1716228	3.185E-07
DOK3	0.1634803	3.219E-07
MAPK12	0.0919034	3.225E-07
IFT74	0.1510561	3.234E-07
NIBAN2	-0.1074374	3.238E-07
GFI1	0.1996427	3.243E-07
SPPL3	-0.2187421	3.25E-07
EVI2B	0.2729593	3.269E-07
EEF1D	-0.1514209	3.325E-07
RRBP1	-0.1674084	3.354E-07
URB1	0.1155693	3.368E-07
NFXL1	0.1291109	3.432E-07
AC008267.5	0.0792933	3.48E-07
ACTN4	-0.2368079	3.497E-07
TRAP1	0.1207174	3.514E-07
NRAS	-0.268209	3.521E-07
PDLIM7	-0.1731414	3.555E-07
RAB6A	-0.2145941	3.599E-07
ZNF117	0.1511384	3.642E-07
CEP162	0.1389322	3.662E-07
UQCR11	0.2214519	3.722E-07
KIAA1841	-0.1337265	3.756E-07
IRF3	0.1473269	3.847E-07
RBM25	-0.286468	3.859E-07
CPSF3	0.1213753	3.898E-07
MCEE	0.1151215	3.906E-07
ZNF708	0.1705028	4.024E-07
CMIP	0.1404974	4.052E-07
DTWD1	0.205305	4.053E-07
TBL1XR1	0.1996897	4.078E-07
PPM1A	-0.1893595	4.135E-07
SUMO3	0.1729777	4.136E-07
CFAP20	-0.1587135	4.16E-07
TMX3	0.1682958	4.166E-07
PHPT1	0.1823021	4.168E-07
DDX23	0.1429193	4.168E-07
TRAF3	-0.2185167	4.174E-07
KCNK17	-0.2026877	4.238E-07
CCDC127	-0.1511858	4.242E-07
HS2ST1	0.1448638	4.25E-07
MMP24OS	-0.2284574	4.25E-07
GPR27	0.1553965	4.283E-07
PHF23	-0.2211883	4.309E-07
PDHA1	0.1556034	4.317E-07
PWWP2B	-0.1951163	4.327E-07
CCNY	0.1797933	4.342E-07
SDSL	0.2312506	4.366E-07
CCT8	0.1896496	4.372E-07
AC106791.1	0.1072463	4.372E-07

STAB1	0.2181969	4.46E-07
PUM3	0.2575601	4.486E-07
SCAMP1-AS	0.1463743	4.495E-07
ZNF121	0.1728304	4.496E-07
THBS1	-0.1439694	4.548E-07
FCHSD2	0.1829393	4.56E-07
GORASP2	-0.2014183	4.6E-07
RPL26L1	0.1230374	4.615E-07
TMEM43	-0.2341993	4.662E-07
WARS2-AS1	0.0917933	4.705E-07
TRPC4AP	-0.1212889	4.73E-07
RPS25	0.1229786	4.918E-07
ARF3	-0.2318782	4.921E-07
ATP6V0E1	0.2229275	4.985E-07
CAPN2	-0.1723729	5.006E-07
SNHG3	0.2722959	5.019E-07
AUXG01000	0.0831961	5.059E-07
GON7	0.135256	5.099E-07
MCFD2	-0.2006663	5.139E-07
NDUFAF4	0.1006424	5.26E-07
WDR74	0.1267513	5.315E-07
MYH9	-0.2787688	5.316E-07
PLXNA3	-0.1776363	5.373E-07
ATG7	0.1048899	5.405E-07
BMF	-0.2003569	5.558E-07
NCBP2	0.2249172	5.562E-07
STIM2	-0.2563067	5.7E-07
FOSB	0.2100205	5.76E-07
PSMD6	-0.24568	6.116E-07
NEXN	0.1084658	6.124E-07
LRRCC1	0.1730642	6.141E-07
PPDPF	-0.2523436	6.154E-07
ZNF22	0.2291454	6.2E-07
POLR2J3	0.2610958	6.284E-07
RPL22	0.1189822	6.291E-07
COX18	0.1303612	6.547E-07
RANBP1	0.272171	6.742E-07
TCF15	0.0665177	6.822E-07
RRM1	0.1480215	7.002E-07
SH2B2	-0.1184081	7.057E-07
SAMM50	0.1064973	7.12E-07
IER2	0.3760004	7.169E-07
CCDC91	0.1904639	7.177E-07
CCDC125	0.1790755	7.212E-07
HEBP1	0.198539	7.295E-07
DGKZ	-0.1902484	7.322E-07
NFKBIE	-0.1707384	7.397E-07
ZRANB1	-0.229776	7.412E-07
ECHS1	0.2004149	7.453E-07
IPO7	0.1849655	7.475E-07

RAB11FIP4	-0.1204946	7.539E-07
PABPC4	0.2319734	7.552E-07
EXOC2	0.0574847	7.636E-07
SPIDR	0.1458685	7.679E-07
SPHK1	-0.1290228	7.691E-07
ASCL2	0.0897862	7.738E-07
SPG21	0.1533271	7.851E-07
SZRD1	-0.2190981	7.913E-07
PDE4DIP	0.1126525	7.921E-07
RABGAP1	0.1825737	7.975E-07
PRMT5	0.1113701	7.979E-07
MED8	-0.144774	8.131E-07
AHCYL1	0.173282	8.216E-07
B3GALT4	-0.1411467	8.862E-07
CEP57L1	0.1362622	9.023E-07
BFAR	-0.1638173	9.035E-07
TRAF3IP1	0.1038408	9.049E-07
C12orf75	-0.1576502	9.061E-07
LITAF	-0.2124877	9.261E-07
ADO	0.0355059	9.448E-07
ASH2L	0.1123459	9.785E-07
CDH26	0.091401	9.785E-07
CENPB	0.112956	9.812E-07
SPAG1	-0.1364466	9.82E-07
RAB7B	0.1339423	1.021E-06
COA7	0.1122136	1.025E-06
MAPKAP1	0.1606116	1.027E-06
CCDC169	0.0914147	1.037E-06
SPRY2	0.0659457	1.038E-06
PFAS	0.0850006	1.045E-06
C11orf24	-0.0977209	1.046E-06
RASGRP2	-0.2221038	1.071E-06
PPP3CB-AS	0.1324567	1.073E-06
LAT2	0.2230633	1.087E-06
BZW1	-0.2501145	1.091E-06
OSCAR	-0.1867389	1.097E-06
HMG20B	0.074571	1.114E-06
LPIN1	-0.20641	1.141E-06
SCOC-AS1	0.0865705	1.207E-06
SNX18	0.0737617	1.226E-06
GLOD4	0.2169836	1.228E-06
CARF	0.106969	1.229E-06
ZNF302	0.2107644	1.253E-06
ZFP62	0.0903939	1.255E-06
NUP98	-0.2132841	1.257E-06
TMEM131L	-0.218613	1.27E-06
JMJD6	-0.2082857	1.275E-06
RNF13	0.1764903	1.296E-06
MRPL55	0.1917927	1.335E-06
CCDC102B	0.1152084	1.343E-06

MMRN1	0.2173596	1.349E-06
TMEM234	-0.1124745	1.356E-06
FUT8	0.0794214	1.361E-06
NT5C3B	0.1364811	1.365E-06
SLC30A9	0.1299298	1.376E-06
AC010642.2	0.1214594	1.377E-06
ELOA-AS1	0.0777132	1.38E-06
CYTH3	-0.0851968	1.397E-06
IFNGR1	-0.1710864	1.435E-06
APOL1	0.0836936	1.438E-06
MRPL50	0.1718838	1.445E-06
ZFR	-0.2458672	1.456E-06
CCT2	0.1787713	1.458E-06
ARPC5	0.286079	1.459E-06
ZNF727	0.1371533	1.47E-06
TMBIM6	-0.1944824	1.489E-06
SLC35B4	0.11269	1.496E-06
GADD45A	0.1232252	1.497E-06
DERL1	-0.2095081	1.507E-06
CTSH	0.1022984	1.514E-06
AL031595.3	-0.1390583	1.514E-06
CPSF7	-0.1679381	1.534E-06
MTHFD1	0.1518623	1.562E-06
BAG2	0.1229964	1.563E-06
LSM6	0.2055303	1.578E-06
PIP4K2B	0.1205345	1.625E-06
SLX4IP	0.1056843	1.627E-06
LINC00205	0.108329	1.642E-06
SFXN1	0.1087847	1.662E-06
TMEM209	0.1012716	1.665E-06
SLC39A8	0.1445381	1.67E-06
COA1	0.1359899	1.673E-06
ADI1	0.2487459	1.686E-06
CHMP5	0.1766353	1.695E-06
RABIF	-0.2069372	1.733E-06
ELF2	-0.1744704	1.733E-06
COL5A1	0.0759339	1.745E-06
CCT6A	0.2555242	1.747E-06
FURIN	-0.166267	1.769E-06
CHCHD10	0.2236183	1.772E-06
RNH1	0.1862984	1.778E-06
SPATA13	-0.1837728	1.789E-06
GAMT	0.1105731	1.802E-06
ERCC5	0.2032401	1.827E-06
EEF1AKNMT	0.130078	1.85E-06
PLXNB2	-0.2177494	1.874E-06
ASRGL1	0.1818374	1.881E-06
SH3TC1	-0.1856392	1.936E-06
SLC45A4	-0.1231133	1.938E-06
AK9	0.0921253	1.945E-06

TACC3	0.2359775	1.953E-06
TNRC6B	0.2466311	1.963E-06
C21orf58	0.1134978	1.99E-06
SCAND1	-0.2864685	2.055E-06
EHMT1	0.2352485	2.095E-06
PRRC2C	-0.2846617	2.113E-06
DMAC2L	0.1429951	2.158E-06
RALGAPA2	0.1281276	2.179E-06
STAT1	0.2065353	2.18E-06
SNX4	-0.1408246	2.181E-06
MPG	0.2991098	2.235E-06
WHAMM	-0.1739075	2.239E-06
CUL4B	0.1427953	2.243E-06
CTNS	0.0879929	2.248E-06
CCDC149	0.1333954	2.288E-06
PIN4	0.2187092	2.302E-06
PTS	-0.1716869	2.321E-06
CHMP2B	-0.199456	2.326E-06
MCM3	0.1637624	2.366E-06
SEC23IP	0.1430835	2.394E-06
YAF2	-0.1684076	2.405E-06
TCEAL3	-0.2241687	2.504E-06
GLCE	0.0760255	2.508E-06
GNL1	-0.1825786	2.637E-06
C15orf65	-0.099417	2.64E-06
APLP2	0.3046659	2.651E-06
ATF1	0.1091395	2.654E-06
TSGA10	0.1180868	2.675E-06
SELENOF	0.179887	2.691E-06
DTNBP1	0.1605602	2.702E-06
MRPL15	-0.1941751	2.716E-06
IFIT1	0.070375	2.719E-06
XAB2	-0.132423	2.768E-06
ATP2B1-AS1	-0.3239515	2.776E-06
TFE3	-0.1327455	2.785E-06
ITGA6	0.1510266	2.827E-06
PNKD	0.218447	2.831E-06
ZNF397	0.1662873	2.888E-06
GMEB2	-0.1167362	2.909E-06
POLR1E	0.1356581	2.927E-06
TRAK2	0.1564464	2.937E-06
HMGB2	-0.2732089	2.94E-06
RAB22A	-0.2356378	2.968E-06
CCDC25	0.1407342	2.978E-06
VWA8	0.0691843	2.979E-06
NEK1	0.1006629	2.98E-06
THOC6	0.1096575	3.012E-06
RPS10	-0.2079979	3.053E-06
PDLIM2	-0.1830692	3.057E-06
PRRG4	0.1133069	3.064E-06

SUCLG2	0.1388429	3.093E-06
C1orf131	0.1633488	3.109E-06
CCT7	0.239238	3.16E-06
PGK1	-0.1855915	3.171E-06
EXOSC2	0.1159542	3.2E-06
ALAD	0.0829401	3.269E-06
ABCE1	0.2571374	3.294E-06
UMODL1-AS	0.0860346	3.295E-06
ATP5MPL	0.2444265	3.3E-06
N4BP2L2	0.2366149	3.3E-06
MRPS5	0.2123079	3.38E-06
STK11	0.1045622	3.405E-06
FAM50B	0.0996079	3.414E-06
SCCPDH	0.1967778	3.443E-06
MVP	-0.174266	3.549E-06
CWC25	-0.2134015	3.549E-06
NAT10	0.1780774	3.564E-06
LMBRD2	0.0708609	3.6E-06
TMEM242	-0.1469989	3.61E-06
AIF1	-0.1843768	3.731E-06
LONRF1	-0.2019923	3.909E-06
TSR1	0.1741351	3.925E-06
PDCL3	0.1419643	3.946E-06
XPO5	0.0902222	3.983E-06
ALKBH2	0.1136367	3.986E-06
KAT6B	0.1394763	3.994E-06
CAMK1D	-0.2097377	4.06E-06
MAGOHB	0.128649	4.084E-06
NUFIP1	0.0965158	4.108E-06
STAU2	0.1377132	4.148E-06
KMT2B	0.1152218	4.161E-06
PRELID1	-0.2337818	4.17E-06
POLE	0.1320077	4.211E-06
RPRD1B	-0.1743562	4.231E-06
MYO18A	0.1117023	4.292E-06
BRI3	0.2191319	4.298E-06
PTGER2	-0.1875585	4.319E-06
GNG10	0.1511792	4.321E-06
PFDN6	0.1411992	4.323E-06
PPM1H	0.0699612	4.427E-06
GLTP	-0.1527447	4.469E-06
ADRM1	-0.2049198	4.504E-06
AC004687.1	0.3328155	4.508E-06
HIST1H2BG	-0.133479	4.673E-06
HNRNPH1	-0.1985717	4.749E-06
DCTN4	-0.2140442	4.753E-06
UBXN7	-0.2186873	4.79E-06
PCNX4	0.1953444	4.848E-06
LIG4	0.1321676	4.916E-06
GPALPP1	0.1903678	4.993E-06

MFSD3	0.0983475	4.994E-06
GNG7	0.1535578	5.003E-06
KIAA0232	-0.2102032	5.051E-06
TIFA	0.0794561	5.065E-06
PIK3CA	-0.2113352	5.092E-06
FAN1	0.074617	5.12E-06
STRADB	0.1169805	5.192E-06
MAGOH	-0.1873185	5.23E-06
ELOC	-0.2102336	5.248E-06
ALG6	0.0863648	5.304E-06
UROS	0.2661621	5.315E-06
TMEM147	0.1977568	5.389E-06
NFIC	0.2127021	5.41E-06
RBFA	0.1824728	5.42E-06
PSIP1	0.2566527	5.429E-06
POMP	-0.2443886	5.476E-06
GALNT7	0.2521388	5.537E-06
DNAJC5	-0.1503725	5.569E-06
CEBPZ	0.2866349	5.586E-06
METAP1D	0.1092951	5.611E-06
CAMTA1	-0.2084837	5.855E-06
INF2	-0.151924	5.859E-06
SMARCC2	0.197982	5.945E-06
NECTIN2	-0.1581	5.961E-06
GSKIP	-0.1482234	6.002E-06
ARHGAP32	0.0727915	6.048E-06
SGPL1	-0.1535901	6.071E-06
CREB3	-0.1522294	6.184E-06
PRKCD	-0.1772692	6.22E-06
GAS6-AS1	0.1541189	6.229E-06
KAT14	0.0962672	6.345E-06
PET117	0.1029653	6.361E-06
ANKRD11	0.0531067	6.382E-06
SPATA5	0.1002014	6.513E-06
TRAPPC4	-0.1954911	6.524E-06
NDUFS2	0.2135148	6.696E-06
IL12A-AS1	0.1530537	6.768E-06
FNDC10	0.0576819	6.837E-06
LMO4	0.1998814	6.917E-06
PPM1F	-0.2204376	6.924E-06
SCLY	0.0731974	6.969E-06
TIA1	0.1915443	7.012E-06
BLOC1S5	0.1398453	7.11E-06
DCTN3	-0.1862839	7.127E-06
GEMIN6	0.125883	7.141E-06
GNG5	-0.1878487	7.167E-06
ACSF3	0.1999214	7.26E-06
STK26	0.205373	7.375E-06
PHF1	-0.1782771	7.556E-06
ZNF322	0.1392258	7.581E-06

RPS13	0.1164715	7.599E-06
DPPA4	0.148359	7.606E-06
AIP	0.1037874	7.608E-06
UQCRHL	0.0540309	7.702E-06
NAE1	0.1444503	7.711E-06
STIP1	0.1559442	7.731E-06
CXCL2	0.0936338	7.772E-06
ABI1	-0.252908	7.962E-06
ZDHHC21	0.1695552	7.971E-06
PTCD2	0.0896565	8.005E-06
ORC2	0.1321736	8.05E-06
C19orf12	-0.1278952	8.172E-06
FADS3	-0.1684835	8.215E-06
CERT1	-0.2524795	8.23E-06
CAPN1	-0.2117461	8.238E-06
UTP20	0.1484771	8.26E-06
TOP1MT	0.1245782	8.296E-06
MOB2	-0.0565428	8.355E-06
DDX27	0.180723	8.408E-06
DIPK2A	0.1334472	8.681E-06
KANTR	0.1352464	8.727E-06
TMEM126B	0.1832458	8.75E-06
CSF2RB	0.2009275	8.803E-06
NNT-AS1	0.1441449	8.827E-06
MORC2	0.1585749	8.883E-06
UBXN8	0.0667059	8.963E-06
TAF4B	0.0793652	8.983E-06
FBXO4	0.088449	8.997E-06
CARD9	0.0964738	9.124E-06
UBA3	0.1712456	9.257E-06
AFG3L2	0.1561984	9.3E-06
ANAPC11	0.1781636	9.476E-06
IFT22	-0.1669002	9.537E-06
HCFC2	-0.1207316	9.662E-06
VPS53	0.1513919	9.695E-06
LINC01637	0.1054373	9.862E-06
PCNT	0.1170313	1.002E-05
DHODH	0.1094256	1.025E-05
PAXBP1	0.2119208	1.033E-05
THOC3	0.1306109	1.039E-05
GLT8D1	0.1013054	1.06E-05
PIP4K2A	0.132865	1.063E-05
LINC02245	0.1053434	1.064E-05
SUN1	-0.1641862	1.069E-05
LAMP2	0.218038	1.074E-05
SAMD1	0.1009354	1.075E-05
ZMPSTE24	0.0977718	1.089E-05
SLU7	-0.2360711	1.101E-05
TCEANC2	0.1157607	1.123E-05
PRDX4	0.2006393	1.123E-05

PRSS2	-0.5946033	1.128E-05
KIAA0100	0.1591863	1.129E-05
SNRNP200	0.1846458	1.129E-05
ANXA6	-0.1754384	1.135E-05
AL360012.1	0.0524843	1.142E-05
NR1D2	0.095972	1.147E-05
SLC31A2	-0.0954254	1.16E-05
H2AFY2	0.0969709	1.174E-05
NPW	0.2071708	1.18E-05
BRD8	0.2238634	1.187E-05
AC016831.7	-0.1297778	1.193E-05
QKI	0.1854151	1.199E-05
SNHG20	0.0973429	1.203E-05
ALDH18A1	0.1021932	1.208E-05
SELENOH	0.2182072	1.215E-05
CDKL3	0.0600457	1.222E-05
MYLK-AS1	0.0882268	1.246E-05
ALCAM	0.2085197	1.249E-05
VPS25	0.0862665	1.257E-05
PXN-AS1	0.0733828	1.265E-05
PEF1	-0.198123	1.282E-05
TTN-AS1	0.1254542	1.294E-05
DCAF17	0.1051584	1.306E-05
RBM22	-0.1190041	1.307E-05
ECM1	-0.1436047	1.32E-05
SLIRP	0.2430614	1.322E-05
BX890604.2	0.2120666	1.34E-05
VSIG10	0.1089629	1.343E-05
ANAPC5	0.2285296	1.37E-05
CD79B	0.1446143	1.384E-05
NACC2	-0.087902	1.387E-05
CRYBG3	0.1600841	1.39E-05
ZNF598	-0.14074	1.4E-05
USP22	-0.2316276	1.402E-05
THAP11	-0.1192223	1.409E-05
IL27RA	-0.2806117	1.409E-05
SSRP1	0.1877261	1.41E-05
DEGS1	-0.2034136	1.413E-05
RNF25	-0.106972	1.44E-05
NUDT9	0.0949343	1.44E-05
SLC16A1	0.0940336	1.449E-05
NUP133	0.1066783	1.45E-05
MTHFS	0.1306294	1.452E-05
HNRNPA3	-0.2689623	1.46E-05
TPP1	0.2053468	1.461E-05
P2RY8	0.1345568	1.485E-05
PINX1	0.1067745	1.516E-05
TRIM47	-0.085008	1.516E-05
SURF2	0.0576991	1.545E-05
RNASEH1-A	0.0817122	1.548E-05

PRMT7	0.1286899	1.551E-05
LAMTOR2	0.2275106	1.561E-05
MARC2	0.0847359	1.567E-05
FECH	0.1242999	1.568E-05
ST3GAL4	0.1609577	1.575E-05
NSUN6	0.1351488	1.59E-05
LINC00467	0.1151457	1.595E-05
LINC01215	-0.1228222	1.596E-05
RPARP-AS1	0.1894783	1.645E-05
SCHIP1	0.1436796	1.645E-05
SLC25A46	0.1549451	1.658E-05
FAM219B	-0.1803705	1.671E-05
NDUFA6	-0.1916533	1.676E-05
GNPDA2	0.0650836	1.683E-05
MPDU1	-0.1156609	1.684E-05
FBXO25	0.1060411	1.696E-05
SNHG30	0.1638461	1.705E-05
P2RY1	0.0764312	1.705E-05
SMIM10	0.0710522	1.72E-05
AMPD3	0.0694388	1.726E-05
GFOD2	-0.1014101	1.732E-05
KTN1-AS1	0.0512618	1.735E-05
ATP6V1F	0.2628821	1.735E-05
ASAP1	0.1020315	1.751E-05
RTN3	0.2065009	1.759E-05
SUSD3	0.0856607	1.768E-05
NAPEPLD	0.113683	1.797E-05
COX15	0.1144825	1.814E-05
NDUFA2	0.1830166	1.817E-05
FGD4	0.1180707	1.82E-05
MIRLET7BH	-0.1824497	1.829E-05
MARS	-0.1370591	1.831E-05
MRM1	0.0543433	1.835E-05
INTS6	-0.2790823	1.851E-05
ITFG1	0.0649384	1.872E-05
PLXNC1	0.1193274	1.95E-05
OSGEPL1	0.1055272	1.957E-05
EHBP1	0.102633	1.968E-05
MCRIP1	-0.1790221	1.969E-05
MRPL12	0.2207391	1.971E-05
RNF44	0.1065772	1.972E-05
MEGF8	0.0638439	1.986E-05
COG2	0.126401	1.993E-05
WHRN	-0.081528	2.009E-05
SNRNP25	0.1566281	2.013E-05
COX5A	0.1280813	2.015E-05
GALM	0.0420181	2.076E-05
AC093157.1	0.0901915	2.082E-05
POLR2B	-0.0912014	2.11E-05
C12orf29	0.1020083	2.117E-05

C7orf50	0.11986	2.136E-05
BICD2	-0.1988133	2.14E-05
MT1X	0.0773512	2.154E-05
NPRL2	0.1331174	2.163E-05
STUB1	0.137001	2.165E-05
NRBP1	-0.2028807	2.171E-05
KDM6A	-0.1750541	2.185E-05
HENMT1	0.1107377	2.196E-05
ATP6V0A2	0.1725303	2.196E-05
SLC46A3	0.117618	2.203E-05
CBL	-0.2073511	2.23E-05
SMIM20	0.1659876	2.236E-05
RUNX2	0.1641117	2.236E-05
RSRC1	0.2139695	2.252E-05
XRCC4	0.0788203	2.273E-05
PTMA	0.1428018	2.277E-05
PPP2R2A	-0.2023528	2.285E-05
HYAL3	0.0806917	2.292E-05
EIF4EBP3	0.215049	2.314E-05
ATP10D	0.1089661	2.316E-05
UBIAD1	0.0851845	2.319E-05
SCRN3	0.0801386	2.323E-05
SLC27A5	0.129856	2.34E-05
SELPLG	0.1339853	2.34E-05
G6PD	-0.2036829	2.357E-05
SRSF1	0.2866816	2.357E-05
LARP4	0.1266373	2.387E-05
LCP2	0.1052543	2.389E-05
DEPDC1B	0.0811089	2.398E-05
EIF2AK1	0.2701757	2.403E-05
COPA	-0.1583778	2.423E-05
RAF1	-0.1406154	2.425E-05
GLI4	0.1167042	2.427E-05
RNF187	0.1456649	2.442E-05
DLGAP4	-0.1815085	2.458E-05
EIF4EBP1	0.2043599	2.537E-05
DCPS	0.1483685	2.544E-05
ACADVL	-0.1866027	2.57E-05
CTPS1	0.1595447	2.622E-05
NDUFB5	0.2195154	2.632E-05
UBALD2	-0.1501224	2.635E-05
PRKCQ	0.1071594	2.663E-05
DNAJC10	0.2102626	2.696E-05
ARFGAP2	-0.1778674	2.699E-05
RPL26	0.1441725	2.707E-05
PBX3	-0.2595178	2.707E-05
NARF	-0.1814211	2.714E-05
MRPL39	-0.1208845	2.779E-05
PRDM8	-0.144619	2.837E-05
PHF6	0.1807576	2.85E-05

ADARB1	0.0749979	2.854E-05
WDR46	0.1508818	2.897E-05
TFEB	-0.1528887	2.933E-05
KBTBD2	-0.1906094	2.943E-05
SLX4	-0.0865257	2.945E-05
ASPSCR1	0.1203025	2.946E-05
DHRS4	0.1118773	3.003E-05
CD52	-0.2448131	3.01E-05
ZNF106	0.1975109	3.024E-05
DMTN	-0.085421	3.071E-05
AC105285.1	0.1358587	3.097E-05
SCAMP1	0.1320701	3.127E-05
CISD3	0.1670669	3.19E-05
HELLPAR	0.1486954	3.213E-05
SYNE1	0.1034278	3.218E-05
RXFP1	0.1042708	3.254E-05
TYW1	0.0783256	3.27E-05
SS18L2	0.0929666	3.319E-05
CCDC61	0.0559383	3.353E-05
APH1A	-0.1386392	3.427E-05
LINC02693	0.056634	3.486E-05
SIN3A	-0.1836329	3.508E-05
DERA	0.1118253	3.519E-05
CISH	0.1311302	3.538E-05
MRPS15	0.1565357	3.544E-05
AL356804.1	0.1053854	3.578E-05
NCBP2AS2	0.1473792	3.586E-05
MBLAC2	0.1036151	3.588E-05
CYBRD1	0.1740836	3.59E-05
NINJ2	0.0933546	3.631E-05
KIFC3	-0.1457341	3.664E-05
GID4	0.0913246	3.706E-05
DPH5	0.09931	3.706E-05
ISG20	-0.1274799	3.738E-05
C5orf24	0.1934922	3.74E-05
PYCR2	0.1860636	3.817E-05
PECAM1	0.2612844	3.818E-05
RASA4	0.1020224	3.825E-05
TTLL1	0.0799127	3.834E-05
SYTL1	-0.1657564	3.88E-05
C17orf49	-0.2287291	3.882E-05
BCL7A	0.1434104	3.897E-05
CD2AP	-0.2564925	3.899E-05
ZNF431	0.2351568	3.957E-05
SYVN1	-0.1722696	3.986E-05
ZNF609	0.1364281	4.014E-05
DGLUCY	0.2071543	4.058E-05
ZBTB34	-0.1079816	4.101E-05
PELP1	0.1022925	4.138E-05
SNHG4	0.1878673	4.154E-05

FAM114A2	0.1416943	4.164E-05
ZNF395	-0.2133084	4.18E-05
WDR13	-0.2026031	4.203E-05
ELP1	0.0755014	4.233E-05
FARSA	0.2174412	4.268E-05
ATR	0.1701247	4.297E-05
RPP40	0.1152524	4.407E-05
LINC00910	0.0418104	4.431E-05
MRPS25	0.162172	4.434E-05
KIF20B	0.2636864	4.473E-05
RAB38	0.1227519	4.484E-05
ZNF83	0.1460074	4.501E-05
SCP2	0.2378827	4.52E-05
SERPINB9	-0.195691	4.528E-05
KAT5	-0.1421287	4.528E-05
PIBF1	0.1602887	4.632E-05
RCCD1	0.0960608	4.64E-05
SH3D21	0.1623012	4.656E-05
TRIM22	0.1814293	4.713E-05
CEP44	0.108729	4.723E-05
AC026124.1	0.1219187	4.729E-05
PPIL3	0.0889984	4.738E-05
PUM1	-0.2130883	4.743E-05
SLC35B3	0.0772051	4.756E-05
MRPS28	0.1415478	4.759E-05
LLPH	0.1494226	4.857E-05
SEC63	0.1709874	4.861E-05
GARS	-0.2188078	4.868E-05
KLHL26	-0.0782925	4.917E-05
TMEM14A	0.0895327	4.939E-05
SRPK2	0.0823721	4.973E-05
TTC14	0.2440985	4.979E-05
BSG	-0.2208113	4.987E-05
PTGES3	0.2185274	4.991E-05
TLR6	0.0795602	5.054E-05
C11orf1	0.1124768	5.146E-05
MED15	-0.1519922	5.165E-05
FAM13A	0.061687	5.183E-05
ATP5MF	0.20224	5.183E-05
DCAF15	-0.1514451	5.209E-05
AC246817.1	0.086648	5.254E-05
CDKN1B	0.0834056	5.286E-05
HAUS2	-0.1906099	5.294E-05
LRP10	-0.1291609	5.343E-05
TCEAL1	0.1168852	5.347E-05
IL4R	-0.1549948	5.358E-05
RYK	0.1377585	5.404E-05
GPCPD1	-0.2032496	5.417E-05
MYO9A	0.1366746	5.467E-05
ZNF593	0.1734978	5.498E-05

AFTPH	-0.2270377	5.53E-05
MRTFA	-0.1952471	5.531E-05
VWA5A	0.0765377	5.611E-05
USP9X	-0.1986144	5.66E-05
MAGT1	-0.1571922	5.729E-05
MECP2	-0.0999913	5.753E-05
RPS15	0.0992847	5.77E-05
PDAP1	0.1726754	5.781E-05
MAGEF1	0.0998922	5.801E-05
CD48	-0.064962	5.807E-05
CASP7	-0.1291924	5.853E-05
TPST2	-0.1200958	5.92E-05
GSE1	-0.2162452	5.955E-05
MTRNR2L8	0.1725768	6.134E-05
EDEM2	0.1118505	6.192E-05
SIRPA	0.0772266	6.213E-05
NUDT4	0.0791756	6.239E-05
LSM1	-0.1147502	6.297E-05
ATE1	0.1230081	6.348E-05
PPP5C	0.0643062	6.476E-05
B2M	-0.1345601	6.493E-05
DUSP1	0.305694	6.512E-05
AC097448.1	0.0695795	6.513E-05
NR1D1	0.0487706	6.531E-05
CCDC173	-0.108294	6.584E-05
UBE2G1	-0.1106565	6.734E-05
ATG4C	0.1511469	6.827E-05
GNL3L	0.1715472	6.828E-05
GAR1	0.1552459	6.836E-05
ACTR6	0.1340396	6.852E-05
SPRED2	-0.1312713	7.023E-05
TAS2R14	0.0625284	7.162E-05
TTN	0.0631676	7.227E-05
OAT	0.1316836	7.275E-05
SLC5A6	0.0610032	7.376E-05
COX6A1	-0.2006584	7.415E-05
CASP6	0.175839	7.431E-05
SLC29A1	0.0770909	7.457E-05
CALR	-0.3155192	7.583E-05
HSH2D	0.1758209	7.586E-05
RNF34	-0.1712772	7.591E-05
XRRA1	0.0674267	7.625E-05
ELANE	0.3237106	7.63E-05
EIF1B-AS1	0.1024755	7.708E-05
RSAD2	0.0924536	7.737E-05
PRNP	-0.1792114	7.93E-05
AP3S1	-0.1702719	7.953E-05
WBP1L	-0.1850811	7.961E-05
CEP250	0.0703003	7.993E-05
CD3EAP	0.0940384	8.004E-05

SMURF2	-0.1529025	8.014E-05
ZNF594	0.0773095	8.037E-05
LSM5	0.1709707	8.087E-05
CNTLN	0.1560479	8.101E-05
TBC1D13	0.1164211	8.114E-05
MRPL49	-0.1191262	8.171E-05
ATP6V1D	-0.1613833	8.181E-05
USP15	-0.2630632	8.302E-05
HLA-C	-0.2313106	8.322E-05
RNF181	-0.2043039	8.355E-05
TMEM80	0.1486573	8.368E-05
CDK19	0.1035378	8.432E-05
CHKA	0.0667792	8.467E-05
HUWE1	0.2354322	8.499E-05
RAD17	0.1080389	8.691E-05
TXK	0.1036747	8.843E-05
STK19	-0.1185817	8.856E-05
CACYBP	0.1418154	8.886E-05
TTC19	0.211984	9.123E-05
AC060780.1	0.189324	9.127E-05
PGBD4	0.0941804	9.194E-05
FAM160B2	-0.1119646	9.244E-05
DNAJC12	0.1166822	9.275E-05
CHPF2	-0.1231308	9.307E-05
UBE2F	0.1261662	9.36E-05
WAC-AS1	0.1651416	9.388E-05
DUBR	-0.109708	9.393E-05
PSMD11	-0.205154	9.418E-05
KCNQ5	0.1036377	9.492E-05
TCTN3	0.1394535	9.534E-05
CCNC	-0.1353492	9.671E-05
MLX	-0.102316	9.715E-05
DHX37	0.1140915	9.729E-05
PAXIP1-AS2	0.1134081	9.808E-05
PRR11	0.1086893	9.824E-05
FKBP3	0.1437151	9.875E-05
MACROD1	0.0916666	9.936E-05
CST3	0.410493	9.994E-05
AL157895.1	0.2291599	0.0001015
MTRES1	0.1048796	0.0001017
ZNF358	0.1143405	0.000104
SP110	0.2054365	0.0001041
MXI1	0.1655775	0.0001042
C20orf197	0.1014669	0.0001054
FBXO7	-0.2144561	0.0001055
GPN3	0.1763366	0.0001073
WDR36	0.1496938	0.0001096
TMA7	0.1482992	0.0001111
POU2F1	-0.131459	0.0001124
BRD3	0.1041846	0.0001137

LRP5	0.0734625	0.0001139
CUTA	0.2230515	0.0001144
HNRNPM	0.2071203	0.0001155
ERI2	0.0921693	0.0001173
RBM42	-0.1644737	0.0001175
CXorf21	0.1894291	0.0001186
H2AFZ	0.151991	0.0001199
SH3BP1	-0.174485	0.0001205
MCCC1	0.0718199	0.0001207
GABARAPL2	0.1347042	0.0001225
FBRS	-0.1857768	0.0001225
SENP6	0.2311254	0.000123
USP3	-0.1489547	0.0001235
TP53TG1	0.1307051	0.0001242
MRPL47	-0.1886233	0.0001253
DNAJC21	0.2119348	0.0001259
AL357079.1	0.0781621	0.0001266
PLEKHM3	0.071663	0.0001278
MCTS1	0.1980542	0.0001278
EIF2B3	0.103581	0.0001285
UPF3A	0.1836518	0.0001286
PRDX1	0.2340578	0.0001288
RPSA	-0.1805505	0.0001296
HEBP2	-0.1747539	0.0001298
CARS	-0.1628182	0.0001306
TIMM23B	-0.0847023	0.000131
PTX3	-0.19106	0.000132
KLHL18	-0.1808725	0.0001329
NANOS3	-0.0716311	0.0001333
ZNF518B	0.0688258	0.0001335
NIF3L1	0.0752319	0.0001338
METTL26	0.2212076	0.0001343
RIC8B	0.0580747	0.0001344
ZNF844	0.0590724	0.0001346
YWHAB	0.1973946	0.0001348
DPYD	0.1581503	0.0001348
CTSA	0.2284728	0.0001356
DPH6	0.0888503	0.0001361
HDAC2	0.1946758	0.0001377
ARHGAP9	-0.1327515	0.000138
MATK	0.1731024	0.0001381
RPL17	0.1200721	0.0001386
NBPF19	0.082191	0.0001387
ALG2	-0.1557004	0.0001387
BLOC1S4	0.0408846	0.0001391
RAB35	-0.1483961	0.0001402
EBLN3P	0.1348911	0.0001404
VEGFA	-0.11957	0.0001408
RPP25L	0.1791573	0.0001429
NUP54	-0.1079894	0.000143

ZNF414	-0.1351441	0.0001436
AKAP9	0.2980566	0.0001442
POLI	0.0856873	0.0001444
SEC24B	-0.1518942	0.0001445
DDX3X	0.2589375	0.0001446
UPF3B	0.1306563	0.0001449
AHDC1	-0.262073	0.0001461
IGFBP6	-0.0888179	0.000147
CCDC88B	-0.188259	0.0001473
RHOBTB3	0.1362152	0.0001487
LSM4	0.1976645	0.0001513
MPHOSPH9	0.1871589	0.0001521
TRERF1	0.0699235	0.0001531
ILF3-DT	0.2082375	0.0001531
NT5C3A	-0.1904961	0.000154
CCDC121	0.0684486	0.0001543
TMEM160	0.1869443	0.0001546
TM4SF1	-0.19385	0.0001554
THAP2	0.0942978	0.0001556
PGM2	0.1769141	0.0001561
RESF1	0.1605392	0.0001584
TAGLN	0.0767441	0.0001595
MRPS30	0.1807434	0.0001604
CA2	0.151023	0.0001614
PRPF4B	-0.2918473	0.0001618
RNF144B	0.0650521	0.0001641
RHBDD2	-0.1589377	0.0001643
PXN	-0.153908	0.0001648
ADAMTS6	0.0765297	0.0001657
STX18-AS1	0.0701684	0.0001701
SSBP4	-0.1549938	0.0001724
AC025040.1	0.0619201	0.0001746
NKAP	0.1224498	0.0001802
FAM228B	0.1410344	0.000182
QDPR	0.1606987	0.0001826
ZNF385A	-0.1664755	0.0001826
THAP5	0.129723	0.0001849
FAM47E	0.0551974	0.000185
DTX3L	0.1389026	0.0001856
LENG8-AS1	0.1262676	0.0001858
ZNF383	0.0776451	0.0001859
KCTD5	-0.0967504	0.0001861
SETD2	-0.2414466	0.0001861
SPECC1	0.1840034	0.0001867
DPF2	-0.0974929	0.000188
MARCKS	-0.1864496	0.000188
ARSD	0.1247427	0.0001883
TFAM	-0.2359549	0.0001897
RHOT1	0.1495399	0.0001897
HDHD5	0.0840648	0.0001935

NUP153	-0.1383455	0.0001942
DIAPH2	0.1743131	0.0001951
AC004540.1	0.0934155	0.0001952
ATP1B3	0.2054723	0.0001954
HELB	0.1285238	0.0001961
CYP4V2	0.0890753	0.0001962
LINC00339	0.1011091	0.0001971
NMRAL1	0.1414916	0.0001985
SKIDA1	0.1622846	0.0002003
RRS1	0.1855408	0.0002025
AGPS	0.1051372	0.000203
CKLF	-0.3594581	0.0002034
PIGS	0.1172014	0.0002044
UBE2T	0.072036	0.000206
SLC39A11	0.0863631	0.0002107
SWI5	0.1334922	0.0002107
SSB	0.2730724	0.0002111
EEF1E1	0.1956255	0.0002144
PSPH	0.0562485	0.0002147
MKLN1	-0.1842244	0.0002154
FAM92A	0.1501171	0.0002202
HSBP1L1	0.1291172	0.0002229
LPXN	-0.1707924	0.0002249
POLR3D	0.1669411	0.0002254
ERP44	-0.1952545	0.0002261
UCHL5	0.1904981	0.0002264
PQBP1	0.1389607	0.0002284
BRD1	-0.1426116	0.0002309
SRA1	-0.1696189	0.0002315
ZNF174	0.0540558	0.0002329
NBPF14	0.0682437	0.0002351
TMEM251	0.1139973	0.0002372
PPARGC1B	0.0867763	0.0002405
ZNF48	-0.1560745	0.0002428
FBH1	0.1141185	0.0002442
SVOPL	0.0540729	0.0002472
BMP2K	0.21601	0.0002475
TUBB	-0.3276012	0.0002475
THEM6	0.0677182	0.0002524
KDM1A	0.2156353	0.000256
COQ9	0.098441	0.00026
LYPLAL1	0.1426222	0.0002602
DYNLL1	0.1853618	0.0002613
PPM1B	-0.2120446	0.0002659
TCFL5	0.0614066	0.000266
LINC01252	0.0746828	0.0002666
TP53I11	-0.1083907	0.0002677
MRPS22	-0.1685215	0.0002685
IFIT3	0.0752807	0.0002694
CD55	-0.2426543	0.0002702

BTBD7	-0.2209251	0.0002705
EXTL2	0.1193102	0.0002717
TMED10	-0.2124949	0.0002718
MRPL10	-0.1205086	0.0002735
PLPBP	0.1530268	0.0002742
TTC24	0.1149443	0.0002758
TIMM17A	-0.1887774	0.0002798
PLXDC2	0.1303319	0.0002813
JTB	0.1710412	0.000282
SSBP3	0.1657059	0.000284
STAT3	-0.1212495	0.0002852
LIPA	0.0949398	0.0002852
CDCA7	0.1236286	0.000286
EXOSC3	-0.0220328	0.0002866
MOSPD1	0.0504256	0.0002922
SKP2	0.0724678	0.0002939
PSMB2	-0.1543116	0.0002939
KIAA0586	0.1287937	0.0002957
POLR1A	0.1102644	0.0002982
RELT	-0.2525892	0.0002988
AL133243.3	-0.1469604	0.0002998
CDK16	-0.0820401	0.0003009
LARP1	0.2028199	0.0003022
TAF1	0.0845741	0.0003036
TATDN1	0.231831	0.0003037
PLXND1	0.1774811	0.0003041
THAP6	0.0981571	0.0003043
DCTN2	-0.1573012	0.0003053
SNUPN	0.1393909	0.0003093
NPM3	0.1282924	0.0003104
UGP2	0.2778514	0.0003114
NUP50	-0.0553907	0.0003117
TMEM41A	0.1137438	0.0003168
MGLL	-0.023256	0.0003182
C1orf162	0.1446622	0.0003213
LDAH	0.0950068	0.0003226
SHMT1	0.0713147	0.0003246
ZNF574	-0.0835538	0.0003277
MAPRE2	-0.2463721	0.0003292
SHANK3	-0.0548509	0.0003302
DIPK1B	0.1074001	0.0003309
NIPBL-DT	0.1676969	0.0003325
RPA2	0.1347229	0.0003357
DST	0.1088062	0.0003374
GTPBP4	-0.1823688	0.0003379
CHMP4A	0.1491263	0.0003385
SEC62	-0.1872898	0.0003426
CLTB	-0.0772347	0.0003426
LTC4S	0.1933781	0.0003434
IGFBP7	0.2414053	0.0003436

MAPK8IP3	-0.079204	0.0003506
TM9SF4	0.0929172	0.0003511
CD79A	-0.1176638	0.0003557
DHTKD1	0.1774071	0.000356
ARL8B	-0.1741739	0.0003567
CCDC57	0.1160509	0.0003605
ENOX2	0.0874907	0.0003629
OAF	-0.0786617	0.0003716
EIF2AK2	0.1810755	0.0003743
AC090825.1	0.0620939	0.0003746
CRLS1	0.1048097	0.0003796
EIF4E	-0.1587467	0.0003801
TSC22D4	0.176696	0.0003867
MESD	0.1980266	0.0003886
ZDHHC7	-0.1280564	0.0003908
ASPH	0.1702596	0.0003946
PTRH2	0.1456272	0.0003947
XPNPEP1	-0.1224585	0.0003971
CYB561D2	0.1459806	0.0003972
BCAP29	0.0339912	0.0003988
MAP2K4	-0.1173332	0.0003996
TP53I13	-0.1241764	0.0004025
NDRG1	-0.240277	0.0004041
TMEM59	-0.2055585	0.0004042
AC090630.1	0.1129898	0.0004048
PALB2	0.0551832	0.0004062
AC079781.5	0.0777263	0.0004089
TMEM259	-0.1729876	0.0004128
LNX2	-0.147655	0.0004214
DNAAF5	0.0674661	0.0004245
ALKBH3	0.0913956	0.0004248
PPP1R7	0.1432091	0.0004285
C3orf33	0.0600438	0.0004331
OGFOD2	0.0754177	0.0004348
CD34	-0.0873813	0.0004379
NPR3	0.1986723	0.0004394
ARMC1	0.0969758	0.0004395
SULT1A1	0.1350297	0.0004401
ZNF622	-0.1382788	0.0004407
JAK1	0.1015996	0.0004409
DHRS7B	0.1437158	0.0004412
NEK6	-0.1601061	0.0004418
ATP6V0B	0.1660739	0.0004457
ACSL3	-0.1557747	0.0004486
ACSL5	0.0997716	0.0004504
UBL7-AS1	0.0858468	0.0004666
ZMYM3	0.0938082	0.0004702
TRMT112	0.2062527	0.0004705
Z97192.1	0.060707	0.0004743
RFTN1	0.1181104	0.0004754

RPIA	0.1451757	0.0004764
SNAPC1	0.0788472	0.0004773
NAALADL1	0.0789436	0.000478
PRDX2	0.1600647	0.0004781
DAD1	0.1331743	0.0004792
CLSTN1	0.0893353	0.0004821
THUMPD2	0.0985564	0.0004847
AIFM1	0.1306876	0.0004858
ZNF33A	0.1177234	0.0004875
AP1AR	0.113368	0.0004902
WASHC5	0.1124867	0.0004924
FAU	-0.1414941	0.0004932
AGGF1	0.1578384	0.0004947
PTGER4	0.1608532	0.0004972
COQ2	0.0838411	0.000498
HEATR3	0.0870517	0.000507
MT-ATP8	0.2000743	0.00051
SPRYD7	0.076646	0.0005141
C12orf73	0.113697	0.0005153
DHRS4-AS1	0.1010865	0.0005169
TNFSF4	0.1113754	0.0005193
PSMB5	0.1543582	0.0005269
WDPCP	0.1059112	0.0005312
KIF1B	-0.1094057	0.0005315
MPP1	0.1863421	0.0005329
TMEM238	0.0812844	0.0005352
POU5F2	0.0538557	0.0005402
NSFL1C	-0.1626833	0.000549
EXOSC8	0.1464325	0.0005509
PSEN1	-0.1998615	0.0005534
GLB1	0.1070917	0.0005545
ZNF382	0.0492368	0.0005562
PRPS2	0.0846432	0.0005616
AEBP1	0.0929208	0.0005697
CCDC174	-0.1678432	0.0005752
NBEAL2	-0.1713668	0.0005755
PCBP1	0.1498471	0.0005768
IFIT5	0.0713546	0.0005784
NOL10	0.0995018	0.0005791
MICU1	0.1380509	0.0005843
RAE1	-0.1494584	0.0005956
DCP2	0.131678	0.0006006
UBL4A	-0.1665119	0.0006022
GOSR1	-0.166355	0.0006071
SNAP29	-0.1366855	0.0006071
BLM	0.1112478	0.0006126
ARFIP1	0.0547817	0.0006248
CYB5RL	0.059709	0.0006288
SYK	0.2481635	0.0006558
MIS18A	0.1279367	0.0006683

SH3KBP1	0.2344093	0.0006749
BEND6	0.0930366	0.0006866
MSN	-0.2106252	0.0006886
MICAL2	-0.0945485	0.0006888
FANCB	0.0485824	0.0006976
CREBZF	0.1394437	0.0006994
FAM200A	0.0403225	0.0006995
RAB11B	-0.1177322	0.0006996
HEMK1	0.0932526	0.0007001
SLC26A2	0.1149119	0.0007004
VAMP3	-0.1215572	0.0007182
MPC1	0.1159637	0.0007197
TMEM120B	-0.1428149	0.0007199
THAP9-AS1	-0.1557835	0.0007339
PIP4P2	0.1063073	0.0007367
UBE2G2	0.2277711	0.0007393
GALK2	0.1178377	0.0007534
ORAI2	-0.1323452	0.0007542
ATP23	0.0927264	0.0007578
TMEM167A	-0.1092133	0.000758
DFFB	0.0854404	0.0007669
FTSJ3	0.1240967	0.0007784
AL121933.2	0.0998867	0.0007821
SGF29	0.0902119	0.0007902
NR4A1	-0.1322501	0.0007921
CAMKK1	-0.0971965	0.0007923
F2R	-0.1412855	0.0008015
COPB1	-0.161501	0.000802
HLA-E	-0.1600737	0.0008198
AF117829.1	0.0977637	0.000834
ABCB7	0.0994056	0.0008451
TARS	-0.1357037	0.0008472
GABARAP	-0.1988437	0.0008543
EAF1	-0.196194	0.0008564
CAPN7	0.0866201	0.0008587
ZNF585A	0.0883129	0.0008631
PDRG1	-0.1418251	0.0008664
SLC22A4	-0.0739748	0.000872
MLF1	-0.1031336	0.0008793
RPL13	0.1218048	0.0008801
SLC9B2	0.0872623	0.0008841
TCF12	0.1101676	0.0008916
TPX2	0.1336095	0.0008931
ANAPC16	0.2128413	0.0009019
ZNF26	0.1735636	0.0009048
GALNT2	-0.1715893	0.0009051
PGBD2	0.0695268	0.0009094
CIB1	-0.1350465	0.0009113
C9orf139	0.2741989	0.0009121
ZNF90	0.074795	0.0009248

METTL8	0.1706773	0.0009302
ITGAX	-0.2382779	0.0009393
TGIF2	-0.1687183	0.0009425
CDCA4	-0.1379205	0.0009512
TPRG1L	-0.0683847	0.0009642
MFSD12	-0.1374858	0.0009658
MRPL51	0.154823	0.0009661
PSMF1	-0.1468501	0.0009699
TDRD3	0.1170327	0.0009742
NABP1	0.1355045	0.0009947
ZNF429	0.1319106	0.0010011
OAZ1	-0.1852261	0.0010085
MRPL4	0.203485	0.0010111
NDUFAF3	0.1221273	0.0010114
ABHD13	0.0874449	0.0010258
QPRT	0.0843965	0.0010269
POLR2G	0.1146652	0.0010286
MRPS21	0.2062597	0.0010307
JUN	-0.0274008	0.0010358
PLEKHA2	-0.1563246	0.0010358
SNX10	-0.2499677	0.0010378
SRP14	-0.2054137	0.0010388
USF1	-0.0905023	0.0010423
CCDC180	0.0612202	0.0010571
ZNF280D	0.1958193	0.0010591
CCDC69	-0.1910898	0.0010638
CUL5	-0.1718324	0.0010645
BSDC1	-0.1150936	0.0010765
RBM41	0.1258287	0.0010805
ZFP3	0.063739	0.0010867
AC026471.4	0.0836929	0.0011008
TRPS1	-0.2348529	0.0011052
ATP6AP1	-0.112469	0.0011068
TUT7	0.1249622	0.0011234
ZNF514	0.1023054	0.0011281
OAS1	0.0774794	0.0011372
CPA3	0.2471424	0.0011405
KLC1	-0.1668328	0.0011658
ARRDC5	0.0635372	0.0011778
ZNF512	0.1007151	0.0011795
CMPK1	0.1735399	0.0011875
HERC4	0.1330963	0.0011934
MPV17	0.1324376	0.0011944
CBX6	0.1262834	0.0011998
GPBP1L1	-0.1821919	0.0012013
CPSF4	0.0717198	0.001204
PLEK	-0.2284122	0.001225
TBL1X	0.0750452	0.0012257
CXXC1	0.1160406	0.0012309
SHPRH	0.1875817	0.001241

ADNP2	-0.19731	0.0012423
TXNDC9	-0.1533988	0.0012466
NIT2	0.2154172	0.0012852
RPS23	0.1135079	0.0012992
DLST	-0.1781847	0.0013047
EPS8L2	0.0687001	0.0013055
B3GALT6	0.1249076	0.0013071
XXYLT1	0.0575346	0.0013088
CBX4	0.0865616	0.0013151
TIMP1	-0.2195587	0.0013185
RARS	0.0732059	0.0013339
UFC1	0.0990779	0.001346
ARPC4	-0.1965766	0.001356
PPID	0.1938833	0.0013579
WDFY4	0.0922002	0.0013769
SNRPD1	0.2230407	0.0013784
APIP	0.1771162	0.0013804
CCDC106	0.1139889	0.001388
LYRM4	0.0644904	0.0013912
KPNA1	-0.0883111	0.0014076
DCAF16	-0.0540437	0.0014106
ZNF428	-0.1228132	0.0014168
N6AMT1	0.0495053	0.0014178
TMEM107	0.1605438	0.0014211
RAB30-DT	0.1109497	0.0014304
EIF3M	0.1876596	0.0014403
CETN2	-0.1246577	0.0014535
LXN	0.1233072	0.001456
FBXL4	0.070876	0.0014571
IRGQ	-0.1197561	0.0014572
LARP7	0.0845064	0.001459
SPPL2A	-0.1496028	0.0014624
SLMAP	-0.158757	0.0014683
LAGE3	0.1922837	0.0014699
TRIR	-0.1272213	0.001475
PSMD4	0.1293586	0.0014814
SCRN1	-0.1551285	0.0014831
BTN3A1	0.0838864	0.001486
KBTBD7	0.0879585	0.0014987
SNRPD2	0.1584158	0.0015049
C1orf43	-0.1250898	0.0015086
S100A1	-0.224309	0.001511
SLC35C2	0.0597525	0.0015112
NCKAP1L	0.1536721	0.001513
SCFD1	-0.1446036	0.0015133
MRPL32	0.1457692	0.0015172
HMCES	-0.1343726	0.0015202
CAST	-0.2166026	0.0015205
SLC39A6	0.1453136	0.0015356
MRPL54	-0.1596681	0.001547

PTGR2	0.0762115	0.0015484
ZNRD1	0.1753234	0.0015496
TIGD2	0.0500603	0.0015521
ZNF85	0.0844034	0.0015618
EPN1	-0.1552021	0.0015637
UROD	0.1267859	0.0015723
MRPL22	-0.1702459	0.0015944
VMA21	0.105862	0.001597
RO60	-0.1571354	0.0016115
THNSL1	0.0655857	0.0016268
FBXL15	0.1366416	0.0016326
GATM	0.069446	0.0016554
SLC27A2	0.0827024	0.0016687
ADIPOR2	-0.1669474	0.0016701
RNF113A	0.142971	0.0016931
MARK3	-0.1263588	0.001699
C12orf65	-0.1679507	0.0017039
RARG	-0.0802141	0.0017058
MDH2	0.0738229	0.0017139
SRP19	-0.162299	0.0017226
SLC2A3	-0.2125058	0.0017433
SBNO1-AS1	0.0737129	0.001762
ATG3	-0.1438403	0.0017622
CHORDC1	0.0896057	0.0017637
SLC33A1	0.073766	0.0017706
C17orf67	0.1241905	0.001772
MRPL35	0.1234121	0.0017917
UBA2	0.1793878	0.0018015
LINC02762	-0.1131851	0.00183
SMIM27	0.0997174	0.0018333
NORAD	0.1202369	0.0018344
UIMC1	-0.1738642	0.0018541
HMGN5	0.0694506	0.0018657
MTMR3	-0.0777699	0.0019016
TRMT10B	0.127497	0.0019204
ARHGAP18	0.2025558	0.0019282
KANSL1	-0.1550323	0.0019337
GRK2	0.1382261	0.0019416
BTBD10	-0.1347781	0.0019454
HPDL	0.0567006	0.0019515
CGGBP1	0.1407661	0.001953
GCHFR	0.1057473	0.0019547
ARL6IP1	0.2169351	0.0019567
NAPG	-0.1787094	0.0019698
COPS7A	-0.009163	0.0019709
SRD5A3	0.1755478	0.0019761
MNAT1	0.1140631	0.0019772
HELZ2	-0.0779884	0.0019774
POLE3	0.206014	0.0019902
TTC27	0.1167902	0.0019951

SDAD1	0.1253135	0.0020236
ROCK1	-0.27638	0.0020332
ZDHHC18	-0.1027658	0.0020467
LTB	-0.2381667	0.0020587
PSMC4	-0.1398677	0.0020689
UBL7	0.0635289	0.0021383
C2CD2	0.1070965	0.0021539
LY6E	-0.2056755	0.0021609
ZNF692	0.0767666	0.0021875
GALNT4	-0.1385675	0.0021883
DDA1	-0.1507978	0.0021986
MAN2A2	0.0761595	0.0022223
AC090114.2	-0.1321603	0.0022457
COA5	0.1311305	0.0022543
GMCL1	0.171666	0.0022597
PIEZO1	-0.0560572	0.0022669
MRPL11	0.1391911	0.002267
EFTUD2	0.103559	0.0022709
AC027097.2	0.0494742	0.002288
HAUS1	0.1112771	0.0023299
FAM200B	0.0643828	0.0023364
BRD2	-0.1792608	0.0023509
LINC02361	-0.0771713	0.0023514
STAT2	0.1254007	0.0023518
TGFBR1	-0.1951962	0.0023714
POLD4	-0.108145	0.002375
TXNDC12	0.1201844	0.0023825
LCORL	0.1395996	0.0023837
ATF5	-0.1496107	0.0023941
SLC22A18	-0.0572539	0.0023989
CWC27	0.177173	0.0024032
HMGCR	-0.1882232	0.0024081
CD59	0.1558662	0.0024097
SUMO1	-0.1989724	0.0024186
AP003068.2	-0.079715	0.0024295
UBXN4	-0.1636725	0.002435
BCKDHA	0.1159329	0.0024383
TGOLN2	0.1389382	0.0024468
EGLN1	0.1053585	0.0024469
ALDH16A1	-0.0704414	0.0024746
SPAG7	-0.1508949	0.0025259
SPTAN1	-0.1792774	0.0025321
LINC01023	-0.0749393	0.0025553
AGXT	0.2180529	0.0025588
TRAPPC3	0.1106624	0.0025626
MAFK	-0.1317783	0.0025848
NTMT1	-0.1212897	0.0026022
ANKLE2	-0.1866661	0.0026053
PWWP2A	0.1112259	0.002634
BAX	0.1693936	0.0026421

HTATIP2	0.1180769	0.0026533
AFF4	-0.2027529	0.0026673
BCCIP	0.1075635	0.002671
PHYH	0.1045898	0.0026879
ASB13	0.0688504	0.002692
IP6K1	-0.1536792	0.0026938
BBS10	0.0635831	0.0027157
LRRC26	0.1443852	0.0027165
RPS18	0.1019152	0.0028041
TRMT5	0.0650088	0.002825
ENDOV	-0.0973344	0.0028313
ALG13	0.0631134	0.0028402
NFIL3	0.0757882	0.0028603
RAB3D	-0.0979567	0.0028637
IFIT2	0.0344488	0.0028648
POLD2	0.1500564	0.0028799
OSTM1	0.0775835	0.0028811
SNHG17	0.1006997	0.0029026
AP002387.2	0.0808636	0.0029119
FAM30A	-0.1571601	0.0029177
AC011939.2	-0.0649125	0.0029362
ANP32E	0.2254724	0.0029411
MPP6	0.1051637	0.0029461
PLEKHA5	0.1686738	0.0029463
PARP11	0.0877439	0.0029719
KIAA1549	0.0605902	0.002983
DGKA	-0.1285124	0.0029955
CCDC136	0.0971678	0.0029964
FAM120C	0.0616596	0.0030389
NSUN2	0.0795811	0.0031225
ATP13A2	-0.1296811	0.003127
KRIT1	0.1199652	0.0031573
WDR27	0.1061108	0.0031574
NGRN	-0.1563319	0.0032026
NTHL1	0.1234502	0.0032113
AC010359.3	-0.0591778	0.0032475
PCYT1A	-0.101257	0.0032593
TSTD1	-0.2360524	0.0032629
RBM4	-0.243394	0.0032668
ODC1	-0.2039105	0.0033278
MRC2	0.05923	0.0034031
ASB8	-0.181923	0.0034538
HAUS4	0.0806426	0.0034604
WDR82	-0.1669681	0.0034782
PIK3R1	0.2037423	0.0035118
TOPORS	0.0979243	0.0035304
LCP1	0.2533492	0.0035325
MANBA	0.1814302	0.0035543
ORC3	0.1015389	0.0035584
ATP6V1E2	0.0442677	0.0035698

SYNJ2BP	0.1112362	0.0035949
PMF1	-0.1595611	0.0036022
IGHM	-0.1875011	0.0036608
ST8SIA4	-0.0047	0.0037254
RBM33	-0.1359807	0.0037316
KIZ	0.1061183	0.0037418
SOS2	0.0741967	0.0037594
AC026979.2	-0.1354194	0.0037742
TNRC18	0.1098352	0.0037872
NIN	0.2127011	0.0038157
FAM126B	-0.1635641	0.0038342
DFFA	0.1368731	0.0038416
CASD1	0.0724288	0.0038466
LINC00923	0.0550946	0.0038742
CHKB-DT	0.0564675	0.0038884
MT-CO2	0.1168661	0.0038946
TEX264	0.1625041	0.0038984
AL355488.1	0.0476146	0.0039239
COMMD9	0.140885	0.0039272
TTC28-AS1	0.0608499	0.0039355
IPO8	0.1295797	0.0039774
TMEM161A	0.0610871	0.0039802
NOL4L	-0.1577982	0.0039883
Z93930.2	0.0766751	0.0040027
FARSB	0.1288716	0.0040496
COX7C	0.1545529	0.0040811
LINC00623	0.0866495	0.004088
ZNF195	0.1179027	0.0040961
HSPA1A	0.0694159	0.0041033
WDR49	-0.127233	0.0041235
BTD	0.0708477	0.0041478
NSMCE1	0.1972394	0.0041725
NME3	0.1751692	0.004178
SEC61G	-0.089189	0.0041954
GMFB	-0.1828793	0.0042164
MIER2	-0.059569	0.0042306
ALS2	0.1134766	0.0042392
PCYOX1L	0.0702369	0.0042404
PIK3R5	-0.1131644	0.0042451
FAM117B	0.0720947	0.0042812
TFRC	-0.2657771	0.0042936
GEMIN4	0.0651044	0.0043189
RFC4	0.0578313	0.0043252
GNB2	-0.1289892	0.0043395
SF3B4	-0.1369411	0.0043757
SOGA1	0.0739868	0.0044328
ZMYND11	0.1322548	0.0044464
EEFSEC	0.1043093	0.0044669
CSNK1G3	-0.1646123	0.0045072
DDX52	-0.011273	0.0045293

EIF2A	0.1830673	0.0045313
ENO1	-0.1720143	0.0045641
JAGN1	0.0985996	0.0045671
AC005670.3	0.1886609	0.0046109
SF3B1	-0.2299297	0.0046196
MAD2L1BP	-0.1195242	0.0046261
DHX40	0.1174763	0.0046323
CEP192	0.1207898	0.004633
SGTB	-0.0888323	0.0046736
HIPK3	-0.1535608	0.0046836
GPD1L	0.0456019	0.0046894
CKAP5	0.1049157	0.0046928
DDX39A	0.1592682	0.004711
EPC1	0.0259199	0.0047227
HACD2	0.0733537	0.0047229
ZNF543	0.0258159	0.0047832
TTC32	0.1142443	0.004797
EIPR1	0.0553085	0.0048316
SEMA6B	-0.0873238	0.0048499
THOC1	0.143043	0.0048816
ARFGEF1	0.0205466	0.0048821
SYF2	-0.1771317	0.0049073
MDM1	0.1077672	0.0049171
RNF208	0.054252	0.0050246
COPE	-0.1544745	0.0050457
HOXA3	0.1432975	0.0050493
RPL18A	-0.1737621	0.0050628
LIX1L	0.0873773	0.0050941
CCDC117	0.1091252	0.0050947
C2orf69	0.0970887	0.0051201
IRAK3	0.2267668	0.0051495
PDIK1L	0.0577493	0.0051534
PRR5L	0.1261321	0.0051566
SFXN4	0.1644553	0.0051714
GGPS1	0.119884	0.0052077
HOTAIRM1	-0.1300189	0.0052451
ESD	0.18477	0.0052624
SURF4	-0.1440428	0.0052625
NFRKB	0.0585498	0.00529
ZBED3-AS1	0.0523651	0.0052984
MFSD14C	0.1032735	0.0053334
HYPK	0.0388233	0.0053624
EFL1	0.0687614	0.0053996
ENY2	-0.0405861	0.0054006
ATP6V1A	-0.1444556	0.0054155
CREBRF	-0.2308598	0.0054239
BISPR	0.0822375	0.005444
BCL2L12	0.0607547	0.0054926
GLE1	0.0701331	0.0055059
SEPTIN9	-0.1774267	0.0055083

MZF1-AS1	0.1016082	0.0055219
PCCA-DT	0.069757	0.0055633
NUP62	-0.0906518	0.0055995
XIST	0.19734	0.0056046
CLK4	-0.1671939	0.0056049
WDR48	-0.1374464	0.0056061
LINC00662	0.0864405	0.0056223
GIGYF2	0.1420964	0.0056463
TAPBPL	-0.1433566	0.0056598
TMEM35B	0.2055584	0.0056943
DPY19L4	0.1467247	0.0056988
PIGN	0.12084	0.005715
STAT4	-0.0876131	0.0057324
PIGH	0.0475566	0.0057418
CCDC107	-0.1522056	0.0057999
SDHAF4	0.0659326	0.0058031
TYSND1	0.0676094	0.0058313
PGP	0.146598	0.0058823
ERGIC1	-0.1308668	0.0058954
MTRF1	0.0666232	0.0059137
EIF3F	-0.1345181	0.005967
AMPD2	-0.0599872	0.0060622
DENR	0.1750402	0.0061288
SLTM	0.1538253	0.0061605
LPIN2	-0.1597373	0.0062087
MRPS27	0.1188157	0.0062609
NDST1	0.0381856	0.0063166
GP1BB	0.2194229	0.0063358
GFM2	0.0539857	0.0063484
STX16	0.1522125	0.0064015
COMMD10	0.0991544	0.0064129
ZNF579	-0.1474571	0.0064522
MFSD10	-0.156113	0.0064989
CLDND2	-0.1435532	0.0065128
AC026401.3	-0.1073895	0.006547
ING1	0.0347684	0.0065635
PDE3B	0.1241734	0.0065716
IFIH1	0.1531671	0.006582
ZNF808	0.0488085	0.0065938
TAF15	0.2203897	0.0066011
GAB3	0.0097283	0.0066108
SIGMAR1	0.1035768	0.0066301
RFT1	0.0821442	0.0066661
NLRP1	-0.1180998	0.0066732
CLASP2	0.0661244	0.0066776
GPR180	0.0716594	0.0066891
MYLIP	0.0363012	0.0066897
CLK2	0.0515425	0.0067861
CPVL	0.1445757	0.0069236
HIST1H2AK	0.0660682	0.0069388

ZNF814	0.1291713	0.0070257
RFNG	0.1122713	0.0070375
DNAJB14	-0.2000307	0.0070414
CDC14A	-0.186763	0.0070503
DERL2	-0.1737426	0.0070517
ELOA	0.0272877	0.0070938
ATPSCKMT	0.0785382	0.007102
MIPEP	0.0545947	0.00711
MAEL	0.0431132	0.0071103
SNRPA	-0.182458	0.0071293
ERG28	0.0062023	0.0071402
TRAPPC2L	0.1133327	0.007148
STK35	-0.0673554	0.0071572
PMM2	0.104287	0.0071626
TRANK1	0.1815433	0.0072174
WDR43	0.2230064	0.0072316
ZNF785	0.0864947	0.0072834
MPV17L	0.0917177	0.0073126
ADSL	0.1352286	0.0073211
HDAC6	0.0390211	0.0073717
HIBCH	0.121858	0.0073946
SLC7A5	-0.1843363	0.0074249
UBE3C	0.0922342	0.0074357
PSMC1	-0.1473208	0.007533
UXT	0.1286033	0.0075571
HMGN2	0.1146379	0.0075915
ZYG11B	0.0808784	0.0076103
ZNF618	0.0927056	0.0076211
DSE	-0.0365118	0.0076546
RABL6	0.1120334	0.007677
EIF2B1	0.1343655	0.0077066
HLX	0.0850137	0.0077176
ZNF486	0.0966319	0.0077281
GID8	-0.0839655	0.0077347
SCARB2	-0.1869615	0.007771
GLMN	0.1500583	0.0078124
AQR	0.1117494	0.0078293
CPD	0.060845	0.0078372
EID1	-0.1509051	0.0079014
APMAP	0.147026	0.0079375
TRG-AS1	0.324661	0.0079811
ZC3H12C	0.0660976	0.0079863
LINC01515	0.061658	0.0080255
G2E3	0.0761735	0.0080483
NADK2	0.0917713	0.0080776
AC016747.1	-0.0971591	0.0080817
B3GNTL1	0.133174	0.0081313
ANXA4	0.085132	0.0081679
GNS	0.1226803	0.0082251
SSX2IP	-0.131169	0.0082302

ZNF519	0.0768308	0.0082433
RDX	-0.1321132	0.0082927
TNKS2	-0.0886691	0.0082936
CBWD2	0.0417796	0.0083782
RNFT1	0.0457744	0.0085624
RNF111	-0.1295246	0.0085748
QSER1	0.0732104	0.0086104
C12orf66	0.08965	0.0086133
PSTPIP1	-0.1215963	0.008638
AP000845.1	0.0601786	0.0086932
XRN2	0.22106	0.0087107
GGA2	0.1120036	0.0087151
DHFR2	0.0623878	0.0087426
CAMKK2	0.1315578	0.0088737
NXT1	-0.0856653	0.00888
SLC15A4	-0.1216244	0.0089784
KCNMB3	0.070841	0.00898
C1orf174	0.0100858	0.0089978
SLC19A1	0.0815584	0.0090087
STBD1	-0.0645166	0.0091295
AMMECR1	0.0323194	0.0091365
TMEM101	0.0906953	0.0092575
LRRC28	0.0555955	0.0092666
PXK	0.1208591	0.0093221
KDELR2	-0.1015979	0.0093546
OAS2	0.0970783	0.0093709
YES1	-0.1432192	0.0093866
GET1	-0.1322638	0.0093976
HMOX2	-0.0887428	0.0094361
KLHL8	0.0834364	0.0094738
HIST1H2AE	-0.1198362	0.0094995
MINPP1	0.0728169	0.0096315
EMC4	0.042479	0.0096924
VGLL4	-0.145983	0.0097077
COL9A2	0.0558436	0.0097136
ANAPC1	0.0802311	0.0097551
AC092944.1	0.0653648	0.0097671
METRNL	-0.1465929	0.0098188
MEIS1	-0.2126705	0.0098536
SNRNP27	0.1025989	0.0098633
WASHC2C	0.0994665	0.0099637
ZC3H14	0.0870934	0.0099773
PDCD7	0.2001435	0.0100127
FNTA	0.0345331	0.0100335
CCDC77	0.0392827	0.0101641
DYRK3	-0.1837238	0.010194
RBPMS	-0.1427809	0.0102174
CEP128	0.0692464	0.0102518
ZNF783	0.0403346	0.0102791
UTF1	-0.1862351	0.0103283

LINC01857	0.0704649	0.0104144
HELQ	0.0806064	0.0104457
KIF16B	0.0536268	0.0105631
RC3H2	-0.237308	0.0106531
CDC34	-0.1247962	0.010676
MRPL16	0.1303122	0.0106883
CCL28	0.0800877	0.0107093
DRAP1	-0.1888922	0.0107415
TMEM67	-0.0866656	0.010821
NOA1	0.0955877	0.010826
LINC01138	0.0357472	0.0108622
MRPL36	0.1408272	0.0109271
MAP10	0.0760426	0.0110499
CETN3	0.1008105	0.0110721
PAFAH1B3	0.0757429	0.011087
WDHD1	0.0715835	0.0110941
INPP4A	-0.0656277	0.0111109
EIF3G	-0.1794407	0.0111211
TSNAX	0.1314498	0.0111612
IL3RA	-0.2004855	0.0111754
AP2M1	-0.1128645	0.0111865
WDR61	0.1555594	0.011208
CD109	-0.1619754	0.0112591
TOR1AIP1	0.103599	0.0112822
CDKN1C	-0.2409266	0.0113087
STIM1	0.027677	0.0113311
CABLES1	0.0672081	0.0113775
PSAP	0.0602057	0.0113979
RNF38	-0.177729	0.011441
TIMM10B	-0.1425671	0.0115463
C8orf33	0.1176476	0.0116005
FOXK1	-0.0609694	0.0116521
ARCN1	0.0129665	0.0116807
ADAMDEC1	0.0893612	0.0117159
ATP5F1B	-0.1461697	0.0117825
DIS3L	0.1270891	0.0118704
CERS2	0.1101146	0.0119263
ZFP28	-0.0714203	0.0119314
METTL2B	0.088864	0.0119612
ZNF346	0.0561294	0.0119749
REV3L	-0.1243999	0.0120151
ABRAXAS2	-0.1098536	0.0120175
UBE3A	-0.1402254	0.0120849
ZNF720	0.140028	0.0122383
COQ8A	0.1147609	0.0122636
TESK1	-0.1018758	0.0122838
METTL25	0.0639216	0.0123803
SF3B2	0.1606133	0.0124769
TRAPPC12	0.0994884	0.0125586
PPHLN1	-0.1698037	0.0125733

NUP107	0.1071877	0.0126198
GRK4	0.0358718	0.0126814
ARHGAP11A	0.1010764	0.0126821
PSMC6	-0.1213212	0.0129523
TMEM42	0.154367	0.0130173
YIPF6	-0.0736453	0.013064
GNAQ	0.2287593	0.0131383
RCAN3	-0.1259968	0.0131682
RIOK2	0.0522085	0.0131914
SLC12A7	-0.1299157	0.0131967
FANCF	0.0979969	0.0132092
CD300LF	-0.110761	0.0132099
ITFG2	0.0940497	0.0132434
TEC	0.1618164	0.0132576
SNRPG	-0.0987169	0.0133832
ZFAND5	0.042777	0.0134107
MPZL3	-0.0616891	0.0134237
ZNF766	0.1194508	0.0134641
COIL	0.1169238	0.013494
SPTSSA	0.1151227	0.0135419
ITPKC	-0.0602547	0.0135592
ENGASE	0.0289709	0.0135809
SLC44A2	-0.086838	0.0136643
NKRF	0.0217075	0.0136822
PPP2R5A	0.0684766	0.013709
VPS72	0.0881568	0.0137217
GSTM2	0.0557362	0.0137524
NSMCE4A	0.0516156	0.0137825
NDFIP1	-0.1592119	0.0138008
AC007038.1	0.0522956	0.0138164
PLCL2	0.0451953	0.0138981
CAPN10	-0.0576383	0.0139023
HOXA6	0.1031035	0.0139405
COX17	0.0724021	0.0139505
MBD3	0.0748646	0.0139794
MMP2	0.1547703	0.0140364
CAAP1	0.1306095	0.0140558
AL138756.1	0.0591167	0.0140705
KMT2A	-0.1227241	0.0140901
CAVIN2	0.1137873	0.0141219
PCGF3	-0.1207073	0.0141627
LINC01534	0.031521	0.0142178
CAPRIN2	0.0145162	0.0143234
COLGALT1	0.1074012	0.0143286
L3MBTL3	0.0660832	0.0143515
RAD51-AS1	0.156332	0.014406
GM2A	0.0963838	0.0144352
AC093673.1	-0.1216743	0.0144834
UBN2	0.1013746	0.0144882
DDX17	0.1729504	0.0145249

PLD6	0.0559261	0.0146315
PLEKHM1	-0.154821	0.0146704
PDGFC	-0.0386383	0.0147736
CDC26	0.1010541	0.0148025
TMEM230	0.1629307	0.0148514
SNAP23	0.1740988	0.0148595
IDH2	-0.1443716	0.0149123
PAGR1	0.0391662	0.0149745
GPAT3	-0.1862795	0.0149869
CDK11A	0.0855437	0.0150725
TRIM66	0.0537426	0.0151367
RNF135	0.0770201	0.0151703
ACAD9	0.053092	0.0152427
CPEB2	-0.0786358	0.0153733
EFCAB14	0.1213534	0.0154755
AC005072.1	-0.0862938	0.0154785
SLC35B1	-0.1489118	0.0154967
AC092428.1	-0.0566741	0.015521
AL356599.1	0.0859854	0.0155224
TSN	0.0738401	0.0155674
PTEN	0.1930203	0.0155775
ARL16	-0.0272046	0.0155874
LINC00476	0.077812	0.0155924
SLC4A7	0.0957699	0.0156974
TMEM150A	0.0773215	0.0156984
NUDCD3	0.0927581	0.0157015
KCTD10	-0.128354	0.0159086
AL513128.2	0.0795164	0.0159335
BNIP3	0.0758173	0.0159358
INTS2	0.0782858	0.0159571
SLFN12	0.04649	0.0161356
RPS6KA4	-0.1287023	0.0164273
PITRM1	0.0664747	0.0164937
TYROBP	0.2269899	0.0165839
ARHGAP23	-0.1124073	0.0165928
PACC1	0.0459582	0.0166175
MEF2C-AS1	0.0847168	0.0166379
MAX	-0.1712161	0.0166787
HACD4	0.1334326	0.0167364
EPM2AIP1	0.2051318	0.0167439
ADAR	-0.0647423	0.0167903
TYW1B	0.0393693	0.0169532
ATP1B1	0.015423	0.017018
NUP214	0.1034076	0.0170356
ZNF124	0.0677307	0.0170951
ACBD6	0.1722571	0.0172731
FAR1	-0.1701257	0.0173382
AC008014.1	0.0667244	0.017352
BCL9L	-0.1526792	0.0173554
IL2RA	-0.240817	0.0174558

ACOT11	0.1407137	0.0175038
MRPS18A	-0.1307165	0.0175457
THADA	0.1272314	0.0176232
YAE1	0.0519267	0.0176742
TRIB3	-0.125275	0.0176914
PRKAB1	-0.0993694	0.017709
XPOT	0.1660971	0.0179228
GDI2	0.1659824	0.017924
SMIM29	-0.1122428	0.018069
NDUFS7	0.164842	0.0181176
FCGRT	0.084388	0.0181285
FOCAD	0.0497941	0.0181459
ZFX	0.109904	0.0182273
KIF18A	-0.1135951	0.0182699
HELZ	-0.1586211	0.0183373
ZNF3	-0.1350443	0.0184916
ZBTB8A	-0.1745373	0.018506
API5	0.0949067	0.0187928
KLF16	-0.1502638	0.0189661
PLEKHG2	-0.0573349	0.0190938
PURB	-0.0673904	0.0191077
SNHG8	0.1835794	0.0191692
NISCH	0.0359269	0.0193344
STAU1	-0.1544734	0.0193427
PRELID2	0.0696383	0.0195908
SLC25A39	-0.0769364	0.0196215
INTS3	0.0487269	0.0196688
UBE2Q1	0.175597	0.0196699
HIPK2	-0.184274	0.0198276
RACK1	0.1332418	0.0200901
TRIQK	0.1010673	0.0201617
SF3A2	-0.1522929	0.0201699
TARS2	0.0941045	0.0202825
POLK	-0.1468443	0.0203594
CNOT7	0.1020437	0.0203818
FUNDC1	0.1480726	0.0205179
CNOT9	-0.1530493	0.020555
GATC	0.0823588	0.0205698
ENTPD5	0.0404134	0.0205721
ATP6AP2	0.0447909	0.0205779
B3GALNT1	0.0816675	0.0206902
RAB44	0.0576305	0.0206966
BUB3	0.1054819	0.0207874
BCL2A1	-0.0702103	0.0208999
TCTEX1D1	0.0567719	0.0209252
ATP6V1B2	-0.1201885	0.0210604
FNBP4	-0.2043556	0.0211031
DGCR6L	0.1149135	0.0212172
DTWD2	0.0591115	0.0212261
AL132709.8	0.0682517	0.0212939

USP33	0.1194859	0.0213393
SND1-IT1	0.0385767	0.0214129
ZFP36L2	0.2365662	0.021417
AL157392.3	0.0592472	0.021585
EEPD1	-0.1443587	0.0216149
HSPBAP1	0.0137364	0.0216861
GABPB1	-0.1154982	0.0217048
MAP7D3	0.1761597	0.0217422
EIF3J-DT	0.1377307	0.0218051
TTBK2	0.1210542	0.0221149
IKBKB	0.0139643	0.0223028
ACLY	0.1444142	0.022451
NOP2	0.0848685	0.0225155
RITA1	0.0868425	0.0225496
GTPBP6	0.158647	0.0227127
ICK	0.0585478	0.0227532
ERICH1	0.1959594	0.0227871
TRIM5	0.1372623	0.0227952
TCF4	0.4116081	0.0228885
ANAPC13	0.156732	0.0230306
SPOUT1	0.075928	0.0230869
AC011462.5	-0.0715491	0.0232339
DBT	0.1071487	0.0234476
ABI2	-0.1082591	0.0237665
FAM133B	-0.21648	0.023806
TMEM260	0.11117	0.0239205
TMEM185A	-0.0947306	0.0240647
CBR4	0.0808436	0.0241731
FAM222B	-0.0679449	0.02418
XBP1	0.2607411	0.024356
MRPS17	0.1195138	0.0245082
MBD4	0.1291434	0.0245492
NRXN2	0.0664953	0.0245637
PAK1IP1	0.1268658	0.0246559
FAM172A	0.0871749	0.0247036
USP27X	-0.1062296	0.0248685
HDHD2	0.1382829	0.0249022
KYAT3	0.0591164	0.0249973
ELAC2	0.0886929	0.0250019
TRMT61A	0.0555948	0.0253563
EIF2S3	0.1944801	0.0254502
ZNF589	0.0487651	0.0254937
MPP7	-0.0909362	0.0255867
SLCO4A1	-0.0974745	0.0255898
PRELID3B	-0.114369	0.0255906
NIFK-AS1	0.1332869	0.0258494
KANSL1L	0.0884744	0.0262244
PDIA5	0.0501677	0.0264165
AC025171.2	0.1568665	0.0267979
KLHDC4	0.0499829	0.0273269

TMEM159	0.107712	0.0277523
ELK4	-0.0622843	0.0279273
ZFP90	0.088088	0.0279636
CTSS	0.2208352	0.0281647
RASA3	0.0813314	0.0281894
MTFR1	0.0510767	0.0282522
RPP21	0.1495624	0.0282905
LBHD1	0.0519168	0.0285204
CCDC58	0.0965548	0.0285605
TATDN2	-0.0696589	0.0285721
UQCRQ	0.1198418	0.0285722
TM7SF2	0.0859588	0.0286038
MMAB	0.1067545	0.0286387
UBR5	-0.2407148	0.0286403
ATP5F1A	0.1576025	0.0287279
BNIP3L	-0.1276376	0.0287391
TMEM182	0.0449901	0.028789
OGFOD3	0.0678588	0.0288395
ZNF70	0.0759179	0.0291575
ASB3	0.0554374	0.0292128
MFHAS1	0.111628	0.0292398
EPB41L2	0.0890261	0.0296249
RINL	0.0675814	0.030037
SUSD6	-0.1314325	0.0300428
ELL	-0.1252471	0.0300915
TMEM64	0.0597789	0.0301282
ANKRD26	0.1106664	0.0302404
ZFP14	0.1226123	0.0303356
MFNG	0.0060848	0.0303725
AC007686.3	-0.0540528	0.0304094
LAPTM4B	0.0772456	0.0304267
CCND2	0.1054738	0.0306047
CNOT1	-0.1825798	0.0306582
NFE2L3	0.1063475	0.0307369
DDX31	0.0732517	0.0307541
ATXN1L	-0.0980466	0.0309359
UBE2I	-0.2050833	0.0309415
CYB5D2	0.1039926	0.0309432
C19orf53	0.1790826	0.0311669
C9orf40	0.0566595	0.0311757
LRRC27	0.0560706	0.0312221
CCDC24	-0.0623828	0.0312888
RRP1B	0.2153582	0.0313891
ALG10B	0.0791272	0.0314497
ACTR10	-0.1345647	0.0315338
TMEM181	0.0521779	0.0317482
BRCA1	0.1024047	0.032075
TAGAP	-0.1899993	0.0325802
EEF2K	0.0943597	0.0329081
GTDC1	0.1056821	0.032927

TMEM147-A	0.0837209	0.0329913
GMIP	-0.1169763	0.0331266
CCNH	-0.0473371	0.0331898
UBXN2A	-0.1221892	0.0332316
PAN3-AS1	0.1205708	0.0332318
ZNF813	0.0585775	0.0332776
WDR4	0.0466574	0.0335343
CISD2	0.1149967	0.0335973
GKAP1	0.0738894	0.0336217
PPP3CC	-0.0949101	0.0336422
RBPJ	-0.1610885	0.0337464
PAK1	0.1347205	0.0338508
RPP38	-0.042768	0.0338585
SLC12A2	-0.0657299	0.0338645
EIF5A	-0.1861167	0.0338699
EFCAB11	0.0597533	0.0340245
FIBP	0.0485864	0.0341671
ZNF71	-0.061858	0.0345767
CHRAC1	0.0771468	0.0348026
ZNF264	-0.1170148	0.0348229
REL	-0.1120645	0.0348415
TMEM219	0.0206783	0.0348524
SDHAF2	-0.1736396	0.0349358
TELO2	0.0235895	0.0349958
EEF2KMT	0.0796325	0.0350443
MT-ND4L	0.182665	0.035146
IRAK1BP1	0.0843786	0.0351761
TYW3	0.1326054	0.0351864
PIM2	0.1499877	0.0353565
SASS6	0.0945196	0.035433
SLC45A3	0.1134601	0.0359774
C16orf58	0.0435337	0.0360536
TRIM33	-0.1870217	0.0362808
RPA3	0.0647939	0.0363648
RPA1	0.1259427	0.0365499
AC124248.1	0.0646128	0.0365848
FER	0.0738129	0.037025
RPL14	0.0970676	0.0371257
TRAPPC6A	0.1293975	0.0373439
CALM3	-0.1598141	0.0373588
KATNA1	-0.1319259	0.0375746
CCDC9	-0.0819562	0.037854
SOX12	0.0632681	0.0381563
POFUT1	0.0548317	0.0381929
PAM	0.0422519	0.0385117
CRNDE	0.0689033	0.0386648
UBE2R2	-0.2238902	0.0388106
AC246817.2	0.0422278	0.0391567
ANTKMT	0.1440573	0.0392522
CEP97	0.0857662	0.0394703

AL021155.5	0.0440055	0.0394982
SPATS2	0.0987292	0.0395384
ARMC6	0.0883776	0.03954
LYPLA2	-0.0961845	0.0399665
IL2RG	-0.2203602	0.0400324
PHF13	-0.0723799	0.0400981
SRSF11	0.0965053	0.0402309
CCDC59	0.0851501	0.0402888
GSK3A	-0.166553	0.0402971
C10orf88	0.0576349	0.0406922
SAC3D1	0.1205379	0.0408776
MICAL1	-0.0722883	0.0409104
RRP8	0.02473	0.0411029
INSR	0.0906178	0.0411361
LYSMD2	0.0689686	0.0411674
HIST1H2BD	-0.1006796	0.0416057
ETNK1	0.0782034	0.041708
VPS13B	0.099033	0.0420012
EVI5	0.082196	0.0423676
SLC25A40	0.0663691	0.042387
DIDO1	-0.0609782	0.0427324
HACL1	0.0761296	0.0430048
TFB2M	0.0193612	0.0432574
AC138150.1	0.0885745	0.0435469
RCHY1	-0.1053694	0.043689
FAM214A	-0.092022	0.0438196
WDR33	-0.0412708	0.0444925
SIL1	-0.1015569	0.0445677
C15orf40	0.0921933	0.0445914
ZBTB6	0.0783601	0.044632
SMS	0.1860692	0.0446399
SH2D3C	-0.1715775	0.044892
AKR1A1	0.1027138	0.0449493
DMXL1	0.1014342	0.0450809
ZFP91	-0.1865978	0.0452481
TNRC6A	-0.0680799	0.0452587
TMTC2	0.0617322	0.0453058
WASHC2A	0.0063382	0.0458973
HPS3	0.1122954	0.0460553
MED30	0.0505816	0.0464011
TRMT1L	0.0763271	0.0465092
MX2	0.0864022	0.046756
IMMP1L	0.1056371	0.0468023
NACC1	-0.1023427	0.0468885
NDUFAF1	0.0605762	0.0469829
C9orf43	0.0975665	0.0470922
HEXB	0.0989125	0.0471005
NCOA7	0.0957446	0.0471491
PAK4	-0.0271194	0.0474312
UHRF2	-0.1263953	0.0477332

PNPLA4	0.1123022	0.0477581
IFT52	0.037828	0.0479072
EDEM1	-0.0965931	0.0480701
FIG4	0.0635	0.0482627
DENND2D	0.0444447	0.0488842
ZFAND2A	-0.179496	0.0489679
RABL3	-0.0796442	0.0490525
RAB11FIP2	0.0735807	0.0492606
RUNX1	0.2470236	0.0493363
ANPEP	0.1591197	0.0493695
STX8	0.110655	0.0494203
MRPS18C	0.1206681	0.049425
TRIM14	0.0991789	0.0497631
TRIAP1	0.0962675	0.0502552
NEK7	0.0576955	0.0502842
MTRF1L	-0.1820135	0.0504151
GLCCI1	0.0595582	0.0504327
RGS14	-0.1371826	0.0505618
L3MBTL2	0.0765585	0.0505625
XYLT1	0.1158956	0.0505766
TCP1	-0.1821545	0.050736
CERK	-0.1156576	0.0509471
ANGEL2	0.1266611	0.0510224
TECR	0.0873355	0.0511858
DPY19L1	0.0605193	0.0512136
EEF1AKMT3	0.057384	0.0514357
POFUT2	-0.0955808	0.0516198
FCF1	0.069164	0.0517228
MRPL45	0.1056101	0.0517946
FBXO3	-0.0831219	0.0520559
METTL4	0.1039585	0.0520877
IMMT	0.1180592	0.0525572
GYG1	0.0297249	0.0526887
HESX1	0.068275	0.0527131
TRIM4	0.0947949	0.0527693
C12orf10	-0.0691378	0.0528657
TNIP2	-0.1342468	0.0528923
C2orf74	0.0897064	0.0531265
HDAC8	0.1280674	0.0531503
COQ7	0.0894278	0.0531632
PCNA	0.1801242	0.0532637
FERMT3	-0.1266646	0.0533806
PLAAT4	-0.0791249	0.0540153
BRD4	-0.109371	0.054439
MRPS6	-0.0397096	0.0546278
MCTP2	0.0136226	0.0551214
COMMD3	0.07778	0.0552241
AC027097.1	0.064921	0.0553189
LINC01278	0.104016	0.0555108
ERCC2	0.0559862	0.0555557

GPN2	-0.0439058	0.0556288
ATG4B	-0.065273	0.0558025
BLMH	0.084197	0.0558657
CTSF	0.0710961	0.0563144
CDC5L	-0.053503	0.0568178
ZBTB8OS	0.1034234	0.0568559
HNRNPC	0.1084072	0.0570231
EEA1	0.2009095	0.0574205
FLOT2	-0.0083178	0.0580717
ZNF626	0.0876378	0.0580893
STX10	0.1269892	0.058221
SLC11A2	0.0262408	0.0582962
CHMP4B	-0.1630046	0.0584002
KLF3-AS1	-0.1441796	0.0587543
YBX1	-0.1278328	0.0588788
HNRNPUL2	0.1544468	0.0589076
FOPNL	0.1448988	0.0595268
CZIB	0.1254288	0.0597758
TERF1	0.1189587	0.0606266
NCOA5	0.0492268	0.0607934
TMEM256	0.1587694	0.060827
GPRC5D-AS	0.0589343	0.0610127
SMIM10L1	0.1097612	0.0612343
HIVEP2	-0.1900881	0.0613602
SS18	-0.189547	0.0616245
HDAC1	0.198623	0.0623272
CCAR1	-0.1171617	0.0626246
RIC8A	-0.115775	0.062689
STARD7	0.1105006	0.0626979
SNHG16	0.1677444	0.063063
RPS7	0.079689	0.0634501
SMARCA2	0.07913	0.0644027
C1orf52	-0.1348655	0.06474
STRN4	-0.1259389	0.0650123
ZBTB2	-0.0909088	0.06516
CBFA2T2	0.0542538	0.0654937
TCN2	-0.0726987	0.0655498
PSMB8	0.2152561	0.0670528
KPNA2	-0.1198417	0.067162
ENG	-0.0894464	0.0674241
CD63	-0.1418739	0.0676678
VKORC1L1	0.080957	0.0676823
RBL1	0.0581234	0.0677095
RBM15B	-0.103577	0.0677174
SDF2L1	0.0430923	0.0679982
MPI	0.0790751	0.0681114
SRRM2	0.1734346	0.0682419
SLC2A4RG	0.1272809	0.0684472
ITCH	-0.1852544	0.0687332
MTX1	-0.1223797	0.0693677

CHPT1	0.0899491	0.0693978
FOXN2	-0.1602569	0.0694022
UBA5	0.1371695	0.0700476
SDE2	-0.1486733	0.0704671
INTS6-AS1	0.0573082	0.0705356
MRPL14	0.1071203	0.0710428
ZNF234	0.0733516	0.0711267
GRINA	0.0289851	0.0713011
ABCA5	0.0542625	0.0713116
GLRX5	0.0671671	0.0714082
LTBR	-0.1744021	0.0714115
CUX1	0.0960812	0.0715764
TPGS1	0.0992729	0.0716805
RPS24	0.0928051	0.0717539
ATP11C	0.0755066	0.0717663
ERCC1	-0.0439661	0.0720855
TMEM86B	-0.0734289	0.0723358
DESI2	-0.0522704	0.0723966
U2AF1L4	-0.0577084	0.0724999
PELI1	-0.1148506	0.0727207
SEPTIN2	-0.0962738	0.073205
YOD1	0.0460433	0.0732134
CYFIP1	0.1479818	0.0732409
SRP9	0.1722066	0.0740731
IQSEC1	-0.0764997	0.0744579
TMEM177	0.0821326	0.0747335
TCEA1	0.0801048	0.0748039
HCCS	-0.1243287	0.0748826
AL592183.1	0.0540727	0.0749615
CMTM2	-0.1111038	0.0751896
DENND11	0.0589679	0.0752532
CPT1A	0.0850301	0.0753679
NUP37	0.1045078	0.0754032
DOCK11	0.0784894	0.0756763
GSPT1	0.2075273	0.0759493
HOXB3	-0.131739	0.0760485
HYAL2	0.0562258	0.0761991
B3GALNT2	0.0805322	0.0762655
ELMO2	-0.0834048	0.0763659
TMEM50B	-0.0918234	0.0764137
HGS	-0.1543088	0.0765662
MLLT11	-0.1074953	0.0771244
HNRNPA1P4	-0.1730863	0.0774813
ST3GAL5	-0.1113112	0.0779639
RLIM	-0.2250128	0.0783288
TMEM19	0.0496138	0.078344
SARNP	-0.1243944	0.0787505
PARPBP	0.0373913	0.0788988
RNF19A	-0.2188123	0.0789783
NSMAF	0.0532151	0.0791997

BCAP31	-0.1255348	0.0795532
AGO1	0.0306669	0.0796066
FAM104B	0.061969	0.0797918
B4GALT3	-0.0860049	0.0808213
ZSCAN30	0.1237064	0.0811581
TOM1	-0.0941321	0.0812117
TMEM144	0.0957325	0.0813336
CHID1	0.1362083	0.0820213
PARVG	0.1056637	0.082699
WAC	-0.1919563	0.0828612
SLC50A1	-0.1279294	0.0829637
CASP1	0.0756155	0.083157
AC132192.2	0.0283159	0.0835116
SNRPA1	-0.1826785	0.083836
ANAPC7	0.1079881	0.0839205
TASOR2	0.0978103	0.0840237
SUCLG1	0.1717622	0.0843323
AC018638.7	0.0511506	0.0844515
ZSCAN5A	0.05998	0.0846894
ANKRD10-IT	0.0633922	0.0851372
IFT122	0.0613132	0.0856135
SMURF1	-0.1133664	0.0857102
SMC6	0.143105	0.0859746
OSBP	-0.1204229	0.0860829
ZBTB25	0.0110415	0.0864598
PCCA	0.0447329	0.0869504
PLEKHA3	0.0598721	0.0879117
PCGF6	0.0533413	0.0880746
SESN1	-0.1920392	0.0891386
OXNAD1	0.0552162	0.0895276
MAP1A	0.2834097	0.0898102
LUC7L	-0.0602493	0.0899298
ALDOC	0.058581	0.0900381
TSPAN5	0.0658056	0.0902847
CSNK1E	-0.0696555	0.0904559
CDK17	-0.1394306	0.0906154
NAB1	-0.1451019	0.0912884
EDRF1	0.0469441	0.0914236
SPIRE1	0.0992406	0.0916959
MED31	-0.0969346	0.0923451
AL513550.1	0.0519715	0.0924404
ZMYND8	0.1367926	0.0925423
KANSL3	-0.1166707	0.0946177
FBXW7	-0.1371842	0.0951557
IKZF4	0.0606934	0.0952672
SLFN11	0.0954623	0.0952806
COPS9	0.1677865	0.095299
SLC35E2A	0.0948752	0.0957599
OBSCN	0.0859324	0.0958412
LENG1	-0.0703288	0.0968376

VAC14	0.0381831	0.0972011
ATF7IP	0.1138441	0.0973448
MARVELD1	0.0787749	0.0974318
ZNF665	0.0839826	0.0975398
CU638689.4	0.0590573	0.0976284
NUDT12	0.042965	0.0978121
MAGEH1	0.0410336	0.0982402
TMEM265	0.044802	0.0984543
ITPR2	0.2164228	0.0992134
MLC1	-0.1014337	0.0996749
RBL2	0.0955076	0.1003886
ZNF880	-0.0964912	0.1003973
EMC1	0.0570847	0.1017537
ALYREF	0.1294818	0.1030896
TRIM38	0.1715725	0.1031528
CTSG	0.2588083	0.1038304
ZNF354A	0.0889049	0.1039348
PPP2R5D	0.0374216	0.1040134
NRDE2	0.0543221	0.1040212
ECSIT	0.0450891	0.1041609
DOCK5	-0.1830964	0.1041733
AARS2	0.0549448	0.1042452
FDPS	0.1026941	0.1046038
VPS50	0.0407402	0.1046057
PSMC5	0.0835505	0.1050019
AC027644.3	-0.0436908	0.1053978
FOXN3	-0.1466056	0.1055992
HAGH	0.0830097	0.1056398
FAHD2B	0.0566341	0.1056806
CCDC138	0.0515658	0.105765
RSRP1	0.2458577	0.1059308
MED25	-0.1433063	0.1061188
GDI1	-0.1211969	0.1062495
PUS3	0.0795409	0.1063694
ARHGDIB	-0.164514	0.1063748
PTK2B	-0.0923955	0.106819
SLC35A2	-0.0694809	0.1071637
SDCBP2-AS	0.0299156	0.1074771
TRIO	0.0561669	0.1075783
WDR45B	-0.0954003	0.1080035
DET1	0.0477987	0.1085378
ZBED5-AS1	0.0757817	0.1088178
CNOT11	-0.0033897	0.1089998
TMEM11	-0.0907418	0.1102552
SRSF4	-0.172211	0.110279
ZSWIM4	-0.0831642	0.1106585
URM1	-0.1133253	0.1110059
OPA1	-0.145118	0.1110586
MTOR	0.0770956	0.1111754
TMEM51	-0.096141	0.1114421

AC084198.2	0.0519099	0.1117042
UBXN1	-0.1090645	0.1117676
AL033543.1	-0.0701132	0.1124621
SCO2	-0.0831608	0.1124879
IFI27L2	0.1617657	0.1129858
FBXO30	-0.100074	0.1130027
PHACTR1	0.1099802	0.1132567
METTL18	0.1198204	0.1136397
FUT7	-0.1181029	0.1137345
MAPK8	-0.131262	0.1140642
VPS29	-0.0445892	0.1140761
RRP1	0.1263241	0.1142406
C11orf49	0.0506665	0.1144924
MRPS10	0.0991834	0.1145207
SNRNP70	0.0810443	0.1146064
ZNF44	-0.1189562	0.1149485
DYNC1LI2	-0.1389994	0.1150468
ARHGAP45	-0.1164418	0.1157486
MSMO1	-0.1498584	0.1158564
CDK11B	0.0338178	0.1159959
CNOT6L	0.0192162	0.1163242
GHDC	0.0947239	0.1174433
RAB11A	-0.1577646	0.1174615
NOSIP	0.0526555	0.1177026
HTRA3	0.0561554	0.1183491
CDK14	0.0701866	0.1185808
SLC48A1	-0.0615902	0.1188436
CNIH1	-0.1604966	0.1189068
ZBTB47	0.033169	0.1194306
ECI1	0.0543464	0.1194601
CRY2	-0.0649109	0.1197053
RRAGB	0.0886827	0.1197514
CRELD2	-0.1013731	0.119813
UNK	-0.0912611	0.1212723
PRKACA	0.0388865	0.1225016
RAD51C	0.090736	0.1232604
ATF2	-0.1259588	0.123422
FIGNL1	0.064044	0.1237441
ZDHHC14	0.0713096	0.1239106
MGST3	-0.0281434	0.1248104
CMPK2	0.0679879	0.1254458
SFT2D1	-0.0353657	0.12578
GOLGA8A	0.0682138	0.126404
HSPB11	0.1337687	0.127047
MT-ND2	0.0975846	0.1273174
DHRSX	0.0776708	0.1281389
LINC01150	0.0583261	0.1286531
EIF1AD	-0.1034655	0.1286694
LONP2	0.1102436	0.1289118
ACAP1	-0.0508767	0.1292858

ABHD8	0.0533319	0.1293169
HMBS	0.0714561	0.1293872
MARCH5	-0.0977454	0.1295661
SULF2	-0.0732209	0.1295885
MTCH1	-0.1897142	0.1295985
SNTB1	0.0824378	0.130101
BUD13	-0.0862611	0.1310789
LINC00667	0.125216	0.1313852
UGGT1	0.1082248	0.1315177
RPS26	0.1497818	0.1335692
SPACA9	-0.1003718	0.1338541
RMDN1	0.1092577	0.1351994
CEBPZOS	0.1271532	0.1352482
S100PBP	0.095147	0.1352818
CENPV	0.0749559	0.1352932
TTC28	0.0183212	0.1355082
JPX	0.1819086	0.1366718
IRAK1	-0.0396167	0.1371228
ZGPAT	0.0036726	0.1373895
RPS12	0.0863358	0.1388155
PEX11A	0.0285676	0.1390027
FAHD2A	0.0804026	0.13953
GBP4	0.0963571	0.1399444
ZNF165	-0.0920084	0.1402129
TMEM184B	-0.0764229	0.1403158
ZKSCAN1	0.1072511	0.140559
NAP1L4	0.0847122	0.1407144
NR2C1	-0.0394491	0.1417187
C3orf38	0.0139536	0.1419404
BTBD6	0.0477885	0.1425304
MED4	-0.1473742	0.1431665
KDM5C	-0.074784	0.1432826
POGK	0.0578029	0.1438578
USF2	0.1534042	0.1444958
GSTM3	0.0487721	0.1450539
PHF21A	0.0710685	0.1451882
SLC25A11	0.1117418	0.1469228
ZBTB4	-0.1250893	0.1469493
VRK1	0.1002917	0.1471537
NCBP3	-0.162325	0.1484987
NXPE3	0.0378196	0.1493283
ZDHHC3	0.018405	0.1507713
ZBED3	0.0883387	0.1509799
ACTR3	-0.1492887	0.1518748
PPP1R8	-0.0609035	0.1524371
ABCF2	0.0984651	0.152503
REEP4	-0.0629299	0.1534064
PSMB7	-0.0604358	0.1540317
SENP2	-0.0764988	0.156066
HIGD2A	0.0970371	0.1563625

MZT2A	0.048021	0.1564555
DEGS2	0.0468738	0.1565538
USP53	-0.0423889	0.1566385
RNF41	-0.1377272	0.1571165
CLN6	0.0868237	0.1574834
BMS1	0.1066998	0.1579544
DRAM2	0.1373438	0.1585981
SOCS7	0.0437169	0.1593867
S100A8	0.0616839	0.1595426
HOXB5	-0.1252126	0.1596671
AK5	0.0953528	0.1605243
H6PD	0.0625814	0.1607247
P4HB	-0.1149976	0.1616737
SERPINF1	-0.0863591	0.1616821
NOL12	0.0410979	0.1618119
MON1B	0.0860681	0.1621556
KCNQ1OT1	-0.3247818	0.1621679
SRI	0.0758686	0.1633678
ERBIN	-0.1537888	0.1647042
ZNF337-AS1	0.0440508	0.1651648
RFX1	-0.1105087	0.1653768
GCN1	0.1047368	0.1654321
ZFP64	0.0366989	0.1657125
CREBL2	0.0439874	0.1659749
PIAS4	-0.1144139	0.1663295
KCTD21-AS1	0.0530893	0.1667424
KIAA2013	0.0935466	0.167236
CUEDC2	0.1028102	0.1672771
AC074117.1	0.0490853	0.1674903
PLD3	-0.109255	0.1678456
ZNF296	-0.0756565	0.1682413
MAP4K4	0.0949176	0.1694028
BTN3A3	0.0414649	0.1696525
RTF1	-0.0373603	0.1701534
MLYCD	0.0701883	0.1706402
CLNK	0.0741294	0.1712499
CSE1L	0.0773061	0.1716732
SLC20A2	0.0808329	0.1724306
CTBP2	-0.1205982	0.1726327
EVL	-0.1031891	0.1726719
LTK	-0.0785356	0.1732345
NOTCH2	0.0774346	0.1739062
PACRGL	0.0579178	0.1740566
AC245014.3	0.0652591	0.1742689
CCDC137	0.0098089	0.1743513
SLC38A7	0.0516043	0.1752877
PGBD1	0.0142351	0.1756451
SCAF8	-0.088383	0.1758461
LAIR1	0.1555327	0.1760744
MRPL30	0.1099354	0.1762051

BAG1	0.1188182	0.1762612
ATXN2L	-0.1057501	0.1763062
NEMP2	0.0477658	0.1769109
CEP76	0.0513792	0.1769844
FLOT1	-0.1831365	0.1771841
PRCP	0.0021456	0.1778998
PI4KB	-0.08482	0.1787809
CASZ1	-0.0608549	0.178799
C21orf91	0.0990241	0.1789876
PDS5B	0.0321041	0.1794275
UBE2E1	-0.048096	0.1794524
RGS3	-0.1465316	0.1798564
RRP15	0.1294409	0.1802751
NABP2	0.0958173	0.1804084
MAD2L1	0.1279645	0.1806207
RSF1	-0.1426227	0.1807959
EDC4	-0.0693333	0.1811967
CFAP298	0.0536429	0.1815322
IQCB1	-0.0925111	0.182054
CLTC	-0.1714714	0.1822509
TDP1	0.0437031	0.183359
CPXM1	-0.1064298	0.1837166
COX11	0.1195914	0.1842578
C1RL	0.1292477	0.184718
LINC01341	0.0676315	0.1847503
VAPB	-0.0852139	0.1848879
ASF1A	0.0670229	0.1849614
WDR20	-0.1040372	0.1871481
TBKBP1	-0.0785246	0.1875814
PSMA3-AS1	0.0933098	0.1884194
MR1	0.0828609	0.1889967
GUF1	0.078976	0.1891502
GTPBP3	0.0789358	0.1896933
DPM2	0.119856	0.1901118
SETD1B	-0.0712679	0.190679
CCNL2	-0.1519942	0.1908271
SLC25A5	0.1400166	0.1911564
RASSF1	0.0143879	0.1920439
GLO1	0.1505173	0.1926623
AC073389.1	0.0380666	0.1927883
MRTFB	0.029958	0.1931103
SLC4A1AP	0.0761253	0.1953608
KIAA1586	0.0601665	0.1955911
NCAPD3	0.123188	0.1961281
IFT172	0.0504246	0.1963637
FANCL	0.0693236	0.1977595
C16orf87	0.0602479	0.1986655
SUSD1	0.1193944	0.1995958
SLC22A5	0.0469419	0.1997649
UBN1	-0.0238016	0.2004359

ARHGEF1	-0.1502886	0.2012725
AP2S1	0.1265979	0.2014515
MRS2	0.0549746	0.2016101
SMAP1	0.073453	0.2021386
LIG3	0.092151	0.2029081
STEAP3	0.0357465	0.2046788
PSME4	-0.1847444	0.205414
DUSP12	-0.0714944	0.206624
ANP32B	0.1532862	0.206892
APOC2	0.1850513	0.208083
TMEM245	0.1255954	0.208449
TMEM203	0.0732325	0.2090829
ADRB2	0.1059799	0.2094783
DHX32	0.0915674	0.2100955
CSTF2T	0.0594537	0.2101597
KCTD20	0.0510545	0.2107605
INCENP	0.0515401	0.2107649
FAS	-0.1153277	0.2114322
EZH2	-0.1367355	0.211468
MAP4K3-DT	0.1315114	0.2118762
CEP350	-0.1520876	0.2123601
TRNAU1AP	-0.0571148	0.212605
NGDN	-0.0665143	0.2130596
MOAP1	-0.0656302	0.2132101
HCP5	0.0346092	0.2134543
PRPF31	0.0921768	0.214062
TNPO1	0.0071002	0.214528
TAMM41	0.0405328	0.215086
ETV6	0.2051531	0.2152833
AP3B1	0.1433605	0.2158085
BCAT2	0.0813054	0.2169092
DDOST	-0.0567121	0.217993
IGFLR1	0.1592641	0.2182615
A1BG	-0.1049522	0.2186304
TBXAS1	0.1067066	0.2187475
CXCL8	-0.4552325	0.2189007
FAM110A	-0.0904462	0.21951
ANKRD17	0.1820875	0.2211901
NAF1	-0.0183002	0.2215713
SLC39A7	-0.0046638	0.2218234
CCNK	-0.1438405	0.2222583
HNRNPA2B1	0.1611709	0.2235064
LAPTM4A	-0.1111511	0.2258914
ZC3HAV1L	0.0623672	0.2259386
MRFAP1	-0.1045891	0.2265683
PCBD2	0.100446	0.2280105
AC058791.1	-0.1132732	0.2289109
PMS2	0.0886541	0.2295639
EXOC3	0.0482452	0.2310908
AC243960.1	0.0899632	0.2311592

ITGB7	-0.1061614	0.2319315
MAK16	0.0630497	0.234779
DNTTIP2	0.038532	0.2351885
ASXL1	0.1224602	0.2365506
AC242426.2	0.0512266	0.2367939
CCNT2	-0.1175572	0.2370932
AC010883.3	0.0354971	0.2374717
PEMT	0.0885164	0.2382902
LATS1	0.0134289	0.2390343
YTHDC2	-0.0289052	0.2394095
ZNF652	0.1161224	0.2394851
RUVBL2	0.1109175	0.2408581
PRKAR2A	-0.097228	0.2434474
ERCC4	0.0933692	0.243808
ZFAND1	0.1433707	0.2440451
AP006621.3	0.0697856	0.2441532
SNAPC4	0.0464121	0.2444796
MRPL57	-0.1991187	0.2461303
STYXL1	0.0844435	0.2463175
TBC1D10A	-0.0643832	0.2464458
MRPL42	0.1208774	0.2468882
KDM3B	0.0475925	0.2470793
YIF1A	-0.0140582	0.2474509
MVB12B	0.0150496	0.2475267
RANGRF	0.1149538	0.2477729
PKD1	0.0439695	0.2483722
CXorf40A	-0.0617191	0.2486106
DNAJC11	0.0724266	0.2489824
STPG1	-0.0570936	0.2510271
RCN2	0.1671093	0.2510404
CATSPER2	0.0651799	0.2516504
TLK2	-0.0872393	0.2520332
ZFAND2B	0.0657865	0.2529647
POLG	-0.0304363	0.2529687
ZNF587B	0.0778069	0.2535189
AC019080.1	0.0556896	0.25402
LRRC57	-0.118807	0.2546705
DNAJC1	-0.1146048	0.2558191
COPS8	-0.1044262	0.2559593
TBL2	-0.0915872	0.2606571
TOLLIP	-0.1202261	0.2607761
BX284668.5	0.0494344	0.2614638
NAXE	0.1215364	0.2614721
KANSL2	0.0315251	0.2667162
TFIP11	-0.1236853	0.266949
GPI	0.1137548	0.2674218
AC107214.1	0.0435023	0.2679033
HINT2	0.0651229	0.2692139
LRRC23	0.0707666	0.2709716
TEX22	0.0475271	0.271507

NRM	-0.087391	0.2720417
NDEL1	-0.1315176	0.2723664
PSMB6	-0.0617453	0.2736161
MACO1	-0.0596552	0.2743431
ERGIC2	0.0609701	0.2753784
ATMIN	0.0093246	0.2755417
ETFRF1	-0.175236	0.2765517
GCDH	0.065262	0.2766847
PPP6R2	0.1001991	0.2772173
CEP83	0.0866314	0.2783413
NCAPD2	0.0815198	0.2788834
EIF4E3	0.0721368	0.2795692
ANKAR	0.0542707	0.2803084
ZNF91	0.1487731	0.2803869
C6orf62	-0.0263598	0.2807503
HEATR1	0.1156765	0.2809888
H2AFX	-0.1221674	0.2826995
PRPF4	-0.0442119	0.2836742
SUZ12	0.1221056	0.2851645
MRM2	0.0590852	0.285791
CREB3L4	0.1092271	0.285796
TMEM208	-0.142152	0.2864504
ZNF43	0.1729868	0.287653
ARL2BP	-0.1179661	0.2878672
NT5DC3	-0.1476279	0.2919741
WT1-AS	0.0698878	0.2946706
MAEA	-0.0620937	0.2959275
GIMAP2	0.0558114	0.2961529
ETFDH	0.0104286	0.2964452
MTMR14	-0.0592592	0.2970384
CTBP1-DT	0.0753628	0.2972833
TIPIN	0.0759982	0.2979051
P2RX1	-0.0874353	0.2990417
TAF3	0.1275452	0.3000867
NRIP1	0.1028461	0.3002269
YIPF5	-0.0994181	0.3008052
AASDHPPT	0.0066068	0.303097
NLRP2	0.0777499	0.3049297
RAB5IF	0.1305538	0.3051753
EPM2A	0.0554962	0.3052325
RPUSD1	0.0821322	0.3098271
AC007383.2	0.0328773	0.3110663
DNASE2	0.0953146	0.3126186
MDH1	0.1446882	0.3129932
NAA40	0.0176313	0.3132526
FAM53C	0.0406758	0.314421
TMEM68	0.0770162	0.3149503
GNL2	0.04827	0.3165112
RBM34	-0.0086665	0.3166642
SOD1	-0.0757759	0.3167794

PARG	0.0678147	0.3168102
TCHP	0.1250404	0.3168499
MSANTD4	0.0984074	0.3184492
TRGC2	0.1265634	0.3187079
LAMTOR3	-0.1529025	0.319273
ZNF354C	0.0544252	0.3212558
BCAS3	0.0513948	0.3214734
FLNA	-0.2269351	0.3214934
RILP	0.0688751	0.3223564
TCTA	-0.1115139	0.3244723
CYB5B	-0.0756463	0.325423
ZNF787	-0.0368354	0.3262071
GMDS-DT	0.0997595	0.3264242
PRMT6	0.02508	0.3268282
NUSAP1	0.0939892	0.3274235
KIF11	0.0826371	0.3274504
GLRX3	0.0804827	0.3277223
AL354740.1	0.0458323	0.3288485
NSD3	0.1139856	0.3293823
MED27	-0.0573744	0.3304101
TBC1D15	0.0409133	0.3317997
RPAP1	0.0479408	0.333815
LHFPL2	-0.0824995	0.3346567
GANC	0.0488435	0.336507
ARHGAP17	0.0141211	0.3391197
CCDC97	-0.1420036	0.3391866
HBS1L	0.0291124	0.3398407
ECD	-0.1036521	0.3398785
ZBTB39	0.0261038	0.3429399
ARMH1	-0.1191851	0.3443168
ACYP1	0.0293564	0.3457975
GBF1	-0.0068568	0.3458112
TSPAN4	0.0490741	0.3465704
STOML2	0.1191913	0.3480008
ZMAT5	0.0759486	0.3494107
MOCS2	0.0733662	0.3513155
SDF2	-0.0757247	0.3526887
EML3	0.047821	0.3552505
PIGU	0.0508123	0.3552832
KATNBL1	0.1970972	0.3555898
EIF4A3	0.0359185	0.3558087
PEX5	0.033369	0.3575051
TK2	0.0392619	0.358954
AGFG1	0.0369898	0.3599533
RNF2	-0.1422328	0.3612349
AGK	0.079036	0.3644037
SERGEF	0.0707775	0.365783
TSPYL1	-0.1300555	0.3660392
SLC27A1	0.0255445	0.3676196
BAG4	-0.1037825	0.3678886

ISCA1	-0.0556808	0.368297
PRKAG2	0.1140109	0.3699545
SHMT2	0.0618627	0.3714347
ZFC3H1	0.1120025	0.3732309
TIMM8A	0.0808522	0.3745706
PFDN2	0.0024008	0.3806542
DBF4	0.0869673	0.3808658
MRPL17	0.1022669	0.3810062
CIC	-0.0904884	0.3815939
PDCD2L	0.0600491	0.3818538
RNF220	0.1070685	0.3829932
AL157834.1	0.055183	0.3832418
MIR646HG	0.0320414	0.3847449
CD36	0.3844339	0.3848449
IDH3A	0.0824518	0.3850024
FGL2	0.0557239	0.3853619
SNRNP40	0.1362152	0.387754
METTL23	0.034629	0.3877695
RPS2	0.1010399	0.387991
SNHG25	0.0646073	0.3906118
WDR5B	0.0838906	0.3915398
ATG13	-0.0925571	0.3918076
GNPTG	0.084376	0.39186
OTUD1	0.025942	0.3931689
HK2	0.0555793	0.3932283
NDUFA13	-0.1782889	0.3932749
CLN8	-0.1007715	0.3954653
TPT1	0.1072211	0.3980593
AVL9	-0.1105495	0.3983509
AC008915.2	0.0437733	0.3984045
ELK1	-0.0333705	0.3987194
NIPAL2	0.1084243	0.400006
PINLYP	0.0366074	0.4015879
PPM1G	0.0756795	0.4025855
TRIM27	0.1243535	0.4040669
LRSAM1	0.0402926	0.4044893
ASXL2	0.0784057	0.4075778
SDHAF1	0.1020763	0.4077292
ZNF529-AS1	0.0579122	0.4078528
ALDH6A1	0.0962039	0.4082646
PPP1R3B	-0.0732832	0.40857
ILKAP	-0.0562333	0.409844
RNF103	-0.1068276	0.410867
ATP11B	-0.1454109	0.4112462
FNDC3A	0.0626935	0.413675
MAP2K2	0.0943415	0.413728
AC159540.2	0.1002507	0.4142335
MIR17HG	0.0422201	0.4177332
CSNK2A1	-0.1067783	0.4187936
CLCC1	0.0640306	0.419685

RBMS1	-0.1662916	0.4225062
AKAP17A	-0.0747945	0.4225182
TMEM164	0.0317524	0.4233415
FAM131A	-0.1000336	0.4242769
MILR1	-0.0967625	0.424865
MHENCR	-0.1186929	0.4288196
GRSF1	0.1456418	0.4293741
MCCC2	0.1131924	0.4296665
SMAD4	-0.0999186	0.4336034
ERVK13-1	0.0303566	0.4340391
TCEA2	-0.1381986	0.4344526
LINC00539	0.0420325	0.434798
RAVER2	0.0810141	0.4370292
ZCWPW1	0.0666563	0.440079
PPP1CC	0.0181395	0.4407759
TMEM115	-0.1041584	0.4408294
LGALS3BP	-0.1221223	0.4420487
VPS28	0.0496846	0.4420583
TMEM109	0.0778295	0.4439112
IRF2BP1	-0.0161836	0.444154
ARID2	-0.0730601	0.4447091
MPND	0.0709295	0.4457418
ALDH9A1	0.0339506	0.4467644
GFM1	0.0451756	0.4475896
CTDSPL2	-0.0764543	0.4500616
ANKRD40	-0.0567864	0.4509133
VENTX	0.0040585	0.4542441
SPICE1	0.0645347	0.4555616
DPP8	-0.0831538	0.4562091
VPS33A	0.0004681	0.4586215
DNAJB4	0.0868199	0.4589294
NPHP3	-0.0129001	0.4592703
SEC13	-0.0486568	0.4601623
SMCR5	-0.1351113	0.4626862
ZCCHC10	-0.0891595	0.4643944
ISOC1	0.041658	0.4685854
IRF5	0.0716113	0.4691835
UBAC2	0.0738667	0.4696899
DLG1	0.089182	0.4704653
TUBA1C	-0.1673348	0.4710748
HMGXB4	-0.0728415	0.4713859
REEP5	0.0522457	0.4745162
TMEM134	0.0491655	0.475092
NECAB3	0.0291743	0.4764544
TMEM267	0.0440189	0.4772802
DDI2	-0.082459	0.4775175
NRAV	-0.0458165	0.4779117
CWF19L2	0.1122732	0.478035
PDE6B	0.0694521	0.4782516
TCEAL9	0.1071041	0.4784414

AC138649.1	0.0298489	0.4786128
TMEM9	0.0558402	0.481766
C8orf76	-0.1040771	0.48187
TMEM30A	0.0341098	0.4824181
NFE2	0.0275672	0.4859466
PTPN12	-0.0775888	0.4864527
TIMM17B	0.0008802	0.4877946
ARID5B	0.0692937	0.488676
NUDT14	0.0729581	0.4918937
AC008105.3	-0.1076989	0.4946344
PEX26	0.0756233	0.4968271
TMEM9B-AS	0.0754006	0.4989619
PHETA1	-0.0613722	0.5016792
RASSF3	-0.0532422	0.5021963
EIF2S1	-0.1180348	0.5035812
NPRL3	0.0728513	0.5051689
MEN1	0.0420408	0.5062575
NCOA1	0.0670772	0.5069952
STN1	0.121445	0.5073642
BORCS7	0.039094	0.5085818
CROT	0.0689842	0.508971
PIGC	0.0986606	0.5095348
PHF2	0.0524787	0.5125666
NDUFS3	0.1588965	0.5142844
BOP1	0.053041	0.5145044
INPP1	-0.096598	0.5153808
UBE2D4	0.0813814	0.5179928
BORA	0.0502299	0.5231276
GSDMD	0.0975383	0.5250537
TADA2A	0.0761529	0.5286398
CTSZ	-0.1026405	0.5292411
SMAD2	-0.1183593	0.5326004
CTNNBL1	0.1135622	0.5328942
PSMB3	-0.1419861	0.5338369
SORT1	0.094254	0.5339647
SQLE	-0.2272622	0.5350202
PIK3C3	0.1308014	0.5351368
RCOR1	-0.0011584	0.535911
WDR92	0.0713799	0.5398753
LINC02175	0.0531237	0.5418327
CDKN2AIP	0.0899422	0.5432285
LINC01355	0.0639269	0.5444653
AC022973.5	0.0695502	0.5454799
UHRF1BP1L	-0.0599709	0.5475182
POLR2E	-0.1199764	0.5483363
ARHGAP10	0.0527069	0.55188
CUL3	-0.1586371	0.5526204
SUPT20H	0.0857183	0.55502
USP42	-0.0411545	0.5553416
MAP3K11	-0.1523117	0.5553654

ASB16-AS1	0.0607494	0.5570647
DAXX	-0.0523814	0.5580961
PLRG1	0.1165893	0.5594405
ZNF561-AS1	0.0749148	0.5595687
PPP1R12B	0.0645958	0.5620746
PLEKHJ1	-0.1103781	0.5628358
AL162458.1	0.0241408	0.563277
NRROS	0.0661259	0.5647521
ZSCAN26	0.0761026	0.5648689
LMF2	-0.1354524	0.5718604
PPP2R3C	0.0532192	0.572842
GTF3A	0.224015	0.5749454
MTMR4	0.0621463	0.5761873
HECA	-0.1608698	0.578996
PCBD1	0.1359585	0.5801332
CPSF6	-0.0837687	0.584006
CASP4	0.1678335	0.5840864
MSTO1	0.0209814	0.5846553
LONP1	0.053314	0.5849297
PICALM	-0.1455438	0.5849365
RFXANK	0.1217034	0.5854448
ZNF616	0.0530878	0.5859898
ATF7	-0.115315	0.5862688
CKMT2-AS1	0.068706	0.5874747
AC036176.1	0.0514061	0.5883884
NOL6	0.0381215	0.5899509
ZNF24	-0.0450044	0.5904471
TIAL1	0.1930352	0.5914831
KIF23	0.0404968	0.5917082
MOCS3	0.0081439	0.5936376
SPEN	-0.1093925	0.594882
LINC01410	0.0712535	0.595691
BCAS2	-0.1308745	0.5968269
ALG9	0.0238931	0.5976647
PSMB4	-0.0769215	0.5981998
RAB9A	-0.0869145	0.6005632
CENPQ	0.0621655	0.6021371
HSPA1B	-0.0250207	0.6021987
LRRC59	-0.1147777	0.6027227
C5orf56	-0.0950445	0.6027619
RPP25	0.0509964	0.604224
ETV5	-0.0807121	0.6057062
CHAMP1	-0.1066103	0.6061342
DAGLB	-0.0868532	0.6072419
IPO5	0.1292505	0.6078023
CPOX	0.0611547	0.6079559
CDC16	0.0565901	0.6081714
ACBD4	0.0516232	0.6085989
ST3GAL1	-0.1820389	0.6119501
IFT20	-0.0798413	0.6143574

GPR135	0.0642256	0.6186756
SERAC1	-0.0453963	0.6191536
KLHL9	-0.0733923	0.6197326
RAD18	0.0539902	0.6219088
WIPF1	0.0841019	0.6219201
ZBTB37	0.1417547	0.621964
FPGT	0.0535848	0.6238207
HAUS3	0.0488148	0.6271445
ATP11A	0.059862	0.6289983
RELA-DT	0.0443473	0.6352122
TMED8	0.0949733	0.6368977
FRMD8	-0.0354406	0.6376012
ARMC5	0.061446	0.6394981
ACAP3	-0.1150118	0.6420117
GATB	0.070138	0.645021
C17orf58	0.0346225	0.6558056
ZDHHC5	-0.06338	0.6558109
DPAGT1	0.0711339	0.6558849
MBNL3	0.0729133	0.657309
ATRAID	0.0735873	0.660244
NAA50	-0.1134721	0.6609807
RRNAD1	0.0516395	0.6613518
CFAP44	0.0768561	0.6615375
ZNF410	-0.157942	0.6629604
TRIM59	0.155838	0.6654552
ZNF800	-0.1217925	0.6656337
NUDT22	0.0668671	0.667408
APPL2	0.0421899	0.6675918
NPEPL1	0.0135969	0.6691903
STIMATE	-0.0590763	0.6709296
FAM43A	-0.0703164	0.6719459
P4HTM	0.0087771	0.6736303
SARS	0.132802	0.6748717
UBAP2L	0.0678652	0.6752791
METTL1	0.0687472	0.6794673
ING4	0.093336	0.6867603
TBCB	-0.1859074	0.6875147
DTD1	0.0884727	0.6957539
CCDC171	0.0572467	0.6963054
GMEB1	-0.1252881	0.6971501
NUB1	0.0483167	0.698673
LTBP4	0.0726007	0.6988403
SLC35G2	-0.0571474	0.6995026
SLA2	-0.0174044	0.7005021
SLAIN2	-0.0368236	0.7014708
ZNF227	0.0393952	0.7039299
TRMT61B	0.0847329	0.7054666
FAM126A	-0.005402	0.7074353
TFPT	0.1047333	0.7102516
MRPL27	-0.0217389	0.7119142

VPS9D1	-0.0588064	0.7156996
RNF217	0.0651923	0.7178254
ZNF829	0.0435131	0.719732
TMEM18	0.1271996	0.7225513
TFCP2	0.0809589	0.7229289
NEU3	0.0363655	0.7266588
PRPSAP2	0.0566888	0.728326
USP11	0.0601262	0.7306753
SOCS4	0.1527463	0.7352372
KPNA3	-0.13447	0.7361517
RWDD1	0.0801488	0.7378251
DDHD2	-0.0028054	0.7380386
MPZL1	0.0810768	0.7383147
NNT	0.1153453	0.7393329
XPA	0.1147565	0.7397125
VHL	-0.0365073	0.742041
BCAT1	0.073304	0.7444084
ABHD10	0.0699661	0.7473185
STK32C	0.0827446	0.7474919
TMED1	-0.031388	0.7480422
ERI3	0.0798533	0.7547001
YEATS4	0.0601274	0.7561469
CHD9	0.0614101	0.7590159
FXN	0.0947669	0.7590347
MLST8	0.075728	0.7610689
RNF5	0.0831121	0.7611612
CARM1	0.0467164	0.7621541
MAP3K14	-0.0594776	0.7622589
Z93241.1	0.0245678	0.7712732
COQ6	0.0105128	0.7716673
ZNF213-AS1	0.0626773	0.7744928
SELENON	0.0545105	0.7744966
IKZF5	-0.1025709	0.7760502
BLCAP	-0.1048525	0.7808095
C1orf216	-0.0563848	0.7868021
SEC22B	0.1227805	0.7879356
CTDP1	-0.0853458	0.7933085
TAF11	-0.0700513	0.7952706
USP36	-0.0478581	0.7956367
CTSO	0.0431821	0.7991921
PIK3AP1	0.1157982	0.8019443
IK	0.0061405	0.8027964
CCDC26	0.0744439	0.8061808
HBEGF	0.0735547	0.8079278
LY6G5C	0.0494508	0.8130672
GOLGA2	0.0226683	0.8134501
GSK3B	0.1028649	0.814667
STXBP2	-0.1119176	0.8166061
PEX1	0.0643428	0.8188837
TRMT2A	0.0663565	0.8191448

GLYCTK	-0.0706486	0.8224657
RASGRP4	-0.0636727	0.8246069
DHX30	-0.0186202	0.8251697
ILF3	0.0691633	0.8258446
POLR3G	0.0757521	0.8305907
FO393401.1	0.0682971	0.8323961
HNRNPD	0.161325	0.8326237
KBTBD3	0.0619306	0.838244
ORAI3	0.0031736	0.8383802
AC005261.1	0.0713265	0.8402296
STAM	0.0247114	0.8411562
MEF2A	-0.1503369	0.8434716
PCED1A	-0.0306538	0.8444013
KIAA1958	0.0741965	0.8462421
SEC31B	0.0274649	0.8463965
MCM8	0.0602541	0.8546137
PGPEP1	-0.0659179	0.8564241
TMCO3	-0.1020941	0.8624401
AP3S2	-0.0731488	0.8645886
KAT8	0.0537022	0.8675685
ZUP1	-0.0995332	0.8744109
TMEM69	0.0772464	0.8750156
U2AF2	0.0693327	0.8758075
TYW5	-0.0283691	0.8766339
AGPAT3	0.0482151	0.8771243
GBA2	-0.0985512	0.8833381
USP10	0.0378639	0.8850621
RPGRIP1L	0.0498628	0.8879897
PRKAB2	-0.0372937	0.8939461
DIRAS3	0.0464328	0.8960032
EIF2AK4	0.1644119	0.8984994
CASP10	-0.1104109	0.9017461
UBE2K	-0.0737842	0.904476
DGUOK	-0.0378551	0.9063582
ANKRD54	-0.0057	0.907292
C20orf194	-0.0991735	0.9096249
ATAD1	-0.0362681	0.9142175
GNAI1	0.1404229	0.9148005
BABAM1	-0.0090054	0.9157222
UBALD1	-0.0856899	0.9180402
TTLL10	-0.0680709	0.9197084
CPT2	0.035755	0.924803
ZNF775	-0.1031816	0.9255627
DBNL	-0.138748	0.9292052
ACSL4	0.1332725	0.9323345
DAZAP1	-0.1505003	0.9356874
LDLRAD4	-0.2015231	0.9360283
ZBTB1	0.0968009	0.939417
PRICKLE1	0.0247112	0.9460863
TRPT1	0.0695619	0.9476106

TUBGCP3	0.0792495	0.9528169
VPS11	0.0593842	0.9544686
C11orf98	0.0504949	0.9564243
POLRMT	0.0227749	0.9575601
TVP23B	0.035565	0.9590395
ZNF169	0.0591649	0.9590829
DHRS4L2	0.0763213	0.9607848
TBXA2R	-0.073872	0.9619455
SCLT1	0.0698542	0.9643488
HINFP	-0.0426839	0.9651401
CCT6B	0.0412673	0.9666618
ATXN3	0.0273287	0.9675424
ACYP2	0.0639876	0.969201
CDK5RAP2	-0.0582739	0.9779125
ZBED1	0.0620768	0.9819054
COX10	0.0225892	0.9837633
RHOBTB2	0.0220638	0.9868686
ZNF639	0.0816699	0.9875508
PLGLB2	0.070606	0.9880353
PUF60	-0.1481899	0.9888272
PSMA3	-0.1703463	0.9892656
LEPROT	0.0998383	0.9906566
GK5	0.0728285	0.9930354
AC020915.2	0.0418212	0.9939143
ZNF580	0.0500119	0.9968111
RPS6KC1	0.0519551	0.9969468
TSR2	-0.0862195	0.9969931
DHFR	0.0631918	1
YKT6	-0.0759744	1
TET2	-0.1520795	1
SOCS5	0.0390157	1
LRRC8D	-0.0110663	1
YIPF3	-0.1360898	1
SNRPD3	0.0902117	1
DNMBP	-0.1039648	1
JPT1	-0.1367921	1
BCL10	-0.0962011	1
TOPBP1	0.1498736	1
MAP4	-0.1200348	1
PLD1	0.0415009	1
RALGPS2	0.0373568	1
SPRYD3	-0.0363426	1
ELP6	0.0922382	1
PLOD1	0.0481412	1
PLAA	-0.0954866	1
APOOL	0.1066179	1
EMG1	-0.0408548	1
CHM	0.1101165	1
STAC3	0.0784774	1
ZDHHC12	0.0160271	1

NME6	0.0393429	1
SCRIB	0.0169143	1
ZNF318	0.0441174	1
VDAC2	0.1205011	1
DHX57	0.0463734	1
FN3KRP	0.0411909	1
LSM3	0.0184218	1
AC004865.2	0.0544262	1
ZNF660	0.0891968	1
TPSB2	0.0419243	1
POLR2J	0.1068684	1
SIK2	-0.0150373	1
BID	-0.1556592	1
LTBP3	-0.1480559	1
LINC00174	0.0623359	1
AC147651.4	-0.089534	1
ADAM10	0.1288215	1
RLF	-0.047791	1
NIT1	0.0514829	1
AC099518.5	-0.00639	1
UTRN	0.0755714	1
LINC00863	0.0138607	1
CCDC18-AS	0.1699334	1
GSTZ1	0.0545897	1
LARP1B	0.0723344	1
NDUFA7	0.1105343	1
PHIP	-0.0745117	1
SEPHS2	0.0289723	1
MAU2	-0.0048852	1
NFKBIB	-0.1433288	1
FLYWCH1	0.0467339	1
MIR762HG	0.0345485	1
ANXA7	-0.1529975	1
BANK1	0.0499295	1
TRIOBP	-0.1028546	1
AL391832.2	0.015274	1
P2RX4	0.0188135	1
ZNF525	0.0657829	1
EDC3	-0.0544818	1
ZHX1	0.0811815	1
CCDC102A	-0.012261	1
AC009318.1	0.1930561	1
PPP2CB	-0.1122752	1
PCYT2	0.0547271	1
LRRC75A	0.0999431	1
SPTY2D1OS	0.0315922	1
CEBPA	-0.0459294	1
GRAMD1A	-0.0684679	1
AC127502.2	-0.0673516	1
MVB12A	0.0846987	1

NOP16	0.1660266	1
COPS4	-0.1394682	1
ELMOD2	0.0647971	1
AC006480.2	0.0318109	1
SQOR	-0.0290324	1
SPNS1	-0.0461079	1
FAM120AOS	-0.1036975	1
ZNF433-AS1	0.1097475	1
EIF3H	-0.0951708	1
MBD5	0.0262859	1
MOGS	0.0337215	1
MFSD8	-0.0749168	1
RNF167	-0.0373987	1
SNHG7	-0.1268923	1
SPATA33	-0.0808655	1
AMACR	0.039137	1
MIIP	0.0522206	1
FARS2	0.0267003	1
POLR3A	0.0961899	1
SLC39A9	0.0336911	1
COPB2	0.0698582	1
FBXW4	-0.0234286	1
CHD8	-0.1391501	1
VTI1A	-0.100039	1
MAP3K12	0.0435883	1
SNHG21	-0.0868036	1
EVA1B	-0.2455049	1
FDFT1	-0.126552	1
STK3	0.0909594	1
CNDP2	-0.050803	1
SSH1	-0.1232091	1
NAGLU	0.0036761	1
CCNQ	-0.0737623	1
MCM3AP	0.0961601	1
MRM3	0.0144833	1
NUP85	0.0300585	1
FBRSL1	0.0664613	1
LRCH4	-0.0626238	1
CAMK2G	0.0893925	1
EPDR1	0.0943057	1
QRSL1	0.0679193	1
ZNF329	-0.0331439	1
SHLD2	-0.0411292	1
TUBGCP2	-0.0754825	1
N4BP2L2-IT2	0.0456402	1
PCSK7	-0.0964834	1
ZNF544	0.098853	1
JADE2	-0.0958324	1
FAM104A	-0.0388585	1
SMIM15	0.0581229	1

TEFM	0.0622398	1
CNP	0.0573074	1
SESN2	-0.0756767	1
ZBTB16	0.2086368	1
KLHDC10	-0.0999782	1
CCDC62	0.0581988	1
FNBP1	-0.1176242	1
MCM2	0.0478226	1
TSHZ1	0.0434088	1
ATF6B	-0.0955874	1
TCTEX1D2	0.0130895	1
ZDHHC2	0.0739619	1
DAP	-0.0824152	1
ZC3H10	0.0218034	1
DALRD3	-0.1325359	1
NDUFA12	-0.0535639	1
MYO15B	-0.107741	1
ANKRD13A	0.0456661	1
INPP5D	0.0794265	1
CCNJ	0.0286052	1
NOB1	0.1367494	1
ATP5PB	-0.1617007	1
FAM135A	0.0685219	1
MRPL18	0.0688683	1
AL645728.1	0.0486497	1
ENTPD3-AS	0.0463474	1
AC008443.1	-0.0053577	1
POLR2I	0.1866575	1
ATP2C1	-0.0929945	1
GCLC	0.0843618	1
PKM	-0.1144899	1
SAE1	0.0179385	1
RPS6KA3	-0.1293362	1
PSMB9	0.1142127	1
LRIG2	0.0094249	1
ROBO3	-0.0873139	1
VPS54	0.0349345	1
EXOC7	0.0111482	1
TAZ	-0.092358	1
WDSUB1	0.0633709	1
ABHD17B	0.0853915	1
OLIG1	0.0269911	1
POGZ	-0.1435061	1
YTHDF3	-0.0420544	1
PGM1	0.0958259	1
WWP1	0.0458528	1
RAD21	-0.119827	1
DDX19A	0.0246216	1
AC025171.1	0.1079249	1
MED13	-0.1204898	1

RAB27A	0.0859559	1
SAMD8	-0.1201165	1
C5orf15	-0.0639574	1
RAB8A	0.1261775	1
WBP1	-0.1156227	1
BIRC2	-0.1255371	1
ERC1	0.0413154	1
PRIMPOL	0.0900619	1
TMEM126A	0.0953738	1
COMMD8	0.1050244	1
GSAP	0.0715322	1
RFX7	0.0736625	1
RAD54L2	0.0405473	1
PIK3R2	0.045447	1
TIMM23	-0.1190356	1
CENPH	0.0604245	1
SF3A3	0.1017318	1
SLC30A7	0.0369511	1
ZNF430	-0.1201396	1
ZNF566	0.0837632	1
NPIPB4	0.0417996	1
FBXW8	0.0549519	1
CC2D1B	-0.0852924	1
ANKRD39	0.0524913	1
KSR1	-0.1212051	1
KIAA0930	0.0556511	1
PLTP	-0.0725241	1
TSPAN31	0.0039112	1
RNF6	-0.0021348	1
ZNF362	0.0372933	1
SUCO	-0.0455364	1
TOR1A	-0.0506031	1
OSBPL11	-0.0843535	1
MTPN	-0.1681363	1
MRPL58	0.0438744	1
SF3B6	-0.0857915	1
PEX13	-0.1173011	1
SMDT1	0.0034773	1
DNASE1L1	-0.0874583	1
UTP6	0.1161619	1
COA8	0.0512284	1
LRRC42	-0.1013674	1
TAX1BP3	0.0660184	1
RMND5A	-0.0268652	1
TMPRSS9	0.0510841	1
MED24	0.0191642	1
USP47	-0.1772748	1
FANCI	0.0555061	1
HGH1	0.0457816	1
COPS5	0.1049896	1

ABHD14B	0.0696389	1
DNAJC2	0.0399041	1
PDIA4	0.0672809	1
SUN2	-0.1105091	1
TRMT11	0.0351142	1
HSPA14.1	0.0265659	1
EPS15	0.0672726	1
ALG1	0.0530278	1
AC142472.1	0.1004256	1
NAT14	-0.0640876	1
PDE4D	-0.0492746	1
TENT4B	-0.0026385	1
PGRMC1	-0.1093278	1
BDNF-AS	0.0719765	1
PARP14	0.1394536	1
BTBD2	0.0661948	1
NEMF	0.1133883	1
ZNF568	0.0704743	1
ZDHHC20	-0.0183684	1
FBXO6	0.0504149	1
RGP1	0.00684	1
SRRT	0.0869098	1
PLBD2	0.0327094	1
CHFR	-0.0936832	1
PNO1	0.0446523	1
MFAP1	-0.0571269	1
TXNDC16	0.044807	1
UQCRB	0.1044787	1
RPL28	-0.1043688	1
AL133243.2	-0.0808658	1
PHB	0.1191835	1
CHML	0.1289691	1
STK39	0.0312666	1
ZNF780A	0.0727728	1
AMBRA1	-0.077015	1
SLC16A1-AS	0.0524599	1
ACAA1	-0.0637178	1
STK10	-0.1252227	1
TXNDC17	-0.0421236	1
SAFB	0.1056432	1
NPIPB5	-0.0004054	1
D2HGDH	-0.056364	1
RCE1	-0.0954147	1
SPTLC2	-0.1515625	1
PPFIA1	0.0532253	1
PITHD1	-0.10622	1
AC139099.2	-0.0293767	1
GIMAP6	-0.1170396	1
PDK1	0.0687515	1
RSPRY1	-0.1106506	1

SUCLA2	0.0504674	1
CTCF	-0.0199615	1
NDUFB7	0.1326092	1
COQ10A	0.0612131	1
ANKRD18B	0.0400328	1
C12orf57	0.1505717	1
EPC2	0.0123993	1
C1GALT1	0.0338806	1
MFN1	0.0480422	1
TUT4	0.0670336	1
JRK	0.0801286	1
PIWIL4	0.0431953	1
PXMP4	0.0622469	1
GATAD2A	-0.0898891	1
HIST2H2AC	0.0305176	1
NDUFA9	-0.0612815	1
GIMAP1	-0.0630915	1
ZFP82	0.0474264	1
AL162253.2	0.0317524	1
ATAD3A	0.0585109	1
ZNF394	-0.0783019	1
FLAD1	0.0288455	1
PPBP	0.338302	1
ARHGAP1	0.0047267	1
RSKR	0.0549028	1
CENPK	0.0572382	1
ACD	-0.0326476	1
PLEKHB2	-0.0938594	1
AAMP	0.0404944	1
CCDC51	0.0459723	1
MX1	0.1146463	1
TBC1D10B	-0.150371	1
PSMC2	-0.0914187	1
AC046134.2	0.0296979	1
TXNDC11	-0.0786456	1
SCAPER	0.0583799	1
NLRP3	0.0007834	1
DHX29	-0.0269257	1
LRRC34	0.0629271	1
CARS2	0.098824	1
UBR1	-0.0459082	1
RXRA	-0.1241566	1
ELP2	0.1018165	1
PTPN9	-0.0947014	1
IGF2BP3	-0.0539029	1
CHTOP	-0.0493215	1
GPRC5C	-0.0396929	1
SMG6	-0.0297083	1
ANGPTL6	0.0687726	1
CCDC30	0.0695647	1

HSCB	0.1138343	1
FAM221A	0.0538091	1
HERPUD2	-0.1018616	1
PRICKLE3	0.0219275	1
WWC3	0.0387016	1
LARP4B	0.0125303	1
NAA60	-0.1005123	1
OSBPL3	0.0337038	1
NIPSNAP3A	-0.0654928	1
PAXX	-0.0296698	1
RPUSD4	0.0502977	1
SNHG29	-0.1471872	1
PYCR1	0.0731741	1
ZNF700	0.0057964	1
GOLGA8B	0.0341663	1
RETREG3	-0.0701268	1
RAB7A	-0.1283734	1
ZNF441	0.0173438	1
RHOT2	-0.0306798	1
H2AFY	0.1461801	1
NMB	0.0866649	1
COMT	0.106612	1
POLE4	0.1141544	1
ZNHIT2	-0.0630252	1
KDM4B	0.0137229	1
PRPF19	0.0641632	1
ATF7IP2	-0.0794256	1
TRMT10A	0.0308884	1
N4BP2L1	-0.0746502	1
ELOVL1	-0.0704863	1
ABT1	-0.1185968	1
EIF2B4	0.0294571	1
AC135050.6	-0.0262974	1
PMPCA	-0.037224	1
NETO2	0.052437	1
AL096870.8	0.0585611	1
TSPOAP1	0.0517658	1
KLHL42	0.0621086	1
TNPO3	-0.0450934	1
DYNLRB1	0.1269658	1
ZNF23	0.0531916	1
UTP4	0.0543493	1
ARL6IP6	0.03598	1
MAPK3	-0.0896903	1
MOB4	-0.0892883	1
C7orf26	0.0452708	1
IMMP2L	0.1012228	1
CENPF	0.1347802	1
TMEM38B	0.1288987	1
XPC	0.1048655	1

UBE2W	0.0329272	1
RHOQ	-0.0845362	1
NKIRAS1	0.0686398	1
TTYH3	-0.0901106	1
SLC41A3	-0.0319272	1
ZDHHC9	0.0748371	1
SMIM7	0.0492117	1
ZNF496	0.0384984	1
ABCD4	-0.0680145	1
TRA2A	-0.1494278	1
SLC38A10	0.1110944	1
RABEP2	-0.1357196	1
RPP14	0.0434169	1
AVEN	0.0368628	1
PSMG3	-0.0087668	1
FAM91A1	-0.023633	1
HOXB4	0.0432697	1
DDHD1	-0.0979892	1
ARL17A	0.0584331	1
ZNF197	0.0591406	1
THAP12	-0.097768	1
MTERF4	0.1390965	1
CBY1	-0.0434782	1
OBI1	0.0687333	1
MED19	-0.1108014	1
SFT2D3	0.0392764	1
NDUFA5	0.0390928	1
PPFIBP1	0.0514759	1
FKBP7	0.0566922	1
GTF2E2	0.0515901	1
GGA1	-0.1487803	1
GSTP1	0.1248849	1
FAM106A	0.0454718	1
XIAP	-0.1062392	1
ZNF559	0.0903637	1
TXLNG	0.092508	1
OGFR	-0.0534163	1
RNF125	-0.1615796	1
TBL3	0.0580032	1
DLGAP1-AS	0.0960442	1
ZNF507	0.0836404	1
LINC00672	-0.0824872	1
EML4	-0.1122327	1
DUSP11	-0.1296592	1
AC009812.1	0.0327633	1
SETD3	0.0157925	1
SMC5	-0.0525739	1
FAM98B	0.1034421	1
CCDC82	-0.1711346	1
FAF1	0.0835538	1

KMT2E-AS1	0.0767557	1
ECH1	0.007628	1
PDCD4-AS1	0.0727077	1
STK16	0.0086059	1
NAGPA	0.086202	1
MCM9	0.0384664	1
TMX2	0.080597	1
CLMN	-0.0599195	1
LTN1	0.1195687	1
HEXD	0.0577698	1
TRMT44	0.0434423	1
C2CD2L	-0.0671742	1
NKIRAS2	-0.1158974	1
NUDT5	0.0196696	1
CEPT1	0.0582303	1
ROMO1	-0.0760554	1
MKRN2	-0.1028108	1
CHRNB1	-0.0278234	1
OXLD1	0.1061388	1
CRISPLD1	0.0774966	1
TATDN3	-0.0235144	1
PLIN2	0.0375747	1
CLIC4	-0.129778	1
FAM13A-AS	0.003895	1
AC005726.1	0.0434629	1
SESTD1	-0.1094141	1
AC092747.4	0.1477413	1
SENCR	0.0534192	1
KIDINS220	0.0888678	1
SENP7	0.0961769	1
PPRC1	-0.0277941	1
SMG7	-0.0908417	1
SRF	-0.0721384	1
CASC4	0.114855	1
AIDA	-0.1340781	1
RIMKLB	-0.1005208	1
ELP3	0.0591452	1
G3BP1	0.1788066	1
MAST3	0.0376943	1
TUSC2	-0.0922228	1
VARS	0.0360127	1
ILDR2	0.0339819	1
LSM14B	0.030296	1
AC012360.3	0.0537714	1
LPAR2	-0.0741736	1
RPL7L1	0.1634122	1
ZBTB42	-0.0430063	1
CIZ1	0.025599	1
RAN	0.1439872	1
DYRK4	0.0466103	1

TRAM1	-0.0788525	1
AC015802.6	0.029609	1
ERO1A	-0.0606436	1
PANK3	-0.101837	1
UBE4B	0.030148	1
RAB12	0.0436317	1
ASH1L	0.0056963	1
FBXO45	0.0422379	1
MCM4	0.1055788	1
FAM234A	0.0520048	1
AP002748.4	0.0411152	1
APTR	-0.0552476	1
MICOS10	0.1731417	1
TBC1D25	-0.0619622	1
GLS	0.0947354	1
GOLGA5	-0.0046905	1
NADK	-0.0354522	1
SNX6	0.0607399	1
GTF2H1	0.0281822	1
ESYT1	-0.1014315	1
SENP5	-0.1043693	1
SAFB2	0.0444278	1
DEF6	-0.0496157	1
EHD4	-0.0889027	1
UBA6-AS1	0.0398428	1
RHBDD1	0.0394726	1
DOCK7	0.0364137	1
EXOC8	0.0236216	1
L2HGDH	0.0641196	1
PDE7A	0.1355851	1
CD151	-0.1646012	1
CYSLTR2	-0.1354422	1
RAB5B	-0.0277957	1
MBD6	-0.0236007	1
RBMS2	-0.0722551	1
SNX3	0.0501218	1
TSPAN33	0.074254	1
SFXN2	0.0551437	1
EIF4EBP2	0.0658336	1
ZNF606	0.0437681	1
ZNRD2	-0.0486695	1
FAM3C	-0.0645526	1
ERLEC1	0.0757619	1
DENND1C	0.0168525	1
WASF1	0.0618383	1
EIF4E2	-0.0920516	1
OXSR1	-0.0368314	1
ORC4	0.0378941	1
DOCK10	0.0435073	1
NHLRC3	0.0293132	1

PATZ1	0.053881	1
DENND6A	-0.0834076	1
DNAJA3	0.0707841	1
MED1	0.024639	1
VPS13D	0.0194595	1
ATXN10	0.068489	1
TMED5	-0.1005426	1
POMT1	0.0145758	1
SDHD	0.120263	1
NUDT16	0.0439106	1
BCS1L	0.0313555	1
ZNF557	0.0718883	1
UBA1	0.072901	1
ZNF426	-0.0051349	1
XRCC2	0.0759824	1
NMT1	-0.0953831	1
TMC8	0.06131	1
PLSCR1	-0.0239965	1
TRRAP	0.0695247	1
SUPV3L1	-0.0818808	1
DPEP2	0.0383114	1
SAT2	0.0913904	1
RBM45	0.041571	1
AC004889.1	-0.0443155	1
TMEM154	0.0446195	1
SYNC	0.0580746	1
ZNF782	0.0370316	1
SIRT7	-0.0986729	1
TUBGCP5	0.0334353	1
METTL9	-0.0704118	1
NRSN2-AS1	0.0769855	1
MTHFD1L	0.0661794	1
ZSWIM9	0.0591921	1
SPECC1L	0.0290119	1
CNEP1R1	-0.0765181	1
MCTP1	-0.036053	1
CHMP1B	-0.1141125	1
RUFY2	-0.1272986	1
CLCN3	-0.0021373	1
AC011447.3	0.0645137	1
PITPNA-AS1	0.07094	1
PLA2G15	-0.0806382	1
CD47	-0.1692822	1
DISC1	0.0827226	1
SNRNP48	-0.0246906	1
DHRS11	0.0727415	1
TOR2A	-0.0479917	1
AAGAB	-0.1023246	1
H1F0	-0.0002795	1
TTLL12	0.048411	1

THG1L	0.0501129	1
OSER1-DT	0.0511097	1
OGFOD1	0.016482	1
ZNF354B	0.0588506	1
TOP3A	0.0231373	1
HNRNPF	-0.1533785	1
ARL3	-0.1039193	1
MAP4K5	0.0416288	1
PNKP	-0.0761594	1
LSM14A	-0.0087561	1
NF2	0.0531048	1
RCBTB2	0.0500288	1
AC093323.1	0.0469732	1
TMOD2	0.0550182	1
DCAF1	-0.0144491	1
PPIH	0.0763885	1
DNM2	-0.0462982	1
HCK	-0.0186029	1
ZNF260	0.0556221	1
PRSS57	0.1233253	1
CCSER1	0.0501677	1
HCST	-0.1378203	1
UBE2S	-0.125358	1
TNFRSF14	0.0843739	1
CHCHD1	0.0602934	1
SAYSD1	0.0444656	1
SDCCAG8	0.2067111	1
REXO1	-0.0871789	1
TRAPPC1	0.0700558	1
ZNF101	0.0550656	1
CHCHD6	0.0482815	1
LINC02482	0.0493941	1
ALKBH4	0.036709	1
ZNF550	0.0511444	1
GUCD1	-0.0604871	1
EXOSC9	0.0231424	1
AK6	0.0492306	1
HTT	-0.1193862	1
CD99L2	0.0396519	1
FAM50A	0.0941973	1
PEX16	-0.076828	1
CTU2	0.0447071	1
NAA10	-0.0490666	1
ZNF738	-0.0197171	1
SLC7A1	-0.0800281	1
PPP1R12A	-0.1298056	1
ODF2L	-0.095055	1
PNN	0.185756	1
GOT2	0.097833	1
COPZ1	-0.0736738	1

ATP6V0E2	-0.0049425	1
DIP2A	0.0980799	1
DHX8	-0.0433251	1
ZHX3	0.0444851	1
RPS6	0.058109	1
BAZ1B	0.0317378	1
MN1	-0.1133786	1
TBC1D14	0.0178667	1
ITM2A	-0.1263965	1
RFFL	-0.1064623	1
APPBP2	-0.0541341	1
ZNF28	0.013775	1
PGAM5	0.0399302	1
NUP155	0.0563004	1
BOD1	0.0505852	1
EMC6	-0.0460651	1
WBP4	-0.0750416	1
LRBA	-0.0380125	1
ERCC6	0.0553698	1
SNHG10	0.0712456	1
TMEM173	-0.1100169	1
PPT1	0.09588	1
TMEM104	-0.0793686	1
LMAN2	-0.100679	1
PIGT	-0.0866519	1
GTF3C3	0.0438588	1
TPRKB	0.0965725	1
ZNF146	0.1600611	1
SWSAP1	0.0636394	1
CEP164	0.0808429	1
PRPF38A	-0.117478	1
SND1	0.07081	1
CERCAM	0.0339752	1
CIPC	0.0116477	1
PIK3IP1	-0.0121035	1
INTS11	0.0148769	1
TMEM106A	0.0029884	1
NDUFS4	0.0835264	1
EXOC5	-0.0910341	1
RPS19BP1	-0.1005509	1
SNHG9	0.116009	1
PHLDB3	0.0260699	1
ZP3	0.0658329	1
USP34	0.057746	1
RPS6KB1	-0.074192	1
EP300	-0.0933101	1
HNRNPL	-0.1162421	1
ZBTB26	0.0350786	1
NFS1	0.0435189	1
C8orf58	-0.0334009	1

ZC3HAV1	-0.145281	1
RFC5	0.0436267	1
ZC3H18	0.0784858	1
WSB1	-0.1716482	1
AC108519.1	0.0875409	1
IFT140	0.0331888	1
SRFBP1	-0.0195363	1
RCC2	0.0238861	1
FNIP1	-0.1232907	1
CDCP1	-0.0472608	1
RPS14	0.0606001	1
XKR8	-0.0760629	1
SHISA5	-0.0764566	1
AP001059.2	-0.0555318	1
IPO9	0.0441615	1
MTMR10	-0.1101201	1
TMEM87B	0.0118332	1
ARSB	0.0347132	1
RDH14	-0.0349866	1
SNHG26	0.0590561	1
ATXN7L3B	0.1550392	1
AC004951.1	0.0581349	1
ATAD2	0.0809911	1
HDAC10	-0.0384645	1
SNF8	-0.0007419	1
INPP5B	0.0273055	1
VIRMA	0.0456807	1
MRGBP	0.0301416	1
RPS6KA1	-0.0343481	1
DLAT	0.0237655	1
AMDHD2	0.0036622	1
NKX2-3	0.0777533	1
DHX9	0.1647334	1
ZNF334	0.0403856	1
CARD8-AS1	-0.0172065	1
CCDC115	0.0757831	1
TAP1	0.0391808	1
TRIM24	-0.0666346	1
ZNF512B	-0.0239892	1
C4orf3	0.1157546	1
CCNDBP1	0.0609562	1
AIMP2	0.0882472	1
BBX	0.1286316	1
OLA1	0.1446337	1
ZCRB1	0.1257988	1
CARD16	0.0019067	1
IRF2	0.0569462	1
DCAF4	0.048712	1
HPCAL1	-0.0665155	1
QTRT1	-0.0864684	1

SNX17	0.0073492	1
SPATA2	-0.0361398	1
KRR1	-0.0619264	1
BRF1	-0.1281526	1
ATP9B	0.0492198	1
PSMC3IP	-0.061549	1
SNX19	0.0178859	1
SIRT1	-0.0699422	1
OTUD6B	0.0751243	1
PRTN3	0.1529981	1
GPANK1	-0.0024274	1
SKP1	0.1610529	1
RAI1	-0.1406635	1
IBTK	0.1058965	1
HOXB6	-0.1554027	1
AC118549.1	0.0890763	1
CENPW	-0.000963	1
RPL7	0.0901712	1
CENPC	-0.0990485	1
XPO7	-0.0054761	1
LYPLA1	0.0871039	1
CCS	-0.0356306	1
TYK2	-0.0500231	1
SLC30A6	0.0612856	1
VPS4A	-0.0356784	1
FANCA	0.0550492	1
CMTM7	0.0471347	1
UCK2	-0.0167797	1
TEX261	0.0058471	1
ALDOA	0.0646699	1
CCDC126	0.0338647	1
HOOK3	-0.0586243	1
SSNA1	0.1422444	1
PHKG2	0.0177892	1
FAIM	0.0629584	1
MAN2B2	0.0539866	1
MTFR1L	0.0772537	1
ANKRD36C	0.0315433	1
CASP8AP2	0.1334193	1
PLD4	0.0554851	1
WDR75	0.0663191	1
EIF4ENIF1	-0.0368242	1
ZNF398	-0.0211434	1
TM2D2	-0.0030193	1
OTUD4	-0.0880654	1
PRPF40A	-0.1548631	1
ROGDI	0.024565	1
HIRIP3	0.0393096	1
DENND4B	-0.0561641	1
CSTF2	0.0130147	1

DPH2	0.0587741	1
CHMP2A	-0.088059	1
ADNP	0.042479	1
PYM1	-0.1533071	1
SRR	0.0327329	1
ZFAND3	-0.0422535	1
STRIP1	-0.0465435	1
JAK2	0.0498473	1
TMEM44-AS	0.0742053	1
FDXR	0.0378991	1
XPNPEP3	0.0624365	1
SLC25A12	-0.0163429	1
ZC3H7B	0.0732605	1
APOBEC3C	0.0970758	1
TMEM156	0.1167676	1
SFI1	-0.0644002	1
URGCP	0.0425661	1
DYNLT3	-0.0149692	1
GATAD2B	-0.0842513	1
RRM2B	-0.101699	1
DIS3	-0.0498428	1
AARSD1	0.0635614	1
LPCAT2	0.0592306	1
FAM160A2	-0.0650409	1
SMCO4	0.0280055	1
C6orf47	-0.0880906	1
TMEM170B	0.0287001	1
CD27-AS1	-0.0500779	1
NOTCH2NL/	0.0014045	1
CALU	0.1005196	1
PRKX	0.038875	1
CASP3	-0.1554346	1
SLC35E3	0.0612448	1
ABCC5	0.0438274	1
PIGV	0.0308099	1
IWS1	-0.0568974	1
PSMG2	-0.1283262	1
HMGCL	0.0272487	1
ARMCX6	-0.0928281	1
TESMIN	0.0525716	1
CD96	0.1632287	1
PLB1	-0.0327271	1
PCMT1	0.0853635	1
C18orf54	0.0396584	1
PARP8	0.033998	1
KIAA1109	0.0308653	1
XYLT2	0.0471855	1
LIN7C	0.045921	1
GNB1	-0.1193498	1
RAB4A	0.0551131	1

ABHD16A	-0.0580688	1
CXorf38	0.0940174	1
GPR141	0.0201552	1
TEN1	-0.0698485	1
BAD	0.0433194	1
TTC39C	-0.0842894	1
DCTD	0.0212478	1
HCLS1	0.191678	1
UNC50	0.1006714	1
HAX1	0.0661109	1
HDAC7	-0.1088627	1
PISD	-0.0696555	1
PI4KA	0.05574	1
TNFAIP3	-0.1294121	1
DZIP3	0.0180975	1
ATG14	0.0441179	1
ZNF283	0.0472299	1
AKT1	0.0649029	1
CTBS	0.1009156	1
RIOK1	0.0395793	1
MAPK14	-0.0467586	1
DHX36	-0.0336181	1
CUTC	0.086311	1
CEP104	0.069669	1
REST	-0.0924064	1
CCDC90B	0.0569416	1
UBP1	-0.059958	1
HPS4	0.0109876	1
JKAMP	0.0783952	1
NUP93	-0.0297675	1
ZNF620	0.0489005	1
ACTR2	0.0800209	1
ASMTL	-0.1224832	1
FCHO1	0.0547425	1
ZCCHC9	0.0576542	1
AC002310.1	0.0707794	1
TUBD1	0.0384241	1
PPP1R21	0.0332636	1
LMBR1L	-0.1044994	1
IREB2	-0.0463712	1
TAF7	0.1751112	1
FKRP	-0.0436957	1
TRAPPC5	0.1204901	1
HGF	0.0573747	1
DHX34	0.0125767	1
AL035587.2	0.0525921	1
SP3	-0.1294581	1
HSD17B8	0.0563398	1
VPS13A	0.0711175	1
PIKFYVE	0.1079094	1

KLHDC1	0.0606589	1
SLC25A25-A	0.0303357	1
SNAPC5	0.0802182	1
ILRUN	-0.0532126	1
ASB7	-0.0454551	1
RETREG2	0.0911378	1
TTC9	-0.0604222	1
RNF40	-0.0040647	1
STK38	0.0578459	1
SNRNP35	0.0170405	1
ETFA	0.0852358	1
MAVS	-0.0616006	1
GNB4	-0.1382351	1
C16orf72	-0.1032009	1
POC1B	-0.0226693	1
AEBP2	-0.057725	1
CCDC167	0.0309315	1
DDX51	0.0618316	1
FBXO28	-0.0955541	1
PPP1R11	-0.0285693	1
AC090204.1	0.0514953	1
DOCK8	0.0936029	1
EIF2B5	0.0062671	1
GPS2	0.0002335	1
CCDC159	0.0099229	1
RNF4	-0.1579499	1
ATL2	0.0446198	1
PRR14L	0.0624017	1
POMGNT1	0.0496172	1
GOLGA8O	0.0802494	1
ABHD17A	-0.1174936	1
TAS2R4	0.0508	1
CCDC47	0.0147683	1
DHX38	0.0180717	1
CBFA2T3	0.0071014	1
ZNF451	-0.107197	1
ANAPC10	-0.0384968	1
INPP5A	0.0330796	1
SETD6	0.0302112	1
UPF1	-0.0870821	1
PRR34-AS1	-0.0729757	1
VPS26C	-0.1348765	1
DPP7	0.1347556	1
ANXA2R	-0.044861	1
FDX2	-0.083123	1
GBE1	0.0197269	1
AL009179.2	0.0631727	1
HAUS6	0.0695949	1
CLIP2	-0.0407367	1
COPS3	-0.0634042	1

SLC37A1	-0.0658683	1
SLC36A4	0.0558509	1
ATAD5	0.1088421	1
CBWD3	0.0225948	1
HIST1H2AH	0.0407512	1
CCDC124	0.0330639	1
MMADHC	0.039339	1
PARP16	0.0398089	1
POLR3E	0.0392865	1
MLXIP	0.0538656	1
SEPTIN1	-0.0758457	1
EIF3D	-0.1034827	1
TBC1D2B	0.0129324	1
MZT2B	0.035457	1
ECPAS	0.0417953	1
CLU	0.1364401	1
CPEB4	0.0565843	1
RAC3	0.0329085	1
DVL1	0.0301391	1
NBR2	0.0566065	1
ZNHIT1	0.1139992	1
SCD5	-0.0774524	1
UQCRC2	0.084593	1
RPLP0	0.0710162	1
IFFO1	0.0197845	1
STX4	-0.0021968	1
MMS19	0.0181113	1
PNPO	0.0663595	1
TMEM223	0.0160991	1
ZXDC	0.037006	1
SYNGAP1	-0.0523586	1
MTFP1	0.0049666	1
FYTTD1	-0.1148184	1
COX10-AS1	0.0474695	1
C12orf4	0.0400125	1
PPME1	0.0451463	1
CTDSP1	-0.1196087	1
RAB4B	0.0870232	1
NPIPB2	0.0455161	1
FBXL17	0.0761589	1
DANT2	0.0293998	1
SWAP70	-0.143865	1
MSH5	0.0230665	1
NCLN	-0.0269653	1
ANKRD36	-0.0275733	1
ORC6	0.0071415	1
CDC27	-0.1166563	1
SAP30L-AS1	0.0313778	1
IBA57	0.0224968	1
DDX60	0.0758711	1

FEM1C	-0.0864095	1
JRKL	0.1043577	1
PSMD8	-0.0589022	1
ICA1	0.0787457	1
ACSS1	0.0024985	1
MAST4	0.0376717	1
AC005520.2	0.0382912	1
OXR1	0.1200439	1
PARL	0.0590087	1
USP24	0.0386617	1
HINT3	0.0205057	1
FLI1	0.0992866	1
PDS5A	0.0645431	1
UTP25	0.0691449	1
TRPM7	0.0694592	1
TBC1D10C	-0.0656468	1
SURF1	0.0323044	1
PPP2R5E	0.0013738	1
TMEM102	-0.0717985	1
STX7	0.0316816	1
APC	-0.0868036	1
SCRN2	0.041484	1
B3GNT2	0.1465446	1
BTG1	-0.0299942	1
ALKBH8	0.0335855	1
SARM1	0.0528669	1
ZNF629	-0.0173343	1
ABTB1	0.0701387	1
DNAJC16	0.0854557	1
CHROMR	0.0518495	1
YBEY	0.0432879	1
CENPP	0.029587	1
DNAJC4	-0.0113253	1
MBTPS2	0.0283825	1
GBA	0.0193924	1
CCDC71L	-0.0637919	1
GPR160	0.0464248	1
MRPS7	0.0506127	1
MRPL20	0.1678609	1
GLG1	0.1020025	1
TASP1	-0.0605411	1
MRPL52	0.0411694	1
TEX10	0.0351337	1
DNPEP	0.0737121	1
RNASEH1	-0.0540713	1
ZBTB14	0.0562207	1
ZSCAN16	-0.0776596	1
CDC73	-0.1345461	1
UBE2L6	0.0225648	1
MED12	0.0939558	1

SOX18	-0.1079688	1
ST7L	0.0510822	1
RIF1	0.0883729	1
LEMD3	-0.0702021	1
ZNF528-AS1	0.048518	1
EOGT	0.0105205	1
AC015871.4	-0.0016786	1
SBNO2	-0.0972438	1
REXO5	0.061156	1
ZNF524	0.046836	1
RBKS	0.0289074	1
NDUFS1	0.0938988	1
RMI1	0.0916066	1
FRG1	0.0315703	1
METTL17	-0.0718449	1
NSMCE3	0.0679122	1
STOM	-0.0417377	1
SVBP	-0.1108605	1
AC103691.1	0.0340903	1
NUDT19	0.0442742	1
MLLT1	0.0224159	1
FNTB	0.0528546	1
TFDP1	0.0629679	1
EPS8	0.065125	1
PGS1	-0.0367526	1
VAV1	0.0679327	1
VAV3	0.0678296	1
KPNA5	-0.1055376	1
BACE1	0.052614	1
CNNM2	0.0405885	1
SPCS3	-0.1654496	1
TFDP2	0.1642848	1
TTF1	-0.0624457	1
EFCAB2	0.0772916	1
SON	0.103384	1
HARS	-0.0140654	1
AASDH	0.1028668	1
PLA2G4A	-0.0113421	1
RAC2	-0.1202157	1
PADI4	0.1084058	1
BAG6	-0.0278168	1
AL109811.2	0.0776063	1
MICAL3	0.0367085	1
LCLAT1	0.0080753	1
USP30-AS1	-0.0362438	1
SFT2D2	-0.1294429	1
PFKL	-0.0060934	1
UHMK1	0.1026895	1
ASCC2	0.0106535	1
PPP2R5C	-0.0813746	1

ATP7A	0.0644875	1
NUDT15	0.0524037	1
SELENOO	-0.0320211	1
KDSR	0.1135127	1
CTDNEP1	-0.0374549	1
TPI1	0.0662378	1
ANKRD42	-0.0766882	1
NEU1	0.0034343	1
FOXRED1	0.0215767	1
SMCR8	0.0311616	1
ULK1	-0.0571958	1
CLN5	0.0075347	1
TMEM273	-0.1118521	1
BCL2L2	0.043891	1
STMN1	-0.0618204	1
OCIAD2	-0.1203971	1
CLEC16A	0.0436413	1
AP2A2	-0.0034813	1
WDR54	-0.0559398	1
TSEN54	0.0460603	1
GAK	-0.0660696	1
MKRN1	-0.1108787	1
TRAM2-AS1	0.0653609	1
TMEM135	0.0350764	1
TRIM56	-0.0838649	1
NQO2	0.0048979	1
SPIN1	0.079119	1
MEPCE	0.0248914	1
CCDC28B	-0.0533303	1
ZNF793	0.0463791	1
UBQLN2	0.0420672	1
TANGO2	0.0207006	1
AC099778.1	0.0342161	1
SMCHD1	0.1150961	1
NENF	0.0971349	1
TXNRD1	-0.114969	1
AC009506.1	0.0639149	1
ADPGK	-0.0325127	1
RBM6	0.137081	1
COBLL1	0.1139026	1
C1D	-0.064546	1
SURF6	0.0881754	1
BMP1	-0.0507444	1
PTK7	0.0146702	1
MGAT5	0.0198956	1
LINC00884	0.0487696	1
RPRD2	0.0448467	1
DGKG	0.0412968	1
WFDC1	0.0465324	1
ZNF655	-0.1136934	1

MXRA7	-0.0400043	1
RBBP9	0.0442244	1
AL049840.4	-0.0593447	1
XPR1	0.0479573	1
ATP1A1	-0.1408024	1
QARS	0.0830039	1
FBXO22	0.0259071	1
NDUFC2	0.1197083	1
SDR39U1	-0.0141642	1
MCM5	-0.0906202	1
AC005562.1	-0.0424325	1
PAN3	0.0081741	1
NDRG3	0.0463041	1
SLC25A6	-0.1099421	1
EAF2	-0.0237395	1
VRK3	-0.0039672	1
AC108134.3	0.0568079	1
ZSCAN21	0.0266869	1
YLPM1	0.1670451	1
SUPT4H1	-0.0716072	1
DHX15	-0.110945	1
TEX9	0.0200086	1
PIGL	0.0406688	1
RPL9	0.0566136	1
HERC3	-0.063475	1
FHIT	0.0690349	1
HCG25	0.0391411	1
EBP	0.0083977	1
NPTN	-0.006146	1
BAHCC1	0.1315888	1
ACY3	-0.0704381	1
ZNF585B	0.0708611	1
GAPVD1	-0.0297551	1
AC007406.5	0.0448585	1
CRYZL1	-0.0626457	1
PABPN1	-0.1206039	1
ZNF714	0.0556883	1
SEC24B-AS1	0.0243986	1
WWP2	-0.0414135	1
FAM98A	0.0722303	1
VWA1	-0.0570327	1
MRPS14	0.042773	1
ACAA2	0.0801126	1
ATP6V1C1	0.0088526	1
STK4	-0.1501207	1
CBX5	0.1486007	1
SIAH1	-0.0482961	1
ERICH6-AS1	-0.1038465	1
ALG11	0.0498777	1
SH3BP5L	-0.0397991	1

KANSL1-AS1	0.0183076	1
GTPBP1	-0.0871557	1
PLAGL1	0.0515681	1
MORC2-AS1	0.0449664	1
CDK12	-0.0071877	1
KMT5C	-0.0562478	1
CDC37	-0.0708529	1
KCNAB2	0.08726	1
PRPF39	0.0384338	1
SAP30	0.0857897	1
NLK	0.0572732	1
POC1B-AS1	0.0706902	1
CENPL	0.0174161	1
TLK1	0.0772183	1
PPP1R2	-0.0991704	1
GPAT4	-0.0335377	1
MCMBP	0.0171437	1
YJU2	-0.0753244	1
AL390728.6	0.0399965	1
NUDT21	0.0149511	1
SLC35D1	0.0191114	1
PAQR7	0.0331484	1
MTHFD2	-0.1620619	1
MYL5	0.0160937	1
WDR60	0.1187597	1
SNHG12	-0.0904673	1
GOLGA7	0.0663812	1
DCUN1D3	0.044249	1
MMD	0.028239	1
BORCS5	0.0547887	1
VAMP2	0.0398073	1
PGM3	0.0227468	1
RFC2	-0.0303772	1
TAF4	0.0238952	1
RFC3	0.0362045	1
SWT1	0.037618	1
TIGD1	0.0464537	1
ATG5	-0.0634706	1
FAAP20	0.1047504	1
TP53BP1	0.1286132	1
SIPA1	-0.0639748	1
VCPIP1	0.0080293	1
TOMM34	-0.0126632	1
MNT	-0.1055234	1
AC096992.2	0.0379063	1
ZNF251	0.0167933	1
MEA1	0.0465734	1
INAFM1	-0.0334112	1
DBF4B	0.0252228	1
SEMA4A	0.006435	1

MARCH2	-0.0655843	1
GANAB	0.1053375	1
TMEM128	0.0432689	1
ZKSCAN2-D	0.0354665	1
AL035446.1	-0.0270176	1
PARP2	0.0124488	1
FAM241A	-0.0639962	1
SPHK2	0.025699	1
C4orf48	-0.073662	1
PRKCI	-0.024024	1
CINP	0.0528048	1
DYNC2LI1	0.04265	1
DUS4L	0.0484358	1
AL138724.1	-0.0485025	1
EHD2	-0.0616642	1
PEA15	-0.0780869	1
TENT2	-0.0715842	1
PGAM1	-0.1357967	1
PRMT1	-0.0267634	1
AGA	0.0534278	1
RASSF7	-0.0419661	1
ALG10	0.0302513	1
ITPR1	0.0876908	1
TRAF6	-0.0735981	1
SLC25A20	0.0199627	1
SNIP1	-0.0534961	1
HS1BP3	-0.0290315	1
BAZ2B	-0.1188761	1
MTHFD2L	-0.1104894	1
USP21	0.0065391	1
LGALS8	0.0700904	1
FKBP14	0.0487828	1
PEAK1	-0.113451	1
MAML3	0.1435045	1
HDAC5	-0.0996027	1
E4F1	-0.0666327	1
WDR83OS	0.0306776	1
KPTN	-0.0213811	1
TMEM62	-0.0260646	1
MITF	0.049685	1
GABPB2	0.0735289	1
FXR1	-0.0825155	1
VPS8	0.0614254	1
SSR3	0.1200643	1
ZNF345	0.0280979	1
GALNT10	0.0249864	1
ZNF672	0.0404015	1
SPSB3	-0.0988729	1
MTG1	0.0603553	1
ZSCAN32	-0.0085851	1

C6orf120	0.0121494	1
ARHGAP19	0.0140495	1
LAS1L	0.1077221	1
TBK1	-0.0362172	1
CWC22	-0.082552	1
RPF1	-0.0116754	1
ZNF184	0.0285282	1
JMJD4	0.0057403	1
BPHL	0.0291817	1
ZFYVE16	-0.0372091	1
MRPL48	0.0471091	1
STT3B	-0.1199444	1
PHKG1	-0.0373383	1
RNPC3	0.0787641	1
C17orf80	0.053877	1
MYO9B	-0.039508	1
ZFPL1	-0.037179	1
ANKHD1	0.0642267	1
MROH6	0.0401088	1
SLC25A32	-0.0232133	1
COQ10B	-0.0112394	1
CTBP1	0.0786004	1
TOB1	-0.087208	1
SCYL2	-0.0387767	1
PKIG	-0.0684633	1
MTERF1	0.0472379	1
SLC2A5	0.0078162	1
TBP	0.0195155	1
DESI1	0.04866	1
PANK2	-0.1123422	1
WAPL	-0.0685636	1
BDP1	0.0507369	1
SLC10A7	0.0221837	1
FGD3	-0.0373048	1
BYSL	0.0268296	1
CSDE1	-0.1686191	1
EIF4G2	-0.0822291	1
AC008969.1	0.0836718	1
MTRR	0.0267533	1
ZNF576	0.0559895	1
GLRX2	-0.0758364	1
DHRS7	-0.0116357	1
HS6ST1	0.0141416	1
AFMID	0.0570208	1
BTF3L4	0.0571201	1
DNAJC8	0.1117778	1
WIPF2	-0.1084398	1
GATD3A	0.0497102	1
PDE6G	-0.0446943	1
GNB1L	0.0388924	1

RMDN3	0.010241	1
P2RY11	-0.0513553	1
PKNOX1	-0.0025999	1
MZT1	0.075491	1
AC002467.1	-0.0240195	1
SLC38A6	0.020437	1
NOC2L	0.0413856	1
DDX20	-0.0802288	1
ANGEL1	0.0266727	1
ARMT1	-0.002171	1
LINC00211	0.059549	1
PSMA2	-0.0513951	1
KIF13A	0.0474351	1
CHST14	0.0594382	1
KTI12	0.0659808	1
PHF19	-0.0981725	1
SLC7A6OS	0.0065375	1
POLH	0.0398355	1
MTHFSD	0.0167099	1
SCAMP3	0.1154016	1
DDX24	-0.0838627	1
KLHDC2	-0.0379156	1
FBXO21	-0.0538894	1
CEP295	0.0425081	1
ENOPH1	0.0515957	1
ZNF669	0.0036323	1
ARSA	-0.0755594	1
TGS1	0.0474024	1
C10orf143	0.0401599	1
DNAJC24	0.0387945	1
RBCK1	-0.0488608	1
ACOT4	0.0372174	1
PPAN	-0.0617938	1
JADE1	0.0558812	1
DYSF	-0.0258362	1
CTU1	0.0393586	1
GOLGB1	-0.0332773	1
ZNF677	0.0533003	1
AC009779.2	0.0336124	1
TLR9	-0.051918	1
CWC15	-0.0723315	1
TMEM141	0.0724112	1
JMY	0.0331807	1
WIZ	0.0088827	1
ZNF649	0.0514112	1
NKTR	-0.1561731	1
SCARF1	-0.0868379	1
PHF10	0.0367988	1
LINC01806	-0.0420549	1
FUT10	0.0840041	1

CGRRF1	-0.1121482	1
ZMYM6	-0.0073909	1
SLC37A4	0.0454051	1
RNF8	0.0627231	1
CSTA	0.1045828	1
ZNF490	0.0433716	1
SCAMP2	-0.0766629	1
MAPKAPK5-	0.099708	1
HSPA14	0.0015049	1
METTL2A	0.0449821	1
SCAI	0.0841343	1
FKTN	0.0861604	1
SOCS2-AS1	0.0239249	1
RAB27B	0.0103413	1
IFI6	-0.0052458	1
MAGED1	-0.0005704	1
CHD1L	0.0392609	1
SART1	-0.0353375	1
CANX	0.1210213	1
EXOC1	0.0008172	1
PSME3	0.0187381	1
GRIPAP1	-0.0807294	1
CNPY3	-0.0927394	1
SAP130	0.0355742	1
NCOR2	0.0100491	1
PDHX	-0.0042051	1
TMEM179B	0.0676939	1
SAR1B	0.0540433	1
GSR	0.0486292	1
VTA1	-0.0652924	1
STX3	-0.071894	1
CCL23	0.0428862	1
PRPF18	0.0491497	1
AP1S3	-0.060105	1
FNDC3B	0.0088148	1
FAM32A	-0.0081232	1
BIRC6	0.0339651	1
ZNF8	0.0428421	1
PPP1R15B	-0.0736276	1
AK3	0.0687867	1
FASN	0.0674781	1
ZNF331	0.0328062	1
ABHD17C	0.019338	1
LUC7L2	-0.1078254	1
MIS12	0.0241587	1
EYA3	-0.0807411	1
FKBP5	-0.0368333	1
NCOA2	0.0267031	1
DHRS1	-0.0471819	1
ICE1	-0.0392379	1

KIF3B	-0.0623391	1
SLC1A5	-0.1129283	1
SMARCAL1	0.0757903	1
KATNB1	-0.0555214	1
LY86	0.0541089	1
LANCL1	0.0243662	1
SLC38A5	0.0389136	1
STAT6	-0.008142	1
ZNF160	-0.1131287	1
FYCO1	0.0401904	1
VASH1	-0.0610891	1
IFT80	0.0216363	1
AL391069.2	0.0497447	1
MBTD1	0.110742	1
PPARA	0.052317	1
ADH5	0.1268817	1
PREB	0.0382067	1
TMEM170A	-0.0486582	1
ELOF1	-0.0802887	1
C2CD5	0.0211311	1
SMYD3	0.157457	1
ATG4A	0.0264447	1
AZIN1	-0.0741707	1
NEMP1	0.0553786	1
ZNF675	0.0764119	1
MSANTD3	0.0215153	1
ZNF317	-0.0024802	1
PPP1CA	-0.0598414	1
KIAA1143	0.0924068	1
QTRT2	-0.0394787	1
ATRIP	0.0280618	1
PTPN18	0.1049843	1
FBXO46	0.0125566	1
SEC11A	-0.0730804	1
PROSER3	-0.0440058	1
ZNF764	0.0147434	1
HPS5	-0.0124377	1
KARS	0.1040927	1
CREB3L2	0.0287229	1
MYDGF	0.0502231	1
EXOC6	0.0640475	1
ZNF791	-0.013254	1
BLZF1	-0.0724022	1
ZNF529	0.0678731	1
ZSCAN9	0.0148182	1
ILVBL	0.0395632	1
SETD9	0.0389167	1
ATP5F1C	0.1069666	1
CYBC1	0.0469996	1
POP4	-0.0352857	1

TOP3B	0.0022085	1
HUS1	-0.0485569	1
SOCS1	-0.0068649	1
ERLIN2	0.0749459	1
COG5	0.0755005	1
UBR2	0.058663	1
NBR1	0.0920522	1
TAF5	0.0282992	1
UCKL1	-0.0697039	1
SLC25A28	0.0293156	1
CLUH	0.0342257	1
CNOT10	0.0034878	1
PBRM1	-0.0358037	1
HPS1	0.0463744	1
RRAGD	0.0351254	1
AL590764.1	0.0502926	1
ZNF460	0.0349181	1
SHQ1	0.0928059	1
LSM7	0.0955233	1
USP16	-0.1476003	1
USP7	-0.0582053	1
PAIP1	-0.0106888	1
FAM168B	-0.1005089	1
ZDHHC6	-0.0105136	1
RALB	0.0056182	1
INO80E	0.0042975	1
TRAPPC6B	-0.0799274	1
AHCY	0.0330953	1
MAF1	-0.0735818	1
PDPR	0.0279286	1
NBPF1	0.010415	1
NFKBIL1	0.0500428	1
SCAF4	-0.0826296	1
PBX2	-0.0015074	1
SNX16	0.0390304	1
DUSP5	-0.0960006	1
GTF2A1	0.058777	1
CDK5	0.0384839	1
EZH1	-0.0070982	1
LCT	-0.0399278	1
SLC52A2	-0.017262	1
ISG15	-0.0771426	1
U2AF1	0.0064133	1
PILRB	0.009661	1
CEP85	0.0376003	1
INPPL1	-0.0268701	1
METTL15	0.0493224	1
PDE6D	0.0306781	1
MED17	-0.0217274	1
MINDY2	-0.1159725	1

NUP88	0.0331181	1
TRIM35	-0.0479606	1
BOLA1	0.0699665	1
LINC00641	0.0368412	1
MFF	0.0724749	1
AC006504.5	0.0377881	1
BCL2L11	-0.0251865	1
ADA	0.0874138	1
L3MBTL1	0.0543773	1
QRICH1	-0.0653076	1
CRBN	-0.0419007	1
HNRNPH2	-0.1319389	1
GHITM	-0.0018667	1
TBC1D1	0.0468552	1
USP51	0.0663628	1
TPCN2	-0.0481469	1
LYRM2	0.0497922	1
SP1	0.0099525	1
ACBD5	-0.0086809	1
HTATSF1	-0.0378095	1
AL118516.1	-0.0868079	1
NRBF2	0.0503933	1
RSU1	0.0527133	1
ZNF667-AS1	-0.0522656	1
LMO2	-0.0822279	1
FASTK	-0.0412848	1
AGAP3	-0.0517841	1
GCLM	-0.0208866	1
CLDN10	0.0576402	1
METTL16	0.0095661	1
PUS1	0.0770891	1
MINDY1	-0.0918112	1
SLC66A2	-0.09371	1
PEX7	0.0367931	1
CYB561	-0.0887209	1
ADPRM	0.0807063	1
MMS22L	0.059843	1
ZCCHC17	0.0257612	1
EI24	-0.0233471	1
REEP3	0.086142	1
RASAL2	0.0549062	1
MFSD4B	0.0567783	1
COMMD1	0.0279511	1
TMEM165	0.0531376	1
AP1G2	-0.0609543	1
GSTO1	0.071607	1
RNF144A	0.0785624	1
PFDN5	0.115979	1
RAP2B	-0.0374542	1
BUB1	0.045947	1

GOLGA4	-0.0429032	1
FAM20B	0.0597917	1
MED21	0.0497412	1
ANO10	0.0032832	1
PPP2R3B	0.0614379	1
TMEM184C	0.0282381	1
CRLF3	0.023764	1
S100B	0.0346939	1
USP30	-0.0425581	1
ZNF627	-0.0578021	1
PSMD10	0.0220802	1
NFATC2IP	-0.0756808	1
PIN1	-0.0846816	1
IARS2	0.0417653	1
TRIM37	0.0078228	1
STXBP3	0.0813078	1
SELENOI	-0.0295773	1
NANS	0.0198211	1
PDE12	0.03652	1
RGS10	-0.0655422	1
IFNAR1	-0.0690728	1
OGT	-0.1060303	1
MIEN1	0.0570273	1
RAP2C	-0.1319907	1
TBC1D23	-0.0888513	1
ZNF721	0.1209557	1
FH	0.0822847	1
ACP6	0.0607734	1
BTG2	0.1934132	1
PHACTR4	0.0387391	1
LYRM9	0.0349935	1
GPSM1	-0.0688654	1
DNHD1	0.0287339	1
TAB3	-0.051909	1
MPHOSPH1	0.1196691	1
CMTR1	0.0110705	1
ACAD10	0.0535039	1
CARD8	0.1168261	1
CLCN7	0.0875318	1
ITPKB	-0.0435126	1
AC016727.1	0.0274791	1
RAB13	0.1230702	1
NUDT8	0.0534513	1
BRWD3	0.0505544	1
ADIPOR1	-0.1037842	1
EPHB6	0.0794568	1
CHKB	-0.0282423	1
AC004148.1	0.0346656	1
PIK3R4	-0.050003	1
LRMP	0.0507099	1

DARS2	0.0480023	1
IFT81	0.0222918	1
COX14	0.0543095	1
HDGFL2	-0.0074047	1
BPGM	0.0043724	1
FOXJ3	-0.0537636	1
ZNF577	0.0558039	1
NFATC3	0.0411179	1
LINC00685	-0.0257058	1
BRAT1	0.0309426	1
HSPB1	-0.0653913	1
CDK2AP2	-0.0918783	1
SLC25A29	-0.0816495	1
ALG5	-0.0114875	1
TIMM10	0.0625425	1
NELFE	-0.0091675	1
MRPS2	0.0878596	1
DNAJC13	0.1094708	1
RBBP5	0.0032949	1
ZNF224	0.0388812	1
SP100	-0.0825489	1
DDT	-0.0850372	1
ZNF562	0.0140232	1
LDLRAP1	0.0099515	1
ACAP2	-0.0574958	1
ZBTB24	0.0375822	1
SLC35E2B	-0.0487352	1
GPAM	0.0490104	1
SIVA1	-0.0215954	1
RTN4R	-0.1446747	1
ASH1L-AS1	-0.0266501	1
FUT11	-0.0041372	1
AC110285.2	-0.004556	1
VCPKMT	-0.0703955	1
SH3BGRL	0.0957359	1
BIVM	0.0522809	1
TCP11L1	0.0039746	1
FAM81B	-0.0425019	1
BCL2L13	-0.0986755	1
CCZ1	-0.1655628	1
PIK3CG	0.0187402	1
CDIPT	0.0375763	1
OTULIN	0.078852	1
TUFM	-0.0675911	1
TAF5L	-0.0745031	1
WDR26	-0.1259277	1
BEX4	-0.1143881	1
POLR2A	-0.0950376	1
NSD2	-0.0811611	1
SERPINI1	0.0427805	1

MAML1	-0.0009247	1
GPR108	-0.0241264	1
CYB561D1	0.029721	1
COG1	-0.0616195	1
HSD17B10	-0.0520474	1
SYNRG	0.0546483	1
MSL3	0.0076676	1
AL391121.1	-0.0560196	1
PELO	-0.0195542	1
ZNF185	0.0318349	1
SCAP	0.0648177	1
UBE2V1	0.0861722	1
ZNF143	0.056896	1
BRMS1L	0.0344244	1
GMNN	0.0086743	1
KPNB1	0.1653069	1
ATAD3B	0.0004767	1
SELENOS	-0.1462299	1
PDCL	0.0435477	1
STON2	0.1277435	1
VOPP1	-0.013692	1
AC009120.2	0.0657122	1
ERCC8	0.01169	1
CXCL3	0.0910228	1
WDR37	-0.0577401	1
MAPK9	0.0754139	1
RUNDC1	-0.0235497	1
SMUG1	0.0362504	1
PFDN1	-0.1067538	1
KLHDC3	0.0934564	1
BLOC1S1	0.0693179	1
EPB41L4A-A	0.1238619	1
SDHC	0.076278	1
PEX10	0.0095856	1
DNAJC7	-0.0787721	1
PIP5K1C	-0.0387543	1
POP7	0.0266818	1
SLC23A2	0.0119045	1
HLA-DQB1	-0.0423872	1
EXOG	0.0524338	1
BRK1	-0.1611488	1
CCDC43	0.0064068	1
VAMP7	-0.0544525	1
MACC1	0.0387978	1
RAP1GDS1	0.003306	1
ZNF273	0.0329142	1
ETAA1	0.084794	1
AC087239.1	0.0260361	1
GDE1	0.0303608	1
RTCA	-0.084815	1

ARHGAP26	0.0305281	1
TRAPPC2	0.0239252	1
AZI2	-0.0136215	1
TXN2	0.0636395	1
RAD52	0.0281615	1
CBFB	-0.0566364	1
LZTFL1	-0.0599145	1
THBS3	-0.0747724	1
RBM43	0.0920091	1
ITGB2	-0.1426896	1
SS18L1	-0.009381	1
DDX6	-0.0804153	1
MROH7	0.0256519	1
AC008074.2	0.0114017	1
HIST1H2BE	0.0222389	1
TTC9C	-0.0445967	1
HLA-DRB5	-0.1281011	1
HOMEZ	0.0302078	1
HEATR5A	0.0198738	1
MTREX	0.0555604	1
SECISBP2	-0.0743938	1
ARL1	0.0409768	1
ZNF69	0.0443565	1
SLC39A1	-0.0734733	1
PTCD1	0.0396928	1
RGL2	-0.0658855	1
WDR18	0.044149	1
MGAT4B	-0.0766807	1
DTNB	0.0397232	1
MARCH7	0.0045241	1
LINC01003	-0.092874	1
ZNF506	0.0780129	1
MYD88	0.0253243	1
MT-CO1	0.0570233	1
NECAP1	-0.0119932	1
SEC61A2	-0.0819424	1
MRRF	0.0625064	1
FMR1	-0.0395804	1
CHCHD4	0.0379683	1
NLRC5	0.0114996	1
CRACR2A	0.0334734	1
RPP30	0.0405414	1
MICALL2	-0.0486428	1
HLA-DPA1	-0.127073	1
LPCAT1	0.0515671	1
SIRT5	0.0627139	1
EP400	0.0950034	1
TBRG4	0.0040691	1
TRBVB	-0.0526252	1
ZNF277	0.0524645	1

AL158152.1	-0.023893	1
ARNT	0.0143615	1
CCDC130	-0.0044931	1
MRPL2	0.040265	1
ASB6	-0.0808323	1
SUMF1	0.0612664	1
C1orf35	-0.0652417	1
ADAM15	-0.1054206	1
SLK	-0.0666946	1
SNRK	-0.0764563	1
ZNHIT3	0.0115035	1
ACOX1	0.032462	1
NOP14-AS1	-0.0519548	1
LNCAROD	-0.0105839	1
INTS5	-0.001505	1
RNASET2	0.1282909	1
SLC35F5	-0.0578475	1
GALNT11	0.1023953	1
RXRB	0.0215997	1
CCDC189	-0.031716	1
C18orf25	-0.1067737	1
AUTS2	0.0404576	1
MRPS11	0.1164627	1
AC022098.1	0.0479441	1
C4orf46	0.0371177	1
ISY1	0.0328825	1
SBNO1	0.0768347	1
MBNL2	0.037844	1
VPS33B	-0.0399258	1
TTC31	0.0571295	1
POLR2D	0.0744783	1
OTUD5	-0.0770963	1
SETD1A	-0.0028769	1
PATL1	-0.0790664	1
FBXW2	-0.0680876	1
PI4K2B	-0.0575737	1
SAAL1	0.0157974	1
UFL1	-0.0829054	1
NCBP1	0.0577123	1
AC107464.3	-0.0156104	1
TMEM248	-0.0640494	1
KCTD18	0.0297976	1
CEP70	0.0677863	1
HEXA	-0.0419312	1
MIR181A1HG	0.0015779	1
PRDM4	-0.0326233	1
TCIRG1	-0.0996504	1
ADAM9	-0.0487145	1
MYPOP	0.0396258	1
ZNF765	0.0597879	1

SLC25A43	0.0438233	1
LINC01089	0.0416595	1
PIK3CB	0.0605966	1
HSBP1	0.0158741	1
ERAP1	0.0667707	1
SSBP2	0.1162405	1
TRIM68	0.0410372	1
CUL2	-0.0802264	1
CHUK	-0.0082849	1
MTMR9	-0.111753	1
JPT2	-0.0209936	1
UMPS	0.0541441	1
NOM1	0.0609701	1
WDR55	-0.024679	1
CEP89	-0.0526518	1
TSFM	0.0656129	1
SEC11C	-0.0263479	1
ATP6V1H	-0.067209	1
KIAA1191	-0.0076662	1
MTM1	0.0592899	1
TSR3	-0.0642927	1
HPS6	-0.0891681	1
CHST7	-0.0492199	1
PRDX6	-0.0676936	1
AP5Z1	-0.0812001	1
FTO	0.0596879	1
AP002360.1	0.062493	1
SRPK1	0.1033519	1
CLIC2	0.1096055	1
TMTC4	0.0175085	1
ATXN7	0.049923	1
MTERF3	-0.0292726	1
AFF1	-0.0416881	1
RNF170	0.0190912	1
ATP6V0A1	0.0067026	1
HYI	0.0355512	1
PYCARD-AS	-0.0400374	1
MYOF	-0.014287	1
RNPS1	-0.1028914	1
ZNF347	0.0455668	1
EXOSC4	-0.0269309	1
PARP9	0.0644321	1
FNIP2	-0.0781276	1
TOP1	-0.0411638	1
PSME1	-0.0412788	1
SOAT1	0.0110665	1
PRADC1	0.0373221	1
SACS	0.0133821	1
RANBP3	-0.0417435	1
STMP1	0.1168464	1

TSTA3	0.0011789	1
FAHD1	0.0640864	1
WRAP53	0.0161484	1
MAP3K13	-0.0691781	1
TOR3A	0.0017928	1
WDR76	0.032448	1
HADHB	0.0570694	1
ZNF790	0.0209719	1
PPP3CB	0.0403432	1
STX5	-0.0978524	1
NCSTN	0.0387495	1
F8A1	0.0346535	1
AC139887.2	-0.1202877	1
USPL1	0.0434874	1
GADD45GIP	-0.0090726	1
TXNIP	0.0070325	1
PURA	0.1019313	1
BCORL1	0.0165961	1
BET1	0.0476237	1
DUSP22	-0.023857	1
RWDD2B	0.0568155	1
GEMIN8	-0.0164096	1
AC064807.1	0.0695387	1
MFSD5	-0.0658924	1
DDB2	-0.0906556	1
FLCN	-0.065856	1
ADGRE1	0.0462868	1
AP000766.1	0.0321417	1
MIF4GD	0.0665111	1
TRIM26	-0.0605879	1
ZSCAN16-AS	0.1108064	1
ZNF418	0.0128752	1
SBF1	-0.0438891	1
MRPL43	0.0478065	1
TRMT2B	0.0265276	1
DAP3	0.0786195	1
INO80B	-0.0030939	1
C5orf22	0.0241547	1
UBQLN4	0.0340924	1
NUP210	0.0779077	1
PRIM2	0.0293925	1
ARL5B	0.0623392	1
BCDIN3D	0.0342841	1
ADCK2	0.0219198	1
VAMP1	-0.0649206	1
SIRT3	-0.0332614	1
ABCF3	-0.0239372	1
ZNF662	0.0449884	1
CMAS	0.0816307	1
DDX21	0.1719197	1

TRAPPC10	-0.0820978	1
CDK2	0.0167923	1
WBP2	-0.0790509	1
DNMT3B	-0.0341167	1
TEP1	-0.0433675	1
GGA3	-0.0159816	1
TMED4	-0.0237823	1
NUP188	0.0615055	1
PRR3	0.0223357	1
LINC02384	-0.0578653	1
ZNF92	0.0936009	1
FCHO2	0.0280928	1
LGALS3	-0.0444322	1
WDFY1	0.0123352	1
OXSM	0.016157	1
GPR107	-0.0913149	1
TNPO2	0.0123643	1
PRORP	-0.0663428	1
PPIL1	0.0636918	1
MEAK7	-0.0294262	1
GET3	0.0059484	1
CRTC3	-0.0881752	1
ZNF587	0.0307929	1
SLC17A9	0.0325427	1
NOL11	0.0351926	1
AP3D1	0.0273958	1
VAMP4	0.0658531	1
UNC45A	-0.0161149	1
NCOA4	0.0677501	1
AGAP9	-0.0115337	1
MAD1L1	0.0609427	1
EEF2	0.1042356	1
PKN1	-0.0223883	1
TDG	0.0597058	1
THAP7	0.0641263	1
CTTNBP2NL	-0.0491238	1
SRSF10	0.0857448	1
SPG7	-0.0033482	1
RBMX2	0.0724408	1
ZDHHC24	0.05	1
GOLGA1	0.0535976	1
UCN	0.0285262	1
LRRC40	-0.0543298	1
SHC1	-0.0281839	1
KAT7	0.0258255	1
BTBD1	0.0136509	1
ZNF646	0.0157215	1
PIK3C2A	-0.0146991	1
ENTPD7	-0.0094714	1
PARVB	-0.0586354	1

KBTBD6	0.0274813	1
RCC1L	0.0147862	1
WARS	-0.1028367	1
CCDC112	0.0581048	1
UHRF1BP1	-0.018249	1
KLHL36	-0.0664209	1
FBXO41	0.0424882	1
MDC1	0.020017	1
OSBPL9	-0.0643698	1
NAA35	-0.0678871	1
EID2	0.0305954	1
EIF6	-0.0339747	1
TRAPPC2B	0.012907	1
RNF14	-0.0519281	1
SBF2	0.0033833	1
SLCO3A1	0.0236689	1
ICAM2	-0.0533466	1
ZNF761	0.0647632	1
DAPK1	0.1050708	1
FBXW9	0.0428841	1
TSPAN32	0.0206184	1
H2AFV	-0.1211644	1
HIF1AN	0.0004333	1
CAMKMT	0.0386134	1
TXLNA	0.0840902	1
PRSS21	0.0639296	1
CRIPT	0.0226422	1
SMPDL3B	0.0530052	1
UCHL3	-0.0162859	1
ISYNA1	-0.0562386	1
ZBTB17	-0.0529548	1
DNAL4	-0.0306476	1
HMGXB3	0.0787899	1
FBXO33	-0.1016404	1
DGAT1	0.0230416	1
MZF1	0.0501325	1
SCNM1	-0.0280564	1
PUM2	0.0188335	1
LINC01678	0.0373467	1
UBLCP1	-0.0229008	1
C19orf81	0.0288308	1
ZNF337	-0.0074723	1
APOL3	-0.0175878	1
INPP5F	-0.0659577	1
ZNF292	-0.1167071	1
POLA2	0.0239024	1
CASC3	0.0027973	1
TMEM183A	-0.0080475	1
RABL2A	0.0041487	1
GSTCD	0.0454971	1

RNPEP	-0.054184	1
C2orf68	-0.0376916	1
SUV39H2	0.0337279	1
ZNF274	-0.0677117	1
TRIM52	0.0623673	1
ETHE1	-0.0825225	1
PDZD11	0.0116946	1
ZNF805	-0.0145273	1
RBMX	0.0682841	1
RAB20	-0.0649669	1
ZNF654	-0.0163928	1
RPRD1A	-0.005171	1
PCNX1	-0.0097862	1
SUGP2	-0.0112097	1
LYRM1	0.1258824	1
PARP15	0.0328776	1
MED11	0.0412697	1
SEL1L	-0.0746034	1
B4GALT7	-0.0243408	1
ZNF724	0.0706854	1
SCOC	0.0200265	1
RTCB	-0.0069601	1
MORN2	0.0109293	1
SF1	-0.1477764	1
MGME1	0.1152115	1
PET100	-0.0857853	1
MRPS16	0.0981361	1
SPPL2B	-0.0028223	1
FAM3A	-0.0242366	1
PLPP5	0.0591916	1
AC010623.1	0.0194156	1
APEH	0.0929513	1
VPS45	0.0456789	1
SREK1IP1	0.0229422	1
RBM26	0.1140421	1
GABPB1-IT1	0.0101969	1
CDC40	-0.0951931	1
NDUFA10	0.0692608	1
NUMA1	0.0783483	1
ZNF511	-0.0346458	1
RABEP1	0.0704205	1
SEMA4D	0.0779357	1
FHOD1	-0.0871544	1
ARHGAP25	0.100019	1
PRKAG1	0.022137	1
C6orf89	0.0275494	1
PEBP1	0.0999531	1
KXD1	0.0267152	1
OFD1	0.0674671	1
MATR3.1	0.046552	1

ZNF84	0.103331	1
NHEJ1	0.022113	1
PRR13	0.0744089	1
TRMT6	-0.0204031	1
PSMD1	0.0819928	1
CCDC22	0.0284028	1
NDUFA1	0.0940784	1
GOLGA3	0.0181211	1
DR1	-0.0160128	1
NOL9	0.0147026	1
GPX7	0.0928769	1
BACH1	-0.0314483	1
UBR4	0.0072375	1
GOLGA8Q	0.0544786	1
UNC93B1	0.0244511	1
PPP1R3D	0.0831194	1
SLC30A5	0.013467	1
SACM1L	0.004861	1
PIAS1	-0.1280205	1
ABCD3	0.0385014	1
LYL1	0.0517954	1
AMZ2	0.0366331	1
DNAJC9	0.0510849	1
CRCP	0.0559852	1
KLHL15	0.0405416	1
SFXN5	0.0647049	1
DDX50	0.0901345	1
CSKMT	0.0266786	1
ZNF789	-0.0544099	1
PTOV1	-0.102071	1
PEX14	-0.0241075	1
AC006141.1	0.064533	1
MRPL44	-0.0137327	1
ZBED4	-0.072723	1
MPHOSPH6	0.0114201	1
PRKCB	0.0874448	1
RP9	0.046163	1
GSTK1	-0.1065501	1
SLC38A2	-0.0772733	1
C9orf85	0.0314209	1
GMDS	0.0014107	1
PHKA2	0.0384384	1
TMEM65	0.0438109	1
MED14	-0.0461436	1
MALSU1	0.017584	1
ZBTB33	0.0583483	1
SLC1A4	-0.0774927	1
ADAMTSL4	-0.0820404	1
RAD9A	-0.0464833	1
IMPDH1	0.0675669	1

GOLPH3	-0.061915	1
ARAP1	0.0330822	1
HOXA4	0.0193272	1
GPR89A	0.0227045	1
ARHGEF9	0.0617361	1
PSMD5	-0.0175957	1
TSTD2	-0.0116155	1
ZKSCAN3	0.0440389	1
RPS3A	0.0586514	1
PTGIR	-0.1170098	1
DRG2	0.0792415	1
UVRAG	0.0619396	1
MGAT4A	-0.0142665	1
ANKIB1	0.08964	1
STT3A	0.0811098	1
CRADD	0.0277712	1
STK11IP	-0.0132169	1
MED6	-0.1120869	1
PPIG	-0.0445519	1
B3GAT3	-0.0272777	1
KDM1B	0.0614865	1
HLA-DRA	-0.0529323	1
MSRB1	0.0047088	1
TMEM167B	-0.1156833	1
STK38L	-0.1464976	1
CAND1	-0.1285498	1
GTF3C5	-0.0544473	1
AKT2	-0.0442028	1
SEC24C	-0.0817203	1
AKAP11	0.0711404	1
MOSPD2	0.039296	1
PSMD9	-0.068208	1
AP001816.1	-0.054921	1
TAOK2	-0.0236438	1
CES2	0.0544135	1
RRP36	-0.0830179	1
TEX2	0.0198941	1
SLC8B1	-0.0183963	1
GFPT1	-0.0749639	1
JHY	-0.0317029	1
PEX6	0.0488988	1
COX5B	-0.0747258	1
SLC52A3	0.0246423	1
HSF2	-0.0540724	1
SPSB2	-0.0834291	1
THAP4	-0.0017691	1
MPPE1	0.0438616	1
ZMAT3	0.0458893	1
PTPN7	-0.0274355	1
ZNF319	-0.0291804	1

CCDC88A	0.0177881	1
DDX54	0.0474202	1
FIP1L1	-0.0773804	1
GNE	-0.0004136	1
FEM1A	0.0225554	1
CCDC92	-0.0175603	1
C5orf51	0.0197941	1
COG4	0.0314976	1
COPG2	0.0473117	1
MBTPS1	0.1122939	1
TFR2	0.0268176	1
GTF2H2	0.0440091	1
MTMR2	-0.0288809	1
SINHCAF	-0.0782218	1
FMO5	0.0385471	1
LINC00513	-0.0623441	1
SNX30	0.0196476	1
SUPT5H	0.0068479	1
C8orf82	0.0432794	1
SLC25A15	0.0093775	1
AL049840.2	-0.0655969	1
PCNP	-0.0983693	1
PRR14	-0.0881363	1
MYNN	-0.0459111	1
DRG1	-0.0112327	1
Z95331.1	-0.0444286	1
NATD1	0.0097691	1
YWHAE	0.0602891	1
GTF2B	-0.106452	1
AC253572.2	0.1256556	1
STAG1	0.0851273	1
ZCCHC8	-0.0087215	1
FGFR1OP2	0.0985601	1
LINC01122	0.0406716	1
RAB3GAP2	-0.057157	1
ZNF830	0.142499	1
MGMT	0.000525	1
EFR3A	-0.0659704	1
ZDHHC13	0.0308739	1
MRI1	0.06772	1
TAF1B	0.0251314	1
VPS35L	0.0627305	1
ZNF581	-0.0078307	1
ILK	-0.0048369	1
GLMP	0.0254906	1
ARFRP1	-0.0418055	1
USP32	-0.013894	1
KIAA0319L	0.0919267	1
CYB5R1	0.0551578	1
DCAF13	0.0820927	1

WDR73	-0.0673884	1
IGBP1	-0.020374	1
MBOAT1	-0.0635669	1
TTL	-0.0614859	1
DDX19B	-0.0667504	1
PPIE	0.0830352	1
ZNF316	-0.041721	1
TXNDC15	0.0326027	1
TNFRSF25	-0.0871104	1
PRPF40B	0.0415539	1
MDP1	0.0762293	1
RNGTT	0.0227438	1
SYNGR2	-0.0444415	1
ZNF148	-0.067109	1
SAMD9L	0.0081102	1
POLR1C	0.0527917	1
KLHL21	-0.0284905	1
AC002454.1	0.0765552	1
UTP23	0.0850148	1
ASCC1	-0.0723447	1
TMEM70	0.0135208	1
AP1S2	-0.0261122	1
RNF31	-0.0184519	1
TMEM138	-0.0103089	1
PSD4	-0.0128672	1
CASP9	0.0031946	1
DDX39B	0.0221769	1
MVD	-0.061925	1
ZNF93	0.0396839	1
APOBR	-0.0422967	1
SMU1	0.0080299	1
PIAS2	0.0501561	1
MAP3K5	0.0110745	1
BIK	-0.0012844	1
EXOC4	0.0345763	1
CEP19	-0.007264	1
TM2D1	0.0856855	1
MED29	-0.0336791	1
KNSTRN	0.0303707	1
DCLRE1C	-0.0707846	1
HDHD3	0.0177975	1
UNKL	-0.0432074	1
ING2	-0.0298542	1
FBXW5	-0.0477072	1
PTDSS1	0.0247337	1
RWDD4	-0.0271032	1
CHMP3	-0.0894924	1
AC010618.3	0.0656047	1
SDHA	-0.0510102	1
TDRD7	-0.0187006	1

DPY19L3	0.0630958	1
UBE2J2	-0.0781734	1
EEF1AKMT2	0.0196208	1
HHEX	0.082524	1
XPO4	-0.0235374	1
AL442125.1	-0.0347934	1
AMMECR1L	-0.0643409	1
RCC1	0.0356958	1
LACC1	-0.0236906	1
CLUAP1	0.0046599	1
ZDHHC23	-0.0480549	1
SPARC	0.1297031	1
E2F6	0.0279185	1
WDYHV1	0.0010244	1
COX8A	-0.0499086	1
CTSB	-0.0590655	1
AL022311.1	0.0096903	1
UBA7	-0.0717483	1
FUCA1	0.0490083	1
IKBIP	0.085366	1
PPM1M	-0.0192506	1
MBNL1	-0.1209255	1
KIAA1522	-0.0776771	1
FBXL5	-0.0338528	1
SFR1	0.0515074	1
CSNK1G2	0.0043078	1
POT1	0.0453928	1
SLC25A33	-0.0256033	1
SGSM2	-0.0729425	1
TBCC	0.1250237	1
TYMP	-0.0462411	1
MIR222HG	-0.1309957	1
SIRT2	-0.106038	1
TLR1	0.0354325	1
EIF3J	0.0584113	1
SPINK2	0.173243	1
FEZ2	-0.011535	1
TGFBR2	-0.0894514	1
MUS81	0.0082332	1
INTS7	0.0383577	1
LINC01473	0.0226206	1
MIEF1	0.0597381	1
TGDS	0.0111911	1
CCNA1	0.0236652	1
VPS52	-0.0014076	1
ZNF571	0.0033299	1
SECISBP2L	-0.1590857	1
USE1	0.0727956	1
SDF4	-0.0265212	1
SMIM4	0.0524213	1

TOMM40L	-0.057077	1
SPIN2B	0.0272008	1
SEC31A	0.0236315	1
ABL2	-0.0671089	1
NDUFS8	0.015597	1
GNL3	0.1192126	1
SLC25A36	-0.0898532	1
AC004540.2	0.1380124	1
GPKOW	-0.0469567	1
AC016735.1	0.0563486	1
KIF27	0.0361024	1
AKIP1	0.0404313	1
ERMARD	0.0192288	1
AC009403.1	0.0739896	1
CYREN	-0.0465291	1
HMGB3	-0.1356276	1
RALA	0.0013835	1
NIPAL3	-0.0054463	1
AC078846.1	0.0201267	1
DIABLO	0.044724	1
TNFAIP8L2	0.0257689	1
ACTR3B	0.0483737	1
CSF3R	0.1527357	1
TTLL5	-0.0018787	1
TMED9	-0.0623167	1
C1RL-AS1	0.0109173	1
BST2	0.1172002	1
YEATS2	0.0181985	1
DUSP7	-0.0677587	1
ZNF217	0.0806487	1
MIR99AHG	0.0851638	1
TBCE	-0.0086887	1
LETM1	0.045629	1
ASCC3	0.1198341	1
NUP205	0.0285115	1
SRSF2	-0.1307051	1
LRCH3	-0.0609587	1
TNFRSF10A	-0.04995	1
SP4	-0.018749	1
WDR45	-0.0271538	1
BPNT1	0.0293256	1
TIMM44	0.0391755	1
YIPF2	-0.0115134	1
SIDT2	-0.0042706	1
PPP1R14B	0.0169887	1
ECHDC2	0.0178456	1
SLC15A2	0.0264893	1
RERE	-0.0682401	1
ITGAE	-0.0216823	1
WDR35	-0.0162674	1

ZNF280C	-0.0017338	1
RUBCN	-0.0295182	1
MCU	0.0261527	1
ARL14EP	0.0857023	1
ARL17B	0.0280675	1
ZNF583	-0.021101	1
WDR83	0.045532	1
GIHCG	0.1515988	1
TRDMT1	-0.0413295	1
APOL6	-0.0932923	1
CLPTM1	-0.0115039	1
OTUB1	-0.0195546	1
HMBOX1	0.0185539	1
RAB1B	-0.0455947	1
FBXL20	-0.0207489	1
ZNF564	0.0010289	1
IDNK	0.0813004	1
LDB1	0.042737	1
TBCE.1	-0.0406882	1
FEM1B	-0.0759081	1
CNN2	-0.1281864	1
RANBP6	0.0892563	1
CC2D1A	0.0042106	1
AGBL5	-0.0477024	1
BMT2	-0.010064	1
PRRC1	-0.0518223	1
ACACA	-0.0280592	1
PCTP	0.022566	1
RAVER1	0.0019193	1
ETV3	-0.0789333	1
BAZ1A	-0.0360284	1
TARBP2	0.0057804	1
TRMT1	-0.0009735	1
KHDC1	0.0361361	1
RMC1	-0.0608575	1
OS9	-0.0089349	1
NONO	0.025349	1
ANAPC15	-0.0588954	1
UGGT2	0.0442645	1
AL133453.1	0.0295678	1
SVIP	0.115539	1
TADA1	0.0042757	1
TTYH2	0.0381049	1
SMYD5	0.0282887	1
ATP6V1G1	-0.0787306	1
CNOT4	0.0111221	1
MFGE8	0.043553	1
AKAP10	-0.0040521	1
TMEM50A	-0.1092494	1
RNPEPL1	-0.0300274	1

PPIL2	-0.0272447	1
KHSRP	0.0727212	1
CDK2AP1	0.0872595	1
HIP1	-0.0396176	1
GORASP1	-0.0190766	1
UTP11	-0.1102	1
NUP35	0.0288994	1
C14orf119	-0.0696255	1
ZNF226	0.0323781	1
ZNF699	-0.0376304	1
SPTBN1	0.048175	1
DCAF5	-0.0122329	1
DEXI	-0.0345481	1
SLC25A44	-0.0459482	1
MTA3	-0.0199654	1
RPN1	0.0101287	1
FOXK2	-0.0299292	1
ZNF257	0.0763125	1
USP25	0.0374151	1
COPS6	0.0147736	1
NUP43	0.037926	1
MED16	0.0037286	1
FTL	-0.1274571	1
NGLY1	0.0367252	1
STAR	0.0358837	1
SLC35A3	0.0015024	1
A1BG-AS1	-0.0144751	1
BBIP1	-0.0192506	1
GMPS	0.0971811	1
MUL1	-0.0694151	1
HOXB-AS3	0.0092257	1
NOC4L	7.941E-05	1
ZNF254	-0.0342991	1
CCDC93	-0.0397749	1
THRA	0.06028	1
AL591623.1	-0.0291212	1
MTA1	-0.0419218	1
CYP20A1	0.0218118	1
FAM122A	0.014103	1
EIF1AX	0.1049554	1
CNNM4	0.0129468	1
CHURC1	0.0833453	1
ARID1A	0.0009971	1
TNFAIP1	-0.0928313	1
NIPA1	-0.0790149	1
FBXO38	-0.0739283	1
IKBKE	-0.0419625	1
ATG10	0.0745762	1
ZNF790-AS1	0.0665256	1
MTMR1	-0.0133868	1

MAP3K3	-0.0486687	1
PHYKPL	-0.1085351	1
AMFR	-0.0488602	1
ZNF746	-0.0193407	1
RAB40C	0.0243947	1
TRAPPC8	0.0106539	1
FUZ	-0.0208893	1
ISCA2	-0.0412185	1
RDH11	0.0032815	1
TAF2	0.0396391	1
KLHL24	0.0473393	1
GPATCH8	-0.068838	1
UBR3	0.0457052	1
CARHSP1	-0.0573185	1
DDX28	0.0098148	1
CAB39	-0.0912627	1
MEAF6	0.0265064	1
TFB1M	0.0468448	1
PRKCSH	-0.0239377	1
AC093827.4	0.01607	1
ZNF845	0.0266781	1
LRR1	0.0129688	1
ZNF678	0.048385	1
PRDM2	-0.1261091	1
TM2D3	-0.0998488	1
TMF1	0.057551	1
HAVCR2	-0.0711532	1
ANKRD46	0.055477	1
SETMAR	0.0554601	1
VPS26B	0.0456525	1
ASAP2	0.0253454	1
ODF2	-0.0016211	1
SMYD4	0.060291	1
ZNF33B	0.1028544	1
WASHC1	0.0395581	1
KDM4A	0.0242839	1
VAMP5	0.0716457	1
ZNF592	-0.0980583	1
GLA	0.0290323	1
PRDM16-DT	-0.0180165	1
NAA16	-0.0110709	1
NBEAL1	0.0317375	1
DCTN5	-0.0111121	1
CSTF1	0.0310663	1
MIR4453HG	-0.0255326	1
SEM1	-0.0812985	1
UBTD1	-0.0582135	1
DNAJC25	0.018181	1
LPP	0.0595597	1
SPCS2	0.0654011	1

CORO1B	0.0382416	1
MYL6B	0.0204587	1
PDPK1	-0.0708117	1
DNAJC3-DT	0.0642005	1
USP1	0.0380308	1
SMYD2	-0.0285322	1
ULK3	-0.0499279	1
ERAP2	0.0195798	1
LINC02591	-0.0026645	1
TAOK1	-0.022684	1
DEPDC5	0.0289256	1
CNTROB	-0.0118211	1
C1orf122	-0.0576988	1
SPAST	0.0180965	1
STAP1	-0.0364307	1
SETD4	-0.0422843	1
LPGAT1	-0.0055897	1
WRN	0.1186875	1
ZNF281	0.0309385	1
NBDY	-0.0584661	1
DVL3	0.0049141	1
ANKRD52	-0.0098564	1
KIN	0.0172434	1
GTF3C4	-0.0292574	1
TSC22D2	-0.0637316	1
RPE	0.0709765	1
TECPR1	-0.0124453	1
NSUN5	0.0165699	1
PSMC3	-0.0425946	1
LBH	0.0635194	1
DERL3	-0.0315644	1
PPP1R26	0.0419098	1
KIAA0556	-0.0286821	1
CD244	0.0412763	1
ZNF445	0.1110379	1
TMEM161B	0.0320376	1
CNPPD1	-0.0609725	1
NEDD1	-0.0192507	1
SNX29	0.0308317	1
USP39	0.0109127	1
SRP68	-0.0512148	1
WDR77	-0.0012664	1
DNAJC14	-0.0074828	1
VCP	-0.163585	1
ISG20L2	0.0462596	1
SLC38A9	0.0355376	1
HOOK2	0.0519715	1
ZNF266	-0.0327333	1
CDYL2	-0.0275241	1
FUBP3	0.0249796	1

OSGEP	-0.0308056	1
BTN2A1	-0.0568754	1
CD33	0.0907917	1
UBE3B	-0.0254893	1
DDX41	0.0475286	1
G6PC3	-0.0521309	1
RAD1	-0.0490374	1
TPRA1	0.0248525	1
ZNF621	0.0256619	1
ATP6V1E1	-0.0618878	1
PWP1	0.0347728	1
PFKP	0.0432432	1
CHMP7	-0.0134786	1
SCAMP4	-0.0419933	1
CUL9	-0.0079291	1
LENG9	0.0473402	1
TAX1BP1	-0.1016136	1
PDIA6	0.0309456	1
ATG2A	0.0265436	1
STAG2	0.0038171	1
PNPLA2	-0.0587717	1
BNIP1	-0.0761735	1
EXOSC1	-0.0195125	1
LINC00847	-0.0045292	1
MIR1915HG	0.031895	1
BCL2L1	0.0196967	1
MDM2	0.0573952	1
GSTM4	0.0450238	1
STMN3	-0.0744391	1
MRFAP1L1	0.0616457	1
AKR1C3	0.1230973	1
FBXL19	0.0121684	1
LETM2	-0.0901745	1
NSUN4	0.0164036	1
ANKMY1	0.0050584	1
EIF2S2	-0.1710762	1
LEPR	0.0573806	1
WDR6	-0.0346147	1
NUBP2	0.0474908	1
PMPCB	0.0961083	1
ARHGEF40	-0.0707787	1
IFRD1	-0.0389127	1
POLD3	-0.0008513	1
ZNF717	0.0602712	1
ZNF408	-0.0435203	1
MRPL13	0.0086133	1
NRG4	0.046224	1
TET3	0.0209156	1
POM121	-0.0156075	1
TCTN1	-0.0470838	1

SULT1C4	0.0462198	1
DNMT3A	-0.0299417	1
TRAF3IP2	-0.0544287	1
ZNF207	-0.0975691	1
BATF	0.0272248	1
MZB1	0.1455206	1
HIC2	0.0362131	1
A2M-AS1	0.0616937	1
RAB3IP	-0.0295608	1
GFER	0.0421656	1
VPS4B	-0.0197074	1
MIR29B2CH	-0.0219048	1
DSN1	0.0273079	1
UAP1	-0.0442239	1
IDH3B	-0.018934	1
RGPD5	-0.0483727	1
CHSY1	0.0091035	1
DCAF11	0.0115157	1
SLC35D2	-0.0134388	1
WDR11	-0.0263608	1
DLEU2	-0.062306	1
YARS2	-0.02791	1
AC244090.1	0.0233074	1
PPP2R2D	-0.0033921	1
FGFR1OP	0.0136248	1
XRCC1	0.0011631	1
ATG16L2	-0.0015485	1
HOXA5	0.0078401	1
NDUFA11	0.0654372	1
MSL1	0.0796789	1
SERINC5	0.0005749	1
ARHGAP12	0.0227078	1
MED23	-0.06125	1
B3GNT5	-0.0552927	1
TADA3	0.0584046	1
ZNF182	0.0313561	1
CCPG1	0.0732734	1
ETS2	-0.1394813	1
WT1	0.0624617	1
ADCY3	0.0273138	1
HDAC4	0.0150597	1
CDC37L1	0.0275393	1
SUOX	0.0372008	1
DROSHA	0.036908	1
WDR41	0.0046915	1
BRAP	-0.1051485	1
UQCC3	0.1106584	1
VIPAS39	-0.0176631	1
BORCS8	-0.0208135	1
PTGES2	0.0213317	1

SRGAP2C	0.0318645	1
CCDC84	0.0103181	1
PRR5	0.0098345	1
KMT5A	0.0121012	1
C1orf109	0.0402959	1
METAP1	0.0725588	1
ANKRD10	0.0403065	1
VAT1	-0.0918465	1
RBM23	0.0005178	1
MRPS18B	0.0831963	1
KDM3A	-0.1028794	1
AC092164.1	0.0181131	1
LGALS9	-0.0206108	1
PSMD14	-0.0807235	1
NDUFB9	-0.1667847	1
DYNLL2	-0.040053	1
BTRC	0.0152161	1
PARP10	0.0136891	1
ATOX1	-0.079455	1
FAM76B	0.0524246	1
NAGK	-0.0386614	1
CHCHD5	-0.0275167	1
COX19	-0.0653712	1
AP4B1	-0.0278504	1
NIP7	0.0068393	1
ACY1	0.0197081	1
NT5DC2	0.033985	1
TRIM21	-0.0347492	1
ZNF740	0.0494982	1
ZBTB41	0.0480753	1
NSMCE1-DT	0.0485968	1
NF1	0.0268872	1
CBX2	0.0306314	1
PNRC2	-0.0254401	1
KLHL6	-0.0155704	1
CCT4	0.0328981	1
TIMM21	0.0492054	1
SLC25A38	0.0385298	1
KANK2	-0.0863183	1
TPMT	0.0341478	1
GRB10	-0.0855361	1
TCF7	0.0095533	1
ZC3H7A	0.004116	1
GIGYF1	-0.0033481	1
AC009831.3	-0.0287081	1
NDUFAF5	-0.0582835	1
FAM41C	-0.0250482	1
GNPNAT1	-0.1178629	1
USP19	0.0440443	1
SETDB1	-0.0361505	1

HNRNPH3	0.0378513	1
SEL1L3	-0.0517686	1
POLDIP2	0.0136256	1
CCAR2	-0.035047	1
TBRG1	-0.0541858	1
ARRDC1	-0.0430374	1
NUMBL	-0.0307675	1
SF3A1	-0.0336975	1
FRS2	-0.0306239	1
CYP2R1	0.0018355	1
AXIN1	-0.0335669	1
CTDSP2	-0.0730394	1
HELLS	0.0010997	1
BNIP2	-0.1526546	1
FLII	-0.0531793	1
SEPSECS	0.0273079	1
HSPA13	-0.1088431	1
UEVLD	0.0215171	1
AAK1	0.0083133	1
CDK5RAP1	0.0012612	1
GALT	0.0284616	1
MAP3K7	-0.0202745	1
AP3M2	-0.0108838	1
ATF6	-0.0925154	1
INTS12	-0.0158346	1
CLHC1	0.0470851	1
ZNF614	0.0169234	1
RNF216	0.0684226	1
PEX19	0.0073166	1
KATNAL1	-0.015041	1
SYMPK	0.025553	1
KLHL11	0.0472096	1
TMEM9B	0.0189601	1
MAIP1	-0.0347276	1
METTL21A	-0.0116684	1
JMJD7	-0.0270701	1
NAGA	0.0297374	1
LSG1	0.0312754	1
REPS1	0.0059412	1
HNRNPDL	-0.1527459	1
ANKRD44	-0.0139606	1
MRPL46	-0.0235351	1
CAMSAP1	0.0029072	1
APLF	0.0269909	1
NUBP1	-0.0684384	1
BTBD8	0.0583733	1
ERO1B	0.0165256	1
ABCC1	0.0025492	1
PHAX	-0.0170706	1
GVQW3	-0.0270997	1

HPRT1	0.0133509	1
CSNK2A2	-0.0074112	1
TSPOAP1-A	-0.0033406	1
GPATCH1	0.0773559	1
METTL22	-0.0304544	1
SNRPF	0.1332006	1
CLCN6	-0.0587918	1
SCML2	-0.0240727	1
USP5	0.0330746	1
UBE4A	-0.0002848	1
CLN3	-0.002977	1
DNAJB12	-0.1075926	1
FBXO42	-0.036251	1
ZNF212	-0.0428477	1
APOL2	0.0315471	1
FRMD4A	0.0003559	1
BCLAF3	-0.0315625	1
PCM1	0.0376112	1
MT2A	-0.1719231	1
COP1	-0.0157664	1
SLC25A26	0.055024	1
CDIP1	-0.0029721	1
TUT1	0.0124796	1
ANKRD49	0.0957616	1
VBP1	0.0578428	1
CXorf56	-0.005964	1
TMEM91	-0.0907811	1
KLHL12	-0.0146902	1
SART3	-0.0490451	1
EIF1B	-0.1541244	1
SPOPL	-0.0620059	1
AL080276.2	-0.0100947	1
PPAT	0.0797366	1
FAM193A	-0.0355435	1
AREL1	-0.0637776	1
DYRK2	-0.0256916	1
AC016065.1	0.0079839	1
LIMK2	-0.0499724	1
DCAF7	0.0610516	1
CAPN15	-0.0063941	1
SNAPIN	0.0281949	1
CNOT6	0.03975	1
C9orf72	-0.0070122	1
HSD17B11	0.0676342	1
TYMSOS	0.032397	1
NDUFV1	-0.0548153	1
PIGB	0.0134307	1
AC097376.3	0.058837	1
MBIP	-0.0355263	1
PROSER1	-0.0157126	1

RETSAT	0.0143182	1
SMARCB1	-0.0315915	1
SMARCD1	-0.0012404	1
ELK3	-0.0961872	1
TMEM185B	0.0562409	1
MIOS	-0.0420431	1
AL035530.2	0.0540814	1
ZNF444	0.0245212	1
TAF10	-0.0197436	1
PHC2	-0.0580475	1
FAM192A	0.1433378	1
NUDT18	-0.046889	1
DHRS12	-0.0225347	1
ZCCHC4	0.0126413	1
PHTF2	-0.10908	1
AGO3	-0.0569189	1
MAD2L2	0.0547972	1
RSPH3	-0.0097379	1
RINT1	0.0023865	1
TAF6	-0.0125869	1
MED7	0.0493113	1
CEP135	-0.0101542	1
ZNF75D	0.0257999	1
NUDT1	-0.075866	1
GTF3C2	0.0077356	1
TIPRL	0.0077561	1
ANKRD36B	-0.0264496	1
CMC2	-0.085781	1
ESCO1	-0.0919591	1
WDCP	0.0384529	1
RNF123	0.0377246	1
CDK10	-0.0043691	1
CNOT8	-0.0933663	1
CHST11	-0.1099055	1
ITPA	0.1190059	1
PLSCR3	0.0017348	1
PHF3	0.0043265	1
POLR2K	-0.0244798	1
FTSJ1	0.0238731	1
ATP5MD	0.0447859	1
GGT5	0.0322648	1
TMEM268	0.0051339	1
ELAVL1	-0.0260301	1
TMEM222	-0.0314595	1
GCC2	-0.0725878	1
RHBDD3	0.0116368	1
AC017083.1	0.0257871	1
ZNF436-AS1	-0.028498	1
GNPAT	-0.0524822	1
PEPD	0.0537414	1

MAP2K5	0.0290841	1
LINC02328	0.0094678	1
NCOR1	0.0345885	1
ZNF526	-0.0311112	1
POLR3C	0.0384302	1
WAS	0.0384024	1
SLC9A6	-0.0269284	1
SNAPC3	0.0368161	1
GTF3C6	-0.0857218	1
BOLA2	-0.0245797	1
HMGN4	-0.0133563	1
CDK7	-0.0452575	1
MTF2	-0.061841	1
AC004854.2	0.0444888	1
CMC4	-0.0254668	1
SNRPC	-0.0700748	1
DENND10	0.0172647	1
USP49	0.0179304	1
STX6	-0.0331732	1
SNRPB2	0.0280587	1
PYROXD2	0.0272642	1
NTAN1	-0.0434878	1
SC5D	-0.0150607	1
REPS2	0.0284377	1
ZMAT1	0.0100052	1
POLR2L	-0.0569832	1
RAB3GAP1	-0.0094136	1
PEX2	-0.0743606	1
DENND1B	-0.010558	1
THUMPD3	-0.0585453	1
ZNF865	-0.0061178	1
ADGRG1	-0.1242383	1
PIAS3	0.0098667	1
SNX5	0.0154184	1
UBL3	-0.046426	1
TOR1AIP2	-0.0808937	1
NBN	0.0911413	1
CLIP4	0.057015	1
TTC38	0.0060393	1
ZRANB3	0.0324675	1
LANCL2	-0.0500689	1
NR2C2AP	0.0550793	1
TPD52L2	-0.0441839	1
EXT2	0.0317404	1
KEAP1	-0.0368589	1
ZFYVE19	-0.0196647	1
CERS6	0.0528454	1
NPAT	0.0300245	1
SSBP1	0.123087	1
MCUR1	0.0333103	1

MYO5A	0.0062623	1
OSBPL7	0.0088389	1
IPMK	-0.0630058	1
KCNK5	-0.0305871	1
NIPSNAP2	0.0294429	1
RDH5	0.0315793	1
KLC4	0.002461	1
LBR	-0.1087729	1
MYOZ3	0.040285	1
POM121C	-0.0148896	1
HHLA3	0.0370744	1
TMEM187	0.0188785	1
FAM210A	-0.0302306	1
TWF1	0.0240912	1
CDK13	0.0258952	1
GOT1	-0.019794	1
SUGP1	0.049561	1
BRPF1	-0.0705629	1
PGAP2	0.0502209	1
METTL14	-0.0134727	1
GIT1	-0.0234475	1
PLPP6	0.0179573	1
CRKL	-0.0453823	1
TXNL4B	0.001582	1
CENPU	0.0045915	1
PHB2	-0.017469	1
ITGA6-AS1	0.0462515	1
WARS2	-0.0149188	1
GIPC1	0.0309243	1
TTC21B	0.0217832	1
TRIM58	-0.0638219	1
UBE2O	0.0368864	1
AC018362.1	0.0297608	1
HSDL1	0.0128432	1
TRAPPC13	-0.044132	1
AC092821.3	0.0307877	1
AKNA	-0.114539	1
NME7	0.0783871	1
UMAD1	0.0434731	1
OGDH	0.0073294	1
PACS2	-0.0195778	1
PDHB	0.0079629	1
SAV1	0.0255908	1
HK1	-0.0706936	1
RANBP9	-0.0803848	1
VPS51	-0.0289058	1
PTAFR	0.0268542	1
CLPX	0.0654257	1
CAPN11	-0.0550533	1
ACADS	0.0231919	1

TRAK1	-0.0792139	1
PPP1R37	-0.0439615	1
BTG3	-0.0747428	1
UGCG	0.0109608	1
MTDH	-0.0307622	1
TUG1	0.0427194	1
RHPN1	-0.0731858	1
BRWD1	-0.0458954	1
MEGF6	0.0490334	1
NSUN3	0.0556204	1
HIST1H3H	-0.0775372	1
ACAT2	0.0235437	1
CTNNBIP1	0.0039589	1
INTS9	0.0337907	1
HNRNPU	-0.1317252	1
SGMS1-AS1	0.0383644	1
LINC01137	0.0293242	1
FOXP1	-0.1017529	1
FAM102A	-0.0018564	1
RNF146	0.0366561	1
C1orf50	-0.1009687	1
SNX8	-0.0494352	1
KCTD2	-0.0542568	1
TLE4	-0.114114	1
CHIC1	0.0133895	1
RNF26	0.0107433	1
NDUFS5	-0.0564987	1
CHAF1A	-0.0362949	1
PHGDH	-0.0368809	1
ARF5	-0.0495298	1
BARD1	0.0160789	1
PHF11	0.0681269	1
STARD4	-0.040137	1
GIT2	0.0318563	1
SLC12A6	-0.0098286	1
NKAPD1	-0.0906417	1
KRT8	0.0717124	1
TRIM71	0.0424238	1
AL121944.1	-0.0759847	1
NUDT2	0.0352763	1
AC008443.5	0.0667712	1
TMED7	-0.0259587	1
DCUN1D1	-0.005957	1
ARSK	0.025561	1
GDAP2	-0.0099133	1
TAF6L	0.0096606	1
REC8	-0.0352994	1
IGSF8	-0.001596	1
AL049840.5	-0.0662268	1
NR1H3	0.0247191	1

DNAJC17	0.0138587	1
GRAMD4	-0.0335191	1
GRB2	-0.004205	1
TIGD5	0.0164924	1
DLC1	0.0345048	1
UNC13D	-0.0539932	1
SNX27	0.0398231	1
CYC1	0.0709975	1
DGKE	0.0549512	1
TRAF7	0.0088109	1
ZNF567	0.0528136	1
C5orf30	-0.0364369	1
CIP2A	0.0216258	1
VPS18	-0.0520971	1
COMMD5	-0.0183837	1
LMBRD1	-0.00872	1
TTLL3	0.0028105	1
UBAP2	0.0329316	1
TRAM2	0.0095333	1
SP140L	0.0352526	1
SCYL1	-0.0498603	1
DOT1L	-0.0936281	1
YIPF1	0.0296307	1
ZC3HC1	-0.0407174	1
AC016394.1	0.0316889	1
SNX15	-0.0081048	1
AL135925.1	0.0617057	1
GABPA	0.0244582	1
BRF2	-0.0136559	1
TRAPPC11	0.0973831	1
TAF1D	-0.1439499	1
AC104162.2	-0.0295461	1
ZFYVE21	-0.026339	1
ADGRA2	0.044537	1
FAM107B	-0.1246024	1
RRP7A	0.0168496	1
MADD	-0.0249897	1
VPS16	0.007581	1
ZNF432	-0.0014181	1
OSBPL8	0.0207162	1
DCUN1D5	-0.0031229	1
DDB1	0.0406636	1
AP2B1	0.0465798	1
GEMIN2	0.0241203	1
CRTC1	-0.0160149	1
CTPS2	0.007255	1
DRAM1	-0.063975	1
PDZD8	-0.0349218	1
PTRHD1	0.0787806	1
TACO1	-0.0422718	1

ABO	0.0502245	1
LRRC58	0.0566853	1
ZNF771	-0.0041339	1
NANP	0.0251379	1
PXMP2	0.0231654	1
UQCC1	0.0623299	1
F8A3	0.0538037	1
ANAPC2	0.0239624	1
CYP51A1	-0.0110288	1
CCDC71	-0.0533824	1
RIPK3	-0.0512691	1
ITPK1	0.0006723	1
UBE2E3	-0.0211583	1
FIZ1	-0.0055594	1
C18orf21	-0.0627957	1
IGF2BP2	-0.0500741	1
SPRTN	0.0423649	1
SRSF9	0.0323687	1
RFK	-0.0229909	1
AC092069.1	-0.0288209	1
PAPOLG	-0.0498244	1
AHCTF1	-0.0012802	1
TCF3	0.073543	1
C2orf49	0.0111959	1
INTS14	0.0018127	1
TIMM22	-0.0727194	1
CLTA	-0.0727034	1
EID2B	0.0290268	1
TOGARAM1	-0.0927095	1
NEPRO	0.0105287	1
DHDDS	-0.0086249	1
ZFHX3	-0.0174767	1
NSMCE2	0.0468359	1
MRPL38	0.0989407	1
PSMG3-AS1	0.0300774	1
BRMS1	-0.0326118	1
FRAT1	0.0446156	1
C17orf75	0.0235343	1
ZNF267	-0.0537726	1
RMND5B	-0.034608	1
BEX3	0.0335682	1
SCO1	0.0464175	1
STRADA	-0.0359373	1
CHP1	-0.0976926	1
SHLD3	0.0355666	1
ELP5	-0.0971063	1
ZFYVE1	-0.0277656	1
CELF2	-0.0767649	1
TBCEL	0.0239371	1
MXD1	-0.087789	1

AC007038.2	0.0332763	1
CCDC28A	-0.0273953	1
FANCM	0.0579064	1
LINC00893	-0.0125096	1
SIKE1	0.0010486	1
UBXN11	-0.028995	1
HINT1	0.0275685	1
C20orf96	0.018101	1
DNAJA4	-0.0089558	1
RABGGTA	0.0188083	1
DECR1	0.006159	1
ELAC1	0.0267055	1
TLE3	-0.0897127	1
PDXDC1	0.0026162	1
YIF1B	-0.0315513	1
DAZAP2	0.0321493	1
PHF20L1	-0.063667	1
ESYT2	-0.1600538	1
EIF3A	0.1479703	1
ZNF510	0.0402237	1
SPTLC1	0.060235	1
DCAF8	0.0663647	1
DENND5A	-0.0669103	1
TMEM216	-0.0609087	1
RECQL5	0.0325589	1
LEO1	0.0360421	1
UAP1L1	0.0105019	1
EXOSC7	0.0709829	1
GPATCH2L	0.0645382	1
SAMSN1	0.0612808	1
ATP13A3	-0.0819398	1
RBSN	-0.0205338	1
RPUSD2	-0.0391111	1
HOXA7	0.0621387	1
FAM193B	-0.011797	1
FAM199X	-0.0534497	1
GYPC	0.049639	1
PSMD13	-0.0151001	1
ABHD5	-0.015256	1
PCCB	0.0153439	1
ANKRD12	-0.0985718	1
MICU3	0.0036884	1
GAA	-0.0151315	1
NAT9	-0.0563684	1
ST3GAL2	-0.0709362	1
ANKMY2	-0.0406506	1
NDUFB4	-0.006734	1
POLDIP3	-0.0895653	1
AATF	0.065606	1
RUSC2	-0.0298642	1

IL17RA	0.0191555	1
AL645568.1	0.0349494	1
SLC35F2	0.0299165	1
HLA-DPB1	0.0875575	1
WWOX	0.0148008	1
SEC61A1	-0.0547701	1
ZNF230	0.0260753	1
NIPA2	-0.0567691	1
MED22	-0.0261806	1
ZNF419	-0.0001036	1
ARL6IP4	-0.0765554	1
MT1F	-0.012187	1
ABITRAM	0.0142723	1
C11orf21	0.0146337	1
PRCC	-0.0627804	1
ACPP	0.0030252	1
MICALL1	-0.037853	1
DANCR	0.0608967	1
TOM1L2	-0.0497958	1
ZNF75A	0.0082973	1
MMGT1	0.0685878	1
RBAK	0.0122218	1
GGCX	-0.0124835	1
TIMM50	0.004271	1
PSMD3	0.0441066	1
AP1B1	-0.001162	1
MEIS1-AS2	-0.0690906	1
SPDL1	0.0781382	1
APBA3	0.0143994	1
EXOSC10	0.0362397	1
ABCB10	0.0414171	1
CEP120	0.0257691	1
USP37	-0.0333047	1
BBS2	0.0103014	1
RSBN1	0.0686172	1
ANKRA2	-0.0005785	1
TMEM214	0.007705	1
BORCS6	-0.0386873	1
WDR19	0.0066978	1
SNHG32	0.1572756	1
CACTIN	-0.0062765	1
SOCS6	-0.0076659	1
CARMIL2	0.0054893	1
LTB4R	-0.0352194	1
PRUNE1	-0.0013259	1
CANT1	-0.0352843	1
GPS1	-4.33E-05	1
AC008124.1	-0.0420806	1
HERC6	0.0396033	1
TTC5	0.1116186	1

ZMAT2	0.0122812	1
MAP2K1	0.0100435	1
SERINC3	0.0199221	1
GTF3C1	0.0417076	1
PIK3C2B	0.0051564	1
NELFA	-0.0171517	1
ITPRIPL2	-0.0555444	1
IL15RA	-0.0197372	1
THEM4	0.0346501	1
COMTD1	-0.046922	1
TIPARP-AS1	0.0311784	1
IMP4	0.0303621	1
RPL15	-0.0616874	1
RFWD3	0.0100717	1
E2F3	-0.0587317	1
UBXN6	-0.0527638	1
NIPSNAP1	0.0504996	1
DDX11	0.029373	1
AC007566.1	0.0504568	1
ITGAV	0.0128824	1
WRAP73	0.0385071	1
ITGB2-AS1	-0.0703695	1
METTL6	0.0266676	1
TMEM39B	0.0264427	1
SERPING1	0.0395141	1
MARK2	-0.0610132	1
ALG12	0.0083913	1
BTK	0.1431396	1
TUBG1	-0.0113833	1
CIAO3	-0.0115684	1
POLR2J2	0.0278358	1
KCTD6	-0.0431145	1
ARRDC1-AS	-0.0215033	1
TBC1D24	0.0344091	1
ATPAF2	-0.0066844	1
ERN1	-0.0634171	1
TP53I3	-0.0557807	1
HARS2	-0.0493201	1
GBGT1	0.0373417	1
CLPP	0.0616276	1
PTRH1	-0.0020993	1
AC004034.1	0.031282	1
RER1	0.0378543	1
ATL3	0.0270126	1
GTF2F2	0.0354129	1
TRIM61	0.0330103	1
MDM4	0.024268	1
KRBA2	0.0921232	1
C9orf16	0.0822514	1
DLG4	-0.0367465	1

EED	-0.0217001	1
LMAN2L	0.0305441	1
NUS1	-0.0420017	1
GTF2F1	-0.0394528	1
CAPZA1	-0.0550074	1
MCM7	0.0270947	1
SLC25A24	0.0117261	1
MTFMT	0.0454161	1
AFF3	-0.0086504	1
MYCNOS	0.035774	1
APPL1	-0.0138305	1
PDSS2	0.0546474	1
AAAS	0.0303297	1
GOLPH3L	-0.0305311	1
PSMB10	-0.0531514	1
AC245297.3	-0.0533959	1
GNA12	0.0417333	1
DIS3L2	0.0088343	1
HYMAI	0.0262979	1
HERC1	-0.1021914	1
CCDC186	-0.1004043	1
LIN54	-0.0283453	1
AP4E1	-0.0081112	1
ZNF816	-0.0079113	1
ZSCAN2	-0.0433325	1
HIST1H4E	0.0930807	1
PARP6	-0.0588313	1
PTPMT1	0.0552548	1
GPAA1	-0.0233407	1
CATSPERG	0.0150163	1
RUFY3	0.0074896	1
ZSCAN29	0.0529895	1
AACS	0.0110522	1
OSBPL5	0.0384753	1
AZIN1-AS1	0.0347235	1
EIF3K	0.0482113	1
RAMAC	-0.0400815	1
SELL	0.0711292	1
NSL1	0.0776824	1
LSM12	-0.0615974	1
ANKS3	0.0426343	1
IKBKG	-0.0531998	1
FOXP1-IT1	0.0353796	1
FUBP1	0.0566893	1
DHX35	0.0043716	1
PDLIM5	-0.0232915	1
C11orf58	-0.0508034	1
TRAPPC9	0.0056474	1
FAM24B	-0.002163	1
SLC25A14	-0.0334293	1

YDJC	0.0740246	1
TMEM120A	0.0111344	1
PYGO2	-0.0196992	1
ARPC3	0.0155017	1
LINC02019	-0.0443722	1
NFU1	0.0183372	1
SEC16A	0.0022961	1
NMNAT1	0.0434903	1
MAPK6	-0.0396771	1
PIGX	0.0124218	1
DCP1B	0.0079137	1
ZNF100	0.057534	1
CIAO2B	0.0279961	1
POLA1	0.0530212	1
IFT88	-0.0365535	1
ERGIC3	0.0064793	1
CBWD5	0.0223312	1
STX18	-0.0128892	1
AC018647.2	0.0200777	1
MCM6	0.0541518	1
RAPGEF1	0.023252	1
FBXL6	-0.0258456	1
B9D2	-0.1160615	1
CHERP	0.0223556	1
CAMTA2	0.0101115	1
POLD1	0.0347499	1
TTC4	0.0158992	1
NSDHL	-0.0363074	1
CDC23	0.0378096	1
SLC25A37	0.0873086	1
PSMD2	0.1106925	1
ZNF17	0.0043073	1
ZNF726	-0.017632	1
WDR5	0.0168489	1
HSPBP1	-0.0191617	1
ABCF1	-0.0477641	1
PAPSS1	0.0657876	1
ZNF484	0.0176544	1
PTDSS2	0.0158466	1
ADAP1	-0.0764949	1
PTBP1	-0.0530702	1
CHD3	0.0625129	1
AC078883.1	0.0441848	1
NPM2	-0.011927	1
MICU2	0.0057179	1
PVR	0.0134694	1
TJAP1	-0.0203837	1
PREP	0.0532025	1
BOLA2B	0.0274163	1
TMEM94	0.0596923	1

BUD23	-0.0147396	1
NOMO3	-0.0101429	1
PTCD3	0.1067187	1
ALKBH5	0.0577714	1
COG7	0.0146821	1
SHLD1	-0.076522	1
IL10RB	-0.0238901	1
BSCL2	-0.0118412	1
FUOM	-0.1121326	1
TMEM186	0.0461069	1
CIAO1	0.1266238	1
MAPKAPK3	-0.0413599	1
YTHDF2	-0.0128827	1
GATD1	0.0160635	1
LPCAT4	0.0009493	1
MLKL	0.0114325	1
SUFU	0.002918	1
DEF8	-0.0571855	1
UBE2E2	0.0674086	1
ZC3H13	0.0651649	1
BRD9	-0.0217483	1
POC5	0.0926733	1
ZBTB40	0.0615646	1
ZNF687	-0.0383352	1
INTU	0.0297574	1
ZSCAN25	0.020267	1
USP46	-0.0129088	1
FTCDNL1	0.0622044	1
BTAF1	-0.0777581	1
ZNF131	0.0579008	1
ALKBH6	0.0012733	1
HGSNAT	0.0192775	1
GNB5	0.0558158	1
FAM168A	-0.0253873	1
KLC2	-0.0461653	1
AC145285.6	8.132E-05	1
EFNB1	-0.0859919	1
ZNF641	-0.0161561	1
PLEKHM2	-0.0505578	1
RAB10	-0.0035165	1
HECTD4	-0.0242167	1
NUCB1	-0.1024695	1
TXNL1	-0.0283792	1
NDUFC1	0.0251076	1
NVL	0.0027304	1
GBP2	-0.0373035	1
DELE1	-0.0039624	1
TRIP6	0.0185858	1
AC025171.3	-0.0269833	1
SMNDC1	0.0360704	1

TBC1D31	0.0289163	1
ZNF836	-0.0422448	1
DHCR7	-0.0144346	1
LSM8	0.015497	1
SCN1B	0.0025078	1
HLA-DQA1	-0.02145	1
PPIP5K1	0.0272073	1
DICER1-AS1	0.0405257	1
ARIH2OS	-0.0072281	1
GPD2	0.0250061	1
CALCOCO1	-0.0303115	1
TAF8	-0.0329709	1
SCAF1	-0.0715924	1
ENC1	-0.0453752	1
MIB2	-0.007557	1
PROK2	0.0239382	1
NMD3	0.0071218	1
DAPP1	-0.0798576	1
PHOSPHO2	-0.0068772	1
TPGS2	-0.0124405	1
COX16	0.0743942	1
CPSF1	0.0105488	1
SPATA20	-0.0230307	1
EDF1	0.0208839	1
MED9	-0.0697053	1
NDUFAF7	0.0105453	1
LTO1	-0.0855426	1
WDR34	-0.0371436	1
NICN1	-0.0051549	1
ZNF142	0.0128974	1
ACO2	-0.025421	1
SFSWAP	-0.0351937	1
TPT1-AS1	0.0261904	1
RAD51D	0.0487253	1
AC015712.2	0.0138299	1
BECN1	0.0434267	1
KLHL28	-0.1012926	1
SNHG5	0.0678169	1
TMEM71	-0.0056024	1
CCNYL1	-0.0952241	1
TAB1	0.0144775	1
RPL8	-0.0992627	1
ZNF440	0.0176163	1
CTSD	0.0624179	1
SLC39A13	-0.0410119	1
SNU13	-0.0141868	1
ELOVL5	-0.0516144	1
C2orf76	0.0300362	1
CLOCK	0.0316871	1
ZNF335	-0.0420386	1

PSMA1	0.0116493	1
ATP2A3	-0.0258262	1
TSPYL2	0.0415058	1
AL133523.1	0.041183	1
TRIM11	-0.0656916	1
ATG2B	-0.0209063	1
AC068587.4	0.0859805	1
SLC12A9	-0.0332201	1
NUP50-DT	0.0272539	1
FRMD4B	-0.1308598	1
AMN	0.0062234	1
AL365295.1	0.0347264	1
SMARCE1	-0.0390825	1
PYCARD	0.0139707	1
GEMIN7	-0.0129786	1
RAB28	0.0173199	1
IER3IP1	0.0528506	1
AUP1	-0.0786712	1
ECT2	0.0164665	1
PHLPP1	-0.0002163	1
PDK3	-0.0036874	1
RAD23A	-0.0287723	1
SLC4A2	-0.0240729	1
PIH1D1	0.0335054	1
EIF3C	0.0253282	1
CFL2	0.0263002	1
AC040970.1	-0.0008295	1
GUSB	-0.0385219	1
AC141424.1	0.0367762	1
PPIA	0.0344959	1
NCK2	0.0517072	1
OTUD3	-0.0015568	1
TSPAN14	-0.0435978	1
KIAA1328	-0.0018091	1
PRC1	-0.0410945	1
KDELR1	0.0781255	1
TCEAL8	0.0159814	1
MERTK	0.0417084	1
BEND5	0.0430794	1
PUSL1	-0.0043046	1
MANBAL	-0.0727689	1
WSB2	-0.0261509	1
PPP2R1B	-0.0223964	1
AGO4	0.0234903	1
CDK5RAP3	0.0105109	1
PGGT1B	0.0445983	1
DCUN1D4	0.0043515	1
BANP	-0.017622	1
DGCR8	0.0010183	1
KMT2D	-0.0031416	1

AC083798.2	0.0184741	1
PHF12	-0.085737	1
MED20	-0.0001685	1
LRRC8C-DT	-0.0003658	1
ZNF768	-0.0200398	1
PTPN11	0.0556138	1
UVSSA	0.036463	1
CWF19L1	-0.0559507	1
NUTM2B-AS	0.0493753	1
KDM2B	-0.000191	1
AC008764.8	0.0366215	1
ZNF696	0.0151663	1
MKS1	0.0280033	1
VTI1B	0.0749386	1
PHC3	0.0100819	1
PNPLA6	-0.0772605	1
CRAT	0.0161128	1
CDKAL1	0.0403824	1
IL12RB1	-0.0415867	1
ZDHHC17	-0.0002397	1
RTL8A	0.0076768	1
IL17RC	-0.0222174	1
MIATNB	0.029282	1
RENBP	0.0024432	1
PRAF2	-0.048545	1
KIFAP3	-0.0346146	1
ZBTB11	-0.0231527	1
RNF121	0.0124389	1
IDH3G	0.0197891	1
COX7A2	0.025762	1
SEC22A	-0.0387834	1
DAPK3	-0.0414763	1
TNRC6C-AS	-0.0259664	1
AC018521.5	0.0227423	1
MAST2	-0.000169	1
NAPA-AS1	0.0012626	1
DYM	0.0752826	1
SEPSECS-A	0.0429769	1
TMEM129	-0.0151174	1
LRG1	0.0155416	1
NMRK1	0.034463	1
ZNF10	0.0014407	1
ABCB8	-0.0624433	1
MUTYH	0.0231461	1
TBCD	0.0396562	1
LRP12	0.0108268	1
DUS3L	-0.0104148	1
ARFGEF2	-0.1130945	1
MRPL20-AS	-0.0330964	1
CPLANE1	0.0422444	1

CSRP1	-0.0176743	1
URB2	0.022238	1
SEPTIN6	0.1147175	1
NUTM2A-AS	-0.0076591	1
GRHPR	0.0269469	1
ATP5IF1	-0.0036377	1
TCF20	0.0234227	1
SEC23B	-0.0036011	1
SPTY2D1	-0.0956769	1
LAMTOR5-A	0.0264506	1
PMAIP1	-0.0511442	1
SNX20	0.0657549	1
UBFD1	0.05534	1
SLF2	0.0323478	1
ZNF12	-0.0151476	1
USP20	0.0171577	1
GPT2	-0.064341	1
ADAT1	0.0106262	1
SIPA1L3	0.0553635	1
CEP68	0.0203154	1
ZNF839	0.0172596	1
PRKCH	-0.0621822	1
LEMD2	-0.0221301	1
FASTKD3	0.0293222	1
MIER3	-0.013746	1
ZBTB7B	-0.0084023	1
C4orf33	0.0398385	1
AC025181.2	-0.0345222	1
KCNMB1	0.0106832	1
NFYA	0.025875	1
MPV17L2	0.0148116	1
MAP4K2	-0.062554	1
RTRAF	0.077731	1
RAPGEF6	-0.0492011	1
CENPBD1	0.0413715	1
SPATA5L1	-0.0016189	1
ADGRG3	-0.0361985	1
ARRDC4	0.0379009	1
MAPKAPK5	0.0244378	1
DMXL2	0.002034	1
PYROXD1	-0.0009792	1
GGH	0.0045972	1
ZNF236	-0.043181	1
RTL10	0.0139352	1
FAM215B	0.0013447	1
PREX1	0.0524274	1
ZNF219	0.0121219	1
PITPNB	-0.0097258	1
PAG1	0.0100697	1
CHD6	0.0172388	1

JDP2	-0.0343296	1
BUB1B	0.0272938	1
GPATCH3	-0.0149777	1
AC005838.2	0.0273232	1
OSBPL2	-0.0178051	1
TNS3	-0.0111893	1
S100A13	-0.0706191	1
AL353807.5	0.0194563	1
TMEM87A	-0.0381291	1
PRRC2A	0.0150444	1
RPS19	0.0081171	1
FADS2	0.058113	1
FUT4	-5.299E-05	1
HTRA2	0.0275282	1
PDP1	-0.0777258	1
THOC5	0.0456212	1
TENT4A	-0.0389302	1
TBC1D9B	0.0692117	1
TMEM39A	-0.0318963	1
COMMD2	-0.0387239	1
CCNB1IP1	0.0381139	1
IDUA	-0.017962	1
SEPHS1	0.0412363	1
ENTPD4	-0.0392681	1
SPN	0.0966442	1
MPRIP	0.0200163	1
LZTR1	-0.0117298	1
MTO1	0.0104245	1
TTPAL	-0.0258413	1
ZNF500	0.0302976	1
TOMM70	0.0889212	1
DHX16	0.0093776	1
AC004812.2	-0.0298044	1
SHARPIN	-0.0153508	1
RPS6KB2	-0.0387005	1
MAP2K7	-0.0099886	1
AC073111.4	0.0110712	1
DCAF10	0.0609369	1
PRKRA	-0.0357646	1
ZNF468	-0.0199137	1
WDTC1	0.0300553	1
ZPR1	0.0249436	1
MRPS35	0.0152111	1
UFD1	-0.0605101	1
BAG5	0.0800173	1
STAT5B	0.0295881	1
FBXL8	0.0440599	1
CRYZ	0.0572039	1
PCNX3	-0.0200921	1
SMARCD2	-0.0293996	1

TRIM13	-0.0571189	1
HNRNPA0	-0.1177958	1
GMPPA	0.0044139	1
GBP3	0.0130176	1
CYB561A3	-0.0192538	1
MRPL23	0.1048525	1
MAGED2	-0.0133789	1
AIG1	-0.0105991	1
EEF1B2	0.0285186	1
ARHGEF18	-0.007493	1
ZNF852	0.0449152	1
ERV3-1	0.0503202	1
ZNF330	-0.0385343	1
SLC35C1	-0.0360865	1
PGD	0.057058	1
CCDC7	0.042613	1
ATPAF1	0.0455134	1
TMED2	-0.043137	1
DNAJB9	-0.0299196	1
AC016876.1	0.0177016	1
FEN1	0.0125935	1
NCOA6	0.0455309	1
FAM229B	-0.0510683	1
NEK4	0.0503923	1
BTN2A2	0.0106273	1
RECK	-0.0212623	1
EMILIN1	0.0361792	1
ORMDL3	0.0484437	1
SRGAP2	0.0164339	1
SKIV2L	0.0022274	1
BTBD3	-0.0560189	1
MTCH2	0.0231527	1
IPO4	0.0242835	1
AC015871.1	0.019291	1
SLC25A16	-0.0353417	1
DNAJB2	-0.0436299	1
HLA-DQA2	-3.725E-05	1
RIOX1	-0.0210185	1
SMIM3	-0.0780454	1
NECAP2	-0.1090091	1
UBR7	0.0059979	1
SERTAD2	-0.0227076	1
IQCE	0.0075697	1
MARK4	-0.0368665	1
HDAC3	0.0083755	1
ATP5MG	0.0264046	1
PANX2	0.0132303	1
C16orf91	0.011454	1
TMEM33	-0.0025262	1
AP002495.1	0.035065	1

TRIM69	-0.0016529	1
KIF2A	0.0276809	1
ZNF644	-0.0145813	1
OBSL1	0.0282621	1
NAA15	-0.0358745	1
CYCS	-0.0244509	1
CMTR2	-0.0273906	1
TCF25	0.0557813	1
AC144521.1	0.0129062	1
MAP3K20	0.0145531	1
SUMF2	0.043178	1
PPP2R1A	-0.0493506	1
DLGAP1-AS	0.0049049	1
STRN3	-0.0211361	1
CSNK1G1	-0.0560944	1
CBWD1	-0.000355	1
UPRT	-0.0013589	1
AL392172.1	0.0261856	1
CYB5R4	0.0460815	1
C12orf43	-0.0179027	1
FAM78A	-0.0511353	1
ATN1	-0.0141492	1
WNK1	0.0268742	1
ZNF107	0.0560577	1
RFX3	0.0320773	1
ZNF282	-0.0404115	1
WAKMAR2	0.0163854	1
CLIP1	-0.0099331	1
TTC33	0.0133501	1
KLF4	-0.1225199	1
PPM1L	-0.0260407	1
AP2A1	0.0032404	1
ACTR3C	0.024167	1
PLCXD1	0.0337667	1
GSTM5	0.0230735	1
LMLN	0.0173537	1
ABAT	0.0288621	1
CCP110	-0.0165125	1
ANKS1A	-0.0535724	1
GOSR2	-0.008907	1
CXorf40B	-0.0446493	1
SYNJ1	-0.053854	1
SMC3	0.0574434	1
KHDRBS1	-0.0729749	1
XPO6	0.0538827	1
SETX	-0.0290361	1
POLR1D	0.0219204	1
CERS5	-0.0314527	1
ZBED5	0.0216199	1
MED26	-0.0325899	1

ARPC1B	-0.1119518	1
KLF2	-0.2515821	1
ANKRD13C	0.0195234	1
EIF4G1	0.0918654	1
ZNF561	0.0223591	1
SMG8	-0.0100387	1
MORC3	-0.089886	1
KLHL2	-0.0246066	1
SNX14	0.0352187	1
PTPN4	0.0408963	1
RALBP1	0.0359086	1
RCOR3	0.0548329	1
SNAPC2	-0.0775128	1
MAP3K2-DT	0.0467498	1
GRWD1	0.019467	1
MDGA1	0.0181463	1
UBA52	-0.0155234	1
NR2C2	-0.0361385	1
VPS35	-0.0628688	1
FARP2	-0.0002766	1
LAMB2	-0.0250078	1
LETMD1	0.0314445	1
AC123768.4	0.0280268	1
ABLIM1	0.0095352	1
ZADH2	0.057667	1
USO1	0.1341494	1
ZNF417	0.0226355	1
POLG2	0.0146779	1
MED28	0.0192198	1
PRPF38B	0.1189187	1
NUBPL	0.0384009	1
PEX11B	-0.0461386	1
DDX56	-0.0324636	1
HIST2H2AA4	-0.0195429	1
SNHG19	-0.0333354	1
AC015871.7	-0.0186829	1
YARS	0.0407099	1
AC005921.2	-0.0237589	1
AGAP2	-0.0320503	1
DPM3	-0.0105619	1
RAB31	-0.0799473	1
ORC5	0.0121927	1
TBC1D17	0.0175314	1
AC002558.3	-0.0123369	1
MRPL37	0.0069983	1
CRYBG1	0.0029313	1
ZNF250	0.006254	1
SLC35A5	0.0387545	1
TUBGCP6	-0.0026971	1
MLLT6	0.0358596	1

SNRPB	-0.1339788	1
CRAMP1	0.008208	1
S100A9	-0.0731295	1
NELFCD	-0.0576501	1
DIP2B	0.0285258	1
NBPF11	0.0121677	1
TMEM25	0.0062353	1
LZTS2	-0.0595525	1
DCAF6	-0.0135313	1
NT5C2	0.0046048	1
LHPP	-0.0391924	1
RNF115	-0.0272963	1
CNOT3	-0.0090017	1
RPL10	-0.0559733	1
RPN2	-0.0082928	1
CASP2	0.0081129	1
CEBPD	0.039291	1
MTHFR	0.0063313	1
TM9SF3	-0.0740711	1
NOP9	0.000235	1
ATAD2B	-0.0987281	1
CRELD1	0.0241921	1
MFSD14B	-0.0078866	1
DDX59	-0.0156517	1
AC099343.3	0.0102601	1
EXD2	0.0266841	1
CTNNA1	0.0288394	1
MSL2	0.0452596	1
CCDC32	-0.0096939	1
AC016949.1	-0.0423043	1
TWSG1	-0.014893	1
LARS2	-0.0003959	1
CLASP1	0.0271766	1
OCRL	-0.0063909	1
TMEM175	-0.0190592	1
UGDH	0.0263071	1
APOBEC3G	-0.0075757	1
ACRBP	-0.0222114	1
USP8	-0.0325996	1
KIF9-AS1	0.0302329	1
SLC7A6	-0.0655194	1
ARIH2	-0.0250885	1
RAP2A	-0.0012306	1
C12orf49	-0.0088709	1
PACS1	0.0003706	1
TRIP4	-0.020722	1
POLR3F	-0.0339401	1
DNM1	0.0123032	1
SSR4	0.0118828	1
TRIM41	-0.0347893	1

ACAD11	-0.0063207	1
PTPA	-0.0335802	1
NPLOC4	-0.0479967	1
ARFGAP1	-0.0510384	1
AP5M1	0.025179	1
RASA1	0.0337612	1
ZW10	0.0256081	1
TP53INP1	-0.0238517	1
NUP160	-0.0111445	1
ZC3H3	-0.0402138	1
RTP4	0.0368791	1
ARMH3	-0.0300276	1
ACTR8	-0.0017788	1
COPS7B	-0.0110337	1
DDX42	0.0589638	1
COMMD7	-0.085562	1
POGLUT3	0.0191959	1
PIK3CD	0.0385155	1
AKR1B1	0.0065143	1
ZNF888	-0.0903802	1
MRPS36	0.0735601	1
UBXN2B	-0.0162309	1
RPLP1	0.0366694	1
MAN1B1	-0.0062276	1
ARMC8	-0.0746677	1
LINC00963	0.0090922	1
HIVEP3	0.0173926	1
WASL	0.0038193	1
SDHB	0.0355334	1
NFX1	-0.0093508	1
IMPAD1	-0.049929	1
VPS13B-DT	0.0229311	1
SLC2A11	0.0244258	1
LRRC41	-0.0039128	1
ZNF77	0.0246659	1
SLC25A1	0.0219581	1
IPO11	-0.0481019	1
UFM1	0.0553698	1
CSTF3	0.0588795	1
NOCT	0.0173057	1
TRMT12	0.0255808	1
AC090517.5	0.0288975	1
XRN1	-0.0334433	1
C3orf62	-0.057183	1
GK	-0.0206524	1
DGCR2	0.0041241	1
FASTKD5	0.0167435	1
RAB21	-0.1105485	1
BAHD1	-0.0548482	1
AC073896.2	-0.0013918	1

TSPYL4	0.0065181	1
DOP1A	-0.0319585	1
PLCB2	-0.0127671	1
TTI2	0.0133876	1
METTL3	0.0374979	1
COX7B	0.0663443	1
FAM8A1	0.0125994	1
PABPC1L	-0.0299188	1
SMN1	-0.0137824	1
KIF22	0.0192963	1
MTF1	-0.0373569	1
COPG1	-0.0043004	1
MFSD11	-0.0275219	1
BBS9	0.0121902	1
CCHCR1	0.0355613	1
IPO13	0.0286784	1
ZBTB45	-0.0416709	1
ZKSCAN8	-0.0143947	1
PAQR6	0.0158044	1
YIPF4	-0.0627796	1
TGFBRAP1	-0.0379655	1
E2F5	-0.000307	1
DNAJC15	-0.0515408	1
ZG16B	-0.017397	1
ASNSD1	0.0396113	1
RRAS	-0.0491451	1
TKFC	0.0057744	1
ANKZF1	0.0364708	1
MYO5C	-0.0062314	1
RBM15	0.0103736	1
TRIM52-AS1	-0.0625662	1
EIF4B	-0.0022968	1
LRRC8B	-0.0661283	1
TBCCD1	0.0129099	1
ATP5F1E	0.0770634	1
ZCCHC2	-0.0397766	1
ZNF749	0.0100029	1
MSRA	-0.049543	1
SH2B1	0.0090739	1
WDR44	-0.0640478	1
MFN2	-0.0262269	1
RFC1	-0.0649391	1
PSENEN	-0.0350961	1
MED18	0.0405953	1
NIBAN1	-0.0009535	1
TAFA2	-0.0055564	1
TOMM22	0.0567157	1
UQCRC1	-0.0682448	1
R3HCC1	0.0384826	1
ZMYM2	-0.0615681	1

THAP1	0.0154301	1
TRNT1	0.0097566	1
ZNF268	-0.0002122	1
MMUT	0.0287242	1
FAM13B	-0.0359041	1
KDM5A	-0.0950904	1
SLC66A3	0.0721837	1
USF3	-0.0206738	1
CEP95	-0.0450784	1
ID2	-0.0909474	1
RCBTB1	0.0635199	1
ZDHHC16	0.0184996	1
IFI44	0.0385022	1
DPH3	-0.0675557	1
KCTD3	-0.0192012	1
ZNF180	0.0234487	1
MOV10	-0.0339996	1
LKAAEAR1	0.0193516	1
TAF13	-0.0397922	1
ADGRG5	-0.0629569	1
ABHD11	-0.0194148	1
INPP5K	-0.0079889	1
LMO7	0.0362806	1
C1orf54	-0.0325723	1
RBM4B	0.0384769	1
PTTG1IP	0.0590369	1
POLM	0.0057312	1
SERP1	0.0955686	1
PEX3	0.0254482	1
TRAF3IP3	0.0776266	1
SLC66A1	-0.0231104	1
SLC29A3	0.0219309	1
TRUB1	0.0334871	1
BCL7C	-0.0693575	1
TAB2	-0.0516916	1
ESRRA	0.0247309	1
VKORC1	0.1192906	1
STRN	-0.011466	1
CENPS	0.0017269	1
SCMH1	0.035027	1
C16orf86	0.0181207	1
KLF7	0.003417	1
SNX1	-0.0272061	1
GTPBP8	0.0069358	1
PRPS1	-0.0568776	1
GRAP	-0.0335182	1
AKR7A2	0.0875529	1
SSR2	-0.0110925	1
TAP2	0.0015924	1
TBC1D2	-0.0320703	1

CBLL1	-0.0482701	1
EFCAB7	0.0249922	1
CREBBP	-0.0178026	1
CCNG1	-0.0921151	1
LSM11	0.0335728	1
CALCOCO2	-0.0242597	1
CDT1	-0.1407949	1
SMPD4	-0.0723576	1
GTF2H4	0.014886	1
CFAP410	0.0242248	1
NQO1	-0.0143218	1
SUPT3H	0.0337354	1
PLOD3	-0.0407307	1
SUPT6H	-0.0713045	1
PGLS	-0.0803854	1
DNAL1	-0.0872017	1
ACAD8	0.0320628	1
SEC24D	-0.0149268	1
IFT43	-0.0246788	1
HSPA9	0.1063297	1
DBR1	0.0271704	1
ERCC3	0.0353234	1
GAB1	0.0256152	1
NXF1	-0.0079668	1
KAT2B	-0.0134798	1
RNF24	-0.018652	1
SH3RF1	-0.0676099	1
B4GALT4	-0.0130484	1
COTL1	-0.0537754	1
LCMT1	0.0097552	1
EIF3L	0.0145716	1
FUNDC2	0.0376616	1
BSPRY	0.0409411	1
IRS2	0.0022766	1
CLP1	-0.024279	1
HOXA9	0.054566	1
WDR91	-0.0029798	1
MOB1B	-0.0170562	1
AC091271.1	-0.0241869	1
INSIG2	-0.0294383	1
MINDY3	-0.0580133	1
PHC1	-0.0131867	1
INO80D	-0.032405	1
INTS4	0.0220515	1
DPP3	0.0080069	1
NAA25	0.0296135	1
FXR2	-0.0611149	1
AC092279.1	0.0004518	1
POGLUT2	0.0177735	1
MAZ	0.059249	1

RNF141	0.0093207	1
DNAJC18	-0.0327976	1
SEC23A	-0.1092643	1
YTHDF1	-0.0506708	1
RASAL3	-0.0313426	1
MAP4K1	-0.0035898	1
RUSC1	-0.029014	1
ESS2	-0.0664162	1
POP1	0.0207344	1
ITGB1BP1	-0.0731114	1
AC120053.1	0.03304	1
RABGAP1L	-0.0094499	1
DDX49	0.0059557	1
PCGF5	-0.0476365	1
TSC2	-0.0364666	1
C3AR1	0.0347301	1
YTHDF3-AS	-0.0389916	1
CD180	0.0565318	1
TNFSF13	-0.0924368	1
ZNF875	0.010388	1
KIAA2026	-0.0104716	1
LIN37	-0.0407626	1
ZNF140	-0.057596	1
RBM48	0.0216697	1
TMEM205	-0.0524381	1
AC007114.1	0.0514486	1
TMEM53	-0.0158892	1
SLC9A7	-0.011523	1
PHRF1	-0.0137487	1
MLH1	-0.0284598	1
ADHFE1	0.0318773	1
NAAA	-0.0368573	1
MOB1A	-0.1262134	1
TRIM34	0.0788015	1
ENPP2	0.0024273	1
INAFM2	0.0296941	1
KAT2A	-0.0098729	1
DZANK1	0.0234184	1
KLHL20	-0.0089634	1
ABHD12	0.0013856	1
KLRG1	0.0304577	1
HNRNPR	0.1091	1
TTC7A	-0.0245967	1
IMPA1	0.0067775	1
DUSP6	-0.1445252	1
ZBTB10	0.0043887	1
PLAGL2	-0.0204577	1
EPS15L1	0.01287	1
FAAP100	0.0059145	1
AC004241.1	-0.029771	1

ARHGEF17	-0.0306216	1
RAB18	-0.0569932	1
OPRL1	-0.0005014	1
NPC1	-0.0454199	1
GRIK5	-0.0177816	1
MCAT	-0.0560142	1
MAP4K3	-0.0326199	1
AL161457.2	0.0060145	1
SEPTIN7	-0.0498508	1
SCYL3	-0.0046303	1
BCKDK	-0.002257	1
NUDT3	0.0416001	1
SLFN13	-0.0046467	1
RING1	-0.01428	1
ALOX12-AS1	0.0037246	1
BLOC1S6	0.015351	1
AGTPBP1	0.0069161	1
SLC31A1	-0.0387314	1
TECPR2	0.0304624	1
SEC24A	-0.0537089	1
ARNTL2	0.0083642	1
ABHD4	0.0050915	1
TMEM250	-0.0700482	1
SH3BP4	-0.0380771	1
UNC119	0.0065593	1
ANP32A	0.077572	1
TRAF2	-0.0280176	1
KLHL7	-0.0505557	1
PRRC2B	-0.0320189	1
SREBF2	-0.0952209	1
EMILIN2	0.0511358	1
HEXD-IT1	0.010163	1
LRWD1	0.0082535	1
AL353194.1	0.0129965	1
AC005332.6	0.0047053	1
NAPRT	-0.0161536	1
TDP2	0.0040704	1
C6orf136	-0.0180523	1
SLC27A3	0.0250484	1
AC010615.2	0.0255006	1
ADAMTSL4-	0.0013793	1
RB1	0.1088664	1
CPNE2	-0.0135616	1
LRP8	0.0026167	1
HOXA10	-0.0052681	1
PPOX	-0.0044058	1
RHOB	-0.0797961	1
PARD6A	-0.0580434	1
ACSL1	-0.0001906	1
GALC	0.0126294	1

TMLHE	0.0356019	1
C11orf71	-0.046922	1
ZNF671	-0.0221689	1
GON4L	-0.1220892	1
MLLT10	0.0598808	1
AP000873.2	0.0351789	1
CCNT1	0.0034941	1
TBC1D20	-0.0264606	1
LIMS1	-0.0151142	1
FBXO8	-0.0372998	1
POLR2M	-0.0132235	1
TBPL1	-0.0028695	1
GYS1	0.0345453	1
ATAT1	-0.004272	1
SPATA7	0.0149053	1
DPP9	-0.0287148	1
POLR2C	-0.021952	1
LINS1	0.0012065	1
ZNF76	0.0120936	1
TM9SF1	-0.0761598	1
SAP30L	0.0100351	1
ZNF688	-0.0587526	1
ANKRD33B	-0.0234329	1
STAT5A	-0.0130881	1
RTTN	0.0264243	1
STARD3	-0.0264672	1
HIST1H1E	0.0613355	1
SLC16A5	-0.0423904	1
MEF2C	0.1093025	1
LPCAT3	-0.0462704	1
FLT3LG	-0.0157354	1
FASTKD2	-0.0067265	1
C18orf32	-0.0561012	1
FLVCR1	0.0089013	1
NR4A2	-0.0046329	1
ELOB	-0.0303642	1
CALML4	0.020054	1
FLVCR1-DT	0.0304373	1
MPO	0.1573395	1
ABCA2	-0.0145899	1
CGAS	0.0069909	1
FRYL	0.0674852	1
ODR4	0.0101831	1
FCHSD1	-0.0210329	1
LMBR1	0.0085976	1
EMC10	0.008627	1
GSS	0.0467984	1
FKBP11	0.0432693	1
ICA1L	-0.0260041	1
MYLPF	-0.0095997	1

IRAK4	0.0708647	1
SARS2	0.0214249	1
CCNG2	-0.0659683	1
ZNF619	0.0308439	1
ING5	0.0417675	1
EFHC1	-0.0690523	1
APEX2	-0.0164763	1
SLC35B2	-0.0021233	1
ARFIP2	-0.0250703	1
INTS1	-0.0227774	1
PAN2	0.0059356	1
ANKRD50	0.0297489	1
URB1-AS1	-0.0432054	1
DICER1	0.0090492	1
FAM117A	0.0024664	1
NADSYN1	0.0278575	1
TRIT1	-0.0067558	1
ZFP30	-0.0004665	1
PHF8	0.0237994	1
ENKD1	-0.0065536	1
KMT5B	-0.0668199	1
NUDC	0.0135873	1
PPP4R3A	-0.010619	1
ARAP2	0.0200158	1
AL662844.4	0.0052429	1
DNASE1	-0.0658039	1
SREK1	0.0699263	1
NFYC	0.0042815	1
SUMO2	-0.0234554	1
AC097634.1	-0.0065596	1
MAN2B1	0.0046298	1
ING3	-0.0241749	1
SRSF8	0.0366717	1
MAN2A1	-0.0267645	1
ACER3	0.0089494	1
NUP58	-0.0107446	1
GPN1	-0.0356441	1
ACTA2	0.0581938	1
SIN3B	0.000573	1
JOSD2	0.0279558	1
TNFRSF10B	-0.0393175	1
CNBD2	-0.0018228	1
NELFB	-0.0238461	1
THRAP3	0.0206612	1
MROH1	0.0242442	1
AC067852.2	-0.0071219	1
NAPA	-0.0839767	1
GXYLT1	-0.0044115	1
QSOX1	-0.0330651	1
NYNRIN	0.0021014	1

SATB1	0.1036818	1
LINC00630	0.0021995	1
ZNF189	-0.0303244	1
CCSER2	0.0175259	1
MIA2	-0.0013127	1
DGCR6	-0.0384115	1
RACGAP1	0.0250356	1
HYOU1	-0.0057493	1
RFX5	-0.0265944	1
MCRS1	-0.0090596	1
NSMF	-0.0389998	1
DNM1L	-0.0240127	1
DENND1A	0.0018178	1
ZSWIM8	-0.0190132	1
LSM10	0.0606015	1
DVL2	-0.0025207	1
TSPO	-0.0093914	1
CPNE1	-0.014452	1
TMEM8A	-0.0224128	1
FBXO5	0.0256699	1
GALNS	0.0287808	1
ALDH2	-0.0573937	1
OXCT1	-0.0440097	1
ZKSCAN4	0.0128921	1
CXCR4	0.0052597	1
DMAC2	0.0270409	1
ZNF350	-0.0424463	1
POLL	-0.0014287	1
RBM10	0.0375547	1
RBMXL1	0.0132446	1
ZNF275	-0.0430224	1
INO80	-0.0236887	1
MAML2	-0.0441695	1
NET1	0.0534288	1
PCK2	-0.0353137	1
KIF13B	0.001726	1
NAA80	0.0285687	1
FGFBP3	0.0087249	1
AIF1L	0.0527556	1
ZNF420	0.0269378	1
DBNDD2	0.0087162	1
PPP1R12C	-0.0362883	1
MEGF9	-0.0558881	1
ZRSR2	-0.0147311	1
OXA1L	0.0766515	1
SMG9	0.0090458	1
ACSS2	-0.0148056	1
HECTD3	-0.0218673	1
TRIM23	-0.0054838	1
BAP1	-0.0325576	1

ZNF175	0.0171572	1
DTX2	0.0040215	1
IFI27L1	-0.0318174	1
STK25	0.0038494	1
TRMU	-0.0112375	1
NEK8	-0.0127502	1
CDKN2AIPN	0.0520547	1
CLYBL	0.0437087	1
NAXD	-0.0214366	1
ZNF623	0.0130931	1
ANKFY1	0.0169341	1
PTTG1	-0.0048562	1
PPP1R3F	-0.0216034	1
LRCH1	-0.0332987	1
RNF185	-0.0201157	1
ATG12	0.0336477	1
VPS37A	-0.0664795	1
MAN2C1	-0.042688	1
MARCH6	-0.0125113	1
ATP5PO	0.0139451	1
AC132872.1	-0.0195792	1
PYGB	0.0121122	1
AC026124.2	-0.0313203	1
CPQ	-0.0433326	1
RAB29	-0.0545767	1
UBTD2	-0.0236293	1
RNF139	-0.0212527	1
PRKAR2B	0.01788	1
AL050309.1	0.0268867	1
SMG1	-0.0582353	1
KRI1	0.0364637	1
TRIM65	0.0287651	1
CLINT1	-0.0920923	1
AAR2	-0.0119786	1
DAPK1-IT1	-0.0160041	1
SLC25A30	0.0142328	1
ZNF584	-0.0109998	1
TIGAR	-0.023352	1
RPL19	0.0328086	1
SAMD10	0.005932	1
DDX58	0.0426325	1
MFSD9	0.0051005	1
PTPN23	0.0076626	1
BROX	0.0339432	1
SPRYD4	0.0402022	1
DDRGK1	0.008494	1
RBM18	-0.0111905	1
TMCC1	0.0002229	1
MRPL9	0.0066223	1
SCAF11	0.0723077	1

FBXL18	-0.0071576	1
ANAPC4	0.0138559	1
MRPL21	-0.0404456	1
ATP8A1	0.0327467	1
ARMCX5	0.0058811	1
FAR2	0.0286231	1
ZNF549	-0.0293218	1
DIAPH1	0.0045887	1
AP5B1	-0.0730449	1
CKM	-0.0045355	1
MARCH1	-0.0250309	1
NAPB	-0.0430146	1
STYX	-0.0107127	1
COQ4	-0.0416252	1
FAM189B	0.0131586	1
ZNF569	-0.0172143	1
PHF5A	-0.0912845	1
OPA3	-0.0063602	1
RARS2	-0.0394766	1
DNAJC30	-0.0579975	1
PNRC1	0.0284593	1
MOB3C	-0.0338805	1
LRRC37B	-0.0789502	1
FAM98C	0.0153291	1
ERMAP	0.001316	1
NIPBL	-0.0538243	1
RAD23B	0.0712588	1
APOO	0.0307174	1
AC027682.4	0.0061256	1
NCAPH2	-0.0241435	1
PJA1	-0.0123894	1
LAMTOR5	0.0029742	1
MT-ND1	-0.1357097	1
COX6C	-0.0478844	1
ICMT	0.0258459	1
ZNF668	0.0176497	1
MYCBP	0.0949554	1
SLC9A8	-0.0303562	1
KHDC4	0.0178244	1
AL355075.4	-0.0006963	1
SLC24A1	0.0396617	1
TSPAN13	0.0266009	1
ZNF211	-0.0358605	1
RWDD3	0.0149274	1
APOBEC3D	0.0141803	1
PAQR3	0.0327071	1
ERAL1	0.0072805	1
ZNF595	0.0146202	1
IFI35	0.0244473	1
E2F4	-0.0037317	1

WASHC4	-0.0154325	1
CDK8	0.0343808	1
MEMO1	-0.0307363	1
ENTPD6	0.0093488	1
RASGEF1A	-0.0087938	1
AGPAT1	0.0186245	1
UTP15	0.0029489	1
BBS7	0.0373533	1
MRPS23	0.0776151	1
UFSP2	-0.0679337	1
TKT	0.0113177	1
ZNF827	-0.0026847	1
USP40	0.0380883	1
AARS	0.0105359	1
TSEN15	0.0132536	1
ZNF773	-0.0085575	1
RANBP10	-0.0131413	1
DCAKD	0.0118571	1
SLC25A13	-0.0285403	1
ZNF470	0.0084582	1
SNX12	-0.0037624	1
MIEF2	-0.0320285	1
UBOX5	-0.0422434	1
NAA20	0.0309189	1
NEIL2	0.0059582	1
MRNIP	0.0241339	1
ELMOD3	0.0396593	1
OSTF1	0.0372491	1
TMEM243	-0.0476476	1
HLA-DMB	-0.0229374	1
AC090152.1	0.0341277	1
STARD8	-0.0535618	1
FBXO31	-0.0325723	1
GORAB	-0.0275115	1
MOSPD3	0.0086046	1
NAA30	-0.0266614	1
TIMM29	-0.0028885	1
PLEKHF2	-0.0729818	1
C3orf80	-0.1278598	1
NCAPG2	0.0207011	1
TRIP12	0.0933745	1
TNFSF12	0.0102009	1
RAB2B	-0.0041466	1
AC092329.4	0.0040875	1
HEATR6	0.0256757	1
CSF2RA	0.039626	1
WDR47	-0.0632824	1
FAM120B	0.0303275	1
OST4	0.0093214	1
SRP54	-0.0054866	1

THAP3	-0.0093499	1
SLC5A10	0.0075491	1
TERF2IP	-0.0373215	1
CCSAP	0.0244346	1
HIST1H3D	-0.0930645	1
CCZ1B	-0.0126281	1
DNAAF4	-0.0072603	1
HACE1	0.0245048	1
PDP2	-0.0007408	1
ABL1	-0.0255445	1
FAM149B1	0.014165	1
LINC00921	0.0103619	1
ARL13B	-0.0464254	1
KLF11	-0.0189301	1
EMC9	0.0083804	1
TXNL4A	0.0566308	1
EPG5	0.0172149	1
AL353622.1	0.0063658	1
DSC2	0.0576019	1
FAM122B	-0.0279977	1
PPP1R9B	0.0034517	1
TCERG1	0.0094706	1
GPR137	-0.0240353	1
ARPC2	-0.0926691	1
BET1L	-0.0314574	1
ACCS	0.0064989	1
MRPL41	0.0037544	1
STX2	-0.0323754	1
NDUFB8	0.0376821	1
SGSM3	-0.0403437	1
HAUS5	-0.007776	1
ZER1	0.0082974	1
RBBP8	0.0411131	1
SNAP47	0.0088685	1
P3H1	0.0423027	1
TAOK3	-0.0429469	1
CLSTN3	-0.0408654	1
TTI1	0.0282506	1
ZNF682	0.0023572	1
ABHD2	-0.0069937	1
MMP25-AS1	0.0265444	1
REV1	-0.0298646	1
CIAPIN1	-0.0149808	1
AC127521.1	0.0420845	1
BABAM2	0.0271463	1
SLC9A1	0.0106633	1
AC100810.1	-0.0424478	1
RANGAP1	0.0338752	1
HM13	0.0367115	1
SORBS3	-0.0242053	1

TMEM127	-0.0740392	1
RPL27	0.0532854	1
GTF2E1	-0.0195014	1
PTAR1	-0.0751215	1
POLB	-0.0163868	1
PDK2	0.0257775	1
LMNB2	-0.0261417	1
MPP5	0.0195805	1
DMAP1	-0.0129473	1
ZNF709	-0.0142575	1
TNRC6C	0.021456	1
RAB33B	-0.0097005	1
ARHGAP35	0.0394881	1
SRGN	-0.0541526	1
CDADC1	0.0514106	1
MIR3936HG	0.0302923	1
MT-ND5	0.0498753	1
POR	-0.0035154	1
SLF1	0.0513421	1
PES1	0.0242716	1
AL121603.2	0.0021515	1
RPTOR	0.0148025	1
ZNF35	0.0011576	1
CENPT	0.0094649	1
GALE	-0.0308285	1
LRRC47	0.0450283	1
ZWILCH	0.0216001	1
ARAF	-0.0612018	1
TNKS	0.0130846	1
DOHH	-0.01219	1
ZMYM5	-0.0633398	1
ZNF736	0.0068801	1
PCGF1	-0.0314574	1
ZNF778	0.0124196	1
SRPRA	0.0014775	1
ARMC7	-0.068656	1
RPL3	-0.005076	1
RHBDF1	-0.030784	1
FAM153CP	0.0165697	1
KRTCAP2	0.0270133	1
RALGAPB	-0.0308933	1
RSL24D1	-0.0872604	1
ABHD15	-0.0629268	1
ZEB1-AS1	-0.0407364	1
C22orf39	-0.0325086	1
AC044849.1	-0.0346572	1
TP53RK	0.0055257	1
HMG20A	-0.0097165	1
MANEA-DT	0.0389728	1
PSKH1	-0.0442175	1

HCFC1	0.0340628	1
GZF1	-0.0088675	1
COX7A2L	0.014138	1
SMIM14	0.0190752	1
SNX11	-0.0124893	1
FANCG	0.012372	1
ZNF846	0.0073991	1
SMIM24	0.1047395	1
AC008875.3	-0.0083248	1
LRFN4	0.020808	1
FGGY	-0.0106222	1
SGMS1	-0.011983	1
TIE1	-0.0029525	1
BCR	-0.0288754	1
GATA2-AS1	-0.0063323	1
IFT46	-0.005898	1
ABHD3	0.0075231	1
KCTD13	-0.0029227	1
MEF2D	-0.0654326	1
ZNF552	0.0029285	1
NIPSNAP3B	0.0361651	1
MEST	0.0306459	1
PICK1	0.0011382	1
ZNF333	0.0091306	1
RPL4	-0.0772812	1
SERPINA1	-0.0396661	1
MSH6	0.0254559	1
PCLAF	-0.0596323	1
YY1	-0.0359711	1
SENP1	-0.0379233	1
COG8	0.018342	1
HIST1H1B	0.0285412	1
ICAM4	-0.0021909	1
CSAD	-0.002736	1
DOP1B	-0.0266878	1
SYS1	-0.028916	1
ZMIZ2	-0.0423152	1
AC048341.1	-0.039764	1
HLA-DMA	-0.052943	1
PSMA7	0.0419649	1
TMTC3	0.0048061	1
AC083862.3	-0.0190348	1
KNTC1	0.0060765	1
UNC119B	-0.0454872	1
FOXJ2	-0.0012403	1
HSPA4	-0.0143712	1
RSBN1L	-0.0389244	1
TOE1	0.0491228	1
CYB5D1	-0.0150878	1
ZNF407	0.0058678	1

TYMS	-0.0488988	1
PRAM1	-0.0312463	1
LINC00294	0.0059534	1
SRCAP	0.0011568	1
BOLA3	0.0265269	1
R3HCC1L	-0.0139527	1
PIGZ	0.0253859	1
PIP5K1A	-0.0545824	1
ZNF611	0.0014814	1
COASY	0.0383101	1
AP1M1	-0.0289476	1
TBC1D22A	0.024006	1
FKBP2	0.0038568	1
PINK1	0.0290347	1
RPL10A	-0.0606139	1
USB1	0.0082202	1
AC083799.1	-0.030042	1
BBS4	0.0303433	1
SYNCRIP	0.0446788	1
AC092338.3	0.0129264	1
DXO	-0.0266521	1
MYO19	0.0330931	1
ABHD18	0.0071648	1
TNNT3	-0.059948	1
TRAF3IP2-A	0.0057965	1
RPAP3	0.064092	1
ZNF586	-0.0016128	1
NEK3	0.0321038	1
WDR81	-0.0291125	1
THOP1	-0.0099998	1
SH3GLB2	-0.040761	1
EIF2D	-0.0299263	1
SMPD1	-0.0275518	1
LIG1	0.0355991	1
MMP28	-0.0176165	1
RIPK1	-0.0085212	1
RAP2C-AS1	-0.0187556	1
ERI1	0.0084687	1
APH1B	-0.0233903	1
ZNF467	-0.0032633	1
ACTR5	0.0120771	1
TUBA1B	-0.2221774	1
ZCCHC3	-0.0104462	1
PPM1D	0.0120356	1
RBM7	-0.0558272	1
UPK3BL1	-0.0379014	1
KIAA0355	-0.0245512	1
HDGF	0.0014986	1
NLE1	0.0112167	1
CCNB1	0.0496308	1

RPL29	-0.065687	1
TPK1	-0.0587419	1
SMARCA4	0.0609079	1
RPL6	-0.0102207	1
VCL	0.0316914	1
HOXA10-AS	0.021132	1
CRNKL1	-0.0275999	1
INO80C	0.0278607	1
RAD51B	0.0186413	1
UCK1	-0.0187413	1
AC114980.1	-0.0303737	1
JMJD8	-0.0158977	1
PITPNM1	0.0132928	1
GCC1	0.005253	1
SIRT6	-0.0036538	1
RCL1	0.0110208	1
SPCS1	-0.1035528	1
SMAD1	-0.0124228	1
AC025159.1	-0.0272749	1
ZBTB18	-0.0370409	1
PTGS2	-0.0241764	1
PIDD1	-0.0238828	1
C1QTNF4	0.031666	1
CEACAM4	-0.0209418	1
AC104532.2	0.0251416	1
ZKSCAN5	0.0008421	1
LDLRAD2	-0.0376581	1
ZNF133	0.0123643	1
HIGD1A	-0.0697533	1
TRMO	-0.031036	1
ZNF527	0.0174192	1
CALM2	-0.0097948	1
SFMBT2	-0.0034292	1
RBM5	-0.0776356	1
PRMT9	-0.013216	1
ZFHX2-AS1	-0.0027893	1
AMT	0.0114627	1
CEACAM21	-0.0187154	1
PFDN4	-0.0242668	1
FYN	0.0260695	1
ZNF792	-0.0234108	1
SUPT7L	-0.0483162	1
FXYD5	0.0163519	1
USP38	-0.0306372	1
LINC00173	0.0036524	1
NTN1	0.0192141	1
OAS3	-0.009611	1
ITGAL	0.071861	1
CUL4A	-0.0187055	1
SZT2	0.0097368	1

LYSMD3	-0.028768	1
JAK3	-0.0026401	1
RABGGTB	0.0539725	1
IER5L	-0.0386387	1
ENPP4	0.012392	1
SNX13	0.0047528	1
HEIH	-0.0213938	1
WDR25	0.0083787	1
SYPL1	0.0115875	1
ZNF324	-0.0102205	1
DOCK1	0.0931043	1
TOM1L1	0.0130314	1
HMGA1P4	-0.0217316	1
FIS1	0.0431508	1
IQCG	-0.0183083	1
MARF1	-0.0218408	1
PUS10	0.0122455	1
TMEM44	0.0220693	1
SLC25A51	-0.0091614	1
ALMS1	0.0228756	1
ITGA2B	-0.012045	1
SHF	-0.0472776	1
OGFRL1	0.0356983	1
SYNGR1	0.0365373	1
TXNRD2	0.0098749	1
IGHMBP2	-0.0694562	1
CCRL2	0.0047315	1
ARMCX1	-0.0178482	1
NDUFV2-AS	-0.0256821	1
ZZEF1	-0.031437	1
CLASRP	-0.0019682	1
ATXN7L3	-0.0213247	1
GOLT1B	-0.0762439	1
PRPF3	0.014227	1
MON2	-0.0386582	1
CYFIP2	-0.0192868	1
GTF2IRD2B	0.0161334	1
PTMS	0.0020422	1
AQP3	0.0239113	1
FAM185A	0.0027052	1
ADAM28	0.0107172	1
RIC1	-0.0356263	1
ZNF776	0.0054617	1
KBTBD4	-0.0200675	1
AKAP8	-0.0164353	1
STAM2	-0.0302562	1
SNHG11	0.0112498	1
ZBTB11-AS1	-0.0116437	1
RMI2	-0.0117001	1
AL512625.3	-0.0228508	1

HIST1H2AG	0.0035606	1
JAG1	-0.0260688	1
ZNF600	-0.0425233	1
REX1BD	0.0813914	1
AC007336.1	-0.0606087	1
GEN1	0.0085568	1
DNAJB1	-0.0235501	1
NDUFB11	0.0220843	1
NKG7	0.0115724	1
GATA2	0.0230674	1
HIRA	0.0270939	1
SMG5	-0.0185416	1
EPB41	0.0518907	1
ZNF136	0.0020614	1
VPS39	-0.02971	1
HVCN1	-0.0383734	1
MTA2	-0.0010553	1
ZBTB48	-0.0133824	1
INPP5E	-0.0428315	1
ACP1	0.0352296	1
AL050341.2	-0.0190511	1
VMAC	-0.0186943	1
TRAFD1	0.0043628	1
GTF2IRD2	-0.0220882	1
TUBE1	-0.0389026	1
CCDC191	-0.0061861	1
COX20	-0.000147	1
INIP	0.0396713	1
DCTN1	0.0081283	1
RHOBTB1	-0.0099971	1
ZDHHC8	-0.0403989	1
RBM19	0.0115683	1
CS	0.0594488	1
INVS	0.0189006	1
ZNF615	0.0073909	1
OSM	-0.0312146	1
GTPBP2	-0.0220994	1
SP2	-0.0564521	1
C19orf25	-0.0134583	1
MBD1	-0.0028607	1
SSH3	-0.0021134	1
TRGC1	-0.0080858	1
FBF1	0.012387	1
KLHL5	-0.0760656	1
LTV1	0.0262746	1
PIGG	0.0120118	1
CLSPN	0.014072	1
IFITM3	0.0364731	1
COQ8B	-0.01313	1
RBM12	-0.0595335	1

ZNF384	0.0323261	1
CFLAR	-0.00493	1
IL12A	-0.0384013	1
TSC1	-0.071267	1
BASP1	-0.0412814	1
SLC35A4	0.0047053	1
BBS1	0.0031595	1
CHEK1	0.004519	1
GINS2	0.0159213	1
PSME2	0.0714002	1
PSPC1	-0.013847	1
BST1	-0.0088643	1
AL139260.1	0.0172919	1
TAF1A	-0.0050389	1
EMSY	0.0023994	1
AL591895.1	-0.0350023	1
PROSER2	-0.0029318	1
MRPL28	-0.0222142	1
ERP29	0.0166353	1
AC012615.1	-0.0145127	1
FAM161B	-0.0222504	1
ASB1	-0.0602376	1
RPL35	-0.0059055	1
ZFYVE28	0.0040453	1
LMF1	0.0221004	1
AC074032.1	0.0262689	1
ATG16L1	-0.0231169	1
LAMP1	0.0037015	1
KDM4C	-0.0013922	1
FPGS	-0.0132008	1
AC087190.1	-0.0138285	1
TMEM263	-0.0016426	1
FAM53B	0.0129157	1
TTC7B	0.0396764	1
NFIX	0.0330219	1
EIF5B	0.0259731	1
RPL7A	-0.0350138	1
EXD3	-0.0269714	1
ZDHHC4	0.0542243	1
BGLAP	0.0034839	1
MIR1-1HG-A	-0.0105458	1
AL096865.1	0.0142959	1
C9orf78	0.0427125	1
SRRD	0.0155766	1
RPS5	-0.0847496	1
ACOT8	-0.015836	1
ZFP1	-0.0349332	1
SIAH2	-0.0019843	1
ASL	-0.0732118	1
C12orf76	-0.0331871	1

ABCA7	0.0090899	1
TBC1D7	-0.0028204	1
DENND4C	-0.0056418	1
NCEH1	0.0246323	1
IL6ST	-0.0281636	1
AEN	-0.0116531	1
CTNNAL1	-0.0090549	1
KRBOX4	-0.0099928	1
BTF3	0.0253734	1
SLC38A1	-0.0848515	1
MYSM1	0.0347009	1
C7orf25	-0.0066822	1
LINC01943	0.013225	1
HCFC1R1	-0.0229164	1
ZBTB5	-0.0112243	1
FGD5-AS1	-0.0134784	1
PTP4A2	-0.0179023	1
DCUN1D2	-0.0054512	1
PROCA1	0.0064602	1
ZNF154	-0.0096558	1
INTS6L	-0.0002588	1
ARHGAP5	-0.0212324	1
TSC22D1-AS	0.0142209	1
ZNF701	0.0137569	1
EML2	0.0314955	1
MEX3C	-0.0581492	1
LYST	0.0105692	1
EIF4A1	0.0001276	1
MTPAP	0.0046344	1
TMCO4	0.0212909	1
HNRNPK	-0.0196305	1
TBC1D22B	-0.0239453	1
TCTE3	0.0029316	1
TMEM254	0.0128858	1
AC103591.3	-0.0779775	1
ASTE1	-0.0338548	1
APTX	-0.0231412	1
RABL2B	-0.0231469	1
CAPS	-0.0122763	1
HIST1H2BF	-0.0152799	1
EBLN2	-0.035646	1
ZNF747	0.0025835	1
FGFRL1	-0.0569517	1
HDAC9	-0.0172446	1
FAM174A	0.0124643	1
TBC1D32	0.0291155	1
ITM2B	-0.0437816	1
AC139530.1	8.724E-05	1
ATP13A1	0.0367415	1
WDR59	0.0273629	1

NDUFA4	7.691E-05	1
MKNK1	-0.0768068	1
LRRC61	-0.0291097	1
C16orf70	0.0013677	1
ZNF263	0.0068574	1
PIK3R6	-0.0009033	1
RTL6	-0.0367877	1
MIGA1	-0.0136219	1
RELCH	-0.0414189	1
TMEM237	0.0149937	1
ZNF134	-0.0088014	1
ZNF7	-0.0182353	1
SLC25A17	0.0222095	1
PRKRIP1	-0.0297653	1
PIGO	-0.0031419	1
ZNF684	-0.0251098	1
ZNF548	0.0109171	1
HIST1H2BN	-0.0264125	1
GLIDR	0.0277409	1
DUSP28	-0.0131698	1
HSP90AA1	-0.0139616	1
DPCD	-0.023311	1
ZNF16	0.0251268	1
AC104530.1	-0.0145041	1
RAB11B-AS1	0.0223208	1
DDX5	-0.0828398	1
CASS4	-0.050509	1
MYH11	-0.0010018	1
ZNF200	-0.0028528	1
YME1L1	-0.0507455	1
CASTOR2	-0.0024529	1
FAM204A	-0.0259091	1
AC023157.3	-0.0179369	1
NRF1	-0.0399857	1
WDR53	0.0203787	1
SLC25A3	0.0114996	1
RPL23A	0.0034994	1
CACNA2D4	0.0245123	1
DNAJA1	0.0219227	1
TOR1B	-0.0301135	1
AMN1	-0.0170165	1
ZNF528	-0.0025721	1
RHNO1	0.006129	1
PPT2	-0.0093554	1
RDH13	-0.0368026	1
CTC1	-0.043713	1
ZNF710	-0.0247911	1
MARCH8	-0.0308886	1
AP3M1	-0.0169174	1
CIDEB	-0.0072848	1

AC009107.2	0.002991	1
ABR	-0.054382	1
MYO1B	0.0112774	1
RAB37	-0.0438951	1
RBBP7	0.0330628	1
AL355001.2	-0.0015328	1
BLOC1S3	-0.015981	1
SLC9A3-AS1	0.0087532	1
AP4M1	-0.0294492	1
DNAJC27	-0.0393691	1
AUH	0.0150959	1
RPL32	0.0154698	1
FKBP15	0.0092781	1
EGFL7	0.0050136	1
NOP53	-0.0049794	1
MGAT2	0.0025552	1
HACD1	-0.0090764	1
TPP2	0.0121802	1
LIPE-AS1	0.0007413	1
ACOT9	-0.0223561	1
BRCA2	-0.0545472	1
ZNF625	0.0325099	1
RRN3	-0.0034378	1
ATP5MC3	-0.0263011	1
LY6G5B	-0.0101094	1
C2CD3	0.0068192	1
TENT5C	0.0190866	1
MTG2	-0.008155	1
RETN	-0.0179444	1
TEPSIN	-0.0169325	1
ZBTB22	-0.0258598	1
APOBEC3F	0.0133533	1
NCF2	0.0049079	1
PITPNA	-0.0526986	1
SVIL	-0.0201341	1
CPPED1	-0.0191696	1
XRCC6	-0.0228077	1
ZNF570	-0.0187761	1
CKB	-0.0329647	1
HLA-DRB1	0.0242487	1
C2orf92	-0.0163823	1
CD38	-0.0554264	1
EXOC3-AS1	-0.0049029	1
SH3PXD2A	0.0348275	1
AL451165.2	-0.0133692	1
COA6-AS1	-0.0233127	1
RAB34	-0.0261253	1
ZNF689	0.0134457	1
ATG9A	-0.019768	1
GDPD1	-0.035996	1

ZFYVE27	-0.0218538	1
ZNF777	-0.008157	1
SRXN1	-0.0200053	1
AP4S1	-0.0233617	1
FAM161A	-0.0053526	1
TMEM217	-0.0154325	1
SYTL4	-0.0054823	1
RPS3	-0.0011641	1
PIM1	-0.0337384	1
IRF7	-0.0398186	1
TMEM106C	0.0197543	1
FAM118B	-0.0084889	1
HEXIM1	-0.0222382	1
CLDN15	0.0196072	1
ACSF2	-0.0077622	1
YPEL1	0.0304958	1
KIF3A	0.0103879	1
SMIM13	0.0019633	1
KIAA0753	-0.0122426	1
TMEM199	-0.0057841	1
RAPGEF2	-0.0085461	1
ZFYVE26	0.0250893	1
SAP25	0.00505	1
RNASEK	-0.0291694	1
NOL7	0.017464	1
AP5S1	-0.0190549	1
AKT1S1	-0.02929	1
MALAT1	-0.1744569	1
HSD17B7	-0.0316543	1
MT-ND4	-0.0212177	1
AC016773.1	0.002698	1
LRRC45	0.0259696	1
RPS4X	-0.009949	1
JAML	0.0479415	1
EEF1G	-0.0169826	1
MIF	0.0032917	1
HLA-F	-0.0349061	1

· cells after niche-like culture versus pre-culture (reference). Only genes expressed in ›

>10% of single cells are reported. Log2 Fold Change (FC) and FDR-adjusted p-value.

Supplementary Table 12. Differential gene expression averaged across 500 progenitor

Gene	log2FC	FDR q-value
RPS10	-1.302091	0
LTC4S	-1.2596337	6.15E-147
MTRNR2L12	-1.0286435	1.69E-142
SKIL	-0.7897849	1.37E-121
FOS	2.3310221	3.20E-108
MMP2	-0.8935289	8.46E-104
RPL38	-0.4763086	2.23E-100
CYTL1	-1.4671716	4.44E-94
ANPEP	-0.8510645	9.52E-92
CTSD	-0.8262585	3.23E-91
MT-CO3	0.5084068	1.53E-86
APOC2	-1.1104556	3.03E-85
SMAD7	-0.4491532	7.12E-79
TPSAB1	-1.8184889	2.48E-78
CNR2	-0.3453214	1.14E-72
IFITM2	0.7218679	5.29E-72
MT-ND4L	-0.7006551	8.93E-71
CD38	-0.6323199	9.59E-71
FOSB	1.3592234	1.53E-70
RPL31	-0.4051034	1.66E-69
IFITM10	-0.3474545	4.23E-69
MT-ATP8	-0.7056511	5.97E-69
MCL1	0.8803093	8.74E-67
CD52	1.052483	9.16E-67
AIF1	0.6842086	9.99E-66
RPL15	0.3263446	1.25E-65
IER2	0.9973649	4.61E-65
JUND	0.7140664	3.50E-63
CD99	0.8336662	5.86E-59
EIF1	0.4500772	6.93E-58
CD69	0.9147348	7.83E-58
SPINK2	0.927367	2.60E-56
RPS29	-0.3163295	3.03E-55
HOPX	0.7884328	2.79E-53
PIM1	0.7022934	1.62E-52
LY6E	0.6331989	2.15E-52
DUSP1	0.8848142	9.20E-51
XBP1	0.6969639	1.84E-50
MZB1	0.9098959	2.91E-50
TNFSF14	-0.3036764	1.27E-49
EGR1	0.9007287	3.58E-49
EGFL7	0.7167445	4.55E-49
JUNB	0.7837937	4.96E-49
MRC2	-0.3895307	5.77E-49
TMSB4X	-0.6526172	2.11E-48
ANKRD28	0.6787728	3.86E-48
RUNX3	-0.5716959	6.09E-48

SKI	-0.4276833	2.97E-47
HTRA3	-0.4036809	1.82E-45
CD96	-0.5355033	1.85E-45
GATD3A	-0.3562996	1.39E-44
NOTCH1	-0.365864	1.77E-44
WASHC1	-0.400955	4.76E-44
CST7	0.6276572	1.22E-43
RPS27	-0.2496889	1.71E-43
HCST	0.8012914	2.73E-43
RPS9	0.3049122	1.14E-42
RASGRP3	-0.5469849	1.32E-42
RPL37	-0.2762695	4.16E-42
JUN	1.1661832	4.93E-41
CTNND1	-0.3931089	5.25E-40
SVIL	0.5798918	7.83E-40
CYP1B1	-0.4011932	4.51E-39
SLC44A1	-0.6014042	9.58E-39
RALGAPA2	-0.3889502	9.85E-39
RPS20	-0.3273754	3.06E-38
DMTN	-0.1806791	4.73E-38
UQCR10	-0.4428398	7.55E-38
MT-ATP6	0.3161488	1.17E-37
STK17A	-0.3577566	2.15E-37
IRF2BP2	-0.4964152	4.83E-37
MPG	0.5191985	1.21E-36
CEBPA	-0.5603371	6.24E-36
PCBP2	-0.3805092	9.31E-36
PWWP3A	-0.3338207	1.29E-35
PLCG2	-0.6615587	2.01E-35
RPL39	-0.2771049	2.41E-35
DUSP6	0.8580076	2.91E-35
GMFG	0.4015139	6.02E-34
VNN1	0.4168944	8.98E-34
SNHG6	-0.3663112	1.25E-33
LPCAT2	-0.5650176	2.69E-33
KLF6	0.850318	2.99E-33
LAPTM5	-0.4923003	3.10E-33
FUCA1	-0.3094557	8.43E-33
FAM30A	0.8384089	1.16E-32
ST3GAL6	-0.3257263	1.92E-32
UNC5B	-0.2255954	3.59E-32
JAML	0.380947	3.86E-32
LACC1	-0.3479855	1.01E-31
MYL12B	-0.3346058	1.39E-31
NUDT1	0.3700079	2.05E-31
CAPG	-0.5451379	3.36E-31
ZFP36	0.5333229	4.88E-31
S100A11	-0.5090226	5.34E-31
NDUFV2	0.4058537	7.67E-31
RPL34	-0.2471415	1.78E-30

S100A9	0.2769292	2.56E-30
FAM43A	-0.2868003	8.34E-30
AHNAK	0.5298441	1.22E-29
AC020916.1	0.625496	1.98E-29
ITGA5	-0.3297787	2.45E-29
ORAI1	0.3661231	4.70E-29
CTR9	-0.4696261	1.22E-28
RPL3	0.2796377	1.28E-28
SOX4	-0.5910137	1.47E-28
NKG7	0.4554928	1.01E-27
ACP5	-0.2057354	1.41E-27
QSOX1	-0.2757676	1.49E-27
ANXA1	0.359836	1.53E-27
MAP1A	0.5266521	1.67E-27
LRRC75A	-0.7482679	2.02E-27
ORAI2	-0.3872229	2.02E-27
FTL	-0.4199557	4.20E-27
WASIR1	-0.1376473	1.58E-26
PLEC	-0.1750994	1.96E-26
RNASE3	-0.3405919	2.01E-26
RPL13	0.2487174	2.14E-26
RGS16	-0.196459	2.91E-26
NARF	-0.2822456	3.13E-26
TSTD1	0.3820451	3.78E-26
NEAT1	0.5366506	4.58E-26
PLXDC2	-0.4415582	5.00E-26
TUBA1A	0.7520331	5.28E-26
MEF2C	0.606914	5.31E-26
ADGRG1	0.3567618	6.11E-26
EMB	0.5103903	7.86E-26
LDLRAD4	-0.3563008	1.40E-25
ARL2	0.2455662	1.48E-25
SLC16A3	-0.3663122	1.71E-25
CDH26	-0.2232781	2.07E-25
CCNL1	0.4629778	2.18E-25
MAPKAPK3	0.3273738	2.76E-25
MGST1	-0.5530965	2.94E-25
FRY	-0.3619164	4.34E-25
ZMIZ1	-0.3846843	6.49E-25
IGLL1	0.814896	1.40E-24
RPL19	0.2247011	2.86E-24
RPL35A	-0.2091658	3.08E-24
NDRG1	-0.3574194	5.25E-24
RPL8	0.2304696	6.06E-24
SQSTM1	-0.3852285	6.71E-24
NCOA4	-0.4243465	7.52E-24
DENND11	-0.314829	8.33E-24
IFITM3	0.4564989	9.94E-24
ATP2B1-AS1	0.3683668	1.34E-23
TPM4	0.5234649	1.42E-23

TIPARP	-0.3928882	1.47E-23
IFI30	-0.4525803	1.52E-23
CTNNB1	-0.3042088	1.55E-23
JCHAIN	1.5353699	1.83E-23
MARCH3	-0.1187736	1.86E-23
AC104530.1	-0.1951295	2.50E-23
MTRNR2L8	-0.3461589	2.52E-23
MT-ND2	-0.3499185	3.77E-23
TUBB	0.4469286	4.63E-23
TXNIP	0.4803252	5.44E-23
TMEM173	0.3606606	5.46E-23
FRG1	-0.2995851	6.54E-23
KCNQ1OT1	-0.762586	4.85E-22
CDKN1C	-0.4937993	5.93E-22
CD82	-0.365545	7.10E-22
COBLL1	0.3824197	7.41E-22
SERPING1	0.2793951	1.27E-21
PLPPR3	-0.3876968	1.64E-21
ST8SIA6	-0.3593483	1.95E-21
STAG3	-0.1106931	2.22E-21
PPBP	0.6845586	2.36E-21
TSPO	0.3553046	2.94E-21
GLIPR1	0.6467331	3.05E-21
LSM7	0.3468464	3.53E-21
RPS15	0.2107642	3.82E-21
ACSM3	-0.6341883	3.97E-21
PABPC1	-0.3192366	4.06E-21
HNRNPH1	-0.3114405	5.56E-21
CALM1	0.4319423	6.45E-21
C3	-0.1042021	1.12E-20
RPL7A	0.193787	1.28E-20
CTSC	0.3567913	1.28E-20
ALDOA	-0.2735631	1.39E-20
RPL18A	0.2687117	1.46E-20
GOLGA8O	-0.150071	1.71E-20
GPR183	-0.280597	1.89E-20
SRGN	-0.7510084	2.11E-20
TNRC18	-0.3792412	2.26E-20
HLA-DPA1	0.4695747	2.27E-20
SQOR	-0.305633	2.92E-20
LCP1	0.4068664	3.07E-20
SPI1	-0.3483064	3.30E-20
FKBP2	-0.1541704	4.30E-20
DERL3	-0.120217	5.17E-20
FLT3	0.5003979	5.32E-20
TRG-AS1	0.5518501	7.68E-20
STAT1	-0.2127535	8.98E-20
AL627171.2	-0.3252219	9.82E-20
CDC42SE1	-0.2168871	1.12E-19
NCF2	0.2792875	1.19E-19

EVA1B	0.2884591	1.49E-19
LRPAP1	-0.4009159	1.54E-19
DNAJB6	-0.326222	1.60E-19
TIMP1	0.4599651	1.69E-19
MANF	0.3418061	2.28E-19
HMOX1	-0.1599619	2.57E-19
C1orf56	-0.2274538	3.12E-19
RPL41	-0.187038	5.24E-19
MAFG	-0.2339252	5.76E-19
GMCL1	-0.2869089	6.95E-19
INSIG1	-0.2475116	7.43E-19
PCNA	0.1110529	8.44E-19
TCF4	0.6238082	9.45E-19
AC244502.1	-0.5634916	9.94E-19
RPL29	0.2174122	1.20E-18
CD79B	0.228503	1.64E-18
ETV6	-0.2911886	1.97E-18
LINC00926	-0.1683429	3.76E-18
SNHG25	-0.2858995	3.85E-18
PLEKHO1	-0.3410686	4.07E-18
TUBB4B	0.337545	4.18E-18
NINJ1	-0.1365159	4.48E-18
VSIR	0.3501091	4.80E-18
PRDX4	0.2822175	5.22E-18
AC007952.4	0.4656429	7.48E-18
NPIPB5	-0.2007855	7.59E-18
PRSS21	0.2263506	7.97E-18
DUSP2	0.35104	8.00E-18
ENG	-0.1731154	8.85E-18
LAMP1	-0.2403496	9.13E-18
ITGAE	-0.2940815	1.80E-17
TGIF1	-0.1789215	2.64E-17
IER3	-0.2336853	3.13E-17
CFLAR	-0.3439727	4.11E-17
PTBP3	0.278529	4.22E-17
NEK6	-0.2277866	4.70E-17
RPS8	0.1931794	5.04E-17
VASH1	-0.1381793	5.89E-17
BIRC2	-0.1904758	8.83E-17
LAMP2	-0.3163452	9.54E-17
NCF1	-0.2273707	1.126E-16
CELF2	-0.4024448	1.134E-16
HOXA3	-0.2034465	1.295E-16
MYCN	-0.3358157	1.342E-16
NCBP3	-0.3376753	1.352E-16
COX6B1	-0.2967554	1.498E-16
PPP3CA	-0.2865251	1.624E-16
ARHGAP25	0.333781	1.651E-16
ERV3-1	-0.1966802	1.844E-16
LMNB1	-0.1848446	2.081E-16

SLC35E2A	-0.1607763	2.529E-16
CD36	0.5536571	2.664E-16
TPM3	0.3484828	2.995E-16
DOCK10	-0.2178194	3.582E-16
TNFRSF14	-0.1843571	3.867E-16
CBWD1	-0.1507259	4.412E-16
ZMAT5	-0.1765298	4.985E-16
RNASET2	0.2606837	5.833E-16
NPIPB4	-0.1441875	5.996E-16
EIF4EBP1	0.1890435	6.471E-16
CCZ1	-0.1580595	6.799E-16
CD300LF	-0.1646798	6.95E-16
PIGX	-0.1847703	7.853E-16
PPP1R14B	0.2968265	1.001E-15
SOCS3	0.2675555	1.045E-15
DDX3X	0.3791458	1.045E-15
MYH9	-0.3393477	1.379E-15
IFNGR2	-0.1882681	1.398E-15
AHRR	-0.076537	1.61E-15
RALGDS	-0.2345257	1.786E-15
NECAP2	0.1648629	1.999E-15
BTG2	0.4393784	2.225E-15
S100A4	0.4231044	2.473E-15
ASCL2	-0.1490001	2.889E-15
CD200	0.2332625	3.041E-15
FMNL3	-0.1064774	3.116E-15
FAM47E	0.1329739	3.205E-15
MRPL17	0.1132658	3.62E-15
SOCS2	0.250118	4.333E-15
FAM89B	-0.1463642	4.665E-15
EFHD2	0.1642815	4.916E-15
SLC1A4	-0.1331464	5.376E-15
GOLGA8N	-0.1908993	6.117E-15
XIRP2	0.1868162	7.082E-15
IL2RA	0.2809442	7.254E-15
GSN	0.2639728	7.866E-15
PTPMT1	-0.1802183	8.187E-15
JMJD6	-0.0654044	8.511E-15
MXRA7	-0.1067897	8.858E-15
LMO4	-0.332806	9.029E-15
SORL1	-0.4540184	9.093E-15
S100Z	0.3384833	1.099E-14
CDK2AP2	-0.0049336	1.101E-14
ENDOG	0.1348041	1.321E-14
SDK2	-0.0803753	1.468E-14
MRPL22	0.0098284	1.638E-14
HSPG2	-0.1261944	1.666E-14
FIS1	0.1886105	1.831E-14
GNA12	-0.2533327	1.841E-14
PFDN1	-0.1923755	1.912E-14

MCM7	0.0709788	2.193E-14
CHMP6	-0.1187378	2.213E-14
ZC3HAV1L	-0.012683	2.258E-14
SELPLG	0.1688187	2.27E-14
TSC22D4	0.0897853	2.278E-14
GOLGA8M	-0.081519	2.361E-14
RASSF5	-0.0939258	2.388E-14
CFD	-0.8444304	2.402E-14
RYBP	-0.2200897	2.474E-14
CNST	0.3054826	2.539E-14
AC068587.4	-0.1781234	2.627E-14
AC092069.1	-0.1515522	2.647E-14
SPON2	-0.1994588	2.657E-14
LST1	0.3999005	2.673E-14
GUCY1A1	0.3879527	3.602E-14
SASH3	-0.1343263	4.182E-14
AL355922.1	-0.4326243	4.207E-14
PPT1	-0.2152445	4.279E-14
MDK	0.3287352	4.298E-14
C1orf162	-0.2974923	4.347E-14
NTHL1	0.1624278	4.803E-14
CCND3	0.2796548	5.044E-14
H3F3A	0.2202523	5.444E-14
NRG4	-0.2326394	5.676E-14
RAB37	-0.247318	6.442E-14
PSIP1	0.2643474	6.556E-14
HCFC1R1	0.0465027	7.087E-14
H2AFV	-0.30004	8.477E-14
OSER1	-0.0950699	1.131E-13
EPDR1	0.1294751	1.333E-13
AC012615.1	-0.0796596	1.391E-13
PCBD1	0.2047361	1.433E-13
SV2A	0.1848974	1.46E-13
ISG15	0.2380961	1.479E-13
CD59	0.248164	1.488E-13
RRP1	0.0591881	1.636E-13
BCL2	-0.2517809	1.676E-13
GPR108	-0.087001	1.81E-13
BIN1	0.2657033	1.827E-13
MIR155HG	0.2603105	1.955E-13
ZNF69	-0.100148	2.036E-13
SAMD11	-0.0729203	2.318E-13
CHID1	0.1221488	2.506E-13
AC005670.3	-0.1406029	2.866E-13
TRABD	-0.1631998	2.921E-13
RPS21	-0.1549585	3.203E-13
COPRS	0.0039069	3.362E-13
PSMC5	0.1644002	3.383E-13
MIA2	-0.1389774	3.477E-13
ADAM28	-0.3225906	4.146E-13

BAHCC1	-0.2569026	4.516E-13
RPS5	0.1887831	4.574E-13
TMEM248	-0.059234	4.616E-13
GTF2A2	0.2179358	4.686E-13
FGR	-0.0671712	4.96E-13
NUTM2A-AS	-0.1811339	5.06E-13
TBC1D15	0.1902018	5.11E-13
NFYC	-0.0591318	5.275E-13
GPN1	-0.0013726	5.76E-13
KLF2	0.1852822	5.965E-13
KLHL24	-0.2288586	6.995E-13
WASHC4	-0.2112103	7.149E-13
GPR132	-0.0751555	8.801E-13
ANGPT1	0.4226399	9.613E-13
RGS10	-0.341642	1.021E-12
RPL27	-0.1314025	1.033E-12
CCDC85B	0.2597449	1.255E-12
FNIP1	-0.2559964	1.314E-12
S1PR4	0.2071479	1.525E-12
SH3GLB1	-0.2547152	1.533E-12
SNRPC	0.0357429	1.551E-12
PRR5L	0.1642802	1.602E-12
CKLF	0.344578	1.783E-12
GOLGA8R	-0.1700886	1.793E-12
PPM1F	0.1707337	1.818E-12
SNHG32	-0.2556628	1.832E-12
RASAL3	-0.1968484	2.002E-12
IFI16	0.3598838	2.015E-12
MAP3K2	-0.2492906	2.127E-12
R3HDM4	0.0388535	2.242E-12
KCNK17	0.1710131	2.279E-12
TNK2	-0.1358678	2.321E-12
PIK3CB	-0.2314715	2.453E-12
PROC	-0.1237447	2.548E-12
LINC02019	-0.072567	2.806E-12
EIF2S3	-0.2647072	3.251E-12
WDR45B	-0.2364368	3.322E-12
RAP2C	-0.094221	3.541E-12
RPS14	0.1797103	3.599E-12
TSPAN32	0.1899854	3.884E-12
H6PD	-0.1135829	3.977E-12
CTNNBL1	-0.0928327	3.997E-12
DOCK1	0.2788711	4.263E-12
DPP7	0.2784084	4.292E-12
RAB1A	0.0324823	4.309E-12
PIH1D1	0.0968886	4.405E-12
MRPL51	0.1584757	4.784E-12
DNM1L	-0.0340896	4.84E-12
DNMT3A	-0.1656202	5.009E-12
RTCB	0.0557173	5.017E-12

NABP1	0.2882355	5.065E-12
CNOT6	-0.0817129	5.184E-12
EVI2B	-0.2680471	5.282E-12
MXD1	-0.2354872	5.445E-12
APOE	-0.0804928	5.68E-12
FUT7	-0.198323	5.79E-12
MRPL11	0.1645004	5.922E-12
POLR2C	-0.0347984	5.938E-12
ERGIC1	0.2216881	6.601E-12
CAMKK2	-0.1578104	6.794E-12
GALNT2	-0.29549	7.165E-12
SEC22B	-0.1224902	7.314E-12
METTL26	0.2452815	7.611E-12
PGS1	-0.1374865	8.064E-12
MRPS34	0.1356576	8.367E-12
PHF5A	-0.1555758	8.562E-12
HADH	0.1130341	8.978E-12
RABL2B	-0.1384423	9.006E-12
LAIR1	-0.2819253	9.143E-12
VDAC3	0.1948623	1.003E-11
AC016773.1	-0.0304016	1.071E-11
ADAM17	-0.1669738	1.079E-11
SNRNP40	0.0108624	1.136E-11
CHMP4B	-0.1791358	1.221E-11
CENPJ	-0.1858481	1.241E-11
MAF1	0.0675938	1.262E-11
IL3RA	-0.2806939	1.345E-11
CBFB	-0.1799515	1.368E-11
NAA50	0.0647514	1.504E-11
CASP2	-0.169138	1.505E-11
AP001059.3	-0.036238	1.509E-11
ITGB1BP1	0.0964244	1.525E-11
PPP1R15A	0.3372759	1.586E-11
P2RY8	-0.280221	1.73E-11
RPLP2	-0.1331511	1.796E-11
TMEM38B	0.2049818	1.989E-11
FCHSD2	-0.1604359	2.013E-11
XYLT1	-0.1089647	2.017E-11
FLNA	-0.2676688	2.04E-11
GRK2	-0.19206	2.057E-11
MAZ	-0.2191414	2.085E-11
ASAH1	-0.3465755	2.178E-11
MAD2L1	0.0216777	2.214E-11
RNF144A	-0.1235993	2.241E-11
SMN1	-0.0763635	2.312E-11
IFT57	0.1797931	2.403E-11
CDKN2AIPN	0.003386	2.41E-11
EPB41L3	-0.0860926	2.417E-11
HPRT1	0.1691475	2.45E-11
TBCB	0.07697	2.453E-11

MCM4	0.1356051	2.482E-11
PALD1	-0.1062613	2.577E-11
ZMPSTE24	-0.0464131	2.644E-11
RPL37A	-0.1525646	2.709E-11
ATG12	-0.1878082	2.768E-11
FTH1	-0.4167102	2.769E-11
GNPAT	-0.031319	2.786E-11
MGST2	0.1975534	2.798E-11
AP1M1	-0.0302468	2.879E-11
C9orf139	-0.1225573	3.027E-11
FAM117A	-0.2569779	3.227E-11
MFSD14A	-0.0415777	3.234E-11
RNF166	-0.1970159	3.374E-11
ATP2C1	-0.2300208	3.401E-11
SNHG19	-0.1425944	3.489E-11
HDAC3	-0.0024655	3.635E-11
MRPL12	0.1292048	3.649E-11
TMEM41B	-0.0359716	3.699E-11
RPL28	0.1650596	3.715E-11
LINC01355	-0.1106723	3.826E-11
MZT2A	0.1993548	3.948E-11
AC026471.4	-0.08131	4.019E-11
ATF6B	-0.0949817	4.391E-11
MZT2B	0.2584169	4.471E-11
HLA-DPB1	0.377652	4.489E-11
GRK6	0.0861283	4.506E-11
ABHD10	-0.0154194	4.668E-11
MAP4K3-DT	0.1688346	4.725E-11
CRYGD	0.2449342	4.92E-11
PHB	0.1737567	5.101E-11
ZNF561	-0.0521731	5.848E-11
KANSL1L	-0.1321576	5.91E-11
HOXB2	-0.189898	5.925E-11
CISH	0.2219928	5.94E-11
UQCC2	0.0936188	6.012E-11
MGMT	0.1320801	6.111E-11
LNX2	-0.0753472	6.165E-11
F8A3	-0.0773401	6.619E-11
C16orf54	-0.1295431	7.221E-11
ZNF511	0.1095561	7.499E-11
SYNC	-0.1318239	7.757E-11
SMG1	-0.2627164	8.487E-11
C19orf25	0.0200602	8.72E-11
AL354733.3	-0.1236448	8.811E-11
JKAMP	-0.1271551	9.36E-11
GNA15	0.236392	9.531E-11
TNFAIP3	-0.1429959	9.54E-11
KCNAB2	-0.2514409	9.823E-11
TRIM2	-0.0710571	9.832E-11
CLCN7	-0.1190516	9.941E-11

DDX39A	-0.0329763	1.004E-10
STARD4-AS	-0.0959556	1.049E-10
LINC01409	-0.1271767	1.068E-10
MGAT2	-0.0430925	1.086E-10
RAB34	0.1195983	1.104E-10
CARD19	0.1250259	1.123E-10
DENND1B	-0.2175423	1.136E-10
PMAIP1	0.2287496	1.185E-10
RAD51C	0.0280999	1.203E-10
PHKG1	-0.1338548	1.208E-10
GYPC	0.2566142	1.251E-10
ATP1B3	0.1648291	1.302E-10
MRPL57	0.2098914	1.365E-10
BMP2K	-0.2409578	1.41E-10
REX1BD	0.1439276	1.503E-10
SLC40A1	-0.5697423	1.512E-10
SUCNR1	0.5186885	1.53E-10
YARS	0.0093584	1.551E-10
OSBPL8	-0.2285698	1.593E-10
CRLS1	-0.0590329	1.607E-10
IRF5	-0.0816682	1.614E-10
CCNC	-0.0865284	1.629E-10
PCMT1	-0.2007396	1.681E-10
ARPC5L	0.1218184	1.695E-10
SMIM7	-0.0044016	1.737E-10
AC092747.4	-0.0994376	1.754E-10
ZBTB8OS	-0.0273968	1.784E-10
ATXN2L	-0.0744365	1.886E-10
ZNF516	-0.1225425	1.937E-10
CYHR1	0.1133157	1.937E-10
NAA20	0.0618129	2.002E-10
MAPRE1	-0.0375628	2.003E-10
CBWD5	-0.1356451	2.026E-10
TMEM128	-0.0347998	2.143E-10
GBGT1	-0.0814358	2.18E-10
AHR	0.2435507	2.267E-10
SAMM50	0.0559037	2.271E-10
AC092171.5	-0.0510913	2.278E-10
HEXD	-0.127769	2.287E-10
NAXE	-0.0077871	2.368E-10
AC009107.2	-0.042643	2.372E-10
DHX8	-0.0361666	2.505E-10
TAB2	-0.0276109	2.521E-10
MRPS12	0.1761594	2.542E-10
ERAP1	-0.0908502	2.638E-10
HNRNPA1P4	-0.0688172	2.642E-10
HCFC1	-0.0392741	2.699E-10
ATP5MC1	0.2283858	2.732E-10
GNS	-0.0552616	2.791E-10
REXO2	0.1038988	2.822E-10

IGFBP7	0.1399365	2.992E-10
RPL22	-0.134685	3.156E-10
INTS11	0.05412	3.377E-10
RIOX1	-0.0145366	3.385E-10
PDLIM2	0.191538	3.483E-10
NDUFV1	0.0775657	3.582E-10
ZFAS1	-0.2307808	3.618E-10
AC010883.3	-0.1015171	3.667E-10
NPTN	-0.0961699	3.677E-10
C4orf48	0.2129872	3.749E-10
AC004918.1	-0.0644869	3.768E-10
PDHA1	0.0366741	3.824E-10
C14orf119	-0.0028663	4.076E-10
STON2	0.2298655	4.283E-10
C11orf54	-0.1290403	4.321E-10
ZNRF1	-0.0824968	4.388E-10
GPX4	-0.2151866	4.6E-10
SNX13	-0.0472195	4.701E-10
LINC01082	-0.0573672	4.738E-10
CCDC107	0.0944794	4.823E-10
PRRC2A	-0.0349413	4.846E-10
PARP1	0.227439	4.879E-10
FHOD1	-0.0712174	5.043E-10
EPG5	-0.0751554	5.057E-10
FAHD2A	-0.0933157	5.222E-10
YBX3	0.2758624	5.252E-10
AC083798.2	-0.061026	5.259E-10
FBRS	0.0221262	5.278E-10
ENC1	-0.0937777	5.387E-10
BHLHE40	-0.1813321	5.5E-10
CEBPB	-0.2558329	5.52E-10
MEX3B	-0.1408227	5.531E-10
RNASEH2C	0.2151591	5.726E-10
SCN1B	0.1500772	5.849E-10
NAGPA	-0.0074224	6.089E-10
TP53	-0.1248732	6.389E-10
MIS18BP1	-0.2482704	6.605E-10
MRPL21	0.0870001	6.89E-10
CD33	0.2041645	7.07E-10
FKBP5	0.1486799	7.077E-10
NDUFA13	0.1898155	7.079E-10
DGKQ	-0.0938029	7.276E-10
S100A6	0.2834033	7.294E-10
NME4	0.1332393	7.33E-10
PIK3R1	0.3401022	7.432E-10
GALK1	0.0740488	7.455E-10
MRPL36	0.1154424	7.537E-10
AC027601.6	-0.167914	7.574E-10
RPA3	0.0716352	7.608E-10
MCM5	0.0283737	7.791E-10

ELP5	0.0460659	7.858E-10
RNF41	-0.0658223	8.289E-10
RCC2	-0.1627037	8.315E-10
TBC1D1	-0.0766213	8.418E-10
VPS39	-0.0540924	8.459E-10
RNASE2	-0.4901644	8.914E-10
SRPRA	-0.0054479	8.966E-10
FAM107B	-0.2479031	9.124E-10
MIR222HG	-0.1072364	9.154E-10
NMRAL1	0.0944552	9.214E-10
PYCR2	0.0191021	9.246E-10
ARID2	-0.1008385	9.276E-10
HMGB1	-0.2204875	9.374E-10
CREBZF	-0.1852401	9.741E-10
GPCPD1	-0.1459218	9.774E-10
EPM2AIP1	-0.1849199	1.013E-09
PGAM1	-0.1753097	1.028E-09
RFFL	-0.1726813	1.035E-09
SACM1L	-0.0036459	1.038E-09
TEX264	0.1174939	1.051E-09
TRAPPC4	-0.0159173	1.061E-09
RAPGEF1	-0.0725706	1.062E-09
CLPP	0.089913	1.077E-09
SIPA1L3	-0.0532767	1.083E-09
SELENOK	-0.0927218	1.104E-09
PLEKHJ1	0.0723925	1.148E-09
ZADH2	-0.027445	1.153E-09
STK17B	0.2557637	1.157E-09
CALR	-0.317976	1.159E-09
PPP1R10	0.2172152	1.218E-09
AHSA1	0.0328327	1.231E-09
AC103591.3	0.2395834	1.248E-09
SLC38A10	-0.0941111	1.249E-09
MGRN1	-0.0787081	1.286E-09
TES	-0.1870162	1.29E-09
TP53INP1	-0.1382601	1.294E-09
PNP	0.2064108	1.325E-09
ARRDC2	-0.1436776	1.328E-09
ATP6AP1	-0.0967539	1.353E-09
MRPS23	0.02363	1.363E-09
CLASP1	-0.0702005	1.441E-09
PTTG1IP	-0.1837947	1.499E-09
AC008105.3	-0.0566904	1.511E-09
HLA-C	0.3422837	1.518E-09
TOP2B	-0.2232132	1.523E-09
JAGN1	0.1781782	1.535E-09
NPW	0.2602498	1.57E-09
TNFAIP8L1	-0.1063942	1.582E-09
AMN	0.1932466	1.584E-09
PDLIM7	-0.1131615	1.605E-09

TSPYL4	0.0730803	1.66E-09
THRA	0.0033867	1.683E-09
MRPS2	0.0610472	1.684E-09
RPL18	0.160031	1.691E-09
LMO7	-0.0959107	1.71E-09
NDUFAF3	0.1724371	1.767E-09
ARHGAP4	0.1332041	1.783E-09
CHP1	-0.1157611	1.816E-09
GRHPR	0.1768661	1.828E-09
MAN2C1	0.0063497	1.83E-09
WHAMM	-0.1479258	1.883E-09
DAPK1	0.2176739	1.963E-09
PINK1	-0.0257634	1.988E-09
DHX40	-0.095666	2.025E-09
RREB1	-0.2654752	2.063E-09
POLR2I	0.0501081	2.064E-09
AC007336.1	-0.1438052	2.089E-09
CCDC106	0.0185362	2.105E-09
MCM3AP	-0.101869	2.163E-09
HOMER3	-0.176811	2.165E-09
ACOX1	-0.0968797	2.203E-09
EXOSC8	-0.0087184	2.205E-09
AUH	-0.057758	2.327E-09
IDS	-0.2575738	2.368E-09
DEK	0.2399276	2.402E-09
UBAC1	0.0118162	2.404E-09
MRPL27	0.0977013	2.476E-09
SNHG30	-0.0210948	2.495E-09
POLE	0.1131226	2.593E-09
GNPTG	-0.1193797	2.615E-09
CPT1A	-0.2173597	2.685E-09
TTF2	0.0382759	2.757E-09
MRPS28	0.0382479	2.759E-09
SLC39A1	-0.0042534	2.789E-09
IL9R	-0.0563218	2.796E-09
MATR3.1	-0.1040915	2.808E-09
DYNC1H1	-0.154825	2.815E-09
SIVA1	0.1204859	2.842E-09
DAB2	0.1747161	2.855E-09
DCTPP1	0.1379289	2.889E-09
SPARC	0.1939064	2.906E-09
RIPOR2	0.2198999	2.919E-09
MYCBP	0.0012544	2.982E-09
TXLNA	0.0331003	3.055E-09
OSGEP	0.0918703	3.06E-09
CNPPD1	0.0025603	3.14E-09
LAMTOR3	-0.0987105	3.19E-09
COPG1	-0.0428374	3.209E-09
LRRC41	0.0313803	3.253E-09
EMC6	0.0637426	3.432E-09

AC004540.2	-0.1078675	3.436E-09
NUP153	-0.0389506	3.499E-09
TRIM16	0.1654935	3.59E-09
CYSLTR1	0.1828467	3.594E-09
LTB	0.4114259	3.597E-09
STN1	0.148099	3.635E-09
CLU	0.3503461	3.675E-09
INKA1	0.2320798	3.706E-09
RHEB	-0.0626741	3.725E-09
MRPS10	0.0269301	3.79E-09
ATAD2B	-0.0546925	3.808E-09
MANBA	-0.1834537	3.849E-09
ERG	0.2443304	3.864E-09
EIF5A	-0.0138809	3.887E-09
SNRPA1	0.0662618	3.893E-09
HERC2	-0.2337864	3.938E-09
SOCS1	0.2060252	3.946E-09
ALG3	0.0584395	3.965E-09
STAB1	-0.2170197	3.982E-09
POLR2H	0.0590383	4.081E-09
DHFR	0.0524044	4.101E-09
ZCCHC3	-0.0829044	4.205E-09
ATP2A3	-0.1819632	4.25E-09
NMT1	-0.1603326	4.295E-09
TMEM11	0.0429649	4.444E-09
MAPKAPK5-	0.0135859	4.481E-09
EFHC1	-0.0613755	4.619E-09
ANKRD33B	-0.0972472	4.624E-09
COX7A2L	-0.1633074	4.698E-09
PUF60	0.0088329	4.777E-09
UTP18	0.0916994	4.86E-09
CASTOR2	-0.0764282	4.886E-09
UBXN6	-0.0297615	4.888E-09
ATP8A1	-0.096486	4.913E-09
MYH11	-0.0641168	5.099E-09
CD7	-0.2183798	5.212E-09
HECTD1	-0.1889972	5.243E-09
C11orf74	0.1688341	5.277E-09
SMAD6	-0.0553449	5.338E-09
ZNF790-AS1	-0.0543111	5.359E-09
SUPT5H	-0.0294445	5.366E-09
DGCR6L	-0.0143318	5.376E-09
EMC7	-0.0079726	5.454E-09
RABGGTB	-0.0478048	5.591E-09
ARL17B	-0.0615626	5.61E-09
PPIH	0.0400922	5.611E-09
MTA2	-0.0213149	5.763E-09
PRPF19	0.0233246	5.773E-09
NIBAN1	-0.2757539	5.96E-09
ABT1	0.0260513	5.999E-09

SNRPD3	-0.2166177	6.174E-09
HAVCR2	-0.004877	6.196E-09
MPV17L	-0.0650481	6.308E-09
AL021368.2	-0.2062399	6.431E-09
RRP7A	0.1096047	6.493E-09
UGGT1	-0.0643878	6.553E-09
TTC24	0.127014	6.636E-09
GET1	0.0121976	6.664E-09
SDSL	0.1301911	6.788E-09
PAQR3	-0.0523306	6.799E-09
TRIM56	-0.273504	6.878E-09
PSENEN	-0.0575663	7.208E-09
SVIP	0.1835218	7.273E-09
HMG20A	-0.0016421	7.295E-09
GTF3C1	0.0499502	7.321E-09
AC025159.1	-0.0566746	7.405E-09
AP3M1	-0.0717536	7.501E-09
NRF1	-0.0352655	7.529E-09
PITHD1	0.0095025	7.543E-09
AKNA	-0.0651621	7.557E-09
SLC20A2	-0.1219398	7.644E-09
WASIR2	-0.054171	7.755E-09
NELFA	0.0217014	8.017E-09
MVB12A	0.0558628	8.124E-09
H2AFY	0.3317026	8.15E-09
DENND6B	-0.0893807	8.276E-09
C19orf48	0.1561335	8.276E-09
IGHM	0.4387597	8.311E-09
EEF1G	0.1677144	8.364E-09
PTCD3	-0.0437352	8.406E-09
PSMF1	0.0033622	8.438E-09
NFKBIL1	0.004693	8.501E-09
GOLIM4	-0.1777461	8.631E-09
MIGA1	-0.0203164	8.642E-09
AL390728.6	-0.1269917	8.916E-09
GADD45A	0.1902294	8.925E-09
NBDY	0.1511969	8.991E-09
USO1	-0.078269	9E-09
SP1	-0.1297917	9.061E-09
HLA-DMA	0.1948061	9.07E-09
EP300	-0.1961117	9.151E-09
NCAPD3	0.0433322	9.194E-09
GNB4	0.070165	9.333E-09
HM13	0.1136699	9.347E-09
AC044849.1	0.1531216	9.641E-09
COMMD9	-0.0921309	9.911E-09
METRNL	0.1533833	1.004E-08
RFX2	-0.1271533	1.042E-08
VWF	-0.2785079	1.044E-08
ZBTB44	-0.2088784	1.056E-08

ZNF680	-0.1184937	1.062E-08
JDP2	-0.0306969	1.07E-08
MBD1	-0.0349601	1.071E-08
ASXL1	0.037232	1.077E-08
BCKDK	0.0841164	1.079E-08
ZNF362	-0.0395874	1.095E-08
ZNF302	-0.1041302	1.098E-08
BST2	0.2451969	1.098E-08
HDDC3	0.1074273	1.114E-08
PNPO	0.0041787	1.116E-08
SS18	-0.1401979	1.13E-08
TMBIM6	-0.16548	1.13E-08
NSD1	-0.0896948	1.173E-08
ARRDC1	-0.0316095	1.198E-08
SH3BP1	-0.0388513	1.232E-08
DKC1	0.1069635	1.246E-08
TRAPPC11	-0.0746067	1.258E-08
E2F4	-0.027872	1.262E-08
RANGRF	-0.0225752	1.268E-08
IMPDH2	0.2569313	1.275E-08
CHROMR	-0.1942743	1.278E-08
CTU1	0.0369905	1.281E-08
RHOH	-0.1218307	1.301E-08
MRPL15	0.1152048	1.313E-08
RNPEPL1	-0.0593108	1.321E-08
CALCOCO1	-0.0461589	1.326E-08
AC100810.1	0.062505	1.331E-08
POLG2	-0.0871815	1.336E-08
ARL8A	-0.0445084	1.344E-08
FAM160B1	-0.0558634	1.364E-08
USP39	-0.0205826	1.371E-08
AL109811.2	-0.0354828	1.388E-08
SLC48A1	-0.1074681	1.424E-08
EMILIN2	0.1104282	1.438E-08
CCNG2	-0.1076563	1.471E-08
PPP4C	-0.022689	1.513E-08
MED27	-0.0005186	1.542E-08
MRPL44	0.0039607	1.635E-08
STIP1	0.0863922	1.645E-08
THOC1	-0.105218	1.681E-08
DNAJC30	0.0054041	1.709E-08
ATP6V0A2	-0.2143625	1.722E-08
CTBP1	-0.1800971	1.747E-08
SCAF1	-0.0121156	1.747E-08
ATG14	-0.084722	1.777E-08
RFXANK	0.0759018	1.792E-08
MAN2B2	-0.0913826	1.824E-08
ZMYND11	-0.0459502	1.925E-08
UBE2M	0.0769032	1.932E-08
LSM6	0.0702475	1.941E-08

PET100	-0.210741	1.947E-08
PNPLA8	-0.0141198	1.997E-08
PIP5K1A	-0.0229716	2.018E-08
TXN2	0.1660157	2.04E-08
ZBTB16	-0.2324251	2.065E-08
EBNA1BP2	0.1061491	2.067E-08
NDE1	0.0630543	2.088E-08
FBXW2	-0.0529593	2.094E-08
ANKRD13D	-0.0480193	2.107E-08
AK5	0.1726388	2.111E-08
DMAC2	-0.0136966	2.19E-08
KHSRP	-0.0698441	2.23E-08
LINC00667	-0.0445028	2.231E-08
SCNM1	0.0059324	2.234E-08
SDHB	0.0048308	2.238E-08
CHMP1B	-0.1247534	2.24E-08
HAUS7	-0.0593245	2.248E-08
TOP1MT	-0.1781672	2.252E-08
MYDGF	0.1419247	2.26E-08
SESN1	0.059023	2.286E-08
URM1	0.0447626	2.289E-08
NCOA3	-0.1337994	2.308E-08
ADAM8	-0.159364	2.321E-08
LYN	0.1823152	2.33E-08
TRPS1	0.1255627	2.36E-08
SORT1	0.0922887	2.389E-08
EXOC4	-0.0416415	2.419E-08
NDUFA8	0.0727944	2.421E-08
PIN1	0.059182	2.462E-08
BSG	-0.1879481	2.463E-08
HOXA6	-0.0848901	2.485E-08
SIN3B	-0.0408382	2.555E-08
GORASP1	-0.0144283	2.566E-08
AC025171.4	-0.0512157	2.621E-08
ZC3H7B	-0.0520931	2.676E-08
RBBP4	-0.1555567	2.727E-08
TUBA1B	0.3531574	2.732E-08
CASS4	0.1391121	2.737E-08
FAM122B	-0.0692628	2.738E-08
HIST1H4J	-0.0618529	2.763E-08
ABTB1	-0.0351305	2.766E-08
MCM6	0.0974612	2.784E-08
TMCC1	-0.0588125	2.804E-08
MKRN2	-0.0108358	2.817E-08
BLVRB	0.1721294	2.866E-08
MRPL37	0.1111305	3.058E-08
DCTN5	-0.0508092	3.089E-08
GSK3B	-0.0669724	3.133E-08
TRIM27	-0.0974883	3.148E-08
AP5B1	-0.1445641	3.158E-08

GNL3	-0.0566817	3.176E-08
FMNL1	-0.1238736	3.21E-08
RMC1	-0.0191932	3.224E-08
NDUFAF8	0.0969059	3.241E-08
WDR26	-0.0593333	3.249E-08
UNKL	-0.1070651	3.26E-08
YIPF3	-0.0001599	3.303E-08
NGDN	-0.0379263	3.319E-08
RNFT1	-0.1183897	3.352E-08
CD244	-0.0845811	3.386E-08
RBM3	-0.2296399	3.396E-08
LAP3	0.035831	3.465E-08
PPM1A	-0.0551338	3.489E-08
CSF3R	-0.2797331	3.552E-08
UBQLN1	-0.0537359	3.563E-08
LINC00685	-0.0841128	3.622E-08
LSM4	0.1474047	3.662E-08
CNOT11	-0.0770736	3.668E-08
SIRT2	0.0147515	3.751E-08
TMEM50B	-0.0572494	3.754E-08
ATP1B1	0.2816336	3.798E-08
ZNF181	-0.0083584	3.918E-08
C22orf39	-0.034514	3.954E-08
ADAP1	-0.1126801	4.003E-08
PPP1R15B	0.0630301	4.086E-08
KRIT1	-0.0291021	4.097E-08
SYNE3	0.1366136	4.231E-08
ITGA4	-0.2457529	4.247E-08
PPIE	-0.0087379	4.292E-08
SF3B4	-0.0438487	4.311E-08
UBE2S	0.0991441	4.382E-08
RBM4B	-0.0177757	4.408E-08
UCP2	0.1161324	4.441E-08
CYB561A3	-0.0163264	4.493E-08
NDUFB8	0.1887625	4.52E-08
RPL10	0.1337422	4.68E-08
INTS8	-0.0050145	4.712E-08
AIF1L	0.1958465	4.746E-08
COTL1	-0.1026829	4.8E-08
TAGAP	-0.0549222	4.805E-08
GUK1	0.1032729	4.848E-08
LINC01226	-0.2401482	4.891E-08
ANKAR	-0.028201	4.921E-08
AC245297.3	-0.2385449	4.943E-08
NDUFA7	0.0983781	5.088E-08
AC138150.1	-0.0182444	5.149E-08
AC253572.2	0.1973721	5.156E-08
CD44	0.2924685	5.158E-08
RING1	-0.039142	5.196E-08
METTL5	0.1069521	5.238E-08

RNH1	0.1036112	5.243E-08
RPL30	-0.119456	5.302E-08
RBM12	-0.0076253	5.323E-08
MIB1	-0.1722744	5.359E-08
COQ5	0.0528074	5.529E-08
TXNRD1	-0.1715519	5.568E-08
SSNA1	0.1382799	5.603E-08
MTHFD1	0.0058851	5.617E-08
CBX3	-0.1949432	5.629E-08
GPAA1	0.0586807	5.664E-08
DTYMK	0.0618789	5.794E-08
CEBPG	-0.0387571	5.829E-08
SSH2	-0.0174897	5.867E-08
LPIN1	-0.054811	5.878E-08
ROBO3	0.1056499	5.904E-08
KLF4	0.0720463	6.035E-08
TNIP2	0.0528905	6.07E-08
NUP58	-0.0024828	6.075E-08
FAM192A	-0.0434167	6.183E-08
ZC3H18	0.0469083	6.524E-08
PCID2	0.0329826	6.547E-08
CDK4	0.1178492	6.557E-08
APAF1	-0.1068013	6.565E-08
CISD1	0.0753496	6.568E-08
SLC9A3R1	0.0147119	6.646E-08
DEDD2	-0.0197151	6.707E-08
DERA	0.0092456	6.854E-08
ZNF433-AS1	-0.0968599	6.869E-08
SIPA1	-0.1209655	6.95E-08
ZNF746	-0.0350471	6.961E-08
BUD31	0.0567683	7.008E-08
ARF5	-0.1160389	7.026E-08
ZNF66	-0.1282062	7.032E-08
TMEM245	-0.083206	7.11E-08
GABARAPL1	-0.0870651	7.111E-08
KCTD5	-0.0977611	7.155E-08
TUBA1C	0.1113822	7.157E-08
AC108863.2	-0.0407123	7.168E-08
CXorf38	0.1502764	7.231E-08
RCBTB1	-0.0912615	7.239E-08
TACC3	-0.0067794	7.295E-08
LDLRAD2	0.1688831	7.309E-08
FAM76B	-0.0500449	7.354E-08
USP4	-0.0417425	7.36E-08
ZNF714	-0.0895413	7.422E-08
ARL6IP6	-0.0311447	7.437E-08
UBR5-AS1	-0.181593	7.505E-08
RPP25L	0.0665627	7.616E-08
ZNF490	-0.076735	7.669E-08
YDJC	0.0657538	7.683E-08

REPIN1	-0.1270563	7.744E-08
ABHD14A	0.110904	7.814E-08
LETM1	-0.0266579	7.829E-08
GIGYF1	-0.0462758	8.001E-08
FAM41C	-0.058244	8.025E-08
SNX20	0.0111289	8.026E-08
FAM102A	-0.0817369	8.057E-08
AP2A1	-0.0159404	8.083E-08
ARID5A	-0.0894828	8.084E-08
CBLL1	-0.0656808	8.092E-08
DDIT4	-0.117069	8.102E-08
DPY19L4	-0.0755016	8.125E-08
COPS6	0.1143169	8.207E-08
THOC3	-0.0867363	8.228E-08
COASY	-0.0031846	8.275E-08
AKR1A1	0.0889291	8.311E-08
AC025040.1	-0.0953948	8.362E-08
SMG7	-0.0165706	8.479E-08
FAM207A	0.1074809	8.508E-08
DMAP1	-0.0112887	8.593E-08
C16orf74	-0.1317086	8.595E-08
DNAJC9	0.1090503	8.645E-08
TMED2	-0.0002608	8.833E-08
ATP8B4	-0.2847312	8.834E-08
OIP5-AS1	-0.2152811	8.914E-08
MAP7D3	0.0376656	8.943E-08
NPM3	0.0841785	9.042E-08
C20orf96	-0.0653508	9.233E-08
EXOSC5	0.1100052	9.239E-08
RAB4B	-0.0394966	9.268E-08
BOLA2B	-0.0874117	9.413E-08
GCN1	0.0118245	9.432E-08
PCMTD2	-0.0539404	9.599E-08
COPS7B	-0.013253	9.607E-08
GMNN	0.0683271	9.62E-08
GTF2IRD2B	-0.0592188	9.677E-08
EIF2D	-0.046949	9.746E-08
PPP1CA	0.094843	9.802E-08
HSBP1L1	0.1401698	9.842E-08
FRYL	-0.0600569	9.86E-08
MCM3	0.0840502	9.97E-08
PDAP1	0.0040911	1.019E-07
RETREG2	-0.0265733	1.022E-07
FANCA	-0.0368313	1.025E-07
NHLRC3	-0.1103793	1.027E-07
STUB1	0.1025452	1.033E-07
AC005921.2	-0.0210068	1.049E-07
SEMA4D	0.0212636	1.05E-07
GTF3C6	0.0149952	1.079E-07
COX15	-0.0214197	1.081E-07

MT-CYB	0.2007046	1.098E-07
SURF4	-0.0639182	1.105E-07
KLHDC3	0.0125087	1.106E-07
TGFBR2	0.0440504	1.108E-07
ADCY7	-0.0724249	1.115E-07
SPSB1	-0.0534612	1.123E-07
RABL2A	-0.0848208	1.126E-07
NSFL1C	0.0167907	1.128E-07
SEPTIN11	0.2052498	1.148E-07
STOML2	0.140233	1.159E-07
STRAP	0.0628001	1.167E-07
C1orf52	-0.008026	1.171E-07
FBH1	-0.0005168	1.18E-07
GNG5	-0.2032812	1.183E-07
SMURF2	-0.0979832	1.183E-07
NRROS	0.0009584	1.188E-07
NOP14	0.0486258	1.2E-07
NLRC3	-0.1062932	1.204E-07
MAML1	-0.0648239	1.222E-07
TMEM170A	0.0137054	1.229E-07
MDP1	0.0797756	1.235E-07
MYB	-0.2388324	1.24E-07
ARL6IP1	-0.2186989	1.246E-07
IFRD2	0.0618546	1.265E-07
TRIP4	-0.0902097	1.3E-07
GATAD2B	-0.1093067	1.311E-07
ALOX5AP	0.1564579	1.324E-07
RANBP1	0.1691126	1.326E-07
RBM23	-0.1048389	1.329E-07
MAPK6	-0.1379212	1.339E-07
BRD9	-0.011403	1.347E-07
ZNF687	-0.028384	1.357E-07
TBC1D2	-0.0660443	1.36E-07
ITPK1	-0.0964824	1.367E-07
RAMAC	-0.0668342	1.421E-07
MFSD14C	-0.0915784	1.434E-07
RPL10A	0.1298612	1.435E-07
POC5	-0.021914	1.446E-07
THYN1	0.0142208	1.449E-07
LINC01252	-0.0721084	1.459E-07
MAPRE2	0.0877865	1.481E-07
CS	-0.0258798	1.493E-07
C5orf51	-0.0100695	1.494E-07
KLF13	-0.1623485	1.501E-07
ARL6IP4	0.2208958	1.505E-07
POLDIP3	-0.0021049	1.521E-07
TRIM28	0.0084349	1.524E-07
APMAP	0.0228887	1.529E-07
RNF24	-0.2014135	1.531E-07
ICAM3	0.1426628	1.556E-07

MFSD1	-0.1627314	1.567E-07
PPP1R3E	-0.1465216	1.586E-07
RRP9	0.0205144	1.598E-07
FBXO22	-0.208723	1.616E-07
OTUB1	-0.0433509	1.619E-07
METTL15	-0.0694967	1.624E-07
FBXO11	-0.0339141	1.639E-07
ZNF593	0.0971247	1.654E-07
HIC1	-0.046698	1.668E-07
CERS5	-0.0147329	1.688E-07
PHPT1	0.1937178	1.703E-07
VRK3	0.0369409	1.711E-07
LZTFL1	-0.0106168	1.714E-07
EPB41	-0.170434	1.715E-07
RPS3	0.1493603	1.722E-07
COMMD7	0.1738964	1.722E-07
NCKAP1L	-0.1586934	1.724E-07
PGGT1B	-0.0829652	1.724E-07
NAGK	0.0005832	1.726E-07
BAALC	0.1836445	1.735E-07
ADH5	-0.0508037	1.741E-07
LSM14B	0.0531651	1.808E-07
MRPL32	0.0343633	1.811E-07
RPL36A	-0.1479175	1.814E-07
FUS	0.241027	1.835E-07
GPS2	-0.0930347	1.853E-07
NFXL1	0.0038828	1.857E-07
USP22	-0.0553896	1.861E-07
TOPBP1	-0.0558928	1.863E-07
BRWD1	-0.1472367	1.909E-07
SMC2	-0.1014245	1.925E-07
NUP42	-0.0405393	1.972E-07
GAB3	-0.0448204	1.974E-07
AC123768.4	-0.0334115	1.991E-07
PSMD3	0.0361597	2.001E-07
PSMG4	0.0706946	2.011E-07
WSB1	-0.2302978	2.014E-07
THAP12	0.0588587	2.058E-07
HECA	-0.0165051	2.095E-07
SPTLC1	-0.0442485	2.097E-07
XPO4	-0.0608666	2.105E-07
PKIG	-0.0976245	2.125E-07
KANSL1	-0.1123042	2.125E-07
ELP3	-0.027311	2.162E-07
MTMR9	0.0179754	2.179E-07
GOSR1	-0.0023919	2.186E-07
WDFY1	-0.1518652	2.193E-07
APOOL	-0.0388164	2.195E-07
CHMP3	-0.0656653	2.198E-07
SUDS3	0.0241125	2.199E-07

DHRS4	-0.0117222	2.204E-07
IST1	-0.1331661	2.21E-07
IREB2	-0.0302128	2.211E-07
FDX1	-0.0395184	2.213E-07
MRPL38	0.0671972	2.217E-07
PDCD5	0.1457626	2.225E-07
LRRK1	-0.110829	2.229E-07
DGKE	-0.1409421	2.237E-07
B4GALT5	-0.128412	2.244E-07
GAMT	0.1788257	2.318E-07
ANKRD49	-0.0332001	2.326E-07
SLC9A3R2	0.0998603	2.335E-07
ARL6IP5	0.3133017	2.35E-07
DUSP10	-0.1049045	2.354E-07
FHL3	-0.1441316	2.357E-07
NT5C3B	0.0553671	2.358E-07
LSG1	-0.1048506	2.368E-07
LIX1L	-0.0314581	2.371E-07
HSPD1	0.2171032	2.389E-07
BUD23	-0.0112556	2.391E-07
SERF2	0.2094794	2.405E-07
RRP36	0.0029068	2.425E-07
QTRT1	0.0209372	2.439E-07
ATP2B4	-0.0892099	2.447E-07
IVD	0.0194706	2.447E-07
AMD1	-0.1152559	2.455E-07
TSIX	-0.0636424	2.46E-07
TXN	0.241642	2.468E-07
MTOR	-0.0178018	2.543E-07
MRFAP1L1	-0.0420416	2.56E-07
RXRA	-0.051438	2.569E-07
MOSPD2	-0.072574	2.572E-07
TNFAIP8	-0.2897536	2.587E-07
LINC-PINT	0.1828828	2.589E-07
LSM1	0.0811247	2.611E-07
SERTAD2	0.1207597	2.64E-07
SNX19	-0.0234768	2.657E-07
GTF2I	-0.21472	2.725E-07
COPS5	-0.0380966	2.732E-07
NFAT5	-0.0686195	2.741E-07
CABIN1	-0.1570935	2.743E-07
ARHGEF2	-0.0512915	2.785E-07
TATDN3	-0.0061967	2.797E-07
ABHD2	-0.1934634	2.805E-07
ADGRE5	0.1840608	2.856E-07
CEP95	-0.0733556	2.874E-07
TMEM189	-0.0281625	2.883E-07
MAD2L2	0.0956885	2.922E-07
STMN1	0.0502528	2.925E-07
HYPK	-0.0546143	2.929E-07

PDHB	-0.0295156	2.933E-07
UBE2J2	0.0199635	2.946E-07
ERVK3-1	-0.037827	2.948E-07
NOMO1	-0.0724449	2.97E-07
CXCR4	-0.1585818	2.974E-07
SNU13	0.2139033	2.982E-07
CDC42SE2	-0.1031027	2.987E-07
MID1IP1	-0.0517875	3.029E-07
LTN1	-0.0657265	3.032E-07
DDX56	0.0281066	3.045E-07
TNFSF12	-0.0747116	3.057E-07
CYB5R4	-0.1296047	3.091E-07
HTATSF1	0.0585453	3.113E-07
RFC4	0.0116255	3.122E-07
FKBP8	0.0606184	3.136E-07
PRKAG1	-0.0196675	3.139E-07
CDK11B	-0.0856275	3.209E-07
DCPS	0.0302188	3.218E-07
SMIM24	-0.3152242	3.241E-07
CSF2RB	-0.3617779	3.265E-07
ICAM2	0.1166236	3.301E-07
NUDC	0.1091142	3.334E-07
CALM3	0.1081131	3.347E-07
HK1	0.0687501	3.375E-07
COQ8A	-0.0317264	3.425E-07
AC006480.2	-0.0869612	3.428E-07
B4GALT1	-0.0536929	3.431E-07
UBA1	-0.0518902	3.463E-07
MYPOP	-0.0384613	3.486E-07
PIKFYVE	-0.1404267	3.497E-07
ACADS	0.0422514	3.504E-07
METAP1	-0.0547646	3.531E-07
CEPT1	-0.0317551	3.55E-07
THEMIS2	0.0917307	3.58E-07
IRAK3	-0.1902257	3.593E-07
DPM2	0.0214201	3.625E-07
NT5C	0.1056397	3.665E-07
MED30	0.0120313	3.704E-07
ISCA2	-0.018519	3.75E-07
LMAN2	0.0765169	3.757E-07
TECR	0.0560374	3.775E-07
SETD2	-0.0803533	3.776E-07
LIMD2	-0.2215527	3.803E-07
RENBP	-0.0748856	3.893E-07
HLA-DRA	0.3094525	3.899E-07
CIZ1	-0.0981919	3.918E-07
MYSM1	-0.0993148	3.921E-07
SYK	0.0586072	3.973E-07
ZNF600	-0.0754331	4.069E-07
OPRL1	-0.0278	4.092E-07

DLGAP1-AS	0.1483398	4.103E-07
FBXL20	-0.1374638	4.109E-07
NUP43	-0.058982	4.14E-07
C20orf27	0.1199905	4.15E-07
RHOT1	-0.0906618	4.182E-07
POLD2	0.0529681	4.211E-07
ARPC4	-0.0500775	4.255E-07
TMEM91	0.0707099	4.26E-07
LMF2	0.0154761	4.412E-07
RPS18	0.1566307	4.422E-07
MAPKAPK2	-0.078264	4.422E-07
HSPA5	0.136402	4.433E-07
ZNF207	-0.1883804	4.443E-07
CYTH3	-0.0461055	4.473E-07
TMEM161B-	-0.2309644	4.491E-07
MRPS35	0.0127508	4.603E-07
ZNF431	-0.2793863	4.611E-07
TDRD3	-0.0368956	4.616E-07
API5	-0.0369823	4.675E-07
TIMM23	-0.0116043	4.683E-07
NOTCH2	-0.1141756	4.692E-07
POLE3	-0.1028351	4.729E-07
SERINC5	-0.1540734	4.761E-07
PHC2	-0.0188425	4.773E-07
SLC25A16	-0.0487159	4.815E-07
CCZ1B	-0.0784371	4.816E-07
CACNA2D4	-0.0192142	4.843E-07
SYNGR2	-0.152098	4.853E-07
NSL1	-0.0073361	4.882E-07
COMMD2	-0.0163772	4.885E-07
FAAP20	0.1312392	4.898E-07
CSNK1G3	0.0023889	4.9E-07
ARL5A	-0.0712772	4.931E-07
THUMPD3-A	-0.1652835	4.946E-07
GORASP2	-0.0235561	4.948E-07
CKS1B	0.1083075	4.964E-07
LRG1	-0.0823239	4.967E-07
B3GALNT2	-0.068868	4.971E-07
HACD3	0.1026867	5.005E-07
TAP1	0.0328244	5.089E-07
TMUB2	-0.0008233	5.109E-07
GPR27	-0.1486596	5.123E-07
DAZAP1	-0.105737	5.132E-07
CCT2	0.1476175	5.133E-07
GSTP1	0.2061933	5.144E-07
KPNA3	-0.102984	5.145E-07
NRBP1	-0.0518778	5.249E-07
SNRNP27	-0.0833262	5.329E-07
BLCAP	0.0170792	5.357E-07
NBPF12	-0.0598532	5.36E-07

RPS19BP1	0.0629224	5.368E-07
MEMO1	-0.09985	5.381E-07
ADAM15	0.0914904	5.402E-07
COQ4	0.0025189	5.483E-07
ZDHHC4	-0.0024711	5.511E-07
ELK3	-0.1838459	5.55E-07
LPCAT4	-0.031961	5.566E-07
ZNF384	-0.0584529	5.577E-07
RGS19	-0.1520002	5.578E-07
WNK1	-0.0472411	5.59E-07
SRP68	-0.028001	5.59E-07
ANAPC13	-0.0081567	5.606E-07
PLD3	0.0085478	5.736E-07
DCK	0.1409417	5.778E-07
DUSP14	0.0113992	5.846E-07
CNTROB	-0.0352951	5.864E-07
MAGOH	-0.0731699	5.881E-07
LPCAT3	0.0801349	5.885E-07
PYM1	0.0711241	5.941E-07
NELFB	-0.0188877	5.946E-07
STX5	0.029489	5.952E-07
TBC1D20	-0.048695	5.983E-07
NSUN5	-0.0889609	6.01E-07
VTI1A	-0.0387112	6.022E-07
TYK2	-0.0569294	6.136E-07
AP002748.4	-0.0319796	6.218E-07
BRAP	-0.0555825	6.236E-07
LRRC26	0.1530006	6.239E-07
PRKCB	0.1422275	6.277E-07
ZBTB4	-0.0604926	6.313E-07
ADIPOR1	0.000584	6.35E-07
MGAT4A	-0.0696086	6.414E-07
TMEM9B	0.0282207	6.533E-07
TUBE1	-0.0269674	6.555E-07
AUP1	0.0654995	6.557E-07
KANTR	-0.0466685	6.566E-07
MYADM	-0.0728614	6.571E-07
TPD52L2	-0.012224	6.625E-07
MRPL4	0.1267496	6.668E-07
SAFB	-0.0755979	6.676E-07
DCUN1D1	-0.0879583	6.704E-07
ERAP2	-0.0729947	6.717E-07
DLST	-0.0225678	6.739E-07
SUMO1	0.0561388	6.757E-07
BAIAP2-DT	-0.0170874	6.802E-07
SLC25A38	-0.058635	6.852E-07
RPRD1A	-0.077244	6.862E-07
PSMB8	0.2377193	6.894E-07
TCF12	0.033416	6.901E-07
LTV1	-0.0210872	6.971E-07

MLLT6	-0.0324639	7.042E-07
UBR2	-0.0350067	7.087E-07
ACO2	-0.0167047	7.136E-07
SNRNP25	0.0448381	7.145E-07
THAP9-AS1	-0.0581057	7.159E-07
PPP3R1	-0.0978414	7.24E-07
ATAD3C	-0.0406308	7.272E-07
LCP2	0.1097891	7.317E-07
PATL1	-0.0087761	7.329E-07
TRIM11	0.0056399	7.344E-07
GMPS	0.0964425	7.363E-07
NSF	-0.0351358	7.409E-07
RNF113A	0.0343694	7.457E-07
SSBP3	-0.0984101	7.502E-07
UMPS	0.0338111	7.508E-07
GTPBP6	0.0423322	7.559E-07
MRPL39	0.0024543	7.634E-07
SDHA	-0.0747589	7.642E-07
GLIPR2	0.1193838	7.655E-07
NUP50	-0.0951742	7.751E-07
PIGK	0.0230466	7.789E-07
VPS26C	0.0740305	7.815E-07
VPS51	0.0573925	7.907E-07
PIN4	-0.0527142	7.925E-07
KIAA1109	-0.1075452	8.004E-07
OCEL1	-0.0172405	8.024E-07
HPS6	-0.0105468	8.033E-07
ALDH6A1	-0.0457441	8.046E-07
SEC14L1	0.0568015	8.046E-07
VPS45	-0.0560211	8.065E-07
ALKBH5	-0.0326938	8.131E-07
SEC61A1	0.007694	8.239E-07
MFN1	-0.0448026	8.263E-07
TOR1AIP2	-0.0375617	8.291E-07
ATP13A2	-0.073594	8.354E-07
CSNK1D	-0.0554158	8.399E-07
WDR70	-0.1234665	8.447E-07
PRRG4	-0.1683063	8.513E-07
SLC25A39	0.0281406	8.595E-07
SUSD6	-0.0387538	8.779E-07
FLOT1	-0.1419002	8.899E-07
CDK7	-0.0559	8.957E-07
TST	-0.0306013	8.959E-07
SWAP70	-0.2097583	8.985E-07
IRAK4	-0.0904672	9.012E-07
SH3TC1	0.0110512	9.024E-07
VKORC1	0.1392761	9.067E-07
RASGRP2	-0.1125062	9.197E-07
CETN3	-0.0498573	9.235E-07
MYO15B	-0.0123348	9.34E-07

MTCH1	0.0175141	9.383E-07
AC099518.5	-0.0493337	9.567E-07
PMVK	0.0446733	9.628E-07
VPS25	0.0556628	9.64E-07
GATA2	-0.4387877	9.679E-07
RAB2B	-0.0010215	9.685E-07
MTMR3	-0.0965232	9.704E-07
PSMD2	0.0095332	9.705E-07
NME1	0.2104088	9.716E-07
AC091607.2	-0.0817612	9.72E-07
MIEF1	-0.0282395	9.806E-07
PADI2	-0.0560183	9.833E-07
XPNPEP3	-0.037467	9.835E-07
NSMAF	-0.0871807	9.888E-07
ACTR1A	-0.0307119	9.902E-07
NFKBIE	-0.0566401	9.909E-07
GNG7	-0.0267251	9.95E-07
HIST1H2AC	-0.1957629	1.001E-06
RO60	-0.0696856	1.004E-06
SNX17	0.071189	1.005E-06
ZNF260	-0.0351879	1.009E-06
TMEM33	-0.0715114	1.019E-06
UVRAG	0.025949	1.049E-06
PSMD14	0.009466	1.054E-06
TM2D3	0.0206234	1.066E-06
TMBIM1	-0.1252383	1.067E-06
PSEN1	-0.1159492	1.071E-06
LINC01410	-0.0429411	1.073E-06
GLB1	-0.0527358	1.089E-06
CAB39	-0.0493733	1.091E-06
SAR1A	-0.0439435	1.094E-06
HDAC9	0.0918416	1.094E-06
CSNK1G2	-0.0706072	1.096E-06
CHST14	-0.0241429	1.098E-06
GOLPH3	0.0657256	1.103E-06
AC005332.6	-0.062158	1.106E-06
MBTPS2	-0.0042585	1.113E-06
UBE2Q2	-0.1286588	1.115E-06
MRPL33	-0.244708	1.117E-06
TOR1A	-0.0915482	1.122E-06
KDM1A	0.0045189	1.123E-06
INO80D	-0.113491	1.126E-06
CBX4	0.0215202	1.128E-06
SYF2	-0.2016889	1.13E-06
MRPL9	0.0561121	1.138E-06
NOC4L	0.0194744	1.147E-06
RMDN3	-0.0185573	1.162E-06
HIGD1A	-0.017906	1.175E-06
LINC02035	-0.0297828	1.176E-06
TSFM	0.016932	1.185E-06

ARFGAP2	0.0442786	1.195E-06
CCT6A	0.1094218	1.2E-06
APTX	-0.0400755	1.222E-06
IGFLR1	-0.0751766	1.226E-06
ETFB	0.1688663	1.227E-06
TOMM70	0.0219548	1.231E-06
ZNF524	-0.0101949	1.235E-06
ZNF84	-0.0839336	1.237E-06
AC009950.1	-0.0316834	1.252E-06
ARF4	-0.1299099	1.254E-06
AFMID	-0.0849771	1.255E-06
FN1	0.107967	1.258E-06
LINC01089	-0.0470961	1.262E-06
UHRF2	-0.0296163	1.263E-06
AL592183.1	-0.1123065	1.267E-06
MAPKAPK5	-0.0123317	1.271E-06
SCAMP3	-0.0212472	1.287E-06
MPC2	0.105206	1.291E-06
UTP11	-0.0741706	1.295E-06
SLC35E2B	-0.0938962	1.298E-06
BCL7C	0.0688798	1.305E-06
ALKBH3	0.0050825	1.311E-06
CYLD	-0.1255801	1.313E-06
MCEE	-0.0265516	1.316E-06
PPP4R1	-0.0435932	1.318E-06
PSMB9	0.1628379	1.321E-06
PDLIM1	0.232029	1.324E-06
KIAA2026	-0.1111938	1.326E-06
AC103769.1	-0.0416938	1.334E-06
RBCK1	0.0909387	1.339E-06
ITGAL	0.2281817	1.367E-06
ALG8	0.0095889	1.37E-06
KDM4A	-0.1842116	1.373E-06
SYMPK	0.004828	1.378E-06
ZNF322	-0.0375866	1.379E-06
CFL2	-0.0777384	1.389E-06
MRPL54	0.0948745	1.395E-06
DBNL	-0.0535309	1.429E-06
STX10	0.0841048	1.429E-06
RTN4R	0.0697439	1.433E-06
TMEM97	0.0172324	1.434E-06
PPP1R35	0.0022915	1.446E-06
MRPS26	0.0594201	1.458E-06
AZU1	-0.5717209	1.483E-06
C1GALT1	0.0303372	1.484E-06
BCL7B	-0.0341882	1.488E-06
BCL2L13	-0.0568646	1.489E-06
FAM102B	-0.0691367	1.498E-06
TFG	-0.0156763	1.504E-06
MED12	-0.0198198	1.507E-06

OSTF1	-0.0695124	1.508E-06
MRPL47	0.0038865	1.514E-06
XAB2	-0.0301036	1.514E-06
PRPS2	-0.0034981	1.516E-06
ARHGEF40	-0.1022121	1.529E-06
YAF2	-0.0918388	1.532E-06
CSNK2A2	-0.0389209	1.536E-06
LENG1	0.0308026	1.538E-06
MEX3C	0.039537	1.553E-06
DDA1	0.0213275	1.558E-06
JOSD1	-0.0175636	1.575E-06
SNCA	0.0583107	1.578E-06
RBM4	0.0318624	1.58E-06
WBP2	-0.0549668	1.595E-06
RNF145	-0.0752171	1.612E-06
RBM18	-0.0217983	1.622E-06
SDHC	-0.1420607	1.64E-06
JPX	-0.213248	1.645E-06
TMEM144	0.0142098	1.648E-06
GRSF1	0.0710447	1.652E-06
ZNF701	-0.0297579	1.665E-06
UQCC3	0.0299967	1.666E-06
EMD	0.0265642	1.668E-06
RBBP8	0.0046952	1.674E-06
ARHGAP18	0.1721063	1.689E-06
ZC3HC1	-0.0072127	1.705E-06
MKNK1	-0.023101	1.713E-06
PRRC2B	-0.0983661	1.718E-06
BLOC1S2	-0.0059342	1.719E-06
TMEM199	-0.0280278	1.729E-06
LYRM1	0.1523212	1.735E-06
PRRC1	-0.0632839	1.742E-06
SLC39A11	-0.0808387	1.744E-06
SLC35A1	-0.0822853	1.751E-06
ZNF224	0.0001238	1.751E-06
GPS1	0.0062428	1.752E-06
CZIB	0.0219544	1.757E-06
VMAC	0.0068621	1.783E-06
AGL	-0.0888336	1.783E-06
SARS	-0.0343564	1.81E-06
ORC3	-0.0006098	1.82E-06
MCRIP2	0.0669586	1.829E-06
CIAO2B	0.0779931	1.83E-06
LINS1	-0.0482315	1.837E-06
RAD23B	-0.0587333	1.849E-06
SUPT7L	0.0277179	1.88E-06
ATMIN	-0.0647341	1.893E-06
COX19	-0.0044901	1.897E-06
C6orf89	-0.0055481	1.904E-06
SURF6	-0.0787368	1.905E-06

COMMD1	-0.0443502	1.906E-06
KMT2C	-0.1051714	1.912E-06
SLC6A6	-0.1300918	1.932E-06
EDEM1	-0.0540873	1.941E-06
COA3	0.0320635	1.949E-06
SENP5	-0.0473812	1.95E-06
HELB	-0.0972549	1.954E-06
SLC2A4RG	-0.0070181	1.954E-06
NDUFAB1	0.1026105	1.959E-06
GPR35	-0.1321029	1.995E-06
CLK3	-0.0529089	1.997E-06
CNIH1	-0.1018955	2.002E-06
NELFCD	-0.0412647	2.005E-06
LPXN	-0.1803372	2.007E-06
UGP2	0.1404367	2.013E-06
YIF1A	0.0768526	2.059E-06
LRRC59	0.0559709	2.075E-06
LRRC28	-0.0346449	2.078E-06
MYLPF	-0.1435587	2.085E-06
LRRC47	-0.0286127	2.109E-06
TRAK2	0.1389893	2.119E-06
CDC26	0.0636686	2.123E-06
MRPL28	0.0119907	2.126E-06
DCAF6	-0.0187605	2.136E-06
LZTS2	0.0305343	2.138E-06
SF3B3	0.0157445	2.14E-06
ADPRHL2	0.0058727	2.147E-06
TXNDC9	0.0331377	2.158E-06
HSP90AA1	0.2434898	2.161E-06
SNX15	-0.0536909	2.193E-06
TRIM65	-0.0222889	2.196E-06
EMID1	-0.0232602	2.198E-06
ESYT2	-0.0141028	2.216E-06
RNF141	0.0175779	2.245E-06
ABCA7	-0.0265613	2.264E-06
PTPN1	0.1009642	2.284E-06
TAF4	-0.0041007	2.295E-06
NMD3	0.0032277	2.317E-06
CSKMT	-0.1525926	2.32E-06
CLEC2D	-0.0686343	2.325E-06
NUP54	-0.0031998	2.339E-06
PTOV1	-0.0363212	2.365E-06
DELE1	-0.0510734	2.395E-06
PGRMC1	0.0018199	2.404E-06
CPSF3	-0.0092007	2.408E-06
ALOX12-AS1	-0.053929	2.416E-06
LEMD3	0.0184587	2.436E-06
PPP1R12B	-0.0938919	2.438E-06
TIMM44	-0.0157767	2.442E-06
PARP10	-0.0308275	2.449E-06

RNF10	-0.1193101	2.455E-06
CTSB	-0.1197079	2.458E-06
LYPLA2	0.0214719	2.47E-06
CDC123	0.0289083	2.497E-06
HSD17B4	-0.2252442	2.504E-06
MRPS16	0.0966913	2.511E-06
PECAM1	-0.0348874	2.525E-06
CYTOR	0.235999	2.532E-06
PIP4P2	0.1189306	2.544E-06
WDSUB1	-0.0868613	2.546E-06
DENND5A	-0.052395	2.55E-06
KIAA2013	-0.0331998	2.567E-06
SMN2	-0.0346699	2.568E-06
ZSCAN18	0.016478	2.603E-06
PSMG1	0.0622639	2.612E-06
USP7	-0.0019714	2.623E-06
TMEM273	0.1383998	2.641E-06
AL161457.2	-0.0537224	2.643E-06
PLEKHM1	0.0091864	2.643E-06
HTT	-0.1302892	2.645E-06
RAB11B	-0.0606378	2.645E-06
GIT1	-0.0167954	2.649E-06
MRPL41	0.0899598	2.656E-06
TYMS	0.258484	2.657E-06
PPP1R9B	-0.0230007	2.663E-06
TCTN3	-0.0183734	2.666E-06
MPPE1	-0.0949514	2.673E-06
NAAA	0.0949926	2.674E-06
VPS37A	-0.0584236	2.678E-06
SNHG29	-0.1854293	2.682E-06
RIDA	0.016417	2.703E-06
PTBP1	-0.0750909	2.714E-06
SLC66A3	-0.0024719	2.719E-06
AAK1	-0.2109717	2.74E-06
ANKRD40	-0.0044118	2.778E-06
BABAM2	0.0106822	2.813E-06
N4BP2L2	-0.1971864	2.815E-06
NLRP2	0.0080272	2.875E-06
STIM2	-0.0169856	2.881E-06
MICU1	0.0019067	2.882E-06
CREBBP	-0.0279425	2.888E-06
PPIL3	-0.0094776	2.893E-06
AZI2	-0.0985825	2.9E-06
MED13L	-0.22078	2.922E-06
ABI1	-0.0665535	3.009E-06
EFCAB2	0.0794612	3.009E-06
AC003681.1	-0.0549486	3.014E-06
NCALD	-0.0366504	3.034E-06
RNF181	-0.0073221	3.055E-06
NUDCD2	-0.0098249	3.071E-06

RNF11	-0.0425624	3.086E-06
PIGF	0.0437193	3.094E-06
SLC25A33	-0.0134615	3.096E-06
C11orf98	-0.0442506	3.098E-06
PRR7	0.0751249	3.1E-06
SOAT1	-0.1138868	3.114E-06
TMEM107	0.1520079	3.158E-06
MSRB1	0.0252324	3.179E-06
TCEA2	0.1177982	3.225E-06
NUMB	-0.0510907	3.237E-06
STAT6	-0.1016649	3.249E-06
TRIAP1	0.0322641	3.277E-06
MMP24OS	-0.0954163	3.303E-06
AGK	-0.0235666	3.311E-06
PNPLA4	0.0295554	3.326E-06
KCNMB3	-0.0348864	3.339E-06
PACS1	-0.0256688	3.344E-06
GALNT1	-0.1357016	3.353E-06
POLDIP2	0.0062671	3.354E-06
EFTUD2	-0.0052525	3.375E-06
NOM1	-0.0110719	3.376E-06
ZNF888	-0.029689	3.396E-06
EIF4E2	-0.0127009	3.413E-06
SLC25A43	-0.0115788	3.425E-06
TMEM80	-0.0184835	3.45E-06
GRAMD4	-0.1022268	3.451E-06
PIM2	0.1556415	3.464E-06
DNAJA2	-0.0495247	3.486E-06
ZNF587	-0.0657612	3.487E-06
RAB9A	-0.1638212	3.49E-06
NIPSNAP3A	-0.092457	3.491E-06
LAPTM4A	-0.1176945	3.492E-06
ZNF514	-0.0107656	3.507E-06
BSPRY	0.1634455	3.511E-06
NSUN2	0.0038535	3.52E-06
DMAC1	0.0672911	3.564E-06
DLD	-0.0129247	3.574E-06
PYCARD	0.2032047	3.612E-06
EDC4	-0.0079807	3.621E-06
FUOM	0.1086322	3.622E-06
PSMD12	0.0241078	3.641E-06
HSD17B7	-0.072162	3.647E-06
DNAJC24	-0.068465	3.652E-06
GET3	0.0151238	3.673E-06
RPS24	-0.123491	3.673E-06
OPA3	-0.0203796	3.739E-06
CCDC66	-0.0493814	3.745E-06
TIMM17A	0.012865	3.803E-06
ACADVL	0.0447367	3.811E-06
ZDHHC21	-0.108711	3.839E-06

POGZ	-0.079364	3.845E-06
NAA25	-0.0002828	3.846E-06
HK2	-0.0410923	3.85E-06
HIST1H4E	-0.0634502	3.873E-06
PHYKPL	0.0534679	3.908E-06
MAP4K3	-0.0039655	3.946E-06
PXMP2	0.0337002	3.958E-06
HOXA7	0.0555426	3.959E-06
POLD4	-0.1005328	3.963E-06
C1orf50	0.0138989	3.974E-06
MLLT1	-0.1385426	3.988E-06
DIP2A	-0.0049714	4.002E-06
RILP	0.0772639	4.033E-06
AC005261.1	-0.0986034	4.056E-06
ZNF350	-0.0345077	4.062E-06
YTHDF1	-0.0287838	4.086E-06
WDR36	-0.0179801	4.095E-06
TSEN34	0.1489107	4.106E-06
DGKD	0.0904243	4.109E-06
GPN3	0.129248	4.11E-06
CCDC28A	-0.0580265	4.147E-06
TXNDC17	0.1045905	4.182E-06
RANBP6	-0.026369	4.194E-06
FAM149B1	-0.0027638	4.198E-06
TTC7A	-0.0425218	4.207E-06
CCNDBP1	-0.084455	4.213E-06
CDK13	-0.1311079	4.219E-06
GCA	0.0347851	4.219E-06
POM121	-0.0538497	4.23E-06
DENND10	0.0188968	4.24E-06
MBD4	-0.1598399	4.27E-06
RFX8	-0.0654928	4.285E-06
UAP1	0.0010554	4.305E-06
FBXO28	-0.0116669	4.308E-06
ZCCHC17	0.0470448	4.309E-06
FAM216A	0.1251488	4.323E-06
ZZEF1	-0.0500161	4.336E-06
MCUR1	-0.0231824	4.355E-06
DDIT3	-0.0857645	4.379E-06
TRA2A	-0.1498125	4.388E-06
SLC35F5	-0.0935822	4.4E-06
NAA30	-0.0224299	4.416E-06
NFX1	0.0523753	4.438E-06
SLC30A5	-0.0287637	4.439E-06
BANF1	0.1261672	4.441E-06
RBM10	0.0134883	4.448E-06
ZNF124	-0.0439815	4.455E-06
LCMT1	0.0046702	4.463E-06
SPN	-0.239577	4.471E-06
C11orf71	-0.0124625	4.498E-06

TBRG4	0.0451677	4.517E-06
ATF2	-0.0539593	4.529E-06
NFKBIZ	0.2084673	4.538E-06
ITFG2	-0.0358033	4.541E-06
NDUFB10	0.1200207	4.545E-06
PSMD9	0.0470574	4.598E-06
AGO2	0.0059896	4.632E-06
SPIN4	-0.1819046	4.633E-06
C6orf62	-0.1774941	4.646E-06
MON1B	-0.0557105	4.664E-06
NRAS	-0.0486732	4.714E-06
GRAMD1A	0.0851441	4.759E-06
MSL2	-0.0743542	4.795E-06
AC011043.1	-0.0537894	4.801E-06
ANKRD10-IT	-0.1047874	4.804E-06
NFATC3	-0.0468186	4.829E-06
SGO2	-0.0892805	4.844E-06
TRAPPC2	-0.0356588	4.845E-06
DDX49	0.0460061	4.852E-06
COA4	0.1165758	4.885E-06
FLYWCH2	0.0377729	4.903E-06
TOMM40	0.0838993	4.917E-06
MCOLN1	0.0226481	4.943E-06
PCLAF	0.3799391	4.943E-06
GCLC	-0.0841634	4.993E-06
INAFM2	-0.0631877	5.01E-06
PARP14	0.12386	5.019E-06
TSR1	0.0668182	5.096E-06
PTGES2	0.021083	5.103E-06
ARL5B	0.0090533	5.158E-06
TNFRSF25	0.0977109	5.161E-06
FAM111A	-0.0660031	5.17E-06
GTF2H3	0.0156926	5.268E-06
TSG101	-0.0280989	5.306E-06
CNOT1	-0.0722335	5.324E-06
PRKCH	0.0942805	5.369E-06
ZNF506	-0.130563	5.374E-06
DPH7	0.0032432	5.376E-06
ZKSCAN8	-0.0564488	5.406E-06
BRMS1	-0.0283736	5.442E-06
DNMT1	-0.0206111	5.458E-06
MRPS18B	0.0574813	5.476E-06
PPP2R2D	-0.0085818	5.494E-06
FGFRL1	-0.0911354	5.552E-06
ZNF875	-0.0269941	5.555E-06
RBPMS	0.143091	5.581E-06
C9orf78	-0.0887089	5.585E-06
EIF3B	0.0735835	5.598E-06
VPS53	-0.0398702	5.614E-06
VPS8	-0.0265033	5.631E-06

SRP9	-0.1065078	5.659E-06
NUP160	-0.0598849	5.668E-06
CD37	0.2179339	5.695E-06
C15orf61	0.0361496	5.723E-06
AL450998.2	-0.0332994	5.724E-06
RUFY3	0.0259096	5.77E-06
TSSC4	0.0588246	5.771E-06
FOXO3	0.0415236	5.811E-06
SPAG9	0.0172211	5.821E-06
HIST1H3D	0.0292217	5.829E-06
SNHG7	-0.2779194	5.845E-06
FCER1G	0.182643	5.853E-06
UNC119	0.0026775	5.887E-06
UNG	0.0861025	5.974E-06
AC093673.1	0.0128773	6.005E-06
DDX41	-0.02663	6.019E-06
ADI1	-0.1651105	6.061E-06
AKT1	0.0678473	6.075E-06
NUDT4	-0.1036887	6.106E-06
KRT8	0.0134751	6.108E-06
MRPL40	0.0901243	6.109E-06
ATAD1	-0.0330499	6.183E-06
LINC01268	-0.187155	6.184E-06
ITPR2	-0.1164934	6.252E-06
GUSB	0.0677081	6.269E-06
SUN2	-0.1124602	6.295E-06
TMEM251	-0.0550387	6.38E-06
QKI	-0.1787863	6.384E-06
TMEM203	0.0047613	6.39E-06
USP33	-0.1015957	6.407E-06
WDR81	-0.047645	6.418E-06
MRPS11	-0.0140586	6.482E-06
TTL	-0.045662	6.499E-06
AAMP	0.0004353	6.503E-06
LYST	-0.2583423	6.518E-06
PARG	-0.0014154	6.544E-06
FOXK1	-0.0510137	6.587E-06
NUP210	-0.0050271	6.626E-06
ATP13A3	-0.0844968	6.661E-06
NPDC1	0.1722362	6.688E-06
GNL3L	-0.0596781	6.695E-06
TM9SF4	0.0015383	6.716E-06
NSMCE1	0.1018941	6.797E-06
UNC50	-0.0037131	6.851E-06
TMEM242	-0.0240672	6.858E-06
SLC35A3	-0.1052819	6.892E-06
SUCLG1	0.1328917	6.938E-06
HBP1	-0.0762021	6.952E-06
RNF126	0.0280986	6.978E-06
PTPRE	0.2622934	6.979E-06

FOPNL	-0.0546663	6.987E-06
PAIP1	-0.0805053	6.988E-06
RAN	0.2123656	6.993E-06
RAB3GAP1	-0.0076073	7.004E-06
LEO1	0.0079011	7.032E-06
BRI3BP	-0.1077481	7.042E-06
B3GAT3	0.0183392	7.071E-06
SREBF2	-0.0994245	7.1E-06
GLRX3	-0.0252884	7.103E-06
FMC1	-0.1063079	7.104E-06
MGME1	0.0565719	7.113E-06
FAH	-0.0279696	7.114E-06
PPM1G	0.0910322	7.145E-06
DMXL1	-0.0499565	7.147E-06
APRT	0.1978284	7.156E-06
VPS26A	0.0042381	7.178E-06
NPIPA1	-0.060713	7.215E-06
VSIG10	0.0748959	7.239E-06
HDAC7	0.068123	7.239E-06
TIMM50	0.0061268	7.24E-06
OXA1L	0.0107375	7.264E-06
STK25	0.0170522	7.265E-06
FEN1	0.0647962	7.283E-06
ANXA6	0.1226892	7.307E-06
YKT6	-0.0380004	7.312E-06
DUS1L	0.0554488	7.335E-06
RB1	-0.0866556	7.338E-06
WIZ	-0.0040672	7.347E-06
ZNF548	-0.0251978	7.372E-06
OXLD1	0.0908205	7.399E-06
DPM3	-0.1320327	7.434E-06
MEA1	0.0152878	7.443E-06
CDS2	0.0159591	7.458E-06
DNAJC5	-0.0215728	7.516E-06
ELAC2	0.0127683	7.522E-06
GLG1	0.0039262	7.59E-06
MRNIP	0.0293867	7.615E-06
ARHGAP27	0.0956193	7.623E-06
TUBGCP5	-0.007175	7.628E-06
CDADC1	-0.0746882	7.63E-06
ETV3	-0.0479207	7.636E-06
FUNDC2	0.0048979	7.639E-06
CASC4	0.0167181	7.702E-06
HAT1	0.0432939	7.716E-06
DHRS7B	0.0878311	7.747E-06
STRN	-0.0193987	7.762E-06
TMEM161B	-0.046912	7.771E-06
HGS	-0.0168457	7.771E-06
JAK3	0.126658	7.889E-06
ZFHX2-AS1	-0.0200927	7.894E-06

ARHGEF9	0.0071018	7.949E-06
YPEL3	-0.1421422	8.003E-06
KRR1	-0.0567296	8.014E-06
MYNN	-0.0162856	8.014E-06
ZSCAN29	-0.0111759	8.023E-06
TMEM150A	-0.0279258	8.055E-06
KPNA2	0.0282091	8.071E-06
GTF2IRD2	-0.0959673	8.175E-06
ALYREF	-0.0222793	8.182E-06
GSDMD	0.082732	8.184E-06
PJA2	-0.0597372	8.202E-06
PPP4R2	-0.0514086	8.218E-06
CYB561D2	0.0433079	8.304E-06
TPP1	0.0827257	8.348E-06
SNAPC3	-0.0610942	8.395E-06
TVP23B	-0.0484301	8.4E-06
NQO2	-0.0403423	8.427E-06
IQGAP2	0.2227184	8.43E-06
PDXDC1	-0.0800021	8.43E-06
MSRA	0.0119801	8.468E-06
DRAM2	0.0363471	8.49E-06
RIC8A	-0.0091554	8.528E-06
NFATC2IP	-0.0353663	8.543E-06
WBP1	-0.0085099	8.586E-06
PSMB5	0.1262196	8.598E-06
UBA2	-0.0009649	8.61E-06
PAPSS1	-0.0311228	8.632E-06
NSD2	-0.0058143	8.641E-06
MORC3	-0.0631	8.648E-06
RRAGC	-0.0314202	8.719E-06
TNFSF13B	0.2222942	8.73E-06
ZC3H8	-0.0617814	8.819E-06
AARSD1	0.0313997	8.845E-06
LMBRD1	-0.0547722	8.899E-06
CDK11A	-0.0754157	8.9E-06
SMARCA4	-0.0454023	8.908E-06
SMCR8	-0.055745	8.918E-06
CNOT8	-0.09055	8.928E-06
RAB18	-0.0247209	8.93E-06
TOX4	0.0268758	8.975E-06
TEX10	-0.052411	9.051E-06
TRAPPC8	-0.0008956	9.063E-06
RARS2	-0.0163858	9.091E-06
TBC1D2B	0.0931695	9.112E-06
PIGN	-0.0453498	9.23E-06
ALG6	-0.0029073	9.238E-06
P4HTM	0.0860832	9.247E-06
SNHG20	-0.0844675	9.291E-06
ZNF410	-0.0104003	9.297E-06
NOL12	0.0281311	9.36E-06

UBALD1	-0.0192732	9.447E-06
TMEM216	-0.0353089	9.453E-06
RNF19A	-0.0112956	9.485E-06
HTRA2	0.0290376	9.522E-06
STXBP2	-0.0683284	9.576E-06
EEF1AKNMT	-0.0463843	9.582E-06
FAM32A	-0.0400986	9.585E-06
ZDHHC5	-0.0536707	9.594E-06
SNX27	-0.070861	9.596E-06
POP5	0.0149002	9.647E-06
SETD1B	-0.0337527	9.654E-06
PGP	0.0058328	9.686E-06
NR2C2	-0.0481944	9.695E-06
TMEM69	0.0120708	9.76E-06
LHFPL2	-0.0021268	9.765E-06
GSK3A	-0.0358753	9.777E-06
KIDINS220	-0.0496945	9.779E-06
SPDYE3	-0.0435028	9.826E-06
PAM16	0.0561985	9.892E-06
KPNA4	-0.0669479	9.918E-06
ORC2	-0.0324233	1E-05
KAT6B	-0.2232963	1.001E-05
ZNF768	-0.0332445	1.009E-05
ARHGEF1	-0.0143968	1.011E-05
GTF2H2C	-0.0795385	1.022E-05
TXLNG	0.0419961	1.025E-05
HDHD3	0.0105781	1.027E-05
VTI1B	0.0342022	1.031E-05
RBM5	-0.0887045	1.032E-05
MDM4	-0.1115511	1.034E-05
DAZAP2	-0.1538843	1.037E-05
AP2S1	0.0805524	1.039E-05
RAB3GAP2	-0.051321	1.039E-05
PACSIN2	-0.1020473	1.044E-05
PRH1	-0.0455865	1.049E-05
WDR41	0.0032255	1.049E-05
ZNF146	-0.0005543	1.058E-05
RFX7	-0.0067234	1.068E-05
AC007038.2	-0.0552113	1.069E-05
SKIDA1	-0.0361506	1.072E-05
MYO1F	-0.127764	1.073E-05
METTL3	-0.0265749	1.074E-05
AC092683.1	-0.1214874	1.075E-05
TOM1	-0.0779688	1.082E-05
NDUFA6	0.0112967	1.086E-05
MAN1B1	0.0168349	1.099E-05
TRIM33	-0.0848853	1.109E-05
AC073957.3	-0.0454496	1.12E-05
PITPNA	0.0001644	1.12E-05
SUMF1	-0.0025632	1.136E-05

HIPK2	-0.121007	1.142E-05
THAP5	-0.0916076	1.143E-05
COG2	-0.0424731	1.145E-05
VPS72	-0.0279508	1.171E-05
AC004854.2	0.0884625	1.172E-05
AP002449.1	-0.0391204	1.174E-05
ARL17A	-0.0791791	1.176E-05
CRKL	0.0134841	1.178E-05
GSAP	-0.0413188	1.188E-05
NISCH	-0.069088	1.192E-05
ILK	0.0366417	1.198E-05
ZER1	0.0444467	1.202E-05
INPP5B	-0.023475	1.204E-05
ZDHHC7	-0.0350292	1.205E-05
NFYB	-0.0270272	1.205E-05
UROD	0.0595061	1.207E-05
GEMIN7	-0.020515	1.218E-05
MPV17L2	-0.0173391	1.219E-05
CUTC	-0.010721	1.223E-05
TIMM17B	0.0112948	1.224E-05
ORC4	-0.0491952	1.228E-05
ITCH	-0.0503133	1.231E-05
TMEM127	0.0001879	1.236E-05
CDK10	-0.0056783	1.238E-05
OGFOD3	0.0144125	1.24E-05
SFT2D2	-0.0813647	1.242E-05
FAM228B	-0.0716407	1.245E-05
DENND1A	-0.0254707	1.246E-05
LINC01500	-0.0325012	1.247E-05
KDELR2	0.0515207	1.259E-05
UPK3BL1	-0.0382426	1.275E-05
GLO1	-0.0428727	1.278E-05
COX14	0.1123475	1.281E-05
VIM	0.2711449	1.286E-05
MNAT1	-0.0045546	1.286E-05
P3H1	-0.0197351	1.304E-05
WDR75	-0.0188803	1.305E-05
STX18	-0.0439022	1.315E-05
PSMD1	0.0252995	1.316E-05
NDUFS3	0.109627	1.318E-05
SPNS3	0.1234885	1.32E-05
CXCL2	0.1476885	1.329E-05
HERC1	-0.0924796	1.332E-05
INSR	-0.0684907	1.334E-05
ZNF529	-0.0437715	1.338E-05
CUEDC2	0.0879967	1.352E-05
RPL7L1	-0.0653218	1.353E-05
EIF4EBP2	-0.1105766	1.364E-05
TBC1D8	-0.058254	1.366E-05
CD2BP2	0.0661147	1.368E-05

ADAMTSL4	0.1401639	1.372E-05
N4BP2L1	-0.0405807	1.381E-05
SLC38A2	-0.125489	1.382E-05
COMMD4	0.0525016	1.392E-05
PAPOLG	-0.0486721	1.396E-05
ASTE1	0.020078	1.405E-05
HSPA9	0.1017024	1.413E-05
STOM	0.1891228	1.414E-05
ADSL	0.055731	1.427E-05
RALB	0.0873847	1.432E-05
C15orf39	0.1138624	1.437E-05
NPEPPS	-0.1618309	1.438E-05
OARD1	0.0107351	1.439E-05
ALAS1	-0.0706446	1.448E-05
FAM98B	-0.0152849	1.462E-05
PTPN11	0.0352717	1.464E-05
NLK	-0.1025609	1.47E-05
MAP3K12	-0.0356884	1.477E-05
YLPM1	-0.0195925	1.496E-05
RAB5B	-0.0659825	1.501E-05
TRIM8	-0.1014618	1.504E-05
PDIA4	-0.0443823	1.512E-05
ZBED5	-0.0159822	1.515E-05
FAM214A	-0.0697625	1.517E-05
CD81	0.1538013	1.519E-05
HIST2H2AC	-0.0981501	1.519E-05
RELB	-0.0502527	1.519E-05
NARS	-0.1313854	1.523E-05
QPRT	-0.3147916	1.525E-05
PRPF4B	-0.1566816	1.533E-05
ZBED4	-0.1248226	1.539E-05
SCAF4	-0.016345	1.541E-05
Z97192.3	-0.1105598	1.546E-05
AC108488.3	-0.0354016	1.549E-05
AATF	0.0660665	1.554E-05
VAMP7	-0.0444043	1.564E-05
ATG10	-0.012109	1.576E-05
PLAA	-0.0364247	1.577E-05
GGA3	-0.0378035	1.58E-05
CENPB	0.0605317	1.585E-05
HUWE1	-0.030106	1.595E-05
RAB3D	-0.07364	1.605E-05
MAP3K11	0.066254	1.606E-05
HLA-A	0.3201895	1.611E-05
VPS18	-0.0038196	1.615E-05
GRN	-0.2572113	1.62E-05
SLC46A3	-0.0142376	1.623E-05
BBS2	-0.0167686	1.624E-05
SMNDC1	-0.085143	1.63E-05
PLEKHM2	-0.0397886	1.633E-05

CD180	0.1304461	1.638E-05
RFX5	-0.0599091	1.638E-05
ARHGDIA	-0.0058357	1.656E-05
HENMT1	-0.0836233	1.669E-05
BAG4	-0.0920934	1.677E-05
SLF2	-0.0616564	1.678E-05
SLC25A11	0.0916814	1.679E-05
HIP1	0.1235365	1.68E-05
LINC01003	-0.0052712	1.69E-05
ZNF43	-0.0511775	1.694E-05
TET3	-0.041672	1.697E-05
FBXO9	-0.0548174	1.7E-05
RANBP9	-0.0613773	1.705E-05
HLA-E	0.2776269	1.709E-05
ZNF594	-0.0013867	1.712E-05
ZNF12	0.0068786	1.718E-05
CAND1	-0.0387993	1.725E-05
PREB	0.0002257	1.733E-05
CDC34	0.0090483	1.744E-05
DPP3	-0.008679	1.745E-05
SRI	0.1747759	1.752E-05
IMMP1L	-0.0216005	1.757E-05
ERMAP	-0.0494647	1.765E-05
LRCH4	0.0397479	1.781E-05
UBE2G1	-0.0327895	1.784E-05
UQCRC1	0.004741	1.785E-05
TRPM2	-0.0586555	1.803E-05
COX16	-0.089486	1.813E-05
POLR3K	0.0303904	1.832E-05
ST3GAL2	-0.0555131	1.835E-05
CDCA7	0.1431717	1.84E-05
UBE2L6	0.0475921	1.845E-05
SNX29	-0.0240201	1.846E-05
SF3A2	-0.0627126	1.852E-05
ECI2	0.0884137	1.879E-05
DUSP23	0.0612063	1.88E-05
PDHX	0.0011227	1.886E-05
PPFIBP1	-0.0309193	1.893E-05
SPIN2B	-0.0291735	1.902E-05
GANAB	-0.053626	1.906E-05
TCERG1	-0.078919	1.911E-05
ZMIZ2	-0.0380635	1.917E-05
SRP54	0.0149758	1.924E-05
MFAP1	0.0741043	1.928E-05
CDC37	0.0721464	1.948E-05
DCTD	0.0080284	1.955E-05
WRN	-0.0127235	1.96E-05
EXOSC10	-0.0165986	1.963E-05
NOB1	-0.0033854	1.965E-05
STX7	-0.19958	1.983E-05

HEIH	0.0225548	1.994E-05
STAM2	-0.0337664	2.005E-05
WDR12	-0.0379533	2.01E-05
AKIRIN1	-0.032864	2.011E-05
ITGAX	0.0744408	2.022E-05
SOD2	0.0624761	2.027E-05
AIP	0.0882244	2.03E-05
RAB11A	0.0068703	2.03E-05
VMP1	0.0360597	2.04E-05
GABPA	-0.078285	2.04E-05
SLCO3A1	-0.1182969	2.049E-05
PGAM5	-0.0351745	2.057E-05
RELT	0.0846568	2.058E-05
AL135925.1	-0.0117076	2.07E-05
SP110	0.0945876	2.073E-05
ZNF273	-0.001251	2.074E-05
MTHFD1L	-0.0306407	2.077E-05
MGAT4B	0.0919413	2.086E-05
PDCL3	-0.0285481	2.093E-05
MITD1	-0.1038034	2.094E-05
SNRPB	0.1263214	2.094E-05
TSEN15	-0.0387203	2.095E-05
CDC27	-0.0289801	2.097E-05
PPP1CC	0.0271383	2.105E-05
RABEP1	0.0183404	2.111E-05
RCC1	-0.0263382	2.113E-05
STK38L	-0.1070822	2.119E-05
SUMO3	-0.1276098	2.13E-05
RBPJ	-0.1062358	2.137E-05
EVI5	-0.095087	2.138E-05
CRLF3	-0.0516175	2.141E-05
PTRH2	0.0579253	2.144E-05
TMEM68	-0.0026024	2.147E-05
UBAC2	0.0210164	2.147E-05
TRADD	0.0240901	2.16E-05
ST3GAL4	0.0078798	2.16E-05
PIGC	-0.0704161	2.175E-05
ZMYM3	-0.030869	2.182E-05
RNF4	-0.0039653	2.214E-05
SF3A3	0.0509668	2.225E-05
NPR3	0.1705842	2.233E-05
PPP2R2A	0.0445778	2.255E-05
UPF1	-0.0153085	2.257E-05
TMEM42	0.0049499	2.269E-05
AC004847.1	-0.0391901	2.271E-05
KIAA0100	-0.042751	2.276E-05
ENO1	-0.1436052	2.291E-05
THAP4	0.0097492	2.298E-05
FADS2	-0.0231228	2.305E-05
PPP2R1A	-0.0273011	2.328E-05

ZNF134	-0.0128873	2.338E-05
SAT2	0.0757135	2.354E-05
GPT2	-0.0072737	2.357E-05
RASSF2	-0.0913885	2.372E-05
NELFE	0.0244367	2.373E-05
ECI1	0.0807546	2.378E-05
STK11	0.0099766	2.393E-05
GSTZ1	0.084146	2.402E-05
HPCAL1	0.1030757	2.405E-05
SMIM20	0.0218038	2.406E-05
RNPS1	-0.0916726	2.41E-05
BRIX1	0.0354	2.418E-05
ROMO1	-0.164131	2.432E-05
ATP9B	-0.03829	2.437E-05
ALG11	-0.0472218	2.441E-05
MTHFD2L	-0.1226958	2.446E-05
RHOQ	0.0144947	2.45E-05
SMIM29	0.0410975	2.459E-05
AC080038.1	-0.0474444	2.47E-05
NUDCD1	0.0634472	2.478E-05
SELENOT	-0.003862	2.492E-05
ANAPC4	-0.0329184	2.502E-05
PIBF1	-0.0428671	2.525E-05
GSE1	-0.196707	2.527E-05
DENND4A	-0.0404532	2.534E-05
KLHDC1	-0.0212581	2.567E-05
SLC66A2	0.0270875	2.573E-05
TBC1D17	-0.0562363	2.575E-05
MAP2K5	-0.0065612	2.575E-05
NSUN6	-0.0404281	2.584E-05
KDM6B	-0.0340253	2.59E-05
ZC3H11A	-0.0392031	2.598E-05
ZBTB26	-0.0074519	2.607E-05
NUP107	-0.0475766	2.61E-05
ZNFX1	-0.0062864	2.61E-05
NAE1	0.0515329	2.616E-05
AC096667.1	-0.1543421	2.623E-05
SEH1L	-0.0596979	2.624E-05
BCAS2	0.0281298	2.627E-05
TTN	-0.0424661	2.628E-05
SPOP	0.0588747	2.642E-05
FAM160B2	-0.0122922	2.645E-05
CDT1	0.1234392	2.649E-05
RRAGB	0.0505073	2.656E-05
ARMCX3	0.158048	2.661E-05
PDE7A	0.0866681	2.695E-05
FBXO42	-0.0170337	2.699E-05
KIAA1143	-0.1020611	2.703E-05
BRF2	-0.0039616	2.715E-05
C1RL	0.0212621	2.716E-05

KLHDC8B	0.0785979	2.719E-05
LAGE3	0.0689352	2.72E-05
PDIA6	0.1245518	2.722E-05
SLC9A3-AS1	-0.0362062	2.74E-05
TMEM63A	0.1058693	2.749E-05
KAT5	-0.0146277	2.756E-05
MPC1	-0.1106519	2.761E-05
UBQLN4	-0.0312776	2.779E-05
TMEM106A	-0.035263	2.786E-05
TRAPPC10	-0.104294	2.795E-05
PDK2	0.0045722	2.795E-05
PRPF38A	-0.027624	2.798E-05
HIST1H2BC	0.013775	2.799E-05
EAF2	0.1693567	2.806E-05
FPGS	0.0232704	2.808E-05
H2AFZ	0.3125859	2.808E-05
SLC30A9	-0.0891204	2.821E-05
KCNN4	0.0235268	2.821E-05
DRG1	-0.0058558	2.824E-05
CREB3L2	-0.0987812	2.833E-05
NUP85	-0.0457302	2.842E-05
PRR12	-0.0544716	2.846E-05
MATK	0.114769	2.848E-05
COQ7	0.0723028	2.849E-05
SLC33A1	-0.0936374	2.85E-05
CUL1	-0.0056051	2.851E-05
HECTD4	-0.0616249	2.861E-05
COG5	-0.0161001	2.864E-05
CREG1	-0.1758699	2.871E-05
MSL1	-0.0335363	2.873E-05
MPDU1	-0.017636	2.881E-05
CTDSP1	-0.0338317	2.884E-05
RNF44	-0.1228697	2.895E-05
EIF2AK1	-0.1106465	2.898E-05
BCR	0.033698	2.902E-05
DTD1	0.0792578	2.905E-05
NUP205	-0.0093909	2.912E-05
AC022098.1	-0.0263084	2.916E-05
SNHG15	-0.0910156	2.925E-05
EMG1	0.0789102	2.942E-05
CHCHD1	0.1023555	2.951E-05
ACTR8	-0.0237363	2.956E-05
FKBP7	-0.0053762	2.978E-05
IRAK1	-0.055582	2.993E-05
MARCH5	-0.0700331	2.997E-05
GIMAP2	-0.004261	2.999E-05
ZNF780A	0.0178232	3.011E-05
LAMTOR2	0.1149319	3.033E-05
VPS33A	-0.0415629	3.038E-05
HEATR5B	-0.0825482	3.059E-05

INTS14	0.0083225	3.079E-05
CRYZ	0.0071814	3.08E-05
MBD2	0.0738259	3.098E-05
IER3IP1	0.0397029	3.102E-05
ZNF33B	-0.054265	3.11E-05
TMEM165	-0.0797715	3.122E-05
RHPN1	-0.0182572	3.124E-05
SENP7	-0.0669425	3.126E-05
PAFAH1B2	-0.0152886	3.128E-05
FAM118A	-0.0595732	3.13E-05
TMEM160	0.1369708	3.133E-05
SEC22C	-0.0081698	3.135E-05
RALA	0.1299853	3.139E-05
SPG21	-0.0269819	3.153E-05
PNPT1	-0.0772519	3.155E-05
MTPAP	-0.0337923	3.177E-05
LPCAT1	-0.0272835	3.184E-05
TRAPPC2L	0.0774417	3.203E-05
TCF3	-0.0206223	3.235E-05
UBR1	-0.0461623	3.238E-05
PPIP5K1	-0.0415067	3.24E-05
TRMT61B	0.0242166	3.249E-05
HIF1AN	-0.0220589	3.253E-05
INPP4A	-0.0173396	3.259E-05
PIM3	0.0496186	3.279E-05
ST3GAL1	0.1168087	3.288E-05
BRD8	-0.0660135	3.299E-05
C1orf109	-0.0372856	3.341E-05
ACACA	-0.0138506	3.342E-05
C1D	-0.0335956	3.348E-05
DCLRE1C	-0.0792895	3.363E-05
AC022087.1	-0.031236	3.368E-05
DCAF16	-0.0876898	3.382E-05
RC3H1	-0.13178	3.388E-05
SLAIN2	-0.0078461	3.392E-05
DCUN1D3	0.1065301	3.397E-05
GLMN	-0.0619994	3.47E-05
SSH1	-0.0702076	3.471E-05
CTDSP2	0.0200578	3.484E-05
LINC02245	-0.0226954	3.49E-05
CMC2	-0.0990701	3.499E-05
PLGLB2	-0.0386894	3.503E-05
ZBTB7A	-0.0488857	3.504E-05
ZDHHC20	-0.0759226	3.514E-05
ZNF814	-0.0683208	3.518E-05
ARIH2	-0.0356369	3.529E-05
TMC3-AS1	-0.0406672	3.537E-05
YWHAQ	0.0355163	3.542E-05
MYL6B	0.0713825	3.552E-05
ZNF566	-0.0618041	3.561E-05

CHMP1A	-0.0190334	3.577E-05
AC064805.2	-0.0332934	3.582E-05
RAB8A	0.135504	3.586E-05
TWF1	-0.0057543	3.636E-05
AKR7A2	0.1361289	3.649E-05
FKTN	-0.0236103	3.652E-05
GDAP2	-0.0157892	3.658E-05
ZNF830	-0.0681019	3.669E-05
PRR14	0.0007555	3.673E-05
MRPS14	-0.0023807	3.677E-05
SMIM27	-0.1724598	3.687E-05
TGFBR1	-0.0319258	3.698E-05
ZNF101	-0.0310964	3.709E-05
GMIP	-0.055148	3.716E-05
ACTR1B	0.0548506	3.743E-05
SLC25A37	-0.1393992	3.765E-05
PIK3C3	-0.1305453	3.77E-05
AC073389.1	0.0092066	3.773E-05
TEN1	-0.0157644	3.778E-05
IMPDH1	0.0589887	3.792E-05
FDPS	0.0513791	3.812E-05
CLEC2B	0.162526	3.828E-05
STMN3	0.0043481	3.839E-05
TNIP1	-0.0484583	3.855E-05
KMT2E	-0.1126426	3.856E-05
EIF4H	-0.0123168	3.862E-05
PTDSS1	-0.1183759	3.872E-05
MXD4	-0.065687	3.872E-05
ATP2A2	0.0536129	3.881E-05
GOLT1B	0.0072631	3.891E-05
ETFA	0.0020441	3.895E-05
PDK3	-0.0393756	3.917E-05
RBL2	-0.0918781	3.932E-05
METTL17	-0.0545477	3.94E-05
DICER1	-0.1472578	3.942E-05
BIN3	-0.0236853	3.961E-05
PRELID3B	-0.0266982	3.962E-05
PSMA7	0.1968221	3.977E-05
CRYZL1	-0.0108344	3.98E-05
SNHG5	-0.1852925	3.994E-05
TPRKB	-0.0445003	4.016E-05
NCL	0.2861048	4.037E-05
LYPLAL1	0.022954	4.05E-05
TNFRSF4	0.2771576	4.059E-05
SLC38A1	0.207949	4.109E-05
RUVBL2	0.0886728	4.111E-05
CBL	0.0541969	4.113E-05
VAMP3	-0.0269776	4.137E-05
AC092171.3	-0.0477332	4.148E-05
GNG10	0.159402	4.151E-05

BMS1	-0.0740166	4.151E-05
HDAC1	0.0366074	4.177E-05
SIRT7	-0.0365276	4.177E-05
RNF38	-0.060873	4.18E-05
STX16	0.1467483	4.223E-05
ZCCHC8	-0.0439171	4.239E-05
FBXO45	0.0824139	4.242E-05
EIF4E	-0.0326925	4.278E-05
NUP88	0.0051931	4.303E-05
ABL2	-0.0245407	4.315E-05
RFC5	-0.0084548	4.346E-05
HEATR1	-0.0219792	4.355E-05
NKD1	-0.0634486	4.382E-05
TLK2	-0.0762069	4.387E-05
BZW1	-0.0581727	4.391E-05
ZMYM4	0.0316229	4.395E-05
SRSF1	-0.0214597	4.4E-05
ZNF622	0.0535437	4.405E-05
TSC2	-0.0547933	4.423E-05
SWI5	0.0655524	4.423E-05
STK38	-0.0638457	4.436E-05
PSMC3	-0.0098016	4.447E-05
SLC35E3	-0.1476651	4.47E-05
LINC01278	-0.0387192	4.476E-05
NUTM2B-AS	-0.0897683	4.496E-05
NADSYN1	-0.0269199	4.521E-05
PAN3	-0.0852613	4.521E-05
DR1	-0.0005117	4.539E-05
FAM193B	-0.0062938	4.582E-05
BORCS5	-0.0293275	4.591E-05
PRKCQ	0.0151161	4.605E-05
PRICKLE1	0.0996976	4.621E-05
PDCD4	-0.2391228	4.643E-05
SEPTIN9	-0.0115475	4.672E-05
AP3S2	-0.0188092	4.672E-05
CREB1	-0.160115	4.712E-05
NFKB1	-0.0153971	4.714E-05
MRPS9	0.0253556	4.74E-05
XPO6	0.0228969	4.743E-05
MPP5	-0.0025114	4.766E-05
SLC17A9	0.0644132	4.79E-05
MTR	-0.0111704	4.798E-05
ARL8B	-0.129974	4.815E-05
PLEKHF1	-0.0400835	4.816E-05
DNAJB4	-0.0085295	4.87E-05
SIK3	-0.0301217	4.877E-05
PHKG2	-0.041825	4.884E-05
SCAP	-0.0058366	4.91E-05
IPO8	-0.0855525	4.915E-05
EBAG9	0.0492538	4.928E-05

LRMP	0.0416549	4.934E-05
SCAMP1-AS	0.0053103	4.973E-05
USP10	0.0597797	4.978E-05
SAP30L	-0.0029605	4.983E-05
ATE1	-0.0530094	5.024E-05
CHIC2	0.0027054	5.037E-05
C19orf18	-0.01384	5.046E-05
CCDC22	0.0305124	5.047E-05
ZFPM1	0.0448479	5.051E-05
ZNF160	0.003801	5.075E-05
R3HCC1	0.0463937	5.099E-05
ALDH5A1	0.0685699	5.104E-05
SDHAF1	-0.0264721	5.109E-05
HSPB11	0.0303838	5.13E-05
AP1B1	-0.0061255	5.134E-05
SFSWAP	-0.0030253	5.137E-05
CEBPZOS	-0.0443941	5.146E-05
HOXA10	-0.1188737	5.158E-05
SPOPL	-0.052092	5.159E-05
TBKBP1	-0.0087963	5.176E-05
KTI12	-0.0164703	5.183E-05
WARS	-0.0088033	5.189E-05
CNOT9	-0.060723	5.201E-05
RIN3	-0.0982223	5.208E-05
GTPBP2	-0.0454439	5.271E-05
SND1	0.0421429	5.282E-05
WDR18	0.086092	5.285E-05
RNF111	-0.018621	5.326E-05
HIBCH	-0.0042495	5.333E-05
VPS13B-DT	-0.0528672	5.354E-05
CBWD2	-0.1023971	5.372E-05
ZNF83	-0.0174196	5.377E-05
SSBP4	0.0667065	5.469E-05
PRMT5	-0.0354735	5.473E-05
ZFAND6	-0.1302916	5.503E-05
CRTC2	-0.0322096	5.538E-05
CYFIP2	0.1720934	5.563E-05
XPC	-0.0767164	5.565E-05
PLCXD1	0.0482796	5.585E-05
FAM168B	-0.0307735	5.594E-05
DEPTOR	-0.0756899	5.605E-05
GPATCH4	0.1459313	5.624E-05
MBD3	-0.0192183	5.625E-05
ST7L	-0.0174945	5.643E-05
MAPK1IP1L	-0.0727065	5.668E-05
CREB3	0.0153496	5.695E-05
FAM120A	-0.0444878	5.705E-05
DDOST	0.0867406	5.709E-05
SURF2	0.045272	5.725E-05
RNF216	-0.0006957	5.732E-05

MBIP	-0.0205189	5.737E-05
MFSD3	-0.007818	5.774E-05
AL355336.1	-0.0429005	5.776E-05
MTFR1	-0.0296223	5.8E-05
IL12A-AS1	-0.1651768	5.804E-05
RSBN1	0.0223572	5.805E-05
ECHDC1	-0.0343004	5.812E-05
PYROXD1	-0.0648121	5.817E-05
RPS28	-0.1338686	5.829E-05
HEATR3	-0.0219892	5.83E-05
GOT2	0.045693	5.868E-05
RBFA	0.1110754	5.876E-05
RAB35	-0.0260736	5.887E-05
METTL7A	-0.12135	5.939E-05
TMEM64	0.0156444	5.964E-05
AC018362.1	-0.0589675	6.027E-05
NCF4	-0.2441665	6.062E-05
SHOC2	-0.1020573	6.064E-05
AC026471.1	0.051067	6.096E-05
TADA3	-0.005254	6.117E-05
TAF8	-0.030079	6.132E-05
SLC8B1	-0.0599538	6.137E-05
AK6	0.0530638	6.14E-05
RRM2B	-0.1430062	6.152E-05
SF3A1	-0.0585091	6.154E-05
NUCB1	0.0030534	6.161E-05
CENPX	0.0244365	6.199E-05
LCT	0.0826491	6.204E-05
SUPT20H	-0.0694765	6.225E-05
KAT8	-0.0206064	6.225E-05
SEC13	-0.042707	6.25E-05
COPB1	0.0244437	6.273E-05
IFI27L1	0.0175842	6.275E-05
UBXN2A	-0.0612678	6.279E-05
ARF3	0.0544696	6.289E-05
CYB5R3	0.0561967	6.319E-05
SAMD4B	-0.0142252	6.321E-05
CCNT1	0.0401873	6.325E-05
CARS2	0.0519785	6.336E-05
MRPL30	-0.0587943	6.358E-05
DNAJB12	-0.0126941	6.381E-05
RNPEP	-0.0152392	6.4E-05
GARS-DT	-0.0809904	6.434E-05
MAGED1	0.1047215	6.446E-05
SNAP29	0.0604668	6.472E-05
SMARCD2	0.0401568	6.476E-05
SETMAR	-0.0052364	6.485E-05
FRMD4B	0.1246784	6.488E-05
LBHD1	0.0201168	6.492E-05
PAK1IP1	-0.0466725	6.504E-05

FAM215B	-0.0147364	6.513E-05
CAPZA2	-0.0876913	6.532E-05
MAP3K5	-0.030758	6.533E-05
TMEM177	0.0212613	6.533E-05
CDIPT	0.0245456	6.537E-05
SP3	-0.0941321	6.542E-05
RRP15	-0.0544237	6.542E-05
IL1B	0.2317297	6.552E-05
ABHD3	-0.0296738	6.584E-05
VEZF1	-0.1005861	6.593E-05
KCNE3	-0.1191316	6.618E-05
NAT14	0.0506822	6.643E-05
SLC22A16	0.0448577	6.665E-05
RAI1	-0.0349997	6.675E-05
ELK4	-0.0819931	6.696E-05
SPNS1	-0.032528	6.706E-05
ZFR	-0.0908791	6.706E-05
HIPK1	-0.1225836	6.719E-05
PHACTR4	-0.1019184	6.731E-05
FABP5	0.2292996	6.77E-05
ISOC2	0.0729909	6.806E-05
TFE3	-0.0314353	6.849E-05
LARP1B	-0.013559	6.854E-05
ATP11A	-0.0400943	6.872E-05
PHF6	-0.0194747	6.917E-05
LMO2	-0.2245026	6.954E-05
PDP1	-0.0416371	6.969E-05
SYNGR1	0.1939188	7.021E-05
CANT1	0.0079643	7.024E-05
GLRX2	-0.002064	7.084E-05
ARHGAP45	-0.0329749	7.104E-05
FAR2	0.012628	7.134E-05
CISD3	0.0887095	7.161E-05
SPRTN	-0.0305539	7.164E-05
PMF1	0.0766788	7.208E-05
LINC00623	-0.0510615	7.226E-05
ATAD2	0.0867771	7.228E-05
IRF2	0.0590448	7.267E-05
KIF22	-0.0676236	7.276E-05
PTK2B	0.0029995	7.328E-05
HNRNPH2	0.0059663	7.348E-05
TIAL1	-0.089575	7.353E-05
HGSNAT	0.0054755	7.362E-05
TCAIM	-0.0897054	7.404E-05
ADARB1	-0.0282144	7.425E-05
CHMP2A	0.1096206	7.441E-05
BCL9L	-0.0590104	7.447E-05
SCAI	-0.0441069	7.452E-05
VPS4B	-0.0802059	7.455E-05
GTF2H4	0.02233	7.484E-05

ICMT	-0.0119397	7.507E-05
THAP11	-0.0191468	7.594E-05
EBP	-0.0510809	7.633E-05
MRPL16	0.0298762	7.645E-05
FAM172A	-0.0539034	7.647E-05
CCDC124	0.0491014	7.672E-05
STMP1	-0.189331	7.717E-05
LARP4	-0.0413503	7.734E-05
RABGEF1	-0.0158804	7.813E-05
BIRC3	-0.1502272	7.83E-05
SMC1A	-0.0229458	7.84E-05
RNF8	0.0308127	7.841E-05
CENPU	0.0537878	7.859E-05
NAPA	0.0187658	7.878E-05
DLEU1	-0.0120144	7.881E-05
XRRA1	-0.0697514	7.884E-05
SSRP1	0.0892748	7.888E-05
PSMC1	-0.0465003	7.92E-05
DPH3	-0.0036952	7.927E-05
FIGNL1	0.0044706	8.046E-05
LINC00847	-0.0061117	8.072E-05
AC004951.1	-0.0795743	8.156E-05
HSPH1	0.0239988	8.157E-05
LASP1	0.0033605	8.218E-05
CLIP2	0.0334375	8.236E-05
MRPL43	0.0404165	8.274E-05
DNPH1	0.1348742	8.275E-05
SETD3	-0.0185367	8.357E-05
U2AF2	-0.050193	8.378E-05
APEH	-0.0048859	8.385E-05
DLG1	-0.0357785	8.393E-05
SURF1	0.0324177	8.417E-05
ARPC5	0.059505	8.452E-05
SEMA6B	-0.0572803	8.493E-05
SLC30A7	-0.0533412	8.5E-05
CCM2	-0.0452417	8.531E-05
RNF146	0.0188098	8.539E-05
BMF	-0.0304556	8.58E-05
MRTO4	0.0429713	8.587E-05
PRMT2	-0.0724383	8.616E-05
ANKRD27	-0.0916618	8.664E-05
ZBED3	0.0823764	8.666E-05
FOXK2	-0.0524772	8.686E-05
MRPL19	0.0399721	8.699E-05
ZNF627	-0.0463603	8.72E-05
EPM2A	-0.0283996	8.748E-05
MRPS27	0.0315219	8.806E-05
POLR2F	0.0223331	8.814E-05
RNF220	-0.0724771	8.831E-05
FAM174A	-0.0106739	8.832E-05

PLEKHA3	0.0059086	8.893E-05
RAD51D	-0.0271941	8.913E-05
NOA1	-0.0017821	8.935E-05
VAT1	-0.0877651	8.99E-05
SMG5	0.005892	9.046E-05
MAP4	-0.0587668	9.073E-05
ZMAT3	-0.0589004	9.128E-05
NCAPD2	-0.003127	9.137E-05
MILR1	-0.0414139	9.211E-05
DROSHA	-0.0293287	9.217E-05
YIPF6	0.0054018	9.229E-05
MMGT1	0.0043328	9.267E-05
MRPL23	-0.0549812	9.295E-05
COQ10B	-0.0555105	9.297E-05
XPNPEP1	-0.0462553	9.299E-05
COX10-AS1	-0.0192156	9.301E-05
PHF23	-0.0545288	9.336E-05
ACCS	-0.003775	9.356E-05
NOG	-0.0797251	9.385E-05
ANAPC7	0.0475154	9.386E-05
SEC23B	-0.0415241	9.387E-05
ZNRF2	0.010956	9.425E-05
ACTL6A	0.0341436	9.471E-05
FTX	-0.2114413	9.493E-05
SELENOP	0.2009145	9.499E-05
ATF7	-0.0273825	9.551E-05
ZC3H7A	-0.07484	9.562E-05
STAU2	-0.0149687	9.573E-05
ERI3	0.0235096	9.579E-05
WDR83	-0.0174138	9.586E-05
HNRNPUL2	-0.0614384	9.607E-05
RPP21	0.0233139	9.617E-05
PGM2	-0.0288151	9.638E-05
ZMYM1	0.0379467	9.648E-05
SLC25A1	0.0467238	9.658E-05
ZFP91	-0.1030745	9.699E-05
WDR59	0.0032428	9.701E-05
THOC5	-0.0210533	9.777E-05
MED4	-0.0349981	9.798E-05
HOOK2	0.0139687	9.802E-05
NANS	0.1182485	9.838E-05
ANKRD36B	-0.0688864	9.882E-05
C5orf56	-0.1292282	9.888E-05
VAMP5	-0.06999	9.949E-05
ATP6V1E1	-0.0924197	9.98E-05
C2orf68	-0.0718913	0.0001001
FUT4	-0.1508097	0.0001002
MZT1	0.0034959	0.0001005
KLHL5	-0.0463652	0.0001007
ASPSCR1	-0.0033991	0.0001007

DNAJC11	0.010425	0.0001011
FBXW5	-0.0029493	0.0001012
SLC15A4	-0.0212296	0.0001023
OTUD6B	-0.081094	0.0001025
GTPBP10	-0.0501912	0.0001027
UFD1	-0.0332366	0.0001028
ATP6V1B2	0.0656461	0.0001028
DCAF17	-0.0047356	0.0001028
TUT7	-0.0714017	0.0001031
PRDX3	-0.0072692	0.0001036
E2F3	-0.1260104	0.0001039
DGCR8	-0.0144568	0.0001041
PRPSAP2	-1.035E-05	0.0001041
UBL7	-0.0352083	0.0001053
AC002310.1	0.0057259	0.0001053
RP2	-0.0432954	0.0001057
UHMK1	-0.1109409	0.0001059
ZBTB33	-0.0402408	0.0001064
PAICS	0.108197	0.0001067
SLC36A4	-0.1147033	0.0001069
SNRPA	0.0167	0.0001069
SEC63	0.0405187	0.000107
ATG5	-0.0031153	0.0001071
HDAC10	-0.0363183	0.0001071
MAIP1	0.0003308	0.0001073
ZNF609	-0.0923544	0.0001075
ZMYM6	-0.090296	0.000108
SAP30	0.0458606	0.0001082
EED	0.0127293	0.0001085
IPO7	-0.0154999	0.0001086
RELCH	-0.0294116	0.0001089
CNOT3	0.0068316	0.0001092
NBAS	0.0816102	0.0001092
SPOUT1	-0.0037627	0.0001095
IKZF5	-0.0494489	0.0001095
PPIL4	0.0474837	0.0001097
AL022311.1	-0.0038032	0.0001102
DDX59	-0.0219617	0.0001107
ENTPD6	0.0006451	0.0001108
UBE2E3	0.0024944	0.0001109
DCAF8	0.0031071	0.0001112
GRB10	-0.0330981	0.0001113
PLA2G12A	0.0742798	0.0001114
MAP2K7	-0.0231121	0.0001116
KLC1	-0.0355529	0.0001119
C12orf66	-0.0108744	0.0001119
TDP2	0.0104378	0.000112
SP100	-0.1691787	0.0001122
TMEM41A	-0.0130815	0.0001125
DEXI	-0.0223367	0.0001125

B3GALT6	0.0340716	0.0001127
RNF2	0.0051597	0.0001128
EEPD1	-0.0055075	0.0001129
VBP1	-0.0277199	0.0001133
NBPF19	-0.1139774	0.0001134
RCN2	-0.0345019	0.0001136
PPP1R21	-0.1078538	0.0001142
STX8	0.0039186	0.0001144
AC124016.2	-0.0437258	0.0001149
ZFYVE26	-0.0046358	0.0001149
KIF2A	0.1936755	0.0001156
TRMT1	-0.0059167	0.0001157
SAC3D1	-0.0149207	0.0001159
SFT2D3	-0.0104366	0.0001163
PRKX	-0.0060651	0.0001163
CDCA7L	0.0826697	0.0001164
DIPK1B	0.0273957	0.0001166
CCDC7	-0.0123699	0.0001168
TLE3	-0.0429715	0.0001169
C1QBP	0.2083987	0.0001175
TMEM30A	-0.0810297	0.0001176
HSD17B10	-0.0290983	0.0001183
HMG20B	0.0324951	0.0001191
CSTF1	0.0117588	0.0001193
DPYD	-0.0148074	0.0001197
ZNF891	-0.0376555	0.0001202
CPSF7	-0.0086985	0.0001203
IMPAD1	-0.0350647	0.0001204
HSP90AB1	0.1851255	0.0001206
LRIG2	-0.0587777	0.0001206
MAT2B	-0.1529052	0.0001206
ACAD8	0.0016814	0.0001211
APOBR	-0.0765864	0.0001215
C12orf57	0.0159256	0.0001217
CCDC82	-0.0086865	0.000122
CCDC32	-0.0274354	0.0001221
NCSTN	-0.0398496	0.0001232
TIMM29	0.0073318	0.0001232
FTCDNL1	-0.0516642	0.0001236
HNRNPUL1	0.024636	0.0001236
TRAPPC13	-0.0328408	0.000124
NDUFA10	0.000832	0.0001243
PAXIP1-AS2	-0.0236461	0.0001244
ZNF652	-0.0366798	0.0001246
DPEP2	0.0169435	0.0001246
RERE	-0.0466159	0.0001247
WBP11	-0.0680785	0.0001264
TPX2	0.106577	0.0001265
MDC1	-0.0030243	0.0001281
FAM53B	-0.0647631	0.0001283

MALSU1	0.0219086	0.0001295
DUSP11	-0.0339362	0.0001298
AC092329.4	-0.0170631	0.0001299
MKKS	-0.0097271	0.0001305
NUDT14	-0.0395829	0.0001305
CD99L2	-0.0405253	0.0001307
GID8	-0.0422387	0.0001309
CTNNBIP1	0.0050596	0.0001316
TPK1	-0.0238434	0.0001317
EHBP1	-0.0001786	0.0001322
BCAS3	-0.0493217	0.0001322
RBM12B	-0.0936981	0.0001333
CR381653.1	-0.0229891	0.0001335
GTF2B	-0.0017416	0.0001348
GAR1	0.027494	0.0001349
FLI1	0.0386907	0.0001351
COG3	-0.0397585	0.0001352
MERTK	-0.0208884	0.0001354
VKORC1L1	0.0121129	0.0001367
POLR2B	-0.0289013	0.0001368
ANKRD46	-0.0369278	0.0001369
BAG5	0.0377753	0.0001369
PIGS	-0.0131379	0.000137
MRPS22	0.055597	0.000137
GALNT11	-0.040987	0.0001371
SPSB2	-0.0156456	0.000138
METAP1D	-0.0280046	0.0001381
FBXW11	-0.0258273	0.0001381
ZNF671	0.0016989	0.0001384
ZNF780B	0.0121752	0.0001402
PTAR1	-0.098952	0.0001403
GFER	0.0638611	0.0001405
WDR54	0.0938762	0.0001408
CCDC189	-0.0702127	0.0001416
GAK	-0.0456161	0.0001421
ACAP2	0.0117601	0.0001422
PRPF3	-0.058846	0.0001425
AL353807.5	-0.0354147	0.0001431
IFRD1	0.034714	0.0001433
USP19	-0.0234666	0.0001444
CNPY3	0.1422616	0.0001445
RNF14	-0.0399939	0.0001449
GGH	0.088717	0.0001451
TRAF3IP2	-0.0256434	0.0001451
RAP1GDS1	-0.0092198	0.0001454
RBM6	-0.0491451	0.0001459
WDR46	0.0532678	0.0001459
INPP5F	-0.0402805	0.000146
CHST11	0.001258	0.0001462
ALG2	-0.0181293	0.0001467

TRUB2	0.0255611	0.0001467
NAA60	8.964E-05	0.000148
RNPC3	-0.1122473	0.0001487
RASA4	-0.0083149	0.0001488
RBM19	-0.0207187	0.0001489
BAK1	-0.0209688	0.000149
LMBR1L	-0.0319191	0.000149
SPINT2	0.183582	0.0001496
AGO1	-0.0943063	0.0001499
GPSM2	-0.1004586	0.0001499
SCML2	0.1015275	0.0001505
AL138756.1	-0.0709304	0.0001512
SMIM8	-0.0539164	0.0001515
TFDP1	-0.0191167	0.0001518
TMA7	-0.1916519	0.000152
ECD	-0.0375114	0.0001521
PRKAG2-AS	0.077555	0.000153
ATR	-0.1686188	0.0001535
TXNDC15	-0.0108368	0.0001535
CTSZ	0.0743999	0.0001537
MSL3	-0.0409512	0.0001544
RNMT	-0.031194	0.0001552
STAT5A	-0.1388277	0.0001554
MRFAP1	-0.0329213	0.0001555
HILPDA	0.0256922	0.0001557
PLXNC1	-0.1580832	0.0001559
RBM7	-0.0279196	0.0001561
GNAI3	0.0182471	0.0001568
UBL3	-0.0085932	0.0001573
AP4M1	-0.0160273	0.0001575
NAA15	0.0373138	0.0001579
IPO5	0.0269388	0.0001586
TCP1	0.0772223	0.0001594
UCHL3	0.001805	0.0001617
CHAMP1	0.0019796	0.0001619
PLXND1	0.1354638	0.0001625
AKT2	0.0271256	0.0001634
NBPF14	-0.0839932	0.0001636
SPRYD4	-0.0310259	0.0001639
TOMM5	0.1332587	0.0001639
TNFRSF1B	0.118565	0.0001641
UBE2N	0.0379205	0.0001649
PLTP	0.0624031	0.000165
SMARCAL1	-0.015598	0.0001653
MSH6	0.0516125	0.0001657
STXBP4	-0.0727858	0.000166
DCAF13	0.0335751	0.0001663
TARDBP	-0.0772197	0.0001667
CPLANE1	-0.0501909	0.0001668
HESX1	-0.0087225	0.0001671

FAHD2B	0.0118852	0.0001675
ZNF26	-0.0902586	0.0001675
STT3A	-0.0345093	0.0001678
DNAJB11	0.0406629	0.000168
MYL6	0.1781507	0.000168
QRICH1	-0.0372748	0.0001684
LRRFIP2	-0.0191381	0.0001696
TMX3	-0.0897805	0.0001707
HSPA1A	-0.0333853	0.000172
FARSB	0.0051267	0.0001727
PPP1R18	0.0759331	0.0001733
FAM13B	-0.053446	0.0001743
BCL10	-0.0518421	0.0001743
RPE	-0.0376652	0.0001746
ODR4	-0.0386266	0.0001748
NAT10	0.0445638	0.0001748
AC093297.2	-0.0424073	0.0001752
AL513128.2	-0.0289054	0.0001753
DNPEP	-0.0011042	0.0001761
ODC1	0.0975105	0.0001769
TIPRL	0.0298492	0.0001773
MKRN1	0.0094641	0.0001773
FASTKD3	-0.018553	0.0001778
NUTF2	-0.053659	0.0001785
SPSB3	-0.0372648	0.0001787
RABIF	0.0207769	0.0001793
C12orf10	0.0793901	0.0001795
NUDT2	0.0078584	0.0001797
ACYP2	-0.095615	0.0001797
TMEM87B	-0.0195256	0.0001798
FYCO1	0.002042	0.0001803
ARMC8	-0.0526406	0.0001803
NUCB2	0.2088578	0.0001814
SH3GLB2	-0.0074823	0.0001822
FKBP3	0.0882518	0.0001833
EEF2	-0.102136	0.0001839
RICTOR	-0.1130045	0.000184
C11orf68	-0.0513384	0.0001841
HIF1A	0.1320845	0.0001842
TRGC2	0.1025796	0.0001843
P2RX4	-0.0996615	0.0001848
TPRG1L	0.0070425	0.0001848
MAPKAP1	-0.0323489	0.000185
NAPRT	0.1009257	0.0001851
MAN2B1	-0.021166	0.0001865
UCKL1	-0.0122313	0.000187
TRIM13	-0.0936956	0.0001871
TSR3	0.0222958	0.0001885
GTF2F1	0.0287691	0.000189
GTF2E2	-0.008929	0.0001891

KDM2B	-0.0606113	0.0001896
ACBD6	0.0517366	0.0001908
AC142472.1	-0.0542651	0.0001925
USF3	-0.0535167	0.0001947
ADPRM	0.0066139	0.0001949
CYC1	0.077913	0.0001956
PBXIP1	0.0130669	0.000196
AC139099.2	0.1092579	0.0001961
UBAP2L	-0.0203692	0.0001978
CPSF4	0.0096862	0.0001986
MKNK2	-0.0768567	0.0002007
LIG1	0.0508231	0.0002032
PANK3	-0.0958289	0.0002034
PAQR7	-0.0473209	0.0002038
MRPL46	-0.0116429	0.0002041
UQCC1	-0.0164718	0.0002041
WDR82	-0.0662406	0.0002042
NDUFA9	0.0212222	0.0002046
IMPA1	-0.0293401	0.0002051
HAX1	0.0616398	0.0002053
UBP1	-0.0248232	0.0002059
ACSS1	0.0550305	0.0002062
MEIS1-AS2	-0.0262836	0.0002063
ZG16B	0.1020833	0.0002066
FLCN	-0.0479945	0.0002067
BAG1	-0.0403084	0.0002071
BTBD2	0.002943	0.0002074
SCAND1	0.0675647	0.0002075
PTP4A3	0.1176085	0.0002077
AC127521.1	0.0403953	0.0002087
SDHD	-0.0305426	0.000209
PPM1B	-0.0364673	0.000209
CYTH2	0.0219475	0.0002105
ERF	-0.1096582	0.0002117
AC008764.8	-0.0217624	0.0002119
PITPNA-AS1	0.0510867	0.0002125
SOCS7	-0.0336395	0.0002127
CARHSP1	0.1098541	0.0002132
POLB	0.002675	0.0002133
ABCF2	0.0085667	0.0002136
BTBD7	-0.0608208	0.000215
DLG5	-0.0345301	0.0002151
DNAJC10	-0.1454628	0.0002152
C1orf122	0.0119449	0.0002154
ELMOD2	-0.0663779	0.0002163
HLTF	-0.032901	0.0002167
MARK3	-0.0586498	0.0002167
NPRL2	-0.0154325	0.0002178
PPP1R3D	0.0127904	0.0002178
PLRG1	-0.0448511	0.0002183

FYTTD1	-0.1261569	0.0002185
COA1	-0.0648552	0.0002189
CLASP2	-0.0702263	0.000219
PDCD7	0.1214933	0.0002197
ZNF274	-0.007012	0.0002199
ATP7A	-0.0623707	0.0002207
ERP44	0.0161005	0.0002211
MAFK	0.0496499	0.0002214
MRPS18A	-0.0034127	0.0002214
PRMT9	-0.0409759	0.0002217
AC004865.2	0.0815849	0.0002219
MRPL14	0.0285325	0.0002219
NHLRC2	-0.1058499	0.0002226
VAPB	0.0044727	0.0002242
NIFK-AS1	0.0177686	0.0002256
MTRES1	0.0511889	0.0002277
SFXN3	0.0045678	0.0002313
BACE1-AS	-0.0490312	0.000233
HINFP	-0.0538174	0.0002336
ACP1	0.0125887	0.0002347
TMED8	0.0425614	0.0002348
PSMD6	-0.0124323	0.000235
STRN4	-0.0080799	0.0002354
RNF5	-0.0722062	0.0002356
POLR2E	-0.0306701	0.0002367
UNC13D	-0.0238396	0.0002369
HMOX2	0.020662	0.000237
LAS1L	0.0403692	0.000237
TMEM218	-0.0107287	0.000237
REV1	-0.0036273	0.0002374
SMIM4	-0.0598089	0.0002379
POMGNT1	-0.012223	0.0002384
SLC25A46	-0.0877357	0.0002384
INTS10	0.0169373	0.0002391
FAM20B	-0.0736987	0.0002407
TPCN1	-0.1027122	0.0002416
PDCL	-0.0650504	0.0002434
TRIM68	-0.0103375	0.0002435
TMEM8A	-0.0186569	0.0002436
MPV17	0.02377	0.0002444
MOB3A	-0.056713	0.000245
CDK19	-0.0821839	0.0002453
PIK3R4	0.0123905	0.0002456
ARPP19	-0.1025779	0.0002464
RNF40	-0.0159717	0.0002468
TMEM71	0.1035092	0.0002478
KAT7	-0.079225	0.0002486
PEF1	-0.0019977	0.0002516
ARNT	-0.0413446	0.000252
NSMCE3	-0.0140145	0.0002523

CHPT1	-0.0947207	0.0002527
SLC35D1	-0.0153652	0.0002533
EML3	-0.018407	0.0002537
TBC1D13	0.0346604	0.0002539
ST6GALNAC	0.0827177	0.0002546
ARIH1	-0.0501443	0.0002552
PEX3	-0.0176275	0.0002554
CLIP4	-0.0287511	0.0002561
CEP85	-0.0291866	0.0002566
SLC30A6	-0.0182461	0.0002585
TAF2	-0.017261	0.0002589
DDX20	-0.0364215	0.0002592
SAT1	0.2497833	0.00026
BRD3	-0.0854662	0.0002606
FBXO4	0.0179069	0.0002608
SLBP	0.0960327	0.0002608
COL24A1	0.1238494	0.0002618
OSBPL2	-0.0443208	0.0002619
DYNLL1	0.2156057	0.000262
PPM1M	-0.0513795	0.000264
RBM14	0.0675526	0.0002645
ANKZF1	0.0144912	0.0002651
NUS1	-0.0346274	0.0002657
ABHD18	-0.0425868	0.0002658
RHOG	-0.1180573	0.0002672
TBC1D10C	0.0565201	0.0002677
AGAP5	-0.0283998	0.0002678
ZNF7	-0.0443839	0.0002681
YME1L1	-0.0924928	0.0002685
PCGF3	-0.0381896	0.0002685
TIA1	-0.1325582	0.0002685
RIOK1	-0.0114832	0.0002691
ZNRD1	0.0784095	0.0002697
FASTKD2	-0.0197328	0.0002698
CBX1	-0.0501614	0.00027
C2CD2	0.0202737	0.0002701
IFT20	0.0033053	0.0002706
SPAG16	0.0808616	0.0002719
ASRGL1	-0.0391879	0.000272
MAGED2	-0.0035216	0.0002722
FUT10	0.025161	0.0002729
TMEM184C	-0.0732882	0.0002738
RBBP7	-0.0048176	0.0002743
SPCS3	-0.0159713	0.000275
PLCB1	0.1155335	0.0002773
GALT	-0.0113228	0.0002804
ARHGAP35	0.0010059	0.000281
MGAT1	-0.0185698	0.0002816
AL390728.5	-0.0403299	0.0002819
OTUD5	0.0327575	0.0002826

CAPN1	0.0126819	0.000283
ZXDC	-0.0721853	0.0002834
ERCC3	-0.0199841	0.0002836
DBP	-0.025461	0.0002842
LTBP3	0.0371592	0.0002842
AP2B1	-0.0481354	0.0002843
LSM12	-0.0027975	0.000285
TMA16	-0.0575536	0.0002851
GGA2	0.005413	0.0002851
FAM217B	-0.0328202	0.000287
HSPA13	-0.0224169	0.0002879
RAB6A	-0.0394105	0.0002892
CCNK	-0.013319	0.0002896
FAM122A	-0.0070426	0.0002916
ATP6V1H	-0.0363522	0.000292
PRKCD	-0.0759031	0.0002922
SLC35F2	-0.0281689	0.0002933
TMED9	-0.0092417	0.0002935
ESYT1	-0.1012115	0.0002947
ZBTB5	0.0027317	0.0002953
CUL4A	-0.0607985	0.0002964
DIP2B	-0.0534857	0.0002965
C17orf80	-0.0417849	0.0002967
DPH5	-0.0347442	0.0002976
BOP1	0.0173495	0.0002978
PEX5	-0.0150144	0.000298
PGD	-0.0666969	0.0002981
RPUSD4	-0.0274428	0.0002983
RMDN1	0.0094521	0.0002988
VAMP2	0.0943096	0.0002988
UVSSA	-0.0355434	0.0002989
SAP30BP	-0.0328601	0.0002994
CAT	-0.2302435	0.0003015
FAM106A	-0.0221123	0.0003027
PHKB	-0.0868899	0.0003029
C5orf15	-0.0765188	0.000303
AASDH	0.00814	0.0003031
PDRG1	0.0079924	0.0003031
CEP70	-0.0418348	0.0003042
ACSL3	-0.0609971	0.0003045
SPPL2A	-0.0625732	0.0003103
HAUS2	-0.0395122	0.0003118
CCDC126	-0.0341619	0.0003123
ATXN3	-0.0856319	0.0003136
SMARCB1	-0.0677596	0.0003142
FGD5-AS1	-0.0468045	0.0003144
TMEM134	0.0964993	0.0003169
UMAD1	-0.0089913	0.000318
CTNNAL1	-0.0052541	0.0003183
OSBPL9	-0.0081535	0.0003188

ZNF689	0.0110604	0.0003194
SETD5	-0.0643896	0.0003199
ERI1	0.0510743	0.0003202
RNF168	-0.047049	0.0003206
GTF2F2	0.0288133	0.0003207
TBC1D23	-0.029966	0.0003212
RABGAP1	-0.0126345	0.0003214
TTYH3	-0.0517717	0.0003219
ACPP	-0.0030787	0.0003235
GALNT4	0.0661626	0.0003236
COPZ1	-0.0109794	0.0003246
TTBK2	-0.0529024	0.0003254
RNF167	0.0843728	0.0003255
SUSD1	-0.040611	0.0003257
DEGS1	-0.0368461	0.000326
GPR89A	-0.0614349	0.000326
DPY19L3	-0.0647363	0.0003264
ANAPC15	0.0301289	0.0003269
ADGRA3	-0.041938	0.0003275
NDUFB6	-0.0390031	0.0003293
VIRMA	0.0422787	0.0003294
QPCTL	-0.0183702	0.0003296
ABHD13	-0.0196049	0.0003297
HDAC5	0.0122796	0.000333
SAMD1	-0.0730498	0.0003334
NRSN2-AS1	0.0216882	0.0003339
GFI1	-0.0599917	0.0003345
MCM2	0.0613701	0.0003346
IKZF1	-0.1780535	0.0003351
TMED2-DT	-0.0368918	0.0003367
ASB7	-0.0256079	0.0003371
RUFY1	0.1430592	0.0003382
CLK4	-0.0958864	0.0003396
TERF2IP	0.1362265	0.0003423
CPNE2	0.0993901	0.0003425
C9orf43	0.0307527	0.0003449
POLR3A	-0.0205627	0.0003458
EAPP	0.0090416	0.0003462
RTN4	0.1686786	0.0003471
CCDC18-AS	-0.0036776	0.0003472
LETMD1	-0.0788458	0.0003486
STRADA	-0.0362619	0.0003488
FBXL15	0.0759193	0.0003488
USP9X	-0.0504623	0.0003497
GBP3	-0.0492726	0.0003506
ZNF493	-0.1327611	0.0003506
AP1S2	-0.1536997	0.0003507
LRCH3	-0.072044	0.0003509
AHCY	0.0309297	0.0003511
NKIRAS2	-0.0077504	0.0003519

XPOT	-0.11155	0.0003537
UBE2L3	0.0937672	0.0003537
HAUS3	-0.0330094	0.000355
FAM120AOS	0.0027756	0.0003552
XRCC6	0.0568602	0.0003559
ATP11B	-0.0698354	0.0003565
PMS1	0.0319701	0.0003577
SDF2L1	0.0674421	0.0003579
PIK3CG	0.0514315	0.0003609
SMU1	-0.0520341	0.0003611
OSGIN2	-0.0462538	0.0003615
COA8	0.0550055	0.0003618
AC093627.4	-0.0298125	0.0003622
NUDT21	0.0165619	0.0003623
CAPZB	-0.0458086	0.0003624
NDUFB2	0.1237466	0.0003626
EBPL	0.1105798	0.000363
TPGS2	0.0111267	0.0003635
TMEM126B	-0.0350369	0.0003639
GTF3C3	-0.0287784	0.0003644
VPS16	-0.0038919	0.0003663
STRIP1	-0.022167	0.0003666
C1orf216	-0.0510776	0.0003681
STAP1	0.1055436	0.0003696
B4GALT4	-0.0165345	0.0003699
AHDC1	0.0717423	0.0003708
ZSCAN12	-0.034702	0.0003708
DCXR	-0.0539232	0.0003711
AGO4	-0.034545	0.0003726
PFDN6	-0.0033586	0.000373
WDR43	-0.0321055	0.0003734
PHF1	-0.0078307	0.0003744
UBIAD1	0.0395104	0.0003748
KLF3-AS1	0.0349529	0.0003754
ATP11C	-0.0105809	0.0003772
IFT52	-0.0527178	0.0003772
TAMM41	-0.0273695	0.0003782
CDK5	-0.0100192	0.0003793
OMA1	-0.0956123	0.0003801
THUMPD3	-0.0183954	0.0003802
POLR2G	-0.0661708	0.0003802
ING4	-0.0556685	0.0003815
SRCAP	-0.0249452	0.000383
DNAJC19	0.0834923	0.0003833
IFT43	0.015408	0.0003846
IKBKE	0.0327255	0.0003851
ILKAP	-0.0747238	0.000386
FEM1B	-0.0331285	0.0003867
AC245060.6	-0.0176006	0.0003881
AC103702.1	-0.1301787	0.000389

POLR3C	-0.0523221	0.0003922
LLPH	-0.1464356	0.0003923
LRPPRC	-0.0726401	0.0003929
NOCT	-0.0499297	0.0003958
NANP	0.0118653	0.0003966
MRPL34	-0.0413337	0.0003977
PLSCR3	0.0006154	0.000398
LRRC58	-0.0713189	0.0003987
VAV3	0.0546544	0.0003991
TMTC4	-0.0252273	0.0004
ZNF429	-0.0482819	0.0004011
COPS3	0.0496515	0.0004026
GSEC	-0.0512791	0.0004036
DDX58	-0.0462204	0.0004056
CTDSPL2	-0.0056438	0.0004063
GLT8D1	-0.0137581	0.0004072
KEAP1	-0.0106051	0.0004073
AL096870.8	-0.0806701	0.0004078
ME2	0.0147054	0.0004079
ZNF417	-0.0492522	0.0004083
ZNF3	0.0276138	0.0004086
ZFYVE27	-0.0411255	0.0004098
PPP2CB	-0.0089356	0.0004103
ZNF117	-0.0849301	0.0004107
DDX19B	-0.026322	0.000413
MED6	-0.0037092	0.0004134
TPRA1	-0.0141526	0.0004139
MRPL2	-0.0099073	0.0004152
ARID3A	-0.1299404	0.0004162
PMPCB	-0.0550818	0.0004165
IDH3G	0.0130676	0.0004166
ERLIN2	0.0261636	0.0004171
CDK17	-0.011849	0.0004174
ILVBL	0.0231444	0.0004174
SMARCAD1	-0.0615507	0.000418
ANO6	-0.0623696	0.0004195
CCDC180	-0.0174152	0.0004198
CASP3	0.0029044	0.0004226
PPP1R3F	-0.0139132	0.0004246
RSBN1L	0.1471583	0.0004273
OGFR	-0.0706992	0.0004283
HIP1R	-0.0257043	0.000429
TMOD3	-0.030014	0.0004299
RGL2	0.0732438	0.0004299
IRF2BP1	-0.0082661	0.0004304
SMS	0.0817915	0.0004312
ATP6V1A	0.0225795	0.0004337
AC016876.1	-0.0280666	0.0004345
TMPO	0.1339467	0.0004357
GLRX5	0.0326372	0.0004361

PLEKHF2	-0.035126	0.0004366
KRBA2	0.0358038	0.0004369
ZNF776	-0.0112566	0.0004392
ATG101	0.0454928	0.0004393
NT5C3A	-0.0783774	0.0004419
DFFB	-0.0185347	0.000442
TAS2R4	-0.0444317	0.0004422
SIKE1	-0.0519417	0.0004431
ADD1	0.0553802	0.0004446
ZNF709	0.0092802	0.0004466
D2HGDH	-0.0113401	0.000448
ZBTB37	-0.0205758	0.0004488
AC011477.2	-0.0229447	0.0004498
NAPEPLD	-0.0532733	0.0004508
BAD	0.0101404	0.000451
MTFR1L	0.0207113	0.0004515
NOLC1	0.0537784	0.0004527
MIF4GD	0.0027909	0.0004534
KIF9	0.0870616	0.0004572
ANTKMT	0.0975396	0.0004591
CDC16	-0.0339391	0.0004603
AGFG1	-0.0375579	0.0004617
ZNF674-AS1	-0.0262873	0.000462
ZDHHC24	0.061168	0.0004629
SHARPIN	0.0052868	0.0004631
CETN2	-0.0447191	0.0004633
ATXN10	-0.0095455	0.0004642
IARS	0.0255035	0.0004644
TSPAN4	0.0923791	0.0004648
FIBP	0.0952722	0.0004675
ACLY	-0.0331224	0.0004703
CMPK1	-0.104866	0.0004709
SATB1	-0.0190703	0.0004731
DCUN1D4	-0.0267622	0.0004741
ZKSCAN4	-0.0347008	0.0004752
IMPA2	-0.0102382	0.0004753
CRYBG1	0.1147867	0.0004763
TAPT1	-0.0363799	0.0004764
POP7	0.0341716	0.0004777
ZSWIM7	-0.1048328	0.0004788
POGLUT1	-0.0500631	0.0004804
ITFG1	-0.0476568	0.0004806
ADK	-0.158463	0.0004816
OS9	-0.0972422	0.0004828
RPA1	0.0129092	0.0004832
NFIX	-0.0212781	0.0004867
WDTC1	-0.0221216	0.0004878
FERMT3	0.1289079	0.0004883
RNASEH2A	-0.0317859	0.0004902
PTRH1	0.0445573	0.0004902

WDR48	0.0063423	0.0004944
SSH3	-0.0113772	0.0004991
PPP6C	-0.0123933	0.0005039
PKN2	-0.0662489	0.000508
SFT2D1	-0.0565039	0.0005085
CAMTA1	0.0184157	0.0005086
DISC1	0.0207517	0.0005106
PEPD	-0.0203253	0.0005108
SPAG7	0.029152	0.0005113
PRR11	-0.0503963	0.0005119
RHBDD2	-0.0468957	0.0005121
ABHD17A	0.0672019	0.0005141
AC006064.4	-0.0214638	0.0005142
MFSD4B	-0.1241799	0.0005153
LYSMD2	-0.0965679	0.0005185
AL031595.2	-0.035323	0.0005198
WASL	0.025176	0.0005198
BCL11A	0.1822101	0.0005203
HMGXB3	0.0481935	0.0005206
HRH2	0.1101575	0.0005214
POM121C	-0.0235327	0.0005225
NFE2L2	-0.1184602	0.0005226
SKP1	-0.1391512	0.0005235
TTLL4	-0.0162717	0.000525
ENTPD1-AS	0.0156921	0.0005277
METTL21A	-0.0018737	0.0005312
FAM174C	0.047694	0.0005332
AL121933.2	0.0625475	0.0005333
CCNH	-0.0363624	0.0005335
LARP4B	-0.0858365	0.0005366
DNAJC15	0.1157415	0.0005373
SDF2	-0.0140782	0.0005391
ZNF24	0.0160036	0.0005404
RNF123	-0.0348718	0.0005412
RBM17	0.0772918	0.0005429
CAVIN2	0.1444694	0.0005477
SCAMP4	-0.0148664	0.0005479
CBFA2T2	-0.0762929	0.0005488
PAGR1	0.0238018	0.0005538
MFSD8	-0.0603662	0.0005551
TMX1	-0.0254504	0.0005559
PLPP6	-0.0358161	0.000556
GPSM1	0.0825	0.0005574
ZNF121	-0.0180613	0.0005577
MXI1	0.0189192	0.0005593
ZDHHC9	0.0267932	0.0005656
ALAD	-0.0529625	0.0005661
PRKAR1A	-0.0979161	0.0005674
QARS	-0.003217	0.0005684
ILF3-DT	0.017771	0.0005707

ZC3H12C	0.0466716	0.0005728
PMS2	-0.0959517	0.0005736
TRAPPC2B	0.0508952	0.0005765
BST1	0.0823886	0.0005809
AL499604.1	0.1024255	0.000584
TAS2R14	-0.040341	0.0005861
CEP120	-0.0654462	0.0005863
RPN1	-0.0040145	0.0005874
MALT1	0.0078085	0.0005881
MFSD14B	-0.0474834	0.0005886
CDYL2	0.0020494	0.0005887
APOBEC3G	0.0262648	0.0005896
PPAN	0.0345893	0.0005907
SLC2A5	-0.1037693	0.0005923
RAVER1	0.0110529	0.0005927
HYMAI	0.0714187	0.0005935
FOXP1-IT1	0.0294553	0.0005938
INTS4	-0.0434914	0.0005961
SLC27A5	0.0653818	0.0005968
ZNF579	0.0315615	0.0006022
SGPL1	-0.0055249	0.0006023
TBCCD1	-0.0328902	0.0006062
RPF1	-0.0091303	0.0006063
SLC38A7	-0.0239954	0.0006067
NEPRO	-0.0549071	0.0006094
FAM50B	0.0369689	0.0006094
SUCLG2	-0.0287897	0.0006114
WTAP	-0.0792194	0.0006115
UBXN2B	-0.075376	0.000612
TSPOAP1	-0.0224938	0.0006142
TP53BP2	-0.0212415	0.0006162
TAF1D	-0.2184563	0.000618
ZNF330	-0.0321743	0.0006181
UBE2E1	-0.0038568	0.0006182
HCLS1	0.0592443	0.0006188
SLC25A17	-0.0238782	0.0006198
TAF1	-0.0723493	0.0006207
WDR6	-0.0281121	0.0006208
TUBD1	0.0058433	0.000622
POLR2A	-0.0532001	0.0006228
CYP20A1	-0.0482133	0.0006228
MBOAT7	0.1383727	0.0006254
MTCL1	-0.0450953	0.0006265
ZC3H3	-0.0216528	0.0006275
RNF138	-0.0126968	0.0006326
CENPH	0.0530227	0.0006351
DEAF1	-0.0065493	0.0006364
RWDD4	0.020535	0.0006366
ZNF785	-0.0886773	0.000637
CPTP	0.0029396	0.0006374

SH2B3	0.0209501	0.0006378
KIF21B	-0.0333688	0.000638
SPTSSA	-0.0901439	0.0006397
BACH1	-0.0372254	0.0006402
TMLHE	0.0091328	0.0006424
USP25	-0.0367185	0.0006427
NADK2	-0.0437614	0.0006429
TEC	0.1476935	0.0006495
DYNLL2	0.0215116	0.0006506
L2HGDH	-0.0543244	0.0006571
ANKHD1	-0.0912803	0.0006585
INPP4B	0.1357744	0.000659
BRCC3	0.0388846	0.00066
CKAP5	0.0044505	0.0006621
HMGCL	-0.0234193	0.0006623
B3GNTL1	-0.0294359	0.0006634
LRBA	0.0691376	0.0006639
TMEM35B	0.0102999	0.0006646
STX6	-0.0332981	0.0006656
AP5M1	-0.0879588	0.0006657
FNDC3A	-0.0283402	0.0006663
GPX7	0.0491384	0.0006697
TTLL3	-0.0013408	0.0006703
FAM98A	0.0106605	0.0006714
KRBOX4	-0.0277631	0.0006716
CEP44	-0.0376579	0.0006718
SMAD5	-0.0980404	0.0006733
ZNF500	-0.0072734	0.0006734
TGIF2	0.0478203	0.0006747
FANCF	0.0126684	0.0006772
SH3KBP1	-0.1560361	0.0006842
PTGIR	0.0771505	0.0006846
PBDC1	0.0503451	0.0006852
CCDC6	0.0294913	0.0006866
LTBR	-0.0360789	0.0006905
PCNX1	-0.1051645	0.000692
TM9SF1	0.030615	0.0006926
DDX5	0.1907904	0.0006928
P2RY11	-0.1003857	0.0006948
NPLOC4	-0.0371487	0.0006959
ZNF639	0.0140193	0.0006962
FLII	-0.0334147	0.0006971
BCCIP	-0.0444592	0.0006976
SPIDR	-0.0394017	0.000698
CTBP2	-0.0175149	0.000698
RITA1	0.0083333	0.000698
GPM6B	-0.0208665	0.0006994
CYREN	-0.044955	0.0006997
YARS2	0.0076695	0.0007
ZNF281	-0.0379414	0.0007022

IMP4	0.052146	0.0007028
RPS6KA4	-0.0158618	0.0007059
CRYL1	0.093009	0.0007078
TFB2M	0.0582021	0.0007114
SYAP1	-0.036106	0.0007157
HSPA4	-0.021159	0.0007164
TMEM167B	-0.0197613	0.0007202
SNRK	-0.0300615	0.0007252
DHPS	-0.0148149	0.0007269
CCDC90B	0.0210864	0.0007272
SEC23IP	-0.0277946	0.0007274
MAP10	0.0039532	0.0007281
RBBP6	0.1292945	0.0007281
RPS6KA1	-0.0638519	0.0007288
CASP1	0.044061	0.0007296
RAB7A	-0.0250438	0.0007337
PLA2G4A	0.0770993	0.0007337
NTN1	0.0713905	0.0007378
NEK9	-0.0075552	0.0007392
STARD7	-0.0694393	0.0007414
IFT27	0.0014554	0.0007458
PCF11-AS1	-0.0048894	0.0007468
EBLN2	-0.014444	0.0007483
ERCC6	-0.0427502	0.0007512
DUSP22	-0.0162018	0.0007534
MRPL55	0.055482	0.0007556
NUDT9	0.0206923	0.0007596
PGM2L1	-0.0070696	0.0007607
LPGAT1	-0.0525766	0.000762
mars-02	0.0070155	0.0007677
POR	-0.037056	0.0007682
LSM10	0.0350512	0.0007723
AGPAT2	0.02562	0.0007733
PRKAA1	0.0656262	0.000774
ARMC7	-0.0437956	0.0007773
CAPNS1	-0.033731	0.0007793
AKAP11	-0.0197876	0.0007802
TPP2	-0.0353142	0.000781
KIFAP3	0.0242167	0.0007833
GIT2	-0.045857	0.0007836
TADA2A	0.0106432	0.0007846
BRD1	-0.0379907	0.0007855
DNAJC13	-0.0285395	0.0007866
GTF3C2	-0.0139485	0.0007886
SIGIRR	0.00508	0.000795
PPP4R3A	-0.0882435	0.0007966
ZNF585B	-0.0227211	0.0007981
PHF10	-0.0038079	0.0007981
FUBP3	-0.0609195	0.000801
PPP1R8	0.0060013	0.0008011

RAB40C	-0.0547025	0.0008019
CYB5A	-0.0407705	0.0008019
ZBTB11	-0.0292792	0.0008026
POP4	-0.0187147	0.0008049
SLF1	0.0421934	0.000805
RPS15A	-0.1225713	0.0008098
BEND6	-0.0702996	0.0008109
GNB1	-0.1435796	0.0008111
COPA	0.0077476	0.0008124
CBWD3	-0.0721266	0.000817
TIGD5	0.0084416	0.0008181
ATP6V0D1	-0.0508699	0.0008182
ADRM1	-0.0027728	0.0008207
ING3	-0.0636356	0.0008262
MPRIP	-0.0380085	0.0008267
ARMC1	-0.0132143	0.0008288
ZNF154	-0.0367267	0.0008291
PEX13	-0.0022472	0.0008302
INTS7	0.0021612	0.0008319
ZNF333	-0.0447933	0.0008335
ATG4B	-0.026116	0.0008343
MOB1A	-0.1332139	0.0008346
ATAD3A	0.0512937	0.0008356
EMC9	0.0111753	0.0008358
MORF4L2	0.0158823	0.0008359
GNL1	0.0688729	0.0008461
MRPS25	0.0456416	0.0008466
F8A1	-0.0323127	0.0008497
LTB4R	0.0591314	0.0008512
TMEM259	-0.0308204	0.0008525
MIF	0.2390371	0.000854
ZNF675	-0.032028	0.0008547
TMEM217	0.0842114	0.0008594
KCTD10	-1.868E-05	0.0008618
CRCP	0.0289169	0.0008619
PACC1	-0.0319051	0.0008621
TRIP12	-0.0851517	0.0008631
BLOC1S6	-0.113032	0.0008662
NUDT16L1	-0.0536383	0.0008699
LMNB2	0.0179952	0.0008709
SNX10	-0.0970817	0.0008743
SHQ1	0.0070017	0.0008757
BAZ2A	-0.0514072	0.0008762
ELMSAN1	-0.0967235	0.000877
SLC38A9	-0.0428123	0.0008784
ZNF550	-0.0031682	0.0008794
FAM78A	-0.0379464	0.0008837
SDHAF2	0.0181668	0.0008846
NTPCR	-0.0306659	0.000886
ZNF641	-0.088874	0.0008871

CCS	0.0542868	0.0008922
DTX3L	-0.0072503	0.000896
C17orf67	-0.0628038	0.0008964
TRA2B	0.2005804	0.0008989
SNHG9	-0.0501185	0.0009036
NSUN3	-0.0448013	0.0009041
NOP2	0.0069332	0.0009047
PGAP2	0.015511	0.0009096
SCO2	0.0535423	0.0009131
DIAPH1	0.0558141	0.0009133
ZNF667-AS1	0.039706	0.0009171
NDUFAF5	-0.0447784	0.0009178
EPHB6	-0.0196881	0.0009208
DHX15	-0.0437947	0.0009212
RPS16	0.1273935	0.0009227
AC139887.2	-0.0269129	0.0009238
ZNF576	0.0486612	0.0009301
MDFI	0.1077555	0.0009302
PRIMPOL	-0.0071319	0.000931
CXorf56	-0.035103	0.0009349
PRPF31	0.043526	0.0009359
ATG3	0.0563964	0.000936
SRBD1	0.0019567	0.0009367
ZBTB40	-0.0305537	0.0009395
MR1	-0.0125199	0.0009398
THAP7	0.0609992	0.000941
AXIN1	-0.0416347	0.0009416
TXK	0.0333524	0.0009455
ARL16	0.0324768	0.0009475
AL035071.1	-0.0341056	0.0009502
PTCD1	-0.0239942	0.0009554
ZNF852	-0.0125203	0.0009564
EAF1	0.0162481	0.0009598
LARP1	-0.1212355	0.000961
FKBP11	0.1077807	0.0009666
COQ8B	0.0094053	0.0009668
NFU1	-0.0537054	0.0009731
CCDC144A	-0.1303514	0.0009746
ARID3B	0.0013043	0.0009766
STAMBP	-0.0365703	0.0009783
TOR3A	-0.0305194	0.0009802
ATM	-0.0738335	0.0009828
FOXRED1	-0.0089696	0.0009832
DIMT1	-0.0333905	0.0009866
ALG10B	-0.0588814	0.0009917
FNBP1	-0.1345115	0.0009929
ZNF585A	0.0312737	0.001001
PRKRA	-0.0242413	0.001004
NKX2-3	0.0224036	0.0010085
PIGM	-0.0870568	0.0010121

WRNIP1	-0.0593477	0.0010255
LYRM2	-0.0279622	0.0010299
FAM3A	0.0095467	0.0010313
CHPF2	0.0024472	0.0010313
STRN3	0.0086248	0.0010396
ZNF227	-0.0065732	0.0010476
DCTN3	0.0922852	0.0010487
PEX2	-0.0506822	0.0010495
AC002558.3	-0.0140968	0.0010574
BOD1L1	-0.0923824	0.0010579
UTP6	-0.0742686	0.00106
YIF1B	0.0301257	0.0010622
MTX1	0.0172322	0.0010772
VPS11	-0.0423248	0.0010773
CD68	0.1270286	0.0010809
ELOVL5	-0.0618644	0.0010809
MRPL50	0.0539738	0.0010822
GPAT3	0.14891	0.0010836
POLR2J3	-0.1619148	0.0010838
DUSP3	0.070147	0.0010859
LONP2	-0.0527722	0.0010868
ANAPC10	-0.0221332	0.0010918
EIF2B5	-0.0194532	0.0010932
MRPS7	0.0951289	0.0010945
ASNSD1	-0.0249816	0.001097
PARL	-0.0360214	0.0010971
ROCK2	0.0262794	0.0010995
PITPNB	0.001681	0.0011002
DERL1	0.0094012	0.0011006
PNKP	0.0642427	0.001101
AC009133.1	-0.0273052	0.0011049
NUP98	-0.0482296	0.0011052
SRRT	0.02466	0.0011057
PDE6D	-0.034875	0.0011067
C15orf40	-0.0361171	0.0011149
FDX2	0.0680373	0.0011163
ICE1	-0.0395933	0.0011182
C12orf76	0.0053123	0.0011195
SS18L2	0.0264871	0.0011203
AFTPH	-0.0222288	0.0011218
DGKA	0.1104215	0.0011228
TENT2	-0.0664394	0.0011233
TPT1-AS1	-0.0719112	0.0011287
TM7SF2	0.0539557	0.0011292
MGST3	0.2138905	0.0011321
KLF10	-0.0896048	0.0011376
B3GNT2	0.0614377	0.0011394
PHTF1	-0.0593547	0.0011448
KHDC4	-0.085271	0.0011477
POLR1B	0.0058706	0.0011486

MED21	-0.0788173	0.0011489
WASHC2C	-0.1153938	0.0011541
DAPP1	0.0666939	0.0011575
MLLT10	-0.0457836	0.0011576
AC097448.1	-0.0353499	0.0011579
SLC7A6	0.0439306	0.0011611
WDR11	-0.1265832	0.0011655
STK10	0.0651945	0.001169
NOL7	0.1497587	0.0011723
ITPA	0.0496785	0.0011757
SLC20A1	-0.0042083	0.0011851
DNAJB2	-0.0259324	0.0011865
SLU7	-0.0206835	0.0011898
DBNDD2	0.092423	0.0011904
JMJD8	0.0216572	0.0011952
EXOC3	-0.127879	0.0011971
TRAPPC6B	-0.0853178	0.0011999
IDI1	0.0240728	0.0012021
YIPF5	-0.0226401	0.0012022
ACSL5	0.0058627	0.0012028
IDUA	-0.0403217	0.0012047
UBN2	-0.1033226	0.0012047
KYAT3	-0.0340168	0.0012065
OSBPL5	-0.0593842	0.0012127
TRAP1	0.054556	0.0012165
UPF2	0.0637642	0.0012205
MED10	0.0644367	0.0012248
MARK2	-0.0498146	0.0012258
TBC1D9B	-0.0433801	0.0012278
FKBP1A	0.0342581	0.0012297
HMGB2	0.1218249	0.0012312
CLSTN3	-0.0174025	0.0012325
NBN	0.0740268	0.0012325
MCPH1	-0.0491347	0.0012339
LRP8	0.0708476	0.0012352
PTRHD1	-0.0041335	0.0012379
PNKD	0.0471949	0.0012423
ELAVL1	0.0019388	0.0012441
DNAL1	-0.0332555	0.0012461
SUPV3L1	0.0075666	0.0012468
DOK1	0.0234706	0.0012501
ZNF414	0.0048787	0.0012532
CBFA2T3	0.0810169	0.0012592
FANCM	-0.0166685	0.0012598
TRAPPC12	-0.017586	0.0012644
GPBP1L1	-0.100432	0.0012652
MRPL52	-0.0341667	0.0012674
SIL1	0.0696156	0.0012684
RFNG	0.0145233	0.0012715
CHTOP	-0.0001125	0.0012792

SRPRB	-0.0175848	0.0012795
BNIP2	0.0952845	0.0012806
DDX54	-0.0003072	0.0012828
GZF1	-0.0046988	0.0012866
SEPTIN1	0.033252	0.0012879
DHRSX	-0.0466378	0.0012879
TP53I13	0.0578954	0.0012883
NF2	-0.000449	0.0012904
ZBTB43	-0.0136863	0.0012906
PFKP	0.1097981	0.0012912
AKR1B1	0.1089402	0.0012939
PDPK1	-0.0200651	0.0012946
THOC6	0.0090687	0.0012974
KRT17	-0.0922459	0.0012975
CARF	-0.0754565	0.0013014
TMEM115	0.0204736	0.0013048
FAM177A1	-9.37E-05	0.0013066
PCYOX1L	0.0134832	0.0013076
MRPL13	0.0930401	0.0013083
SMIM19	-0.0340557	0.0013096
PRORP	0.032229	0.0013101
CNP	-0.0166237	0.0013109
PM20D2	0.1347124	0.0013114
PATZ1	-0.0073759	0.001319
WEE1	-0.0158377	0.0013191
SHKBP1	-0.0151965	0.0013203
TSPYL1	-0.0946119	0.0013229
VARS	0.025909	0.0013237
EID3	-0.0237415	0.0013244
MRPL18	0.1044693	0.0013254
DTD2	-0.0052945	0.0013264
EDEM2	-0.0080202	0.0013287
TRMO	0.0099711	0.0013291
MYBBP1A	0.0048341	0.0013292
CLK2	0.0243717	0.0013292
SHFL	-0.0351339	0.0013302
ZCCHC9	-0.0114079	0.0013334
STX4	0.0672953	0.0013359
ORC6	0.0531671	0.0013367
AC245014.3	0.1041864	0.0013376
ELMO1	-0.0537758	0.0013422
PITPNM1	-0.0296155	0.0013462
TLE5	0.1128793	0.0013514
ANGPTL6	-0.0048886	0.0013541
MARCH1	0.1180199	0.0013555
NEK7	-0.0385263	0.0013585
PRXL2C	0.0038351	0.0013595
UTP15	0.0054889	0.0013628
YPEL5	0.0736194	0.001367
GMFB	-0.0379691	0.0013685

SUGP1	-0.0001146	0.0013702
CNOT10	0.0145872	0.0013702
BCOR	-0.0966225	0.0013771
TAF15	0.0013863	0.0013827
MTREX	-0.0303776	0.0013839
PHC3	-0.0310571	0.0013856
PHC1	0.0171101	0.0013898
TOM1L2	0.00495	0.0013915
TMEM260	-0.039084	0.0013987
GFM1	-0.0449798	0.0013991
SETDB1	-0.0233898	0.0014011
TRIQK	0.0217635	0.00141
ALDH3B1	-0.0077438	0.0014126
TGS1	0.0228999	0.0014145
ACBD5	-0.0753602	0.0014169
SMYD4	-0.0150676	0.0014186
NOSIP	0.0170431	0.0014196
SELENOW	0.1703888	0.0014198
DCAF12	0.0416799	0.0014199
TMEM70	0.05363	0.0014241
ITGB2	0.1274562	0.0014297
KRI1	0.0319222	0.0014312
ATG13	-0.0558099	0.0014324
AC016735.1	-0.014036	0.0014352
KDM2A	-0.0447575	0.0014458
RPA2	-0.0800263	0.0014458
KXD1	-0.0385183	0.0014461
GNAI1	0.1890388	0.0014526
PPA2	-0.0175386	0.0014526
ARMC10	-0.1027972	0.001454
COPG2	-0.0418595	0.0014551
VPS13B	-0.0113541	0.001458
AL157392.3	-0.0657948	0.0014586
COMMD5	-0.0333311	0.0014591
ABHD8	-0.0401031	0.0014765
TSTA3	0.1161134	0.001484
ACOT2	-0.0215254	0.0014895
TOR4A	-0.0367294	0.0014906
AL513550.1	0.0077728	0.001491
C1QTNF4	0.1643549	0.0014918
ZKSCAN2-D	-0.0604207	0.0015017
AC104083.1	-0.0037567	0.0015066
CALHM6	0.1602097	0.001509
TRIO	0.0163006	0.0015155
AL035587.1	-0.0246528	0.0015223
RSU1	-0.0724356	0.0015286
RRS1	0.0482769	0.0015309
LDB1	-0.0113538	0.0015347
CYB5B	-0.0397981	0.0015364
RER1	0.0422051	0.0015375

SLC25A32	-0.0114111	0.0015383
ZSCAN16-A:	-0.1094216	0.0015394
TTC5	0.0023926	0.0015418
BRAT1	0.0128489	0.0015459
EPS15L1	-0.0777387	0.001552
DDX28	0.0216417	0.0015586
MADD	-0.0728103	0.0015597
IKBKB	-0.038438	0.0015605
NT5C2	0.001078	0.0015663
ZNF451	-0.0642129	0.0015705
COQ9	0.0302687	0.0015756
LINC00996	-0.1199154	0.0015757
RBM48	0.0078869	0.0015772
MRPL3	0.0494791	0.001578
AC211476.6	-0.0422128	0.0015833
CERT1	-0.010302	0.0015858
CEP104	-0.0368397	0.0015888
UBA7	0.009493	0.0015891
KBTBD7	-0.0194462	0.0015906
HOXB3	-0.1481684	0.0015927
CHD6	-0.0316147	0.0015931
C16orf70	-0.016916	0.0015941
ZNF619	-0.0036304	0.0015961
METTL4	9.226E-05	0.0015997
DRG2	0.0200255	0.0016025
PIGT	0.0378829	0.0016043
TBC1D22A	-0.0274586	0.0016079
SLC7A6OS	0.0210038	0.0016087
PSMG2	-0.0418451	0.0016095
ARF6	-0.031679	0.0016175
BTK	-0.0247256	0.0016225
PLCB2	0.0005053	0.0016228
DYNC1LI1	0.0293407	0.0016353
GSTM4	0.0507156	0.0016443
TIGAR	-0.0283192	0.0016485
ZSWIM1	-0.0490374	0.0016529
PACRGL	-0.02685	0.0016584
PICALM	-0.1082555	0.0016626
TTC17	-0.0490408	0.0016757
SEC23A	-0.0526516	0.0016793
HIRA	-0.0036742	0.0016808
HCCS	0.009741	0.0016865
TIMM10	0.0354013	0.0016879
UBR3	-0.0412364	0.0016892
VCP	0.0128034	0.0016944
ZNF621	-0.0240165	0.0016952
PHB2	0.1092414	0.0016987
SMAP1	-0.0609455	0.0017003
TMEM94	0.0377413	0.001704
RAB11FIP4	-0.0352327	0.0017049

CNN2	-0.0563283	0.0017111
PORCN	-0.0184109	0.0017152
MANBAL	0.0176084	0.0017159
SRPK1	0.0289212	0.0017214
POLR2J2	-0.005379	0.0017219
ZNF736	-0.0562726	0.0017235
APOL1	-0.0325592	0.0017278
CCDC28B	0.0841188	0.00173
USPL1	-0.0207886	0.001731
ACOT8	-0.0073197	0.0017323
CEP128	-0.0715846	0.0017324
RAB1B	-0.0003582	0.0017344
MTX2	-0.0205475	0.0017355
NCOA6	-0.0441519	0.0017366
DERL2	0.0003515	0.0017371
A1BG	0.1162979	0.0017394
SRFBP1	0.0108135	0.0017499
ABHD14B	0.0944091	0.0017637
COA7	0.0337931	0.0017735
TUBB6	0.1084251	0.0017773
EIF2B1	-0.0223946	0.0017798
THUMPD1	-0.1824269	0.0017808
ZBTB1	-0.058671	0.0017822
GOLGA7	-0.0064349	0.0017892
KLHL20	0.0117229	0.00179
MTMR6	-0.0220675	0.0018041
VAPA	-0.1991218	0.0018096
ERGIC3	-0.0516223	0.001816
UBFD1	0.0161193	0.0018203
ZNF407	0.0159871	0.0018228
CCAR2	-0.0206122	0.001824
DYRK4	0.0129851	0.001826
PUSL1	0.0041835	0.001829
ODF2	-0.058745	0.0018302
GUF1	-0.0247556	0.0018352
AGPAT5	0.0483176	0.0018502
DVL3	-0.0737185	0.0018519
AC090630.1	-0.0360203	0.0018532
TRAF3	-0.069333	0.0018576
TMEM205	0.1186025	0.0018634
MTF2	0.0833228	0.0018669
TP53RK	-0.0245602	0.0018748
SATB1-AS1	0.0175965	0.001876
ZNF510	0.0018733	0.0018811
TMEM18	-0.0399241	0.0018814
JPT2	0.0166255	0.0018829
ZNF649	-0.0034289	0.0018903
AVL9	-0.0678004	0.0019034
OSCAR	0.0135495	0.001905
DRAP1	-0.2009239	0.001905

STAT5B	-0.0305908	0.0019105
BROX	-0.1078319	0.001915
MSRB2	0.1371709	0.0019152
GNA13	-0.0287438	0.0019162
PREP	-0.0423039	0.001921
HADHB	0.0078838	0.0019222
CIAPIN1	-0.0072775	0.0019236
EMC3	-0.0158513	0.0019424
VPS4A	0.0394806	0.001957
ZFPL1	-0.0117182	0.0019612
PRIM1	0.0904667	0.0019662
RPS4X	0.1241826	0.0019789
NBR2	-0.0387595	0.0019809
WFDC1	0.065972	0.0019837
CFAP410	-0.0078467	0.0020024
DDB1	0.0284985	0.002013
NUP93	-0.0330565	0.0020163
AC019205.1	-0.0734705	0.0020165
IDH2	0.0127527	0.002027
LRRC57	-0.0594773	0.0020315
SMIM3	0.1417695	0.0020326
NNT	-0.0629403	0.0020359
C11orf21	0.0972217	0.0020373
TAF9	0.0625849	0.0020387
AGO3	-0.1751651	0.0020413
TET2	-0.1270399	0.002047
JAG1	0.102087	0.0020478
AC127502.2	-0.0391731	0.0020543
SLC35B1	-0.042965	0.0020573
PIGH	-0.0295846	0.0020609
IBTK	-0.0524194	0.0020644
ZNF469	-0.0352463	0.0020659
TMED10	0.0171741	0.0020788
NGRN	-0.0200267	0.0020791
USP36	0.033091	0.0020799
PSMB6	0.0778247	0.002085
TMEM126A	0.0670112	0.0020852
WDR1	0.0691938	0.0020878
NDUFB7	0.1365146	0.0020918
PRKCSH	-0.056918	0.0020939
DYM	-0.0308583	0.0021066
ABITRAM	-0.0324905	0.0021102
SULT1A1	-0.0246413	0.0021152
CLK1	-0.0798611	0.0021152
GNL2	-0.0349836	0.0021155
LANCL1	-0.0111678	0.0021231
IDNK	-0.0647665	0.0021254
BRCA1	0.1017173	0.0021269
ATAT1	-0.0148169	0.0021276
TRPT1	-0.05803	0.0021289

ZNF44	-0.0756797	0.0021312
PDLIM5	-0.0635187	0.0021323
CINP	0.0475647	0.0021332
ANKRD10	-0.0331043	0.0021343
AC087190.1	-0.0080848	0.002138
PNPLA2	-0.0284007	0.002142
NXT1	0.0120309	0.0021498
CFAP20	0.0326865	0.0021567
NUBP1	0.0049687	0.0021627
METTL25	-0.0263417	0.0021721
TJAP1	-0.0296165	0.002173
TAZ	0.0488566	0.0021732
ECPAS	-0.0704384	0.0021889
FBXL5	-0.0297579	0.0021956
MAP2K4	-0.0022109	0.0021986
YWHAH	-0.0081552	0.0022048
IDH3B	0.0229577	0.0022064
CKS2	-0.0794991	0.0022192
SLC39A10	-0.1013363	0.0022195
ZNF623	-0.0286017	0.0022198
UCHL5	0.0295057	0.0022273
GOLGA8A	-0.0406025	0.0022281
RASAL2	-0.0598535	0.0022331
MAN1A2	-0.0765652	0.0022368
MYL5	0.0570388	0.0022375
CAMK2G	0.0529345	0.0022441
TIMM21	-0.0237807	0.0022464
TMEM256	0.0022544	0.0022483
NIPSNAP3B	-0.0295706	0.0022559
TMEM184B	-0.0067036	0.0022572
CMBL	0.0881542	0.0022596
FOXN3	-0.1163664	0.0022605
CNTLN	0.105965	0.0022626
KBTBD2	-0.0527591	0.0022634
SPIN1	-0.0659095	0.002264
AC079781.5	-0.0284159	0.0022652
SYTL1	0.0125121	0.00227
NECAP1	-0.0257467	0.0022715
ACAT2	-0.0276358	0.0022742
RNF19B	-0.0448472	0.0022759
ABL1	0.0051827	0.0022779
B3GALNT1	0.0011722	0.0022798
UBE2F	-0.1454347	0.0022841
PLAGL2	0.0088168	0.0022878
CARD8-AS1	-0.0125543	0.0022962
AC007566.1	-0.0707319	0.0023058
AC004148.1	-0.0244623	0.0023162
LRMDA	-0.0947401	0.002321
H2AFY2	-0.0023504	0.0023322
SLC25A25-A	-0.0314609	0.0023438

CHERP	0.0331297	0.0023566
SNHG12	-0.1980182	0.002362
PCYT2	-0.0263424	0.0023624
TEX261	-0.0144462	0.0023678
CCNT2	-0.0453896	0.0023814
CAHM	0.0086329	0.002394
ISOC1	-0.0200346	0.0024039
RNF125	0.1146294	0.0024082
BCKDHB	-0.040403	0.0024099
KMT5B	-0.0244846	0.002413
ALKBH4	-0.0182636	0.0024185
TAPBPL	0.0243121	0.0024224
C1orf131	0.0444069	0.0024302
FOXN2	-0.0380834	0.002437
LIPE-AS1	-0.0438274	0.0024534
LY6G5B	-0.0302183	0.0024553
BIK	-0.0630931	0.0024584
NARS2	0.0132785	0.0024598
TSHZ1	-0.0249687	0.0024626
LIN54	-0.0277881	0.0024703
GINS2	0.0899081	0.0024745
HIRIP3	0.0678946	0.0024786
YTHDF3	-0.0576155	0.0024866
MYO1G	-0.1228169	0.002489
GON4L	0.0221461	0.0025083
AL035530.2	-0.022486	0.0025196
EIF4ENIF1	-0.0187818	0.0025214
RHOF	-0.0272008	0.0025252
TARS	-0.0174512	0.0025262
CUX1	-0.1445427	0.0025354
EIF4A3	-0.0268729	0.0025354
SDR39U1	0.0605369	0.0025458
HIF1A-AS3	-0.0407326	0.0025528
NRXN2	0.0629878	0.0025596
KDM5C	-0.046986	0.0025609
RELA	-0.0575061	0.0025626
EXOC3-AS1	-0.0406252	0.0025748
CLN5	-0.0772708	0.0025785
ATP6V0C	-0.1669598	0.0025818
ESRRA	-0.0046811	0.0025831
EXOC5	-0.0359423	0.002616
SECISBP2L	-0.0536276	0.0026203
DCAF11	-0.06367	0.0026211
DTX2	0.0089613	0.0026272
OSTM1	-0.0494027	0.0026367
LPAR2	-0.0293938	0.0026424
SEPHS2	-0.0928742	0.0026479
DAP	-0.0268957	0.0026518
FAM234A	-0.0120566	0.0026539
LIMK1	-0.0560426	0.0026549

RXFP1	0.0986179	0.0026553
ZNF793	-0.0643787	0.0026555
RBM15	0.0152012	0.0026589
DAP3	-0.0066407	0.0026602
MSMO1	-0.1036794	0.0026618
Z83843.1	-0.1289537	0.0026661
ACAA1	0.0490942	0.0026671
OTUD4	-0.0490559	0.0026713
BTAF1	-0.034209	0.0026737
TNFRSF18	0.121665	0.0026837
RABGGTA	-0.0247733	0.0026868
CCDC88C	-0.0565137	0.0026915
PDE1B	-0.0146293	0.0027082
SNX1	-0.0319568	0.0027143
ELP6	0.0404459	0.0027144
CIAO1	0.0437488	0.0027175
MAP2K3	0.0499723	0.0027192
ATG16L2	-0.0547466	0.0027207
MLF2	0.0193809	0.0027267
KDM6A	-0.0439903	0.0027296
RAB33B	-0.002856	0.0027306
STAT2	-0.0891147	0.0027309
CSNK1E	0.0333674	0.0027312
ABCA2	-0.0262589	0.0027501
TP53I3	0.0155795	0.0027867
EVL	-0.0203177	0.0027868
PICK1	0.0186331	0.0027911
TIMM8B	0.0526702	0.0027926
ELP4	-0.0165767	0.0028124
SGMS1-AS1	-0.0300291	0.0028291
TRIOBP	-0.0098741	0.0028291
RP9	0.0344396	0.0028325
EIF2B2	-0.0034737	0.0028373
HDAC2	-0.0895178	0.0028423
STAU1	-0.0205008	0.0028498
NAGLU	-0.0112086	0.0028543
RDH14	0.0170208	0.0028603
APOLD1	-0.1077733	0.0028646
TUFM	0.1355482	0.0028706
SAR1B	-0.0098688	0.0028774
TLE4	-0.0015515	0.0028903
RIOK2	-0.0017085	0.0028939
NEK4	-0.0229834	0.0028977
BRD7	-0.10564	0.0029029
TBC1D10B	-0.0108303	0.0029126
USP20	-0.0560717	0.0029175
SAFB2	-0.0604072	0.0029265
ZNF217	0.0448895	0.002937
TRMT13	0.0002201	0.0029385
NFATC1	-0.0313951	0.0029404

DGCR2	-0.0315476	0.0029421
PARP6	-0.0078991	0.0029525
TMEM179B	0.0421103	0.0029547
DOCK7	0.034847	0.0029815
BAG6	0.0052521	0.0029837
NFE2L1	-0.0907792	0.0029841
RCC1L	0.0169805	0.0029898
RAD23A	-0.0135818	0.0029918
PTBP2	-0.0642191	0.0030108
NME3	0.1156581	0.0030182
TMEM250	-0.0292799	0.0030191
UBE3C	-0.0130148	0.0030289
RAB5C	-0.0352486	0.0030308
RCBTB2	-0.0108816	0.0030427
C2CD3	0.0038392	0.0030466
SRP14-AS1	0.0459112	0.0030483
CNIH4	-0.049571	0.0030536
FMR1-IT1	-0.0426827	0.0030608
USP1	0.0761479	0.0030663
AEBP2	-0.0362492	0.003074
BABAM1	-0.0056637	0.0030979
ABHD12	-0.0142695	0.003106
ZCCHC10	-0.0896057	0.0031142
MDM1	-0.021249	0.0031172
EIF3G	0.1497953	0.0031331
SRM	0.117033	0.0031386
CBX6	0.0863185	0.0031437
C18orf25	-0.026245	0.003162
GDI1	0.0076768	0.0031675
PTPN22	-0.018893	0.0031681
ZRSR2	-0.0611594	0.0031753
DIRAS3	0.066107	0.0031773
MFSD5	-0.025965	0.0031782
PSMB2	0.0652448	0.0031878
MRPS5	-0.0632491	0.0031885
AGGF1	-0.0381303	0.0031903
PML	0.0242184	0.0031939
ZMYM2	-0.1281474	0.0031958
DPF2	-0.0217679	0.0032068
HOXB6	-0.0853519	0.0032101
EIF4G1	0.0030013	0.003211
ENTR1	-0.0049365	0.0032116
AFG3L2	-0.0287667	0.0032144
RRM1	0.0750298	0.0032281
MAGOHB	0.0145369	0.003254
MRS2	-0.0499255	0.0032631
CD151	0.0424863	0.0032677
SYTL4	0.0985707	0.0032722
ZFP82	-0.000187	0.0032891
MPHOSPH1	-0.0266312	0.0033131

PDCD10	-0.0111532	0.0033254
HINT2	0.054437	0.0033278
LINC01150	0.0189562	0.0033282
MRPL20	0.0476806	0.0033332
L3MBTL2	-0.011184	0.0033488
CARMIL2	0.0172281	0.0033492
C16orf58	-0.010339	0.0033506
CPPED1	0.0384913	0.0033939
PCBP1-AS1	-0.0738787	0.0034004
SLC7A1	-0.0136703	0.0034031
PROSER1	-0.0305136	0.0034126
NIPAL2	-0.0300962	0.0034303
PPP3CB-AS	-0.001836	0.0034372
C2orf69	-0.0289722	0.0034415
STK32C	-0.0967571	0.0034477
ACAP3	0.0185614	0.0034506
PCED1A	-0.0325369	0.0034688
PRKDC	0.0876013	0.0034729
IGSF10	-0.0020106	0.0034748
MFSD11	-0.0178732	0.003476
OTUD6B-AS	0.0428714	0.0034835
TCAF1	0.0037379	0.0034888
FER	-0.0564419	0.0034983
BYSL	0.0353338	0.0035161
RNGTT	0.0147744	0.0035165
CORO1C	0.0860954	0.0035248
SDHAF3	-0.0044292	0.0035249
TRAF3IP3	0.0500862	0.0035362
CCDC97	-0.0205766	0.0035411
BLZF1	0.021039	0.003549
EIF1AD	-0.0230597	0.0035547
ZSCAN25	-0.0153525	0.0035556
PRMT3	-0.0350456	0.0035615
PFDN2	0.0911444	0.0035628
WDR60	-0.0120879	0.0035633
PIK3CD	-0.0263299	0.0035638
ZNF91	-0.0824884	0.0035678
DHODH	0.007344	0.0035907
KMT2B	0.0385843	0.0036015
ZNF140	-0.0235935	0.0036075
JRKL	-0.016098	0.0036089
LZIC	0.019761	0.0036096
ZNF737	-0.1128802	0.0036277
CHUK	-0.0206172	0.003634
PHF8	-0.0299511	0.003634
HMGXB4	0.0639294	0.0036347
HAUS1	-0.019449	0.0036422
RNF34	-0.0057928	0.0036455
SPATC1L	0.0400247	0.0036541
SERPINB6	0.1236842	0.0036583

IDH3A	0.0185656	0.0036755
PSTPIP1	0.068796	0.0036766
SCRN2	0.0673604	0.0036907
ZNF408	-0.0042102	0.0036937
ZBTB24	0.0160416	0.0036941
SETDB2	-0.1089228	0.003698
RIT1	-0.0602114	0.0037029
ITFG2-AS1	-0.0411303	0.0037068
DCTN4	-0.0504731	0.0037152
GNB5	-0.0447916	0.0037503
ELOVL1	0.0009783	0.0037506
CORO1A	0.1417466	0.0037542
TMED3	0.0486797	0.0037962
SMG8	-0.0561904	0.0037969
PPP6R3	-0.0521508	0.0037981
EXT2	0.0063405	0.0038042
GIMAP1	0.0091647	0.0038085
DPP9	-0.00108	0.0038091
PPP4R3B	-0.0302269	0.0038329
AC024075.1	-0.0327531	0.0038491
RPL9	-0.106395	0.0038503
BCL2L2	-0.027314	0.0038505
IRF7	-0.0039417	0.0038529
MBTPS1	0.0149981	0.0038571
ATXN7L1	-0.0234306	0.0038656
HMGN4	0.030926	0.0038811
WDR61	-0.0718529	0.0038892
SCO1	-0.0386605	0.0038954
TASOR	-0.022943	0.0039025
AL589863.1	-0.031474	0.0039106
SNX2	0.0623852	0.0039106
LINC00963	-0.0223797	0.0039129
RAB13	0.2074413	0.0039434
CCNB1	0.0279634	0.0039442
ACSL1	0.0168954	0.0039557
CLTC	-0.0407563	0.0039568
HIKESHI	0.0077462	0.0039755
DOK3	0.0179247	0.0039814
TNFSF4	0.1148127	0.0039953
PSMC4	-0.0042549	0.0039965
UBAP1	0.0102697	0.0040186
PPWD1	-0.0786485	0.0040269
CATSPERG	-0.0603312	0.0040298
SELENOF	-0.0847316	0.0040359
NEMP1	-0.0366723	0.0040378
USP34	-0.1037127	0.0040409
ZNF331	0.033514	0.004043
HIST1H1B	0.186698	0.0040489
PANK2	-0.0191439	0.0040514
ANKLE2	-0.0362117	0.0040592

CES2	0.0088581	0.0040624
AL133453.1	-0.0088113	0.0040898
AL138963.4	-0.0420788	0.0040946
AC015802.6	-0.0552768	0.0040988
SERPINF2	-0.0330621	0.0041083
NT5DC1	-0.0505233	0.0041435
CASC3	-0.0191234	0.0041442
TNPO3	-0.0054329	0.0041634
POC1B	0.0532332	0.0041654
FUCA2	0.0457822	0.0041664
NOL9	-0.0229339	0.0041704
TBC1D7	-0.0126482	0.004172
DACH1	-0.0364983	0.0041736
PRDX1	0.236709	0.0041742
ZNF337	0.0021237	0.0041747
SCMH1	-0.0089485	0.0041762
ARSA	0.0044024	0.004177
TNKS	-0.0113538	0.0041783
INTS12	-0.0364181	0.0041786
MYO19	0.0012683	0.0041819
USE1	0.030174	0.0041819
NCBP2	-0.1001079	0.0041864
CSRP1	-0.0718829	0.0041917
TTC14	-0.1376569	0.0042108
LEMD2	-0.0040163	0.0042119
TPGS1	0.1051375	0.0042213
PTPN4	0.0079141	0.0042532
TTLL12	0.002899	0.0042635
MALAT1	-0.1697526	0.0042662
INPP5K	-0.0220052	0.0042728
GPKOW	-0.0153051	0.0043068
UQCRB	-0.1423255	0.0043191
CCDC117	-0.0318373	0.0043276
PIK3C2A	-0.075505	0.0043338
ARPC1A	0.0197732	0.004352
C8orf33	0.0048782	0.0043529
WDR92	0.0057907	0.0043537
CHST13	-0.0186204	0.0043636
UBXN7	-0.0466019	0.0043638
PRPF8	-0.0308978	0.0043692
FLOT2	0.0341623	0.0043965
CYBC1	0.0053572	0.0044065
JADE3	0.0171499	0.0044243
CSGALNACT	-0.0751665	0.0044252
SNRPD1	0.1699756	0.004431
ZNF32	-0.0037014	0.004433
MTERF1	-0.0222588	0.0044514
SH3GL1	-0.055989	0.0044646
ETFRF1	0.0437128	0.0044646
PIP4P1	-0.0519534	0.0045008

VPS36	-0.0573451	0.0045009
FAM210A	0.0177742	0.0045122
NSMCE2	0.0268872	0.0045277
WDR5	0.0091335	0.0045277
EFR3A	-0.0256213	0.0045303
USF2	-0.0971903	0.0045344
UBE2W	-0.0339358	0.0045429
ANKRD50	-0.0514767	0.0045513
ACTB	-0.1481549	0.0045546
CEP19	-0.0569668	0.0045703
ZNF808	-0.0100101	0.0045705
C6orf47	0.0004695	0.0045985
ZNF335	-0.0082428	0.0045985
CWF19L2	-0.0817772	0.0046163
C3orf62	-0.0184561	0.0046252
RABGAP1L	-0.0358331	0.0046385
ARHGAP1	-0.0263882	0.0046474
SELENOO	-0.0228155	0.0046508
FBL	-0.0035516	0.0046608
DEF6	-0.011059	0.004661
PAN3-AS1	-0.0219992	0.0046766
USP47	0.0055791	0.0046841
AC074032.1	-0.0237924	0.004689
LIMK2	-0.0036045	0.0046955
SART1	-0.0287508	0.0046972
PPME1	0.0248339	0.004702
SLC25A20	-0.0334307	0.0047097
ASMTL	0.0150011	0.0047136
BOLA2	-0.0572389	0.0047314
TCTE3	-0.0132903	0.0047439
GPATCH3	-0.0181038	0.00476
SOS1	-0.0898852	0.0047627
FAM104B	0.0121347	0.0047706
RBAK	-0.0302644	0.0047934
AC245060.5	-0.1768928	0.0047984
PDXK	0.0052865	0.0048066
TMEM230	-0.0421552	0.0048175
ZNF587B	-0.0270409	0.0048349
ANKIB1	-0.0457227	0.0048547
HDHD2	-0.0172543	0.0048569
NUP37	0.0367721	0.0048579
TBP	-0.0419933	0.0048621
ARL3	0.0093442	0.0048692
ZNF276	-0.0530039	0.0048732
FAM89A	0.0807691	0.0048753
AL049840.2	0.0091689	0.0048764
KATNA1	0.0260384	0.0048862
BRD4	-0.022946	0.0048976
SFXN4	0.0621763	0.0049015
CCDC125	-0.0507685	0.0049126

ZBTB41	-0.0360283	0.0049235
GPR107	-0.0207519	0.0049331
ELMOD3	-0.0253089	0.0049361
ARRDC5	-0.0326551	0.0049461
PUM2	-0.1157959	0.0049542
AL451085.2	-0.0115888	0.0049582
PHF12	-0.0421119	0.0049673
NDFIP1	0.1164877	0.004971
ZNF70	0.000633	0.0049743
GIPC1	0.0349804	0.0049873
IQCG	-0.0339642	0.0049875
TEX2	-0.035234	0.0049977
KDSR	-0.0168567	0.0050432
MOSPD3	-0.0352866	0.0050465
TSGA10	-0.0638898	0.0050479
POLM	0.0073641	0.0050497
KARS	0.0272531	0.0050852
YRDC	0.0883367	0.0050919
PCBP2-OT1	-0.0291383	0.0050921
MGLL	-0.034137	0.005104
MARC1	-0.0386804	0.0051326
INTS6	0.0910633	0.0051434
SHLD2	-0.0493188	0.0051573
ZNF787	0.0247022	0.0051922
TTC19	0.0695792	0.0051926
MAPK9	-0.0699862	0.005231
ZNHIT3	0.0302955	0.0052499
SDHAF4	-0.0494003	0.00528
HDAC6	-0.0637411	0.0052826
EGLN2	0.0187437	0.0052972
PARP9	0.0573651	0.005299
NUP133	0.0033868	0.0053003
RPAP3	-0.0122506	0.0053018
ABHD15	-0.0347201	0.005314
PSMC2	-0.0602727	0.0053188
TTC21B	-0.0308543	0.0053252
TMC6	-0.0275549	0.0053499
PAFAH1B3	0.0821469	0.0053907
TMEM131L	-0.0012403	0.0054255
BCL2L1	0.030956	0.0054623
PFDN4	0.0246809	0.0054657
RFWD3	-0.0048991	0.0054833
MEPCE	0.0292815	0.0054843
KDM4C	-0.0478068	0.0054893
KHNYN	-0.0172259	0.005511
CKAP2	-0.1342044	0.0055217
ABHD4	0.0144039	0.0055355
ILF3	0.029109	0.0055369
AC002454.1	0.0443458	0.0055628
ABRAXAS2	0.0072467	0.0055671

ZNF436-AS1	-0.0120533	0.0055702
C12orf29	-0.0127487	0.0055772
FAM50A	0.0155378	0.0055798
PPHLN1	-0.0427664	0.0055799
ARRB2	0.138179	0.0056001
TMCC1-AS1	0.0068568	0.0056021
ZNF720	-0.0029293	0.0056054
ABHD17B	0.023747	0.0056075
TRAPPC3	-0.0412708	0.0056096
FIP1L1	0.0148498	0.0056151
AGAP2	0.0214129	0.0056198
METRN	0.0712665	0.0056555
CDC37L1	0.0187637	0.0056866
GCDH	-0.0010502	0.0056923
GPR135	-0.0015682	0.0057014
ZFP62	0.0282476	0.0057086
CLPX	-0.0052482	0.0057141
DDX1	0.0224787	0.0057281
CSF1R	0.0838104	0.0057423
ARMCX6	0.0742472	0.0057428
UXS1	0.0651809	0.0057705
NCOR2	-0.0555507	0.0057781
EWSR1	-0.0794687	0.0057805
GBA	-0.0067113	0.0057854
PLEKHA8	-0.0359987	0.0057984
RPN2	0.1016278	0.0058062
ZNF263	0.0046102	0.0058121
SAMD9	0.031658	0.0058244
TMEM222	-0.0010693	0.0058323
IFI6	0.0272914	0.0058619
SMAGP	-0.023688	0.0058744
PSME2	0.0059083	0.005875
GLMP	-0.0071723	0.0059113
PLBD2	-0.0418626	0.0059234
ANKRD39	0.0116977	0.0059236
PELP1	0.002229	0.0059344
YEATS4	0.0525738	0.0059441
AC022150.4	-0.0187905	0.0059876
POLR2M	-0.0572772	0.0059904
SIN3A	-0.0578663	0.0059971
CTSG	0.1707443	0.0059972
COLGALT1	0.0075691	0.0060007
STARD4	-0.0208218	0.006022
CDK8	-0.0245447	0.0060497
SDAD1	-0.0040109	0.0060571
RAP2B	-0.0491886	0.0060681
DHRS12	0.0480328	0.0060763
MED13	-0.0821755	0.0060929
REPS1	-0.0437267	0.0060933
VAMP4	-0.0204253	0.0060942

PAPOLA	-0.094686	0.0060957
ITM2C	0.1950241	0.0061207
ATG16L1	-0.0117852	0.0061242
KPNB1	0.0960533	0.0061242
ADRB2	-0.1011984	0.0061593
HLX	0.0854964	0.0061908
A2M-AS1	0.0453755	0.0061909
TCHP	0.0412578	0.0062008
UBE2H	-0.0175709	0.0062072
CALU	0.0129522	0.0062075
RRAGA	-0.03736	0.0062663
TFPT	0.0449098	0.0062706
KIAA0930	-0.1088729	0.0062806
EXOC6	0.041726	0.0063147
MRPS24	0.0937749	0.006319
GDI2	-0.1752252	0.0063275
TACC1	0.0480598	0.0063328
FAM210B	-0.0452392	0.0063445
SKP2	-0.006121	0.0063525
SNHG4	-0.0192771	0.0063536
CSTF3	-0.0169082	0.0063562
NASP	0.1211303	0.0063724
GRWD1	0.0277347	0.0063868
PARK7	0.1386776	0.0063878
EXTL2	-0.0258778	0.0063901
AC107375.1	-0.0235337	0.0063933
PIGP	0.0109193	0.006409
CCDC115	-0.0328474	0.0064241
HMBOX1	-0.0647956	0.006429
CRTAP	0.0491122	0.0064441
HMGN1	-0.1456099	0.0064505
RABL6	0.0554154	0.0064576
AC069243.1	-0.0219771	0.0064793
STYX	-0.0579047	0.0064901
LENG9	-0.0461044	0.0065152
NEMF	-0.0004177	0.006527
VPS35L	-0.0061534	0.0065339
BX664615.2	-0.0085223	0.0065358
HEBP1	0.0067446	0.0065997
RTCA	-0.0192085	0.0066052
ANAPC5	-0.1438453	0.0066081
MIRLET7BH	-0.0354091	0.006617
LUC7L2	0.0326023	0.0066175
TRIM23	-0.0149384	0.0066182
UBE2G2	-0.0066382	0.0066238
FCF1	-0.0710965	0.0066309
APOBEC3D	-0.0347561	0.0066362
IARS2	0.0020461	0.0066362
FAM13A	0.0438961	0.0066468
CARD6	0.0059755	0.0066687

TMEM120B	-0.0473992	0.0067032
LIPT1	0.0270757	0.0067066
MCFD2	0.0106152	0.0067125
COPB2	0.0378001	0.0067139
TMEM208	0.0858519	0.0067261
MCM3AP-AS	-0.0020717	0.0067297
LRRC61	-0.0091215	0.0067397
RFC2	0.0083142	0.0067408
SNX4	-0.0682642	0.0067422
DYNLT1	0.065903	0.0067534
C2orf74	-0.0317153	0.0067589
RUNX1	-0.1122775	0.0067598
SCLT1	-0.0212722	0.0067673
IMMP2L	0.0312918	0.0067784
EHBP1L1	-0.0178044	0.0067834
EEF1AKMT2	-0.0485994	0.0068003
AC068888.2	-0.0207604	0.0068011
PPP1R7	0.0298199	0.0068044
EMSY	-0.0437226	0.0068064
MEF2D	0.0267954	0.0068532
C16orf86	0.0283934	0.0068685
ULK1	-0.0308586	0.0068977
DBF4	-0.0340202	0.0069027
UBL7-AS1	0.0241449	0.0069056
DIS3L2	0.0031117	0.0069445
TTC32	0.0126995	0.0069456
SEC24B	-0.0281596	0.0069461
SLC25A13	-0.0361681	0.007021
GOLGA8Q	-0.0192984	0.0070316
MFF	0.0147492	0.0070322
UQCRFS1	0.063536	0.0070342
CDK9	-0.0020137	0.0070367
SUPT3H	0.0131338	0.0070698
CXCL3	0.1767119	0.0070815
MUS81	-0.0402255	0.0070867
RC3H2	-0.0331358	0.0071122
RNF7	0.1353769	0.0071157
GLUD1	-0.0074762	0.0071492
SPPL2B	0.0708097	0.0071516
WDR73	0.0282151	0.0071547
METTL8	0.0003275	0.0071614
RAD17	-0.0428165	0.0071614
USP32	0.0175669	0.0071782
TAB3	-0.0344808	0.0071858
AC036176.1	-0.006297	0.0071881
KDM3A	-0.0439932	0.0071951
SLC37A1	0.0159966	0.0071957
DPY30	0.0577973	0.0072191
LINC02384	0.0201595	0.0072213
EIPR1	0.0096179	0.0072217

PI4KB	0.0091488	0.0072353
TMEM101	-0.0058673	0.0072373
CPSF2	-0.019582	0.0072656
IVNS1ABP	-0.0274671	0.0073538
CMAS	0.0655675	0.0073571
MDN1	-0.0220168	0.0073586
ISYNA1	0.0087753	0.0074055
MT1X	0.0252066	0.0074062
AC027682.4	-0.036152	0.007419
ANP32B	0.1994004	0.0074346
DDRGK1	0.0460217	0.0074769
DNAJC3-DT	-0.0246402	0.0074777
CDK2	0.0164259	0.0075019
CHML	-0.1217647	0.0075055
ACTN4	0.0281489	0.0075154
KLHDC4	0.0189724	0.0075156
AZIN1	-0.0817704	0.0075205
YPEL2	0.0098073	0.0075601
MZF1	0.0202158	0.0075919
SOX18	0.0839225	0.0075934
UBE2R2	0.0104275	0.0075999
TMF1	0.1602076	0.0076208
MAP3K1	-0.0364557	0.0076303
LIG3	-0.0401813	0.0076377
NDUFB3	0.0407286	0.0076424
TANK	-0.0825059	0.0076896
MARCH8	0.0365991	0.0077015
DHX37	-0.0500452	0.0077042
ATF4	0.0764802	0.0077104
TIMM8A	0.0555485	0.0077603
SCARB2	0.0176925	0.0077872
WDR45	0.0079912	0.0077941
GNPNAT1	-0.0009861	0.0077965
ERO1A	-0.0420444	0.0078365
WBP1L	-0.0302921	0.0078409
CCDC71L	0.0093913	0.0078725
AKIRIN2	0.0586698	0.0078828
ZNF100	-0.0334532	0.007884
COPS2	0.0034816	0.0079344
COMTD1	0.058204	0.0079372
MTHFD2	-0.0173888	0.0079534
HARS	-0.0134933	0.0079605
TRAF3IP2-A	-0.0073423	0.0079621
SWSAP1	-0.0056067	0.0079699
CA5B	-0.0345283	0.0079818
CHFR	-0.092761	0.0079952
ANKRD36	-0.0935903	0.0079999
ATN1	0.0026389	0.0080034
TOR1AIP1	-0.1015123	0.0080119
ZNF397	-0.0563172	0.0080122

SETX	0.0487774	0.0080225
CDC5L	0.0645203	0.0080271
LINC00665	0.008355	0.0080298
OSBPL11	-0.0169762	0.0080348
AC097376.3	-0.0095601	0.0080393
KDM3B	-0.0546792	0.008041
TMEM185B	0.0177471	0.0080738
TUSC2	-0.0135945	0.008083
ATL2	-0.0739788	0.0081463
RPS6KB2	0.0227367	0.00816
DDX10	-0.0064377	0.0081741
PLSCR1	-0.0204969	0.0081885
CPNE1	-0.0137894	0.0081897
ZNF638	-0.0614271	0.0081961
PRPF18	-0.0420928	0.0081997
PIDD1	0.0167235	0.0082238
SLC35A4	0.0169792	0.0082238
PER1	0.0459347	0.0082376
ZNF467	0.0817244	0.0082506
CPNE3	0.0894184	0.0082817
BAX	0.0276241	0.0082877
SHC1	0.0144772	0.0083327
FAM222B	0.0221823	0.0083402
ASXL2	0.0001101	0.0083821
TGFBRAP1	0.0242597	0.0084117
NUFIP1	0.0307898	0.0084491
CDK5RAP3	0.0108961	0.0084803
IQCH-AS1	-0.0485664	0.0085005
SLC25A30	-0.0291952	0.0085083
KIF5B	-0.0593338	0.0085156
RIPK1	-0.0134842	0.0085682
MTRF1	-0.0343613	0.0085846
ZNF419	0.0022418	0.0085964
PDE12	0.0283041	0.0085971
POLR2J	0.0114853	0.0086095
TAX1BP1	-0.1955695	0.0086123
ANGEL2	-0.0559351	0.0086184
IFNAR1	-0.0611641	0.0086231
DEFB1	-0.0635314	0.0086291
SNHG21	-0.0247341	0.0086316
ACTR10	-0.0415874	0.008632
AC025171.3	-0.0107003	0.0086358
MDM2	-0.0356043	0.0086683
GRIPAP1	0.0035351	0.0086812
LRRC34	-0.0001135	0.0086821
USP11	-0.0230761	0.0087109
ZNF850	-0.0059444	0.0087477
DECR1	0.0449304	0.008755
SBNO2	-0.029346	0.0087779
S100A8	0.0935626	0.0087782

ARHGAP22	0.1355841	0.0087843
ATP1A1	-0.0425603	0.0088031
NCK2	0.0316782	0.0088075
UBE2K	-0.088457	0.0088169
MEIS1	-0.0845	0.0088253
RBM42	-0.0387011	0.0088316
GLTP	-0.0054867	0.0088443
SERPINB9	0.1047199	0.0088471
TEP1	0.004153	0.0088836
LRSAM1	0.017529	0.0088876
TFRC	-0.0419953	0.008945
AGPS	0.0023818	0.0089458
CHAF1A	0.0657089	0.0089471
APH1A	-0.0204166	0.0089616
AAMDC	0.0259594	0.0089761
NDUFA5	-0.0720385	0.0089803
TERF2	0.0167403	0.0089827
UBA5	0.0155049	0.0089847
TYW3	0.0877791	0.0089885
AP003068.2	-0.0435502	0.0089938
AC138649.1	-0.0990845	0.0089959
MAP4K5	0.0210591	0.0090084
ST3GAL3	0.0146981	0.0090649
USP21	-0.0128456	0.0090689
DENND4B	-0.0395359	0.0090798
IP6K1	0.0415431	0.0091253
ZNF557	-0.004961	0.0091265
TM2D1	-0.0946078	0.0091277
ARHGEF19	-0.0293428	0.0091356
GAS6-AS1	-0.0337218	0.0091653
SEPTIN2	-0.1127277	0.0092013
CDC40	0.0033614	0.0092286
DDHD1	-0.0448417	0.0092369
CFAP44	-0.0170762	0.009247
DCP1B	0.0041904	0.0092509
MRPS15	0.0444346	0.0092569
AC074117.1	-0.0299649	0.0092632
NR1H2	0.0231947	0.0092794
GSTCD	-0.021683	0.0093055
PTGDS	0.4304982	0.0093114
ZNF324B	0.0040178	0.0093196
TELO2	-0.0004942	0.0093235
AC005332.3	-0.0248866	0.0093379
OSER1-DT	0.0139906	0.0093695
MIIP	-0.0098451	0.0093762
HSD17B12	-0.0925272	0.0094046
SF3B2	0.0345258	0.0094216
CYCS	0.0494453	0.0094278
ZNF804A	-0.0492301	0.009433
P2RX1	-0.0405025	0.0094519

COIL	0.0380133	0.0094538
MKS1	0.0067049	0.0094795
SOCS5	-0.0191296	0.0094987
SBNO1-AS1	0.1039365	0.0095004
INTS13	0.0813763	0.0095037
SDE2	0.0710902	0.0095295
FBXL18	-0.0424035	0.0095416
MAD1L1	0.0178305	0.0095542
AC010615.2	-0.0332332	0.0095961
C18orf32	-0.1144877	0.0096024
TMX2	0.0199598	0.0096044
HEXB	0.0206473	0.0096069
PDIA3	-0.0752115	0.0096384
TXNL4A	0.0592177	0.0096508
VMA21	-0.0021077	0.009653
IL10RB	-0.0298985	0.0096599
HPF1	0.0737675	0.0096724
MARCH2	-0.0388039	0.0097114
PIGL	-0.0272766	0.0097233
THAP2	-0.0051903	0.0097559
VPS35	0.0033684	0.0097762
GYG1	0.0210422	0.0097763
PSMB7	0.0916695	0.0097894
SH3PXD2A	-0.0389962	0.0097942
CEP192	-0.0373897	0.0098038
AGXT	0.1644024	0.009816
LEPROT	0.0085371	0.009824
CIAO2A	-0.0158668	0.0098501
ABCE1	0.1278321	0.0098556
ZNF544	-0.0035048	0.0098782
UBE2A	-1.619E-05	0.0098984
BECN1	0.0098387	0.0099107
RFC3	0.0314532	0.0099463
BTBD1	-0.0285667	0.0099476
DCTN6	0.0165791	0.0099635
NANOS3	-0.0301725	0.0100275
KIAA1958	-0.0804564	0.0100288
AC018638.7	-0.0177366	0.010042
SAMSN1	-0.1164468	0.0100752
SNRNP48	-0.0521751	0.0100819
NBEAL1	-0.0545083	0.0100911
AP1G2	-0.0184855	0.0101245
KLHL6	-0.0121055	0.0101357
ZBED1	-0.0538057	0.0101661
RBMXL1	-0.0196044	0.0101801
RTF2	0.045123	0.0102096
PHRF1	-0.0157946	0.0102096
DYNC1LI2	-0.0296599	0.0102258
FUZ	0.0690933	0.0102274
ZNF468	-0.0181972	0.0102379

URGCP	0.0037988	0.0102391
MFSD12	-0.0091219	0.01025
ANKS1A	-0.0024775	0.0102554
ZC3H4	-0.0483914	0.0102605
TMEM19	-0.0342171	0.0102767
RFK	0.0324963	0.0102961
UIMC1	-0.0430316	0.0103206
IFI44	0.0817168	0.0103251
ZNHIT6	-0.0218555	0.0103313
PRR5	0.0643337	0.0103343
UBE2T	0.0267978	0.0103618
AC006059.1	-0.0415313	0.0103647
EPN1	0.0392015	0.0103767
FTSJ3	0.0222009	0.0103896
GLRX	0.1229757	0.0104419
AC073896.2	-0.0222412	0.0104439
DCTN2	0.0434936	0.0104654
XPO7	-0.0642403	0.0104896
METTL23	-0.0106598	0.0104972
RNF115	-0.0263214	0.0105126
ATIC	-0.0162074	0.0105366
SLIRP	-0.02898	0.0105927
GOSR2	-0.0022672	0.010598
B9D2	0.0285626	0.0106136
HDAC8	-0.0689934	0.0106545
MARCH6	-0.0584584	0.0106712
ZNF92	-0.0150569	0.0106721
KPNA6	-0.0229158	0.010677
NDUFS6	0.115716	0.0107413
SIGMAR1	0.0045279	0.0107671
KLHL2	0.0177774	0.0107733
TUG1	0.0007025	0.0107919
BTN3A1	0.0047663	0.0108418
STAG2	0.0167601	0.0108557
CWC27	0.070174	0.010901
ZNF16	-0.016291	0.0109114
PARP8	0.0013674	0.0109344
ZNF805	0.004289	0.0109423
PRKAG2	0.0138021	0.0109533
AC144521.1	-0.0322141	0.010959
GABARAPL2	-0.07787	0.0109922
CLIC2	0.0404574	0.0110047
ALKBH8	0.029431	0.0110138
KLHL18	0.0715088	0.0110667
MICAL3	-0.0021597	0.0110825
CHD8	-0.0457636	0.0110865
NAMPT	0.0408486	0.0111023
HERC4	-0.0899352	0.0111042
SCARF1	0.0453125	0.0111346
LCOR	-0.0214139	0.0112363

KANK2	0.0552974	0.0112702
HACD4	0.1463926	0.0112758
LTBP4	-0.0118839	0.011281
AKAP8L	-0.0291954	0.0112856
ADNP	-0.0127559	0.0112884
RFXAP	0.0405148	0.0113402
LHPP	-0.0043615	0.0113437
JPT1	0.0243933	0.011344
ZNF800	0.0368292	0.0113877
WAC	-0.0343584	0.0113904
ECSIT	0.023206	0.0114659
LINC00539	-0.0137166	0.0114913
TRIM5	-0.0239832	0.0115174
ACO1	-0.0312023	0.0115536
FKBP4	0.1034684	0.0115861
GSKIP	-0.0133567	0.0115907
ETF1	0.0419926	0.0116269
AC005072.1	-0.0291265	0.0116324
FAHD1	0.0173859	0.0116454
ZNF664	-0.0015338	0.0116703
LINC00641	-0.038211	0.0116882
ATG2A	-0.0054421	0.0117087
SLC25A28	0.0651475	0.0117138
CDC42EP3	-0.0373032	0.011717
GNAQ	-0.068545	0.0117448
RHBDF1	0.0536494	0.0117636
SIAH2	0.0687269	0.0117948
PER2	0.0039503	0.0118052
STRADB	0.0153194	0.0118061
SF3B5	0.0412726	0.011822
NDUFAF1	0.0155746	0.0118375
BLMH	-0.0067198	0.0118501
ARL1	0.003943	0.0118674
PPP2R5A	0.0512409	0.0118968
AL450384.2	-0.0161272	0.0119254
ASB16-AS1	-0.0293269	0.0119605
SLC10A3	-0.0287378	0.0119998
MIR181A1H(	-0.0221536	0.0120066
TMED7	-0.0216629	0.0120598
CCNB1IP1	-0.0654011	0.0120776
CARS	-0.0277885	0.0120907
GPR174	0.1552836	0.0120919
VEZT	0.0134616	0.0121006
BCL2L12	0.0226882	0.012145
RAB5A	-0.0041073	0.012148
MED16	0.0215108	0.0122374
BOLA1	0.0411445	0.0122522
AC105285.1	0.0131146	0.0123085
HMGCR	-0.0748134	0.0123118
ZFAND3	-0.0919299	0.0123175

BTN3A2	0.040901	0.0123311
NIPSNAP2	-0.0733964	0.0123493
CGGBP1	-0.1049861	0.0123862
BAZ1B	-0.0085925	0.0124003
PHF14	-0.0775774	0.0124208
C16orf91	0.0190306	0.0125319
NEIL2	0.022971	0.0125353
NUDCD3	-0.0304368	0.0125445
RAB7B	-0.0563188	0.0125813
ZNHIT1	-0.0293256	0.0125933
ATRN	-0.040257	0.0126722
S100PBP	-0.065266	0.0127054
PLPP5	0.034564	0.012709
TNPO1	-0.0888277	0.012715
RETREG3	-0.0285415	0.0127787
ZNF35	-0.0007744	0.0127882
ARAF	-0.0261888	0.0128546
NUP188	-0.0184975	0.0128646
WASHC2A	-0.1202095	0.0128702
ANKRD16	0.0411512	0.0129107
AC084198.2	-0.0039261	0.0129552
STX12	0.009438	0.0129612
ITGAV	-0.0390861	0.0129613
DGKZ	-0.0625043	0.0129986
LYSMD3	-0.0605999	0.0130345
DNASE1	-0.0534853	0.0130347
SLC43A3	0.0514373	0.0130488
METTL2B	-0.0088334	0.0130639
ZNF395	-0.0149461	0.0130786
TRIM21	-0.0153619	0.01309
MAVS	0.0058555	0.0131121
CCNL2	-0.0400646	0.0131409
TRAF6	0.0005854	0.0131619
MROH1	-0.0308894	0.0131721
RGS14	0.0264519	0.0131737
RNF114	-0.0744083	0.0131774
LRRC8C-DT	0.0259862	0.0131918
TMEM170B	-0.0656851	0.0132005
RCOR3	-0.098383	0.0132057
RALY	0.0488704	0.0132114
ARHGEF3	-0.026297	0.0132531
SMG6	-0.0190138	0.0132681
PAXX	0.0247085	0.0132705
WWC3	-0.0542602	0.0132756
HDGF	-0.1104308	0.0132804
PAK1	0.1230328	0.0133091
NTMT1	0.0005978	0.0133492
ASH2L	-0.0118539	0.0133512
SNAP47	0.0529158	0.0133518
BID	-0.1040088	0.0133772

ZBTB21	0.0058069	0.0133897
THUMPD2	-0.0509254	0.0134084
ANKRA2	-0.066641	0.0134122
CTCF	0.0289493	0.0134162
COX20	-0.0609629	0.0134238
XRN1	-0.0829954	0.0134742
ZNF669	-0.0219833	0.0135098
IFIH1	-0.0353456	0.0135751
SGSM3	0.0136585	0.0135843
AP3D1	-0.0268478	0.0135888
PKM	0.0681563	0.0135991
GPRC5D-AS	0.0049811	0.0137438
GTDC1	-0.0255887	0.0137554
KIN	0.0454881	0.0137863
RSAD1	-0.0160805	0.0138052
CAPN10	-0.0126153	0.0138269
MTM1	-0.0401	0.0138277
ARFGEF2	-0.0635593	0.0138328
DYNLRB1	-0.0788952	0.0138611
RUNX2	0.0078254	0.0139083
ENOPH1	-0.0131825	0.013915
DDX47	-0.0176352	0.0139255
SLC9A8	-0.0249567	0.013967
SMCHD1	-0.1181188	0.013985
RAB11FIP1	0.1621497	0.0139963
SLC12A6	-0.1148799	0.0140558
MCRS1	-0.0265968	0.0140651
SUPT16H	0.0668886	0.0140871
AC015871.4	-0.0404734	0.0140875
BUD13	-0.0141087	0.0140975
FAM120B	-0.0138109	0.0141212
EXOSC7	0.0287286	0.0141488
AP000873.2	0.0230566	0.0141917
AC243960.1	0.0941316	0.0141932
AC006213.2	-0.0394294	0.0142238
ACTA2	0.0942569	0.0142297
GPR89B	-0.0425027	0.0142955
NMI	-0.0441753	0.0143269
ZNF268	-0.0353403	0.0143518
NABP2	0.0184247	0.014379
MACROD1	0.0326547	0.0143822
EIF2B4	-0.0385587	0.0144306
C20orf194	-0.0122451	0.0144474
C10orf95	-0.0528597	0.0144627
PHAX	0.0170386	0.0145554
GANC	-0.019135	0.0145558
TTC9C	-0.0345252	0.0145778
SMIM12	0.0007966	0.0145881
NOL10	0.0088776	0.0145909
ZNF699	0.0041798	0.0146283

FAM91A1	-0.0284291	0.0146876
KIAA1191	-0.0366233	0.014731
POLR2K	-0.0629166	0.0147978
XIAP	-0.0636241	0.0148143
MRPS36	-0.0346004	0.0148358
MLYCD	0.0020139	0.0148381
TPST2	0.0630102	0.0148453
OSBPL1A	0.0404169	0.0148802
INPP5D	-0.0780375	0.0148887
PSMB8-AS1	0.0923506	0.0149469
FBXO34	-0.0058274	0.0149682
SZRD1	0.002638	0.0150042
CRK	-0.0401724	0.0150158
ATPAF2	-0.0147302	0.0150401
ZNF562	-0.0483892	0.0150405
COX11	0.0047469	0.0151246
INPPL1	-0.0374219	0.0151369
NEU1	0.0501272	0.0151502
LGALS3BP	0.1271434	0.0151584
GTF3C5	0.0153775	0.0151818
STK11IP	0.0274342	0.0152088
SQLE	-0.039004	0.0152441
ERO1B	-0.0606174	0.0152444
VCL	0.1321305	0.0152586
SDCBP	-0.0573946	0.0152835
LINC00528	-0.018544	0.0153018
RTN2	0.0537479	0.0153629
DCUN1D5	0.0654268	0.0153776
MIS12	-0.0135092	0.0154306
CBR4	-0.0500127	0.0154377
NIP7	0.0589339	0.0154384
TOGARAM1	0.0114368	0.0154616
TATDN2	-0.020439	0.0154653
WSB2	-0.017835	0.0154763
CHORDC1	0.014901	0.0155177
APOL6	0.0267201	0.0155293
HNRNPF	0.0457526	0.0155311
NMB	0.0631316	0.0155764
HACE1	-0.0217829	0.0155923
AC108519.1	-0.0151604	0.0156129
SMCR5	-0.0296989	0.015689
INVS	-0.0133548	0.015712
AC106707.1	-0.0045793	0.0157199
MTO1	-0.0427548	0.0157406
SNHG10	0.0264563	0.015938
CFP	0.0724491	0.0160449
PKN1	-0.0177471	0.0161407
TRMU	-0.0012979	0.0161963
TMC8	-0.0253955	0.0162991
RSPRY1	0.0109501	0.0164106

RBM15B	-0.0174614	0.0164415
LRRC40	-0.035247	0.0164858
CDK16	-0.0148713	0.0166179
ERMP1	0.0061061	0.0166413
TAOK3	-0.1205775	0.0167346
RUVBL1	0.0918015	0.0168188
NFKBID	0.0640624	0.0168511
MPHOSPH6	-0.0440124	0.0168581
ACYP1	0.0289943	0.0168683
ZNF708	-0.0561908	0.0169162
FXR2	-0.0155859	0.0169388
GTF2IRD1	-0.0198262	0.0169444
DNM2	-0.0456325	0.0170078
ABCB10	-0.0205032	0.0170084
LINC02256	-0.065991	0.0170264
EZH1	0.0151379	0.0170278
AL353622.1	-0.0403449	0.0170436
C5orf24	-0.0463987	0.017046
LENG8	-0.058204	0.0170534
NDUFAF6	-0.0148994	0.017097
DNAJC7	0.0745124	0.0171122
NAP1L4	-0.023985	0.0171288
CANX	-0.0999028	0.017153
POT1	-0.0365388	0.0171571
ARL11	-0.0212611	0.0171713
PABPC4	-0.0578745	0.0171756
NET1	-0.0073583	0.017185
GGA1	0.0009844	0.0172299
ALKBH2	0.018407	0.0172947
GCAT	0.0422051	0.0173119
SUOX	-0.0155478	0.0173674
SHMT2	0.0306494	0.0173924
TMEM39A	0.0008317	0.0174345
PTGS1	-0.0579703	0.0174441
ATP5MG	-0.1386326	0.0174463
CCNYL1	-0.044974	0.0174662
CCDC138	0.0078604	0.0174674
ZNF791	-0.0452099	0.0175084
MOB4	-0.030013	0.0175607
EEF1E1	0.0081216	0.0176068
KAT6A	-0.0692203	0.0176862
PPP1R11	-0.040203	0.0176902
UBTF	-0.0895185	0.017694
FAM136A	0.0485027	0.017715
NAA38	0.0650387	0.017792
PA2G4	0.1803334	0.0177975
BCAP31	0.110998	0.0178441
CWF19L1	-0.0430515	0.0179282
LINC00205	-0.0160993	0.0179339
SLC25A12	0.0112962	0.017977

TNFAIP1	-0.014691	0.0179875
KAT2A	0.0003838	0.0179896
SNX11	-0.0286356	0.018001
ZBTB6	0.0192541	0.0180018
ZKSCAN3	0.0058078	0.0180127
VPS37B	0.0514601	0.0180151
AURKAIP1	0.1035871	0.0180603
C12orf73	0.0661739	0.0180615
CSK	0.0165805	0.0180975
PTMA	-0.1059171	0.0182126
PRPF39	-0.0076349	0.0182149
LUC7L	-0.0480731	0.0182404
RAB14	-0.0312208	0.0182567
GARS	0.0448978	0.0182654
CCDC57	0.0703051	0.0184098
DUT	0.2219765	0.0184357
F11R	-0.0502068	0.0184448
CEP152	-0.0356572	0.0185432
IL1RAP	0.0942662	0.0186654
ZNF257	-0.0045737	0.0186838
ORMDL2	0.0559374	0.0187141
RNF135	0.0008525	0.018835
IK	0.0072523	0.0188826
SHLD1	0.0039744	0.0189199
ITPR1	-0.0668363	0.0189269
MCCC2	-0.0001341	0.0189316
TNPO2	-0.0245001	0.0189347
RPS6KA5	-0.0548431	0.019015
IRF8	-0.1238935	0.0190192
SUMF2	-0.0935097	0.0190687
TSC22D3	0.1531634	0.0191085
LACTB	-0.0897914	0.0192181
ELOC	-0.0446185	0.0192271
TOR1B	-0.0127658	0.0192521
PSMD13	0.09067	0.0192551
ZNF212	-0.0092557	0.0192553
PLAU	-0.0086123	0.0192941
CPOX	-0.0023656	0.019297
GPANK1	0.0119733	0.0193688
RNF187	0.0196805	0.0193887
NUBP2	-0.0040181	0.0193895
RABEP2	-0.0080925	0.0194655
ACAD10	-0.0138265	0.0194754
ZNF777	0.005622	0.0194884
SRF	-0.021742	0.019618
DNAJB1	-0.0395771	0.0196784
DENND4C	-0.0186881	0.0196935
ACIN1	0.0064201	0.0197344
KCTD2	-0.0241148	0.0197526
SLC35C2	-0.0269493	0.0197653

SNF8	0.0480479	0.0197719
WDR37	-0.0292359	0.0198884
TFCP2	-0.0627828	0.0198891
TOR2A	-0.0041163	0.019968
ZDHHC3	-0.0060193	0.0199804
PPIL2	-0.0433142	0.0200499
PEX10	0.0196824	0.0201196
ASCC3	0.069971	0.0201811
RUSC2	0.0125768	0.0202725
ABCG1	0.0812498	0.0202932
ALMS1	0.0065765	0.0203792
MFN2	-0.0188477	0.0205007
HAUS4	0.0472479	0.0205507
SCP2	-0.0972694	0.0205854
AC007262.2	-0.0555834	0.0206709
JOSD2	0.0667521	0.0207049
COP1	-0.005486	0.0207658
ZDHHC23	0.0073959	0.0207953
ZNF226	-0.03051	0.0208062
PLEKHM3	-0.0596549	0.0208289
AC009404.1	-0.0310992	0.020836
UBE2V2	-0.0181241	0.0208368
YIPF1	-0.0198547	0.020846
GAPT	-0.1421217	0.0208463
RASA3	-0.0816911	0.0209605
ILF2	0.0088144	0.02109
DTNBP1	0.0369412	0.021179
SH3BP2	0.0522012	0.0212057
TSC22D1	0.1313307	0.0212212
DRAM1	0.0188206	0.0212347
FAM120C	0.0152608	0.021249
RPP40	0.0139423	0.0212728
CPD	-0.0516892	0.0213658
FBXL3	-0.0096193	0.0213844
USF1	-0.0180563	0.0214112
ZNF655	-0.0239238	0.0214283
SYS1	0.0066594	0.0215085
ZNF316	0.0060981	0.0215754
ZNF195	-0.0846093	0.0215886
CMTR2	-0.0309506	0.0216092
ZNF836	-0.0163768	0.0217178
MMP28	0.0400211	0.0217337
QDPR	0.0569291	0.021766
COA6-AS1	-0.0078622	0.0217906
DCTN1	-0.0234456	0.0218297
ZNF169	-0.0090607	0.0218666
RNF13	0.0271213	0.0219109
KANSL2	-0.0039732	0.0219569
RBM34	-0.0606733	0.0219765
SNRNP200	-0.0379856	0.0220161

RNF213	0.0460865	0.0220247
UBE2D4	0.0191884	0.0220413
MYC	0.3020218	0.0220492
MBNL3	-0.0874147	0.0221259
SLC2A13	-0.0278717	0.022141
ANKMY2	0.0049473	0.0222203
LY75	-0.0947605	0.0223186
SLC52A2	0.0314949	0.0223191
SCCPDH	-0.0014117	0.0223354
MOSMO	0.0113393	0.0223861
PLPBP	0.0088127	0.0223874
MRPS33	0.0592918	0.0224236
PRKRIP1	0.0106105	0.0224507
CREM	-0.041671	0.0224601
TAP2	-0.034578	0.022531
AC060780.1	-0.0372234	0.0226038
POLR1C	0.0013458	0.022613
PBRM1	-0.1384704	0.0226189
FBXO25	-0.0635017	0.0226351
WDR76	0.0570052	0.0226601
GNAI2	0.149108	0.0226877
AC010618.3	-0.0206686	0.0226926
CYTH4	0.0872881	0.0226961
TMEM106C	0.0703032	0.0227449
NBPF9	-0.0332676	0.0227826
TMEM129	0.0142952	0.0228004
VHL	-0.0223595	0.022836
CBR1	0.0809256	0.0228461
FAM98C	0.0004153	0.0228773
ETAA1	0.0041273	0.0228906
SMAD4	-0.0303434	0.0229396
PIP4K2B	-0.0091724	0.0229543
SELL	0.1064402	0.0229838
POLR2D	-0.0694614	0.023011
GSTM2	0.0251745	0.023013
PAAF1	-0.0315234	0.0230534
BACE1	0.071602	0.0230715
NAT9	0.0077518	0.0230907
TMEM141	0.0897783	0.0231603
LNPK	-0.0244534	0.023183
OAZ2	0.0253707	0.0231878
VTA1	-0.0342074	0.0232334
SCARB1	-0.0208548	0.0232422
PET117	-0.0480248	0.0232947
MPHOSPH9	-0.0122679	0.0233247
TM9SF2	-0.0382093	0.0234316
SERPINF1	-0.0644773	0.0234716
UBTD2	0.0161363	0.0235173
COG4	-0.0314693	0.0235184
ZNF738	-0.055316	0.023621

NOC2L	0.0218097	0.023628
DPPA4	-0.1455586	0.0236618
TMUB1	0.0607158	0.0236876
PCNX4	-0.1507228	0.0237631
CEP164	0.0311828	0.0238042
ACTR6	0.017803	0.0239304
STXBP3	-0.1192049	0.0239802
CMC1	0.0433978	0.0239992
TOMM22	0.0004745	0.024088
SEC16A	-0.0282856	0.0240942
FAM184B	-0.0257714	0.0241272
GLE1	-0.0065114	0.0241455
SMDT1	0.1055526	0.024176
CUL9	-0.0256382	0.0242213
CAPN15	0.0139139	0.0243304
PURB	-0.0524912	0.024378
USP3	0.0078147	0.0243899
ZNF211	0.0079162	0.0244087
TAF7	-0.1220923	0.0244318
ATP2B1	0.1303782	0.0244766
AIMP2	0.0251956	0.0244837
MROH6	-0.0313909	0.0245296
GTF2H2	-0.0692045	0.0245983
RGPD5	-0.0213344	0.0246305
PPAT	0.0485431	0.0246317
AKAP13	0.0460144	0.0246772
PPP2R5D	-0.0223275	0.0246896
ABHD5	-0.056083	0.0247206
DPCD	0.0289966	0.0247211
CASD1	-0.018295	0.0247236
SLC25A19	0.0130326	0.0248063
PDCD11	0.0053812	0.0248239
SMCO4	0.0249544	0.0249524
ABCB7	-0.0342721	0.0249918
NEDD4	0.0619803	0.0249922
AC244090.1	-0.0069513	0.0251076
CARD16	0.0185327	0.0252969
SAP130	-0.0227674	0.0253032
ATXN1L	-0.0107531	0.0256204
ATP5PB	0.0633554	0.0256511
MRPS6	0.0540674	0.025691
PLAC8	0.1908455	0.025717
NPHP3	-0.0479278	0.0257635
FAM229B	0.0562593	0.0257918
TOM1L1	0.0277752	0.0258357
CTDNEP1	0.0056153	0.0259453
CD9	-0.5442613	0.0260597
IFFO1	-0.0197587	0.0261266
CLTB	0.0311695	0.0261366
ZFYVE21	-9.235E-05	0.0261532

PTCD2	0.0110636	0.026188
E2F5	0.044448	0.0262686
QRSL1	-0.0377312	0.0262852
SPATA7	0.0018638	0.0264043
SBDS	0.0541161	0.0265137
CATSPER2	-0.0489758	0.0265689
HPS1	-0.070887	0.02661
HSPA8	0.2164709	0.0266267
NCBP1	-0.0307438	0.026636
MORN2	0.0084337	0.0266785
AC005562.1	-0.0275119	0.0267903
G6PC3	0.0542114	0.0268095
PRUNE1	-0.0034722	0.0268154
SGTA	-0.0173516	0.0268917
EHMT1	-0.0098777	0.0269489
UBE2O	-0.0017517	0.0269991
SEC24C	-0.0269706	0.0270204
BAP1	-0.0236413	0.0270779
Z93930.2	0.0248335	0.0270831
U91328.1	-0.0259804	0.0273347
FGD4	0.0764461	0.027364
AC009403.1	0.0059451	0.0274454
ACTR3B	-0.0104388	0.0274636
EMC4	-0.0624726	0.027471
C18orf21	-0.0211227	0.0275296
NDUFS8	0.0456985	0.027541
MBLAC2	0.0257311	0.0275497
SPAST	-0.0654532	0.0275889
FHIT	-0.0038561	0.0276671
PABPN1	-0.0350968	0.0276717
AC015712.2	-0.0017322	0.0277181
ERMARD	-0.0312169	0.0277242
TAB1	-0.0061704	0.0277298
PRDM11	-0.003982	0.0278152
CRNKL1	0.002844	0.0278353
CWC25	0.068214	0.0278535
PIK3R5	-0.0462139	0.0280463
RNF139-AS1	-0.0129494	0.0280767
CWC22	0.0372433	0.0280858
TMED4	0.0815589	0.028309
ZNRD2	0.0365285	0.0283253
DEPDC1B	0.0543734	0.0283794
ZCRB1	0.1016908	0.0284406
TSPAN33	0.1019662	0.0284751
LMAN1	0.1325789	0.0284758
C2orf49	-0.0469164	0.0284942
AGBL3	-0.0223059	0.0285549
PRDM16-DT	-0.0758553	0.0286396
RAB24	-0.0217553	0.0286722
NUDT22	0.0244013	0.028681

DDX39B	0.0867818	0.0286982
TBCC	0.0374411	0.0288319
ZBTB14	-0.0186868	0.0289734
OCLN	-0.0837926	0.028974
KIF13A	0.0484772	0.0290133
CEBPZ	0.0380598	0.0291526
NUDT19	0.0055504	0.0291878
LINC00672	-0.0040896	0.0292151
TEX30	0.0492004	0.0292822
EIF6	0.0614748	0.0293196
MIEN1	-0.0004255	0.0293215
FAM8A1	0.0092084	0.029337
ETHE1	0.0553469	0.029374
C5orf22	-0.0129212	0.0293852
ZBTB46	0.0098411	0.0294683
AC024060.2	-0.0410167	0.0294913
DDX42	0.0106115	0.0295218
SLC50A1	0.0815557	0.0296309
TMEM187	-0.0081921	0.0297012
TRRAP	-0.0415996	0.0297027
ZBTB20	-0.2618545	0.0297085
PSMD5	-0.0218037	0.0297804
NSA2	-0.1952888	0.029868
SETD4	-0.0146959	0.0298732
EHMT2	-0.0181392	0.0299502
RPS6KC1	-0.0189182	0.029954
ERG28	-0.0189807	0.0300699
APBB1IP	0.0903063	0.0303446
PRKCQ-AS1	0.0332667	0.030375
MUTYH	0.0206743	0.0305355
ZNF444	0.0436916	0.0305459
ATP5F1D	0.1669613	0.0306893
C4orf46	-0.0494383	0.0306917
ADIPOR2	-0.062334	0.0307572
ARID1B	-0.1081836	0.0307766
ATG7	-0.051039	0.0309182
MAFIP	-0.0303466	0.0309278
CLUH	0.0088523	0.0309985
C1orf174	-0.0709267	0.0310106
EEF2K	0.0154385	0.0311218
WAPL	-0.0421269	0.0311552
HSPB1	0.1985642	0.0312637
HINT3	-0.0626929	0.0312954
SERINC3	-0.0684414	0.0313787
PIK3R2	-0.0160845	0.0314314
SAAL1	-0.0122876	0.0317754
CYP51A1	-0.0597357	0.0318035
TARS2	-0.0030114	0.031805
SETD1A	-0.016006	0.0318317
BTBD3	-0.0087656	0.0318356

SOS2	-0.159036	0.0321393
FEZ2	0.0050731	0.0321731
ITGB1	-0.0669341	0.0323002
KRAS	0.0186199	0.032382
ZYG11B	-0.0248715	0.0323947
TRPC4AP	0.0037145	0.0325109
ARV1	-0.0013891	0.0326117
DNMT3B	0.0344045	0.0327165
TRIM73	-0.0787615	0.0327205
PYURF	0.0443985	0.0327252
GMDS	0.0115358	0.0327466
ST8SIA4	-0.0529048	0.0328385
MLX	0.0074217	0.0329132
GDE1	-0.0002662	0.032935
RALBP1	0.1159165	0.0329636
SEC61A2	0.0020511	0.0330507
BTBD6	-0.0135935	0.0331257
PREX1	0.0018821	0.033178
CUL4B	0.00023	0.0331998
AMFR	-0.0507435	0.0332547
LINC01341	0.0148493	0.033291
TRIM22	0.0651635	0.0333455
MED12L	-0.0567146	0.0333561
AC006504.5	-0.0897706	0.0333661
ABI2	0.116524	0.0334681
ENTPD4	-0.0484072	0.0335398
AC104162.2	-0.0279784	0.0336059
SERINC1	-0.0247664	0.0337206
ADPGK	-0.0607041	0.033769
WASHC5	-0.0319894	0.0338046
UCK2	0.0310913	0.0338572
SNAPIN	0.0034013	0.0338791
ADGRG5	0.0168428	0.033905
RNF6	-0.0161111	0.0339894
MORC2	-0.027284	0.0339895
LUZP1	0.0832629	0.0340493
AC017083.1	0.0137431	0.0340837
CDK14	-0.0529057	0.0341872
PAXBP1	-0.0582671	0.0341964
AC124283.1	-5.448E-05	0.034209
SLA2	0.0137974	0.0342603
UBE2E2	0.068454	0.0344634
MAPK1	-0.0724787	0.0345233
ROGDI	0.0703949	0.0345745
AIG1	0.0365201	0.0346189
MACO1	-0.0186836	0.034899
BSDC1	-0.0065786	0.0349211
BMT2	-0.0430615	0.0349951
RHNO1	0.0048694	0.0350737
CCT7	0.0708544	0.0352048

AP3S1	0.0299463	0.0352383
VWA5A	-0.0958117	0.0352757
ASB6	0.0035828	0.035376
MIATNB	-0.0295167	0.0354085
HEXIM1	-0.0327188	0.0354842
SMIM10L1	-0.044845	0.0355104
GOLGA3	-0.0488749	0.0355756
PRMT1	-0.0330222	0.0356254
BUB3	-0.0089802	0.0356527
LINC01138	-0.0485342	0.0356634
CHD1L	-0.0534432	0.0357104
GSS	0.0075346	0.0357608
BLOC1S5	-0.0504094	0.0357621
ATP5F1E	-0.1267786	0.0357786
SMYD3	-0.0016316	0.0358438
MICU2	-0.0573746	0.0358948
NIPA2	-0.0132355	0.0359447
ZKSCAN5	-0.019782	0.0359607
CIRBP	0.1889585	0.0360211
DOCK8	-0.0452119	0.0360782
ZNF688	0.0163298	0.0361098
C3orf80	-0.0740974	0.0361876
RCSD1	0.0052508	0.0362873
HELLS	0.1470921	0.0364175
SETD9	-0.0099156	0.0364795
LRRC27	-0.0191274	0.0365347
NKAPD1	-0.0460347	0.0365394
USP8	-0.054262	0.0366045
YTHDF2	-0.0039173	0.0366615
MIS18A	0.0275672	0.036692
ASCC2	-0.0738673	0.0367495
NIT1	-0.0218587	0.0368291
SEC31A	-0.0464428	0.0369133
MEF2C-AS1	0.0442784	0.0369646
PPP6R2	-0.0016596	0.0369765
APOBEC3F	0.0030556	0.0369835
CENPW	0.0396283	0.0370616
ZNF592	-0.0458279	0.0370847
AC007325.4	-0.0169375	0.0371911
ORC5	-0.0146396	0.0372122
PIAS2	-0.1207573	0.0373388
GHITM	-0.1019308	0.0376464
FBXW7	0.0232053	0.0377708
CEP76	-0.0556204	0.0377738
FRAT2	0.02873	0.0379285
DOP1A	-0.0418982	0.0379511
TFPI	0.0430156	0.0379601
CNBP	0.108057	0.0379831
KIF20B	0.058887	0.0379889
NUDT18	0.0252429	0.0381254

KCTD18	-0.0137223	0.038191
ETFDH	-0.029483	0.0382573
GNAS	-0.1353145	0.0383071
RIMKLB	0.0282148	0.0383217
ILDR2	0.009169	0.0384247
ATP23	-0.0322411	0.0384473
TMEM43	-0.0567548	0.0384514
TINF2	0.0222752	0.0386084
ZNF48	-0.0051278	0.0386518
ICOSLG	-0.0373805	0.0386841
TXNL1	-0.0160376	0.038704
LPP	0.0184975	0.0388807
PLXNB2	0.0086236	0.0392354
PIK3R6	0.0231861	0.0392723
PCNP	-0.0724965	0.0394046
PPM1H	-0.0459032	0.0394057
RABAC1	0.1745913	0.0394378
NSMCE4A	0.0270153	0.0394525
AHCTF1	0.0154938	0.0395708
APOL4	0.1074546	0.039661
UFM1	-0.0641973	0.0396702
INO80	-0.0011692	0.0397406
TRMT6	0.00981	0.0398838
RAD9A	-0.0106722	0.0399157
ZNF580	0.0406991	0.0399938
TMEM60	0.0343842	0.0399993
APOBEC3C	-0.0022724	0.0400803
NPRL3	-0.0058335	0.040156
TRIM14	0.0514083	0.0402432
NCOA1	-0.0988814	0.040252
SOX12	-0.0343506	0.0402566
DNAAF4	-0.0733506	0.0403737
GLOD4	0.0059761	0.0405366
HERC3	-0.0177154	0.0405394
MYO5A	-0.0621595	0.0405622
NOL4L	-0.0774182	0.0406412
TK2	0.0029856	0.0406528
HNRNPDL	0.1301441	0.0408734
SRRD	0.0193277	0.0410551
TCF20	-0.0342633	0.0411079
RECQL	-0.0177321	0.0411438
Z97192.1	0.0078693	0.0414034
GSR	-0.0290356	0.0414424
OBSL1	0.0151278	0.0415508
IL15RA	-0.0061171	0.0416184
SFR1	-0.0377933	0.0416332
ASCC1	-0.0186267	0.0416574
KLHDC2	-0.0096005	0.0417392
WDR34	0.0328261	0.0418059
CHN2	-0.0419558	0.0419317

ATF1	-0.0207845	0.0420556
PTPRC	0.1844386	0.0421138
CAMK1D	-0.0778579	0.0421384
ZNF727	-0.0282044	0.0421635
FBXO3	-0.0029995	0.0422205
CAMKMT	-0.0177117	0.0424467
AC055822.1	-0.009426	0.0426406
ABCC4	-0.2060258	0.0426646
TMX4	-0.0383473	0.0426679
TMEM147	0.0490069	0.0426828
RUNDC1	-0.023516	0.0428019
THOP1	0.0313018	0.0428315
G6PD	0.0273085	0.0428428
INO80B	-0.0590126	0.0430847
ASB8	-0.0242261	0.0432793
RAD1	-0.0507514	0.0432957
AC007383.2	-0.0405324	0.0432978
DDB2	0.0389359	0.0433153
NDUFV2-AS	-0.0383178	0.0433204
PRIM2	-0.0138709	0.0433314
AC087473.1	-0.039806	0.0436441
ATL3	-0.0153038	0.0436578
ZFP14	-0.0285126	0.043662
BICD2	-0.057921	0.0437053
NDUFA1	-0.1177659	0.0437091
PXK	-0.1390427	0.0437202
ARRDC4	0.0626178	0.0437254
CSF2RA	-0.0561719	0.043888
SLITRK4	0.115272	0.0439215
BOD1	-0.000454	0.0440494
RIOK3	0.0903066	0.0441519
TAF3	0.0694825	0.0442267
C2orf76	-0.0077342	0.0443801
HS6ST1	-0.0843894	0.0444703
ITGA6-AS1	-0.0103897	0.044553
DGKG	0.0034702	0.0445622
CYB5R1	0.0400783	0.0445907
HPS3	-0.0053799	0.0446327
DNASE2	-0.0282071	0.0446608
NUP155	-0.0398816	0.0447881
PRPF6	0.0058038	0.0448695
RALGAPB	-0.0112826	0.0449439
HLA-F	0.0485195	0.0451234
BCDIN3D	0.0003157	0.0452197
POLR3D	0.0714876	0.0452376
C3orf38	0.0098401	0.0454307
ECHDC2	0.0232853	0.0454459
OXSM	0.0226829	0.0455366
NKTR	-0.0902715	0.0455694
TIMM22	-0.0009404	0.0456055

ADCY3	-0.0171441	0.0456369
TBCD	0.0287956	0.0457266
CCDC122	-0.0391637	0.0457288
PPIL1	0.0040585	0.0457298
MVD	0.0109798	0.0458084
TBCE	-0.026502	0.0458298
VPS26B	0.0124741	0.0458589
KBTBD6	0.0021311	0.0459432
RCN1	0.0333915	0.0459506
ZNF235	-0.0141367	0.0459865
ZNF460	-0.0236532	0.0460478
MED9	-0.0148532	0.0462743
ZNF345	-0.0378236	0.0462962
GTF2E1	0.0250421	0.0464106
WDR91	0.0026962	0.0466341
USP24	-0.0551543	0.046904
C1orf35	0.0163075	0.0469349
UPRT	-8.514E-05	0.046975
ALG10	-0.0224213	0.047396
RCHY1	0.0276622	0.0475138
DPM1	-0.0078867	0.0475657
IQCK	-0.0041263	0.0475665
IFI35	0.0910719	0.0477633
TNFSF13	0.0255451	0.0478122
TCF25	-0.0221687	0.0479243
SLC9A6	-0.0114936	0.0479582
PSMD11	0.0159322	0.0481471
SEC24A	-0.0063539	0.0482595
FASTKD1	-0.041809	0.0484096
CYSTM1	-0.003483	0.0485776
FASTK	0.0401186	0.04861
LAMB2	0.0426875	0.0486795
STYXL1	0.0347617	0.0487082
ALDH18A1	0.0355599	0.0487707
SSR4	0.1470692	0.0488823
DHX32	-0.0072327	0.0488834
CDKN1B	0.0458696	0.0490451
AC020915.2	-0.0199813	0.0491102
RPIA	0.0022792	0.049141
UCK1	0.0071356	0.0491411
FOXJ3	0.0194639	0.0493773
TOLLIP	-0.0173513	0.0494442
AMMECR1L	-0.0248889	0.0495621
PANX2	-0.0074166	0.0495805
ZNF615	-0.0283185	0.0496729
MIB2	0.0206846	0.0496749
AL591895.1	0.005966	0.0497135
HNRNPLL	-0.0576931	0.0497163
REXO4	-0.0701913	0.0497215
PEA15	-0.019748	0.0497398

C17orf49	-0.1732159	0.0498214
RAB21	-0.0347754	0.0500621
TBC1D32	-0.0157433	0.0501156
ULK4	-0.0151916	0.0501358
TOP3B	-0.033027	0.0501709
FBXW8	-0.0041638	0.0503684
SLFN11	0.0179	0.0505563
TMEM209	-0.0199121	0.0508504
COMMD10	0.0128573	0.0509774
CMIP	-0.0693798	0.0511309
R3HDM2	-0.1384948	0.0511388
MSH2	0.0744614	0.0513669
BCAT2	0.0163424	0.0513995
ERCC5	0.0039701	0.0514325
EIF2S1	0.0324428	0.0515492
BOLA3	0.0282185	0.0516512
SAE1	-0.0163704	0.0517108
VAV1	0.0428707	0.0517156
NUP62	-0.021322	0.0517786
CBY1	0.0144713	0.0518047
EEFSEC	0.0015606	0.051905
BRPF1	-0.0115076	0.0519716
URB2	0.0038567	0.0520853
HSCB	-0.0353249	0.0522114
C1GALT1C1	-0.0223051	0.0523937
ANAPC16	-0.0512608	0.0524127
MARF1	-0.0543732	0.0525256
GFPT1	-0.02089	0.0526858
MAN2A1	0.0058674	0.0527316
AL356019.2	-0.029072	0.0528379
DDAH2	0.0224983	0.0528769
FAM126B	-0.0693916	0.0529165
PAXIP1-AS1	0.0335163	0.0529389
SH2D3C	0.0085373	0.0529652
DMTF1	0.0129664	0.0532
MAP2K1	-0.0009	0.0532479
ZDHHC2	-0.0198039	0.0533708
ZNF441	-0.0236215	0.0533996
P2RX5	-0.1020135	0.053411
TRGC1	0.0093571	0.0534283
ZNF706	0.0847126	0.0535403
KIT	-0.1331824	0.0535914
SLC29A3	-0.0110248	0.0536561
ZRANB1	-0.0601573	0.0536622
FAM3C	0.0042531	0.0536636
CENPC	-0.0736251	0.0538979
SRSF10	0.0258727	0.0539444
GLYR1	-0.0731889	0.0539984
DCAF7	-0.0699212	0.0541691
METTL16	0.0067147	0.054383

PPP2CA	-0.029037	0.054515
USP38	-0.0309918	0.0545517
MRTFA	-0.0499598	0.0546521
FBXO7	-0.0637865	0.054658
WWP1	-0.016703	0.0547571
PARVB	0.0859283	0.0547586
VPS28	0.1369366	0.05481
HIST1H1E	-0.1301623	0.0548422
SNAPC5	0.0392806	0.0548742
GID4	0.0054072	0.0548971
AC092821.3	-0.0036063	0.0549109
ACAP1	0.1694765	0.0549268
SRP19	-0.0346004	0.0550123
UTP23	0.0310313	0.0550971
RHOT2	-0.0024392	0.0551312
RRP1B	0.0722789	0.0554302
ZNF184	-0.0460778	0.0554618
MCM9	-0.0273199	0.0554727
CEP57	0.0424624	0.0556412
SNAI3	-0.0130578	0.0556931
IP6K2	-0.0370283	0.0557095
NUDT8	0.0892047	0.0557173
COA5	0.0208955	0.0558209
PHKA2	-0.0563065	0.0558724
TSC22D2	-0.0762322	0.0558757
RNASEH1-A	0.0301219	0.0559146
AC141424.1	0.0242654	0.05613
MFSD10	0.0127031	0.0561325
DOP1B	-0.0531893	0.0563674
NHP2	0.0999041	0.0565206
AC009961.4	-0.0094086	0.0567384
HIST1H2BN	-0.0330883	0.0568618
TAF5L	-0.0252739	0.0569225
MED19	0.0209558	0.0569308
NR2C1	-0.0579501	0.0573249
MIR17HG	-0.044775	0.0574594
C21orf91	-0.0434635	0.0575608
AC092338.3	-0.0286971	0.057677
SMPDL3B	0.0347679	0.0577014
CBLB	0.0106346	0.0577315
GMDS-DT	-0.0181851	0.0577508
TTPAL	0.0040802	0.0578694
TOMM40L	0.0095949	0.05787
SNAPC4	-0.0199315	0.0578892
CLUAP1	0.0237371	0.057901
TAF12	-0.0002134	0.0579587
SKIV2L	-0.0085069	0.0580625
SDF4	0.0297363	0.0581976
FBXO38	-0.0216857	0.0585444
RPARP-AS1	-0.0932888	0.0586046

HBEGF	0.0297626	0.0586577
NOP16	0.052006	0.0587359
AMPD3	-0.0117187	0.0588603
PRDX6	0.1444122	0.0590314
GLI4	0.0078619	0.0592987
ERLIN1	-0.0103316	0.0595094
KRCC1	0.0891847	0.0597133
PUS1	0.010771	0.0598415
BTN2A1	-0.0380131	0.0598423
LRRC23	0.0178406	0.0598869
ARHGAP11A	-0.0583545	0.059906
STARD3NL	0.0265845	0.0600087
MITF	-0.0380612	0.0601592
MAEA	-0.0068091	0.0601643
IPPK	-0.0169466	0.0601789
OCIAD2	0.0115592	0.060203
AIDA	-0.0014753	0.0602225
C2orf92	0.0284292	0.0602401
WDR3	0.0215462	0.0603067
ELP1	-0.0161693	0.0604249
FAM168A	-0.0155894	0.0604781
MCCC1	0.0384493	0.0606139
PEX11B	-0.0035425	0.0606582
ZNF439	0.0069828	0.0607321
LIPA	0.0394858	0.0608618
MRPL35	0.0341031	0.0609033
HS2ST1	0.0180102	0.0609266
G3BP2	-0.0018784	0.0610345
MRPL10	0.0246492	0.0610449
NRBF2	0.030715	0.0610751
MMACHC	-0.0269021	0.0611645
CFAP298	0.0729553	0.0612068
H1F0	0.0829934	0.061242
GALNS	0.0259754	0.0613028
RAPGEF6	0.0087978	0.0616563
AKAP7	0.0643911	0.061658
PEBP1	0.1666357	0.0618894
TNS3	0.0900009	0.0619337
LIN7C	-0.0501559	0.0621404
RLIM	-0.0060399	0.0621629
PUM3	0.0514998	0.0622146
FAM219B	-0.0253383	0.0622714
TWISTNB	0.1927213	0.0623785
HEXA	0.045948	0.0625429
SLC10A7	-0.0217129	0.0626246
PIGG	-0.0117506	0.0626476
CCDC91	-0.0055887	0.062672
CTPS1	0.0061289	0.0627024
AC005520.2	0.017778	0.0628786
BCKDHA	-0.0032853	0.0629358

NDUFS1	-0.1037589	0.062936
PTK7	-0.0280034	0.0629666
CCP110	-0.0109614	0.0631649
LRRC37B	-0.0227558	0.063357
REPS2	0.0106091	0.0634228
C6orf120	0.0544881	0.0634237
RAB28	-0.0212716	0.0634302
SIDT2	-0.0146988	0.0634625
AC009113.1	0.0106213	0.0634773
AP5Z1	-0.0277873	0.0634815
CLPB	-0.0167758	0.0635151
YBEY	-0.0200764	0.0636141
LIN37	0.0083601	0.0636494
TENT5C	-0.0732044	0.0636783
TBC1D31	0.0154159	0.0639411
AC023157.3	0.0407546	0.0639431
RTL6	-0.0331219	0.0641945
L3HYPDH	-0.0075338	0.0642334
ZFP64	-0.0311484	0.0644112
GPATCH2	0.0073659	0.0644886
WDR25	-0.0159207	0.0646777
DNAAF2	0.0377055	0.0647029
BCAT1	0.0932074	0.0647184
AL592435.1	0.0007481	0.0647264
PUS10	-0.0211089	0.064737
NFATC2	0.0580596	0.0647613
TM2D2	-0.0238846	0.0648742
MTMR14	-0.0242463	0.0649257
TSC1	-0.0369692	0.0649349
PHYH	0.0416954	0.0649674
ZNF654	0.0089389	0.0649869
TDG	-0.064557	0.0650654
PIWIL4	-0.05498	0.065141
TRAPPC6A	0.0303908	0.0652827
RAB11FIP2	-0.0447199	0.0654629
GBP2	0.0361555	0.0659726
PSMA3-AS1	-0.1272213	0.0659792
C7orf26	0.0090091	0.0662328
DCUN1D2	-0.029066	0.0663475
AC118549.1	-0.0228433	0.0664436
COX5B	0.1508335	0.0666551
AAAS	-0.0186647	0.0668601
PDK1	0.0414239	0.0669024
HIST1H2BD	-0.0453273	0.0669879
ZNF559	0.0004886	0.0670924
WDR13	0.0056635	0.067251
ZNF781	-0.0112564	0.0673147
RLF	-0.0183635	0.0674615
GPATCH8	-0.0374482	0.0676649
GCLM	-0.054935	0.0677327

HDAC4	-0.0089108	0.0681328
SDCCAG8	-0.0265842	0.0681496
ZNF346	-0.0010694	0.0683128
IRS2	0.0111412	0.0684343
NFKBIA	-0.0811245	0.0686354
EIF2A	-0.041532	0.0687911
NFKB2	-0.0477775	0.0691426
CRBN	0.1364713	0.0691955
MTMR10	-0.0374414	0.0693141
KIAA0753	-0.0037904	0.0694067
PSMB10	-0.1105365	0.0694199
AC127035.1	-0.025901	0.0694283
ACOT11	-0.0243326	0.0696361
GAPVD1	-0.0197165	0.0697416
ZNF277	-0.0472405	0.069855
MOCS3	-0.0095428	0.0698956
RMND5A	-0.0680947	0.0700796
IPO9	-0.0364455	0.0701676
SMUG1	0.0029284	0.0703787
PIAS1	-0.031242	0.0704118
MBTD1	-0.0329093	0.0704362
AL049840.4	-0.0042231	0.0705116
RNF214	0.0292929	0.0706838
ULK3	-0.003232	0.0708323
MMUT	0.0064317	0.0708696
TTN-AS1	-0.0760134	0.0708727
ABCD4	-0.0227975	0.0708807
DOCK11	-0.0679216	0.0709101
MAT2A	-0.0379386	0.0710663
TUBGCP4	-0.012664	0.0710713
PDS5B	-0.0909919	0.0711846
TCF7	-0.0196226	0.0712809
SCAMP2	0.0484949	0.0715923
AC008957.1	0.0005679	0.0718256
RPAP2	-0.1488048	0.0719443
CD84	-0.1129415	0.072167
PRTN3	-0.1585505	0.072273
CHD1	0.0406335	0.0722794
ICE2	-0.0248307	0.0725914
MAPK12	-0.0196228	0.0727228
SLC25A26	0.0144811	0.0731884
TAF6L	-0.0117129	0.0732918
KCTD11	-0.0252042	0.0733998
SFXN5	-0.0235086	0.0735437
TMEM62	-0.0245648	0.0736249
MTF1	-0.0083618	0.0738941
CLIP1	-0.0596871	0.0740634
AC104825.1	-0.0052435	0.0740708
NDRG3	0.0022403	0.0740862
WASHC3	0.0732963	0.0741892

PES1	0.0115496	0.0742282
TIGD1	-0.0115396	0.0742372
BRWD3	-0.0185976	0.0743512
ZNF574	-0.0129588	0.0743855
ARSB	-0.0547699	0.0743942
KIAA1586	-0.0381894	0.0744658
MON2	-0.0866883	0.0745995
ZNF394	0.0463379	0.0747098
IFI27L2	0.0587593	0.0751211
EIF4EBP3	0.0996385	0.0751442
MHENCR	0.0091779	0.075359
PDSS2	-0.0194755	0.0753607
EGLN1	-0.0051294	0.0755198
IAH1	-0.0109488	0.0760402
TMEM106B	-0.0205139	0.0760535
AAGAB	-0.0294729	0.0761176
SRXN1	-0.009772	0.0761565
RBM33	-0.0200267	0.0762585
IRF1	-0.0799971	0.0763045
DNAL4	0.024195	0.0765206
SERGEF	0.0056387	0.0767302
ADO	-0.0022282	0.0767988
PCGF1	-0.0068231	0.0771074
ZNF264	-0.0110759	0.0771413
ATAD5	0.0473215	0.0771868
UNK	-0.0395169	0.0772338
GON7	-0.0216144	0.07734
SLC45A4	-0.0186506	0.0774653
AL354740.1	-0.0344664	0.0774986
SERPINA1	-0.0489306	0.0775371
DHRS4-AS1	0.0112284	0.077589
STARD3	0.0061062	0.0776468
AL590764.1	0.0266898	0.0777981
NME6	-0.0047423	0.0778798
SPPL3	-0.0350848	0.0779322
TSPAN5	0.0509167	0.0779823
PAG1	0.0679718	0.0781463
NCOA7	0.0964018	0.0782005
SLC1A5	0.0558658	0.0782007
AP3B1	-0.018406	0.0783822
PSMG3	0.0095713	0.0784733
ZSCAN2	-0.037567	0.0785645
CTDP1	0.0105386	0.078621
CHKA	-0.0117278	0.0787998
UBC	0.2218114	0.0790229
AC004034.1	0.015122	0.079195
KLHL9	-0.0028021	0.0793124
ZNF577	-0.027229	0.0796612
PPP2R3C	-0.024628	0.0797924
YBX1	0.2369885	0.0797938

SH3YL1	-0.0042481	0.0798798
SLC39A6	-0.0037881	0.0798892
DNAJB14	-0.0431971	0.0799912
FAM204A	-0.0546906	0.0801901
YES1	0.0382529	0.0802502
ZDHHC12	0.0532302	0.0803818
CDC23	0.0225677	0.080575
NOL11	0.0053677	0.0806759
BORCS7	0.0553871	0.0806848
SCYL3	-0.0351175	0.0808493
AC246817.1	-0.0389343	0.0809665
GEN1	-0.0476237	0.0810442
AC008035.1	-0.0077408	0.0810493
FLYWCH1	-0.020961	0.0810619
ARSD	-0.1571463	0.0814634
CTSO	-0.0340401	0.0815459
ZNF564	-0.0385192	0.0816245
RHEX	-0.2570127	0.081742
CTBP1-DT	-0.0307453	0.0818267
ARID5B	-0.0607673	0.0818305
ZBTB22	-0.0013297	0.0819288
PTPN23	-0.0026745	0.0823113
IGHMBP2	0.015234	0.0823412
ACP6	-0.0048928	0.0823878
WWP2	-0.0261422	0.082404
ZNF827	0.0041628	0.082442
ENDOV	-0.0006667	0.0824502
TCTN1	0.0063264	0.0827261
MICOS13	0.1186226	0.0828913
DNTTIP1	0.0573573	0.0829402
BCL3	-0.020867	0.0832582
AGPAT1	-0.01021	0.0832959
CCDC174	-0.0373545	0.0832967
LINC01122	0.0002377	0.083349
AC092279.1	-0.03255	0.0836248
ZNF740	0.002707	0.0838412
SNHG17	-0.0102565	0.0839215
METTL6	-0.0050722	0.0840539
NDUFAF4	-0.0165963	0.0841206
EI24	0.0126174	0.0841605
TRPV2	0.0675799	0.0844291
MPZL1	-0.0228444	0.0845018
LINC00863	-0.0417329	0.0848495
LINC01637	0.015126	0.085369
STX3	0.0551169	0.0854737
PDP2	-0.0140248	0.0855837
IL16	0.0539897	0.0859552
MTA1	-0.0163092	0.0860486
ATG4C	0.0138661	0.0867556
AC139530.1	-0.0070591	0.0869487

LRRC8D	-0.0188081	0.0873994
RNF139	0.0026658	0.0876653
UBR4	-0.0212018	0.0877071
UBE2J1	0.0818491	0.0877536
N4BP2L2-IT2	-0.0284571	0.0877586
CXorf21	-0.0392569	0.087965
LEPROTL1	0.0329758	0.0880392
ACTR3C	-0.0236606	0.0882284
AP1S1	0.0680283	0.0883273
SERAC1	-0.0398515	0.0884215
CYP7B1	0.0574821	0.0884744
EEA1	-0.0165094	0.0885618
SRSF4	0.0346665	0.0885628
FBXL19	-0.0131361	0.0886744
AP4S1	0.0391467	0.0887642
EHD4	0.046745	0.0887647
ZNF605	-0.0572737	0.0889111
GATC	-0.041236	0.0889775
KHDRBS1	0.0339681	0.0889853
DDX55	-0.0491116	0.0891991
CYFIP1	0.0228069	0.0895127
C5orf63	-0.015183	0.0895812
ENOSF1	0.0490436	0.089741
SLC38A6	-0.0221684	0.0900766
SUGT1	0.0403517	0.0901464
ZNF790	-0.0052393	0.090274
RIC1	-0.0559198	0.090451
TASP1	0.0037743	0.0904988
ELOF1	0.0057422	0.0905003
CLASRP	0.0036646	0.0905302
SLC26A1	-0.0541098	0.0906924
GGCT	0.0512043	0.090834
SIRT5	-0.0210022	0.0908791
CTNNA1	-0.0435084	0.0909202
FBXO32	-0.038389	0.0910402
GCSH	0.0297741	0.0910957
SLCO4A1	-0.0483427	0.0915581
TRAF7	-0.0227688	0.0917583
NDUFS2	0.0182002	0.0919626
DNAJA3	-0.0044841	0.0919669
ZNF586	-0.0423461	0.0920598
SGF29	-0.0215195	0.092068
TMEM186	0.0354674	0.0921245
AC012306.2	-0.0236737	0.0921322
GRPEL1	-0.0381877	0.0923646
GPI	-0.056552	0.0925247
FH	0.0144944	0.0925728
PCCB	0.0070197	0.0931953
SNRNP70	0.0168985	0.0936091
AZIN1-AS1	-0.0077646	0.0936164

GGPS1	-0.0260998	0.0938166
PSMD10	-0.0022095	0.0944682
MRE11	-0.0100698	0.0947014
TRIM71	0.0722408	0.0947155
AKT1S1	0.0176512	0.0951938
TMCO3	-0.0538077	0.0954655
NAF1	-0.0420865	0.0954702
ADGRG3	0.0477152	0.0954922
TEFM	-0.0282157	0.0956726
MAP3K2-DT	-0.031434	0.0957447
ADAR	-0.0663769	0.0958339
LGALS9	-0.1151992	0.0958461
EXD3	0.0249642	0.0959772
MRM3	0.0208152	0.0961568
CEP170	0.0104249	0.0965839
SLC35B3	-0.0450903	0.0971349
INTS3	0.0058631	0.0973058
MALRD1	-0.0013325	0.0976837
TMEM220	0.0175248	0.0979566
COL9A2	-0.0227413	0.0984708
UBXN11	0.0329594	0.098789
IFNGR1	0.0339467	0.0988619
MRPS30	0.0030226	0.0988989
C12orf4	-0.0236468	0.0991054
AC104041.1	0.0202161	0.0991621
PPP1R37	-0.0217173	0.0996034
RBBP9	-0.0107719	0.0997981
TADA1	-0.0133099	0.0999216
MRPL45	-0.0231113	0.1000538
LPIN2	-0.0738912	0.1003662
TKFC	0.0133569	0.1004078
KLF16	-0.0209248	0.1004575
PBX2	-0.0351373	0.1005214
DAXX	0.0360831	0.1007942
SLC39A13	-0.007463	0.1011469
FECH	0.0190232	0.1012606
NUDT15	0.0082141	0.1014545
ZNF185	0.0844969	0.1015094
ZNF598	0.015161	0.1022121
MAP1LC3B	-0.0333469	0.1022746
CEP135	0.0256423	0.1023116
SIRT6	0.0049688	0.1027512
LINC01184	0.0310002	0.1030294
AC012358.3	-0.028845	0.1034785
DHRS4L2	-0.0045583	0.1035174
UNC93B1	0.1093223	0.103727
FEM1A	-0.0237223	0.1039219
NSUN4	-0.0020096	0.1042293
STAT3	0.0378029	0.1043628
GATA2-AS1	0.0446723	0.1044386

MPZ	-0.0387892	0.1045753
TTC28-AS1	-0.0414692	0.1048384
ERVK13-1	-0.0195742	0.1048553
MANEA-DT	0.0047885	0.1049375
HNRNPAB	0.1489196	0.1053942
AL135791.1	-0.0055826	0.1054498
MYLK-AS1	0.0117987	0.1054837
UBR7	-0.0036926	0.1056842
PIGU	0.0039534	0.1059418
CD34	0.1165508	0.1059605
MED11	0.026477	0.1063805
TXNRD2	0.004285	0.1064263
FBRSL1	-0.0196276	0.1071161
AC040977.1	-0.0164588	0.1071889
TRIM38	-0.0713702	0.107197
ANKRD42	-0.0160658	0.107587
GUCD1	-0.0730321	0.1077914
SSBP2	0.069207	0.1077916
MAP3K4	-0.0081169	0.1079042
ERICH1	0.0431516	0.1080693
USP16	0.0225174	0.1082969
DAD1	0.0814407	0.1087217
KCTD13	-0.0395966	0.1087807
MTPN	0.145491	0.1088689
PLEKHA2	-0.0727877	0.1094552
BICRAL	-0.0142159	0.1096497
DHDDS	-0.0070821	0.1097041
CIC	0.0187185	0.1098487
ZNF606	-0.0204666	0.1105648
FANCG	0.0160512	0.1105655
NEDD1	-0.0179402	0.1106355
GGNBP2	-0.0074768	0.1106661
PWWP2B	0.0203327	0.1110712
AL355488.1	-0.0265942	0.1110986
GTF3C4	-0.0103004	0.1112673
ZNF816	-0.0049098	0.111321
RAE1	0.0099674	0.111384
LIAS	-0.0046884	0.1114513
BTBD8	0.0041738	0.11156
SIGLEC6	-0.0645656	0.1116542
PPID	0.0383126	0.1117057
RAB20	0.0489342	0.111969
GCHFR	0.1048757	0.1121288
TMEM168	-0.0296852	0.1126935
ZNF644	-0.0197902	0.1129401
COPE	0.1309874	0.112968
MED29	0.020722	0.1130048
AC090825.1	-0.025015	0.1132802
MEN1	-0.0030621	0.1137587
HOTAIRM1	-0.1344475	0.1139328

KIF27	-0.0523019	0.1141121
ZNF133	-0.0008266	0.1142443
BBX	0.1203444	0.1143651
KRT10	0.0311963	0.1143873
AL080317.2	-0.0450452	0.1143908
TBPL1	0.0430014	0.1145736
SAP30L-AS1	-0.0215288	0.1151334
AC090114.2	-0.0136508	0.1156311
Z93241.1	0.0605113	0.1159176
TBL1XR1	-0.0051603	0.1159767
BCS1L	0.0117886	0.1162008
FAM193A	0.0074128	0.116209
THAP3	0.0207729	0.1164855
HNRNPL	-0.1212201	0.1164977
TFB1M	0.0065322	0.1165604
ZNF230	-0.0257507	0.1175591
MT2A	0.1223837	0.1175986
AGTPBP1	-0.0239867	0.1179947
SLC38A5	0.0265626	0.1180701
IFNAR2	0.0245953	0.1185523
CCDC51	0.0550961	0.1185643
RPS6KA3	-0.1113149	0.118994
ZNF573	-0.0040147	0.1195528
HPS4	-0.0097254	0.1196098
PDS5A	-0.0469483	0.1196491
TMEM44-AS	0.046749	0.1197732
TGFB1	0.0999329	0.1198286
L3MBTL1	-0.0129325	0.1201721
TMED1	0.0219695	0.1203446
GOPC	0.0871296	0.1206871
VPS13C	-0.1412858	0.1208638
BICD1	0.053091	0.1210055
B4GALT3	-0.0163	0.1216601
XPA	0.0163042	0.1217448
HACL1	-0.0226727	0.1219446
KRT18	0.0879118	0.1219768
PRPF38B	-0.077375	0.1220529
RAB22A	0.0229174	0.1221569
ZSCAN32	-0.0061904	0.1222852
TRMT10B	-0.0231226	0.1223748
DEPDC5	-0.0286359	0.1224423
TFAM	0.0906606	0.122463
ZMYM5	0.0156953	0.1224802
SH3BP5-AS1	-0.0159472	0.1226398
TMEM223	0.0780642	0.1227023
SUPT6H	0.028052	0.1230067
TRNAU1AP	0.0371724	0.1231549
DLGAP4	0.0358641	0.123243
REXO1	-0.0007721	0.1232928
ZNF266	-0.0340198	0.1236134

USP37	0.030041	0.1238287
RDH13	0.0081445	0.1243769
MMP19	-0.0237415	0.1246678
AC008982.2	-0.0180222	0.1247448
SLC9A7	0.027471	0.1248841
MUL1	-0.0082295	0.1251054
TP53BP1	-0.0034961	0.1253727
KATNBL1	-0.0442514	0.1254539
NCKAP5L	-0.00791	0.1257324
FO393401.1	0.0444112	0.1260025
ZNF347	-0.0132102	0.1262511
ZCCHC2	-0.0413834	0.1262908
PLEKHA5	-0.026373	0.1263217
FAM241A	0.0632459	0.1265137
IL2RG	0.1119042	0.1267176
TSEN54	0.0440394	0.1268061
PDE6B	-0.0009386	0.1268142
C6orf136	0.0002389	0.1270031
FAAP100	0.0074945	0.1271611
YAE1	-0.0003314	0.1272534
GPD2	-0.02726	0.1274178
MLEC	-0.0036445	0.127424
SNAPC2	0.0015319	0.12761
UBR5	-0.0523497	0.1278864
GTF2H1	0.002854	0.1281283
ZSWIM6	0.0228904	0.1281811
NAA10	0.0552968	0.1282712
TMEM156	-0.0264022	0.1284265
HYOU1	-0.0049827	0.1288749
RTTN	-0.0286669	0.1289883
ANKRD13C	0.0207242	0.1290384
HYAL2	0.0448119	0.1290478
RBM45	-0.0238632	0.1293534
AC008074.2	0.0091157	0.1295223
KLF7	-0.0194169	0.1295754
SUZ12	-0.0094595	0.1297514
KCTD20	-0.0422008	0.1299881
ARID1A	-0.0181275	0.13045
AC067852.2	0.0050307	0.1308266
AC015871.7	-0.0314961	0.1308916
SNIP1	-0.024581	0.1310419
C11orf1	-0.0604262	0.1316997
MEAF6	0.036204	0.131835
PPTC7	-0.0024603	0.1319521
PRADC1	0.0013589	0.1319588
ERI2	-0.0283216	0.1323916
INO80C	-0.0107865	0.1329788
TLNRD1	0.0045514	0.1331451
PHLPP1	-0.0007623	0.1331662
IWS1	-0.0174648	0.1333385

SIMC1	-0.0195757	0.1333559
DYRK1A	-0.0346127	0.1333785
NKRF	0.0200251	0.1342856
FSD1L	-0.0043376	0.1346238
COQ2	0.0201123	0.1348047
IKBKG	-0.0017215	0.1352415
LRR1	-0.0072657	0.1354965
ZNF234	0.0229874	0.1355215
CELF1	0.0145831	0.135762
SLC41A3	-0.0192824	0.1359676
CSTB	-0.0172997	0.1361753
DLEU2	-0.0696519	0.1365844
ZNF93	-0.071575	0.1366944
RAD18	-0.0413037	0.1368258
IGF2BP2	0.0493593	0.137746
AL080276.2	-0.0329968	0.1378219
SYNJ2BP	-0.0442847	0.137862
ZC3H13	-0.0746873	0.1379441
GPALPP1	-0.0084422	0.1386281
IL17RC	0.0390884	0.1388745
LATS2	0.0748642	0.13907
RBM27	0.0699692	0.1392289
GATAD2A	-0.0095534	0.1401754
ZNF789	-0.0298754	0.1402694
SPRYD7	-0.0301712	0.1411898
SORD	0.0546672	0.1413981
BSCL2	-0.0189047	0.1416461
PPP2R5E	-0.0496029	0.1419072
PDCD2	0.0161363	0.1421382
NMNAT1	-0.002954	0.1421641
NPAT	-0.0096505	0.1422009
MEGF6	0.0082473	0.1422891
SNX30	-0.0522793	0.1424243
DDX50	-0.0391397	0.1426065
MKLN1	-0.0800032	0.1426837
BRF1	-0.0306558	0.1429935
NIT2	0.0393765	0.1431428
IL27RA	0.032654	0.1435427
ASB3	0.00433	0.1437789
DYRK3	0.0879609	0.1438252
RPL26L1	-0.0073127	0.1441856
PROCA1	0.0021418	0.1443166
NRIP1	0.1388723	0.1445655
RHBDD1	-0.0120832	0.1450877
FMO5	0.0206208	0.1456942
PTDSS2	0.0199118	0.1460527
ALG9	-0.0026717	0.1462203
CCDC130	0.0526824	0.1462468
CERK	0.002412	0.1465684
RPL32	-0.0734251	0.1467898

NGLY1	0.0006165	0.1472575
KANK1	-0.031585	0.1474126
CIB1	-0.0910959	0.1474732
FURIN	-0.1146232	0.1478159
NDST2	-0.0224959	0.1479105
POLR3H	0.037766	0.1480064
AREG	-0.0273745	0.1480332
AC135507.1	-0.0173012	0.1483876
ARSK	-0.020492	0.1485067
XRCC5	0.1402323	0.1488692
PLIN3	0.0081021	0.1490833
BLOC1S1	0.0763383	0.1490958
AC009061.2	-0.0112651	0.1492166
AP000766.1	-0.0258049	0.149813
CPSF6	-0.1008081	0.1498883
SLC35E1	0.0076743	0.150492
NXPE3	-0.072702	0.1507953
WIPF2	0.0034235	0.1510204
C10orf25	-0.0134467	0.1513099
NCEH1	-0.020965	0.1513563
OSBP	-0.0013427	0.1515553
WDR20	-0.0189622	0.1522356
RSKR	-0.0014767	0.1523584
IGFBP2	0.1791427	0.1525988
CLCN3	0.0018392	0.1530828
DUS4L	0.0136221	0.1533903
AL354822.1	-0.0368188	0.1534017
PPP2R5C	-0.0287552	0.1539725
CGAS	-0.1014202	0.1542904
FAS	0.0314808	0.1547294
PSMC6	-0.0512298	0.1549131
MPLKIP	0.0332911	0.1549563
UBE2D2	0.1265991	0.1549885
AC079922.2	0.010707	0.1550135
WDR74	0.0357018	0.1557654
EID2	-0.0274089	0.156104
DGLUCY	-0.0245934	0.1562126
TTC4	-0.0317203	0.1562666
FAM76A	-0.0445598	0.1562854
SPECC1	-0.0265382	0.1564624
CDK2AP1	0.0596	0.1566309
MCMBP	-0.0536702	0.1566776
ZNF561-AS1	-0.0286759	0.1567787
PCIF1	-0.0419006	0.1569181
TAPBP	-0.0879785	0.1569903
AL390195.2	-0.0270283	0.1573229
MAST4	0.083598	0.1573241
INTS6-AS1	-0.0158269	0.157922
AC048341.1	-0.0372453	0.1581462
CCDC30	-0.0723581	0.1582429

AL096803.2	-0.0197554	0.1587955
UBQLN2	-0.0366398	0.1588262
SLC12A2	-0.094977	0.159054
ERGIC2	-0.0273636	0.1591917
ISG20L2	-0.0506281	0.1595199
MDH1	0.0197252	0.1597554
TBL2	-0.0018224	0.1598541
FRS2	-0.0208322	0.1610045
TNFRSF1A	0.0157883	0.1611323
PLGRKT	0.0010132	0.1613609
SIAH1	-0.0274019	0.1614185
NBPF10	-0.0225327	0.1618103
PMPCA	0.027143	0.1620452
IKBIP	-0.0211493	0.1620838
MINCR	-0.0663541	0.1622775
MTHFS	-0.046307	0.1624524
ZNF267	0.0183302	0.1626142
AC007406.5	-0.0086996	0.1626352
CXorf40A	-0.0094846	0.1627155
ZSCAN9	0.0186482	0.1630955
TRIM24	-0.0603435	0.1630967
TEPSIN	-0.0178044	0.163234
CHCHD4	-0.0210656	0.1634028
GTPBP4	0.0714302	0.1640309
CDKAL1	0.0011737	0.1649541
HTATIP2	-0.0003634	0.165376
PRAF2	0.0138055	0.1660075
NR2F6	0.041008	0.1660715
CLN8	0.05993	0.1668257
ZSWIM9	-0.002833	0.1669688
TRMT2A	0.0305292	0.1669844
ZBTB34	-0.0351326	0.167453
NRM	0.0637508	0.1679102
AC016747.1	0.0438234	0.1679434
SLC5A3	-0.014735	0.1680581
SRA1	-0.004838	0.1680708
PRKACA	-0.0111223	0.1681161
CAST	0.1337396	0.1682552
SNRNP35	0.0591471	0.1687557
SLC66A1	-0.0023691	0.1688216
MED18	0.0404297	0.1689194
MED23	-0.0311837	0.1689872
WRAP73	0.0061613	0.1690562
DHX16	0.0207817	0.1696521
JARID2	-0.0926109	0.1698092
RPS6	0.0951243	0.1699295
NEMP2	0.0082776	0.1700599
RAD51-AS1	0.0607017	0.1706949
FADS3	0.0206021	0.1708615
IL6ST	0.0673048	0.1710537

CCNY	-0.0039966	0.1713036
DYNC2LI1	-0.0230551	0.1715435
TOPORS	0.0347639	0.1719295
NOC3L	0.0133148	0.1723318
ARCN1	0.0365164	0.172495
GIMAP6	0.0224087	0.1725172
ANKFY1	-0.0169949	0.1726414
GAA	-0.0062565	0.1732098
TAF6	0.0191627	0.173282
FANCI	0.042768	0.1736651
ZNF28	-0.0624573	0.1743011
ACOT13	0.0331996	0.1743589
MCTP2	0.0736993	0.1747654
YIPF2	0.0321289	0.1749448
CORO1B	-0.0085158	0.1750336
CALCOCO2	0.0398056	0.1761369
VPS13D	-0.0386487	0.1763831
RPL4	0.1591388	0.1770049
REEP3	0.0050915	0.1774399
PISD	0.0289953	0.1776422
RDH11	-0.0317515	0.177793
EPB41L4A-A	-0.0766749	0.1783624
TENT5A	-0.1330382	0.1785633
FN3KRP	-0.0261526	0.1792586
TXNDC11	-0.0236274	0.1794134
SPNS2	0.0385271	0.179605
BFAR	0.0266556	0.1798727
AC008969.1	-0.035785	0.1798777
HOXB-AS3	0.0347412	0.1802115
CDC73	-0.0476962	0.1810177
CLCC1	0.0466693	0.181176
ZNF496	0.0029887	0.1816852
THAP1	0.0306233	0.1816904
CTNS	-0.006351	0.1820434
LNCAROD	-0.1124624	0.1822732
BNIP3L	0.0175715	0.1827791
GTF3A	0.1587167	0.1828651
AKR1C3	0.1335021	0.18362
COMMD8	-0.0383866	0.1836454
CIITA	0.0493504	0.1838881
FBXO6	0.0135434	0.1840219
NUP50-DT	-0.0081102	0.1842962
ZFP3	-0.001316	0.184492
REEP4	-0.0317083	0.1846363
ADGRG6	0.102659	0.1853691
NFRKB	-0.0317335	0.1861039
DTWD2	-0.0422354	0.1866446
PRKCI	-0.0292834	0.1867567
RBBP5	-0.0414979	0.1869691
NMRK1	0.0019938	0.18727

VRK1	-0.0039285	0.1876485
KLF3	0.0594711	0.1877288
TTC33	-0.0145198	0.1884188
FAM153CP	-0.0156184	0.1886857
INSIG2	-0.0144092	0.1894806
TCF15	-0.0318101	0.1897438
ZDHHC17	-0.0344772	0.1905763
DDX19A	-0.0497221	0.1905955
C12orf65	0.035663	0.1906006
HMGCS1	-0.1311789	0.1907563
CENPBD1	0.0154725	0.1910629
GMPPA	-0.0034035	0.1913123
BORCS6	-0.0172431	0.1917906
KIAA1328	-0.0157771	0.1917976
IRAK1BP1	0.0195745	0.1918782
SNW1	-0.0577358	0.1919706
AC008124.1	-0.0344503	0.1930564
ANXA7	-0.1250225	0.1937427
SIRT1	-0.0288743	0.1938268
PRR13	-0.1387833	0.1940296
INTS1	0.0195295	0.1943374
MRI1	-0.0171215	0.1943689
SELENON	0.0112516	0.1945939
TMEM39B	-0.0048417	0.1947554
TMEM147-A	-0.0227318	0.1948571
LAMTOR4	0.1019095	0.1950349
ZNF180	0.0066121	0.1953012
BBS4	-0.0129431	0.195582
AKIP1	0.0460459	0.1959918
KLHDC10	-0.0240498	0.19601
RFX3	-0.0173672	0.1962498
GIGYF2	0.0252445	0.1963002
UBE4A	-0.0423943	0.1965268
LMBR1	-0.0020312	0.1965959
TBXAS1	0.0176218	0.1978712
CASP7	0.0273166	0.198013
RGP1	0.0382131	0.1991407
AC107214.1	0.0092605	0.1991899
RAB5IF	-0.0111034	0.199635
GGCX	-0.0856669	0.2002862
XPR1	-0.0335056	0.2005032
RPUSD3	0.0219633	0.200537
THAP6	-0.0092141	0.2005884
TIMMDC1	0.0531698	0.2006195
ACSF3	0.0538583	0.2011041
EIF2S2	-0.0190898	0.2013539
FLVCR1	-0.0359068	0.2015378
RBM22	0.0015393	0.2016466
RAP1A	-0.0710024	0.2020881
CPEB2	-0.0416311	0.2026703

ANKRD54	0.0443977	0.2028391
RCL1	-0.0023545	0.2028847
GOLGA2	0.0337771	0.2029479
YEATS2	-0.014311	0.2031087
FARSA	-0.0679435	0.2031815
SLC29A1	-0.0465422	0.2031989
COA6	0.03392	0.2034032
HSDL2	0.0690881	0.2035075
EDEM3	-0.1208003	0.2038425
ZNF674	-0.0128955	0.2041505
MLXIP	-0.0216697	0.2054026
SREK1IP1	0.0249404	0.2054031
ANKRD13A	0.0098852	0.2056506
MAST3	-0.0505945	0.2061129
ADAT2	-0.0181267	0.2064165
NOP56	0.1128382	0.2064997
REL	-0.0302651	0.206571
CLPTM1L	-0.0148278	0.2075818
MRTFB	-0.0222221	0.2075956
PRNP	-0.0045848	0.2076591
FGFR1OP2	0.1006904	0.208033
DDX31	0.010048	0.2082755
SLC31A2	0.0032061	0.2094531
SNX6	0.0833713	0.2097007
MFAP3	0.0066765	0.209978
UBALD2	0.0431642	0.2102056
SPATA13	0.0360832	0.2107289
AL157834.1	0.0075529	0.211319
TMEM87A	-0.0298263	0.2115338
CRAMP1	-0.0362751	0.2119086
MBD6	-0.0195746	0.2120483
SUGP2	-0.1041846	0.2128369
G3BP1	-0.0836048	0.2129652
FES	0.027337	0.2133346
TAS2R30	-0.0232268	0.2135809
AC010745.5	-0.0389285	0.213953
TSR2	0.0138621	0.2148657
MED8	0.005067	0.215302
SZT2	-0.032662	0.2157534
ASB13	-0.0172765	0.2161716
RUFY2	-0.0242757	0.2162016
NR1H3	0.0559353	0.2173611
KCTD3	-0.0129338	0.217566
HECTD3	-0.0056995	0.2177981
RTCA-AS1	-0.0316655	0.2178872
AIFM1	-0.0136024	0.2182386
C1QTNF6	-0.0352252	0.2182871
ALG5	0.0460364	0.2184806
ANP32E	-0.0218216	0.2192157
U2AF1L5	-0.0233542	0.219577

MOB1B	0.1116599	0.2201929
MRPL1	-0.0015556	0.2203964
SHPRH	0.0047648	0.2216238
PIP4K2A	-0.0128554	0.2222035
ITGB3BP	-0.0030627	0.2236112
MRM2	0.0804648	0.2239328
NDUFS7	0.0272194	0.2240262
ZNF555	-0.0546305	0.2244234
NDUFAF7	-0.0385572	0.2248847
AL391069.2	-0.0885306	0.2250153
ARHGEF18	-0.0145472	0.2262623
USP53	0.0066323	0.2264863
POLR1A	0.0075613	0.2269256
FXN	-0.0363023	0.2276206
KDM4A-AS1	-0.032563	0.2279977
TP53TG1	0.0493002	0.2280043
IFT172	-0.0181544	0.2280312
AL009179.2	-0.0379828	0.2280809
AC002464.1	-0.0171583	0.2282801
EXOSC3	0.0515604	0.2284205
NIPA1	-0.0290203	0.2293857
FRMD8	-0.046285	0.2294301
SMARCC2	-0.0340724	0.2297558
ZFYVE1	-0.027191	0.2300517
PLAAT4	0.0711743	0.2303036
CDK12	0.0457147	0.2303428
MAP4K2	-1.327E-05	0.2314281
MAPK14	-0.0382611	0.2317533
ZNHIT2	0.0275633	0.2318818
UQCRHL	-0.02273	0.2333409
ARFGAP3	0.0771603	0.2334631
COG1	-0.0202503	0.2335239
UBA3	0.0065636	0.2339278
MTERF3	-0.0170142	0.234048
DUSP12	-0.0201715	0.2340894
ITPRIPL2	-0.073449	0.2341136
YIPF4	-0.1685006	0.2341922
AC006449.6	-0.0160855	0.2351279
IRF2BPL	-0.04539	0.2353092
NME8	0.006493	0.2356482
TANGO2	0.019133	0.2365198
MTMR1	0.0158813	0.2366747
GLIDR	-0.0074465	0.2370753
AL117336.2	-0.0345163	0.2378992
U2AF1L4	-0.0308489	0.2393303
EMC10	0.0171153	0.24026
HCG25	-0.023519	0.2403943
CCDC86	-0.005702	0.2410866
NUSAP1	0.035456	0.2419816
HSH2D	0.0900786	0.2420042

POLI	-0.0176081	0.2435162
DNAJA1	0.0401251	0.243987
EBLN3P	0.0095832	0.2442936
AHI1	-0.0362795	0.2449058
APIP	0.0067398	0.2452359
AL590064.1	-0.0120344	0.2453291
RBMS2	-0.0391838	0.2456058
ZNF569	-0.0307764	0.2463274
MBNL2	0.0045271	0.247064
ZNF420	-0.0032273	0.2470958
MED1	-0.0143228	0.2476981
TRIM46	-0.0019159	0.2485082
MARC2	0.0134833	0.2501078
CORO7	0.0262929	0.2504776
RAB12	0.0176352	0.250605
SLC7A5	-0.0586166	0.2510036
ZNF618	-0.0249683	0.2515598
TRMT112	0.0946064	0.2521018
HCG11	-0.0540234	0.2522049
PAF1	0.0236711	0.2534143
ZNF428	-0.0101596	0.2534239
ZNF529-AS1	-0.0135848	0.2551263
RBMX2	0.0276164	0.2554918
PYCR1	0.014194	0.2558365
SFI1	0.0114542	0.2573082
TMEM59	0.1160074	0.2579076
ZNF253	-0.0096127	0.2580662
EDRF1	-0.0129413	0.2583717
MAPKBP1	0.0227834	0.2586275
CCDC186	-0.006897	0.2600328
DARS	0.0353055	0.2601846
DCAF15	-0.0189003	0.260705
RBM26-AS1	-0.0313968	0.2610976
PTGER2	0.0675973	0.2621413
EYA3	-0.006518	0.2623929
AHCYL1	0.0053795	0.2625855
RPP14	-0.0208499	0.2628902
THNSL1	0.0037995	0.263201
STK16	0.0223546	0.2643371
EIF2AK4	0.0213618	0.2644668
CHSY1	-0.0885262	0.2652831
FGFR1OP	-0.0202816	0.265642
SNTB1	-0.0403843	0.2657805
FASTKD5	-0.0284911	0.2658752
RAB32	-0.1713403	0.2662187
SLX4IP	0.0647715	0.2667834
KLHL23	0.1081044	0.2676953
DCAKD	-0.0157799	0.2678523
TUBGCP3	-0.0194355	0.2681254
FCSK	-0.0131635	0.2682418

RPRD2	0.0077859	0.2683911
PPP5C	0.0372695	0.2693936
ARHGAP9	-0.0169634	0.2694778
LENG8-AS1	0.031199	0.2695507
CCL23	0.0588533	0.2695703
HDGFL2	-0.0187997	0.2700756
ARMH3	0.0099063	0.2706585
MARS	0.0402662	0.2707108
CYBRD1	0.0596792	0.2711635
ZNF668	-0.0094899	0.2711648
FOXO4	-0.0205858	0.2712013
YPEL1	-0.0036901	0.2722833
RASGEF1A	0.0067769	0.2726479
MPI	0.0410138	0.2726898
KDM1B	0.0035939	0.2728437
BBS1	0.0098236	0.2728437
AC102953.2	-0.0137903	0.272856
RPLP1	0.0825103	0.2730926
LBR	-0.0114517	0.2735128
BIN2	0.114145	0.2740532
DDX60L	-0.0020013	0.2745853
SREK1	-0.0594137	0.274941
TWF2	0.0496279	0.2750315
BEST1	-0.0338322	0.2757995
TCIRG1	0.0224497	0.2759687
PITRM1	0.0100765	0.2760124
PGPEP1	0.0120244	0.2760275
FUBP1	-0.1003602	0.2760597
RCOR1	-0.0769733	0.2765471
DNAJC14	-0.032536	0.2765917
LINC02193	-0.0232437	0.2767044
ADHFE1	-0.019027	0.2769187
RAD54L2	-0.0015215	0.2771131
PDXP	0.0193843	0.277361
NAPG	0.0146381	0.2780712
ZNF501	0.0107727	0.2783093
KDM4B	-0.0110513	0.278713
NAA35	-0.0567995	0.2789845
PARP12	0.0074116	0.2792952
AC005726.5	-0.021218	0.2800655
ACOT9	0.0122335	0.2805657
AP003486.1	-0.0192003	0.2815102
HOXB5	-0.0454556	0.2828786
ZNF518B	-0.019565	0.2835905
SLFN13	-0.0290671	0.2838299
AC096992.2	-0.006845	0.2849714
CNOT2	-0.0588373	0.286234
ALG1	0.0115956	0.2867466
MPHOSPH8	0.0361677	0.2870088
NPIPB2	-0.0381998	0.2879714

ZCWPW1	0.0071896	0.2887549
PDCD6IP	-0.1207874	0.2888878
EXOSC2	-0.0097484	0.2893436
ARHGAP30	0.1293225	0.2894318
CAPN10-DT	-0.0420739	0.2899224
NCDN	0.0022825	0.2905369
ARMCX1	0.0581961	0.2905865
BET1	-0.0104692	0.2906842
MYO9A	-0.0087536	0.2914617
ICA1	0.0571859	0.2915137
AC107464.3	0.0320474	0.2915771
ZEB1	0.0609832	0.2916173
AGTRAP	0.0095912	0.292079
MAP7D1	-0.0338197	0.2923724
TFDP2	0.0954377	0.2935163
TMCO4	-0.0088203	0.2936358
APOL2	-0.032469	0.2937593
BAZ2B	-0.1415828	0.2943504
CUL3	-0.0004738	0.2962711
GALC	-0.012078	0.2964844
TBL1X	0.0797384	0.2980596
KLHL11	0.0083488	0.2985804
CLDN15	0.0158102	0.2986138
USP15	0.0613812	0.2987201
UBL4A	-0.0179127	0.2988351
NCLN	0.0233876	0.2991574
IL17RA	-0.0464657	0.2991816
SLC25A15	-0.0352969	0.299618
CDAN1	-0.0052455	0.3001983
SMAD1	0.0221404	0.3012218
FAM199X	-0.0449217	0.3012508
SNX5	0.0019045	0.3021776
MICU3	0.0331258	0.3026995
PXN	0.008694	0.3027719
ZNF764	0.0233868	0.3028463
NKIRAS1	0.0523114	0.3029406
RWDD3	0.022943	0.3029536
PHF7	-0.0138577	0.303996
SPTAN1	0.0337082	0.3049231
NPC2	-0.1404356	0.3055327
TTC31	0.0482862	0.3064302
GMPR2	0.055683	0.3068611
LYAR	0.1051732	0.3088518
WDR5B	-0.0008696	0.3089312
KAT14	-0.0073466	0.3090201
PPM1L	-0.0223003	0.3103942
DNAJC12	0.0476077	0.3106153
TMEM44	0.0098842	0.3116067
CENPF	0.0719145	0.3116971
GVQW3	-0.0163995	0.3128061

RBMS1	0.0085675	0.3131758
AC078846.1	-0.0276437	0.3135554
TNNT3	0.0309281	0.3140032
NUMA1	-0.0456584	0.3140811
MCRIP1	-0.0371603	0.314217
TM7SF3	0.0087444	0.3143397
AL158152.1	-0.0622644	0.3145023
AP002360.1	0.0212713	0.3146914
AC025171.1	-0.018082	0.3151553
SBNO1	-0.0242059	0.3156813
OAS2	0.0683639	0.3159399
THEM4	-0.0350269	0.3160868
WDFY2	0.049959	0.3167004
ARMC5	-0.0203887	0.317237
TXNDC12	-0.0398904	0.3176215
AGPAT4	-0.0233129	0.3180366
MVP	0.0063158	0.319421
GNPDA1	0.1050443	0.3196682
NONO	-0.0154103	0.3198779
CD109	0.1293135	0.3199988
NXT2	-0.0071448	0.3203954
GSTM5	0.0302975	0.3217787
HEBP2	0.1205841	0.3220045
DNAJB9	-0.001675	0.3227934
TRANK1	0.0231123	0.3228906
SPATA33	0.0184135	0.3234062
ARHGAP15	0.0533928	0.3238695
AC026124.1	-0.0293531	0.323967
TTC7B	-0.0138265	0.3240234
ISCA1	0.0400521	0.3243092
CLN3	-0.0224619	0.3266618
TYW1	-0.0345836	0.3276742
CCDC12	0.0638657	0.3277643
FTO	-0.0200804	0.3277663
AL080317.1	-0.028194	0.3281299
VCPIP1	-0.004693	0.3290815
SMAD2	-0.1086068	0.3291111
UBOX5	-0.014072	0.3331206
C1orf43	-0.015323	0.3336496
TIMM23B	-0.0236223	0.3343168
TTI1	0.0053725	0.3345348
GPATCH2L	-0.1131389	0.3347757
CLPTM1	0.0240951	0.334992
PPP1CB	-0.1511245	0.3349941
HIST1H2AD	-0.0341946	0.3353919
PPIG	0.0971861	0.335412
WDR33	-0.0602804	0.3358382
ZSCAN30	0.0190888	0.3367349
PPIF	-0.1189958	0.3371788
ZNF33A	-0.1109288	0.3374255

SEC24B-AS:	-0.0204475	0.3382599
AP2M1	0.0424596	0.3385131
AC016588.2	-0.0271349	0.3386317
KMT2D	-0.0504945	0.3393778
YWHAE	-0.0912325	0.3394014
AC010642.2	0.01446	0.3407364
FAM118B	-0.0005387	0.3410533
STT3B	0.0091157	0.3415932
RDX	-0.0315342	0.3418051
SNHG26	-0.0461487	0.3430336
SLC35A5	-0.002993	0.3435474
NAB2	0.013191	0.3447469
ATOX1	0.0500442	0.3453232
BRD2	-0.0637983	0.3458251
DCP2	-0.0332444	0.346365
ZNF611	-0.0131659	0.3464177
HOXB4	-0.0103118	0.3464802
ZFAND2B	0.0358627	0.3466187
CSNK1G1	0.0184768	0.3472452
SNRPG	0.0322114	0.3473039
FADS1	-0.0013851	0.3500782
ALPK1	-0.0060487	0.3524655
UNC119B	0.0091441	0.3531624
NBPF26	-0.0239858	0.3531811
AFF4	-0.0407054	0.3534931
MNDA	-0.0318182	0.3539743
TESPA1	-0.1150091	0.3548168
CAPRIN1	-0.0347711	0.3549049
PDE3B	-0.0413625	0.3570955
PTTG1	0.0358373	0.358313
METTL18	-0.0116992	0.3583531
MESD	0.073929	0.3601823
ARFGAP1	-0.0040333	0.3612984
BLOC1S4	0.0322374	0.3614267
USP12	-0.0606773	0.3619566
METAP2	-0.0714022	0.3625377
GPATCH1	-0.0064607	0.3645582
DANCR	-0.0828792	0.3651177
KBTBD3	0.0050914	0.365875
MBD5	-0.0099749	0.3676475
ARID4B	-0.0814216	0.3686038
TIMP2	0.0333436	0.368674
HELQ	0.0056069	0.368928
WIPI2	0.0321965	0.3696107
AC083799.1	0.0080398	0.369914
GRK3	-0.0182771	0.3699958
DIPK2A	-0.0004738	0.3714563
TPSB2	-0.0380678	0.3715979
AP002387.2	-0.0189725	0.3721797
ANKRD36C	-0.097433	0.3732797

PTER	-0.0257888	0.3735235
TBXA2R	0.0568009	0.3756608
CTPS2	-0.0016492	0.3759189
IDH1	0.0317196	0.3779941
AC097634.1	-0.0284481	0.3782841
FRAT1	0.0362297	0.3799084
AC027020.2	-0.0156686	0.3805987
ZNF761	-0.0232051	0.3819054
LY86	0.0517807	0.382175
GATAD1	-0.0437401	0.3825526
AQR	-0.0395413	0.382807
AASDHPPT	-0.0136118	0.3832704
LIMS1	0.025949	0.3840428
SINHCAF	-0.0063605	0.3849518
ZNF775	0.005193	0.3851932
RFC1	0.0729129	0.3864736
SNX12	0.0008426	0.3867722
CAPN7	-0.0511148	0.3869213
OXCT1	0.0441541	0.3871545
PTGR2	-0.0264554	0.3873328
PKD2	-0.0385462	0.3897739
LGALS8	-0.0224981	0.3899038
SERTAD3	0.006928	0.3899647
PPP1R2	-0.0062883	0.3905401
STARD9	-0.0563886	0.3912132
RAB27B	0.1919365	0.3939034
NMNAT3	-0.0008309	0.3939711
EVI2A	0.1127965	0.3940304
TMEM183A	-0.0088576	0.3949234
TTLL5	-0.0276305	0.3961696
AL121652.1	-0.0251857	0.3964811
OSBPL3	0.0689585	0.3965938
CXXC1	0.000242	0.3981466
PTPN7	0.0938321	0.3982239
MX2	0.0307751	0.398914
RPP30	0.0100864	0.4002993
RHOBTB3	0.0983753	0.4004871
MNT	0.0034761	0.4012367
HEXD-IT1	-0.0244195	0.4019373
CDCA4	0.0249214	0.4024892
MTFMT	-0.0083744	0.4035859
EMC1	-0.0313395	0.404231
DHX9	-0.0131679	0.4057674
AC242426.2	-0.0014538	0.4058881
ZEB1-AS1	0.0290002	0.4097124
SMIM10	0.0631413	0.4101582
FIG4	-0.0100956	0.4102686
ZNF430	-0.061819	0.4111091
AC005523.2	-0.0010135	0.4136584
SENP2	-0.0374125	0.4138028

CLNK	0.0727614	0.4141527
PHF11	-0.0673402	0.4141988
TMEM14B	0.0305975	0.4157646
CIAO3	-0.0080779	0.4161849
UBAP2	-0.015595	0.4164095
RSRC1	0.0286322	0.4183525
NR1D2	-0.0593406	0.4188064
SNHG1	0.0282334	0.4191954
RASSF7	-0.0100896	0.4195025
ZNF480	0.0078347	0.4224026
TESMIN	-0.0455466	0.4242057
FAM122C	0.0083901	0.4247213
WDPCP	-0.0065123	0.4270541
AC073896.3	-0.0230833	0.4276934
SMC6	-0.0472289	0.4277
CERS2	-0.0506906	0.4282315
CCAR1	-0.0083375	0.428554
ZCCHC7	-0.0306745	0.4325558
PPOX	0.0128903	0.4329732
PARP4	-0.0562795	0.4335879
CLEC11A	0.0792237	0.4338483
QTRT2	-0.0321717	0.4343077
SLC43A1	0.0120557	0.4344228
NCK1	-0.0044792	0.4354843
NOTCH2NL(	-0.0514886	0.4357205
DNAJC16	-0.0156473	0.4360134
DMAC2L	-0.0521198	0.4375034
RABEPK	0.0210885	0.4382106
MAGEF1	0.0477017	0.438325
NOP9	-0.0031023	0.4391301
LITAF	-0.049604	0.4401847
LSM14A	-0.0364824	0.4402879
ZMAT2	-0.0110629	0.4423463
SHISA5	0.0110882	0.4450463
UGGT2	-0.0195283	0.4463097
CDK5RAP2	0.0028386	0.4490446
ACTN1	-0.124295	0.4518972
SART3	-0.0073915	0.4520995
CASP8	0.04834	0.4531358
SYPL1	-0.0812656	0.4534407
FBXL17	-0.005278	0.454233
KIAA0319L	-0.0151393	0.4543519
CBX5	0.0295712	0.4551203
LIMD1	0.0066357	0.455524
ZNF692	0.0020319	0.4563601
SLC4A1AP	0.0021174	0.4564431
CFAP97	0.1286906	0.4575464
CRIP1	0.1611963	0.4577743
NBPF11	-0.0254441	0.4586315
SLC3A2	-0.0029029	0.4586582

BIVM	-0.0203162	0.4594252
MTRF1L	-0.0457364	0.4595964
RSAD2	-0.0036999	0.4608794
ELP2	-0.0198377	0.4615647
GPSM3	0.0682858	0.4622158
CCDC88A	-0.1265261	0.4638748
RASSF3	-0.0542273	0.4643973
EFCAB7	-0.0209942	0.4661937
CLCN6	-0.0163636	0.4675217
ZNF581	0.0400907	0.4682107
LINC00683	-0.0273409	0.4700868
ZP3	0.0056427	0.4705549
NOTCH2NL/	-0.0404355	0.4708804
WASF2	0.134345	0.4720681
TRAFD1	-0.0055672	0.4721279
GAS7	-0.0242356	0.4757528
TMEM267	-0.0416176	0.4757886
LINC00339	0.0231463	0.4764548
TMEM191B	-0.0216585	0.4768226
RCE1	-0.0174294	0.4794637
STX11	-0.0343251	0.4796069
DPY19L1	-0.0449073	0.4801092
N6AMT1	-0.0374068	0.4811797
MOGS	-0.0061869	0.481817
LRIF1	-0.0202007	0.4836484
SCAMP1	-0.0511115	0.4845506
STK40	-0.0186148	0.4845537
UCN	0.0028649	0.4855318
FANCL	-0.0153532	0.4872536
WDR55	-0.0063643	0.489449
INTS5	-0.0061425	0.4897279
SULT1A3	-0.0478586	0.4897631
DPP8	-0.0660508	0.4903224
KIAA0232	0.0280461	0.4915585
ZNF337-AS1	-0.015614	0.491679
MOSPD1	0.0033198	0.4921022
AC136944.2	-0.0305634	0.4922011
TMED5	-0.0372292	0.4936619
ZNF286A	0.0776223	0.4937351
PVR	0.0327431	0.4942169
HDLBP	0.0093614	0.4949315
ZBTB25	-0.0076855	0.4958675
DIS3L	-0.0193905	0.4959984
KATNB1	0.0500562	0.4967887
UBE2Q1	0.0653839	0.4974861
EMC8	0.0056858	0.4980466
EFCAB11	-0.0128388	0.4983206
CHRNB1	-0.0435975	0.4988825
ZC3HAV1	-0.0185308	0.4994447
ZNF717	-0.0093345	0.4996482

CHST12	-0.0211667	0.5000245
PTPN18	0.0223608	0.5025825
ZNF254	-0.0201176	0.5035806
AC146944.4	-0.0159262	0.503679
RRP8	0.0467711	0.5045303
PROK2	-0.0505225	0.5073344
GABPB2	-0.0314063	0.509293
RARA	-0.0008144	0.5102928
POLR3GL	0.0173522	0.5104584
KCTD6	0.0095097	0.5113734
MRRF	-0.0058672	0.5122862
AL031595.3	-0.0032709	0.5133379
LINC01943	0.0257417	0.5133846
SON	-0.1443359	0.5136874
EIF4G3	0.0168426	0.5137127
FBXO46	-0.0216886	0.5137397
AC021016.2	-0.0247698	0.5156543
CASP9	-0.0153206	0.515997
PPP3CB	-0.0204617	0.5166724
MYO5C	0.0772865	0.5187078
AL512625.3	0.0114723	0.5197746
THADA	-0.0236645	0.5204213
STRBP	-0.0463767	0.5215472
ADGRE1	-0.0343405	0.5216935
CHMP7	-0.0058163	0.5235904
ZNF765	-0.0194896	0.5240732
SLC39A3	-0.0245825	0.5256474
IFT122	-0.0206182	0.5260616
PARP11	-0.0314102	0.5270596
FAF1	-0.0102503	0.5273653
PHTF2	0.0043348	0.5282482
OGA	0.0032284	0.5288077
SAMD9L	0.0346319	0.5299257
TBRG1	-0.0670843	0.5303502
RHOC	0.0803107	0.5309504
ARFIP2	-0.0050184	0.5321032
G2E3	-0.0204062	0.5333287
TMEM159	0.0136454	0.5336014
TFIP11	-0.0018044	0.5336424
FARS2	0.0531564	0.5339146
FBF1	-0.0048237	0.5352703
ZNF528-AS1	-0.0705998	0.5352937
NR2C2AP	0.0284035	0.535912
PADI4	0.1390482	0.5360262
FAR1	0.0057135	0.5390529
GOLGA1	0.032074	0.5391251
WT1	0.0207635	0.5406305
ZWILCH	0.0034419	0.5408513
DENR	0.0279668	0.5412058
C21orf62-AS	-0.0356265	0.5423177

MORC2-AS1	0.0024489	0.5424038
UBE2Z	0.038396	0.5433137
NT5DC3	0.0032574	0.545076
LYPLA1	-0.0442474	0.5454155
PTPN9	-0.0328087	0.5471058
ZNF567	-0.0020566	0.5486945
EPB41L2	-0.0099364	0.5499951
ZNF197	-0.0027966	0.5503408
AGA	-9.902E-05	0.5509036
SOGA1	-0.0139883	0.5525598
ZNF528	-0.0335256	0.5529144
XKR8	0.0061842	0.5547083
CCNJ	-0.0373294	0.554857
UNC45A	0.0140571	0.5554817
GLCE	-0.0095833	0.5555262
CTC1	-0.0100934	0.5558879
DOT1L	-0.0061564	0.5563143
AKAP10	-0.0568529	0.5564243
CLEC16A	-0.0177281	0.5568661
PUS3	-0.0383303	0.5570546
AC025171.2	-0.0697861	0.5585323
AL353194.1	0.0273855	0.5610669
FAM160A2	-0.0279974	0.5615279
ZNF724	0.0018598	0.5616823
HARS2	-0.0211462	0.5621112
ZNF667	0.0179185	0.562772
POLG	-0.0208604	0.5632975
CHMP4A	0.0056293	0.565901
TMEM116	-0.0177455	0.5662528
CUL2	-0.0335821	0.5679297
FBXW9	0.0608652	0.5681655
CSE1L	0.0266708	0.5709312
HSD17B1	-0.0053549	0.5713285
ZNF248	-0.0229332	0.5716184
ANO10	0.0031475	0.5743568
HBS1L	-0.0093566	0.5748687
SLK	0.0405058	0.5767241
TNFAIP8L2	0.0455699	0.5769915
DSTN	-0.0137372	0.5775256
FAM13A-AS	-0.0353058	0.5782154
WDR27	0.0315713	0.580239
MAP1S	-0.0634395	0.5804417
LONP1	0.0182054	0.5818568
CXorf40B	-0.0078242	0.5830173
CRACR2A	-0.0123312	0.584093
ZNF138	-0.0274016	0.5885035
MMS19	0.009464	0.5899964
LRP5	-0.0021312	0.5916365
PGM1	0.0171035	0.5941762
MOAP1	-0.0052579	0.5952871

HCG18	-0.1029943	0.5971361
TNFAIP2	-0.0472063	0.5988688
SGMS1	0.0453466	0.5992844
TRUB1	0.0055715	0.5993034
SMARCA5	0.07196	0.6015235
TRIM52	-0.0008128	0.6017657
AC005838.2	-0.0048919	0.6022577
TACO1	0.0432071	0.6042568
MTERF4	-0.0211334	0.604455
ATG2B	-0.0314147	0.6059898
TIFA	-0.02788	0.6068541
SCFD1	-0.0073573	0.6071248
ING5	-0.0022865	0.6089289
ZDHHC8	0.0100771	0.6108417
MCTP1	0.0337964	0.6134734
MIER3	0.0044109	0.6142769
CLIC4	0.034738	0.6152743
DNASE1L1	-0.011023	0.6154393
EXOSC1	0.0119962	0.6155604
SUSD3	0.0680761	0.6160937
NF1	-0.0420284	0.6174711
CCT6B	-0.0147781	0.617735
ARF1	0.0808513	0.6194929
ATP5ME	-0.1111152	0.6208871
DDX60	0.0058705	0.6214594
ZDHHC13	-0.039596	0.623081
NAIP	-0.0904427	0.6247721
NBR1	-0.0370826	0.6247975
INPP5A	-0.0137923	0.625369
BEX4	0.0466171	0.6257139
ISY1	-0.0140279	0.6288503
AC008393.1	-0.0149533	0.6300328
PVT1	0.0576475	0.6312053
PFAS	0.0275765	0.6317447
ZNF512B	0.0240632	0.6330628
GNB2	0.0439982	0.6331147
NIPSNAP1	0.0086653	0.6332246
A1BG-AS1	0.0190766	0.6348831
AC159540.2	0.0204273	0.6351622
C7orf31	-0.0142409	0.6351657
ZUP1	-0.0047812	0.6359639
TESK2	-0.0374552	0.6362095
DNAJC21	-0.0088993	0.6367019
TRIP6	0.0467391	0.6372917
PPIP5K2	-0.0425169	0.6375212
MAP3K13	-0.0249741	0.6393126
GABPB1	-0.025797	0.6399697
MMADHC	-0.0180548	0.6406245
SLC25A51	0.0147796	0.6409022
BATF	0.0024371	0.6417677

SUFU	-0.007226	0.6438354
ATP13A1	0.0328739	0.6446386
AAR2	0.0156413	0.6471961
SLC26A2	-0.144132	0.6480741
NOP58	0.075801	0.6486934
KYNU	-0.0055548	0.6497858
ZNF445	0.0007951	0.649974
ENPP4	0.0066754	0.6517788
C20orf197	0.0783514	0.652219
AC008875.3	0.0187131	0.6537732
WDR47	-0.0041388	0.6545885
ERLEC1	0.0133256	0.6550805
TMEM67	0.014314	0.6553505
KLHL36	-0.0376023	0.6563947
HUS1	-0.0358893	0.6602596
MFNG	-0.0057723	0.6608376
UHRF1BP1L	-0.0513816	0.6649299
RACGAP1	-0.0110131	0.6652267
SPTY2D1	0.0445563	0.6656131
ZNF107	-0.0447862	0.6664178
GMEB1	-0.0407207	0.6665874
NATD1	-0.0027279	0.6679523
SNAP23	-0.0463417	0.6689536
CAPZA1	-0.0849886	0.6690252
ST6GALNAC	-0.0012378	0.6729944
MFHAS1	0.072005	0.6730946
MLC1	-0.0387255	0.6743391
NEDD9	0.0563886	0.6750326
HAUS6	0.0180409	0.676632
ZNF282	0.0289257	0.6766712
CCNG1	-0.0805185	0.6767665
PYGB	-0.009478	0.6769796
CRY2	-0.0180142	0.6784166
BISPR	0.0102763	0.6799042
PTPN12	-0.0497859	0.6799078
NR6A1	0.0109129	0.6809712
CDC42-IT1	-0.024127	0.6824281
PHOSPHO2	0.0076152	0.6842497
AC024075.2	-0.0252987	0.6880247
UTRN	0.0513067	0.6913465
ABCC5	-0.0243543	0.6918247
CAMLG	-0.1071165	0.6934691
JUP	0.0708463	0.6941482
SEL1L	-0.0334942	0.6943422
AC120053.1	-0.0497909	0.6947543
PLEKHB2	-0.028955	0.6968469
PSME4	-0.0492828	0.6972064
ANKRD17	-0.0183351	0.697247
AK2	-0.1467579	0.6975576
SOCS4	0.0344541	0.6981568

B3GNT8	-0.0177982	0.698282
UBXN8	0.0180867	0.701327
EIF3J-DT	-0.0234522	0.7041272
ZC3H6	-0.0138185	0.7048053
AC007878.1	-0.0058141	0.7053858
CNBD2	-0.0108924	0.7055223
AC078883.1	0.0236815	0.7056786
ZNF865	-0.0471325	0.7090999
NDUFB9	-0.0415686	0.711448
SAMD10	0.0322691	0.7142124
ZNF280D	-0.0071053	0.7149125
TYW1B	-0.0449554	0.7152221
CD63	0.1709855	0.7168632
ACSL4	-0.0109931	0.7178147
ESCO1	0.0023864	0.7182226
TRAK1	-0.011602	0.7226561
ASPH	-0.022024	0.723377
TRIM59	0.0071133	0.7265349
TMEM135	0.028688	0.7287244
CLN6	0.0242136	0.7296543
USP30-AS1	0.0017614	0.7314675
FDFT1	-0.0795393	0.7315243
AL138724.1	-0.000264	0.7319252
TMEM9	0.0048466	0.731929
MIR1915HG	0.0321908	0.732396
AC009118.3	-0.0119844	0.7330408
ZFP28	-0.0066747	0.7334967
GBE1	0.0144188	0.7345809
ZNF174	0.0097388	0.7358493
CMSS1	0.0709199	0.7363535
PRPS1	-0.0090352	0.7365682
SECISBP2	-0.0801671	0.7368386
AL391832.2	0.0171144	0.7370393
CDK5RAP1	-0.0126712	0.7375006
HNRNPA3	-0.004121	0.7388553
TRAM2	-0.0035932	0.7401589
AARS	0.0170994	0.7416385
HDHD5	0.0200622	0.7416842
TOB1	-0.0302917	0.74351
RGPD2	-0.0171589	0.7453141
KDM5A	-0.036481	0.7485415
ZBTB48	-0.0060476	0.7487873
OAT	-0.024336	0.7500163
CHEK2	0.0127759	0.7511306
KLHL7	0.0107112	0.7535965
RASGRP4	0.0574816	0.7540983
ZNF879	-0.0137803	0.7562944
POGK	-0.0175584	0.7592183
ZNF713	-0.0435666	0.7599032
ZNF845	0.0056741	0.7603026

SUPT4H1	-0.0132441	0.760487
AHCYL2	-0.0292364	0.7614014
YOD1	-0.047886	0.7622866
GALNT7	-0.0191593	0.762483
ADD3	0.0066483	0.7632862
MAML3	0.0480509	0.7638869
RIPK2	0.0054843	0.7657137
RHBDF2	0.0172846	0.7683265
SORBS3	0.056574	0.7725172
ERAL1	-0.018861	0.7737976
RAF1	-0.0715362	0.7746755
TYSND1	0.0202866	0.7756739
TRIM25	-0.0168132	0.7756954
SARS2	0.0325533	0.7764564
IGBP1	0.0014137	0.7792964
ACAT1	-0.0051102	0.7864199
HNRNPK	-0.1291092	0.7874177
C22orf34	-0.0173106	0.7896531
TMEM214	-0.0174341	0.792758
UFL1	0.0078879	0.793222
FLAD1	-0.0228069	0.793914
FOXJ2	-0.0048158	0.7946293
CHMP2B	0.0297364	0.7953238
AL133243.2	-0.0009501	0.7975556
PPA1	0.1398142	0.7986804
MAPK8	-0.0376501	0.7988974
IMMT	0.0547835	0.799896
FBXO33	-0.0120049	0.8015671
FAM53C	-0.0101343	0.8039068
ABHD16A	0.0255162	0.8041545
CSAD	-0.0087731	0.8045004
TRERF1	0.0524867	0.8068872
TAGLN	0.0699948	0.8091277
KANSL3	-0.02198	0.8124152
TSNAX	-0.0679587	0.8132695
ZNF778	-0.0118295	0.8161422
NEDD8	0.1068926	0.8168801
BLOC1S3	0.0194278	0.8182327
HOXA5	0.0215007	0.819107
ZNF549	-0.0219953	0.8199508
HERPUD2	-0.0367124	0.8208394
MIPEP	-0.0170215	0.8221803
IRF9	0.032642	0.8248552
SMC5	0.009324	0.8260361
AC009005.1	-0.0397625	0.8280611
AARS2	-0.0040506	0.8286033
GABPB1-IT1	-0.0063042	0.829233
KPTN	0.0687437	0.8295974
TATDN1	0.0083228	0.8298461
CPSF1	-0.0329406	0.8309409

PAIP2	-0.0261175	0.833109
INTS6L	0.012283	0.8334709
GINM1	-0.0023231	0.8338766
ZXDB	-0.0042464	0.8357607
MED28	-0.0643457	0.8374357
COG8	-0.0361181	0.8408327
VOPP1	-0.0271452	0.8425523
MED17	0.0112504	0.8436523
AP1G1	-0.0270342	0.8449488
MTHFR	-0.0303665	0.8470624
DDI2	0.0007335	0.8498389
STK3	0.0051541	0.8500934
AMZ2	-0.0069261	0.8502104
TAPT1-AS1	-3.354E-05	0.8513717
TECPR2	-0.0046408	0.8526359
H1FX	0.2749199	0.8528826
QSER1	-0.0218814	0.8529582
ADAM10	-0.0453812	0.8547512
SH3D21	0.0566135	0.8574374
SLC30A1	-0.0209744	0.8603645
REXO5	-0.0062098	0.8622969
POLE4	0.0676466	0.8624015
ZNF646	-0.0041369	0.8625607
HIC2	0.0430532	0.8629703
OGDH	0.0197452	0.8635748
APH1B	0.0158389	0.8660546
ECHS1	0.0636351	0.8663703
AC016831.5	0.0660718	0.8670715
CENPT	0.0030644	0.8673172
ZBTB18	-0.0286353	0.8674596
HSPA14.1	-0.0313965	0.8696552
PPM1D	-0.0083116	0.8742067
SERF1A	-0.0170194	0.8745542
ELOA	0.0036884	0.8762605
ELF4	-0.0406641	0.8857519
CD2AP	0.0715383	0.8865228
AL355001.2	5.64E-05	0.8867377
UBE2B	0.0455101	0.8873154
ZSCAN5A	0.0329479	0.8923019
TBC1D16	-0.0428128	0.8926465
TMEM25	0.0545511	0.8934878
IPO13	0.0074176	0.8948818
APPL2	0.0060242	0.8970777
AC022973.5	-0.0136094	0.8995265
DESI2	0.0458992	0.899968
POMP	-0.0352275	0.9006373
CEP41	-0.0288572	0.9014048
CHCHD3	0.0406126	0.9014549
ZPR1	0.0117124	0.9021155
ARHGAP17	0.0161678	0.9064561

SLC19A1	0.0325737	0.9076084
ABRAXAS1	-0.0230139	0.9118925
MEST	0.0177879	0.91393
PRDX2	0.1811329	0.914193
PPP1R26	-0.0112749	0.9159705
ACSS2	-0.0038963	0.9174385
NOL8	-0.0327237	0.9189702
UTP4	-0.0177908	0.9203449
NEK3	0.0115225	0.9205872
ARAP1	-0.0199977	0.920806
EIF3M	-0.0757326	0.9212041
SLC9B2	0.0291489	0.9212835
MIR762HG	-0.0232461	0.9222158
ZNF141	-0.0501497	0.9250881
BRAF	-0.1054884	0.9262294
ANAPC2	-0.0370085	0.9267839
AC108134.3	0.0213149	0.9295742
FCHO2	-0.0009709	0.9334432
MPO	0.4011183	0.9373652
C8orf82	0.0105777	0.9374054
GXYLT1	-0.015959	0.938821
CYB561D1	-0.0187501	0.9409579
TLR2	-0.0466633	0.9431799
LINC00893	0.0156194	0.9447912
URI1	0.0460715	0.9476792
TIE1	0.0221252	0.9490204
CCDC71	-0.027965	0.9491636
AL133243.3	-0.0195399	0.9494525
EEF1D	0.1312771	0.9494793
ZNF385A	0.0317941	0.9508697
ATP6AP2	-0.0283274	0.9517297
CGRRF1	0.0189661	0.9519689
AL096865.1	0.0038746	0.952494
MTDH	0.1790687	0.9534151
C17orf58	0.0267014	0.9539656
LARS2	0.0023895	0.9541308
CRELD2	0.0222481	0.9567824
GTF2H5	0.0469746	0.9596058
IPMK	-0.0428861	0.961436
AC021097.2	-0.0119703	0.9659884
KCNK5	0.0198648	0.9672468
MTRR	-0.0136435	0.9697508
ALDH9A1	-0.001169	0.9706573
CASP8AP2	0.0433103	0.9716301
GGT5	0.04479	0.9721095
SPIRE1	0.0378027	0.9725399
PRMT7	0.0386898	0.9755302
SLC39A4	0.0404198	0.9764266
PIGZ	-0.0139448	0.9813766
ANXA11	0.0041243	0.9815407

LIMA1	0.0711687	0.981865
ZNF747	-0.0044963	0.9867515
MTFP1	0.0147975	0.9905214
ABAT	0.0091604	0.9929246
IFT80	0.0233544	0.9932996
UBE2V1	0.0400971	0.994887
VRK2	0.0070369	0.9962308
ODF2L	-0.0371391	0.998289
COPS8	-0.0221109	0.9989555
APPBP2	0.0485767	0.9992416
ZCCHC14	0.0005722	0.9998119
MED31	0.0031673	1
RETSAT	-0.0088158	1
MTG1	0.0202452	1
SLC16A7	0.0499864	1
PPCS	-0.0474045	1
ZNF552	-0.0159793	1
RWDD2B	-0.0065537	1
RAP2C-AS1	-0.0124662	1
CHMP5	-0.0496034	1
UTP3	0.0511854	1
TCP11L2	-0.011173	1
TCEAL4	0.1042863	1
C6orf226	-0.0169448	1
ATXN7L3	-0.0442742	1
RFTN1	0.0265306	1
PJA1	-0.0027308	1
ERC1	-0.0086281	1
PROM1	0.1459467	1
TOB2	0.0242208	1
AF117829.1	-0.0048861	1
BTG3	0.002698	1
SNX3	-0.0906144	1
PLCE1	-0.0143738	1
PDPR	-0.0112361	1
ZBED5-AS1	0.0002813	1
CDIP1	0.0078187	1
KIAA1841	-0.0081596	1
CAPN2	0.0454777	1
NENF	0.100696	1
TTF1	-0.0105157	1
FRA10AC1	0.0198335	1
ZC3H10	-0.0208359	1
FAM135A	-0.0252716	1
SLC25A35	-0.0100923	1
MED7	0.0227503	1
AC114980.1	-0.0041235	1
ARL14EP	0.0130525	1
CROT	-0.0397174	1
NAALADL1	0.0273892	1

TUT1	0.0072183	1
ACTR2	-0.1107905	1
AL391684.1	-0.036213	1
OXR1	0.016553	1
NDC1	0.0118003	1
GNPDA2	-0.023345	1
EML2	0.0026663	1
SPTBN1	0.0474994	1
CAD	0.013147	1
RSF1	0.0434232	1
TMEM131	-0.1087435	1
HDDC2	-0.0117093	1
N4BP2	-0.0383351	1
RAD21	-0.0122059	1
MMAB	0.0288305	1
AC104532.2	0.0142278	1
ZNF681	-0.0294091	1
WARS2	0.0180432	1
DCAF10	-0.0249887	1
CMPK2	-0.0489935	1
P2RX7	-0.0187824	1
RPTOR	-0.0071549	1
CARNMT1	-0.0487114	1
S100B	0.148342	1
PIP4K2C	-0.0293848	1
UBE2D1	-0.0373066	1
SCRIB	-0.0008999	1
KIF9-AS1	0.0220376	1
PLIN2	-0.1274322	1
WDR89	0.0295794	1
CD302	0.1043704	1
RASSF1	0.0529416	1
CHCHD10	0.0401577	1
TOE1	-0.0099582	1
ATP6AP1L	-0.0139842	1
CHCHD5	0.0234922	1
TSN	-0.0482892	1
ITPRID2	-0.068951	1
SARM1	0.0166619	1
C16orf72	0.0228458	1
MINDY1	0.0047689	1
PHGDH	0.0975785	1
RAD50	0.092377	1
EID1	0.0662223	1
AOPEP	0.0149478	1
RAB27A	-0.1182897	1
SSR3	0.0155667	1
DESI1	0.0639329	1
C12orf43	0.0143101	1
CCDC58	-0.018379	1

CYTIP	0.0977523	1
PHF20	0.0452822	1
SAV1	-0.0163529	1
FEM1C	0.00025	1
ORMDL3	0.0193794	1
MTIF2	0.0074471	1
USP46	-0.0055002	1
AFG1L	-0.0138947	1
YWHAG	-0.1182718	1
PTPN2	-0.0441397	1
KATNAL1	-0.0013155	1
OXSR1	0.0061614	1
ZNF398	-0.0009879	1
TEX22	0.0291252	1
OTULIN	-0.0250012	1
BLM	0.0181468	1
ZNF558	-0.0042453	1
TBK1	-0.037403	1
GYS1	-0.033483	1
AL365272.1	0.0069083	1
ZNF8	-0.0338075	1
VPS29	-0.0001557	1
TYMP	-0.0419474	1
AP2A2	-0.0149023	1
TNRC6A	-0.0387188	1
VENTX	0.0316376	1
NVL	-0.0170931	1
CCDC88B	-0.055299	1
FTSJ1	0.0054817	1
DNAJC27	0.0074403	1
SRSF7	0.1353227	1
ACBD4	0.0318713	1
DBT	-0.0632201	1
MTCH2	0.0231006	1
CPQ	-0.0064892	1
EIF3C	-0.0356686	1
TAF1B	0.0359006	1
MYO9B	-0.0330304	1
NFE2L3	-0.0764244	1
MARCH7	0.0053706	1
ARMC6	0.0271781	1
TFAP4	-0.0074902	1
TMTC3	-0.007129	1
PSTPIP2	0.0226121	1
POLL	-0.0200555	1
ING1	-0.0031324	1
HACD2	-0.0179024	1
STX17	-0.0487698	1
DIS3	-0.0030647	1
CPT2	0.0065512	1

CIPC	0.0006374	1
TIGD2	0.0104465	1
AC007066.2	-0.0246994	1
FBXW4	-0.0032329	1
TIMM9	0.0373377	1
FBXO41	0.0059727	1
UBE3B	-0.0389747	1
CLP1	-0.0159978	1
ATXN7	0.0418377	1
MTAP	0.0299931	1
LINC01002	-0.0234418	1
HOMEZ	-0.0099293	1
DBR1	0.0019763	1
PFKL	0.0127263	1
NUDT5	0.0471178	1
EIF3D	0.0937087	1
PRPF4	-0.0027295	1
TGDS	0.0024184	1
PSMD7	0.0399739	1
RTN3	-0.1166619	1
AC009506.1	0.037589	1
MIDN	-0.0024057	1
MAP3K3	0.0213833	1
CCRL2	0.0229653	1
MANEA	0.0213443	1
ZBTB47	0.0065689	1
TAX1BP3	-0.0290347	1
ZNF519	-0.0119314	1
COG7	0.0445255	1
CD83	-0.0121837	1
SHPK	-0.0026331	1
ATP6V1E2	0.0127017	1
MPP6	0.0692785	1
AUTS2	0.0724959	1
RAB38	0.0742433	1
MED14	-0.0564356	1
ING2	-0.0114382	1
RABL3	0.0115163	1
DHX57	-0.002058	1
BZW2	0.1200339	1
SLC35D2	-0.0195186	1
TPMT	-0.0187389	1
FANCB	0.01009	1
VPS50	-0.0324697	1
ZNF354C	0.0091068	1
TAF1A	-0.006004	1
TMEM138	-0.0368684	1
PSPH	-0.0159072	1
RASA2	-0.0115329	1
USP14	-0.0103069	1

LINC01006	0.0026289	1
PLAUR	0.0026833	1
TMEM241	-0.0057938	1
OLA1	0.001133	1
AL356599.1	-0.0127393	1
MTLN	0.0445768	1
GLA	-0.0022505	1
TIMM10B	-0.0078218	1
BCL2A1	0.0429421	1
C19orf53	0.1475728	1
ERBIN	-0.0229948	1
CCDC43	-0.0001614	1
LMAN2L	-0.0180228	1
PHF20L1	-0.0875014	1
PSMB3	0.0047879	1
FBXL13	-0.0085307	1
CCDC69	-0.0495665	1
MYD88	0.0172413	1
IFIT5	0.0245607	1
ZBTB10	0.0125464	1
SERTAD1	0.0442757	1
LRRC42	-0.0009076	1
SLC12A7	0.0599928	1
ELAC1	0.0062533	1
FAM133B	-0.0118816	1
IMP3	-0.0358163	1
DAGLB	-0.0317625	1
TYMSOS	0.0071924	1
SESTD1	-0.0410056	1
TRIM58	-0.0698197	1
BMP1	-0.0116962	1
CXXC5	-0.1278321	1
PEX26	-0.0644871	1
PTGER4	0.0283571	1
SSB	0.119725	1
PSMB4	-0.0029041	1
KCMF1	-0.0551321	1
PNMA1	-0.0104579	1
RAPGEF2	0.0759452	1
FCHO1	-0.0112783	1
GCC1	0.0137708	1
UBA6-AS1	-0.0285992	1
LRRC37A2	-0.0302766	1
DAPK3	-0.0176389	1
RALGPS2	-0.0276138	1
AC106791.1	-0.0246907	1
H2AFJ	0.0696347	1
ZSCAN31	-0.0026476	1
MIR1-1HG-A	-0.0155264	1
RPS11	-0.0733529	1

FNTB	-0.0343994	1
C1RL-AS1	0.0241365	1
CPEB4	-0.0392558	1
PIAS4	0.009292	1
SH2B1	0.0089457	1
NICN1	-0.0033415	1
PARVG	-0.0274338	1
CARD9	-0.0157485	1
AC096711.2	-0.0277427	1
CTIF	-0.0177227	1
ZNF358	0.0231047	1
LINC00476	-0.027079	1
GLCCI1	-0.0065198	1
PMM2	0.0111068	1
XIST	-0.1463977	1
ZNF426	-0.0026729	1
RBM41	-0.0534333	1
AP4B1	-0.0018358	1
HS1BP3	0.0139135	1
OSBPL7	-0.0071081	1
WDR44	-0.0044138	1
E2F3-IT1	-0.0132259	1
UPF3B	-0.0497038	1
AC011462.5	-0.0219735	1
PHF21A	-0.0254538	1
ZNF90	-0.0201605	1
XRN2	0.0600915	1
ZNF461	-0.003746	1
CPB2-AS1	-0.0193096	1
MEF2A	-0.0275343	1
CLOCK	-0.0203895	1
TSTD3	-0.0118516	1
TMEM243	-0.0016223	1
SLC5A6	0.0163902	1
AL109628.2	-0.0334417	1
TSPAN3	0.0141245	1
AC010623.1	-0.0053583	1
SUCLA2	0.0146266	1
CRISPLD1	0.0138486	1
TRPM7	-0.0923598	1
MMP17	-0.0104081	1
LZTR1	-0.0101751	1
ZNF782	-0.0213556	1
SMG9	0.013122	1
ZNF324	0.0140198	1
ABCC1	-0.0653937	1
ARRDC3	-0.1149433	1
NR4A2	0.0522223	1
ZNF718	-0.0543878	1
AC093323.1	-0.0279271	1

FLVCR1-DT	-0.033135	1
ZNF625	-0.0058343	1
RANBP3	-0.0124957	1
MAU2	-0.0538301	1
BBS10	0.0027152	1
DUS3L	0.0292227	1
ANKLE1	0.0105929	1
CSTF2T	-0.0262887	1
RUSC1	0.0007708	1
SNX32	-0.0152989	1
CKMT2-AS1	-0.0058326	1
SPART-AS1	-0.0178372	1
PPCDC	0.0337486	1
ZNF527	-0.008716	1
GATD1	0.0125347	1
PRCC	0.0103544	1
ZBTB11-AS1	0.0005688	1
ZFP90	0.0207688	1
RNF103	-0.0040394	1
RBM28	0.019429	1
PPP6R1	0.0244321	1
C5orf30	0.0217217	1
ATPSCKMT	-0.0163834	1
GRINA	0.0137847	1
RPUSD2	-0.007462	1
SRGAP2	0.0302921	1
BNIP1	0.0013727	1
LYRM4	0.0478653	1
DHX29	-0.0078192	1
PPP3CC	-0.007577	1
ADAT1	0.001367	1
USP6NL	0.0317357	1
COL4A5	0.0260491	1
AC110285.2	-0.0312889	1
ZRANB3	-0.0047818	1
GOT1	0.0073386	1
GFOD2	0.0177762	1
OAF	0.0901817	1
TDP1	-0.0170112	1
ACAD9	0.0124701	1
CNOT7	-0.0462874	1
MYO7B	-0.0116873	1
AP4E1	-0.0275934	1
KMT5A	0.0041781	1
DZIP3	0.0095618	1
PEX1	-0.0045531	1
CD47	0.1034751	1
XPO1	0.0354082	1
ERCC6L2	-0.0469981	1
SKAP2	0.0107691	1

PCYOX1	-0.0086314	1
SLC17A5	-0.0447065	1
MLKL	0.0880788	1
RPS2	0.100219	1
SAYSD1	0.0078877	1
KCNC4	-0.0185195	1
ERN1	-0.0328909	1
ZNF570	0.0134633	1
GPR160	-0.0071605	1
PPARA	0.0090832	1
CHD2	-0.0468968	1
ZNF136	0.0064986	1
NUCKS1	0.1648029	1
ELF1	0.119707	1
PAXIP1	-0.0068357	1
TPR	0.0588561	1
CCDC9	-0.001489	1
NT5M	0.0179337	1
ZC3H15	0.0225501	1
CCDC62	-0.0117948	1
UFSP2	0.0013308	1
SPINDOC	0.0046025	1
ZSCAN26	0.0136574	1
ABR	-0.017943	1
MECP2	-0.0509309	1
AC015813.1	-0.0395328	1
UGDH	-0.0202446	1
ZNF512	-0.0349294	1
SLC23A2	-0.0100745	1
HIST1H1C	0.0759308	1
CD300A	0.0413981	1
AC006141.1	-0.049087	1
GTPBP3	0.0078763	1
KCTD9	0.0337618	1
ZNF518A	-0.0189105	1
CACYBP	-0.1453491	1
HHEX	0.0598529	1
SLC25A40	-0.0358102	1
PDIK1L	-0.0120204	1
SPG11	-0.049259	1
TRAF5	0.0380619	1
AL353804.1	-0.0168892	1
PIGO	-0.0015788	1
CCDC14	-0.0716837	1
CLIC1	0.1566752	1
USP48	0.0267394	1
ZSCAN21	-0.0003706	1
FSBP	-0.0143142	1
PIGBOS1	-0.0125754	1
NRDE2	-0.0505315	1

AC027097.1	-0.0334532	1
FYB1	0.0241868	1
FAN1	-0.0074531	1
MRPL49	-0.0190014	1
RRN3	-0.0191314	1
ZNF678	-0.0065452	1
GBP4	-0.1202766	1
TRIP11	-0.0304765	1
CERS6	-0.0102355	1
AC058791.1	0.0306227	1
CHEK1	0.0787344	1
TRIB3	0.0289753	1
PBX3	0.0039646	1
ALS2	-0.0913669	1
TTC39C	0.0202349	1
AP1AR	-0.0271867	1
OLIG1	0.0415209	1
SEC62	-0.0970679	1
EIF1B-AS1	-0.0157908	1
NOMO3	-0.0273378	1
AC068205.2	-0.0302666	1
HVCN1	-0.0062951	1
APLP2	-0.1716966	1
C12orf45	-0.0134934	1
ARL2BP	0.0260896	1
IRF3	0.0107743	1
TAF10	0.0226401	1
NFE2	-0.1462465	1
AC018521.5	0.0454745	1
MTG2	-0.0090988	1
AC019080.1	-0.0099328	1
ZNF283	-0.0044047	1
NEK1	0.0134866	1
FAM114A2	0.0317558	1
RGS3	0.0098541	1
SENCR	0.0645078	1
C12orf49	-0.026851	1
PCGF5	-0.0409755	1
PRAM1	0.0515247	1
ZNF71	-0.002127	1
BCL2L11	-0.0264099	1
WAS	0.0329861	1
FKBP15	0.0312111	1
ETV5	-0.027076	1
AMACR	0.0069391	1
TXNL4B	0.0033012	1
PKP4	0.0648039	1
XPO5	-0.0146643	1
AL162595.1	-0.0093132	1
RDH5	-0.0035585	1

JADE1	-0.0146195	1
ETNK1	-0.0773064	1
TMEM65	0.0008254	1
CARD8	-0.0263938	1
ZNF620	-0.0004578	1
EXOSC4	-0.0138981	1
PPP2R3B	0.039426	1
DHRS1	-0.0077424	1
ATG4A	0.0088209	1
RNASEH1	-0.0038978	1
LGALS3	-0.059338	1
FUNDC1	0.0476415	1
HSD17B11	-0.088475	1
PILRB	-0.0094253	1
EEF1AKMT3	-0.010124	1
DLAT	-0.0080494	1
ZNF275	0.0166256	1
ZNF525	0.0157524	1
AL157938.3	-0.0218883	1
ZNF844	-0.0388705	1
ZNF546	-0.0067159	1
PPRC1	0.0129964	1
SLC25A24	0.0303547	1
NLE1	0.0187282	1
CMTR1	-0.0262666	1
ACAA2	0.0722896	1
DECR2	-0.0233095	1
EME2	0.0017328	1
EEF1AKMT4	0.0286201	1
MLLT11	-0.0276216	1
ENTPD7	0.0145263	1
RAB11FIP3	-0.0202029	1
ZEB2	-0.2445092	1
MSH3	-0.043421	1
ARHGAP26	0.0606676	1
CLTA	0.1038754	1
LRCH1	0.011276	1
MARK4	-0.0183826	1
AC020910.5	-0.0077051	1
NFYA	-0.0076209	1
KCNMB1	0.0441663	1
BX842570.1	-0.0279026	1
PLP2	-0.1199531	1
TAOK2	-0.0134145	1
GCSAML	0.0655766	1
C1QTNF3	-0.0170858	1
PSMA3	-0.0093368	1
TMEM238	0.0434199	1
SLC35B4	0.0320099	1
RHOBTB1	0.0181854	1

CHD9	-0.0637041	1
MTMR2	0.0031721	1
PSPC1	-0.0223255	1
TBL3	0.0143083	1
IL12A	-0.0217026	1
AL731563.3	-0.0257527	1
APBA3	-0.0053908	1
ZNF131	0.0220979	1
LINC02591	-0.0337146	1
TRIM52-AS1	-0.0395408	1
ADRB1	-0.0103351	1
HOXB-AS1	-0.0175022	1
XYLT2	-0.0016679	1
ATXN2	-0.053759	1
MPST	0.0187917	1
AC007114.1	0.0061367	1
AC008267.5	0.0162873	1
AL359504.2	0.0372968	1
AP5S1	0.0134566	1
KIAA1522	0.0074214	1
MZF1-AS1	0.0861444	1
MCPH1-AS1	-0.014278	1
FYN	0.0786846	1
NCAPG2	0.0079144	1
LNPEP	-0.093782	1
ZNF250	-0.002475	1
MRPL58	0.0062561	1
KTN1	-0.1253851	1
ASF1A	-0.0419983	1
UHRF1BP1	-0.0437205	1
TRMT12	0.0041742	1
TBC1D5	-0.0678496	1
CHD3	-0.0295114	1
BORA	-0.0005125	1
MCU	-0.001701	1
SS18L1	0.0012413	1
WDR77	0.0048791	1
WRAP53	-0.0025988	1
ZRANB2-AS	-0.0256441	1
PGRMC2	0.0162058	1
AC093525.6	-0.005838	1
DDX18	-0.0689065	1
FAM49B	-0.0063122	1
ZFAND1	-0.006905	1
DNAJC25	-0.0147695	1
CCND2	0.1015094	1
SYNCRIP	0.0268639	1
AC240274.1	-0.0271883	1
SEMA4A	0.0055999	1
TRIM34	0.0022988	1

DNAJC28	-0.0276922	1
MORF4L1	-0.0872719	1
SIRPA	-0.0217073	1
ANGPT2	-0.0192912	1
CCDC61	0.0014715	1
BRK1	0.0537505	1
FAM27C	-0.0295834	1
SSR1	-0.0905237	1
EHD1	-0.0104328	1
H3F3B	0.1271988	1
SPATA5L1	0.0051034	1
ANKRD44-IT	-0.0056329	1
RGS18	0.0762231	1
N4BP1	-0.0303741	1
FAM92A	0.0291822	1
MINPP1	-0.0157881	1
RALGAPA1	-0.056104	1
RPS19	0.0731081	1
HSF1	-0.000411	1
ZBTB42	0.0073379	1
GOLGA5	-0.0003022	1
PLOD3	0.0288225	1
DHX34	-0.0017023	1
MEGF9	-0.0557104	1
TOMM34	0.0118714	1
OSGEPL1	-0.0170179	1
PXN-AS1	-0.0040726	1
DDX6	-0.0780877	1
ZNF106	-0.0687195	1
OBI1	0.001997	1
PARN	-0.0135261	1
SPG7	-0.0122126	1
DNAAF5	-0.0027484	1
NCK1-DT	-0.0009474	1
LIN52	-0.0258304	1
OXNAD1	-0.0111814	1
THOC2	-0.0125744	1
RXYLT1	-0.0596535	1
ZSWIM8	-0.0468564	1
TCEAL1	-0.0007539	1
PIK3CA	-0.0418575	1
CHKB	0.008979	1
C16orf87	-0.0260336	1
CIR1	-0.0773105	1
BTF3L4	0.0129835	1
OPA1	-0.0085917	1
KPNA5	-0.0759065	1
PTEN	-0.078647	1
PEX6	-0.0016673	1
HSPBAP1	-0.0506039	1

PARD6A	0.0088712	1
COQ6	-0.0136091	1
KDM5B	-0.0432898	1
PYGL	0.0196767	1
PEX16	0.0091112	1
ZNF329	0.0027111	1
BAZ1A	0.0311288	1
ACOX3	0.006089	1
WT1-AS	-0.0005182	1
CCHCR1	0.0021077	1
ZNF589	0.0050055	1
ZNF326	-0.0835589	1
C2orf42	-0.0118081	1
SELENOS	-0.0689035	1
C11orf49	0.0159282	1
PLEKHG4	-0.0074267	1
AGAP9	-0.043772	1
HMCES	-0.0146639	1
ZKSCAN1	-0.0064464	1
CCT4	0.0689631	1
RHBDD3	-0.0070636	1
MIR99AHG	0.0115296	1
DOLK	0.0041677	1
CBR3-AS1	-0.0470969	1
TRMT10C	0.0707057	1
PIEZO1	0.0144232	1
CREBL2	0.0242065	1
NPC1	-0.0347035	1
FAM161B	-0.0107927	1
PIK3IP1	-0.0690627	1
BEX3	0.1050337	1
TLR1	0.0043574	1
TAFA2	0.0658467	1
PUM1	-0.0578432	1
MAP3K20	0.0309188	1
ZNF440	-0.0208741	1
NECTIN2	-0.0062078	1
FAM104A	-0.0128428	1
AC097382.3	-0.0089081	1
ZNF677	0.0075562	1
THEM6	-0.0037447	1
DCAF5	0.0040028	1
GATB	0.017089	1
NME7	0.0248841	1
SIK2	0.0066172	1
BIRC6	-0.0543054	1
ATP6V0E2	0.0354621	1
ZNF571	-0.003846	1
MMRN1	0.1049511	1
EIF4E3	-0.0674085	1

CHCHD7	-0.0240833	1
LYRM7	-0.0446092	1
TMEM102	0.0139177	1
SLC24A1	-0.0180218	1
IQCB1	-0.0067986	1
CRIPT	-0.0533984	1
RRBP1	0.1435812	1
KIFC3	-0.0512508	1
CDV3	0.1130966	1
AGPAT3	0.0158647	1
TRIM37	-0.0385168	1
LATS1	-0.046286	1
KNOP1	0.0112181	1
TMEM161A	0.0416857	1
EPHA1-AS1	0.0200793	1
SLFN12L	-0.0403801	1
AC021097.1	-0.018759	1
CFDP1	0.0421671	1
NUDT16	-0.0086164	1
LRFN4	-0.0100238	1
SMIM6	-0.006892	1
PNO1	-0.0322938	1
SYNRG	-0.0436244	1
METTL9	-0.0122286	1
SAP25	-0.0100089	1
AC008443.1	-0.0147587	1
PPFIA1	-0.0526928	1
TUBGCP6	0.0063833	1
LINC02615	0.0002223	1
MOCS2	-0.0179246	1
BMI1	-0.0718534	1
CALML4	0.0095417	1
PCTP	-0.0202155	1
SLC25A29	0.0043268	1
PIP5K1B	-0.0254093	1
HOXA4	-0.0278292	1
CELF6	-0.0089119	1
CA2	0.0307819	1
KIAA0586	0.0030475	1
TRNT1	0.0014525	1
FBXL6	0.0335663	1
DUSP18	-0.0329173	1
PI4K2B	-0.0068827	1
HCK	0.0178355	1
CHD4	0.0682409	1
EID2B	0.0254479	1
ATP6V1C1	0.0363856	1
HLA-DRB1	0.0520821	1
RANBP2	-0.0148347	1
RPS7	0.0797611	1

SYNE1	0.0218215	1
ZFC3H1	-0.0276232	1
KLHL42	0.0216075	1
EFCAB14	-0.0485411	1
ABO	-0.0562267	1
HIBADH	-0.0303219	1
AGBL5	-0.0025522	1
CLEC12A	0.0467427	1
CEP89	-0.0319571	1
DST	-0.0737803	1
UBE3D	-0.0189936	1
RANGAP1	-0.0073935	1
MCTS1	-0.0113332	1
AC093627.5	-0.0141527	1
CCDC92	-0.0060672	1
KIF11	0.0327935	1
MAN1A1	0.1083028	1
TTI2	-0.0060598	1
TVP23C	-0.0173252	1
CYB561	0.0187916	1
CDYL	-0.0789681	1
JADE2	-0.031698	1
CRNDE	-0.0702609	1
SFPQ	0.1073898	1
TENT4A	-0.0148266	1
C19orf12	0.0012314	1
UBE3A	-0.0400777	1
ZNF672	0.0178274	1
ELK1	-0.0029322	1
WDR53	0.0137137	1
TPRN	-0.0094409	1
ATP5MC3	0.1250578	1
METTL14	0.0040465	1
ELMO2	-0.0181597	1
BAG2	0.0082117	1
RNF121	-0.0214413	1
ZNF783	-0.017633	1
SRP72	0.0751354	1
PNRC2	0.0541384	1
OAS1	0.0797357	1
AC245884.8	-0.013953	1
HLA-F-AS1	-0.0097174	1
RINT1	-0.0151064	1
PROSER2	-0.0032224	1
CEACAM4	0.0352957	1
PNPLA6	0.0394705	1
HPDL	0.0233003	1
ZMAT1	0.0236038	1
INIP	-0.0085613	1
LINC02328	0.0208727	1

FAM214B	-0.0189079	1
PRKACB	-0.1215605	1
AEBP1	0.0008194	1
ANKRD52	-0.0221854	1
PCBD2	-0.0301164	1
C8orf37	-0.0311553	1
FBXO8	-0.0022592	1
MECR	0.0182093	1
GATM	0.0028246	1
FMR1	-0.0387436	1
TCEANC2	-0.0163553	1
FASN	-0.0035182	1
MIR29B2CH	0.0590174	1
MIA3	-0.0680919	1
MAPK3	-0.0179297	1
SMC3	0.0838808	1
NCOA5	-0.0194939	1
ZFX	-0.0462962	1
AL133410.1	-0.0190694	1
PPP1R12C	-0.0149504	1
CAAP1	0.0205817	1
NDUFS4	-0.069498	1
AL121603.2	0.0098886	1
TMOD2	0.0265993	1
IQCE	-0.0208681	1
ARNTL2	0.0118908	1
FGFBP3	-0.0175035	1
NDUFA2	0.0149219	1
MAGT1	0.0050193	1
MINDY3	0.0013944	1
NUP35	-0.011734	1
ABHD11	-0.0061997	1
USP40	-0.0037428	1
GAB2	-0.014122	1
EMC2	-0.0015313	1
XRCC1	0.000577	1
HTR1F	0.0010049	1
SF3B1	0.065318	1
CCDC93	0.0182816	1
GCFC2	-0.0100047	1
XXYLT1	-0.0523199	1
C3AR1	0.0743603	1
IL6R	-0.0078402	1
BX890604.2	-0.0535889	1
PRDM16	-0.0614338	1
NUFIP2	-0.0720827	1
UQCRC2	0.0414062	1
RXRB	0.0102883	1
NDUFV3	-0.0377652	1
LINC01572	-0.0175092	1

C2CD5	0.0047528	1
PSAP	-0.1010661	1
EXOC1	-0.0248859	1
DHX38	-0.0051335	1
PSMG3-AS1	-0.0050442	1
DENND3	-0.0210631	1
UBN1	-0.0417861	1
CYTH1	-0.0053021	1
HDAC4-AS1	-0.0088125	1
POFUT1	-0.0309596	1
KLHL15	0.0023803	1
PRKD2	0.0225565	1
PDE4D	0.0151003	1
ATP6V1D	-0.0063346	1
ZNF182	-0.0168167	1
RRP12	0.0405819	1
SPCS1	-0.007568	1
SMARCD1	-0.0238779	1
CDKN2AIP	0.0449801	1
ZKSCAN2	-0.0027199	1
WDHD1	0.0346939	1
MMP25-AS1	0.0155609	1
ALKBH6	-0.0127184	1
MRPS17	0.0184007	1
HIST2H2BE	-0.0061583	1
MMS22L	-0.0360919	1
NAA80	-0.0162171	1
AC093690.1	-0.036511	1
ZNF684	-0.0059343	1
GFM2	-0.0049117	1
BDP1	0.0633857	1
KLHL8	-0.0076223	1
MCAT	0.0329433	1
CRELD1	0.0203741	1
FUT11	-0.0103399	1
DIDO1	-0.0170339	1
ZNF770	-0.0527454	1
UBA6	-0.0186968	1
SNUPN	-0.0439399	1
PSKH1	0.0074088	1
PDE6G	0.0236598	1
ATP10D	-0.0643411	1
LINC02175	0.0034242	1
ADCK2	0.02297	1
APEX2	-0.0125308	1
TUT4	-0.1126815	1
RPF2	-0.0133854	1
PCGF6	-0.0112827	1
AKAP8	0.010144	1
GTF2A1	-0.0204742	1

NSDHL	0.0072382	1
POLR3E	-0.0124137	1
ATPAF1	0.0525213	1
BTBD10	0.0184395	1
SPATA5	-0.0109268	1
OGFOD1	-0.0036587	1
ZNF846	-0.0245778	1
GM2A	-0.0590726	1
SEPHS1	0.0079204	1
GADD45GIP	0.1020457	1
LAMTOR1	0.0966265	1
ARMC2	-0.0105179	1
CCNQ	0.015513	1
CCDC84	0.0015	1
ABCF3	0.0092691	1
FNDC3B	-0.113808	1
CYB5D2	0.0083868	1
LINC01678	-0.0419153	1
PABPC1L	0.0121327	1
SKA2	0.0043378	1
CCDC18	-0.0135699	1
TBCE.1	-0.0342754	1
SEC31B	-0.0171253	1
NFIC	-0.0133522	1
ACAD11	-0.0034475	1
CCDC127	0.0081843	1
AC099343.3	0.0386013	1
HPS5	-0.0108724	1
GTPBP1	-0.0123746	1
DEDD	-0.0056266	1
CMTM3	0.0852497	1
LRWD1	-0.0162191	1
USB1	-0.0176102	1
RMI1	-0.0219738	1
NRAV	-0.0108884	1
MRPL20-AS	0.0080362	1
KCTD21-AS	-0.0363382	1
NAXD	-0.0083166	1
RFT1	0.0132212	1
HIST1H2AH	-0.0154805	1
NFKBIB	0.0115163	1
PRKAB1	-0.0233278	1
INTU	-0.0191775	1
SELENOI	-0.0148343	1
DPAGT1	0.0100244	1
ZNF665	-0.0137805	1
CERNA2	-0.0199126	1
ZNF583	-0.0110776	1
TESK1	0.002886	1
ASAP2	0.0286633	1

COX18	-0.0184409	1
PAQR6	0.0347949	1
AC093157.1	0.0026932	1
MACF1	-0.0044506	1
U2AF1	-0.0217198	1
SH3BGRL3	0.0943379	1
RPL6	0.0869452	1
C7orf50	0.0038249	1
CHM	0.010537	1
RAVER2	0.0379797	1
COX17	0.0049212	1
PASK	-0.0144723	1
SMPD1	-0.0176477	1
SLC35B2	0.0045783	1
TNRC6B	-0.0756587	1
GOLGA8B	-0.0347283	1
NCOA2	-0.0220607	1
ARHGEF7	-0.0053874	1
KLRG1	0.0483041	1
GAB1	0.0198447	1
RPL17	0.0973682	1
AP002495.1	0.0105071	1
CLMN	0.0300422	1
JAK1	-0.061037	1
ZNF568	-0.0020254	1
MAD2L1BP	-0.0052434	1
NUDT7	0.0064053	1
OGFOD2	0.0247617	1
LINC02802	-0.0308416	1
ZNF662	0.0211654	1
SLMAP	-0.0152725	1
SPTLC2	-0.049477	1
AC010969.2	0.006749	1
STIMATE	-0.0196401	1
BDNF-AS	0.0141414	1
AL645568.1	-0.0038616	1
ARHGEF6	-0.0193156	1
UEVLD	-0.0018916	1
CMTM7	0.082373	1
IL11RA	-0.025291	1
ZNF432	-0.0313064	1
AC108463.3	0.0291275	1
PAFAH1B1	0.0042218	1
ALDH2	-0.0539704	1
ATRAID	0.0476164	1
MARVELD1	0.0235316	1
LSS	-0.0032961	1
DHX35	-0.0098702	1
JMY	-0.0148328	1
TAF5	-0.0011192	1

ARMC9	-0.0433432	1
HSPBP1	0.0171938	1
TMEM9B-AS	-0.0036268	1
HOXA9	-0.1654532	1
YJU2	-0.0188116	1
LRRC8B	0.0260039	1
ZDHHC14	0.0180619	1
RBKS	-0.0359957	1
CENPK	0.0516391	1
SLC27A2	0.0471675	1
DDX52	-0.0461148	1
SCOC	-0.0040907	1
ARFGEF1	-0.0402374	1
LINC02848	-0.0172597	1
MON1A	0.0098239	1
MAP3K7	-0.0383463	1
ZFAND2A	-0.0117041	1
ZNF599	-0.017226	1
ZNF81	-0.0048838	1
MYCNOS	0.0152909	1
POLA1	-0.0016486	1
PGBD4	-0.0130252	1
CACUL1	-0.0443796	1
SP140L	-0.0130085	1
ZNF418	-0.0047814	1
AC026979.2	0.0472164	1
NIF3L1	-0.0165414	1
PKD1	-0.0337184	1
SF1	0.0797626	1
TLK1	0.0166241	1
FKBP14	-0.0283545	1
HSD17B8	0.0420024	1
AC008014.1	0.0414902	1
LINC00630	-0.0038686	1
WDR7	-0.0232951	1
APC	0.0054707	1
ILRUN	-0.0299399	1
POLH	-0.0417557	1
TUBG1	0.0309895	1
ZNF75D	-0.0298133	1
PSMB1	0.1012796	1
EXOG	0.0070436	1
PI4KA	-0.063109	1
TOMM7	-0.1005197	1
HERPUD1	-0.0035376	1
TRMT10A	-0.0122134	1
MIR4435-2H	-0.0021986	1
HMBS	0.0170416	1
ZNF317	-0.0013702	1
GEMIN4	-0.0148116	1

AC090152.1	0.0668098	1
CD46	-0.1294357	1
TENT4B	0.011988	1
PHF19	0.0514269	1
RMND5B	-0.0112792	1
NOL6	-0.0432303	1
AC132872.1	0.0021922	1
ZNF766	-0.0258252	1
TBC1D19	-0.0055106	1
SLC11A2	-0.0411845	1
DDX21	0.1137431	1
EIF2B3	-0.0043148	1
ANKRD44	-0.0037178	1
ZNF34	-0.0049438	1
CSPP1	-0.0014773	1
ZNF554	-0.00114	1
SLC31A1	-0.0045309	1
ZSCAN16	-0.0099062	1
ZYX	0.0186561	1
OPN3	0.0032736	1
PHACTR1	-0.0436576	1
MSTO1	0.0140305	1
KLHL28	-0.0178101	1
TUBGCP2	-0.0095279	1
PLAGL1	0.0219091	1
CREB3L4	0.016576	1
URB1-AS1	-0.0074854	1
KPNA1	-0.0250128	1
AC104596.1	-0.0213211	1
LSM5	0.0089973	1
SLC39A9	-0.0557778	1
GEMIN6	0.0156498	1
SLC39A7	-0.0047812	1
NIN	0.0259599	1
NAT1	-0.0023587	1
RMI2	0.0347717	1
LINC01238	-0.0048871	1
ZFYVE19	0.0217444	1
RPL27A	0.077785	1
RNF170	0.0047214	1
CSDE1	-0.0282061	1
EIF3H	-0.0667647	1
EPC2	-0.050294	1
MRPL53	-0.0442289	1
HELZ	-0.0138817	1
PNRC1	-0.1167516	1
MRGBP	-0.0178244	1
ARL13B	0.0084317	1
GPATCH11	-0.0533041	1
RNF149	-0.035268	1

LIME1	0.0832115	1
GRK4	0.0226457	1
F2R	0.0028873	1
ACOT4	0.0135645	1
RIOX2	0.0149396	1
RNF169	0.0308054	1
ARID4A	-0.0457897	1
AK3	0.0147517	1
LYRM9	0.0008817	1
TCTN2	-0.0180592	1
HHAT	-0.0131238	1
ZNF200	-0.0179023	1
SEC11A	-0.0095473	1
ZNF75A	-0.0212592	1
NIPAL3	0.0194582	1
CIP2A	-0.0110146	1
SNHG11	0.0173278	1
LRRC69	-0.0284123	1
AC133550.3	-0.0108336	1
FAF2	0.0284415	1
SEPSECS	-0.0148101	1
MLH3	0.0366582	1
TRAM1	-0.0411474	1
PDZD8	-0.0852471	1
ABCB8	-0.0010535	1
NBPF1	-0.0183172	1
IPO11	0.0208471	1
RAB31	0.103954	1
WARS2-AS1	-0.0153514	1
FXR1	-0.1561977	1
DUSP28	-0.0173581	1
ALDH1B1	0.0296855	1
DNAJC18	0.0335831	1
DCAF1	-0.0101993	1
L3MBTL3	-0.0117129	1
ABCF1	-0.0083544	1
RPSA	0.0929917	1
DET1	-0.0093776	1
KIAA0513	-0.0187246	1
DTWD1	-0.0311358	1
ANKS3	-0.0037251	1
CYB5RL	4.252E-05	1
BDH1	-0.0113683	1
TARBP2	-0.0250908	1
ATP5MF	0.0573837	1
ANAPC1	-0.0190207	1
ZNF473	-0.0111945	1
MBOAT1	-0.0068867	1
CEP68	0.0337986	1
RGS12	-0.0452097	1

ATF6	0.0312632	1
ATF7IP2	-0.0428336	1
RPGR	0.0215904	1
ANGEL1	-0.0037314	1
AC007038.1	-0.0192692	1
MINDY2	0.0291492	1
NINJ2	-0.035963	1
C19orf44	-0.0061174	1
ZW10	0.0175634	1
HIST1H2AE	-0.0127665	1
ARMCX5	0.0076502	1
WDR4	0.0353621	1
GBF1	-0.0121093	1
MYO1B	0.0675235	1
AP000845.1	0.0332805	1
RAC2	0.1128905	1
LAMTOR5-A	0.0215955	1
LINC02693	-0.0159238	1
PDE4DIP	-0.0707912	1
PELO	0.030279	1
TMEM192	-0.0499961	1
AC109460.2	-0.0219556	1
C15orf41	0.0027073	1
LINC00894	-0.0344095	1
AC005076.1	-0.0347522	1
VCPKMT	0.0128343	1
SMAP2	0.0609642	1
TCOF1	0.0348119	1
COX4I1	0.091987	1
PGBD1	-0.0182856	1
COX7A2	0.0455559	1
METTL1	-0.0130608	1
ETS2	0.1720184	1
ZC3H14	-0.0118947	1
GTPBP8	0.0361708	1
KAT2B	0.0094976	1
INF2	0.0235288	1
OAS3	0.0444285	1
ZNF10	0.0028087	1
ZDHHC18	0.0165425	1
TAF13	-0.0081576	1
RPGRIP1L	-0.0062181	1
EIF3J	-0.0566135	1
AC090517.5	0.0041641	1
LRP10	0.0672442	1
SYNGAP1	0.0184348	1
PPIA	0.0995271	1
DDX23	-0.0204981	1
ZNF37A	0.0255009	1
ATF5	-0.02912	1

ACSF2	0.0012214	1
TLR6	0.0279386	1
PPT2	0.016418	1
RBM38	-0.005743	1
ZNF773	0.0041128	1
LINC00921	-0.0046557	1
SLX4	-0.0214059	1
HNRNPA0	-0.117764	1
HERC6	0.0120056	1
TMCO1	-0.0111353	1
ZNF23	-0.0078104	1
EXOC7	-0.0045227	1
CCDC25	0.0445779	1
HGF	0.0676022	1
RESF1	0.0189049	1
PAN2	-0.0015893	1
RCCD1	0.0096788	1
CNOT4	-0.02402	1
CFAP70	-0.0231402	1
SCD5	0.041863	1
TWNK	0.0073237	1
TRIM4	0.0080843	1
DICER1-AS1	-0.0050945	1
DOCK5	-0.0466802	1
DNTTIP2	0.0690906	1
STEAP3	0.0113603	1
GCH1	-0.0028545	1
HIST1H2AL	-0.0343038	1
CPNE8	0.026316	1
CUL5	-0.0327728	1
COPS4	0.0301511	1
SLC22A5	0.0100602	1
PUDP	0.0136749	1
HOXA10-AS	-0.0740917	1
EPS8	0.0623977	1
SNHG3	0.0920895	1
CPXM1	0.0291863	1
HAGH	0.0064611	1
AL365295.1	0.0571045	1
ZC3H12A	-0.0087713	1
PAQR8	0.022157	1
AC090559.1	-0.1337074	1
CWC15	0.0362324	1
TMEM50A	-0.0599853	1
SPECC1L	-0.0210664	1
SARNP	0.0158892	1
SHMT1	-0.0016901	1
FANCD2	0.0087065	1
POLD1	0.0448699	1
STK35	0.0126113	1

SCYL1	0.0031179	1
CCDC102A	0.0081198	1
DVL2	-0.0220236	1
ZNF213-AS1	-0.0318596	1
LSM3	0.0502198	1
MOV10	0.0201763	1
GHDC	-0.0084882	1
SNX18	-0.0455958	1
RB1CC1	-0.086614	1
BEND5	0.0427223	1
HEMK1	-0.0164448	1
MTMR12	-0.0064735	1
PCNX3	-0.0185435	1
AC073111.4	-0.006002	1
CYBA	0.1209375	1
UBE4B	-0.0387041	1
AC027644.3	-0.0081036	1
ATP6V0B	0.0592115	1
SLC25A36	0.0651763	1
TMEM237	-0.0096911	1
ERCC4	0.0040727	1
HNRNPR	-0.063255	1
PCSK7	-0.0401711	1
TM9SF3	-0.0709688	1
TMEM109	0.0327631	1
ORAI3	-0.0289828	1
ROCK1	0.071399	1
C12orf75	0.087224	1
ZNF318	-0.0146745	1
LARS	0.0450703	1
RRAGD	0.0007162	1
RSRP1	-0.0835691	1
B3GNT5	0.0115813	1
NAB1	0.0048623	1
PSD4	-0.0453607	1
AC046134.2	0.0028405	1
AC027097.2	-0.0081768	1
TARSL2	-0.0484223	1
ASB1	-0.027957	1
TCTEX1D1	-0.0124443	1
NDST1	-0.0274846	1
BCL7A	0.0916035	1
AC008443.5	-0.0037725	1
DYNC1I2	-0.0979845	1
CHRAC1	0.0397493	1
ZNF721	0.0132546	1
FAM110A	0.0108002	1
CCDC167	0.0547043	1
RPUSD1	0.0255854	1
RBSN	0.0045317	1

RBMX	-0.073286	1
EXOSC9	0.000443	1
GUCY1B1	0.0968074	1
AC005674.2	0.000483	1
MLST8	0.0469446	1
NDUFB11	0.0601705	1
AC010680.4	0.0019909	1
LMLN	-0.0151485	1
AMT	0.0167774	1
SLC16A5	-0.0203201	1
FBXL8	-0.0047922	1
LAT	0.0184142	1
YY1AP1	-0.0065023	1
PDCD6	-0.0787233	1
PWWP2A	0.0148978	1
NDUFA11	0.084649	1
ZMYND8	0.0713262	1
NAALADL2	0.0112995	1
TMTC2	-0.0056831	1
ANXA4	-0.0754601	1
FNBP4	-0.0466313	1
MAX	-0.1310603	1
MROH7	-0.0053344	1
UTF1	0.0460104	1
R3HDM1	-0.0172564	1
HSPA14	-0.010874	1
LSM8	-0.0677818	1
SNX14	-0.0065945	1
LIG4	-0.0075063	1
DHCR7	-0.0619817	1
MTMR4	0.0040416	1
FUT8	0.0038977	1
BBIP1	-0.0120321	1
AC068888.1	-0.012889	1
DOCK2	-0.0407855	1
ICA1L	0.0080259	1
ZNF486	-0.0124685	1
PIP5K1C	-0.0253821	1
REC8	-0.0265087	1
ERCC2	-0.0071142	1
ADSS	-0.025286	1
AC110769.2	0.0031638	1
USP5	-0.014989	1
AC009779.2	0.0111307	1
TBC1D24	-0.0310074	1
DLC1	-0.0152241	1
TERF1	-0.0057624	1
CASP6	0.0146846	1
ELOA-AS1	0.0157508	1
IBA57	0.0455035	1

CRADD	0.0123288	1
ACY1	0.0108202	1
PWP1	0.0089905	1
ATRX	-0.0925817	1
CIDEB	0.0048128	1
SCOC-AS1	-0.0376918	1
NLRP3	0.0532139	1
TMEM175	0.0064383	1
AL109615.4	-0.0297412	1
TSPAN31	0.0078011	1
PRDM8	0.0188434	1
CBX2	0.0160641	1
CYP4V2	-0.003732	1
OCIAD1	0.0234776	1
FAM185A	-0.0098486	1
HIST1H2BG	-0.0331767	1
EPC1	-0.0969053	1
CRTC1	0.0086656	1
FGD3	-0.0550496	1
RNF217	0.0043171	1
RANBP10	0.0089343	1
HIVEP2	0.0322636	1
ACBD3	0.0449751	1
AC024560.4	-0.0114159	1
ARMT1	0.0087388	1
DHTKD1	0.0919671	1
AL135999.1	0.0017745	1
SVBP	-0.0450679	1
IFT22	-0.0145709	1
AL355075.4	0.050535	1
NCOR1	-0.0915131	1
SEPSECS-A	-0.0208058	1
NDUFA3	-0.0901944	1
NIFK	0.0170099	1
TTC28	-0.019232	1
POU5F2	-0.0119304	1
MDH2	0.0812557	1
NDEL1	-0.0200951	1
SOCS6	0.0060213	1
YTHDC1	-0.1070873	1
COPS9	-0.0627898	1
AC009318.1	0.1315811	1
SLC39A8	-0.0004566	1
LXN	-0.0320611	1
RHOBTB2	-0.0024733	1
CRYBG3	0.1083659	1
ZNF786	-0.0191805	1
TMEM263	0.0089583	1
NUP214	0.0082491	1
IL10RB-DT	-0.0279011	1

CDC14A	0.0333728	1
NUDT3	-0.03848	1
AC103703.1	-0.0177381	1
RAB3IP	-0.0357027	1
NCAPH2	0.0040938	1
DEPDC7	-0.0069931	1
SRSF2	-0.0105918	1
WWOX	4.322E-05	1
MT1F	0.0440294	1
GDF11	0.0507465	1
OBSCN	0.0382921	1
PGBD2	0.0092217	1
ARHGAP10	-0.010892	1
CCT5	0.0885141	1
UMODL1-AS	0.004422	1
VGLL4	-0.0038519	1
DHRS11	-0.0126437	1
MOB3C	0.0056598	1
PTS	0.0039646	1
SHLD3	0.0037443	1
TFR2	0.0003756	1
SEPTIN7	0.0071532	1
HACD1	-0.0279093	1
EIF4G2	-0.1279401	1
AL136038.5	-0.0074366	1
DFFA	-0.0021885	1
SLC35A2	0.0176249	1
PNISR	-0.0630218	1
PACS2	-0.0096262	1
ZNF792	-0.0218708	1
WBP4	0.0523188	1
ERH	0.0175499	1
AC135050.6	-0.0253049	1
ATG4D	0.0006497	1
NUB1	-0.0400169	1
SUCO	-0.0619146	1
CLINT1	-0.0531549	1
RAB8B	0.0295481	1
PRKAR2A	-0.0398292	1
ZNF799	-0.0134128	1
RPS6KB1	-0.020786	1
GFOD1	0.0429454	1
ENSA	0.0490401	1
SAMD8	0.013302	1
VPS41	-0.010385	1
AP001816.1	0.0107395	1
TMEM219	0.0287546	1
LINC01578	-0.1142055	1
AC011447.3	-0.0252801	1
NSD3	-0.0457828	1

CCDC102B	-0.0319451	1
CD53	0.0333625	1
LINC00342	-0.0811969	1
CEP63	-0.0525354	1
APTR	0.0029807	1
AC021054.1	-0.0175658	1
CAPS	0.0009962	1
PSMA4	-0.0118396	1
ZNF354A	-0.0186838	1
CAMSAP1	-0.0244728	1
AC093827.4	0.0265962	1
RAP2A	-0.0211761	1
SPCS2	0.0354409	1
GART	-0.0129122	1
TNKS2	-0.0301393	1
EEF2KMT	0.0004447	1
SENP6	-0.0018748	1
VIPAS39	-0.0106066	1
FOXP1	-0.0779891	1
BPNT1	-0.0004048	1
AP003307.1	-0.0001123	1
SRPK2	-0.0193971	1
RILPL2	-0.0609736	1
KRTCAP2	0.1037125	1
SGTB	-0.0186538	1
PEX14	-0.0013049	1
TNNI2	0.065549	1
TOP3A	0.0024589	1
FAM117B	-0.0135074	1
C19orf81	-0.0282337	1
AL021155.5	0.0362486	1
STIM1	0.0236585	1
TBCK	-0.0407078	1
TWSG1	0.0464533	1
IER5	-0.0636753	1
SYVN1	-0.0375496	1
LAMTOR5	0.0851003	1
PSMD8	0.0821957	1
HIST1H1D	0.1882721	1
MAP2K2	0.1242864	1
FADD	0.0064923	1
Z99127.4	0.0478608	1
TIMM13	0.1115164	1
SMIM30	-0.0130994	1
MIOS	-0.0183279	1
PARPBP	0.0215057	1
NDUFA12	0.0342121	1
RNF130	-0.1431726	1
UAP1L1	-0.0079607	1
SF3B6	0.1433087	1

PRDM2	-0.0456003	1
DNAJC4	6.558E-05	1
C8orf76	-0.0028853	1
AMBRA1	0.0038669	1
NEK8	0.00926	1
SMPD4	-0.041961	1
FAIM	-0.0105902	1
ELF2	-0.0378964	1
HEATR5A	-0.0276693	1
MPP1	0.0337239	1
AL161421.1	-0.0020172	1
SPEN	-0.0849622	1
SIAE	-0.0077953	1
ZNF319	-0.0072675	1
ACD	0.0049745	1
CACNA1C-A	0.0157269	1
USP28	-0.0313585	1
TCF7L2	0.0507731	1
PRELID2	0.0454538	1
PREPL	-0.0378164	1
CREBRF	-0.0309869	1
PLEKHO2	-0.0034367	1
POLR3F	0.0028719	1
JMJD7	0.0041619	1
LSM11	-0.0079637	1
SMIM1	0.0177773	1
CRAT	-0.0104245	1
LACTB2	0.0288426	1
IGSF8	0.0004093	1
PCYT1A	-0.0127814	1
NIPBL-DT	-0.0016546	1
MED25	0.0128408	1
ARHGAP32	0.0191978	1
VWA8	0.0021014	1
SCHIP1	-0.0088053	1
PRCP	-0.0256424	1
ZNF682	0.0087743	1
EIF2AK2	0.0411381	1
SEC11C	-0.0008417	1
ARAP2	0.0085483	1
POU2F1	-0.0378794	1
EPGN	-0.0259501	1
RUBCN	-0.0428018	1
RAB44	-0.0482304	1
PGLS	0.0286551	1
HIST1H2AM	-0.0377717	1
SMYD2	-0.0087123	1
SPDL1	-0.025986	1
PLD1	-0.0908609	1
GRAP	0.0241119	1

UPP1	-0.0206544	1
ISCU	0.1245249	1
TSPOAP1-A	-0.0166343	1
KLHL21	-0.0165733	1
SNRPF	0.0253174	1
AL683813.1	-0.0096981	1
CFAP36	-0.1035737	1
LINC00467	0.0297903	1
LMF1	-0.0213689	1
GOLPH3L	-0.0055918	1
TXNDC5	-0.1531021	1
LINC00899	0.0310097	1
SCYL2	-0.0409215	1
MAP4K1	0.0099963	1
HIVEP3	0.0417572	1
PSMD4	-0.0071875	1
AC024560.5	0.0035545	1
IFT74	0.0144697	1
MAEL	0.0142427	1
TMEM254	0.0192672	1
IL12RB1	-0.0264797	1
RNF208	0.0095161	1
PCCA	0.0121022	1
NACC1	0.0013819	1
SMURF1	-0.0088574	1
NADK	-0.0118595	1
AC026124.2	-0.0068069	1
BDH2	-0.0456501	1
XRCC2	0.0325008	1
ZNF660	-0.0336286	1
ZFAT	-0.0026992	1
TRMT5	0.0200899	1
AKAP17A	-0.0109017	1
RNF185	-0.007945	1
ADGRA2	0.0013481	1
LINC01534	-0.0030825	1
CMC4	-0.0038804	1
SYTL2	-0.0232742	1
PPP2R1B	0.0194984	1
AC009120.2	0.003319	1
COX5A	0.1068734	1
AC234772.2	-0.0150812	1
HSBP1	0.0427803	1
C1orf54	0.0144058	1
RNASEK	-0.0987462	1
TMEM14A	0.0468388	1
CSNK2A1	-0.0519734	1
KDELR1	0.0199176	1
TTC37	-0.0578735	1
PSME1	-0.1118443	1

MICOS10	0.0536946	1
BUB1B	0.0257896	1
MTA3	0.0165036	1
LIN9	-0.0206135	1
MPND	0.0217089	1
LINC00653	-0.0224489	1
FAM162A	0.0348493	1
AL392172.1	-0.0091186	1
ASL	-0.0239976	1
PCCA-DT	0.0086864	1
FAM81B	-0.0133735	1
EXD2	0.0005855	1
LINC01684	0.0435411	1
ACADM	-0.0137929	1
TBCEL	0.0015231	1
SPATA2	-0.007459	1
CHI3L1	-0.0628718	1
EGFL8	-0.0091904	1
CU638689.4	-0.0008088	1
METTL22	-0.0308967	1
ZBTB9	-0.0076353	1
SULT1C4	0.0355772	1
HIST1H2BK	-0.001434	1
TMEM154	0.0234756	1
TCFL5	-0.0195324	1
SMARCE1	-0.044169	1
BAHD1	-0.0082062	1
PXMP4	-0.017073	1
DUSP7	-0.0008082	1
WAKMAR2	0.0122134	1
PROSER3	0.0061955	1
SYT11	0.0044055	1
CASP4	-0.039783	1
CERCAM	-0.0069423	1
CENPV	0.0191129	1
DCP1A	0.0169546	1
SLC2A11	-0.0103922	1
ADA	-0.0023499	1
RBL1	-0.0013017	1
MED15	-0.0152849	1
AC090204.1	-0.0010188	1
AKAP1	0.0101193	1
TMEM14C	0.0285477	1
AL662844.4	0.0102535	1
DYSF	0.0310343	1
CUL7	-0.0071109	1
OTUD3	-0.0123241	1
SLC2A3	0.0825897	1
CSTF2	0.0137164	1
EXOSC6	-0.0609259	1

RAC3	0.0456074	1
CMTM6	-0.0028946	1
XRCC4	-0.0076351	1
AC009065.4	-0.0129071	1
EIF4B	0.0123405	1
FAM200B	0.0080733	1
ZNF616	-0.038191	1
SLC43A2	0.0139223	1
NFIL3	-0.0729279	1
MRPL48	-0.0010725	1
MTIF3	0.0017757	1
PAK2	-0.109208	1
DCAF4	0.0134461	1
ASH1L-AS1	0.0189927	1
AP002381.2	-0.024866	1
MBP	0.1198074	1
SBF1	0.0086314	1
QSOX2	0.0106216	1
MTURN	0.0784745	1
OGFRL1	-0.0007048	1
CTSF	0.0378865	1
C6orf163	-0.0204728	1
MRPL42	-0.0681699	1
DPH6	0.0374228	1
DNAJC8	-0.0230355	1
SMARCA2	-0.1139214	1
EEF1B2	0.1033814	1
ITPKB	0.0083019	1
SCFD2	0.0149989	1
GK5	-0.0428021	1
SNRPE	0.0033312	1
HSP90B1	0.1582657	1
SENP1	-0.0393401	1
GRIK5	0.0303077	1
SOCS2-AS1	0.0311698	1
CCSER2	0.0234783	1
SETD6	-0.0228533	1
ATP6V0A1	0.0037916	1
MRPS18C	-0.0292185	1
HSPE1	0.1263008	1
EPS15	-0.0548418	1
ANXA2	0.0477806	1
UTP20	-0.0003355	1
KLC4	-0.0170503	1
NECAB3	0.0139597	1
SPATS2	-0.008112	1
RCAN3	-0.0012645	1
EFL1	-0.0006698	1
BICRA	-0.0096137	1
KTN1-AS1	0.0033842	1

LINC01134	-0.0258426	1
KCNQ5	-0.0335489	1
CACNA1A	-0.0054802	1
UTP14A	0.0008956	1
DPY19L2	-0.012945	1
DLGAP1-AS	0.0388824	1
ATP6V1F	-0.1017191	1
PTGS2	0.085948	1
PRKAB2	-0.012736	1
ZNF223	-0.0003451	1
WIPF1	-0.0084281	1
TRIB1	0.0588685	1
FPGT	-0.003227	1
COPS7A	0.0078511	1
AL050309.1	-0.0002466	1
GLUL	0.1493408	1
TMEM164	-0.0207084	1
HBD	0.90636	1
AL034397.3	0.1059738	1
DSN1	0.0220528	1
DHFR2	0.008204	1
TTC27	0.0493738	1
AL357079.1	0.0162325	1
HAUS5	-0.0026885	1
NUDT6	-0.0162934	1
CLYBL	-0.0290294	1
SLC25A14	-0.0007192	1
BGLAP	0.0293862	1
ZNF334	0.030924	1
AP006621.3	0.0313716	1
ZDHHC16	0.0062664	1
RPAIN	0.0023671	1
UBLCP1	-0.0793091	1
SPRY2	-0.009308	1
NLRC5	0.0319408	1
SCRN3	-0.0025585	1
ARMH1	-0.0586316	1
SNHG16	-0.051384	1
EPRS	0.1243356	1
DNAJC3	-0.0251264	1
ACY3	-0.0686516	1
NUMBL	0.0288155	1
NCBP2AS2	0.0172417	1
SWT1	0.0200319	1
PDGFC	0.043159	1
TPI1	0.0751359	1
USP42	0.0062776	1
SDCBP2-AS	-0.0268609	1
CHKB-DT	-0.0017131	1
ZNF614	0.0063276	1

RAB10	0.0949931	1
CCDC137	0.0934258	1
OFD1	0.0006322	1
ZNF485	0.0065961	1
AC004241.1	0.0110286	1
ASAP1	-0.006715	1
POGLUT3	0.0170838	1
PSME3	0.0088549	1
FLT3LG	0.00852	1
APOO	0.0333967	1
ZNF251	-0.0022056	1
HNRNPH3	-0.0210554	1
TET1	-0.0254631	1
TMEM265	-0.0193318	1
PRR34-AS1	-0.02783	1
USP54	-0.0126807	1
USP49	-0.0127974	1
KSR1	0.0283824	1
STAC3	0.023872	1
AC064807.1	0.0336149	1
UPF3A	-0.0833813	1
FBXO30	0.0191695	1
ZNF595	-0.016218	1
ZNF45	-0.0083832	1
AC105339.6	-0.0036147	1
HYAL3	-0.0192926	1
NIPBL	-0.0959499	1
HIPK3	-0.018524	1
SPICE1	-0.0141685	1
CDC37L1-D	-0.0033977	1
AQP3	-0.0639749	1
DMXL2	0.0206403	1
BPTF	-0.0652449	1
TSPAN14	-0.0256184	1
E4F1	0.0009364	1
DDT	0.11661	1
AC004889.1	-0.0057592	1
RAB29	0.0113806	1
AL162458.1	0.0102378	1
PIGB	-0.0287354	1
RFLNB	-0.1424677	1
ZFAND5	-0.0354156	1
AC147651.4	-0.0329852	1
JRK	-0.0149939	1
MRPS31	-0.0249367	1
PALB2	-0.0162678	1
BTD	-0.0058962	1
SPATA20	0.0096507	1
PSMA1	-0.0838138	1
PTPRA	-0.0122656	1

MIER2	-0.0015746	1
YWHAZ	-0.0106084	1
FAM234B	-0.0042324	1
JMJD1C	0.102057	1
GBA2	-0.0448964	1
ERCC8	-0.024329	1
CALHM2	0.0346556	1
RNF31	-0.0149809	1
MCM8	0.0298407	1
TECPR1	-0.0160047	1
TMEM268	0.0184159	1
SLC2A1	-0.044591	1
HYI	0.0052637	1
TMPRSS9	-0.0186509	1
CCSAP	0.0092807	1
CC2D1A	0.0064969	1
P2RY10	-0.0114604	1
ZNF280C	-0.026795	1
SRGAP2C	-0.0038757	1
CDC42	-0.0423457	1
PELI1	0.0013417	1
CYP2R1	-0.015178	1
IRGQ	-0.0117476	1
ZGPAT	0.0036337	1
POLRMT	-0.0478821	1
BCLAF1	-0.0643934	1
ZNF354B	-0.0326081	1
JAK2	-0.02113	1
CYB5D1	0.010635	1
AACS	-0.0121467	1
CD55	0.0084732	1
WAC-AS1	-0.060595	1
AC025181.2	0.0156075	1
NDUFAF2	0.0184443	1
PCBP1	-0.0944636	1
CITED2	-0.0427747	1
FRMD4A	0.0251293	1
PPM1K	-0.025633	1
MAFF	0.0680312	1
MLH1	0.0034641	1
NUBPL	-0.0187749	1
LDLRAP1	0.0401058	1
CNPY2	0.07468	1
TRAF2	0.0161578	1
DYTN	-3.209E-06	1
CLSTN1	0.0401048	1
PSMA6	0.1163607	1
MYL12A	0.1279751	1
TAF4B	-0.0040819	1
ACTG1	-0.0900051	1

GEMIN8	-0.0046488	1
NKAP	0.0289056	1
OCRL	0.0023816	1
ARHGAP19	0.0083871	1
SP2	-0.0092467	1
CEP57L1	-0.017188	1
PARP2	-0.0216851	1
BX640514.2	-0.0323227	1
TRAF4	0.0654377	1
SSBP1	0.0503085	1
LMBRD2	-0.0368525	1
NDUFS5	0.0080947	1
AC132192.2	0.0352797	1
EP400	-0.0517768	1
RFX1	-0.020096	1
CCDC191	0.0021955	1
CSRNP1	0.0389664	1
AC008915.2	0.0078117	1
RMDN2	-0.0089373	1
NEU3	-0.009701	1
GNE	-0.0239625	1
C3orf33	0.0037206	1
NOP14-AS1	0.0010296	1
NRDC	-0.030362	1
TFEC	-0.0009647	1
PAK4	-0.0140148	1
TICAM1	-0.0003164	1
SGSM2	0.0077489	1
ZNF213	-0.0031639	1
PYGO2	0.0009319	1
DNAJC17	0.0280442	1
TIRAP	-0.0064247	1
EIF3A	0.0657558	1
UBE2D3	-0.0167855	1
DEGS2	-0.0103722	1
PLK3	0.0431695	1
SPRED2	0.0289859	1
PSTK	-0.0037255	1
CENPP	0.0016075	1
MGAT5	0.0107863	1
DDX11	-0.0002235	1
GLS	0.1308375	1
AMER1	-0.0091497	1
AC145207.5	-0.0167954	1
P4HB	-0.0041893	1
AC093227.1	-0.0150818	1
ARFRP1	0.0284302	1
SEPTIN6	0.1215183	1
KMO	-0.018446	1
MSANTD3	-0.0079632	1

NFS1	0.0177319	1
RIPOR1	-0.0132318	1
FBXL4	-0.0015601	1
TASOR2	-0.0196495	1
EIF3E	-0.0580292	1
PRDM10	-0.0206393	1
IFFO2	0.0297911	1
ZNF658	0.0112011	1
C10orf143	0.0187339	1
LINC00211	0.055692	1
FCHSD1	0.0078934	1
GDPGP1	-0.006371	1
ZRANB2	-0.0885353	1
SSX2IP	-0.0251598	1
PRDM4	-0.0154773	1
CCT3	0.1311471	1
ZMYND19	-0.0081776	1
NAIF1	-0.0031551	1
RRAS	0.0149096	1
GRB2	-0.0913432	1
DNAJC27-AS	0.0132978	1
SLC25A53	0.0084136	1
LINC01857	-0.0068644	1
DHX33	0.0327258	1
TSTD2	-0.0202141	1
ZNF551	-0.0117661	1
ENOX2	-0.0179659	1
ZFP1	0.0151932	1
SLC25A42	-0.0091131	1
SLC52A3	0.0374742	1
MAK16	0.0291573	1
INTS2	-0.0088581	1
TSEN2	0.0085884	1
CCDC136	-0.0197859	1
ZNF543	-0.0221623	1
AC067930.8	-0.007013	1
ZNF404	-0.0176589	1
RNF20	-0.0298438	1
EMC1-AS1	0.006808	1
KBTBD4	0.0222755	1
THRIL	-0.0161967	1
HIST1H2BF	-0.0099615	1
CAMTA2	0.016103	1
CARM1	-0.0018573	1
STAG1	-0.0003806	1
MRM1	0.01115	1
CD58	-0.0034754	1
AC108047.1	-0.0154635	1
BPGM	-0.0326191	1
REST	-0.0871328	1

DAAM1	0.0539113	1
EXO5	-0.0032397	1
REEP5	0.0545665	1
UTP25	-0.0031424	1
NTAN1	0.0095451	1
ELL	-0.0119842	1
ZNF22	0.1216459	1
TMEM167A	0.0174047	1
RMND1	-0.0122088	1
CITED4	-0.0708267	1
NNT-AS1	-0.0026424	1
UBASH3B	0.0492461	1
KIF3B	-0.0257662	1
RASA1	0.0207922	1
COMMD3	-0.0177853	1
TRMT44	-0.0112313	1
AMN1	-0.0140637	1
LINC00909	0.0202013	1
HOMER1	-0.0265866	1
GSPT1	0.0841983	1
SACS	0.0300471	1
DGUOK	-0.0105812	1
POLA2	0.0185356	1
WDR35	-0.0234474	1
ZNF772	-0.0146354	1
EIF1AX	0.0791529	1
PLOD1	0.002552	1
AC092164.1	0.0076901	1
DHX36	-0.0363208	1
BRD3OS	-0.0103501	1
RBX1	-0.1193621	1
COX10	0.0138964	1
ZNF696	-0.0081067	1
ATP5IF1	0.0553223	1
DOCK8-AS1	-0.000225	1
PIGA	-8.644E-05	1
DDX24	0.1052317	1
ORMDL1	-0.1045738	1
CCDC59	-0.0021552	1
PATL2	0.0037775	1
STK4	0.0355623	1
KIF1B	-0.0073434	1
ALDH3A2	0.0054211	1
RELA-DT	0.0107314	1
NAA40	0.0098507	1
PEX19	0.0014231	1
DYRK1B	-0.0015573	1
INTS9	-0.0063507	1
AC010680.3	-0.003518	1
SEL1L3	0.0002305	1

TSPYL2	0.0467241	1
DENND1C	0.0130847	1
SH3BGRL	-0.0912053	1
ZNF382	0.0084345	1
GALK2	0.0030376	1
IFIT3	0.0156632	1
NSRP1	-0.0752414	1
RNF26	-0.0107725	1
CD27-AS1	0.0344481	1
NDUFB5	0.0252497	1
ABCC10	-0.0028054	1
ICAM4	0.0002237	1
HNRNPD	0.0586399	1
BX284668.5	-0.0278043	1
APLF	0.0023609	1
NAPB	-0.0072448	1
ZKSCAN7	-0.0156434	1
EIF5B	0.0620587	1
TRAM2-AS1	0.0063878	1
AC004076.2	-0.0114016	1
ZNF148	-0.0447303	1
ERICH6-AS1	0.0063511	1
MRPL24	0.058561	1
DHRS7	-0.0787851	1
TBC1D25	-0.032122	1
AL080250.1	-0.0117402	1
DALRD3	-0.0109124	1
ZNF629	-0.0269752	1
ATP8B3	-0.0212387	1
POLD3	0.0163002	1
CHIC1	0.0256921	1
TESC	0.0207727	1
E2F6	0.0156364	1
PRXL2B	-0.0197509	1
CENPS	0.0248016	1
HAGHL	0.069033	1
DIAPH2	0.0145768	1
GSTM3	0.0182124	1
CDR2	-0.0294757	1
SLC45A3	-0.0717854	1
EPS8L2	0.0482947	1
AC016727.1	0.0107584	1
AP1S3	-0.0016305	1
RRNAD1	-0.0072124	1
AL035587.2	0.0164654	1
ZNF292	0.0525583	1
TTC3	-0.0958966	1
GDPD1	0.0162579	1
ZNF76	-0.0193181	1
COL5A1	0.0349107	1

SLC35G2	0.0168167	1
DDHD2	-0.0274218	1
WDR24	0.0063909	1
B4GALT6	-0.023693	1
ADGRE2	0.0349013	1
ZNF484	0.0045523	1
RAB2A	0.0135093	1
RPP38	0.0099224	1
KMT2E-AS1	0.0835418	1
B4GALT7	0.0202616	1
BPHL	0.014338	1
POMT1	-0.0231602	1
ATP5F1C	0.0992612	1
FBXO5	0.0135875	1
BANP	0.0085178	1
THOC7	-0.0384054	1
NHEJ1	-0.0203685	1
MESP1	0.0415519	1
PKNOX1	-0.0077564	1
LCLAT1	-0.0216136	1
GALNT10	-0.0118653	1
LTO1	-0.0275164	1
AC009093.2	-0.0274991	1
AC069544.1	-0.0076388	1
MGA	-0.0677923	1
GPD1L	0.0042727	1
RAB4A	-0.0012256	1
DDX51	-0.0111483	1
CTSS	0.01781	1
C19orf54	-0.0046209	1
DLG4	0.0144094	1
GADD45B	-0.0058523	1
LINC00294	0.0145067	1
UROS	0.0639839	1
SLTM	-0.1323217	1
PIAS3	-0.0095207	1
SBF2	0.0127442	1
CC2D1B	-0.010834	1
DDX27	0.0499674	1
ZNF626	0.0463466	1
CNDP2	0.0082633	1
ARHGEF17	0.034421	1
GOLGA4	-0.012441	1
LEPR	0.0014791	1
TTC9	0.00771	1
TRBVB	-0.0085593	1
HMGA1P4	-0.0080247	1
CCDC159	0.0073336	1
NHSL2	0.0009435	1
SNX16	-0.0040048	1

PRC1	0.0123934	1
ADPRH	-0.0052147	1
INPP5E	0.0070973	1
NLN	-0.003075	1
BARD1	0.0033474	1
RIF1	0.0135317	1
ADNP2	0.0199554	1
FARP2	-0.0207258	1
ACER3	-0.0177237	1
SSR2	0.0599357	1
ZNF18	-0.0077513	1
TARBP1	0.0775623	1
SPTY2D1OS	-0.0167357	1
AL078587.2	0.0017796	1
EIF5	0.0404735	1
URB1	0.0072292	1
INCENP	0.0236461	1
LKAAEAR1	0.037855	1
AUXG01000	-0.0330646	1
LSM2	0.0621876	1
MINK1	-0.0069663	1
MICB	-0.0032872	1
KLHL12	0.0027396	1
BCAP29	0.0114636	1
AC245452.1	-0.0071009	1
NBEAL2	0.0369404	1
STK24	-0.0582095	1
DEF8	0.021824	1
PIGW	-0.0034039	1
PTPA	-0.0064335	1
CSNK2B	-0.0068447	1
BCLAF3	0.0114803	1
C9orf72	-0.0157358	1
MX1	0.0846154	1
FAM189B	0.0195282	1
NT5DC2	-0.0131534	1
TAOK1	-0.0672596	1
SARAF	-0.0964788	1
SH3BP5L	-0.0059625	1
MAGEH1	-0.0115278	1
AL590652.1	-0.0079099	1
RORA-AS1	-0.0326828	1
TIGD7	0.0019896	1
M6PR	0.0096135	1
FSCN1	0.019755	1
ANKRD11	-0.0700244	1
UBXN4	0.0473104	1
CLHC1	-0.0363194	1
MMAA	-0.0116894	1
EIF2AK3	-0.0100822	1

MEAK7	-0.004694	1
GPBP1	-0.0343441	1
MORF4L2-A	-0.0293724	1
ARL4A	-0.0671734	1
PLA2G15	0.0362797	1
OGT	0.0471044	1
RELL1	0.0114263	1
ANKMY1	-0.032479	1
PLD4	-0.0705873	1
KIF23	-0.0243828	1
STK32B	-0.0079232	1
IFT81	0.0164949	1
C4orf3	0.0412059	1
MEF2B	-0.0107049	1
NR3C1	0.0148787	1
CDC7	0.0031017	1
CDKL3	-0.0162272	1
FOXRED2	0.0251579	1
FOSL2	-0.0361412	1
PEMT	0.0431123	1
DPH2	0.0130514	1
REV3L	-0.0091816	1
ST13	-0.0923233	1
PHF3	0.0135656	1
LINC00174	0.0007763	1
C9orf40	0.0312714	1
MAP4K4	0.0263053	1
GPAT4	0.0202604	1
TMEM185A	-0.0055432	1
ZNF839	0.0254375	1
BCORL1	-0.0031747	1
AC012146.1	0.0002972	1
AC016065.1	0.0103889	1
ZFP30	-0.0135447	1
HIVEP1	-0.041758	1
SCAF8	-0.0393355	1
IMPACT	-0.0327138	1
PEAK1	0.0095256	1
ALDH16A1	-0.0174295	1
SREBF1	0.0347758	1
FDXR	0.0135703	1
ZNF77	0.0076423	1
HEATR6	0.0115807	1
ESF1	0.0053518	1
DNM1	0.0169344	1
IDE	-0.0283135	1
PCNT	-0.0452879	1
HMGN3	-0.0010772	1
SLC25A44	-0.0099182	1
ZNF829	0.0094222	1

AC011603.2	0.0003669	1
ZNF175	-0.0146742	1
DENND6A	-0.0624878	1
LRP12	0.0080076	1
HHLA3	0.0159174	1
PCM1	-0.0406546	1
NOP10	-0.0462689	1
SHF	0.0180974	1
HADHA	-0.0471331	1
HCFC2	0.0023642	1
TRIM44	0.0145303	1
CISD2	-0.0137051	1
HSF2	-0.0081839	1
NORAD	-0.0049274	1
SET	-0.0775588	1
SIRT3	-0.0050164	1
IFIT1	0.0553792	1
SC5D	-0.0195926	1
SPATA2L	2.658E-05	1
RNF217-AS1	-0.0032607	1
PRKAR1B	-0.017373	1
H2AFX	-0.0653541	1
RAD51B	0.0114911	1
AC012020.1	-0.0065234	1
NSMCE1-DT	0.0224391	1
DOHH	0.0197424	1
SMAD3	0.0229209	1
R3HCC1L	0.0060101	1
PQBP1	0.0123766	1
ZNF507	-0.0157007	1
RARS	-1.369E-07	1
INPP1	-0.0408712	1
MICA	-0.0093599	1
CASP10	0.0373756	1
PRKAR2B	0.02904	1
CXCL8	0.4079263	1
GSTK1	0.0643345	1
NAPA-AS1	0.0259563	1
DTNB	-0.0175392	1
MEGF8	-0.0094437	1
CEP350	-0.0874859	1
RBFADN	-0.0202764	1
GAS8	0.0084284	1
AC087239.1	0.0526653	1
TBC1D22B	0.0004835	1
MAP3K8	0.0236113	1
LDHB	0.1357534	1
AL022162.1	-0.0083502	1
ENY2	0.0486104	1
SLC16A1	0.0341441	1

AL132709.8	0.01606	1
HSD11B1L	-0.0045366	1
S100A1	0.0399291	1
CENPQ	0.0020324	1
AC007390.2	-0.0262453	1
SNRPB2	-0.0644344	1
KIF18A	0.0167684	1
TCEAL8	-0.0341644	1
ID2	-0.1115483	1
RPS26	-0.0547492	1
ALG13	-0.0552978	1
YJEFN3	0.0098377	1
AC016394.1	0.0020384	1
CCDC121	0.0213117	1
TTLL1	0.0295577	1
TANC2	-0.0276234	1
AC004803.1	-0.0226419	1
RBM39	0.1639785	1
AC092620.1	-0.0138362	1
NETO2	0.021026	1
AP3M2	0.0056635	1
SMYD5	-0.0031734	1
GPR146	0.0514145	1
EZH2	-0.0109453	1
AIFM2	-0.0009238	1
DNAJA4	0.0139978	1
BTRC	0.0047238	1
TRIM35	-0.0088132	1
AC005726.1	-0.0093502	1
ANKRD26	-0.0183651	1
ZNF219	-0.0068738	1
ZDHHC6	-0.0111319	1
TOMM6	0.0536716	1
SSU72	0.0026539	1
ZBTB38	-0.0247741	1
ASH1L	-0.0443912	1
DSE	-0.0124372	1
CEP78	0.0045117	1
AC093849.4	-0.0091528	1
GEMIN2	-0.0045312	1
ZNF85	-0.034726	1
LRRC45	0.0207864	1
AC026979.4	-0.0171491	1
STX18-AS1	0.0010849	1
MTHFSD	-0.0180654	1
HMGB3	-0.0851151	1
ZNF837	0.0002218	1
LAT2	-0.0960517	1
ARRDC1-AS	-0.0028875	1
MAP7	0.0313071	1

SH3RF1	0.0214564	1
AMPD2	0.0366974	1
ATP5MPL	-0.0911249	1
LRRC8C	-0.0310462	1
PRDX5	-0.0817558	1
RBM43	0.0372535	1
AC103691.1	0.0066865	1
ZNF74	-0.0130014	1
EXOC2	-0.0357732	1
AC026401.3	-0.0067536	1
ZHX1	0.0146285	1
SLC5A10	0.0463874	1
RIC8B	-0.0047544	1
SCRN1	0.0481203	1
GAPDH	0.1023499	1
ATRIP	0.0066914	1
TXNDC16	0.0067173	1
NXF1	-0.0069829	1
CKM	0.0091338	1
STBD1	0.0317106	1
KIZ	0.0244223	1
TMEM182	-0.0185185	1
TPCN2	-0.0195639	1
FAM200A	0.0048863	1
FNDC10	0.0299722	1
ITSN2	-0.0169154	1
SPART	-0.1362544	1
TTC8	-0.0148609	1
CAPRIN2	-0.0646876	1
GP1BB	0.2146806	1
CROCC	-0.0261229	1
KIAA0355	-0.0505867	1
INAFM1	0.0043398	1
ATAD3B	0.0256209	1
AC090559.2	-0.011922	1
AC000065.2	0.008685	1
SLC12A9	0.0407712	1
ATP5F1A	0.0218235	1
EDF1	0.1035671	1
HRAS	0.0279574	1
ALCAM	-0.0410422	1
DCLRE1A	-0.0088336	1
AL162253.2	-0.00303	1
ATXN7L3B	0.0999891	1
GPAM	-0.0007137	1
ERCC1	-0.0202283	1
DHX58	-0.0058881	1
DNHD1	0.0010157	1
CCDC142	0.0160681	1
LINC02482	-0.0292054	1

MYCBPAP	0.0158553	1
BORCS8	0.0153566	1
SRSF11	0.0833037	1
TRIM41	-0.0097981	1
ZNF526	0.0052762	1
SP4	-0.0165709	1
ENTPD5	-0.0059437	1
EIF3I	0.0688411	1
AREL1	-0.0308193	1
RBM26	-0.0433379	1
NREP	0.025464	1
ABLIM1	-0.0066889	1
LINC01137	0.0208136	1
AL158212.3	-0.0173821	1
POC1B-AS1	0.0101495	1
COQ10A	0.0101889	1
OSM	0.026922	1
DUSP5	0.042565	1
MSANTD4	0.0255785	1
RECK	0.0233529	1
RTL10	0.0189006	1
VPS52	-0.0138631	1
ZNF383	-0.0040237	1
ZNF225	-0.0053475	1
PINLYP	0.0121718	1
LCORL	-0.0110904	1
BET1L	-0.0131662	1
STXBP5	-0.0178391	1
AC130895.1	-0.0191559	1
CDKN2D	0.0436277	1
HSPA1B	0.0278702	1
APPL1	0.0462666	1
DARS2	0.0128242	1
MT-CO1	0.0535814	1
VPS54	-0.0511415	1
VEGFB	-0.019912	1
AC019069.1	0.0242294	1
ANXA2R	0.0330581	1
ZNF813	0.0050684	1
ZBTB2	-0.0160078	1
SLC49A4	-0.0036159	1
PUS7	0.0204043	1
AL359644.1	0.0010205	1
WDR83OS	0.0657286	1
TRIM16L	0.0016161	1
EML4	0.0009007	1
GSPT2	-0.0100354	1
ELL2	-0.0278558	1
TRMT2B	-0.0420827	1
ZNF30	-0.0125643	1

KBTBD11	0.0002097	1
EMP1	0.0772343	1
RBM8A	-0.0650588	1
LINC00662	-0.0213879	1
CNOT6L	-0.0110582	1
AC020658.5	0.0046342	1
ALKBH7	0.02342	1
DSTYK	0.0030584	1
ABHD17C	0.028106	1
DYNLT3	0.0169456	1
ARHGAP5	0.0035566	1
POU2F2	0.0679161	1
DZANK1	0.0135087	1
KNTC1	0.0371278	1
ADAMDEC1	0.0238047	1
AC006427.2	0.0012898	1
CD164	0.1906051	1
CACTIN	-0.0116398	1
RAB30-DT	0.0448421	1
RPLP0	0.0770737	1
TIPIN	0.0211529	1
ZNF236	-0.0197524	1
COX7C	-0.0539511	1
CSRNP2	-0.035265	1
ADAL	-0.0367841	1
GCNT1	0.0124598	1
TMEM120A	0.0022194	1
LY6G5C	0.0301387	1
ZBTB45	0.0063606	1
INO80E	-0.0029601	1
SMIM14	-0.0124031	1
AC124248.1	-0.007036	1
KIAA0556	0.0082773	1
AC127496.5	-0.0246745	1
SLC4A5	-0.0172	1
AL442125.1	-0.012443	1
PARD6B	0.0081411	1
MDGA1	-0.0051163	1
DGAT1	-0.0112293	1
EXOC8	-0.0266218	1
RECQL5	-0.0128395	1
ESD	0.0570102	1
AC090517.2	-0.0232966	1
TDRD7	-0.0054217	1
PSMA5	0.0102672	1
DNAH14	0.0016776	1
ZNF470	-0.0129104	1
LINC00910	-0.0264711	1
LDAH	-0.021429	1
CNNM2	-0.0345386	1

CDCP1	0.0164072	1
GIMAP8	-0.0099301	1
LSP1	0.0345707	1
PRPSAP1	0.0205548	1
DENND2D	0.0071127	1
ZNF513	0.003496	1
TRAPPC9	0.0107805	1
CHCHD2	0.0551975	1
AL391121.1	0.0043086	1
TTC38	-0.0014554	1
GAS5	-0.0730722	1
AFF1	0.0460738	1
RTRAF	0.0256847	1
KMT2A	0.0654806	1
RHOA	-0.0572276	1
ECT2	0.0152541	1
PRR14L	-0.0363805	1
ADAMTS6	0.0106709	1
HSDL1	-0.0121194	1
SMIM13	0.0019949	1
ZNF670	-0.0042029	1
HIST1H3H	-0.0647405	1
RETN	-0.0187049	1
TNRC6C-AS	-0.022321	1
RAB9B	0.0038123	1
ZNF296	0.0075164	1
SMIM26	-0.0623267	1
ARRB1	0.0211098	1
MOB2	0.0137631	1
RTL8A	0.011688	1
ZNF691	0.0099946	1
FIZ1	0.0037142	1
GIHCG	-0.0703626	1
TTC1	-0.0127695	1
THRAP3	0.0457474	1
SBF2-AS1	-0.0226806	1
DBI	0.0012126	1
MICALL1	-0.0107979	1
CCDC146	-0.0090114	1
RPAP1	-0.0098434	1
SPACA9	-0.009981	1
SOD1	0.0746627	1
CNNM3	-0.0005893	1
PRICKLE3	0.0099699	1
AL451165.2	0.0022437	1
ANP32A	0.0092039	1
GPR137	0.0083427	1
PDIA5	0.0410225	1
RTN4IP1	0.0067027	1
LINC02021	-0.0071915	1

CNEP1R1	-0.0082473	1
TMEM51	-0.019747	1
RPL5	0.0599546	1
NPM2	0.0342407	1
CAP1	0.0764238	1
PTP4A2	-0.1025974	1
RINL	0.0224945	1
POLR1E	0.037139	1
ABCA5	-0.0061221	1
GAN	0.000664	1
MACC1	0.0138186	1
SEC61G	-0.0112875	1
RGCC	0.0698213	1
TCP11L1	-0.0112147	1
ID1	0.0297558	1
AC012360.3	-0.0127944	1
SULF2	-0.0144579	1
ZNF726	0.003523	1
TSPAN13	0.0378123	1
RPS27L	0.0592322	1
DONSON	-0.0089353	1
AC119396.1	-0.0125285	1
PARP15	0.0162824	1
BRCA2	0.0283862	1
TYW5	-0.050123	1
SENP3	-0.0076626	1
MIR3936HG	0.0095686	1
GKAP1	0.0377086	1
ATP8B2	0.0343262	1
MSRB3	0.0616393	1
SMARCC1	-0.0276373	1
MAN2A2	-0.0702507	1
CLNS1A	0.035497	1
TSPAN17	0.0066495	1
SH3BP4	-0.0002701	1
OAZ1	-0.0848627	1
ENTPD3-AS	-0.0119439	1
TMEM53	0.0116758	1
MAST2	0.0040799	1
SCAPER	0.01699	1
LTA4H	-0.0356427	1
EIF3K	0.0765121	1
SRSF9	0.1014047	1
PPP1R12A	0.039609	1
TOP1	-0.0290466	1
CASTOR3	0.0012644	1
DUS2	-0.0100028	1
TGOLN2	-0.0165447	1
EARS2	0.0053411	1
AL121944.1	-0.0030825	1

TBCA	-0.12677	1
APOL3	0.0030682	1
CCL28	0.0336617	1
TMBIM4	-0.0136695	1
FBXL12	9.929E-05	1
AC004687.1	0.073143	1
SCAF11	-0.0263326	1
PRPF40A	0.0367348	1
PYROXD2	-0.0127492	1
PHF2	-0.0240918	1
AC018647.2	-0.0099087	1
MSH5	0.0139185	1
LINC01176	-0.0205699	1
ACVR1	-0.0399259	1
OR2L2	0.0160114	1
CCNA1	0.0072239	1
PEX11A	0.0146744	1
KIF1BP	0.0378069	1
DVL1	0.0043928	1
ARHGAP23	0.0244388	1
PIK3C2B	0.0368621	1
FCGRT	-0.0227149	1
ITM2B	0.0676438	1
CUTA	-0.0069988	1
WDYHV1	-0.0081157	1
ZNF17	-0.0047136	1
VDAC1	0.044386	1
LONRF1	0.0153108	1
RAD52	-0.0264497	1
SASS6	-0.0374757	1
ARIH2OS	-0.0282784	1
EMILIN1	0.0375428	1
TAF11	0.008898	1
VAC14	-0.0082716	1
TCTEX1D2	0.0169522	1
MVB12B	-0.0161867	1
ZNF14	-0.0027533	1
AL592295.5	0.0015819	1
MFSD13A	-0.0093054	1
VILL	0.0002874	1
MED24	-0.0073815	1
CEP250	-0.0023878	1
CD74	0.0691956	1
BBS7	-0.0155319	1
EIF4A1	0.0920591	1
JMJD4	0.0269133	1
DNAJC2	0.0538592	1
AC004540.1	-0.0294038	1
PPIB	0.1166812	1
TIAM2	-0.0109502	1

ACRBP	0.017051	1
AL035446.1	0.0979576	1
OTUD1	0.0080832	1
RYK	0.0606712	1
AGMAT	-0.0016199	1
ATG9A	0.0316561	1
RPRD1B	0.0098236	1
C10orf88	0.0025268	1
ZNF749	0.0108783	1
C14orf93	-0.0042502	1
AC008771.1	-0.0066223	1
CNPY4	-0.000585	1
ZBTB3	0.0050437	1
TRMT1L	0.0066117	1
TRDMT1	-0.0169803	1
FAM24B	0.0038269	1
CTSW	0.0262064	1
PCF11	-0.0910888	1
NPEPL1	0.0161829	1
ESS2	0.007433	1
MED20	0.013618	1
UBE2I	-0.0336004	1
TBC1D14	0.0189847	1
BRMS1L	-0.0136721	1
FZR1	-0.0054156	1
NUDT12	0.0281492	1
SCLY	0.0115015	1
EEF1AKMT1	-0.0029081	1
PRPF40B	-0.0062926	1
TRIM26	0.0008956	1
CASTOR1	0.0041356	1
CDPF1	0.0114251	1
MIGA2	-0.006712	1
AL355377.3	-0.0159822	1
TRMT61A	0.0161914	1
FAM131A	-0.0015432	1
MYCBP2	-0.0068669	1
CCDC171	-0.0099586	1
LINC01473	-0.008395	1
CA3-AS1	-0.0095371	1
CA13	0.0022578	1
SLC27A3	0.0313081	1
C16orf46	-0.0041589	1
SGK3	-0.0466019	1
LARP7	-0.0887366	1
RPL13A	-0.0440454	1
CRTC3	-0.0292823	1
CTSA	-0.0204399	1
PDCD4-AS1	-0.0200759	1
PNMA6A	-0.0050815	1

CCDC149	-0.0309531	1
SEC24D	-0.0118741	1
PLXNA3	0.0077642	1
CEP162	-0.0360936	1
C4orf33	0.0162319	1
ZNF710-AS1	-0.0403727	1
CEP295	-0.0315863	1
RAB40B	-0.0224543	1
PLCL2	0.0012224	1
FAM161A	0.0083989	1
PIK3AP1	-0.0171267	1
MICALL2	-0.0188083	1
TRIR	0.0391231	1
GNPTAB	0.0110688	1
NDUFB1	-0.0919764	1
LINC02470	0.0515233	1
METTL2A	0.0148753	1
SLC25A6	-0.0689026	1
MIER1	-0.0237869	1
ATP1A1-AS1	-0.0008209	1
TKT	-0.0733888	1
DYRK2	0.0086164	1
PHYHD1	-0.0229632	1
IFT140	-0.0004562	1
SMIM15	0.0313384	1
KIF13B	0.0149902	1
IGF2BP3	0.0481237	1
COMMD6	-0.0438105	1
TRMT11	0.015339	1
GMPR	-0.0258438	1
ANKRD12	-0.0296672	1
TULP3	0.0114558	1
PPARGC1B	0.0025273	1
MYO18A	-0.000106	1
CLDND1	0.0533232	1
MSN	0.0397758	1
AL591845.1	-0.0017799	1
STAM	-0.0279523	1
AC027279.1	-0.0059504	1
EOGT	-0.0121005	1
DSC2	0.0520874	1
SLC35C1	-0.0109899	1
HNRNPA2B1	0.1301545	1
AC004943.2	-0.0009064	1
ENKD1	0.0088468	1
NME2	0.1099275	1
TNRC6C	-0.0198438	1
ATP5PD	-0.0391217	1
AC091057.6	-0.0099108	1
SLC25A3	0.0809087	1

ADAMTSL4-	0.043526	1
VARS2	0.0013701	1
PSMA2	0.0363536	1
ARFIP1	0.0101703	1
IL4R	0.0135744	1
SRGAP2B	-0.011423	1
NEXN	-0.0494856	1
RTP4	0.025751	1
IQSEC3	-0.0003891	1
ZHX3	0.017231	1
COX6C	-0.0677185	1
UXT	0.0123246	1
ZNF763	-0.0023377	1
AC091729.3	-0.0141172	1
MTBP	-0.0115425	1
AL117332.1	0.0051474	1
ZNF880	-0.0120488	1
C18orf54	-0.0198405	1
ARRDC3-AS	-0.0205645	1
RNF25	0.001393	1
TCEAL9	-0.0616529	1
TMEM181	0.0026247	1
ZNF284	-0.0178656	1
MARCKSL1	-0.0612795	1
ALG1L2	-0.0183287	1
C3orf35	-0.0132256	1
ALG12	-0.0123124	1
OST4	0.0979949	1
PRR3	-0.0065115	1
XAF1	-0.0372324	1
HIGD2A	0.0505405	1
CENPL	-0.006863	1
HMGN2	0.0431221	1
VANGL1	-0.0039008	1
AC095057.3	-0.0230262	1
SPRYD3	0.0052922	1
SLC1A3	-0.0308884	1
PTGES3	-0.0655482	1
CPVL	-0.1238028	1
AC009812.1	-0.0135608	1
EMP3	-0.0142608	1
TIPARP-AS1	0.0157977	1
LINC02352	-0.0357731	1
BTN2A2	0.0066784	1
HINT1	0.0348746	1
VPS33B	0.0089302	1
C21orf58	0.0145832	1
HIST2H2AA	-0.0330751	1
ELANE	0.0469829	1
FOCAD	0.0024897	1

MT-ND4	0.0630676	1
GALNT6	-0.0027661	1
SEC22A	-0.0066002	1
JTB	0.0296996	1
TRPV1	-0.0085637	1
AEN	0.0021739	1
EML2-AS1	-0.0129687	1
HIST1H2AK	-0.0459868	1
PEX7	-0.0086204	1
CNTRL	0.0644417	1
ARHGAP12	0.0146628	1
ZNF700	-0.0230321	1
NDUFC1	-0.054986	1
ANAPC11	0.0431809	1
SPHK2	-0.0147504	1
SFXN1	0.0433115	1
U2SURP	0.0210306	1
DDX46	-0.0086444	1
RPS23	0.056027	1
ANKRD18B	0.0327729	1
APOM	0.0009147	1
CYP2U1	0.010518	1
IFIT2	0.0296525	1
IPO4	0.0206017	1
SVOPL	0.0263535	1
MED22	0.0057205	1
RCAN1	0.0190382	1
SUN1	0.0056089	1
TRAPPC5	-0.0366206	1
AURKA	-0.0148591	1
TLN1	-0.0290472	1
AC145285.6	0.0268899	1
ZFYVE16	-0.0571848	1
DHRS13	-0.005843	1
EIF3L	-0.0345602	1
TRIM66	-0.0043276	1
KNSTRN	0.0121393	1
EIF1B	-0.0051567	1
SLFN12	-0.020903	1
IFT46	-0.0004348	1
ZNF624	-0.0047036	1
MIEF2	0.0052981	1
CHCHD6	0.0354213	1
HIST1H4H	-0.0220324	1
CNNM4	0.023709	1
CEBPD	-0.0293695	1
PURA	0.0432074	1
WDR31	0.0060598	1
CHURC1	0.073228	1
AC004812.2	-0.0015244	1

TRIM61	0.0196215	1
KIAA1549	-0.0131703	1
YY1	-0.0285179	1
GEMIN5	0.0340656	1
ZNF771	-0.0011737	1
MIR646HG	0.0182269	1
SRSF6	-0.0825298	1
WDR19	-0.028917	1
TRAPPC1	0.0719086	1
PHLDB3	0.0189679	1
MAP11	-0.0113819	1
TAF1C	-0.0068545	1
SLC15A2	0.011553	1
SHANK3	0.0441512	1
AC016949.1	-0.0058467	1
TYROBP	-0.0799854	1
BNIP3	0.0284882	1
PLEK	0.0301822	1
SNHG8	-0.0572731	1
UQCRQ	-0.0469945	1
RPL12	-0.0513509	1
B3GALT4	0.0123671	1
KIF16B	0.0151219	1
HIST1H2BE	0.005689	1
RSRC2	-0.0355142	1
ZFYVE28	0.0178057	1
RAP1B	-0.0461071	1
BBS9	-0.0008583	1
ABCD3	-0.0112007	1
AL139260.1	-0.0121693	1
SNX24	-0.0172221	1
POLK	-0.0162278	1
THG1L	0.0229804	1
TSC22D1-AS	-0.0018056	1
CD3EAP	0.0061436	1
PCMTD1	0.0435437	1
RGL4	0.0182206	1
ACTR3	-0.023865	1
ADAM9	-0.0063882	1
HNRNPM	0.0375591	1
FNTA	-0.0170285	1
SLC37A4	0.0048969	1
TRIT1	0.0169795	1
RNASEL	0.0039775	1
AC020656.1	-0.0591515	1
EDC3	-0.0087291	1
COX7B	0.0035452	1
MFSD9	0.0022021	1
MYOZ3	0.0331738	1
CCPG1	0.0388754	1

ACADSB	-0.0232807	1
SUV39H2	0.0092689	1
DNA2	-0.0052744	1
NBPF3	-0.0248775	1
ZBTB8A	0.0176253	1
RNASEH2B	0.0221731	1
GORAB	-0.0081244	1
AK9	-0.0334324	1
SERBP1	0.1159249	1
LBH	-0.1010279	1
CEP83	-0.0285701	1
STK26	-0.0216828	1
MED26	0.0041926	1
PIGQ	-0.0106492	1
C9orf85	0.0148524	1
STAR	0.0139722	1
FAM221A	-0.0093801	1
HCP5	0.0139979	1
ZBTB7B	-0.019245	1
AOAH	0.0031876	1
VDAC2	0.0096362	1
PFKFB3	-0.0550641	1
UGCG	-0.007969	1
EZR	0.0391111	1
GPR180	0.008452	1
SNX8	0.0186323	1
SMPD2	-0.0051765	1
PAM	0.0211489	1
BUB1	0.0225132	1
RPL23A	-0.0345757	1
RPS27A	0.0325299	1
ITGA6	0.0623046	1
PDZD11	0.0054214	1
RAB11B-AS:	0.0144651	1
AC147067.1	-0.0099383	1
MAPK8IP3	-0.0140365	1
DHX30	-0.0451074	1
AL133523.1	0.0310796	1
SNAPC1	0.0109	1
MYLIP	-0.0229858	1
LINC01004	-0.0143268	1
SRRM2	0.0749892	1
IKZF4	0.0171961	1
DCLRE1B	-0.0058778	1
RIPK3	-0.0045841	1
NDUFC2	-0.0311188	1
PHACTR2	-0.0106217	1
CSTA	-0.0362041	1
CSNK1A1	0.0309691	1
SRSF3	0.0230092	1

HYLS1	-0.041783	1
SLFN5	-0.0337635	1
TLR4	-0.0102065	1
AC009962.1	-0.0088883	1
LIN7B	-0.0223963	1
PHIP	-0.0399657	1
Z95331.1	-0.0049536	1
ZNF189	-0.0279543	1
CTU2	0.0215255	1
SYNJ1	-0.013511	1
BTN3A3	-0.0140888	1
C11orf95	-0.0090274	1
C7orf25	-0.0063262	1
SFXN2	-0.0013173	1
KMT5C	0.009792	1
USP27X	0.0143403	1
TCTA	-0.0569986	1
COG6	-0.0315346	1
WDFY4	-0.0028986	1
DIABLO	-0.0067057	1
AL049840.5	-0.010869	1
KLF11	-0.0065021	1
MVK	-0.0012453	1
UFC1	0.0299285	1
GK	-0.0257774	1
AIMP1	0.0071905	1
FIRRE	0.0337577	1
STK39	0.0005081	1
NDUFB4	0.0446278	1
PRDM15	0.0090909	1
ALDOC	-0.0169098	1
MRPS21	0.067808	1
SLA	0.0550905	1
MFSD6	0.0021661	1
ATF7IP	-0.0233266	1
LYZ	-0.0561914	1
FBXO21	0.0178171	1
LRRCC1	0.0402061	1
AL645728.1	0.0228672	1
P4HA1	-0.0208093	1
VPS13A	-0.0321038	1
ZCCHC4	-0.0027322	1
LINC00884	0.0030633	1
C17orf75	0.0301649	1
CABLES1	-0.0027291	1
RPL14	0.0570658	1
AL359643.3	-0.0060686	1
NAA16	-0.0183077	1
VAMP1	-0.0103223	1
CCL5	0.028285	1

MT-ND5	0.0947813	1
MT-ND1	0.0242271	1
PTAFR	0.0391762	1
PLD6	-0.0074242	1
ENGASE	-0.0019749	1
IFT88	0.0309637	1
FAAP24	-0.0114349	1
SRRM1	-0.0677406	1
HELLPAR	-0.0046736	1
AC015871.1	-0.0023468	1
RWDD1	-0.0416896	1
NOD1	-0.0031865	1
ZNF607	0.0126449	1
SLC25A22	0.0038061	1
ANTXR2	0.0166989	1
WDCP	0.0027441	1
ELOB	0.0231531	1
LANCL2	0.0015088	1
SLC25A45	-0.0077615	1
HOOK3	0.0310561	1
SELENOH	0.0726163	1
PPP1R3B	-0.0103541	1
ARPC1B	0.0454694	1
TRIM69	0.0183622	1
ZBED3-AS1	0.0134174	1
KHDC1	0.0086388	1
UBXN1	-0.0038917	1
PINX1	0.0086308	1
ZNF142	0.0034455	1
AL356804.1	0.058577	1
HLA-DRB5	0.0650999	1
MBNL1-AS1	0.0023753	1
NACA	-0.0059188	1
ZNF25	-0.0200267	1
VAMP8	-0.0705053	1
ERP29	-0.0188932	1
LDHA	0.0640316	1
PGK1	0.016354	1
TEDC1	0.0163763	1
AC040970.1	0.0101542	1
BRPF3	-0.0144781	1
MAP1LC3A	0.0029574	1
LAPTM4B	0.0036768	1
MAP2K6	-0.0092555	1
TIGD6	-0.0055703	1
VIM-AS1	0.0018695	1
C11orf24	-0.0165889	1
RBM25	-0.010483	1
HNRNPA1	-0.0229583	1
NSMF	-0.0005224	1

HIST1H2AG	0.0191281	1
CCDC34	0.0558794	1
POLR3G	0.0186508	1
NYNRIN	0.018938	1
ATP5MC2	0.0345476	1
FAM126A	-0.0263632	1
TTYH2	0.0292923	1
CCDC47	-0.0224405	1
PIGV	0.0078745	1
USP51	0.0208984	1
AMMECR1	0.0048166	1
SEM1	-0.0341541	1
AC015982.2	-0.0153769	1
AP001505.1	-0.011329	1
BASP1	-0.0126935	1
VASP	0.0230375	1
ENPP2	0.0506011	1
ST6GAL1	-0.0029593	1
FKBPL	0.012853	1
AC092944.1	0.0151368	1
APEX1	-0.0191561	1
TMEM258	-0.0305098	1
C2CD2L	-0.0083908	1
ZNF280B	-0.0019551	1
PFDN5	-0.0416982	1
AC099778.1	0.0148969	1
NR1D1	0.0044523	1
HLA-DQA2	0.0162827	1
LINC00173	0.0337422	1
TALDO1	-0.0532619	1
LCMT2	-0.0122375	1
CHST10	-0.0020151	1
LYL1	0.0119346	1
SLC27A1	0.0098815	1
RAC1	0.0041745	1
PPDPF	-0.0269882	1
PGM3	-0.0169811	1
S100A13	0.0009127	1
CCDC77	-0.0146544	1
SMC4	0.1493751	1
ACTR5	0.0011453	1
ZNF584	0.0200826	1
KIF3A	0.0097014	1
RBIS	-0.0661237	1
RAMP1	-0.033817	1
CCDC26	0.0428682	1
PDSS1	0.0003077	1
SLC4A7	0.0095644	1
HP1BP3	-0.0062529	1
WASF1	-0.0028386	1

JHY	-0.0008835	1
DANT2	0.002995	1
BTG1	-0.0074386	1
AGAP3	0.0157564	1
PTPN6	-0.0964332	1
CCT8	0.0518369	1
YWHAB	-0.0278315	1
HNRNPU	0.1141141	1
RTF1	0.0501249	1
DXO	0.0163255	1
AC246817.2	0.0078271	1
C1orf112	-0.0079741	1
AKAP9	-0.082341	1
ITM2A	0.0096253	1
LINC00923	0.0100028	1
ICK	-0.0066333	1
CCDC169	0.0038479	1
PDCD2L	0.0089101	1
GSTO1	0.015538	1
GCC2	-0.0399101	1
ATP5F1B	0.0541852	1
NAGA	0.014996	1
GIN1	-0.0200973	1
CALCRL	-0.0268031	1
SRD5A3	0.0170731	1
GPN2	-0.0197469	1
IQGAP1	0.0331872	1
TRMT9B	-0.0173565	1
SLC4A2	-0.001775	1
UBL5	-0.0321791	1
CALM2	0.0345653	1
TCEAL3	-0.0084451	1
IER5L	0.0017281	1
EIF3F	-0.0144044	1
NEFH	0.0162014	1
GBP5	0.0057359	1
HLA-DMB	0.0271872	1
GPR141	-0.0013914	1
ECH1	0.015058	1
ATP5MD	-0.0323683	1
RNF144B	0.0146749	1
SUB1	0.0348576	1
DSCC1	0.0078551	1
POGLUT2	0.014718	1
KANSL1-AS:	-0.0026731	1
CEP290	0.0129735	1
GABARAP	-0.0552718	1
RPL36	-0.0285494	1
TPT1	-0.0235706	1
STK19	-0.009052	1

HLCS	-0.0030989	1
ZBTB17	-0.0070508	1
FNIP2	0.0057553	1
RPP25	0.0194505	1
HIST1H4C	0.2380557	1
LRRFIP1	0.0283794	1
CKB	0.0121438	1
ARF4-AS1	-0.0164341	1
PHLDA1	0.0104982	1
SRR	0.0115579	1
CEP97	0.000973	1
POFUT2	-0.0005189	1
NPM1	0.0796596	1
SND1-IT1	-0.0082219	1
PTMS	0.0250804	1
VWA1	0.0290739	1
AVEN	-0.0211505	1
MFGE8	0.0292374	1
LRRC8A	-0.0077316	1
MBNL1	-0.0543194	1
DBF4B	-0.0030876	1
AC002467.1	-0.0191005	1
CLDN10	0.0333366	1
CTBS	-0.007659	1
IL18	0.1039681	1
CYSLTR2	-0.0114727	1
LINC01515	0.0192699	1
STARD7-AS	-0.0168252	1
LFNG	0.0351124	1
CCSER1	-0.0074665	1
TMEM201	-0.0026923	1
ZNF710	-0.0158322	1
GALM	-0.041965	1
SLC16A1-AS	0.0446236	1
PRMT6	0.0160373	1
RPL11	-0.0214836	1
PITPNC1	0.0037112	1
TRAF3IP1	-0.0216934	1
GABPB1-AS	-0.0080545	1
B2M	0.033424	1
YTHDC2	-0.0038812	1
ATP6V0E1	0.0195764	1
STX2	-0.004411	1
ZNF143	-0.0188306	1
CCDC15	-0.0169349	1
DNAJC1	0.0622044	1
BRI3	-0.0149592	1
LMNA	-0.0014177	1
POLR2L	-0.0185851	1
GOLGB1	-0.019971	1

BAIAP2	-0.0372325	1
MSI2	-0.0559181	1
ATP5PO	0.0243364	1
MMD	0.0107517	1
PARP16	-7.042E-05	1
SEC61B	0.0152592	1
TCEA1	0.0165534	1
PNN	0.0316459	1
GNB1L	0.0004253	1
UBA52	0.0078797	1
SAP18	0.0476449	1
PRELID1	0.0083979	1
AMDHD2	0.0248703	1
COX6A1	-0.0403503	1
ZBTB39	0.0008129	1
CST3	0.0412744	1
CTSH	-0.0431248	1
AFF3	0.0158999	1
TOMM20	0.0133759	1
AL050341.2	-0.002966	1
RPS25	-0.03398	1
PRSS57	-0.0277351	1
SNRPD2	-0.0289128	1
RPL23	0.0500212	1
POP1	0.0086335	1
CCDC112	0.0260226	1
CDK6	-0.0642994	1
RPL21	0.0371859	1
HLA-B	0.1085008	1
GAS2	-0.0250117	1
SFMBT2	0.0138321	1
P2RY1	0.0293157	1
ARPC3	-0.018789	1
AL358780.1	-0.003044	1
AL360012.1	0.0177284	1
SRSF8	-0.0070438	1
C11orf58	-0.0401114	1
PUS7L	-0.0226638	1
BANK1	0.0186315	1
SRSF5	0.0577991	1
MIR4453HG	-0.0047429	1
SERP1	0.0222933	1
PLB1	0.0205407	1
HLA-DQA1	0.0048432	1
DENND5B	-0.0095961	1
SLC27A4	-0.0082527	1
HERC5	0.0020723	1
LUC7L3	-0.0188073	1
RPS13	0.0060482	1
CPA3	-0.0038542	1

HAUS8	0.0009958	1
MT-ND6	0.0475949	1
TMEM123	-0.0463647	1
AC091271.1	0.0257747	1
ARHGDIB	-0.0304505	1
HIST2H2BF	-0.0216758	1
RACK1	0.0387841	1
C9orf16	0.0274012	1
NAP1L1	-0.0007142	1
LGALS1	-0.0559139	1
SUMO2	0.0838156	1
UQCR11	0.0282192	1
NQO1	-0.0054465	1
HMGN5	-0.0144757	1
ZFP36L2	0.0716183	1
ZFHX3	-0.0182783	1
DOK2	-0.0143143	1
POLR1D	0.0185741	1
HGH1	0.0127954	1
RPL24	0.0086131	1
FAU	-0.0251454	1
RSL24D1	-0.058491	1
NDUFA4	-0.0501998	1
CCNI	-0.0229572	1
IL13RA1	-0.0235088	1
ATP6V1G1	-0.0072954	1
RPL26	0.0096471	1
TAGLN2	-0.0085479	1
RPL22L1	0.0094407	1
NOP53	0.0131499	1
RPL7	0.0204999	1
RPS3A	-0.0105505	1
SLC9A1	0.0067296	1
EIF4A2	0.0225406	1
AC069185.1	-0.0112805	1
SYNJ2	-0.0024185	1
RPS17	0.0037707	1
UQCRH	0.0256837	1
OSTC	-0.0080112	1
ATP5PF	0.0230053	1
COMT	0.0213853	1
SRP14	-0.0336582	1
BTF3	0.0541633	1
RHOB	-0.0129555	1
RPL36AL	0.0284769	1
UBB	-0.0157248	1
ARPC2	-0.0348333	1
EEF1A1	0.0180658	1
SERPINB1	-0.0061256	1
RSPH3	0.0101571	1

RSL1D1	0.0609817	1
TEX9	0.005764	1
SERPINI1	-0.0213082	1
DDX17	-0.0047254	1
S100A10	0.0152342	1
AL157895.1	-0.084611	1
ABRACL	-0.0040396	1
ARGLU1	0.0458557	1
PRRC2C	-0.0171143	1
RPL35	0.0200478	1
PFN1	0.0030059	1
FXYD5	-0.0196299	1
HNRNPC	0.0078814	1
FGL2	-0.0600085	1
HMGA1	-0.0126186	1
COX8A	-0.0191155	1
SLC25A5	0.0284587	1
MYOF	0.0033097	1
RPS12	0.0013285	1
TMSB10	-0.0251127	1
MT-CO2	0.0039174	1
MT-ND3	0.0025674	1
CFL1	0.0076509	1

· cells after niche-like culture versus after standard culture (reference). Only genes exp

ɒressed in >10% of single cells are reported. Log2 Fold Change (FC) and FDR-adjusted

p-value.
